# Bellidifolin Improves Pulmonary Artery Smooth Muscle Cells Proliferation by Targeting the IGFBP5-Mediated PI3K-AKT-mTOR Pathway and Dilates the Pulmonary Artery

**DOI:** 10.3390/biom16071059

**Published:** 2026-07-19

**Authors:** Qiuqin Hu, Hongmai Wang, Yujie Qiao, Qingqing Xia, Jiacheng Hu, Xiangyun Gai, Yulin Li, Tao Chen, Zhanqiang Li

**Affiliations:** 1 Department of Pharmacy, Qinghai Minzu University, Xining 810007, China; 19899704821@163.com (Q.H.); wmm510921@163.com (H.W.); qiaoyujie1212@163.com (Y.Q.); xqing97@163.com (Q.X.); huxiansheng621@163.com (J.H.); 2 Northwest Institute of Plateau Biology, Chinese Academy of Sciences, Xining 810008, China; liyulin@nwipb.cas.cn (Y.L.); chentao@nwipb.cas.cn (T.C.); 3 Plateau Medicine Research Center, Qinghai University, Xining 810016, China; zhanqiang_li@163.com

**Keywords:** pulmonary hypertension, Bellidifolin, pulmonary arterial constriction, pulmonary arterial remodeling, pulmonary artery smooth muscle cells proliferation

## Abstract

Pulmonary hypertension (PH) is a progressive disease that severely compromises right ventricular function, characterized by two major pathological features: pulmonary arterial constriction and pulmonary arterial remodeling. Bellidifolin (BEL), a natural ketone compound, exhibits potent anti-inflammatory and antioxidant effects; however, its role in PH remains unexplored. This study evaluated the impact of BEL on the two pathological processes: pulmonary arterial constriction and pulmonary arterial remodeling. First, the effects of BEL on pulmonary arterial constriction were evaluated using wire myography. The study revealed that BEL (6–96 μmol/L) inhibited the contraction response of intact endothelial and denuded pulmonary arterial rings precontracted with norepinephrine (1 μmol/L) in a concentration-dependent manner. BEL (90 μmol/L) suppressed pulmonary constriction induced by intracellular calcium release and extracellular calcium influx. In cellular experiments, BEL inhibited the proliferation, migration, and phenotypic transformation of pulmonary arterial smooth muscle cells (PASMCs) induced by 10 μmol/L CoCl_2_ (72 h), as evidenced by upregulation of the contractile phenotype markers α-SMA and SM22α and downregulation of the synthetic phenotype markers OPN, vimentin, and PCNA. Multi-omics analysis identified Aldh1A1, Mgp, Col4a6, and Igfbp5 as significantly enriched candidates. Among these candidates, Cellular Thermal Shift Assay demonstrated that BEL enhanced the thermal stability of Igfbp5 in PASMCs, suggesting that Igfbp5 may be a potential direct target of BEL. Moreover, KEGG pathway analysis revealed significant enrichment of the PI3K-AKT-mTOR pathway, which is known to be involved in cell proliferation and is regulated by Igfbp5. BEL may inhibit the PI3K-AKT-mTOR pathway by suppressing Igfbp5. The results indicate that BEL may regulate Igfbp5 to inhibit the PI3K-AKT-mTOR pathway, thereby exerting anti-proliferative and inhibitory effects on migration and phenotypic transformation.

## 1. Introduction

Pulmonary hypertension (PH) comprises a group of life-threatening pulmonary vascular diseases characterized by abnormal pulmonary arterial constriction and pulmonary arterial remodeling, leading to increased vascular pressure and resistance, resulting in right ventricular hypertrophy and eventual death from right heart failure [1,2,3,4].

The prevalence of PH among individuals aged over 65 years worldwide is 10%; these patients have a poor prognosis and high rates of hospitalization and mortality [5]. Drugs currently used in clinical practice for treating PH, such as bosentan (an endothelin receptor antagonist) and sildenafil (a phosphodiesterase-5 inhibitor), mainly improve symptoms by dilating pulmonary blood vessels [6]. However, they have only minor effects on pulmonary arterial remodeling, and treatment outcomes for PH remain unsatisfactory [7]. Drugs that simultaneously and effectively improve abnormal pulmonary arterial constriction and pulmonary arterial remodeling are lacking in clinical practice. Pulmonary arterial remodeling involves the thickening of the vascular intima/media due to the excessive proliferation, migration, collagen deposition, and decreased apoptosis of pulmonary artery smooth muscle cells (PASMCs) [8].

The transition of PASMCs from a contractile phenotype to a synthetic phenotype plays a crucial role in the pathogenesis of PH [9]. Phenotypic modulation of PASMCs is characterized by changes in the expression levels of several marker proteins, including α-SMA, SM22α, and vimentin. A decrease in contractile markers and an increase in synthetic markers in PASMCs are generally considered hallmarks of phenotypic switching [10]. As an important marker of PASMCs, α-SMA plays a role in skeletal support and regulation of cell contraction in highly differentiated PASMCs and is highly expressed in PASMCs exhibiting a contractile phenotype. Similarly, SM22α is specifically and highly expressed in contractile PASMCs. In healthy blood vessels, these contractile or differentiated contractile cytoskeletal proteins are abundantly expressed in vascular smooth muscle cells, which exhibit very low proliferation activity [11,12]. However, during phenotypic switching, the expression levels of α-SMA and SM22α decrease or may even disappear, resulting in the loss of contractile function [13]. Dedifferentiated PASMCs acquire enhanced proliferative, migratory, and invasive capabilities. They also produce large amounts of extracellular matrix and specific proteins, including matrix metalloproteinases (MMPs) and osteopontin (OPN). Proliferation-related proteins, such as proliferating cell nuclear antigen (PCNA) and cyclin D1, are abnormally highly expressed [13]. In postnatal individuals, vimentin expression is restricted to endothelial cells, fibroblasts, and smooth muscle cells. However, vimentin can be re-induced in many cell types cultured in vitro, and its expression is associated with cell migration, cell contraction, and cell-extracellular matrix interactions [14]. OPN is a multifunctional extracellular matrix protein involved in various biological functions. Many stimuli, including cytokines, reactive oxygen species, and hypoxia, can promote OPN expression [15]. Studies have shown that increased OPN expression promotes the proliferation and migration of human coronary arterial smooth muscle cells [16]. In animal models of hypertension, elevated OPN expression in vascular smooth muscle cells promotes MMP-2 production, thereby facilitating vascular remodeling [17]. Pulmonary arterial remodeling is the major cause of PH, and its reversal is fundamental to improving patient survival and quality of life. Therefore, new drugs capable of inhibiting both abnormal pulmonary vasoconstriction and pulmonary vascular remodeling are urgently needed.

The PI3K/Akt/mTOR signaling axis is involved in various key biological processes, including cell growth, proliferation, differentiation, migration, and apoptosis [18]. It is also one of the key signaling pathways regulating cell proliferation and survival. Moreover, activation of the PI3K/Akt/mTOR pathway promotes pulmonary vascular remodeling through multiple downstream mechanisms. Activated Akt and mTOR increase the expression of proliferation-related proteins, such as cyclin D1 and c-Myc, thereby accelerating cell cycle progression of PASMCs from the G1 phase to the S phase and promoting their proliferation [19]. Akt activation can also phosphorylate and inhibit pro-apoptotic proteins, including the members of the Bcl-2 family and forkhead box O (FOXO) transcription factors, thereby reducing the PASMC apoptosis and allowing abnormal cell proliferation to persist [20]. The PI3K/Akt pathway is also involved in regulating cytoskeletal rearrangement and the expression of adhesion molecules, thereby promoting PASMC migration from the vascular media to the intima and contributing to vascular wall thickening [19]. The transition of PASMCs from a contractile to a synthetic phenotype is a critical step in pulmonary vascular remodeling. Activation of the PI3K/Akt/mTOR pathway promotes synthetic PASMC characteristics, including increased Extracellular Matrix (ECM) synthesis and secretion, which ultimately leads to vascular wall fibrosis and stiffening [19]. Given the central role of the PI3K/Akt/mTOR pathway in PH pathogenesis, inhibitors targeting this pathway are considered promising therapeutic strategies for PH.

Bellidifolin (1,5,8-trihydroxy-3-methoxyxanthone, BEL) is a natural tetraoxygenated xanthone with a molecular formula of C_14_H_10_O_6_ and a relative molecular weight of 274.22 [21]. BEL has strong pharmacological activity in many aspects, primarily including cardiac cardioprotection, cytoprotection against cell damage, and inhibition of cell proliferation [21,22]. Li et al. combined various biological experiments, network pharmacological techniques, in vitro analysis, and molecular docking studies reported that BEL regulates the expression of STAT3 and the protein activity of COX-2, consequently effectively inhibiting the proliferation of A549 cells in a time and concentration-dependent manner [21]. Zhou et al. reported that BEL inhibits the NADPH oxidase 4 (Nox4)/reactive oxygen species (ROS)/a disintegrin and metalloproteinase (ADAM17) signaling pathway in a bromodomain-containing protein 4 (BRD4)-dependent manner, thereby improving isoproterenol-induced myocardial hypertrophy. Moreover, BEL alleviates myocardial hypertrophy and improves cardiac dysfunction by regulating the BRD4/Nox4/ROS signaling pathway [22].

Aerobic glycolysis, also known as the Warburg effect, was discovered by Warburg in malignant tumor cells, indicating that the glycolysis pathway significantly increases under aerobic conditions, manifested as increased glucose uptake and lactic acid production [23,24,25]. In patients with PH and in animal models of PH, PASMCs, pulmonary artery endothelial cells, fibroblasts, and right ventricular cardiomyocytes undergo a metabolic shift from mitochondrial oxidative phosphorylation to enhanced glycolysis. This metabolic reprogramming promotes excessive cell proliferation and resistance to apoptosis, thereby contributing to pulmonary vascular remodeling and disease progression [26,27,28]. Three newly identified pro-cancer molecules, including ENO1, AK4, and PKM2, are upregulated in PH models and drive disease progression through interconnected signaling pathways involving HIF-1α, Akt, AMPK, and NF-κB. ENO1 promotes PASMC proliferation and metabolic switching via the AMPKα1-Akt signaling axis [26]. AK4 is induced by HIF-1α under hypoxia and participates in a positive feedback loop involving AK4, Akt, and HIF-1α [27]. PKM2 is activated by PARP1 and then translocates to the nucleus, where it cooperates with NF-κB to promote inflammation and glycolysis [28]. These molecules not only provide new insights into the pathogenesis of PH but also represent potential therapeutic targets. Pharmacological interventions targeting these molecules, including ENOblock, PARP1 inhibitors (olaparib), and PKM2 activators (TEPP-46), have been shown to alleviate pulmonary vascular remodeling and right vascular dysfunction in animal models [26,27,28]. Because PASMC proliferation shares several characteristics with cancer cell proliferation, and BEL has been shown to inhibit the proliferation of A549 cells, we hypothesize that BEL may also affect PASMC proliferation.

Despite evidence of the therapeutic effects of BEL on tumors, no reports are currently available on its impact on the prevention and treatment of PH, warranting further investigation. Therefore, this work aimed to provide a preliminary experimental basis for the treatment of pulmonary hypertension with BEL and to further investigate its medicinal value. Thus, this study evaluated the effect of BEL on abnormal pulmonary arterial contraction and pulmonary arterial remodeling, and investigated the potential mechanisms involved.

## 2. Materials and Methods

### 2.1. Reagents

BEL was provided by the Northwest Institute of Plateau Biology, China Academy of Sciences (Xining, China, HPLC > 98%). Noradrenaline Bitaetrete (NE, Cat# V54662), NBI31772 (Insulin-like growth factor-I binding protein IGFBP inhibito, Cat# S55971), and LY294002/PI3K inhibitor (Cat# S43088-25 mg) were purchased from OriLeaf (Shanghai, China). KH_2_PO_4_, NaCl, MgSO_4_·7H_2_O, glucose, and NaHCO_3_ were purchased from Tianjin Fuyu Fine Chemical Co., Ltd. (Tianjin, China). Acetylcholine chloride (ACh), EGTA, and cobalt chloride (CoCl_2_) were purchased from Merck Sigma (St. Louis, MO, USA). CaCl_2_ was purchased from McLean Biochemical Technology Co., Ltd. (Shanghai, China). Fetal bovine serum was purchased from Gibco (Thermo Fisher Scientific, Christchurch, New Zealand, Cat# 10091-148). DMEM high-sugar (Cat# PM150210), trypsin 0.25% (Cat# PB180225) and streptomycin (Cat# P1400) were purchased from Punose (Wuhan, China). CCK-8 detection kit (Cat# CK-A361) and cell cycle assay kit (Cat# CK-A351) were purchased from Elabsciene Company (Wuhan, China). Rabbit anti-α-SMA (Cat# 67735-1), rabbit anti-SM-22α (Cat# 83922-2-RR), rabbit anti-vimentin (Cat# 80232-1-RR), rabbit anti-OPN (Cat# 80912-4-RR), rabbit anti-PCNA (Cat# 10205-2-AP), rabbit anti-Aldh1a1 (Cat# 15910-1-AP), rabbit anti-MGP (Cat# 10734-1-AP), rabbit anti-Igfbp5 (Cat# 55205-1-AP), mouse anti-Akt (Cat# 60203-2-IG), rabbit anti-p-Akt (Cat# 80455-1-RR), mouse anti-mTOR (Cat# 66888-1-IG), mouse anti-p-mTOR (Cat# 67778-1-IG), mouse anti-PI3K (Cat# 60225-1), β-actin antibody (Cat# 66009-1-Ig), α-Tubulin antibody (Cat# 66031-1-Ig), GAPDH antibody (Cat# 60004-1-Ig), goat anti-mouse secondary antibody (Cat# SA00001-1), and goat anti-rabbit secondary antibody (Cat# SA00001-2) were purchased from Proteintech (Wuhan, China). Rabbit anti-Col4a6 was purchased from Abclonal (Wuhan, China, Cat# A22673). Rabbit anti-P-PI3K was purchased from Affinity (Beijing, China, Cat# AF3242). RNAsimple Total RNA Extraction Kit (Cat# DP419), FastKing One-step Genomic cDNA First Chain Synthesis Premix Reagent (Cat# KR118-02), and FastReal Rapid Fluorescence Quantitative PCR Premix Reagent (Cat# FP217-02) were purchased from Tiangen Biochemical Technology Co., Ltd. (Beijing, China). KC7F2 (Cat# HY-18777) and Fluo-4-AM (Cat# HY-101896) were purchased from MedChemExpress (Shanghai, China). BCA Protein Assay Kit was purchased from Boster Bioengineering Co., Ltd. (Wuhan, China, Cat# AR0051). Urethane was purchased from Shanghai Macklin Biochemical (Shanghai, China, Cat# U820333-100g). BEL was dissolved in DMSO to prepare a 200 mM stock solution, which was then aliquoted and stored at −20 °C in sealed, light-protected vials.

### 2.2. Preparation of Pulmonary Artery Ring from Rats

SPF Sprague-Dawley (SD) male rats (weighing 180 ± 20 g) were purchased from Laboratory Animal Center, Health Science Center, Xi’an Jiaotong University, with the animal production license number SCXK (Shaanxi) 2023-002. The rats were kept at the Qinghai University Plateau Medicine Research Center, and all the animal experimental procedures were performed in accordance with the regulations of the People’s Republic of China on the management of experimental animals. The rats were sacrificed by cervical dislocation, the chest cavity was quickly opened from the diaphragm, the heart and lungs were collected, and the blood around the heart and lungs was gently removed. The lungs were quickly immersed in clear Krebs–Henseleit (K-H) liquid precooled at 4 °C, which was composed of (mmol/L): 118 NaCl, 4.7 KCl, 2.5 CaCl_2_, 1.2 MgSO_4_·7H_2_O, 1.2 KH_2_PO_4_, 25 NaHCO_3,_ and 11.1 glucose (pH = 7.4) [29]. The adipose tissue and connective tissue around the blood vessel were removed to avoid endothelial damage, yielding vascular rings with intact endothelium (Endo+). Endothelium was removed in some rings by gently rubbing the intimal surface with a fine steel wire, resulting in endothelium-denuded (Endo−) vascular rings. The blood vessel was collected, and the vascular ring was cut with a length of approximately 2–4 mm for subsequent use. 5 mL of K-H solution was introduced to the bath, and a 95% O_2_ + 5% CO_2_ mixture was continuously added; the bath was heated to 37 °C. 1 μmol/L NE was added after 60 min to contract the blood vessels, and 10 μmol/L ACh was added after reaching the maximum value to relax the blood vessels. The endothelium was considered intact if the maximum relaxation rate of the blood vessels reached 75%. The endothelium removal was considered successful if the maximum relaxation rate of blood vessels was <15%. The instrument for measuring vascular tension was an isolated microvessel myograph system (BIOPAC, Goleta, CA, USA, MP160).

### 2.3. Pulmonary Artery Function Assessment

The prepared pulmonary artery ring with Endo+ and Endo− was subjected to contraction of the pulmonary vascular ring until reaching the maximum using 1 μmol/L NE and 60 mmol/L KCl, and then different concentrations of BEL (3–96 μmol/L) were added. Thus, the concentration-response curve of BEL was established. The effect of BEL on extracellular calcium influx and intracellular calcium release in PASMCs was evaluated as follows: The pulmonary artery ring was placed in a bath containing 5 mL of K-H solution for 10 min to achieve equilibrium. A certain initial tension was applied (2–5 mN), and after 10–15 min of equilibrium, 1 μmol/L NE was added to the bath. When the contraction rate exceeded 100%, indicating good vascular responsiveness, the experiment could be conducted. The vessel was washed with 0.2 mmol/L EGTA calcium-free K-H solution and incubated for 15 min. This procedure was repeated three times to chelate extracellular Ca^2+^. Then, the vascular ring was incubated with calcium-free K-H solution without EGTA for 30 min. Then, the vascular ring was pre-incubated with calcium-free K-H solution without EGTA containing BEL (90 μmol/L) for 20 min. Then, the vascular ring was exposed to 1 μmol/L NE to assess the contraction amplitude of the blood vessels. Once the contraction reached the plateau, 2.5 mmol/L CaCl_2_ was added to the bath to restore calcium. The changes in the maximum contraction amplitude of the pulmonary artery rings induced by NE and CaCl_2_ were compared between each group. The instrument for measuring vascular tension was an isolated microvessel myograph system (BIOPAC, Goleta, CA, USA, MP160).

### 2.4. Immunocytochemistry

Cells were seeded into 6-well plates (with coverslips placed at the bottom of the wells). When the cell density reached 80–90%, cell staining was performed. The cells were fixed in 4% paraformaldehyde for 20 min, washed with PBS, and then incubated in 3% Triton X-100 for 20 min to permeabilize the cells. The endogenous enzyme was inactivated at room temperature for 10 min by adding 3% H_2_O_2_. Normal goat serum was added, and the cells were incubated at 37 °C for 30 min. Approximately 1 mL of anti-α-SMA (1:200), anti-Aldh1a1 (1:200), rabbit anti-Mgp (1:200), and anti-Igfbp5 (1:200) were added, and the cells were incubated overnight at 4 °C. Goat anti-Mouse/Rabbit secondary antibody was added, and the cells were incubated at 37 °C for 30 min. A DAB working solution was prepared according to the instructions of the two-step immunohistochemical kit, and positive brown cells were counted. Images were acquired by the Inverted digital biological microscope (Leica Microsystems (Shanghai) Co., Ltd., Germany, MATEO TL RUO, Shanghai, China), and 10–15 fields under ×10 magnification were acquired randomly from each cell sample (*n* = 3). The percentage of positive cell area (Area%) in the captured images was measured using the Image-Pro Plus 6.0 image analysis system (Media Cybernetics, Rockville, Maryland, USA).

### 2.5. Cell Proliferation Analysis

PASMCs were digested with 0.25% trypsin, then resuspended and seeded into 96-well plates at a density of 5 × 10^3^ cells per well, with 6 parallel wells per group. The concentrations of CoCl_2_ used for 24 h were 25, 50, 100, 200, 400, and 600 μmol/L. The concentrations of CoCl_2_ used at 48 h and 72 h were 5, 10, 20, 40, 80, 100, 160, and 200 μmol/L. The final concentration of BEL used under each condition was 10, 20, 40, 80, 120, 140, and 160 μmol/L. The medium in the 96-well plate was removed, and 100 μL of diluted CCK-8 working solution was added to each well. The plate was incubated at 37 °C for 2 h in the dark. The absorbance (OD value) was measured at 450 nm using an Multimode plate reader (POLARstar Omega, BMG LABTECH, Ortenberg, Germany). The cell viability of each group was calculated according to the following formula: cell survival rate (%) = (absorbance of the experimental group − absorbance of the blank group)/(absorbance of the control group − absorbance of the blank group) × 100%. Cell inhibition rate (%) = [1 − (absorbance of the experimental group − absorbance of the blank group)/(absorbance of the control group − absorbance of the blank group) × 100%]. The “blank group” refers to wells containing working solution only (without cells), which are used to correct for background absorbance.

### 2.6. Scratch Assay

PASMCs were seeded into 6-well plates at a concentration of 5 × 10^5^ cells per well. Once the cells adhered to the bottom of the well, several scratches were made at the center of each well using a 20 μL pipette tip. The cells were further treated with 0- (control), 20, 50, and 80 μmol/L BEL and 10 μmol/L CoCl_2_. Wound healing was observed after 72 h, and images were captured under a Inverted digital biological microscope (Leica Microsystems (Shanghai) Co., Ltd., Germany, MATEO TL RUO). The migration distance and healing area were analyzed using Image-Pro Plus 6.0 image analysis system (Media Cybernetics, USA) according to the following formula: cell mobility (%) = [scratch area (0 h) − scratch area (48 h)].

### 2.7. RT-qPCR

The total RNA of cells in each group was extracted according to the instructions of the RNAsimple Total RNA Extraction kit (Cat# DP419). The concentration and purity of RNA were determined using the QuickDrop Ultra Micro spectrophotometer (Molecular Devices, San Jose, CA, USA, Cat# E113783). Reverse transcription of RNA into cDNA was performed according to the instructions of the FastKing One-step Genomic cDNA First Chain Synthesis Premix Reagent (Cat# KR118-02). Table 1 lists the primers used to perform RT-qPCR. The reaction system was prepared according to the FastReal Rapid Fluorescence Quantitative PCR Premix Reagent (Cat# FP217-02), and the PCR reaction was carried out using an Applied BiosystemsTM Quantum Studio 3 (Thermo Fisher Scientific, MA, Waltham, USA) fluorescent quantitative PCR instrument. The protocol used was as follows: 95 °C, 2 min; 95 °C, 5 s, 60 °C, 15 s for a total of 40 cycles. β-actin was used as the reference gene, and the relative expression of each gene was calculated by the 2^−ΔΔCT^ method.

### 2.8. Western Blot

Once the cell treatment for each group was completed, the cells were lysed using RIPA Lysis Buffer (Proteintech, Wuhan, China, Cat# PR20035), and protein concentration was determined by the BCA protein quantitative kit. Protein separation was performed using SDS-polyacrylamide gel electrophoresis, and proteins were transferred to a PVDF membrane. The membrane was blocked with 5% (*w*/*v*) skimmed milk powder in TBST (Tris-buffered saline containing 0.1% Tween-20) for 2 h at room temperature and then incubated with the following primary antibodies at 4 °C overnight: rabbit anti-α-SMA (1:1000), rabbit anti-SM-22α (1:2000), rabbit anti-vimentin (1:1000), rabbit anti-OPN (1:1000), PCNA antibody (1:1000), rabbit anti-Igfbp5 (1:500), mouse anti-Akt (1:5000), rabbit anti-p-Akt (1:500), mouse anti-mTOR (1:5000), mouse anti-p-mTOR (1:2000), and rabbit anti-Col4a6 (1:500). Goat anti-mouse IgG (H+L) labeled with horseradish peroxidase and goat anti-rabbit IgG (H+L) labeled with horseradish peroxidase were used as secondary antibodies (1:5000). The optical density of the protein bands was captured using a Chemoluminescence imaging system, quantified with the Image-Pro Plus 6.0 image analysis system (Media Cybernetics, Rockville, Maryland, USA), and normalized according to the reference protein. β-actin (1:5000), α-Tubulin (1:5000), and GAPDH (1:5000) were used as loading controls.

### 2.9. Transcriptomic-Quantitative Proteomic Combined Analysis

Combined transcriptomic-quantitative proteomic analysis and differential expression analysis were performed on the normoxia group, CoCl_2_ group, and CoCl_2_+ BEL (50 μmol/L) group (*p* < 0.05, FC > 2.0 or FC < 0.5) to screen for differentially expressed genes (DEGs) and differentially expressed proteins (DEPs). KEGG and GO analyses were conducted on DEGs and DEPs, and the potential targets and signaling pathways of BEL in PH were identified. The volcano maps and enrichment results analyzed by KEGG and GO were generated using the R software package ggplot2 (version 3.4.3), while the heat maps and density maps were generated using matplotlib (version 3.8.0).

### 2.10. Molecular Docking Studies

The structures of Aldh1A1 (PDB ID: 4wj9) and Igfbp5 (PDB ID: 7ufg) were retrieved from the Protein Data Bank (PDB). Water molecules and co-crystallizing ligands were removed using PyMOL 2.6.0 [30]. The processed receptor structures were then prepared using AutoDockTools 1.5.7 by adding hydrogen atoms and assigning charges [30]. The 3D structure of BEL was downloaded from the PubChem database and converted to MOL2 format using OpenBabel 2.3.2. The ligand structure was then processed with AutoDockTools 1.5.7 by adding hydrogen atoms, defining rotatable bonds, and assigning charges [30]. Potential binding pockets were predicted using P2Rank 2.5.1. Molecular docking was performed using AutoDock Vina 1.2.7, with the number of docking modes set to 50 [30]. After docking, ligand-receptor complexes were visualized using PyMOL 2.6.0 for 3D analysis, while LigPlot+ 2.2.9 was used for 2D interaction analysis [30]. The detailed docking procedures were as follows. To predict the binding mode of BEL with the target proteins, molecular docking was performed using AutoDock Vina. The receptor protein structure files were 4wj9_end.pdbqt and 7ufg_end.pdbqt, and the ligand structure file was MOL007969.pdbqt. For Aldh1A1, the docking grid center was set at x = 180.943, y = 183.597, and z = 191.767, with box dimensions of 126.0 Å × 126.0 Å × 126.0 Å to ensure complete coverage of the predicted binding region. For Igfbp5, the docking grid center was set at x = 44.281, y = −14.777, and z = 19.727, with box dimensions of 88.0 Å × 92.0 Å × 72.0 Å, to ensure adequate coverage of the predicted binding pocket.

### 2.11. Cellular Thermal Shift Assay

To further identify the target protein of BEL, we performed a Cellular Thermal Shift Assay (CETSA), which is commonly used to evaluate drug-target interactions within cells [31]. First, BEL or DMSO (vehicle control group) was added to the cells and incubated at 37 °C for 12 h. The cells were then treated with trypsin for digestion and centrifuged at 1000 rpm for 5 min at 37 °C to obtain the cell pellet. For each group (BEL or DMSO), 1 × 10^6^ cells were resuspended in RIPA lysis buffer. The cell suspension was divided equally into 8 aliquots and heated at temperatures ranging from 37 °C to 65 °C. After heating, the samples were cooled to room temperature and further lysed at 4 °C for 15 min. The lysates were subsequently centrifuged at 12,000 rpm for 20 min at 4 °C, and the supernatants were collected. Sample loading buffer was then added, and the samples were denatured at 95 °C for 10 min. Protein expression was analyzed by Western blot. Finally, semi-quantitative analysis of the target protein was conducted using the Image-Pro Plus 6.0 image analysis system (Media Cybernetics, USA).

### 2.12. Cell Cycle Analysis

PASMCs were detached using 0.25% trypsin, centrifuged at 1000 rpm for 5 min, resuspended, counted, and incubated at 37 °C for 72 h. Cell cycle analysis was carried out according to the manufacturer’s instructions for the cell cycle analysis kit. After 72 h of incubation, the cells were resuspended at a concentration of 1 × 10^6^ cells/mL. The cells were centrifuged, and the supernatant was discarded. The cell pellet was washed with PBS and re-centrifuged, and the supernatant was discarded. Hundred microliters of RNaseA solution and 400 uL of PI staining solution were added to each cell pellet. The samples were vortexed and subjected to flow cytometry analysis. The distribution of cells in different cell phases was analyzed using ModFit LT 5 software.

### 2.13. EdU Cell Proliferation Assay

Following the EdU incubation time used for similar cell types in the references, the EdU incubation concentration was set at 50 μmol/L, and the incubation time was 3 h [32]. After incubation, the medium was aspirated, and 1 mL of PBS containing 4% paraformaldehyde was added to each well for fixation for 15 min. After fixation, the cells were washed three times with 1 mL of PBS containing 3% BSA per well, 5 min each time. Subsequently, 1 mL of PBS containing 0.3% Triton X-100 was added to each well for cell permeabilization for 20 min. The permeabilization solution was then aspirated, and the cells were washed three times with 1 mL of PBS containing 3% BSA per well, 5 min each time. Next, 500 μL of Click reaction solution prepared according to the specified formula was added to each well to ensure uniform coverage of all cells. The cells were then incubated for 30 min at room temperature in the dark for fluorescence labeling. After incubation, the reaction solution was discarded, and the cells were washed three times with 1 mL of PBS containing 3% BSA per well, 5 min each time, to completely remove residual Click reaction solution. For DNA staining, 500 μL of DAPI working solution was added to each well, and the cells were incubated for 5–10 min at room temperature in the dark. The cells were then washed three times with 1 mL of PBS containing 3% BSA per well, 5 min each time. The results were observed under a fluorescence microscope (Nikon Corporation, Tokyo Metropolis, Japan, H600L) using appropriate filter sets, and 10–15 fields of view at ×10 magnification were randomly captured from each cell sample (*n* = 3).

### 2.14. Measurement of Intracellular Ca^2+^ Concentration by Flow Cytometry

Cells were seeded into 6-well plates. When the cells reached 80–90% confluence, they were incubated with BEL at final concentrations of 20, 50, and 80 μmol/L for 12 h. After washing three times with PBS, the cells were incubated with NE (1 μmol/L) for 15 min. The cells were then washed three times with PBS and digested with trypsin. The cells were counted, and a total of 1 × 10^6^ cells were taken from each sample. The cells were collected by centrifugation (1200 rpm, 5 min, 4 °C) and washed once with PBS. After that, they were resuspended in PBS and added with Fluo-4-AM to a final concentration of 1 μmol/L. The cells were incubated at 37 °C for 30 min, washed three times with PBS (by centrifugation at 1200 rpm for 3 min at 4 °C), and then resuspended in 1 mL of PBS. Fluorescence intensity was analyzed using a flow cytometer (Cenlang Biotech, FongCyte, Beijing, China; Ex/Em = 485/526 nm).

### 2.15. Measurement of Intracellular Ca^2+^ Concentration by Immunofluorescence

Cells were seeded into 6-well plates. When the cells reached 80–90% confluence, they were incubated with BEL at final concentrations of 20, 50, and 80 μmol/L for 12 h. After washing three times with PBS, the cells were subsequently incubated with NE (1 μmol/L) for 15 min. Following incubation, the cells were washed three times with PBS and then incubated with Fluo-4-AM (final concentration = 1 μmol/L) at 37 °C for 30 min. After washing three times with PBS, the cells were observed and photographed under a fluorescence microscope (Nikon Corporation, Japan, H600L). 6–9 fields of view (10× magnification) were captured. The average fluorescence intensity was quantified using Image-Pro Plus 6.0 software and used as an indicator of intracellular calcium concentration.

### 2.16. Statistical Analysis

GraphPad Prism 9.0 was used to plot the graphs, and SPSS 27.0.1 was used for data analysis. Student’s *t*-test was used to compare two groups with normal distribution, and one-way analysis of variance was used to compare multiple groups. The experimental results were expressed as mean ± standard deviation ($\bar{x}$ ± SD). A *p* < 0.05 was considered statistically significant.

## 3. Results

### 3.1. BEL Dilated the Isolated Precontracted Pulmonary Artery

**Figure 1:**
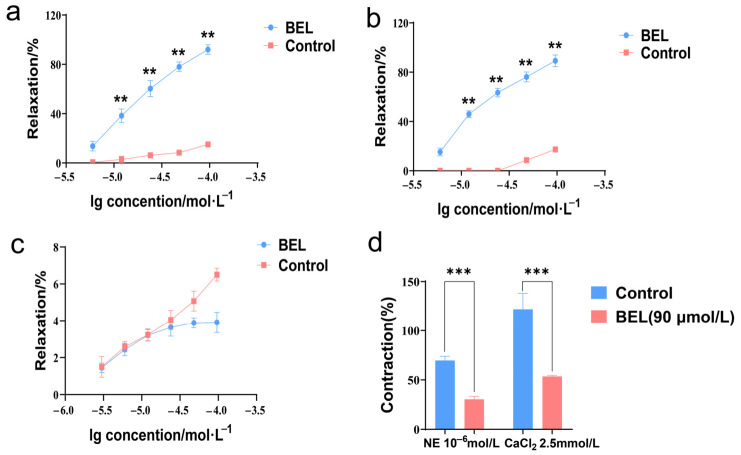
(**a**,**b**) Effect of BEL (6–96 μmol/L) on NE-precontracted pulmonary artery rings of Endo+ and Endo− (*n* = 6). (**c**) Effect of different concentrations of BEL on KCl-precontracted pulmonary artery rings (Endo+) (*n* = 6). (**d**) Effect of high BEL (90 μmol/L) concentration on internal calcium release and external calcium inflow (*n* = 3). The results are expressed as mean ± SD (** *p* < 0.01 and *** *p* < 0.001).

The maximal relaxation rates (Emax) of BEL (6–96 μmol/L) on NE (1 μmol/L)-precontracted pulmonary artery rings were 92.08% ± 3.84% in the Endo+ pulmonary artery rings and 89.24% ± 4.73% in Endo− pulmonary artery rings, respectively (Table 2). The half maximal effective concentration (EC_50_) for the effect of BEL in the dilation of pulmonary blood vessels was 16.91 ± 3.18 μmol/L in the Endo+ pulmonary artery ring and 17.40 ± 2.12 μmol/L in the Endo− pulmonary artery ring, respectively (Table 2). BEL relaxed the intact Endo+ and Endo− pulmonary artery ring in a concentration-dependent manner, and the difference was statistically significant compared with the model group (*p* < 0.01) (Figure 1a,b). No statistically significant difference was observed in the maximum relaxation rate of BEL (6–96 μmol/L) on NE (1 μmol/L) precontracted Endo+ and Endo− pulmonary artery rings, and no statistically significant difference was observed in the EC_50_ between Endo+ and Endo− pulmonary artery rings (Table 2). The cumulative concentration of BEL (3–96 μmol/L) did not exert any evident effect on isolated pulmonary artery rings with Endo+ precontracted by KCl (60 mmol/L) compared with the model group (Figure 1c).

### 3.2. BEL Induced Vasodilatation by Inhibiting Intracellular Calcium Release and Extracellular Calcium Flow in PASMCs

Pre-incubation with BEL (90 μmol/L, 20 min) resulted in the inhibition of the vasoconstriction caused by NE, with the vasoconstriction rate decreasing from 69.70% ± 0.04% to 30.45% ± 0.03% (*p* < 0.001). BEL (90 μmol/L) significantly inhibited the vasoconstriction caused by CaCl_2_, with the vasoconstriction rate decreasing from 121.93% ± 0.16% to 53.59% ± 0.01% (*p* < 0.001). Therefore, BEL exerted its vasodilatory effect by inhibiting the release of intracellular calcium and the influx of extracellular calcium (Figure 1d).

### 3.3. Effect of BEL on Intracellular Calcium Concentration in Rat PASMCs

**Figure 2:**
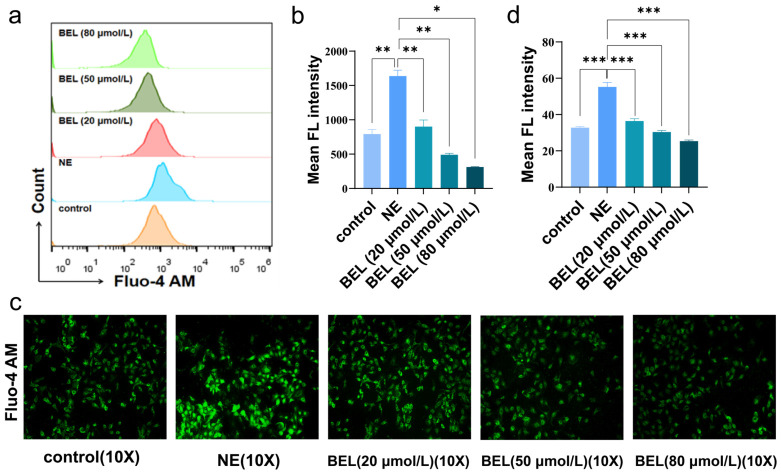
(**a**,**b**): Intracellular Ca^2+^ concentration in rat PASMCs measured by flow cytometry. (**c**,**d**) Intracellular Ca^2+^ concentration in rat PASMCs measured by immunofluorescence. The results are expressed as mean ± SD (*n* = 3). (* *p* < 0.05, ** *p* < 0.01 and *** *p* < 0.001).

The results showed that NE (1 μmol/L) significantly increased intracellular calcium concentration in rat PASMCs (*p* < 0.05). In contrast, BEL decreased the mean fluorescence intensity (*p* < 0.05, Figure 2). The above results indicate that BEL attenuated the NE-induced increase in intracellular Ca^2+^ concentration.

### 3.4. The Effect of BEL in Proliferation of PASMCs Induced by CoCl_2_

Primary rat PASMCs were successfully cultured using the standard tissue explant adherent method. The cells exhibited the typical “hill-and-valley” morphology and showed high expression of smooth muscle markers (Figure S1) [33]. Based on preliminary screening, we identified that treatment with 10 μmol/L CoCl_2_ for 72 h induced the strongest proliferation effect on PASMCs (Figure S2).

**Figure 3:**
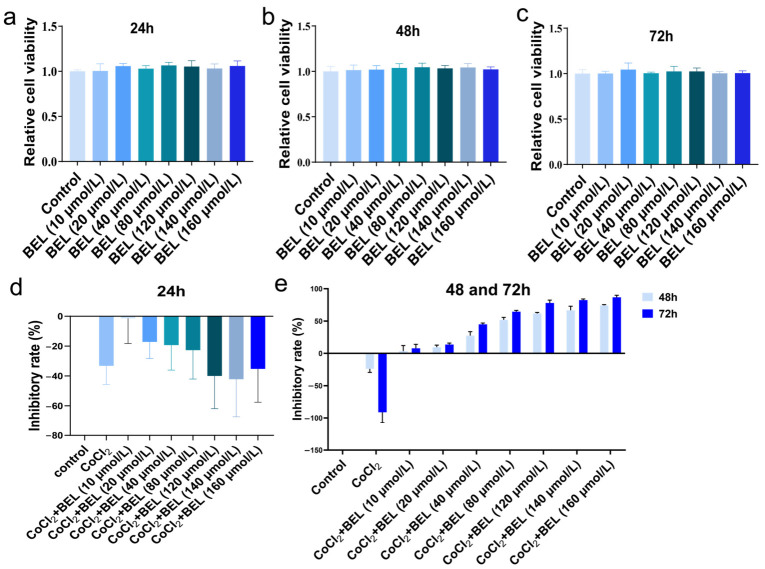
(**a**–**c**): Effect of BEL (10–160 μmol/L) on the proliferation of PASMCs at 24 (**a**), 48 (**b**), and 72 h (**c**). (**d**) Effect of BEL (10–160 μmol/L) on the proliferation of PASMCs induced by CoCl_2_ 24 h. (**e**) Effect of BEL (10–160 μmol/L) on the proliferation of PASMCs induced by CoCl_2_ 48 and 72 h. The results are expressed as mean ± SD (*n* = 3).

BEL (10–160 μmol/L) did not exert any significant effect on the viability of PASMCs after exposure for 24, 48, and 72 h compared with the control group (*p* > 0.05), with no cytotoxic effects on PASMCs at the tested concentrations (Figure 3a–c). BEL did not inhibit the growth of PASMCs induced by CoCl_2_ at 24 h compared with the control group (Figure 3d). However, BEL inhibited the proliferation of PASMCs induced by CoCl_2_ at 48 and 72 h in concentration-dependent manner (Figure 3e). The IC_50_ of PASMCs treated with CoCl_2_ was 71.65 μmol/L at 48 h and 48.77 μmol/L at 72 h. The inhibitory effect of BEL on PASMCs treated with CoCl_2_ was increased with increasing incubation time, and the effect was more evident at 72 h. Thus, 72 h was selected as the subsequent experimental time, and 20, 50, and 80 μmol/L were selected as the subsequent experimental concentrations.

### 3.5. BEL Treatment Arrested the Cell Cycle at the G2/M Phase

**Figure 4:**
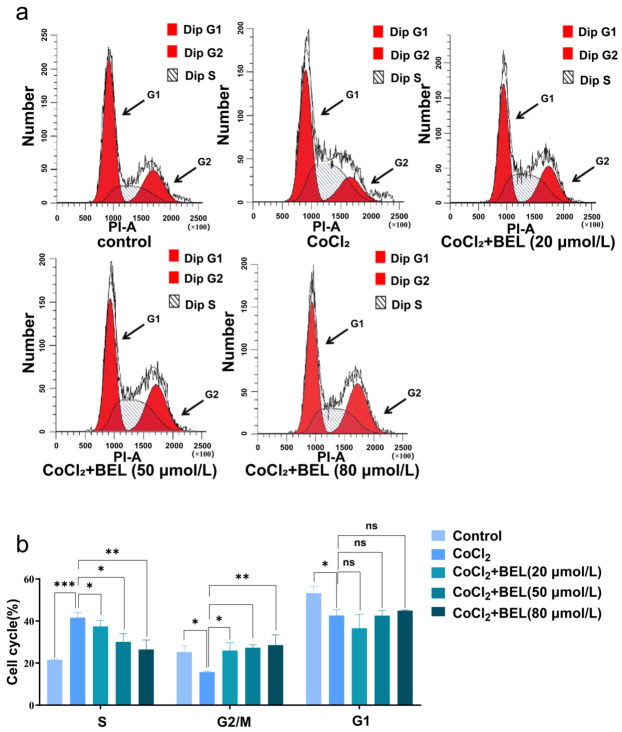
BEL treatment arrested the cell cycle at the G2/M phase. (**a**) PASMC circulation in each group was detected. The black curve in **a** indicates the overall model fit. (**b**) Quantification of the S, G2/M and G1 phases of each group of cells. The results are expressed as mean ± SD (*n* = 3). (ns, not significant (*p* > 0.05), * *p* < 0.05, ** *p* < 0.01 and *** *p* < 0.001).

The number of PASMCs in the S phase significantly increased after CoCl_2_ treatment for 72 h compared with the control group (*p* < 0.01). The number of cells in the S phase was lower than that in the CoCl_2_ model group (*p* < 0.01), and the number of cells in the G2/M-phase was higher than that in the CoCl_2_ model group (*p* < 0.01) after 72 h of treatment with BEL (20, 50, and 80 μmol/L). These results suggested that BEL treatment arrested the cell cycle at the G2/M phase (Figure 4).

### 3.6. BEL Inhibited PASMCs Migration Induced by CoCl_2_

**Figure 5:**
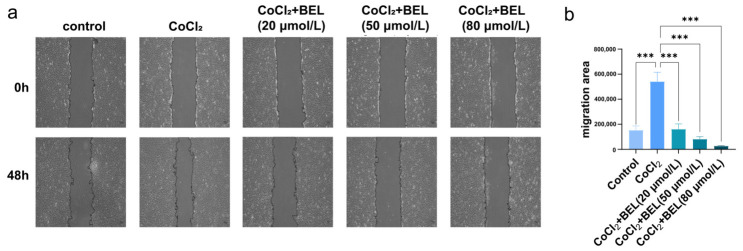
Effect of BEL on PASMC migration. (**a**) Effect of BEL (20, 50, and 80 μmol/L) on the migration of PASMCs induced by CoCl_2_. (**b**) Quantification of wound closure. The results are expressed as mean ± SD (*n* = 3) (*** *p* < 0.001).

The scratch assay was performed to verify whether BEL affected the migration ability of PASMCs induced by CoCl_2_ in vitro. The migration area of the cells in the CoCl_2_ group significantly increased at 48 h compared with that in the control group (*p* < 0.05). The migration area of PASMCs significantly decreased after 48 h of exposure to BEL (20, 50, and 80 μmol/L) compared with that in the model group (*p* < 0.05). These results revealed that CoCl_2_ increased PASMC migration, whereas BEL reduced the CoCl_2_-induced migration (Figure 5).

### 3.7. BEL Regulated the Expression of Phenotypic Transformation Marker in PASMCs

**Figure 6:**
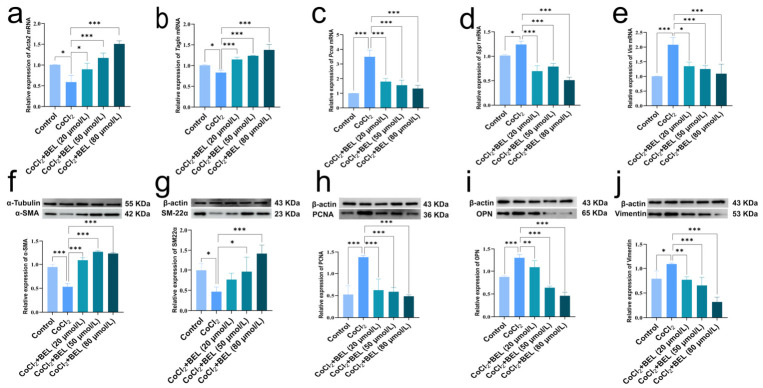
Effect of BEL on the expression of *Acta2* (**a**), *Tagln* (**b**), *Pcna* (**c**), *Spp1* (**d**), and *Vim* (**e**) mRNA in PASMCs treated with CoCl_2_. Effect of BEL on the expression of α-SMA (**f**), SM22α (**g**), PCNA (**h**), OPN (**i**), and vimentin (**j**) proteins in PASMCs induced by CoCl_2_. The results are expressed as mean ± SD (*n* = 3). (* *p* < 0.05, ** *p* < 0.01 and *** *p* < 0.001). Original western blot images see Supplementary Material File S1.

The mRNA expression of the synthetic marker genes *Spp1*, *Pcna*, and *Vim* significantly increased after CoCl_2_ (10 μmol/L) treatment for 72 h compared with the control group (*p* < 0.05 or 0.001), while the mRNA expression of the contractile marker genes *Acta2* and *Tagln* in CoCl_2_ group decreased compared with the control group (*p* < 0.05). The mRNA expression of *Acta2* and *Tagln* significantly increased, and the mRNA expression of *Spp1*, *Pcna*, and *Vim* significantly decreased compared with their expression in the CoCl_2_ group (*p* < 0.05 or 0.001) after BEL(20, 50, and 80 μmol/L) treatment. Therefore, these results indicate that after BEL intervention, the transition of PASMCs from the contractile phenotype to the synthetic phenotype is inhibited (Figure 6a–e).

The expression of the contractile-related proteins α-SMA and SM22α in PASMCs treated with CoCl_2_ (10 μmol/L) significantly decreased compared with the control group (*p* < 0.05). However, the expression of vimentin, PCNA, and OPN significantly increased compared with the control group (*p* < 0.05). The expression of α-SMA and SM22α significantly increased after treatment with BEL(50 and 80 μmol/L) compared with that in the CoCl_2_ group (*p* < 0.05). The expression of α-SMA significantly increased after treatment with BEL (20 μmol/L) compared with that in the CoCl_2_ group (*p* < 0.001). The expression of OPN, vimentin, and PCNA significantly decreased after treatment with BEL (20, 50, and 80 μmol/L) compared with the CoCl_2_ group (*p* < 0.01). The above experimental data indicate that BEL can counteract the dedifferentiation response of PASMCs to CoCl_2_ (Figure 6f–j).

### 3.8. Transcriptome-Quantitative Proteomics-Combined Analysis Identified Potential Targets and Signaling Pathways of BEL in PH

**Figure 7:**
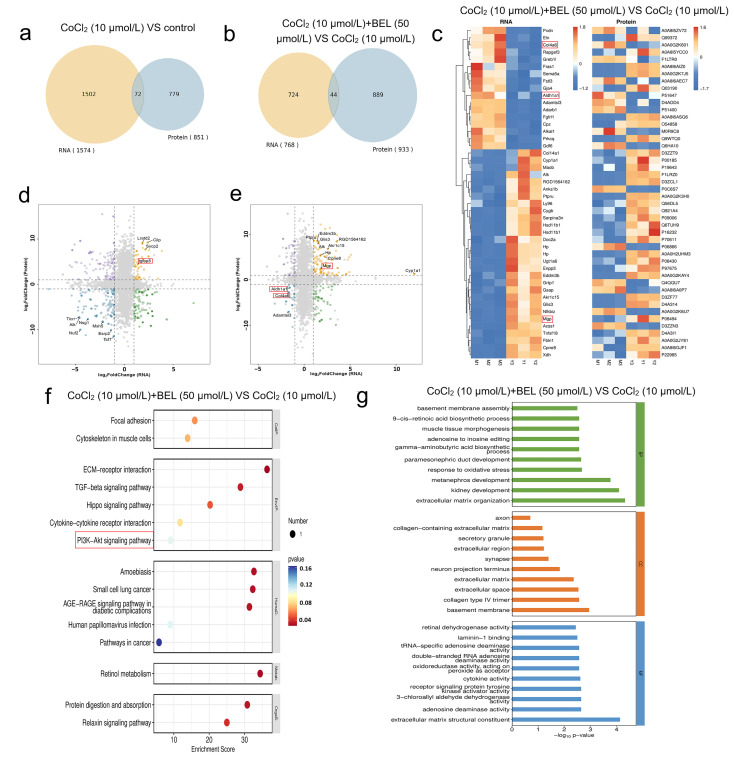
Transcriptome-quantitative proteomics-combined analysis. (**a**) Venn diagram showing the intersection analysis of DEGs and DEPs between the CoCl_2_ group and control groups. (**b**) Venn diagram showing the intersection analysis of DEGs and DEPs between the CoCl_2_ (10 μmol/L) group and the CoCl_2_ (10 μmol/L) + BEL (50 μmol/L) groups. (**c**) Heat map showing the expression profiles of DEGs and DEPs in the CoCl_2_ (10 μmol/L) + BEL (50 μmol/L) group vs. the CoCl_2_ (10 μmol/L) group. (**d**,**e**) The volcano plot displays the annotation information for genes and proteins that show differences in omics expression. (**f**) KEGG analysis (the CoCl_2_ (10 μmol/L) + BEL (50 μmol/L) group vs. the CoCl_2_ (10 μmol/L) group). (**g**) GO analysis (the CoCl_2_ (10 μmol/L) + BEL (50 μmol/L) group vs. the CoCl_2_ (10 μmol/L) group). The red boxes indicate the key genes and proteins /pathways identified through screening in the (**c**–**f**). In the four quadrants of (**d**,**e**), gray dots represent features with no significant differences in either omics dataset; light-colored dots indicate features that are significant in only one of the single-omics analyses; dark-colored dots denote features that show significant differences in both transcriptomic and proteomic analyses.

PASMCs were divided into three groups: control, the CoCl_2_ (10 μmol/L), and the CoCl_2_ (10 μmol/L) + BEL (50 μmol/L). Cells were treated with drugs for 72 h. Transcriptomic and quantitative proteomic analyses were performed on these groups, followed by a combined analysis to identify differentially expressed genes (DEGs) and proteins (DEPs). The intersection of DEGs between transcriptome and quantitative proteomics was assessed, identifying 72 DEGs (Figure 7a) between the CoCl_2_ (10 μmol/L) group and the control groups, and 44 common DEGs between the CoCl_2_ (10 μmol/L) group and the CoCl_2_ (10 μmol/L) + BEL (50 μmol/L) groups (Figure 7b). A multiomics expression profile analysis was performed on the intersection genes (CoCl_2_ (10 μmol/L) + BEL (50 μmol/L) group vs. the CoCl_2_ (10 μmol/L) group, Figure 7c).

DEGs and DEPs were evaluated across all groups, and genes were ranked based on the integrated omics expression differences. Among the significantly altered genes and proteins in the CoCl_2_ (10 μmol/L) group, Igfbp5 was identified as a prominent candidate (Figure 7d). Compared with the CoCl_2_ (10 μmol/L) group, the CoCl_2_ (10 μmol/L) + BEL (50 μmol/L) showed significantly differential expression of Aldh1A1, Mgp, and Col4a6 at both the transcriptomic and proteomic levels (Figure 7e).

KEGG and GO enrichment analyses were performed using DEGs. According to the KEGG results, the CoCl_2_ (10 μmol/L) + BEL (50 μmol/L) group showed significant enrichment in signaling pathways such as PI3K-AKT compared to the CoCl_2_ (10 μmol/L) group (Figure 7f). The GO analysis results indicated significant enrichment in processes such as “response to hypoxia,” “oxidative stress response,” “nitrite reductase (NO-formation) activity,” “calcium-dependent phospholipid binding,” “redox enzyme activity,” and “extracellular matrix structural components” (CoCl_2_ (10 μmol/L) + BEL (50 μmol/L) group vs. the CoCl_2_ (10 μmol/L) group, Figure 7g). The occurrence and development of PH were related to these biological processes.

### 3.9. Validation of Differentially Expressed Genes and Proteins

**Figure 8:**
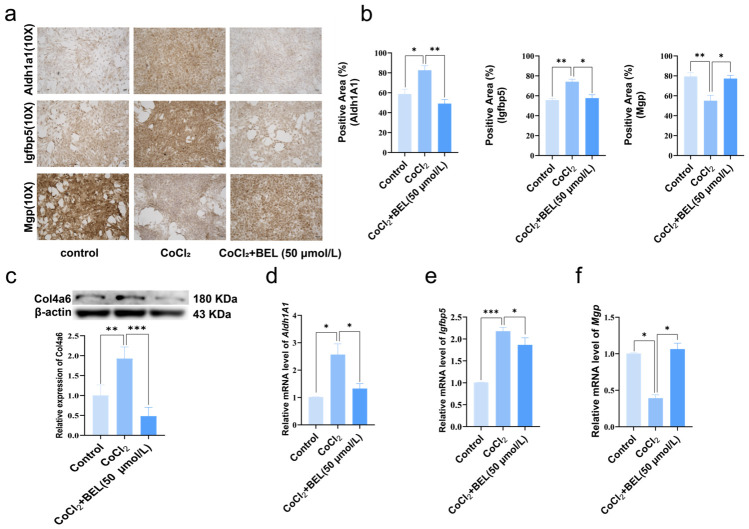
RT-qPCR, immunocytochemistry and Western blot validation of differentially expressed genes and proteins. (**a**,**b**) Representative results of Aldh1A1, Mgp, and Igfbp5 immunocytochemistry staining. (**c**) Representative results of Col4a6 Western blot analysis. (**d**–**f**) Representative results of the relative expression levels of *Aldh1A1*, *Igfbp5,* and *Mgp* mRNA. The results are expressed as mean ± SD (*n* = 3) (* *p* < 0.05, ** *p* < 0.01, and *** *p* < 0.001). Original western blot images see Supplementary Material File S1.

Further verification confirmed the inhibitory effect of BEL on the protein and mRNA expression levels of Aldh1A1, Mgp, Col4a6, and Igfbp5 in CoCl_2_-induced PASMCs. Western blot and immunocytochemistry results showed that, compared with the control group, after treatment with CoCl_2_ (10 μmol/L) for 72 h, the expression levels of Aldh1A1, Col4a6, and Igfbp5 proteins significantly increased, while the expression level of Mgp protein significantly decreased. After intervention with BEL (50 μmol/L), the expression levels of Aldh1A1, Col4a6, and Igfbp5 proteins significantly decreased, and the expression level of Mgp protein significantly increased. RT-qPCR results showed that, after 72 h of BEL treatment, the mRNA expression levels of Aldh1A1 and Igfbp5 were significantly decreased compared to the model group (CoCl_2_ group) (*p* < 0.05). In addition, the elevated mRNA expression level of Mgp induced by CoCl_2_ was also significantly reduced (*p* < 0.05). This trend is consistent with results from combined transcriptomic and quantitative proteomic analyses (Figure 8).

### 3.10. To Validate the Role of Igfbp5 as a Potential Target of BEL

**Figure 9:**
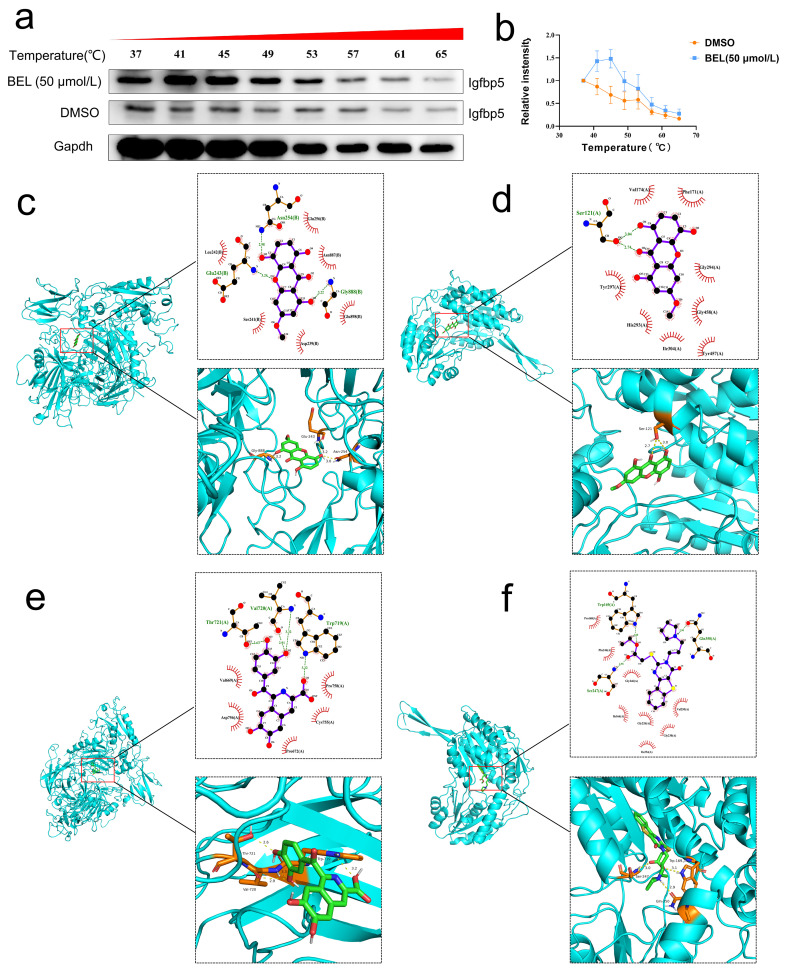
The molecular docking image of the key proteins and BEL. (**a**,**b**) The representative image of Igfbp5’s CETSA, (*n* = 3). Schematic diagrams of molecular docking: (**c**) Igfbp5 with BEL, (**d**) Aldh1a1 with BEL, (**e**) Igfbp5 with NBI31772, and (**f**) Aldh1a1 with CM037. Original western blot images see Supplementary Material File S1.

Next, we used CETSA to examine whether BEL affected the thermal stability of Igfbp5. The results showed that the residual amount of Igfbp5 protein in the BEL (50 μmol/L) group was higher than that in the DMSO group, indicating that BEL (50 μmol/L) enhanced the thermal stability of Igfbp5 in the PASMCs lysate (Figure 9a,b).

In-depth molecular docking was conducted between BEL and the Key differential proteins, revealing intricate details of their molecular interactions. In the docking results, the minimum binding affinities of BEL with Igfbp5 and Aldh1A1 were −7.1 and −8.4 kcal/mol, respectively. The binding energies, a pivotal indicator of ligand-receptor affinities, were considered strong when their values were lower than −5 kcal/mol. Higher absolute values of the binding energies implied more substantial hydrogen bond formations, suggesting a more stable ligand-receptor complex. Through literature review, we identified CM037 as an inhibitor of Aldh1A1 [34,35] and NBI31772 as an inhibitor of Igfbp5 [36]. Molecular docking was then performed between CM037 and Aldh1A1, as well as between NBI31772 and Igfbp5. The results showed that the binding energy of CM037 to Aldh1A1 was −8.0 kcal/mol, and that of NBI31772 to Igfbp5 was −9.8 kcal/mol. The results showed that the absolute value of the binding energy between BEL and Igfbp5 was smaller than that between NBI31772 and Igfbp5, while the absolute value of the binding energy between BEL and Aldh1A1 was similar to that between CM037 and Aldh1A1.

Further observations of the molecular docking results through PyMOL visualization (Figure 9) revealed that the formation of multiple hydrogen bonds was a common feature of the successful docking conformations. BEL formed hydrogen bonds with GLU B898 and GLU B243 of Igfbp5 (Figure 9c). Similarly, BEL interacted with SER A1221 and CYS A302 of Aldh1A1 through hydrogen bonds (Figure 9d). These visual mappings revealed the spatial configuration and interaction sites, showing how BEL formed hydrogen bonds at specific amino acid residues, further stabilizing the drug-target complex, and indicating that this interaction was highly specific. This detailed molecular insight highlights the potential of BEL to regulate the functions of key proteins involved in PH, providing a strong impetus for continued therapeutic exploration and development.

### 3.11. Effects of BEL on the PI3K-AKT-mTOR Signaling Pathways

**Figure 10:**
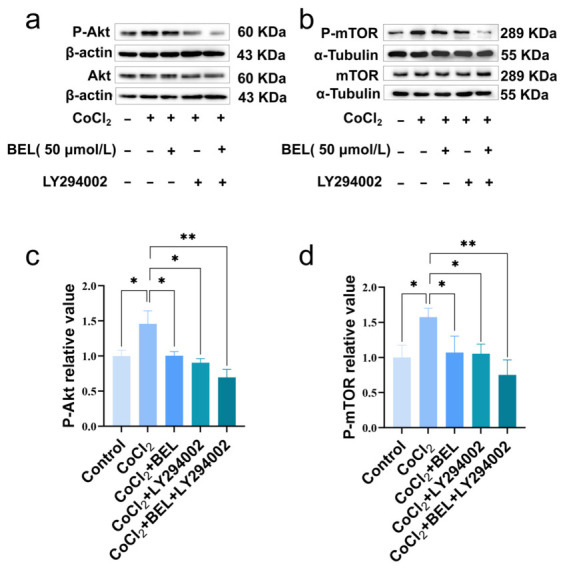
(**a**–**d**) The protein expression of p-Akt and p-mTOR was detected by Western blot in the PASMCs. The results are expressed as mean ± SD (*n* = 3) (* *p* < 0.05 and ** *p* < 0.01). Original western blot images see Supplementary Material File S1.

Literature reports that Igfbp5 is an important member of the IGFBP family and has a high affinity for insulin-like growth factors (IGFs), which are key upstream activating factors of the phosphatidylinositol 3-kinase/protein kinase B/mammalian target of rapamycin (PI3K-AKT-mTOR) signaling pathway [37,38,39]. The PI3K-AKT-mTOR signaling pathway was enriched in the KEGG analysis (Figure 7f). We used the PI3K inhibitor LY294002 [40] to observe the effect of BEL on the PI3K-AKT-mTOR signaling pathway. The results indicated that CoCl_2_ could induce the expression of Igfbp5 protein and mRNA, as well as P-AKT and P-mTOR in PASMCs, while BEL could inhibit the expression of Igfbp5 protein (Figure 8b) and mRNA (Figure 8e), P-AKT, and P-mTOR induced by CoCl_2_. These data suggested that in the hypoxic environment induced by CoCl_2_, Igfbp5 was upregulated, thereby activating the PI3K-AKT-mTOR signaling pathway. However, this activation effect could be counteracted by BEL (Figure 10).

### 3.12. Effects of BEL on the Igfbp5-PI3K-Akt-mTOR Axis

**Figure 11:**
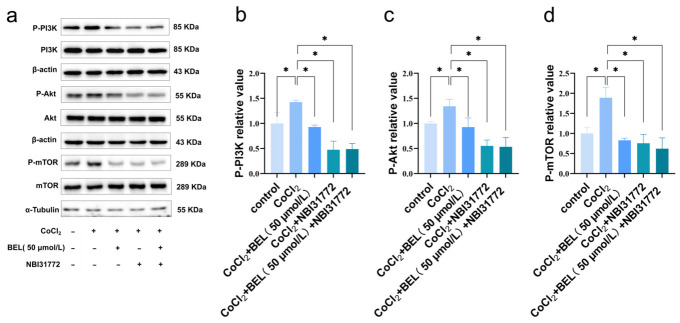

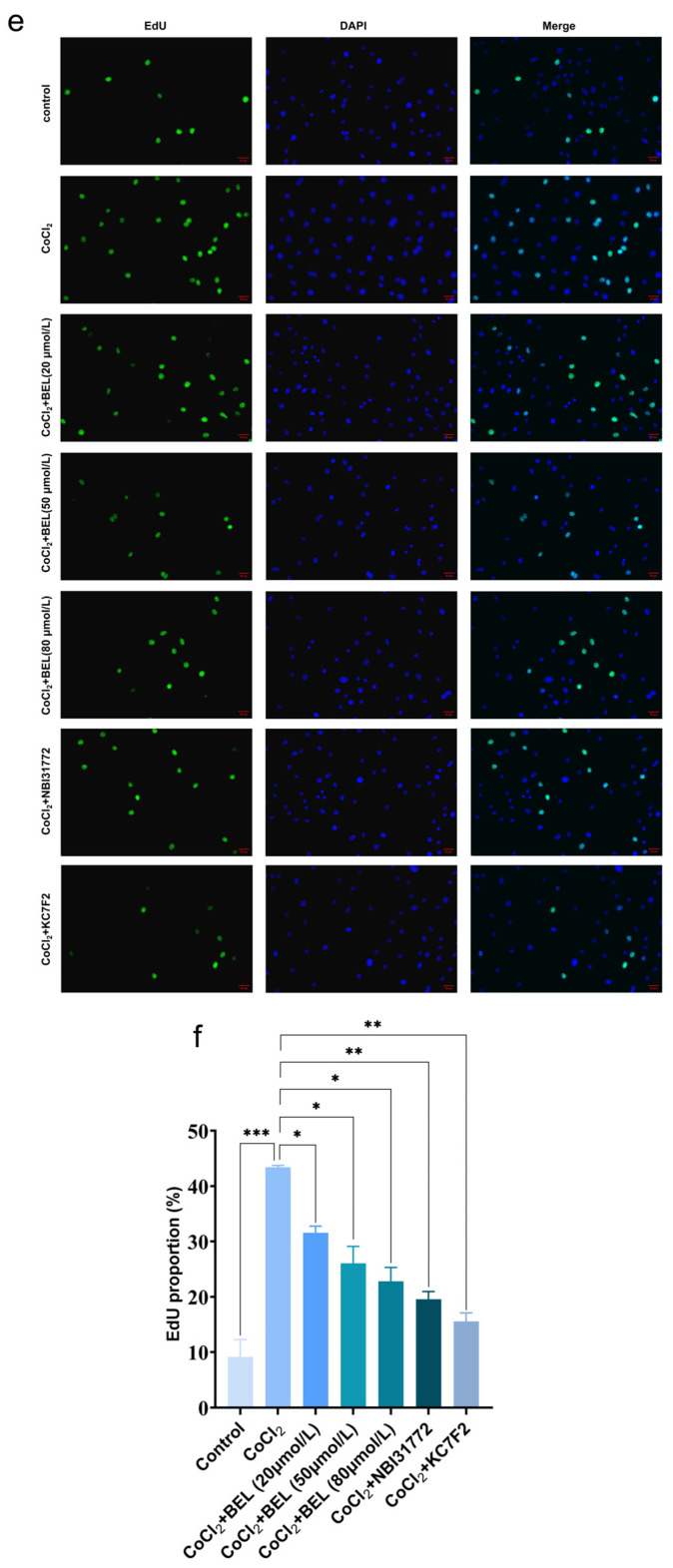
BEL suppresses abnormal PASMC proliferation through the Igfbp5-mediated PI3K-Akt-mTOR signaling axis. (**a**–**d**) Protein expression levels of p-PI3K, p-Akt, and p-mTOR in PASMCs detected by Western blot. BEL suppresses abnormal PASMC proliferation through the Igfbp5-mediated PI3K-Akt-mTOR signaling axis. (**e**,**f**) Effects of BEL on PASMC proliferation through Igfbp5. The red scale bar in the lower right corner of **e** represents 50 μm. Data are expressed as mean ± SD (*n* = 3) (* *p* < 0.05, ** *p* < 0.01 and *** *p* < 0.001). Original western blot images see Supplementary Material File S1.

Western blot analysis (Figure 11a–d) demonstrated that, compared with the control group, the phosphorylation levels of PI3K, Akt, and mTOR proteins (p-PI3K/PI3K, p-Akt/Akt, and p-mTOR/mTOR) were significantly increased in the CoCl_2_ group (*p* < 0.05). These results further confirm that a hypoxic microenvironment can markedly activate the PI3K-Akt-mTOR signaling pathway. Compared with the CoCl_2_ group, BEL (20, 50, 80 μmol/L) treatment significantly reduced the phosphorylation levels of PI3K, Akt, and mTOR. This indicates that BEL can effectively suppress the aberrant activation of t his signaling pathway. In addition, treatment with the Igfbp5 inhibitor NBI31772 also significantly reduced the phosphorylation levels of these signaling molecules, suggesting that inhibition of Igfbp5 may attenuate activation of the PI3K-Akt-mTOR signaling pathway. Based on the above results, it can be inferred that the inhibitory effects of BEL on the PI3K-Akt-mTOR signaling pathway are consistent with those observed following Igfbp5 inhibition. These results support the possibility that BEL inhibits the PI3K-Akt-mTOR signaling pathway by targeting and regulating Igfbp5 activity.

The EdU proliferation assay results (Figure 11e,f) showed that, compared with the control group, the proportion of EdU-positive cells was significantly increased in the CoCl_2_ group (*p* < 0.05), indicating that the hypoxic microenvironment markedly promotes the abnormal proliferation of PASMCs. This observation is consistent with the pathophysiological characteristics of pulmonary vascular remodeling and further validates the hypoxia model. Compared with the CoCl_2_ group, the proportion of EdU-positive cells was significantly reduced following BEL intervention at different concentrations. Moreover, the antiproliferative effect exhibited a concentration-dependent trend. These results suggest that BEL inhibits CoCl_2_-induced PASMCs proliferation in a concentration-dependent manner. Furthermore, treatment with NBI31772 alone (Igfbp5 inhibitor group) or KC7F2 alone (positive control group) significantly reduced the proportion of EdU-positive cells compared to the CoCl_2_ group (*p* < 0.05). The antiproliferative effects observed in these groups were comparable to those observed in the medium- and high-concentration BEL treatment groups.

## 4. Discussion

PH is a heterogeneous clinical disease characterized by an abnormal increase in pulmonary artery pressure. Pulmonary vasculopathy, characterized by pathologic remodeling and vasoconstriction of the pulmonary arteries, results in exercise intolerance, right ventricular failure, and death [41]. Drugs such as prostaglandin analogs and endothelin receptor antagonists mainly exert their therapeutic effects by correcting the imbalance between vasoconstriction and vasodilation, but they have only limited efficacy in arterial remodeling [42]. PASMCs in PH have unique characteristics, such as hyperproliferation and lack of apoptosis, similar to the characteristics of tumors, which mainly lead to occlusive vascular disease with medial hyperplasia and proliferation disorder [43]. However, at present, few drugs effectively inhibit the excessive proliferation of PASMCs or improve the remodeling of pulmonary arteries, which is a key factor in PH severity [42]. Although remarkable progress has been made in the diagnosis and treatment of PH in recent years, patients with PH still have a high mortality rate [5]. Therefore, it is of utmost importance to continue the search for new, effective drugs that address abnormal pulmonary artery constriction and remodeling.

As the main cellular component of pulmonary vessels, PASMCs maintain the normal function and structure of the pulmonary artery. There is a dynamic balance between the proliferation and apoptosis of PASMCs under physiological conditions [44]. However, long-term exposure to hypoxia leads to excessive proliferation and migration of PASMCs, which in turn leads to the further development of PH. Overproliferation of PASMCs is the most prominent feature of pulmonary arterial remodeling [45]. The results showed that seven BEL concentrations (10–60 μmol/L) did not exert any evident effect on PASMCs under normoxia after 24, 48, and 72 h. BEL (10–160 μmol/L) inhibited the proliferation of PASMCs induced by CoCl_2_ for 48 and 72 h in a concentration and time-dependent manner. The above results indicated that within the tested concentration range, BEL did not exhibit cytotoxicity in the normoxic condition. In the hypoxic condition induced by CoCl_2_, BEL could significantly inhibit the proliferation of PASMCs. The IC_50_ of BEL was determined, revealing that its effect was more pronounced at 72 h.

In this study, CoCl_2_ was used as a chemical hypoxia mimetic; however, several limitations should be acknowledged. First, the effects of CoCl_2_ are not entirely specific to the HIF pathway, as cobalt ions can directly induce reactive oxygen species generation and impair mitochondrial function [46,47]. Second, prolonged exposure may trigger non-specific cellular responses associated with metal ion toxicity [48]. Third, the 72 h treatment duration exceeds the typical time frame associated with direct HIF-1α activation. Therefore, the proliferative effect observed following treatment with 10 μmol/L CoCl_2_ at 72 h may reflect a combination of HIF-dependent hypoxic signaling and non-specific cobalt ion-mediated effects. Future studies should incorporate cross-validation under physiological hypoxic conditions using a hypoxic chamber and/or alternative hypoxia mimetics (e.g., DMOG) to distinguish HIF-specific responses from non-specific metal ion effects.

The contraction and dilation of blood vessels are regulated by the smooth muscle cells in the middle layer and the endothelial cells in the inner layer [49]. To explore the specific sites of BEL-mediated regulation of vascular dilation, we conducted experiments to assess the drug’s effect on vascular dilation under both Endo+ and Endo− conditions. The present study demonstrated that the concentration-dependent inhibitory effect of BEL on NE-precontracted pulmonary arterial rings was not statistically different between Endo+ and Endo− preparations, indicating that BEL may mainly exert the vasodilatory effect by relaxing the smooth muscle layer of blood vessels. Therefore, we speculate that BEL may also offer therapeutic advantages in treating endothelial damage associated with diseases such as atherosclerosis, hypertension, and diabetes. Studies have shown that although hypoxic pulmonary vascular contraction can be enhanced by endothelium-derived vasoconstrictors (such as endothelin and thromboxane) and inhibited by endothelium-derived vasodilators (such as nitric oxide and prostacyclin), the core mechanism of hypoxic pulmonary vascular contraction is located in PASMCs [50]. Therefore, we subsequently investigated the effects of BEL on PASMCs. KCl induces Ca^2+^ influx through voltage-dependent Ca^2+^ channels, which subsequently triggers Ca^2+^ release via the ryanodine receptor. In vascular smooth muscle cells, NE simultaneously activates PLC-IP_3_-mediated intracellular calcium release, and is also involved in promoting Ca^2+^ influx through voltage-gated channels [51]. Our study showed that BEL could inhibit NE-induced pulmonary arterial contraction, but did not affect KCl-induced pulmonary arterial contraction. The findings indicate that the inhibitory effect of BEL on pulmonary vascular contraction may not be mediated through voltage-dependent Ca^2+^ channels. Further studies are required to elucidate the underlying mechanism.

The contraction response of pulmonary artery rings to NE can be divided into two stages: the release of Ca^2+^ from the endoplasmic reticulum into the cytoplasm, and the influx of Ca^2+^ from the extracellular fluid and the influx of Ca^2+^ from the extracellular fluid via a secondary mechanism [52]. The intracellular calcium release induced by NE in the absence of calcium K-H solution and the extracellular calcium influx caused by CaCl_2_ in the extracellular K-H solution. The results showed that BEL dilated isolated rat pulmonary arteries by inhibiting extracellular Ca^2+^ influx and intracellular Ca^2+^ release.

The cell cycle is the core regulator of cell proliferation, and cell cycle disorder is the key to abnormal cell proliferation [53]. This study showed that after CoCl_2_ treatment, the proportion of PASMCs in the S phase significantly increased, whereas after BEL treatment, the proportion of cells in the G2/M phase also significantly increased. BEL inhibited hypoxia-induced abnormal proliferation of PASMCs. Hypoxia-induced PASMC migration is also a significant cause of pulmonary arterial remodeling [54,55]. VSMC migration occurs during vascular development, vascular injury, and vascular wall remodeling. When the blood vessels are stimulated by external factors or injured, Vascular Smooth Muscle Cells lose the expression of contractile proteins and acquire the ability to proliferate and migrate from the media to the subendothelial space, resulting in the thickening of the media and intima, and playing an essential role in the progression of various cardiovascular diseases [56]. Based on previous research, our study showed that BEL could inhibit the abnormal proliferation and migration of PASMCs induced by CoCl_2_, thereby improving pulmonary arterial remodeling and alleviating pulmonary hypertension. Further in vivo experiments are needed to observe the effect of BEL on pulmonary arterial remodeling.

Most PASMCs are at rest to maintain contractile function in the physiological state, which is considered their differentiated state. Various growth factors and inflammatory mediators (such as platelet-derived growth factor, TGF-β, and angiotensin-II), as well as mechanical stimulation, epigenetic mechanisms, and changes in the composition and structure of the ECM, are among the decisive factors in their transformation from the contraction type to the synthesis type. In this process, Vascular Smooth Muscle Cells gain the ability to proliferate, migrate, and express specific protein markers [57,58,59]. α-SMA and SM22α are commonly used to indicate the phenotype of contractile smooth muscle cells, and these proteins are primarily used as components and regulatory factors of cell contraction. During the transition to dedifferentiation or synthetic phenotype, the expression of these contraction markers decreases, and the expression of smooth muscle actin even disappears. In contrast, the expression of synthetic phenotype markers (such as OPN) increases during the transition [60]. Increasing evidence indicates that the phenotypic transformation of PASMCs under hypoxic exposure is closely associated with hypoxic pulmonary arterial remodeling [61,62]. The results showed that BEL reversed the phenotypic transformation of PASMCs induced by CoCl_2_ and inhibited the abnormal proliferation of cells caused by the phenotypic transformation in the hypoxic environment.

The key target proteins identified through combined transcriptomic and proteomic analysis include Igfbp5, Col4a6, Aldh1A1, and Mgp. Col4a6 is a structural protein [63,64]. Since structural proteins typically lack obvious active sites or easily accessible small pockets, they are traditionally more difficult to directly target with small-molecule compounds [65]. To identify the BEL target among the three proteins Aldh1a1, Igfbp5, and MGP, we performed molecular docking and CETSA. The molecular docking results showed that the binding energies between BEL and all three proteins were less than −5 kcal/mol. However, in our CETSA results, only Igfbp5 showed a positive result. Therefore, we identified Igfbp5 as the target of our research and continued to verify its downstream signaling pathway, PI3K-AKT-mTOR.

Although Igfbp5 showed significant differences only in the CoCl_2_ group compared with the control group in the combined transcriptome and proteome analysis, it showed significant differences in the CoCl_2_ + BEL (50 μmol/L) group compared with the CoCl_2_ group in the transcriptome analysis. This may be because the mRNA levels of genes (transcriptome) and their corresponding protein levels (proteome) do not have a simple linear relationship. There are numerous post-transcriptional regulatory steps, and transcript abundance can only partially predict protein abundance. This means that the regulation of gene expression extends far beyond the transcriptional level. Although Igfbp5 mainly regulates the activity of IGF, it also has many independent biological functions beyond IGF, including inflammatory response, cell adhesion, cell migration, cell proliferation, and phenotypic transformation [66,67,68,69,70]. As a member of the IGF family, Igfbp5 primarily influences the PI3K-AKT-mTOR pathway indirectly by regulating the bioavailability of IGF to its receptors, such as IGF-1R [71]. When IGF-1 binds to IGF-1R, it can activate PI3K, which in turn leads to the production of phosphatidylinositol-3,4,5-trisphosphate (PIP3). PIP3 then recruits and activates Akt, which further phosphorylates and regulates multiple downstream targets, including mTOR [71,72]. As a key downstream effector molecule, mTOR regulates processes such as cell growth, proliferation, metabolism, and protein synthesis [73,74,75]. Multiple studies have shown that abnormal activation of the PI3K-AKT-mTOR signaling pathway is closely associated with the development and progression of PH. In PH, HIF-1 can activate the AKT-mTOR pathway, promoting PASMC proliferation [76]. Resistin-like molecule β (RELM-β) acts as a mitogenic factor in PH, promoting PASMC proliferation through Ca^2+^-dependent PI3K-AkT-mTOR and PKC-MAPK signaling pathways [77]. Tripartite motif protein 32 (TRIM32) inhibits the proliferation and migration of PASMCs in PH by inactivating the PI3K-AKT pathway [78]. In summary, the PI3K-AKT-mTOR signaling pathway is an important area for PH pathophysiology research and the development of treatment strategies. Our study still has some limitations and requires further experimental verification in vivo.

## 5. Novelty, Limitations, and Future Directions

This study demonstrated that BEL may induce the relaxation of pulmonary arteries, primarily through effects on the vascular smooth muscle layer. BEL may inhibit CoCl_2_-induced proliferation, migration, and phenotypic switching of PASMCs by regulating the PI3K-AKT-mTOR pathway through mechanisms involving Igfbp5. These findings provide preliminary evidence supporting the potential therapeutic values of BEL in preventing and treating PH. However, several limitations should be acknowledged.

- This study used CoCl_2_-induced chemical hypoxia as a model, which cannot fully replicate the physiological hypoxic conditions. Future studies should employ authentic hypoxic conditions to validate these findings.
- Due to experimental limitations, CETSA was used to identify Igfbp5 as a potential target of BEL. Although the results suggest a possible interaction, additional target-validation approaches, such as SPR, are needed to confirm the direct target of BEL.
- The present study was conducted primarily at the cellular level and lacks in vivo experimental data. Therefore, future studies will establish animal models of pulmonary hypertension to further investigate the effects of BEL, its molecular targets, and its associated signaling pathways in vivo. Such studies should provide more comprehensive and reliable evidence supporting our findings.

## 6. Conclusions

BEL dilated the pulmonary artery in a concentration-dependent manner and improved pulmonary arterial remodeling; this dilation effect mainly depended on the relaxation of the smooth muscle layer. BEL might regulate the PI3K-AKT-mTOR pathway by downregulating Igfbp5, effectively suppressing the proliferation, migration, and phenotypic transformation of PASMCs induced by CoCl_2_, thereby preventing and treating PH. Pulmonary arterial contraction and remodeling are two key pathological mechanisms of PH. Our research results indicated that BEL may have therapeutic effects on PH.

## Figures and Tables

**Table 1:** The primers used for qPCR.

Gene	Species	Primers	Sequence (5′→3′)
*Actb*	Rat	Forward primer	CCCTAAGGCCAACCGTGAA
		Reverse primer	CACGCACGATTTCCCTCTCA
*Acta2*	Rat	Forward primer	GGACGTACAACTGGTATTGTGC
		Reverse primer	TCGGCAGTAGTCACGAAGGA
*Tagln*	Rat	Forward primer	CTTGAAGGCAGCTGAGGATTAT
		Reverse primer	CAAACTGCCCAAAGCCATTAC
*Pcna*	Rat	Forward primer	CAATTTCTAGCAACGCCTAAGAT
		Reverse primer	AAGAGGAAGCTGTGTCCATAGAG
*Spp1*	Rat	Forward primer	AGACCAGCCATGAGTCAAGTCA
		Reverse primer	TGAAACTCGTGGCTCTGATGTT
*Vim*	Rat	Forward primer	CAATGCTTCTCTGGCACGTC
		Reverse primer	GGAAACGTCCACATCGATCTG
*Igfbp5*	Rat	Forward primer	CCCTGCGACGAGAAAGCTC
		Reverse primer	GCTCTTTTCGTTGAGGCAAACC
*Aldh1A1*	Rat	Forward primer	GGGCTGACAAGATTCATGGT
		Reverse primer	GGAAAATTCCAGGGGATGAT
*Mgp*	Rat	Forward primer	TGCCAACACCTTTATATCCC
		Reverse primer	AGACTCCGAAACAAAGCGACT

**Table 2:** The effects of Bellidifolin on the Endo+ and Endo− pulmonary artery rings precontracted by NE.

	Emax	EC_50_
intact endothelium pulmonary artery ring (Endo+)	(92.08 ± 3.84)%	(16.91 ± 3.18) μmol/L
endothelium-denuded pulmonary artery ring (Endo−)	(89.24 ± 4.73)%	(17.40 ± 2.12) μmol/L

## Data Availability

The original contributions presented in this study are included in the article/Supplementary Materials. Further inquiries can be directed to the corresponding author(s).

## Supplementary Materials

The following supporting information can be downloaded at: https://www.mdpi.com/article/10.3390/biom16071059/s1, The references cited in the Supplementary Materials can be found in the reference list of the main text [33,79,80,81,82,83,84]. Figure S1. Morphological identification and immunocytochemistry staining of rat PASMCs. Figure S2. Establishment of a primary PASMCs proliferation model induced by CoCl_2_. File S1. Original western blot images.

## References

[1] Hassoun P.M. (2021). Pulmonary Arterial Hypertension. N. Engl. J. Med..

[2] Agrawal V., Kropski J.A., Gokey J.J., Kobeck E., Murphy M.B., Murray K.T., Fortune N.L., Moore C.S., Meoli D.F., Monahan K. (2023). Myeloid Cell Derived IL1β Contributes to Pulmonary Hypertension in HFpEF. Circ. Res..

[3] Agrawal V., Lahm T., Hansmann G., Hemnes A.R. (2020). Molecular mechanisms of right ventricular dysfunction in pulmonary arterial hypertension: Focus on the coronary vasculature, sex hormones, and glucose/lipid metabolism. Cardiovasc. Diagn. Ther..

[4] Zhang J., Gu X., Cheng T.L., Qi Y.J., Liu D.Y., Wu N., Wang D.P., Huang Y., Zhu Z.M., Fan Y. (2025). ASH2L Deficiency in Smooth Muscle Drives Pulmonary Vascular Remodeling. Circ. Res..

[5] Hoeper M.M., Humbert M., Souza R., Idrees M., Kawut S.M., Sliwa-Hahnle K., Jing Z.C., Gibbs J.S. (2016). A global view of pulmonary hypertension. Lancet Respir. Med..

[6] Jeong E.M., Pereira M., So E.Y., Wu K.Q., Del Tatto M., Wen S., Dooner M.S., Dubielecka P.M., Reginato A.M., Ventetuolo C.E. (2022). Targeting RUNX1 as a novel treatment modality for pulmonary arterial hypertension. Cardiovasc. Res..

[7] Zheng W., Wang Z., Jiang X., Zhao Q., Shen J. (2020). Targeted Drugs for Treatment of Pulmonary Arterial Hypertension: Past, Present, and Future Perspectives. J. Med. Chem..

[8] Bhogal S., Khraisha O., Al Madani M., Treece J., Baumrucker S.J., Paul T.K. (2019). Sildenafil for Pulmonary Arterial Hypertension. Am. J. Ther..

[9] Stenmark K.R., Frid M.G. (1998). Smooth muscle cell heterogeneity: Role of specific smooth muscle cell subpopulations in pulmonary vascular disease. Chest.

[10] Ma B., Cao Y., Qin J., Chen Z., Hu G., Li Q. (2023). Pulmonary artery smooth muscle cell phenotypic switching: A key event in the early stage of pulmonary artery hypertension. Drug Discov. Today.

[11] Campbell J.H., Campbell G.R. (1986). Endothelial cell influences on vascular smooth muscle phenotype. Annu. Rev. Physiol..

[12] Gordon D., Schwartz S.M. (1987). Replication of arterial smooth muscle cells in hypertension and atherosclerosis. Am. J. Cardiol..

[13] Owens G.K., Kumar M.S., Wamhoff B.R. (2004). Molecular regulation of vascular smooth muscle cell differentiation in development and disease. Physiol. Rev..

[14] Paulin D., Lilienbaum A., Kardjian S., Agbulut O., Li Z. (2022). Vimentin: Regulation and pathogenesis. Biochimie.

[15] Shirakawa K., Sano M. (2021). Osteopontin in Cardiovascular Diseases. Biomolecules.

[16] Liu J., Ren Y., Kang L., Zhang L. (2014). Oxidized low-density lipoprotein increases the proliferation and migration of human coronary artery smooth muscle cells through the upregulation of osteopontin. Int. J. Mol. Med..

[17] Seo K.W., Lee S.J., Ye B.H., Kim Y.W., Bae S.S., Kim C.D. (2015). Mechanical stretch enhances the expression and activity of osteopontin and MMP-2 via the Akt1/AP-1 pathways in VSMC. J. Mol. Cell Cardiol..

[18] Zhang Q., Cao Y., Luo Q., Wang P., Shi P., Song C., E M., Ren J., Fu B., Sun H. (2018). The transient receptor potential vanilloid-3 regulates hypoxia-mediated pulmonary artery smooth muscle cells proliferation via PI3K/AKT signaling pathway. Cell Prolif..

[19] Crosswhite P., Sun Z. (2014). Molecular mechanisms of pulmonary arterial remodeling. Mol. Med..

[20] Yang L., Wan N., Gong F., Wang X., Feng L., Liu G. (2023). Transcription factors and potential therapeutic targets for pulmonary hypertension. Front. Cell Dev. Biol..

[21] Yan L., Yali L., Chenghao L., Caiqin F., Zhongbo Z., Weiyu R., Yu M., Xiaotian Z., Biwen W., Xiaojie J. (2020). Bellidifolin Inhibits Proliferation of A549 Cells by Regulating STAT3/COX-2 Expression and Protein Activity. J. Oncol..

[22] Zhou D., Liu W., Zhang J., Dong Y., Wu J., Zhang Y., Dai C., Zhang T., Yang G., Zhang Y. (2023). Bellidifolin ameliorates isoprenaline-induced cardiac hypertrophy by the Nox4/ROS signalling pathway through inhibiting BRD4. Cell Death Discov..

[23] Warburg O. (1956). On the Origin of Cancer Cells. Science.

[24] Zhu X., Xuan Z., Chen J., Li Z., Zheng S., Song P. (2020). How DNA methylation affects the Warburg effect. Int. J. Biol. Sci..

[25] Chen X.S., Li L.Y., Guan Y.D., Yang J.M., Cheng Y. (2016). Anticancer strategies based on the metabolic profile of tumor cells: Therapeutic targeting of the Warburg effect. Acta Pharmacol. Sin..

[26] Dai J., Zhou Q., Chen J., Rexius-Hall M.L., Rehman J., Zhou G. (2018). Alpha-enolase regulates the malignant phenotype of pulmonary artery smooth muscle cells via the AMPK-Akt pathway. Nat. Commun..

[27] Wujak M., Veith C., Wu C.Y., Wilke T., Kanbagli Z.I., Novoyatleva T., Guenther A., Seeger W., Grimminger F., Sommer N. (2021). Adenylate Kinase 4-A Key Regulator of Proliferation and Metabolic Shift in Human Pulmonary Arterial Smooth Muscle Cells via Akt and HIF-1α Signaling Pathways. Int. J. Mol. Sci..

[28] Shimauchi T., Boucherat O., Yokokawa T., Grobs Y., Wu W., Orcholski M., Martineau S., Omura J., Tremblay E., Shimauchi K. (2022). PARP1-PKM2 Axis Mediates Right Ventricular Failure Associated With Pulmonary Arterial Hypertension. JACC Basic Transl. Sci..

[29] Gai X.Y., Wei Y.H., Zhang W., Wuren T.N., Wang Y.P., Li Z.Q., Liu S., Ma L., Lu D.X., Zhou Y. (2015). Echinacoside induces rat pulmonary artery vasorelaxation by opening the NO-cGMP-PKG-BKCa channels and reducing intracellular Ca^2+^ levels. Acta Pharmacol. Sin..

[30] Paggi J.M., Pandit A., Dror R.O. (2024). The Art and Science of Molecular Docking. Annu. Rev. Biochem..

[31] Gao P., Liu Y.Q., Xiao W., Xia F., Chen J.Y., Gu L.W., Yang F., Zheng L.H., Zhang J.Z., Zhang Q. (2022). Identification of antimalarial targets of chloroquine by a combined deconvolution strategy of ABPP and MS-CETSA. Mil. Med. Res..

[32] Han W., Liu G.N. (2010). EGR-1 decoy ODNs inhibit vascular smooth muscle cell proliferation and neointimal hyperplasia of balloon-injured arteries in rat. Life Sci..

[33] Zhou C., Guo H., Meng L., Ji Z. (2016). The inlfuence of two vascular smooth muscle cell primary culture methods on cellular contractile pheno-type. J. Wenzhou Med. Univ..

[34] Morgan C.A., Hurley T.D. (2015). Characterization of two distinct structural classes of selective aldehyde dehydrogenase 1A1 inhibitors. J. Med. Chem..

[35] Ciccone V., Terzuoli E., Ristori E., Filippelli A., Ziche M., Morbidelli L., Donnini S. (2022). ALDH1A1 overexpression in melanoma cells promotes tumor angiogenesis by activating the IL-8/Notch signaling cascade. Int. J. Mol. Med..

[36] Rinker T.E., Philbrick B.D., Hettiaratchi M.H., Smalley D.M., McDevitt T.C., Temenoff J.S. (2018). Microparticle-mediated sequestration of cell-secreted proteins to modulate chondrocytic differentiation. Acta Biomater..

[37] Ma J., Sawai H., Matsuo Y., Ochi N., Yasuda A., Takahashi H., Wakasugi T., Funahashi H., Sato M., Takeyama H. (2010). IGF-1 mediates PTEN suppression and enhances cell invasion and proliferation via activation of the IGF-1/PI3K/Akt signaling pathway in pancreatic cancer cells. J. Surg. Res..

[38] Bionaz M., Loor J.J. (2008). Gene networks driving bovine milk fat synthesis during the lactation cycle. BMC Genom..

[39] Sobolewska A., Gajewska M., Zarzyńska J., Gajkowska B., Motyl T. (2009). IGF-I, EGF, and sex steroids regulate autophagy in bovine mammary epithelial cells via the mTOR pathway. Eur. J. Cell Biol..

[40] Li N., Su S., Xie X., Yang Z., Li Z., Lu D. (2024). Tsantan Sumtang, a traditional Tibetan medicine, protects pulmonary vascular endothelial function of hypoxia-induced pulmonary hypertension rats through AKT/eNOS signaling pathway. J. Ethnopharmacol..

[41] Johnson S., Sommer N., Cox-Flaherty K., Weissmann N., Ventetuolo C.E., Maron B.A. (2023). Pulmonary Hypertension: A Contemporary Review. Am. J. Respir. Crit. Care Med..

[42] Li J., Meng Z.Y., Wen H., Lu C.H., Qin Y., Xie Y.M., Chen Q., Lv J.H., Huang F., Zeng Z.Y. (2024). β-sitosterol alleviates pulmonary arterial hypertension by altering smooth muscle cell phenotype and DNA damage/cGAS/STING signaling. Phytomedicine.

[43] Boucherat O., Vitry G., Trinh I., Paulin R., Provencher S., Bonnet S. (2017). The cancer theory of pulmonary arterial hypertension. Pulm. Circ..

[44] Cao Y.Y., Ba H.X., Li Y., Tang S.Y., Luo Z.Q., Li X.H. (2020). Regulatory effects of Prohibitin 1 on proliferation and apoptosis of pulmonary arterial smooth muscle cells in monocrotaline-induced PAH rats. Life Sci..

[45] Wang J., Yan X., Feng W., Wang Q., Shi W., Chai L., Zhang Q., Chen Y., Liu J., Qu Z. (2021). S1P induces proliferation of pulmonary artery smooth muscle cells by promoting YAP-induced Notch3 expression and activation. J. Biol. Chem..

[46] Orozco-Ibarra M., Muñoz-Sánchez J., Zavala-Medina M.E., Pineda B., Magaña-Maldonado R., Vázquez-Contreras E., Maldonado P.D., Pedraza-Chaverri J., Chánez-Cárdenas M.E. (2016). Aged garlic extract and S-allylcysteine prevent apoptotic cell death in a chemical hypoxia model. Biol. Res..

[47] Zheng F., Chen P., Li H., Aschner M. (2020). Drp-1-Dependent Mitochondrial Fragmentation Contributes to Cobalt Chloride-Induced Toxicity in *Caenorhabditis elegans*. Toxicol. Sci..

[48] Hopkins C.D., Wessel C., Chen O., El-Kersh K., Cave M.C., Cai L., Huang J. (2023). Potential Roles of Metals in the Pathogenesis of Pulmonary and Systemic Hypertension. Int. J. Biol. Sci..

[49] Knox M., Vinet R., Fuentes L., Morales B., Martínez J.L. (2019). A Review of Endothelium-Dependent and -Independent Vasodilation Induced by Phytochemicals in Isolated Rat Aorta. Animals.

[50] Dunham-Snary K.J., Wu D., Sykes E.A., Thakrar A., Parlow L.R.G., Mewburn J.D., Parlow J.L., Archer S.L. (2017). Hypoxic Pulmonary Vasoconstriction: From Molecular Mechanisms to Medicine. Chest.

[51] Trufanov S.K., Rybakova E.Y., Avdonin P.P., Tsitrina A.A., Zharkikh I.L., Goncharov N.V., Jenkins R.O., Avdonin P.V. (2019). The Role of Two-Pore Channels in Norepinephrine-Induced [Ca^2+^]i Rise in Rat Aortic Smooth Muscle Cells and Aorta Contraction. Cells.

[52] Qu J.T., Zhang D.X., Liu F., Mao H.P., Ma Y.K., Yang Y., Li C.X., Qiu L.Z., Geng X., Zhang J.M. (2015). Vasodilatory Effect of Wogonin on the Rat Aorta and Its Mechanism Study. Biol. Pharm. Bull..

[53] Li H., Zhong Y., Cao G., Shi H., Liu Y., Li L., Yin P., Chen J., Xiao Z., Du B. (2022). METTL3 promotes cell cycle progression via m^6^A/YTHDF1-dependent regulation of CDC25B translation. Int. J. Biol. Sci..

[54] Wang Y.T., Chen J., Li X., Umetani M., Chen Y., Li P.L., Zhang Y. (2019). Contribution of transcription factor EB to adipoRon-induced inhibition of arterial smooth muscle cell proliferation and migration. Am. J. Physiol. Cell Physiol..

[55] Trepat X., Chen Z., Jacobson K. (2012). Cell migration. Compr. Physiol..

[56] Riascos-Bernal D.F., Ressa G., Korrapati A., Sibinga N.E.S. (2023). The FAT1 Cadherin Drives Vascular Smooth Muscle Cell Migration. Cells.

[57] Roostalu U., Aldeiri B., Albertini A., Humphreys N., Simonsen-Jackson M., Wong J.K.F., Cossu G. (2018). Distinct Cellular Mechanisms Underlie Smooth Muscle Turnover in Vascular Development and Repair. Circ. Res..

[58] Wang G., Jacquet L., Karamariti E., Xu Q. (2015). Origin and differentiation of vascular smooth muscle cells. J. Physiol..

[59] Lechartier B., Berrebeh N., Huertas A., Humbert M., Guignabert C., Tu L. (2022). Phenotypic Diversity of Vascular Smooth Muscle Cells in Pulmonary Arterial Hypertension: Implications for Therapy. Chest.

[60] Liu M., Gomez D. (2019). Smooth Muscle Cell Phenotypic Diversity. Arterioscler. Thromb. Vasc. Biol..

[61] Huang S., Chen P., Shui X., He Y., Wang H., Zheng J., Zhang L., Li J., Xue Y., Chen C. (2014). Baicalin attenuates transforming growth factor-β1-induced human pulmonary artery smooth muscle cell proliferation and phenotypic switch by inhibiting hypoxia inducible factor-1α and aryl hydrocarbon receptor expression. J. Pharm. Pharmacol..

[62] Yan G., Sun R., Chen Z., Pan X., Sheng Z., Tang C. (2021). PTBP1 Targets ILK to Regulate the Hypoxia-Induced Phenotypic Transformation of Pulmonary Artery Smooth Muscle Cells. Drug Des. Dev. Ther..

[63] Oohashi T., Ueki Y., Sugimoto M., Ninomiya Y. (1995). Isolation and structure of the COL4A6 gene encoding the human alpha 6(IV) collagen chain and comparison with other type IV collagen genes. J. Biol. Chem..

[64] Wu Y.H., Wu P.Y., Huang Y.F., Chen C.C., Huang S.C., Cheng-Yang C. (2025). Collagen type IV alpha 6 promotes tumor progression and chemoresistance in ovarian cancer by activating the discoidin domain receptor 1 pathway. Oncogenesis.

[65] Sun Q., Wang H., Xie J., Wang L., Mu J., Li J., Ren Y., Lai L. (2025). Computer-Aided Drug Discovery for Undruggable Targets. Chem. Rev..

[66] Nguyen X.X., Muhammad L., Nietert P.J., Feghali-Bostwick C. (2018). IGFBP-5 Promotes Fibrosis via Increasing Its Own Expression and That of Other Pro-fibrotic Mediators. Front. Endocrinol..

[67] Sureshbabu A., Okajima H., Yamanaka D., Tonner E., Shastri S., Maycock J., Szymanowska M., Shand J., Takahashi S., Beattie J. (2012). IGFBP5 induces cell adhesion, increases cell survival and inhibits cell migration in MCF-7 human breast cancer cells. J. Cell Sci..

[68] Simon S., Grabellus F., Ferrera L., Galietta L., Schwindenhammer B., Mühlenberg T., Taeger G., Eilers G., Treckmann J., Breitenbuecher F. (2013). DOG1 regulates growth and IGFBP5 in gastrointestinal stromal tumors. Cancer Res..

[69] Liu L., Wang J., Li X., Ma J., Shi C., Zhu H., Xi Q., Zhang J., Zhao X., Gu M. (2015). MiR-204-5p suppresses cell proliferation by inhibiting IGFBP5 in papillary thyroid carcinoma. Biochem. Biophys. Res. Commun..

[70] Xiang A., Guan H., Su P., Zhang L., Chen X., Yu Q. (2025). IGFBP5 Promotes Atherosclerosis in APOE(-/-) Mice Through Phenotypic Transformation of VSMCs. Curr. Issues Mol. Biol..

[71] Chen J., Alberts I., Li X. (2014). Dysregulation of the IGF-I/PI3K/AKT/mTOR signaling pathway in autism spectrum disorders. Int. J. Dev. Neurosci..

[72] Deng H., Chen Y., Li P., Hang Q., Zhang P., Jin Y., Chen M. (2023). PI3K/AKT/mTOR pathway, hypoxia, and glucose metabolism: Potential targets to overcome radioresistance in small cell lung cancer. Cancer Pathog. Ther..

[73] Janku F., Yap T.A., Meric-Bernstam F. (2018). Targeting the PI3K pathway in cancer: Are we making headway?. Nat. Rev. Clin. Oncol..

[74] Glaviano A., Foo A.S.C., Lam H.Y., Yap K.C.H., Jacot W., Jones R.H., Eng H., Nair M.G., Makvandi P., Geoerger B. (2023). PI3K/AKT/mTOR signaling transduction pathway and targeted therapies in cancer. Mol. Cancer.

[75] Tewari D., Patni P., Bishayee A., Sah A.N., Bishayee A. (2022). Natural products targeting the PI3K-Akt-mTOR signaling pathway in cancer: A novel therapeutic strategy. Semin. Cancer Biol..

[76] Liu P., Gu Y., Luo J., Ye P., Zheng Y., Yu W., Chen S. (2019). Inhibition of Src activation reverses pulmonary vascular remodeling in experimental pulmonary arterial hypertension via Akt/mTOR/HIF-1<alpha> signaling pathway. Exp. Cell Res..

[77] Tian H., Liu L., Wu Y., Wang R., Jiang Y., Hu R., Zhu L., Li L., Fang Y., Yang C. (2021). Resistin-like molecule β acts as a mitogenic factor in hypoxic pulmonary hypertension via the Ca^2+^-dependent PI3K/Akt/mTOR and PKC/MAPK signaling pathways. Respir. Res..

[78] Hu Z., Song Q., Ma H., Guo Y., Zhang T., Xie H., Luo X. (2021). TRIM32 inhibits the proliferation and migration of pulmonary artery smooth muscle cells through the inactivation of PI3K/Akt pathway in pulmonary arterial hypertension. J. Bioenerg. Biomembr..

[79] Ran C., Lu M., Zhao F., Hao Y., Guo X., Li Y., Su Y., Wang H. (2024). Ginsenoside Rg1 alleviates vascular remodeling in hypoxia-induced pulmonary hypertension mice through the calpain-1/STAT3 signaling pathway. J. Ginseng Res..

[80] Jiao Q., Zou F., Li S., Wang J., Xiao Y., Guan Z., Dong L., Tian J., Li S., Wang R. (2022). Dexlansoprazole prevents pulmonary artery hypertension by inhibiting pulmonary artery smooth muscle cell to fibroblast transition. Am. J. Transl. Res..

[81] Muñoz-Sánchez J., Chánez-Cárdenas M.E. (2018). The use of cobalt chloride as a chemical hypoxia model. J. Appl. Toxicol..

[82] Li Q., Ma R., Zhang M. (2017). CoCl_2_ increases the expression of hypoxic markers HIF-1α, VEGF and CXCR4 in breast cancer MCF-7 cells. Oncol. Lett..

[83] Zhang C., Chen M., Tao Q., Chi Z. (2021). Cobalt chloride-stimulated hypoxia promotes the proliferation of cholesteatoma keratinocytes via the PI3K/Akt signaling pathway. Int. J. Med. Sci..

[84] Wang Q., Lu G., Chen Z. (2018). MALAT1 promoted cell proliferation and migration via MALAT1/miR-155/MEF2A pathway in hypoxia of cardiac stem cells. J. Cell. Biochem..

