# Supplementary material for: Bellidifolin Improves Pulmonary Artery Smooth Muscle Cells Proliferation by Targeting the IGFBP5-Mediated PI3K-AKT-mTOR Pathway and Dilates the Pulmonary Artery
Source: Biomolecules. 2026 Jul 19;16(7):1059. doi: 10.3390/biom16071059 (PMC13406516; doi:10.3390/biom16071059)
Supplement: Supplementary file 1 [file biomolecules-16-01059-s001.zip › biomolecules-4422122-supplementary.pdf]

Supplementary Materials

Bellidifolin Improves Pulmonary Arterial Smooth Muscle Cells Proliferation by Targeting the IGFBP5-mediated PI3K-AKT-mTOR Pathway and Dilates the Pulmonary Artery

Qiuqin Hu [1], Hongmai Wang [1], Yujie Qiao [1], Qingqing Xia [1], Jiacheng Hu [1], Xiangyun Gai [*,1], Yulin Li [2], Tao Chen [2], Zhanqiang Li [3]

1 Department of Pharmacy, Qinghai Minzu University, Xining, Qinghai, China
2 Northwest Institute of Plateau Biology, Chinese Academy of Sciences, Xining, China
3 Qinghai University Plateau Medicine Research Center, Xining, China
* Correspondence: 2015002@qhmu.edu.cn; Tel.: +86-15297219426
These authors contributed equally to this work: Qiuqin Hu, Hongmai Wang

Supplemental Methods

Supplemental Figures and Figure Legends

Supplemental References

Supplemental Methods

1. Isolation and Culture of Primary PASMCs

Primary rat PASMCs were isolated using the standard explant culture method [33]. 6-week-old SD rats were anaesthetized with urethane (20%, w/v%, ip) [79,80], the SD rats were soaked in 75% ethanol for 5 min. The heart and lung were collected and quickly immersed in precooled PBS (containing 1% penicillin-streptomycin) to clean the blood on the organ surface. The pulmonary artery was removed and placed in PBS, the endothelial cells were removed, and the adventitia was peeled off to obtain the smooth muscle layer. The tissue was cut into 1×1-mm pieces, and then cultured in DMEM medium containing 20% FBS for 5-7 days. The medium was not replaced during this process. The cells were passaged when confluence in the standard tissue culture (TC)-treated flask reached 80-90%. The cells used in all experiments were between passage 3 and passage 6.

Supplemental Figures and Figure Legends

1. Morphological and Immunohistochemical Identification of PASMCs

The cells moved from the edge of the tissue after 5-7 days of adhering to the bottom of the well (Figure S1 a-b). After passage, the cells were spindle-shaped or long spindle-shaped under observation at low magnification (Figure S1 c-d). In some areas, the cells were multi-layered, and in others, single-layered, fluctuating and growing in a "peak-valley" pattern with characteristics of vascular smooth muscle cells (VSMCs). α-SMA is a specific protein expressed during the differentiation of smooth muscle cells and is considered a specific marker of smooth muscle cells. In this experiment, an anti-α-SMA antibody was used to identify the isolated and cultured pulmonary artery smooth muscle cells by immunocytochemistry. The immunocytochemistry results showed strong expression of α-SMA on PASMCs, confirming that the cells used were smooth muscle cells. The actin of smooth muscle cells was visible at high magnification, in the cytoplasm of the myofilaments (Figure S1 e-f).

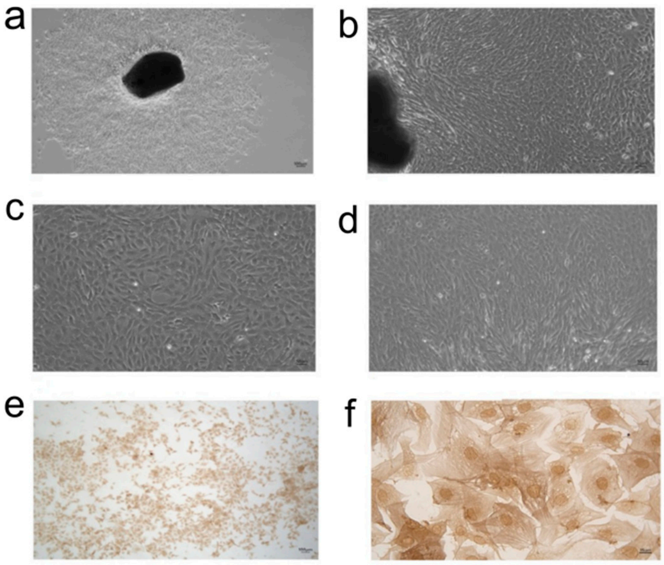

Figure S1. Morphological identification and immunocytochemistry staining of rat PASMCs. (a-b) ×4 magnification. (c-d) ×40 magnification. (e-f) Immunocytochemistry staining of rat PASMCs.

2. Effects of $CoCl_2$ on PASMC Viability in a Concentration- and Time-Dependent Manner

$CoCl_2$ is one of the most widely used chemical agents for mimicking hypoxia in vitro [81]. Within an appropriate concentration range, $CoCl_2$-induced hypoxia can significantly promote cell proliferation [82-84]. In our study, $CoCl_2$ was used to simulate a hypoxic environment and establish a hypoxia-induced cell proliferation model.

The results showed that the relative cell viability of PASMCs after 24 hours of exposure to 25, 50, 100, and 200 μmol/L $CoCl_2$ was higher than that of the control group ($P < 0.001$; S2 a). The relative cell viability of PASMCs after exposure to 400 and 600 μmol/L $CoCl_2$ for 24 hours was lower than that of the control group ($P < 0.001$, Figure S2 a). Thus, 200 μmol/L $CoCl_2$ for 24 hours induced the best proliferation effect on PASMCs compared with the other concentrations. The relative cell viability of PASMCs after exposure to 80 and 100 μmol/L $CoCl_2$ for 48 h was higher than that of the control group ($P < 0.05$, Figure S2 b). Thus, 80 μ mol/L $CoCl_2$ for 48 hours induced the best proliferation effect on PASMCs. The relative cell viability of PASMCs after exposure to 5, 10, and 20 μmol/L $CoCl_2$ for 72 hours was higher than that of the control group ($P < 0.01$, Figure S2 c). The relative cell viability of PASMCs induced by 100, 160, and 200 μmol/L $CoCl_2$ for 72 hours was lower than that of the control group ($P < 0.001$, Figure S2 c). Thus, 10 μmol/L $CoCl_2$ induced the greatest proliferative effect on PASMCs compared with the other concentrations.

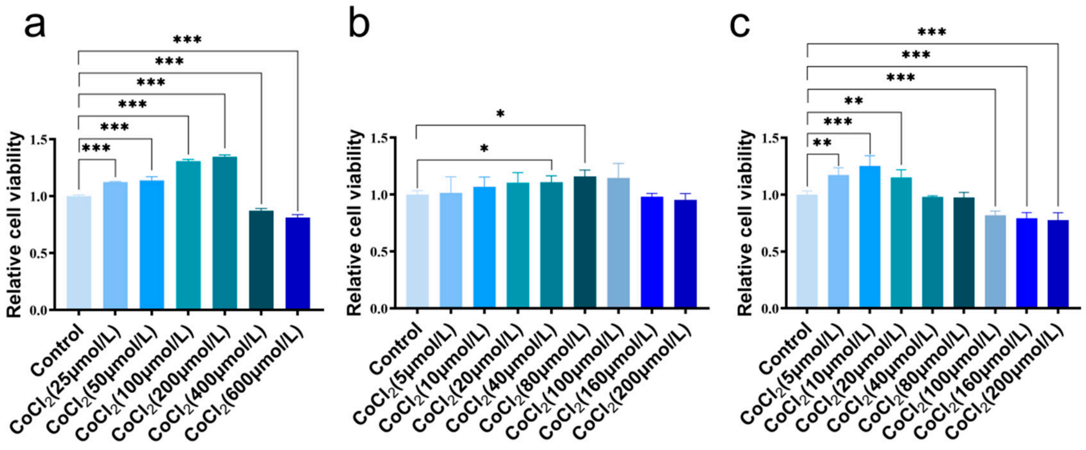

Figure S2. Establishment of a primary PASMCs proliferation model induced by $CoCl_2$. (a) PASMCs treated with $CoCl_2$ at different concentrations for 24 h (n = 3). (b) PASMCs treated with $CoCl_2$ at different concentrations for 48 h (n = 3). (c) PASMCs treated with $CoCl_2$ at different concentrations for 72 h (n = 3). The results are expressed as mean ± SD ($*P < 0.05$ and $**P < 0.01$, $***P < 0.001$ vs. the control group).
