# Supplementary figures and images for: Bellidifolin Improves Pulmonary Artery Smooth Muscle Cells Proliferation by Targeting the IGFBP5-Mediated PI3K-AKT-mTOR Pathway and Dilates the Pulmonary Artery (part 2 of 2)
Source: Biomolecules. 2026 Jul 19;16(7):1059. doi: 10.3390/biom16071059 (PMC13406516; doi:10.3390/biom16071059)

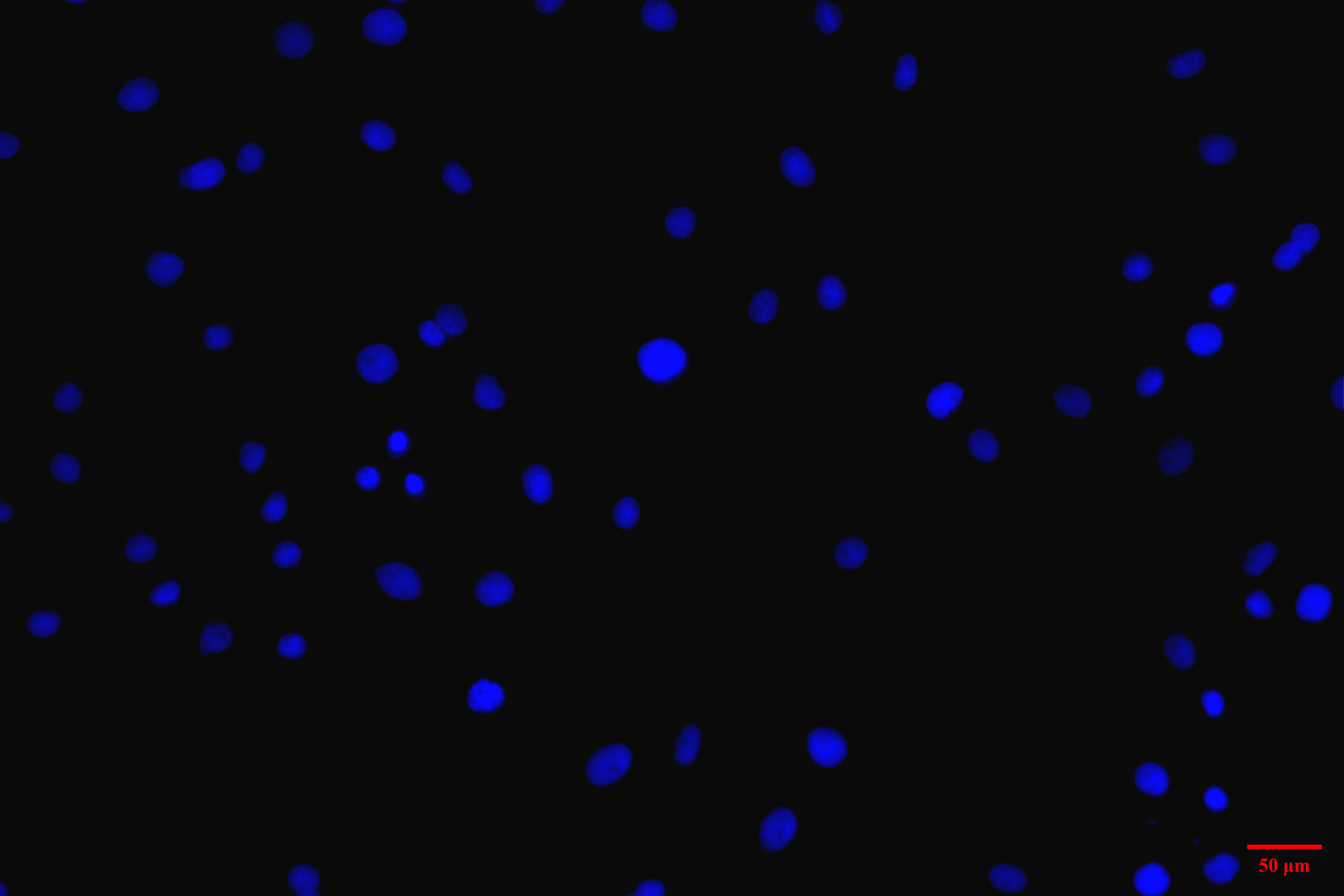

Supplement: Supplementary file 1 [file biomolecules-16-01059-s001.zip › File S1/Figure 6-8-11 Western blot original drawing/Figure 11e/CoCl2+NBI31772/DAPI-NBI-2.jpg]

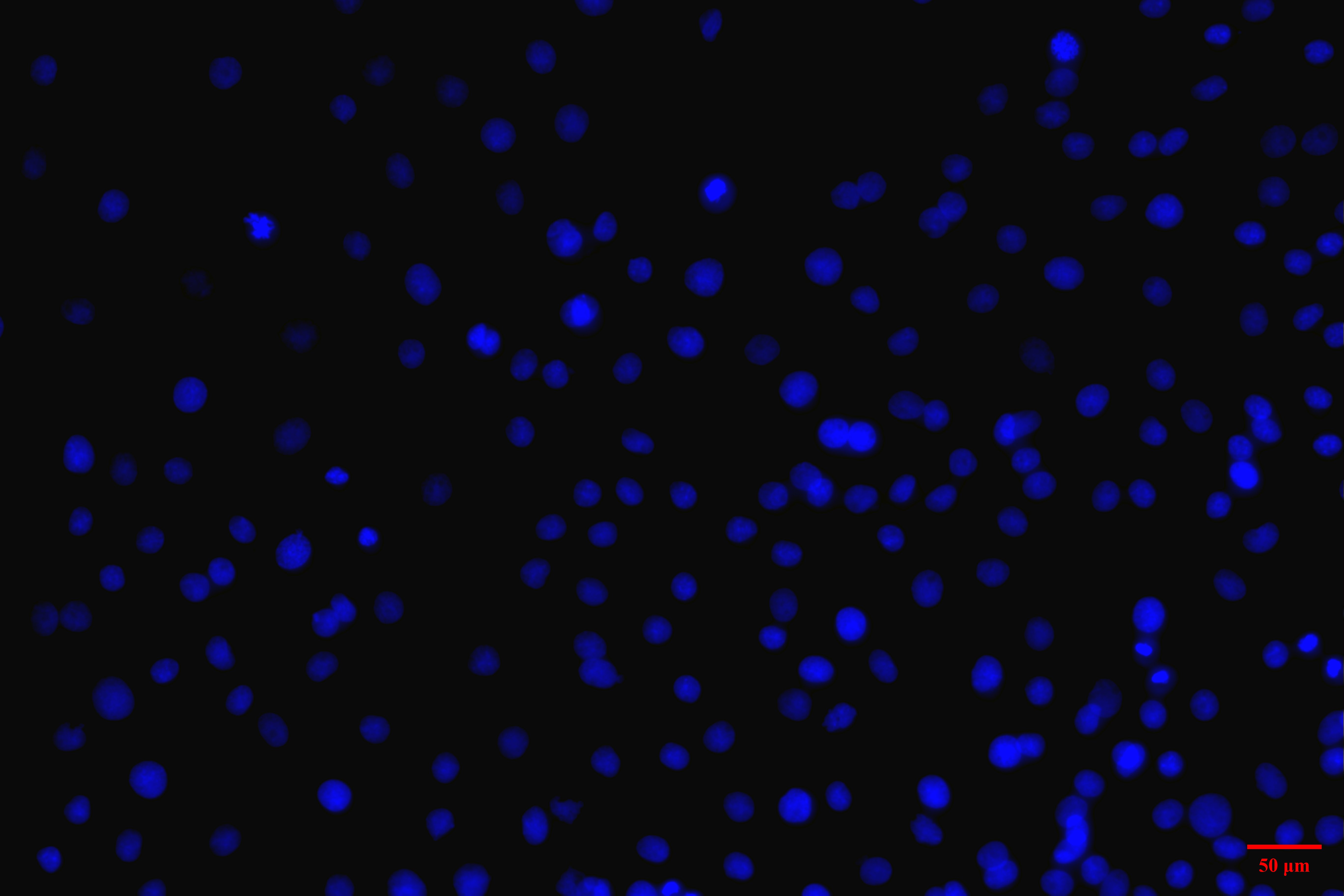

Supplement: Supplementary file 1 [file biomolecules-16-01059-s001.zip › File S1/Figure 6-8-11 Western blot original drawing/Figure 11e/CoCl2+NBI31772/DAPI-NBI-3.jpg]

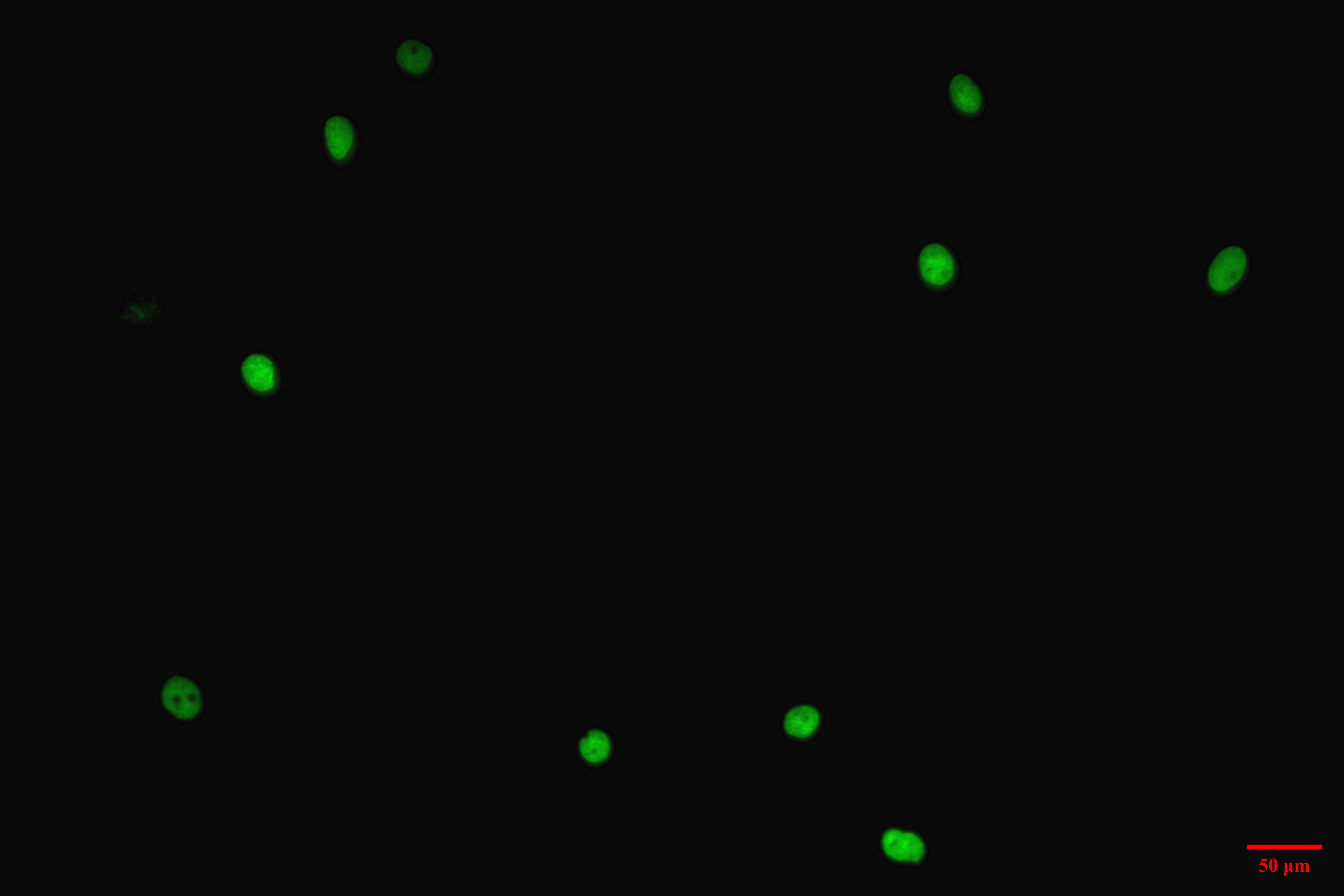

Supplement: Supplementary file 1 [file biomolecules-16-01059-s001.zip › File S1/Figure 6-8-11 Western blot original drawing/Figure 11e/CoCl2+NBI31772/EDU-NBI-1.jpg]

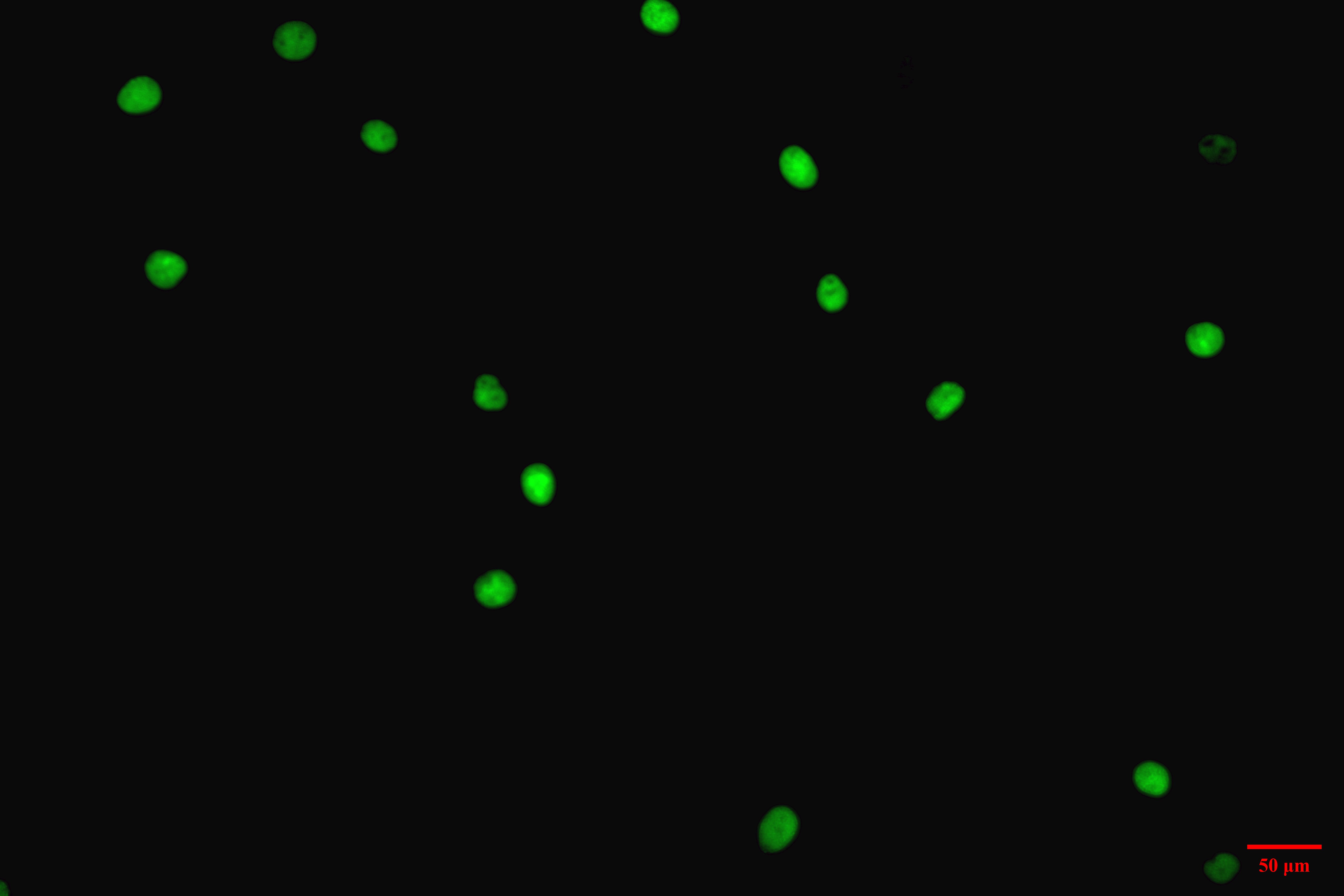

Supplement: Supplementary file 1 [file biomolecules-16-01059-s001.zip › File S1/Figure 6-8-11 Western blot original drawing/Figure 11e/CoCl2+NBI31772/EDU-NBI-2.jpg]

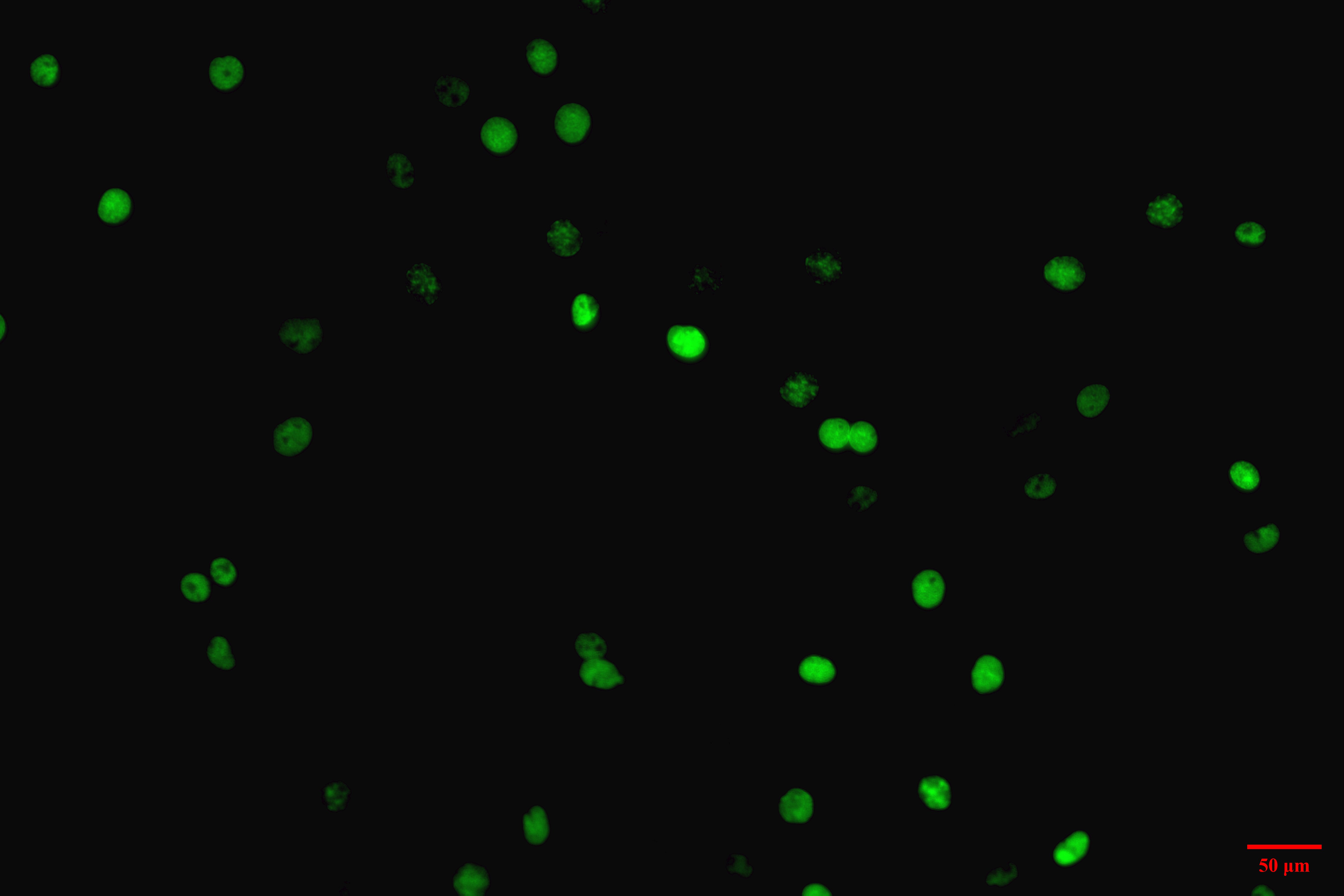

Supplement: Supplementary file 1 [file biomolecules-16-01059-s001.zip › File S1/Figure 6-8-11 Western blot original drawing/Figure 11e/CoCl2+NBI31772/EDU-NBI-3.jpg]

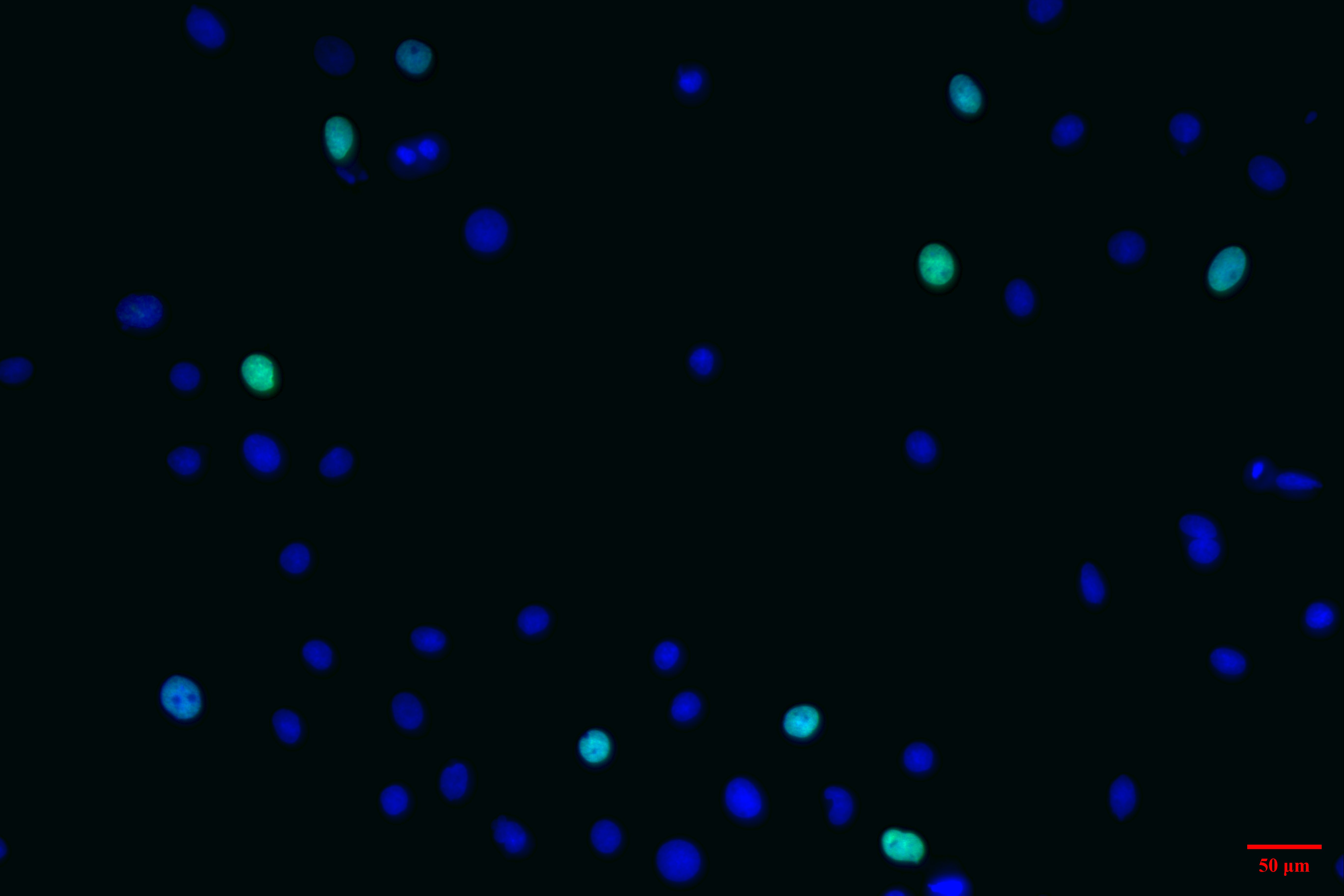

Supplement: Supplementary file 1 [file biomolecules-16-01059-s001.zip › File S1/Figure 6-8-11 Western blot original drawing/Figure 11e/CoCl2+NBI31772/Merge-NBI-1.jpg]

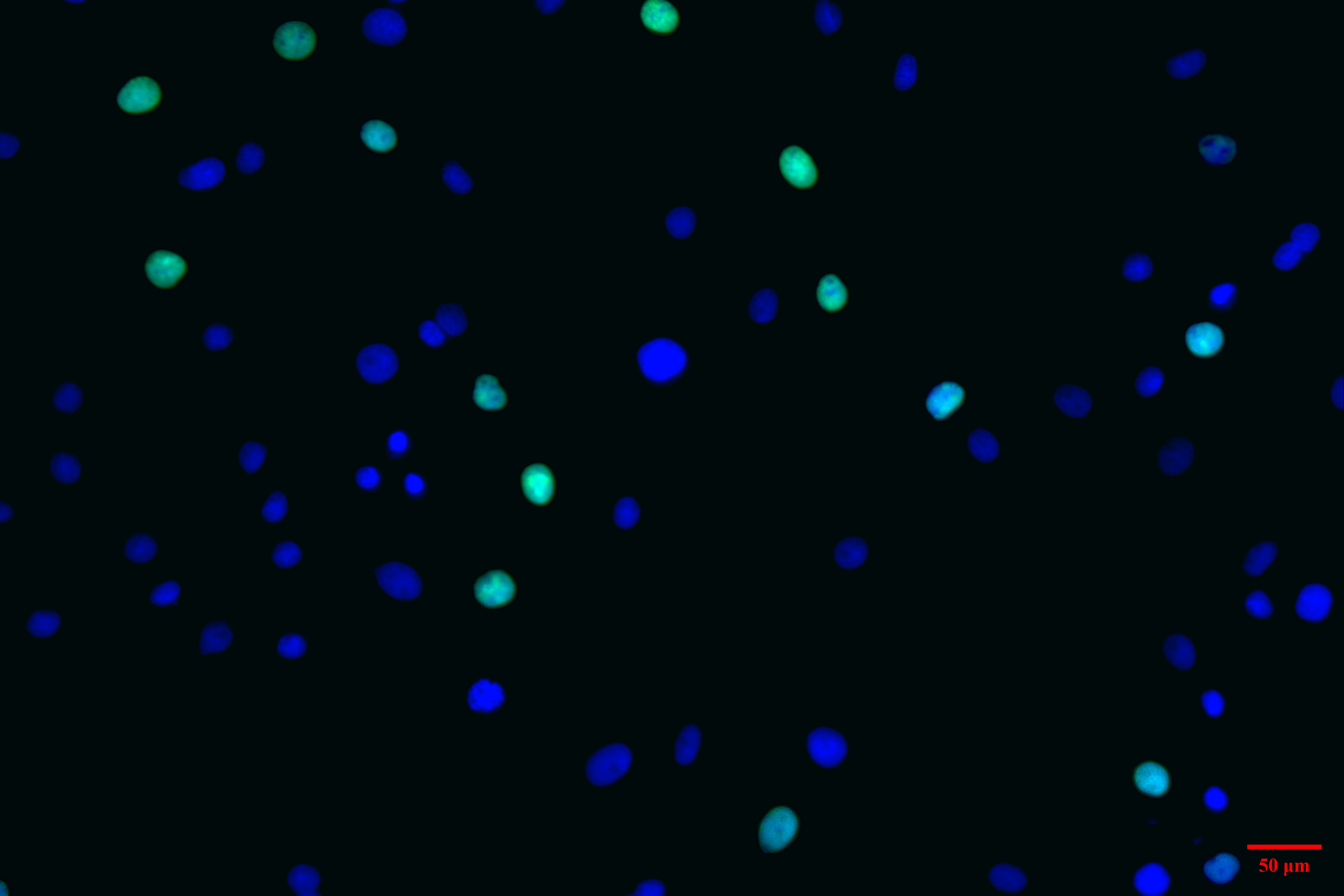

Supplement: Supplementary file 1 [file biomolecules-16-01059-s001.zip › File S1/Figure 6-8-11 Western blot original drawing/Figure 11e/CoCl2+NBI31772/Merge-NBI-2.jpg]

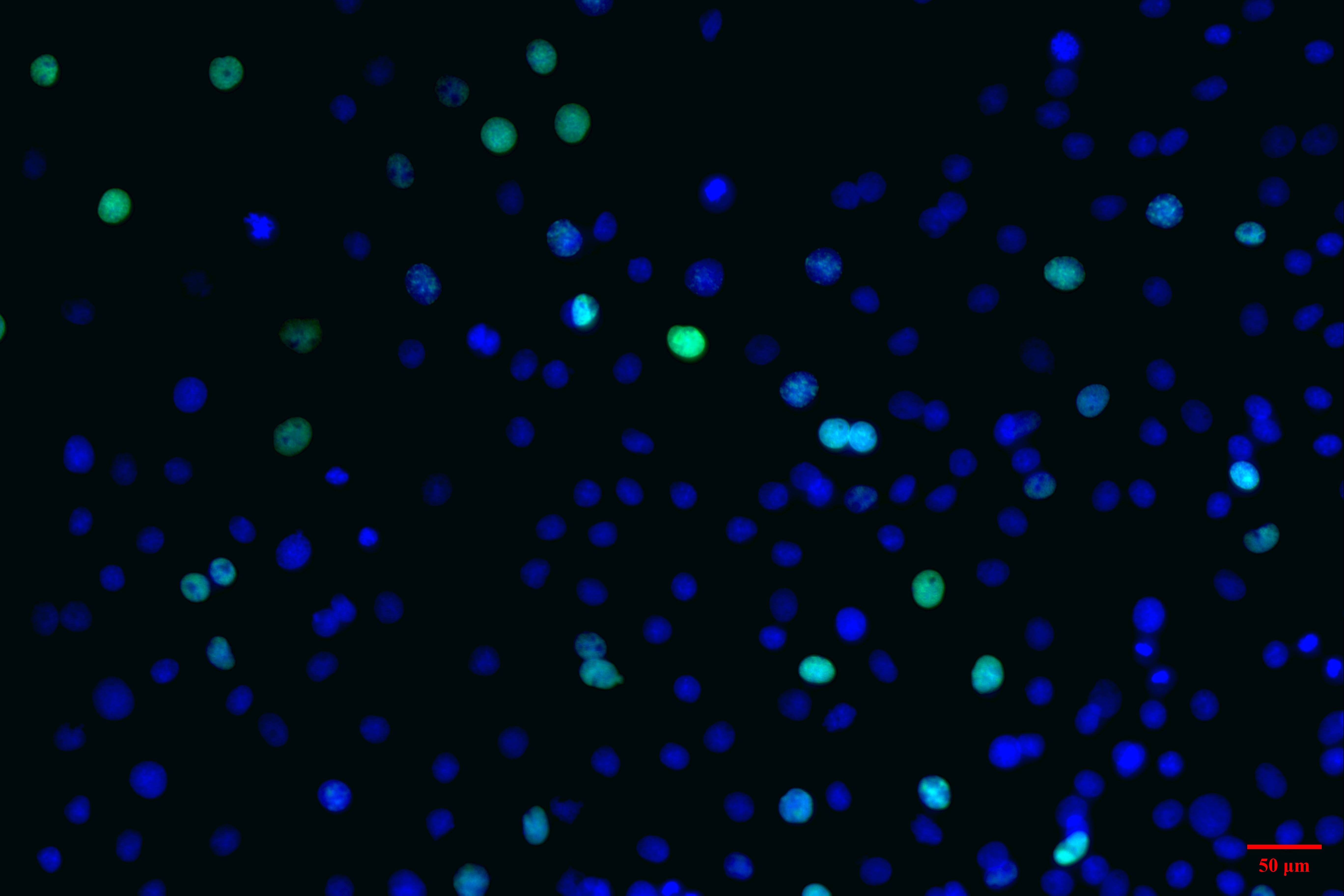

Supplement: Supplementary file 1 [file biomolecules-16-01059-s001.zip › File S1/Figure 6-8-11 Western blot original drawing/Figure 11e/CoCl2+NBI31772/Merge-NBI-3.jpg]

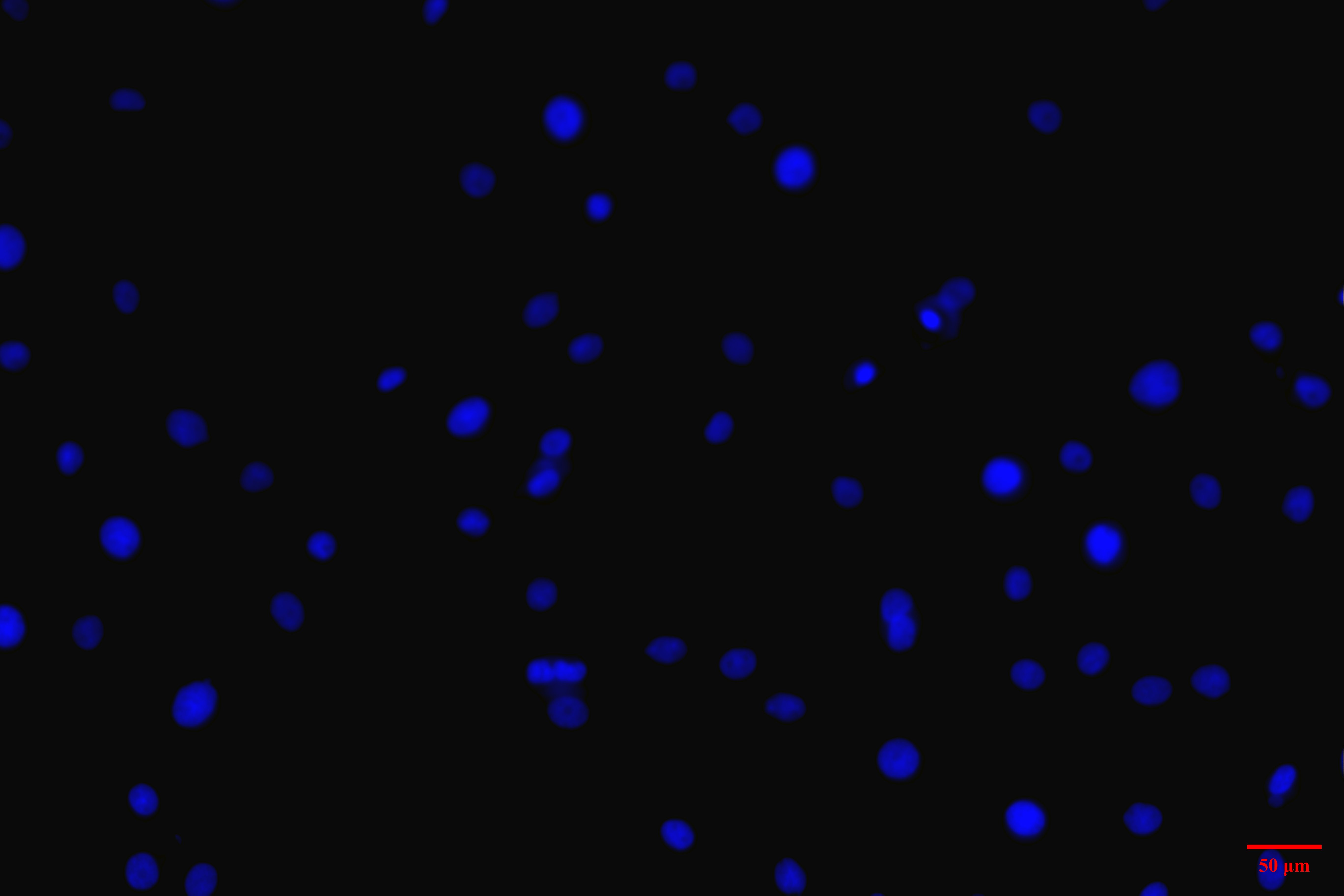

Supplement: Supplementary file 1 [file biomolecules-16-01059-s001.zip › File S1/Figure 6-8-11 Western blot original drawing/Figure 11e/control/DAPI-C-1.jpg]

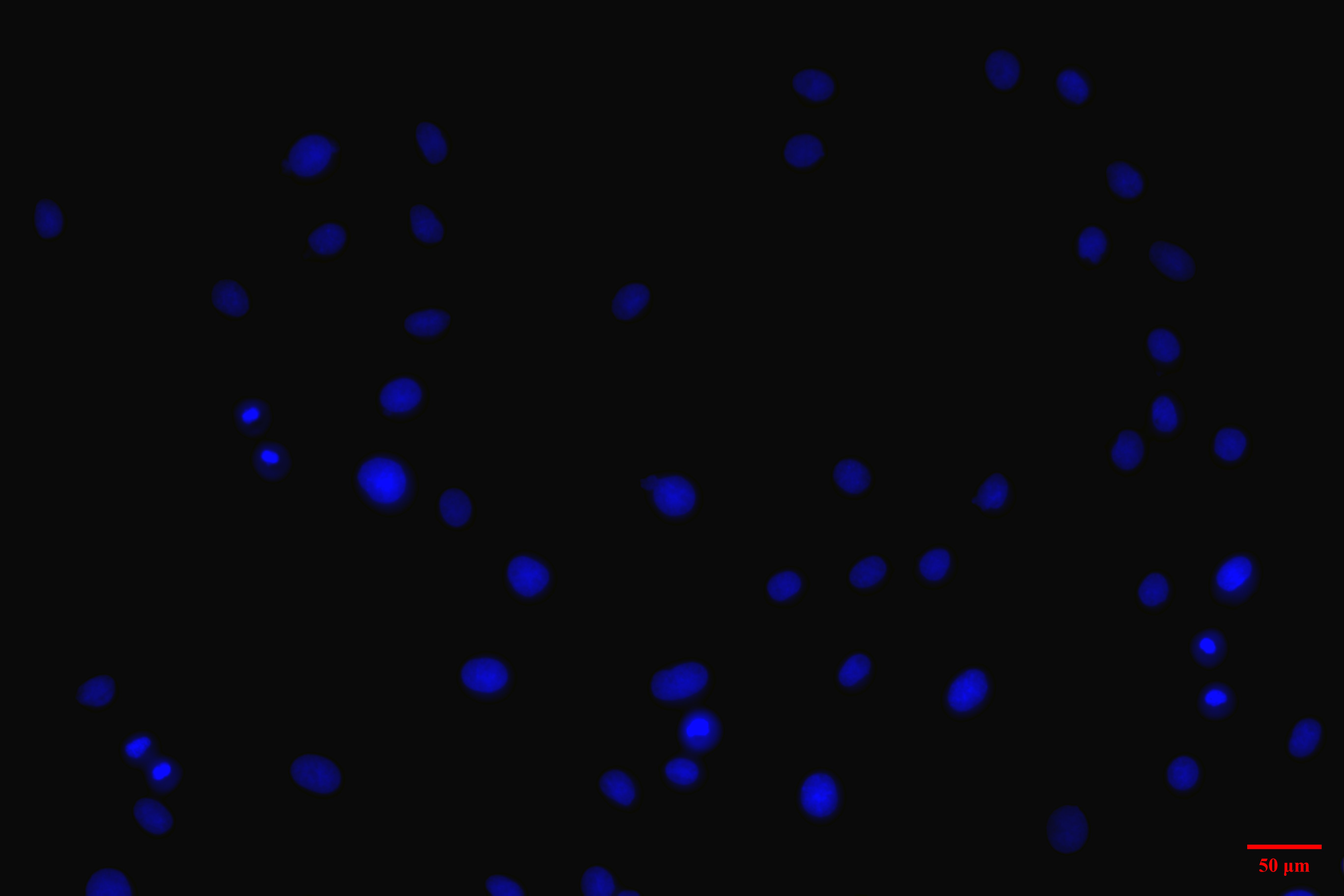

Supplement: Supplementary file 1 [file biomolecules-16-01059-s001.zip › File S1/Figure 6-8-11 Western blot original drawing/Figure 11e/control/DAPI-C-2.jpg]

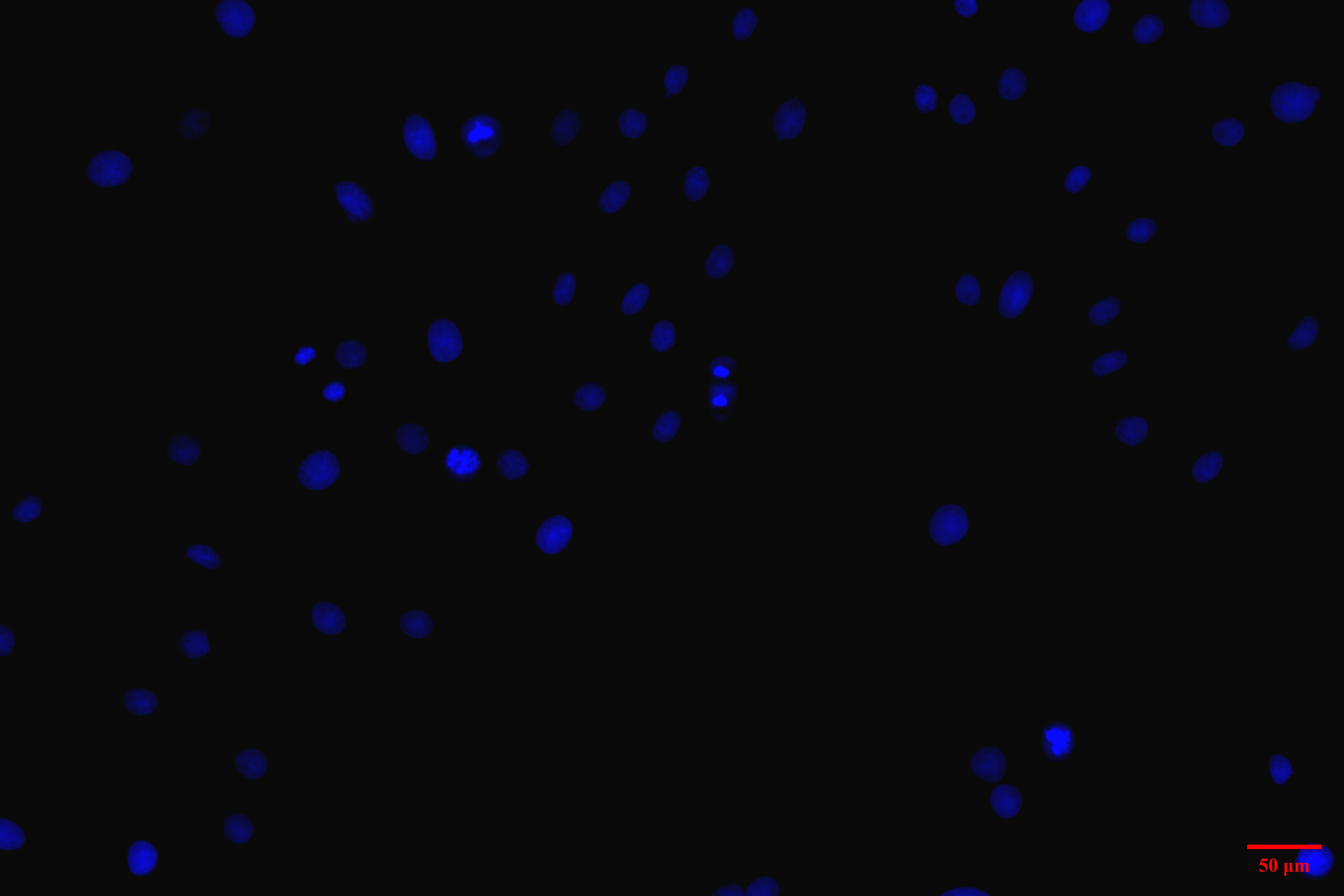

Supplement: Supplementary file 1 [file biomolecules-16-01059-s001.zip › File S1/Figure 6-8-11 Western blot original drawing/Figure 11e/control/DAPI-C-3.jpg]

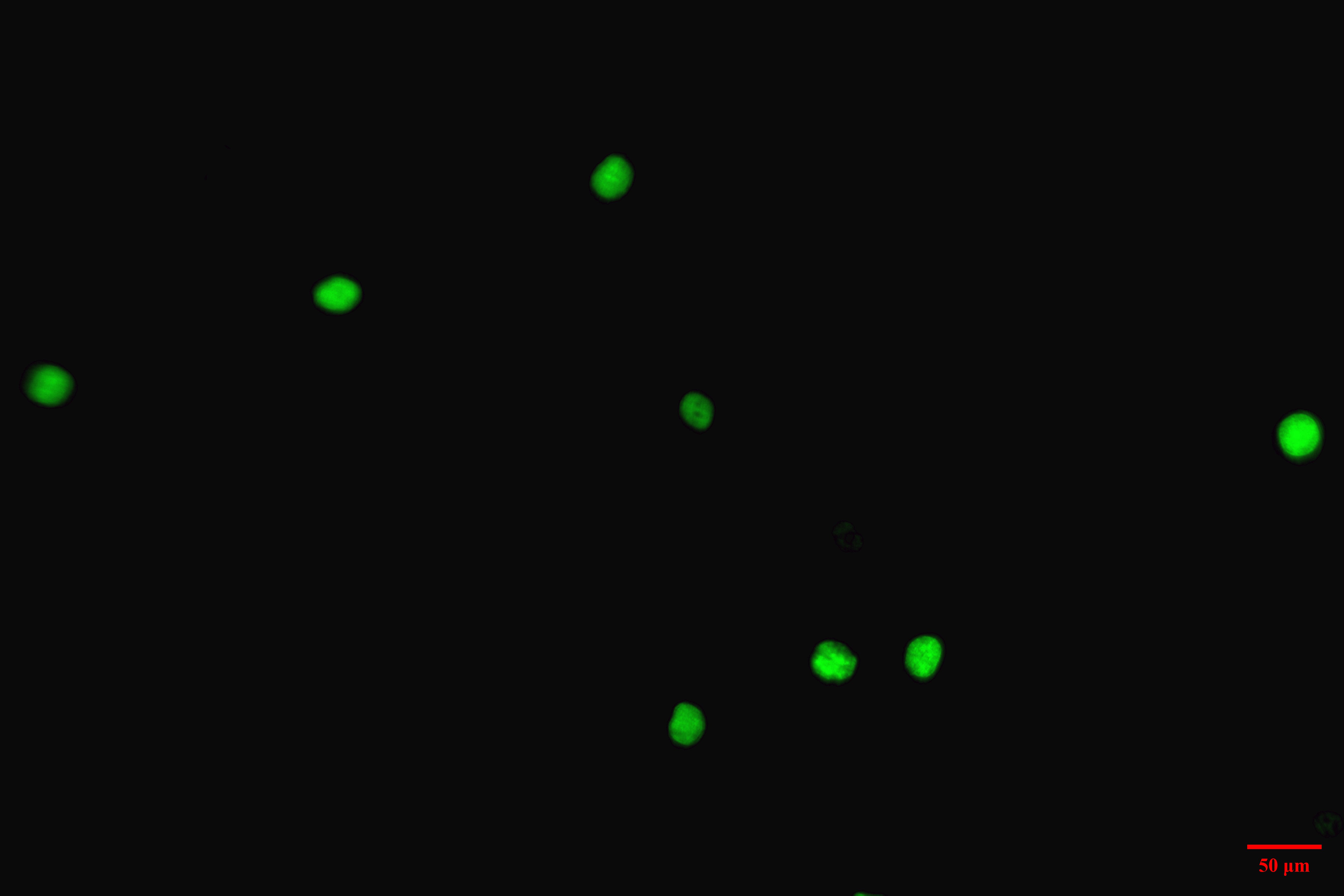

Supplement: Supplementary file 1 [file biomolecules-16-01059-s001.zip › File S1/Figure 6-8-11 Western blot original drawing/Figure 11e/control/EDU-C-1.jpg]

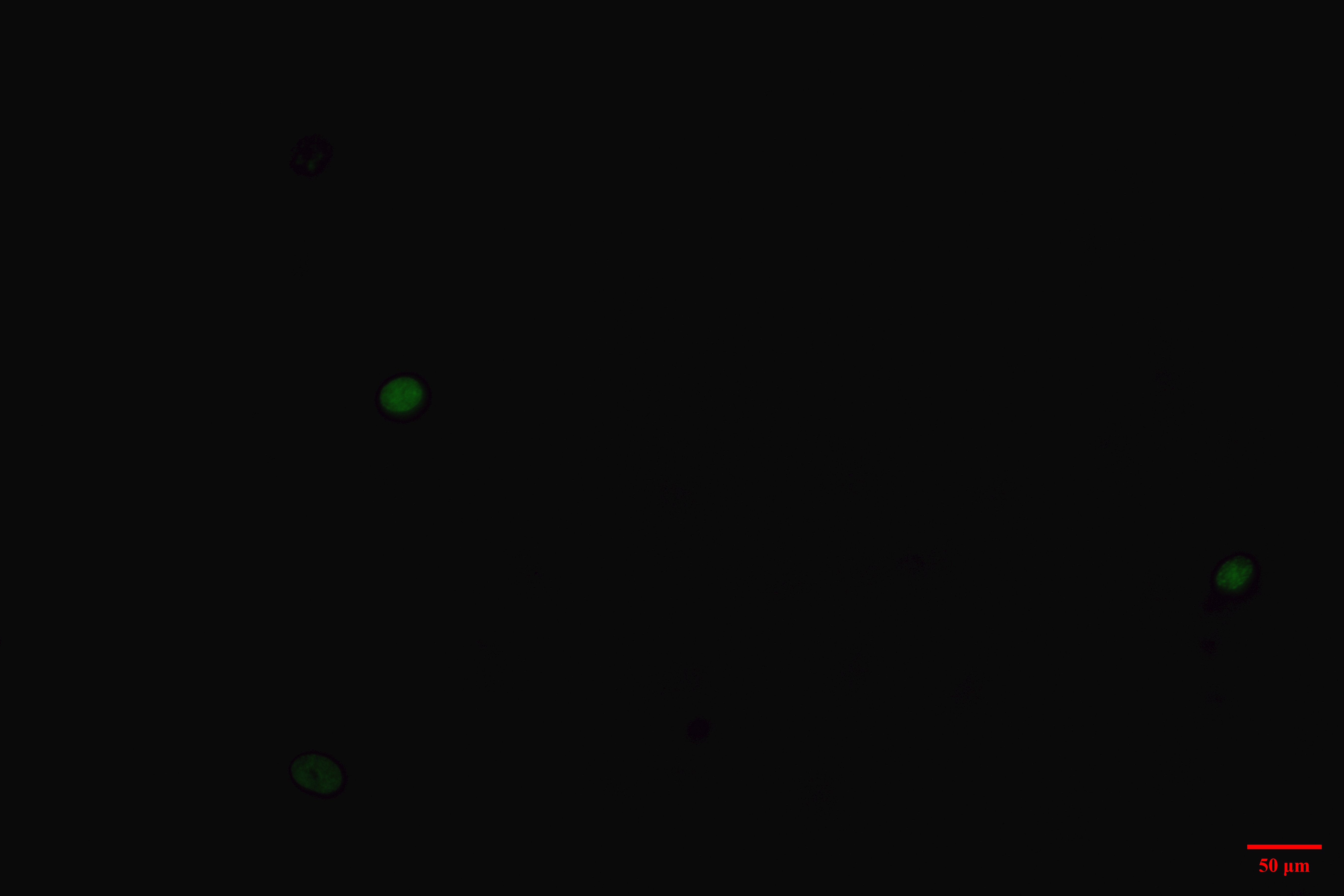

Supplement: Supplementary file 1 [file biomolecules-16-01059-s001.zip › File S1/Figure 6-8-11 Western blot original drawing/Figure 11e/control/EDU-C-2.jpg]

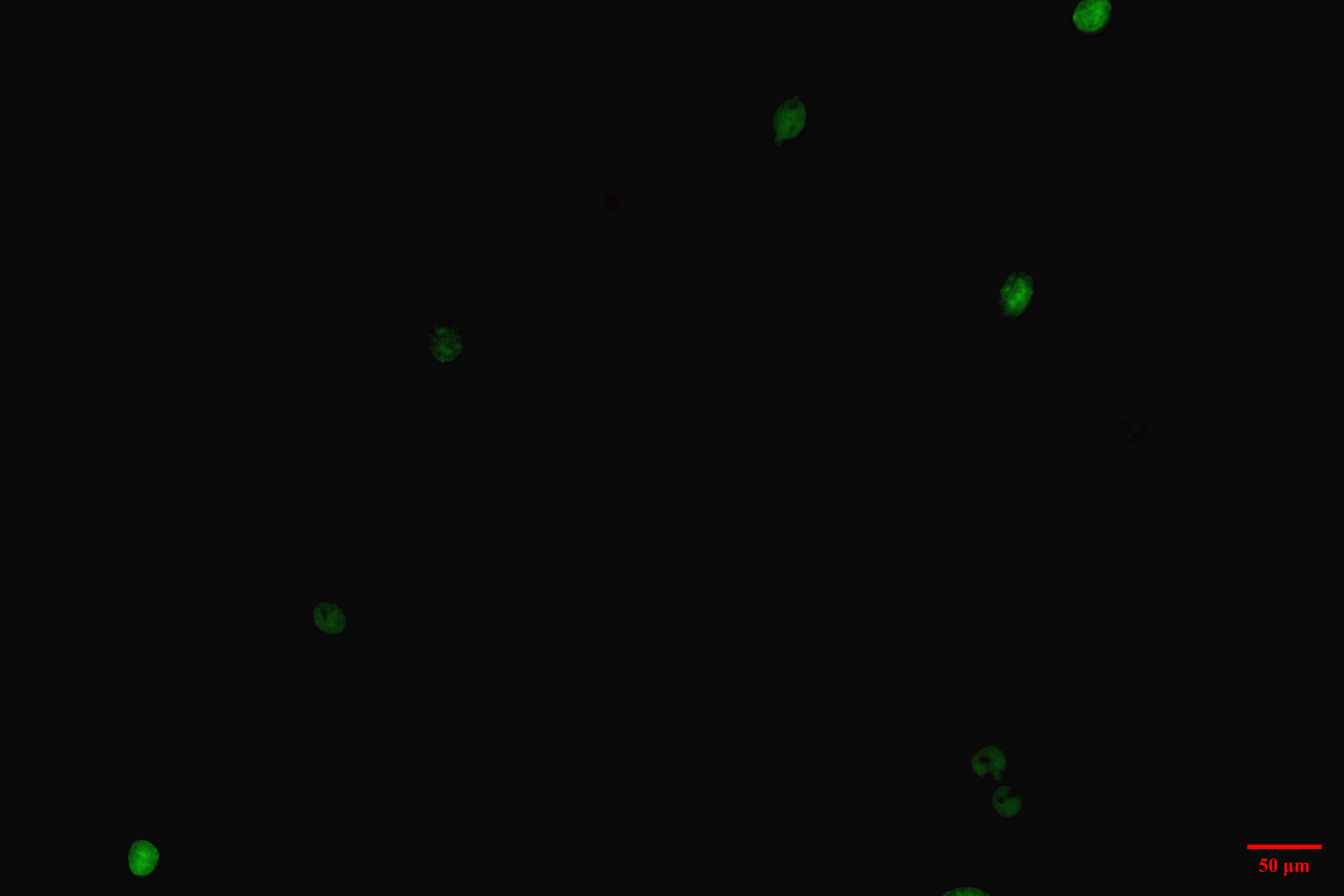

Supplement: Supplementary file 1 [file biomolecules-16-01059-s001.zip › File S1/Figure 6-8-11 Western blot original drawing/Figure 11e/control/EDU-C-3.jpg]

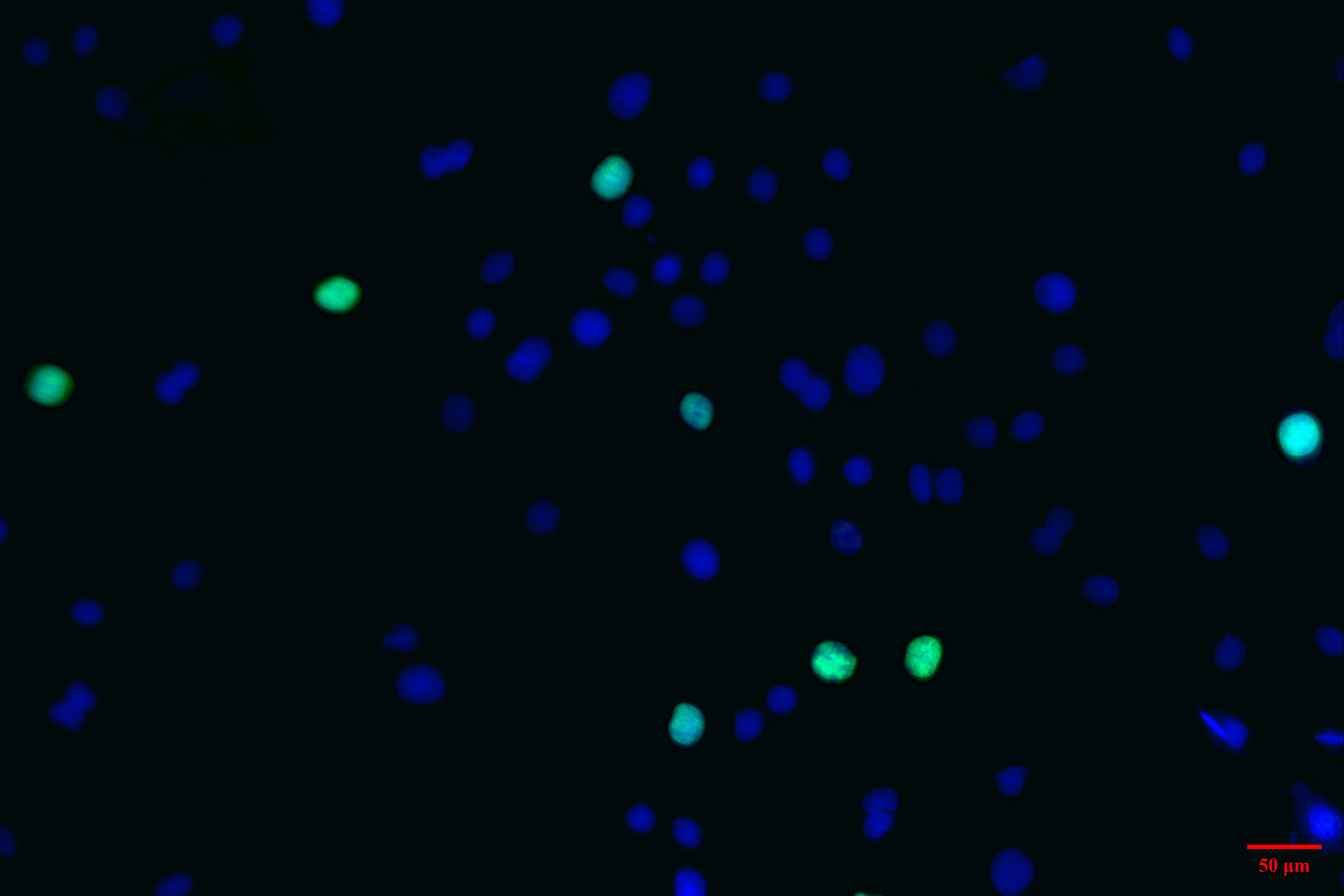

Supplement: Supplementary file 1 [file biomolecules-16-01059-s001.zip › File S1/Figure 6-8-11 Western blot original drawing/Figure 11e/control/Merge-C-1.jpg]

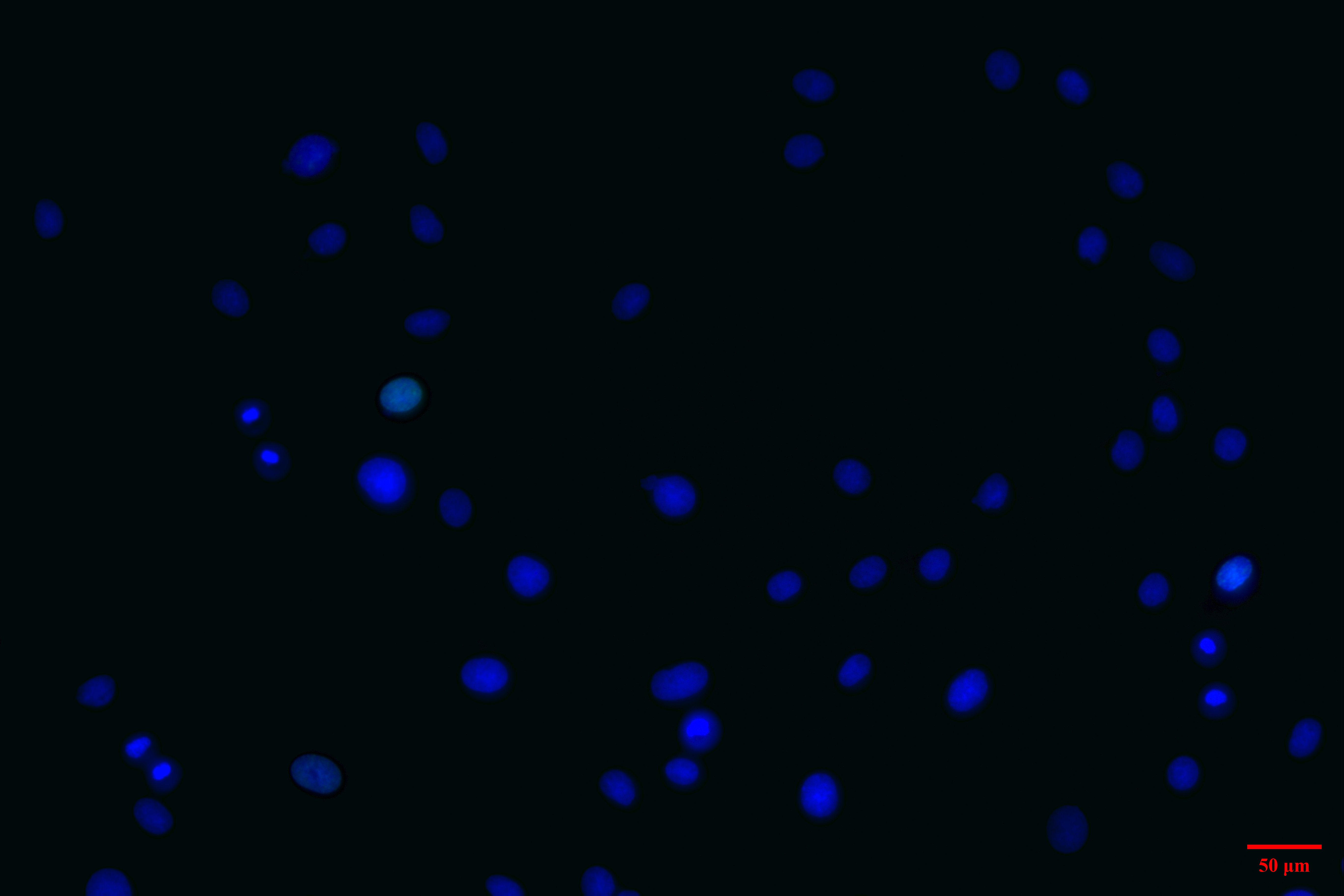

Supplement: Supplementary file 1 [file biomolecules-16-01059-s001.zip › File S1/Figure 6-8-11 Western blot original drawing/Figure 11e/control/Merge-C-2.jpg]

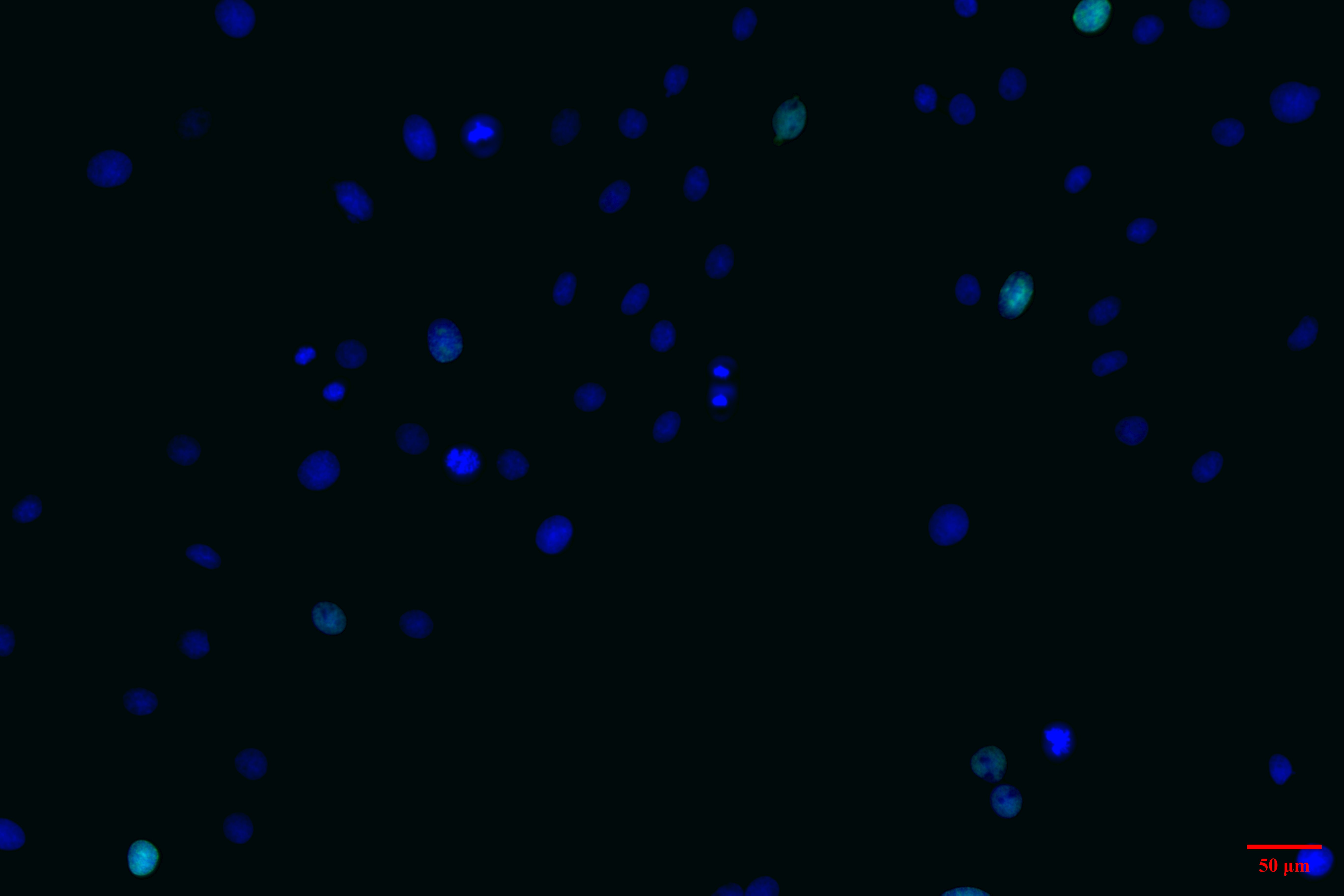

Supplement: Supplementary file 1 [file biomolecules-16-01059-s001.zip › File S1/Figure 6-8-11 Western blot original drawing/Figure 11e/control/Merge-C-3.jpg]

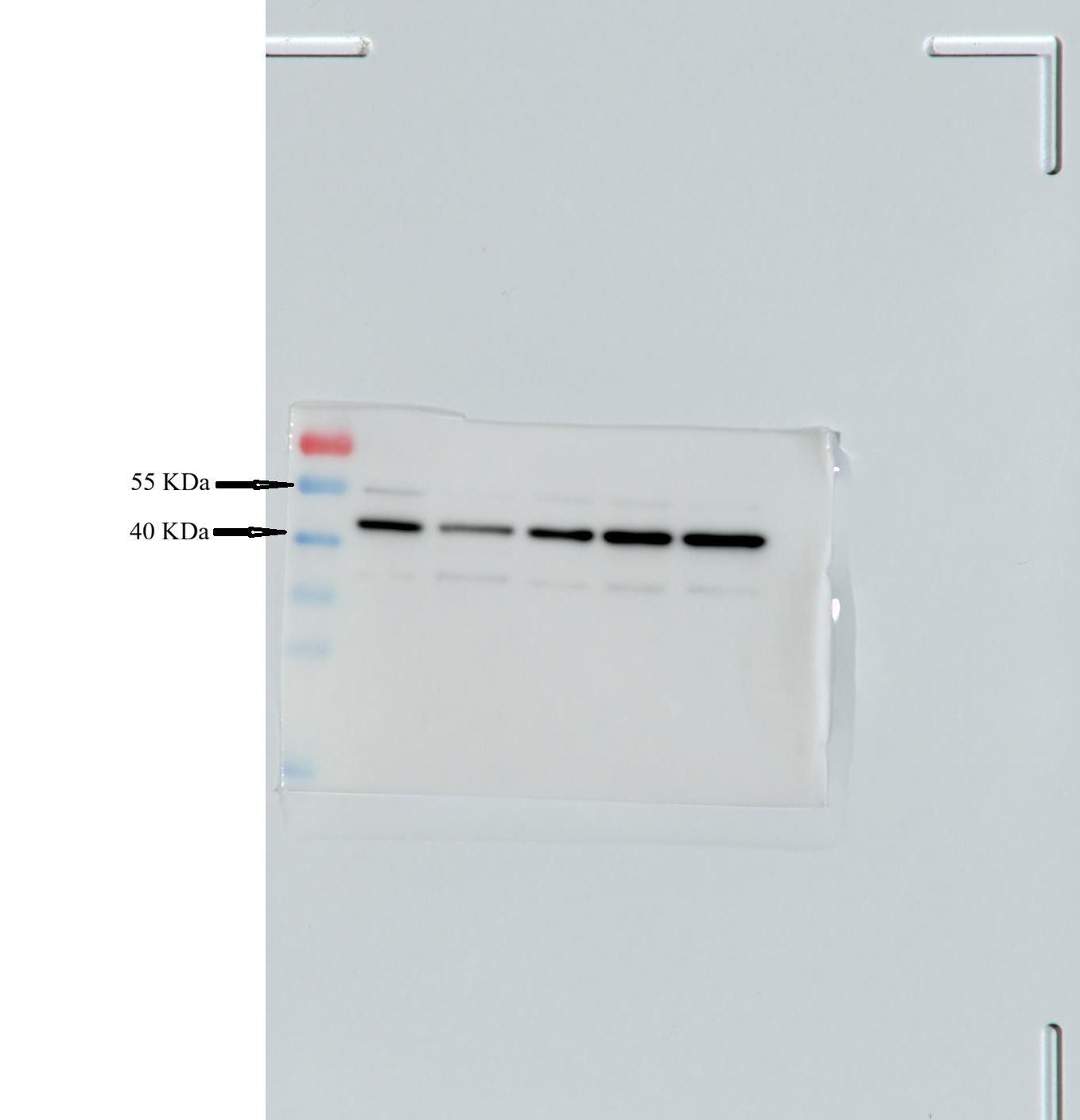

Supplement: Supplementary file 1 [file biomolecules-16-01059-s001.zip › File S1/Figure 6-8-11 Western blot original drawing/Figure 6 a α-SMA/α-SMA-1.jpg]

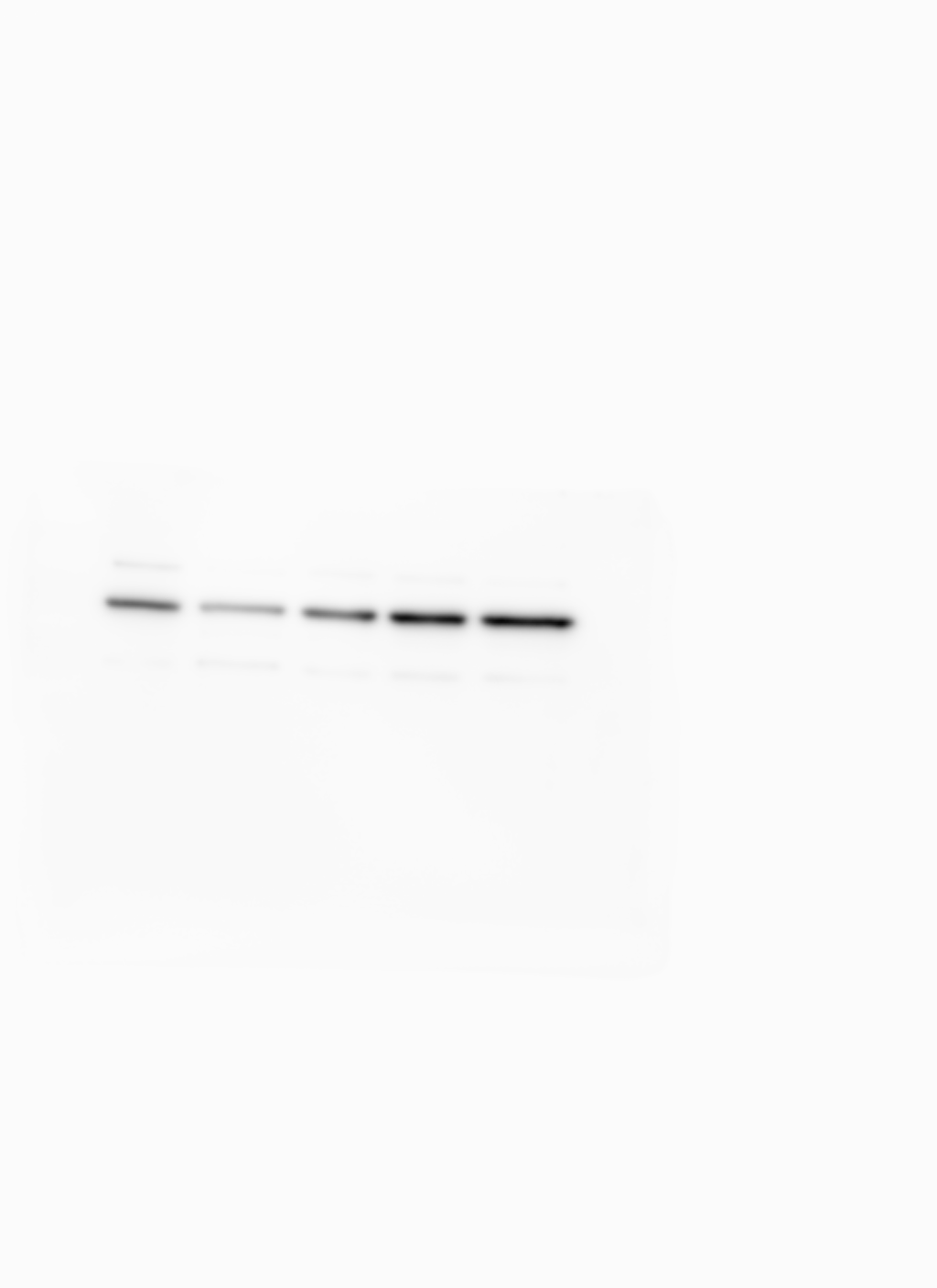

Supplement: Supplementary file 1 [file biomolecules-16-01059-s001.zip › File S1/Figure 6-8-11 Western blot original drawing/Figure 6 a α-SMA/α-SMA-1.tif]

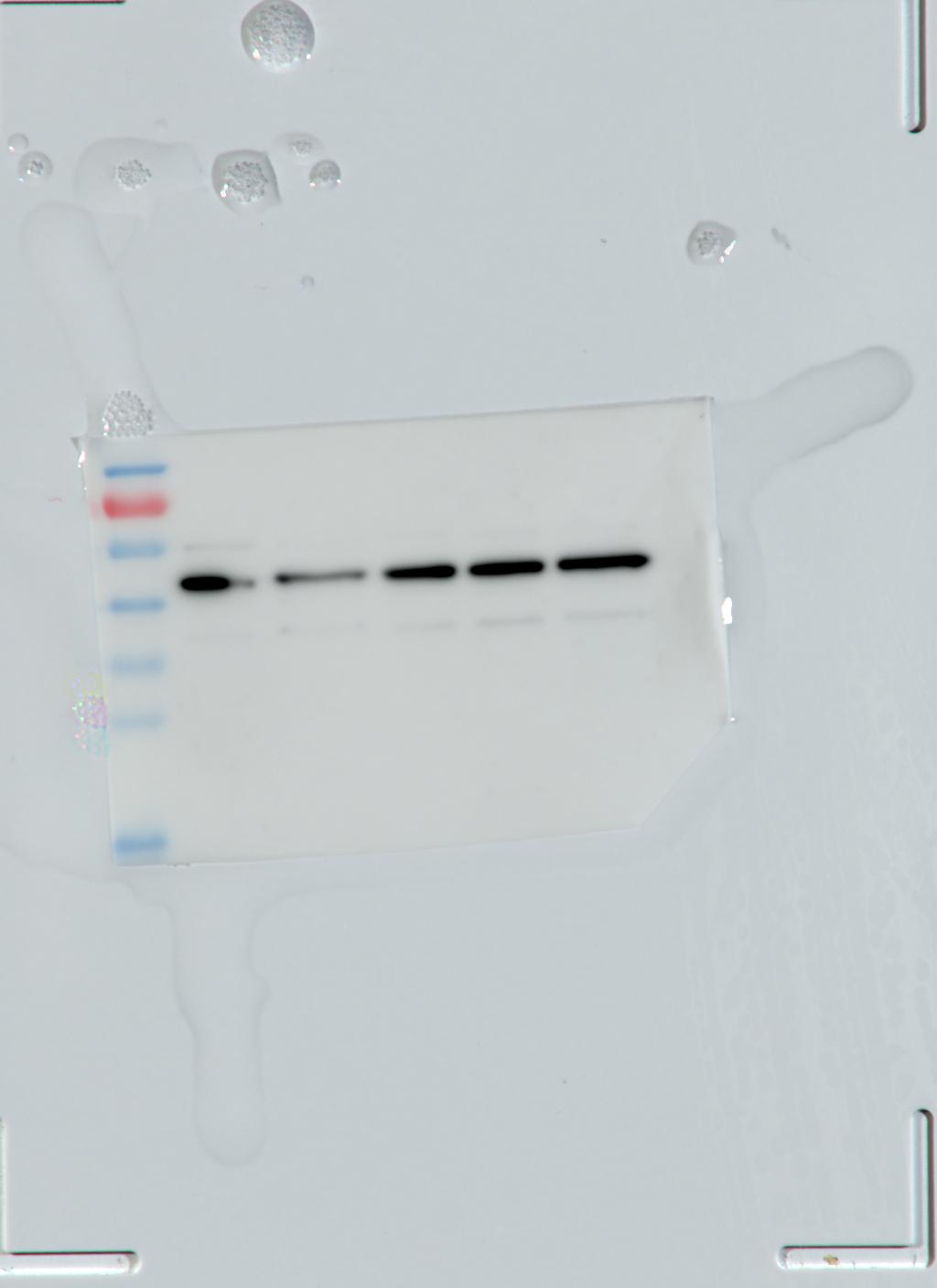

Supplement: Supplementary file 1 [file biomolecules-16-01059-s001.zip › File S1/Figure 6-8-11 Western blot original drawing/Figure 6 a α-SMA/α-SMA-2.jpg]

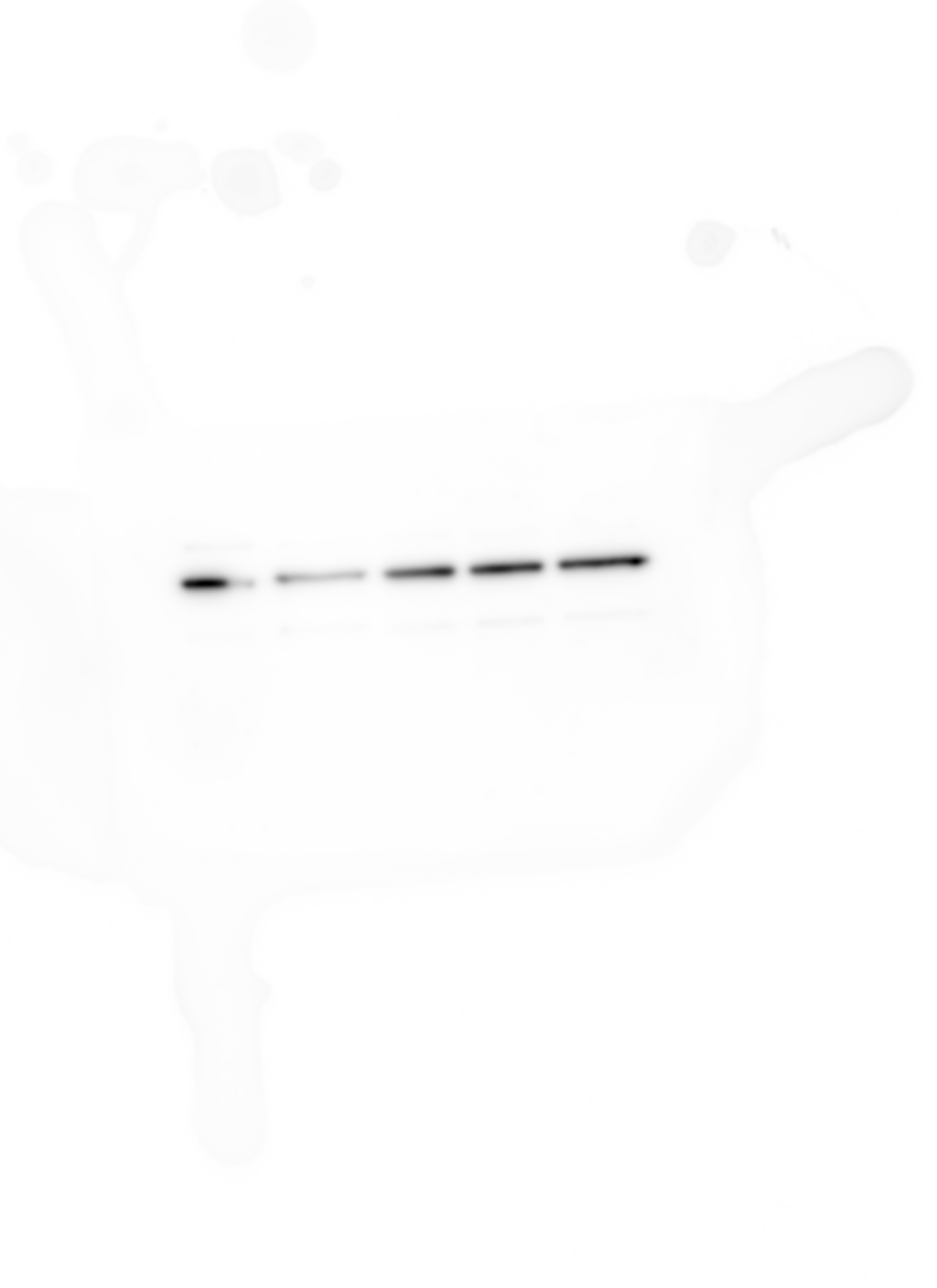

Supplement: Supplementary file 1 [file biomolecules-16-01059-s001.zip › File S1/Figure 6-8-11 Western blot original drawing/Figure 6 a α-SMA/α-SMA-2.tif]

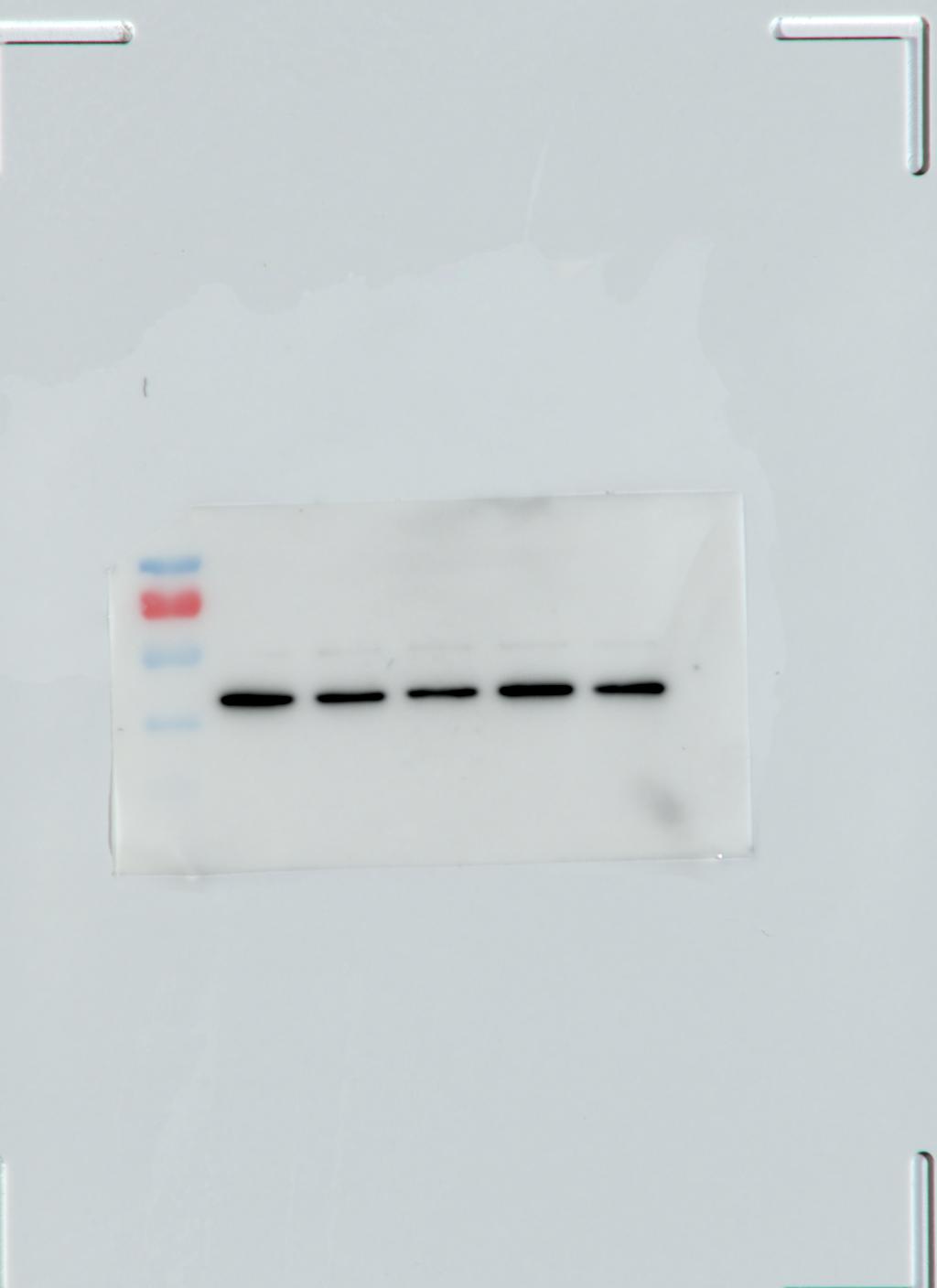

Supplement: Supplementary file 1 [file biomolecules-16-01059-s001.zip › File S1/Figure 6-8-11 Western blot original drawing/Figure 6 a α-SMA/α-SMA-3.jpg]

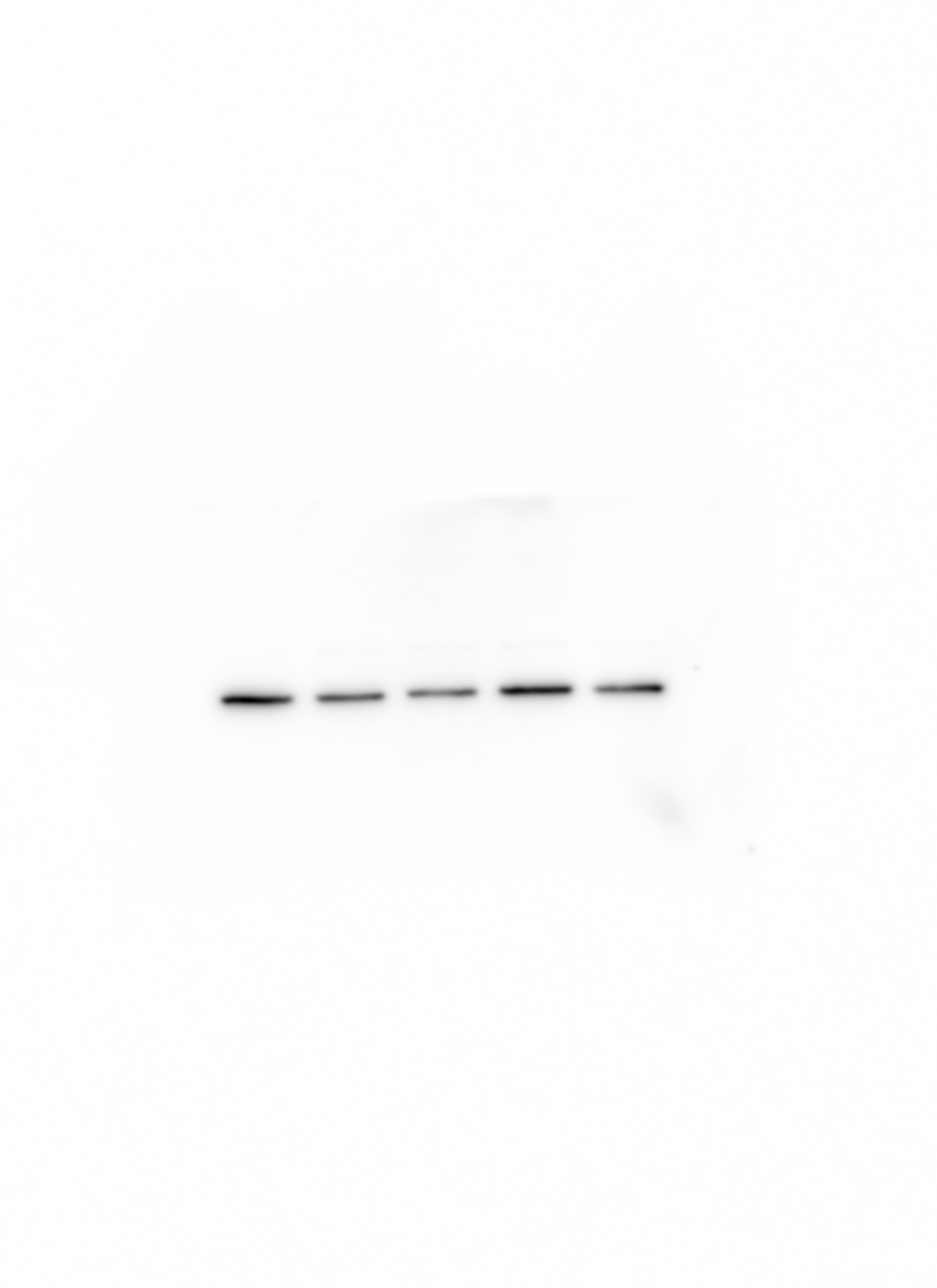

Supplement: Supplementary file 1 [file biomolecules-16-01059-s001.zip › File S1/Figure 6-8-11 Western blot original drawing/Figure 6 a α-SMA/α-SMA-3.tif]

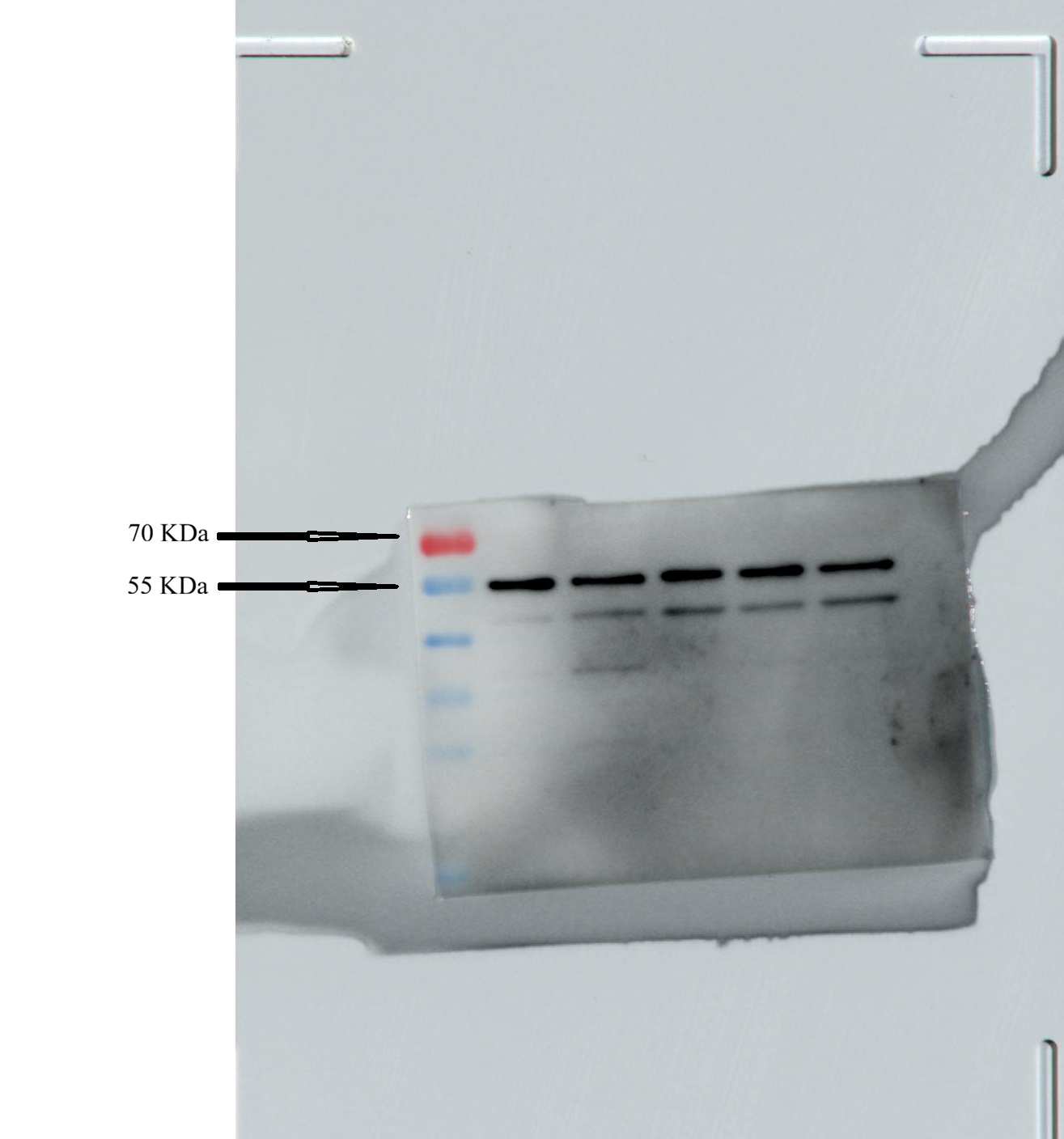

Supplement: Supplementary file 1 [file biomolecules-16-01059-s001.zip › File S1/Figure 6-8-11 Western blot original drawing/Figure 6 a α-SMA/α-tubulin-1.jpg]

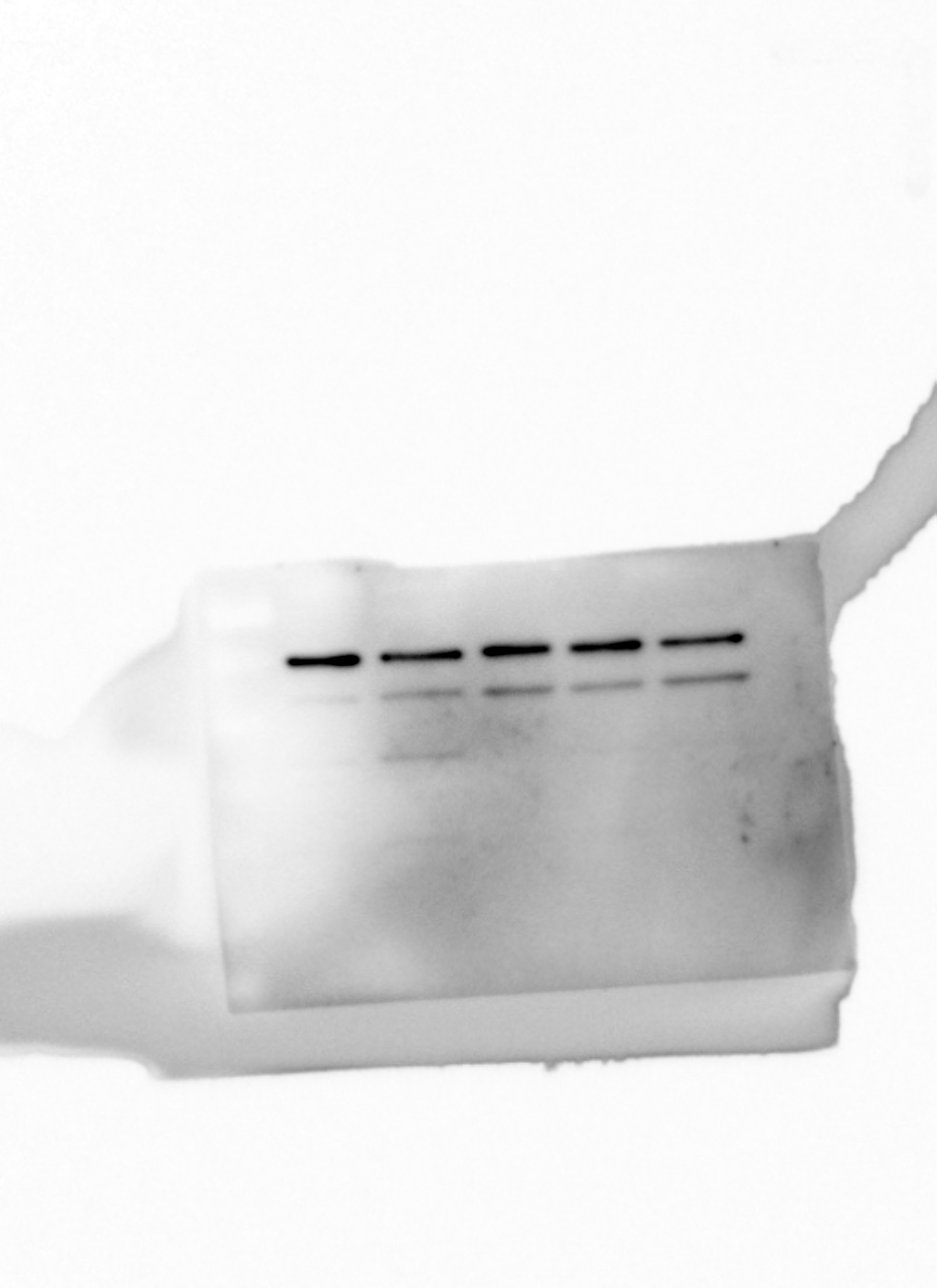

Supplement: Supplementary file 1 [file biomolecules-16-01059-s001.zip › File S1/Figure 6-8-11 Western blot original drawing/Figure 6 a α-SMA/α-tubulin-1.tif]

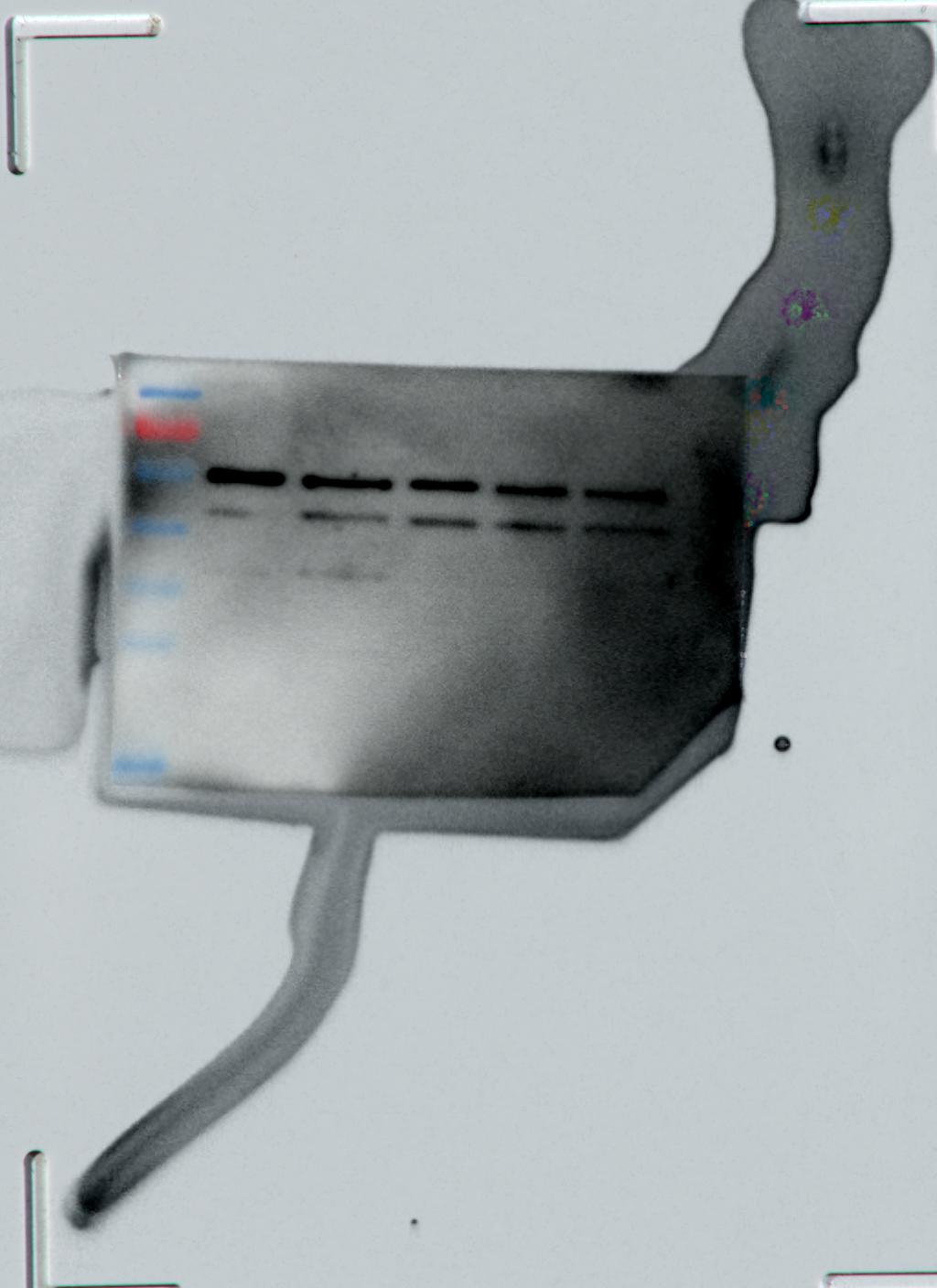

Supplement: Supplementary file 1 [file biomolecules-16-01059-s001.zip › File S1/Figure 6-8-11 Western blot original drawing/Figure 6 a α-SMA/α-tubulin-2.jpg]

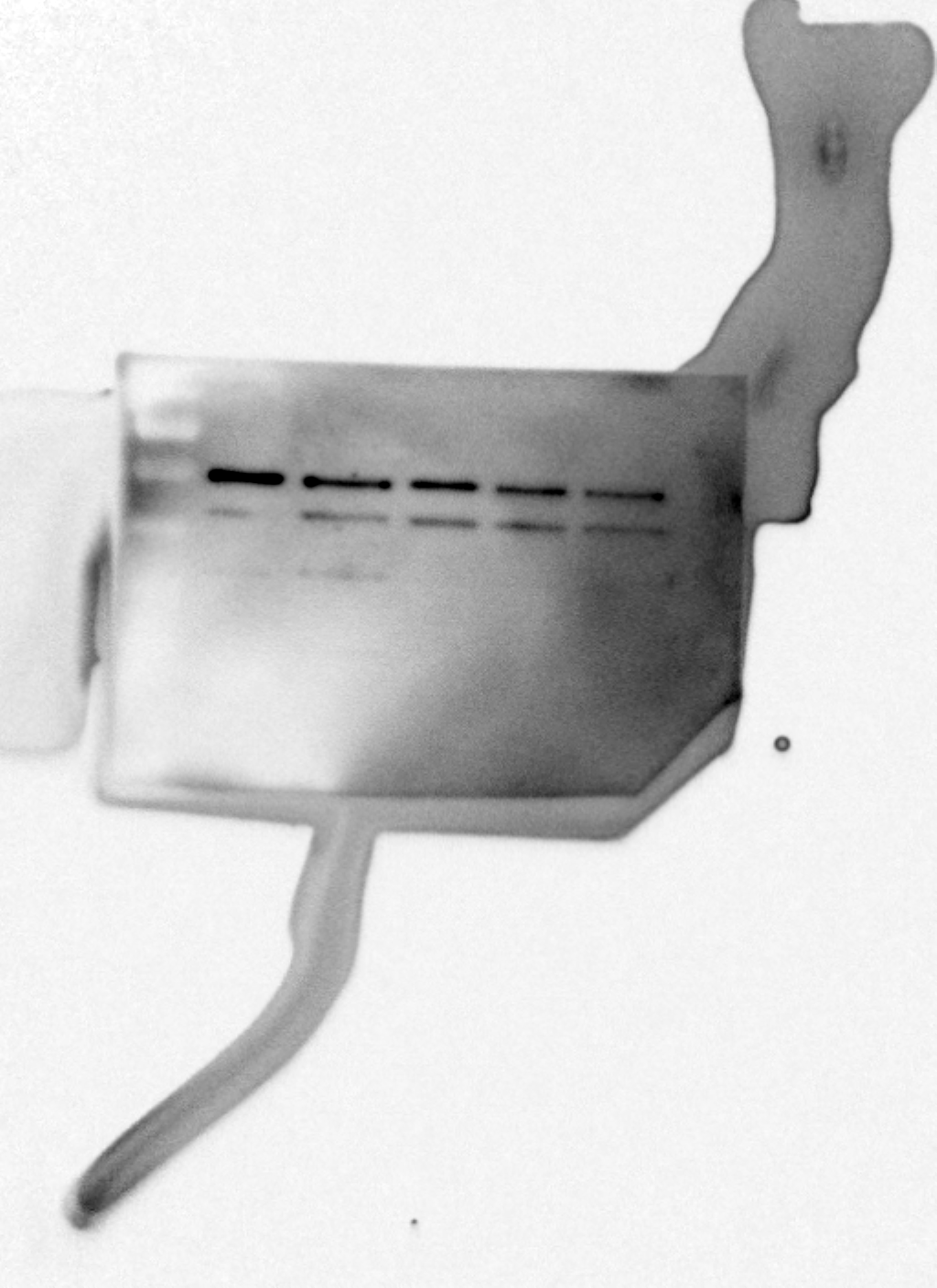

Supplement: Supplementary file 1 [file biomolecules-16-01059-s001.zip › File S1/Figure 6-8-11 Western blot original drawing/Figure 6 a α-SMA/α-tubulin-2.tif]

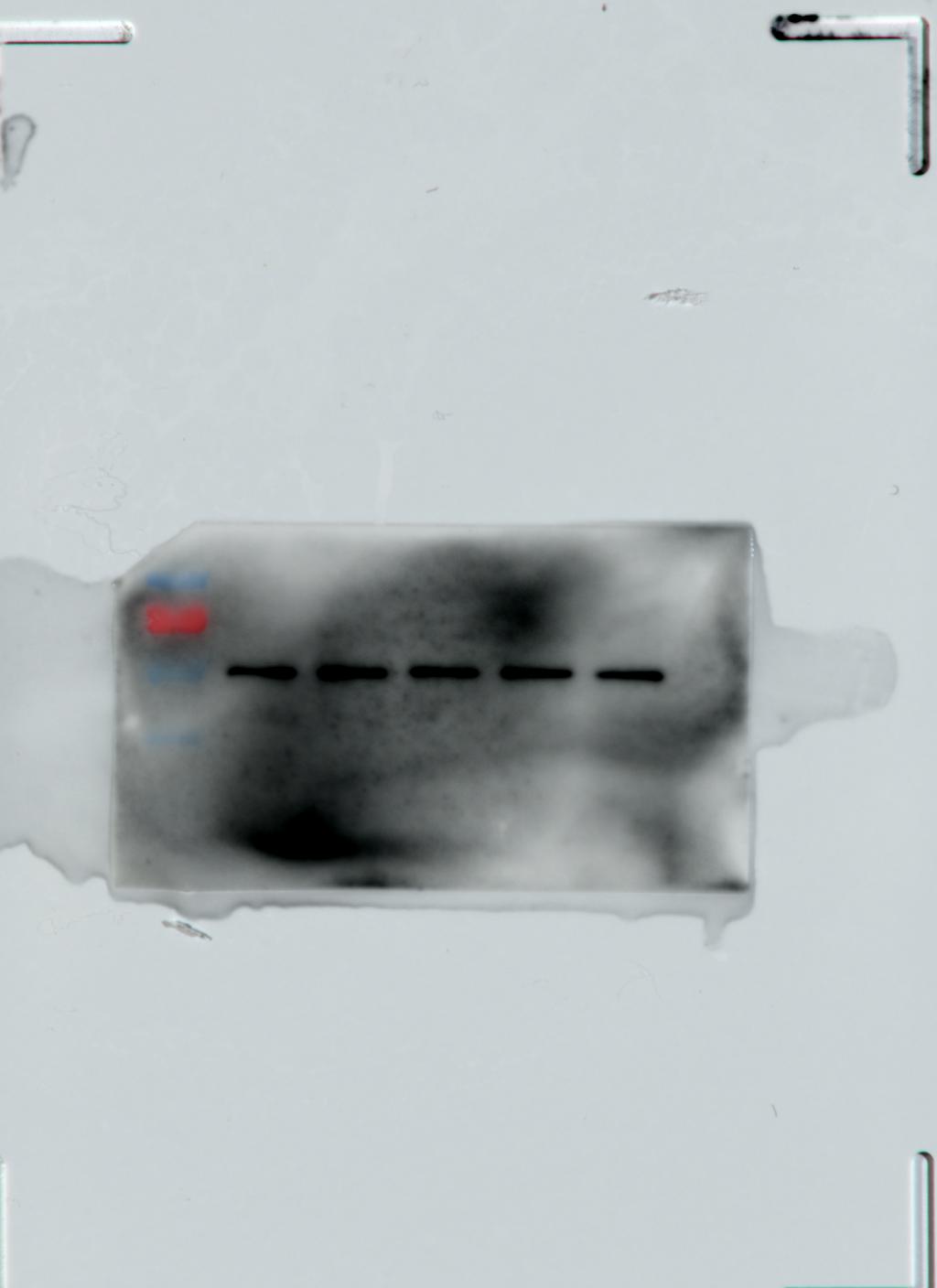

Supplement: Supplementary file 1 [file biomolecules-16-01059-s001.zip › File S1/Figure 6-8-11 Western blot original drawing/Figure 6 a α-SMA/α-tubulin-3.jpg]

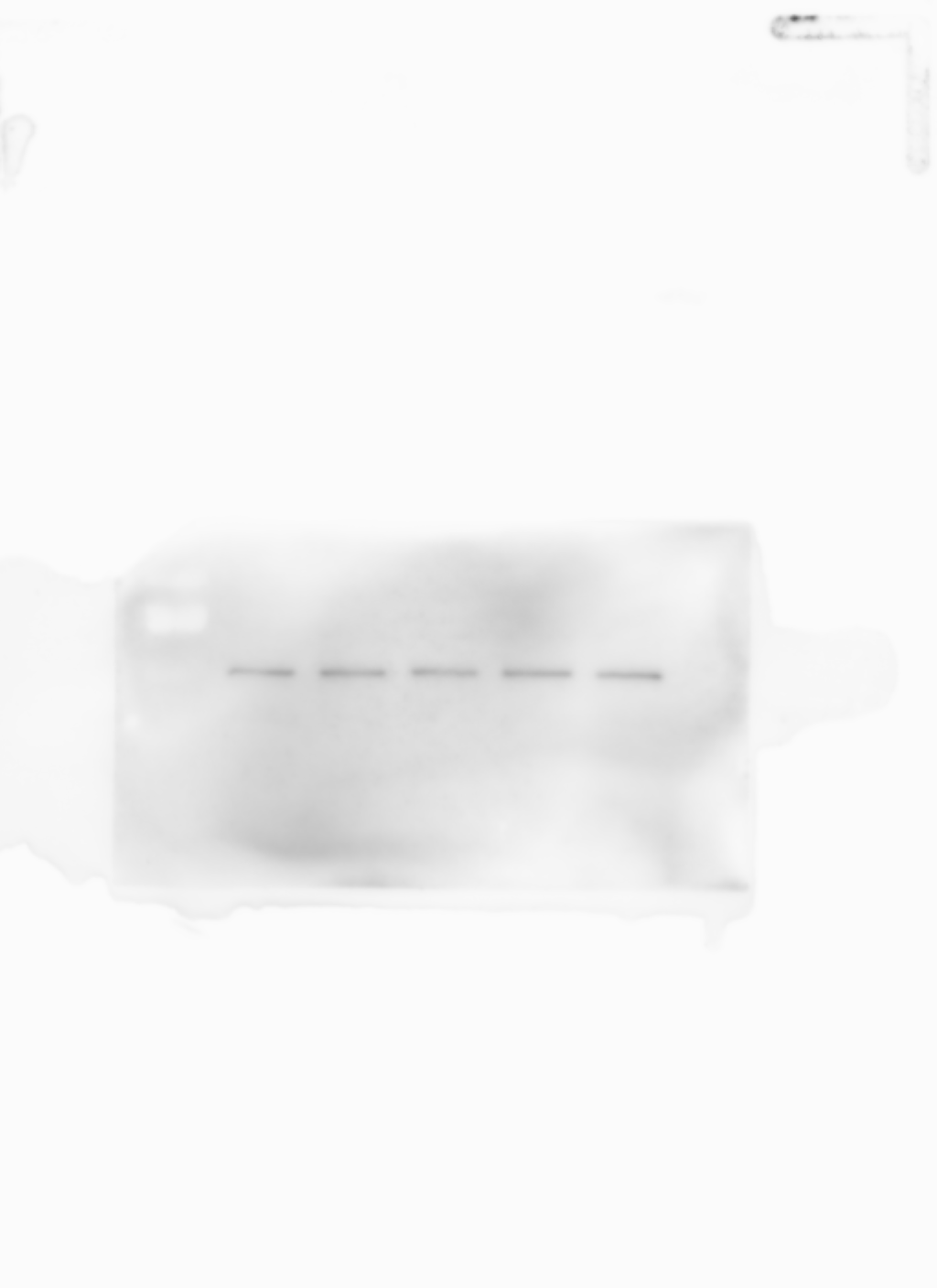

Supplement: Supplementary file 1 [file biomolecules-16-01059-s001.zip › File S1/Figure 6-8-11 Western blot original drawing/Figure 6 a α-SMA/α-tubulin-3.tif]

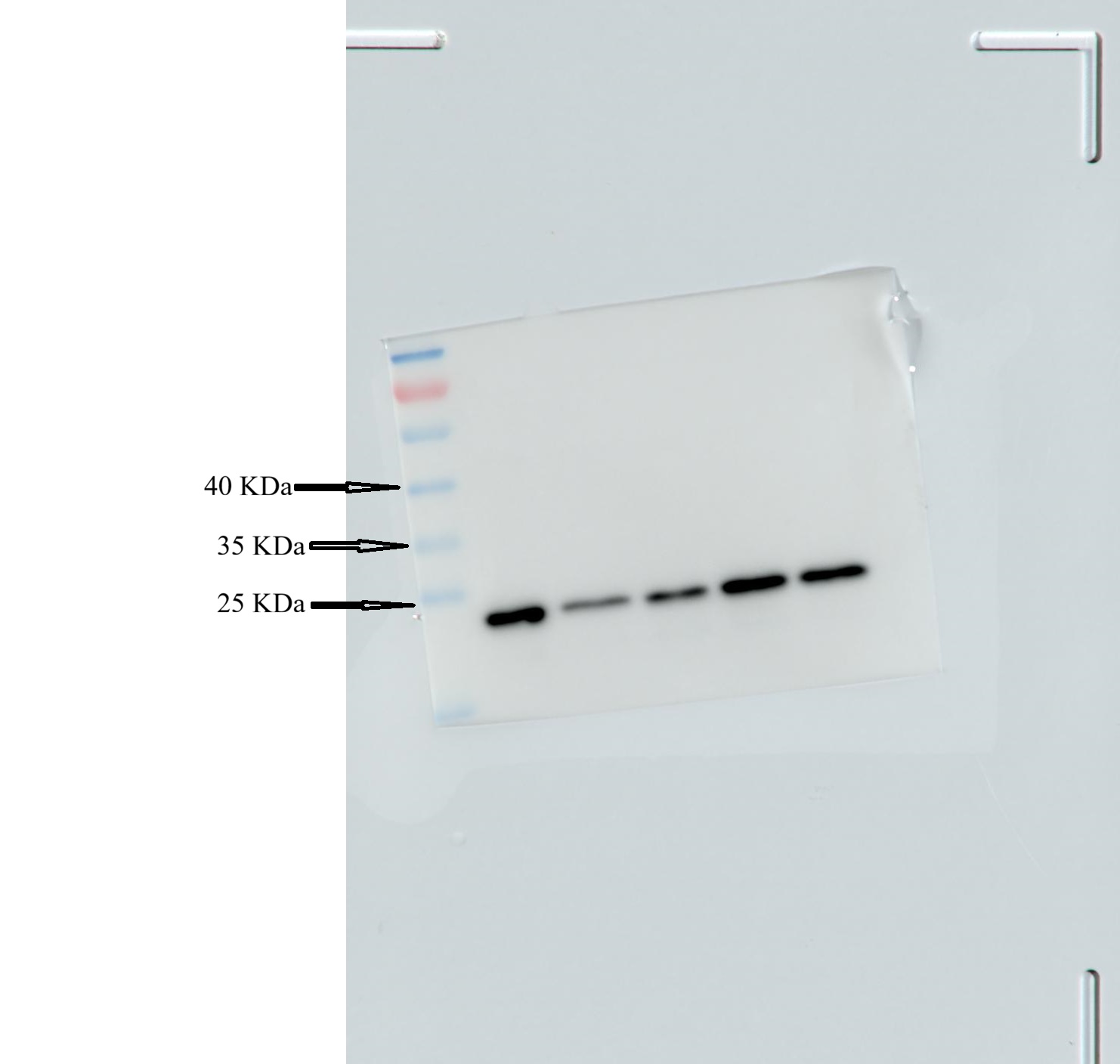

Supplement: Supplementary file 1 [file biomolecules-16-01059-s001.zip › File S1/Figure 6-8-11 Western blot original drawing/Figure 6 b SM22α/SM22α-1.jpg]

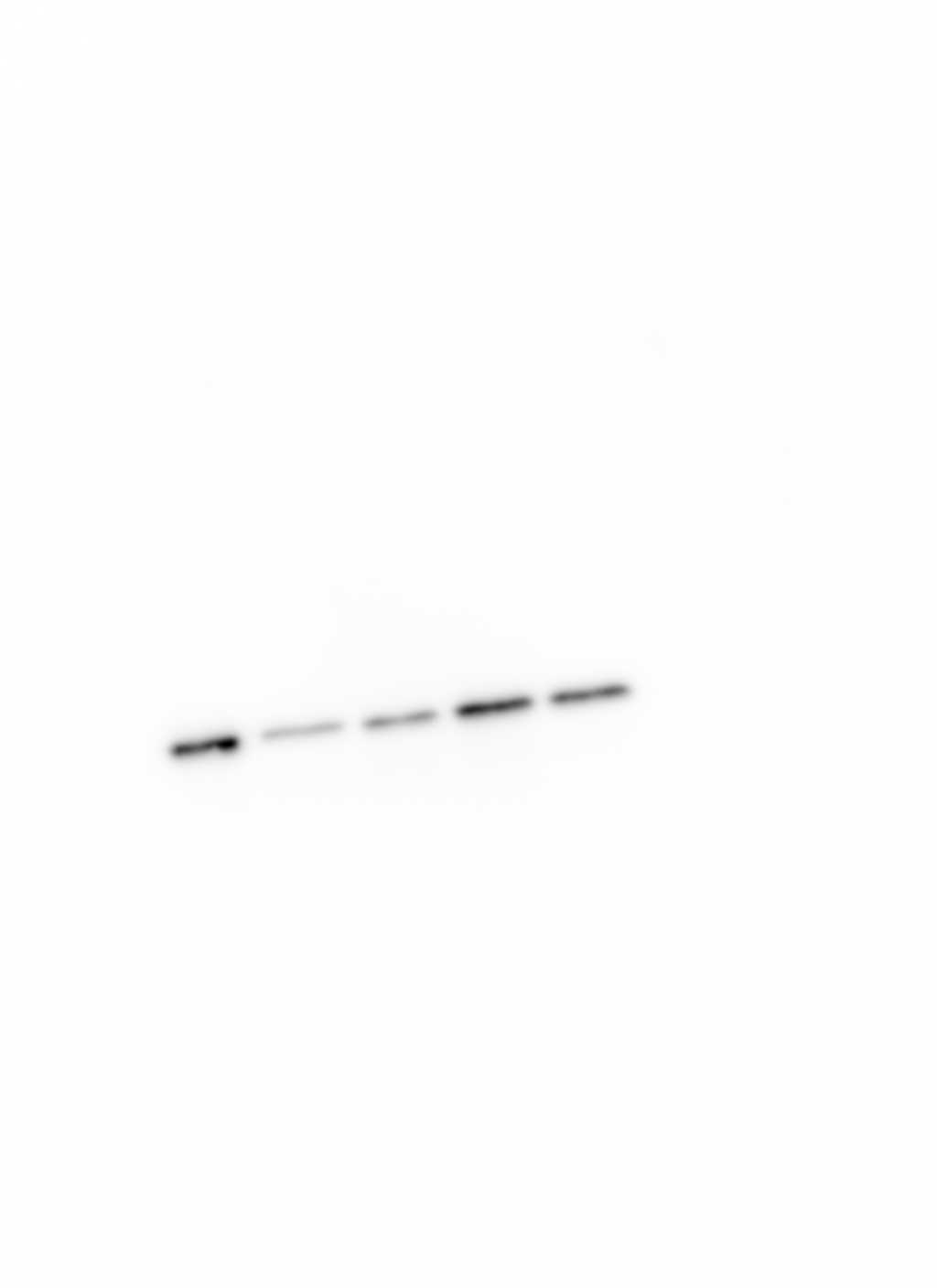

Supplement: Supplementary file 1 [file biomolecules-16-01059-s001.zip › File S1/Figure 6-8-11 Western blot original drawing/Figure 6 b SM22α/SM22α-1.tif]

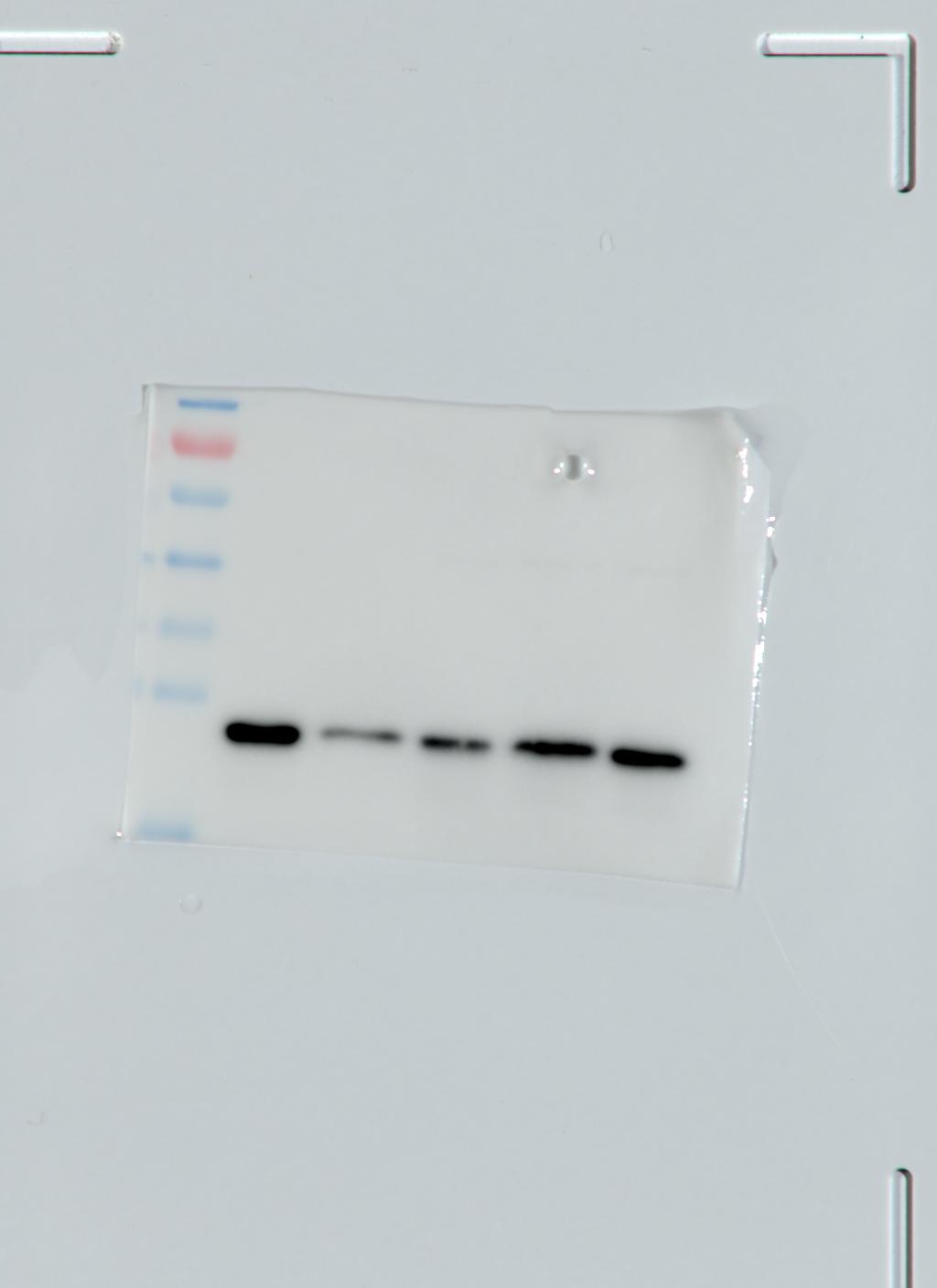

Supplement: Supplementary file 1 [file biomolecules-16-01059-s001.zip › File S1/Figure 6-8-11 Western blot original drawing/Figure 6 b SM22α/SM22α-2.jpg]

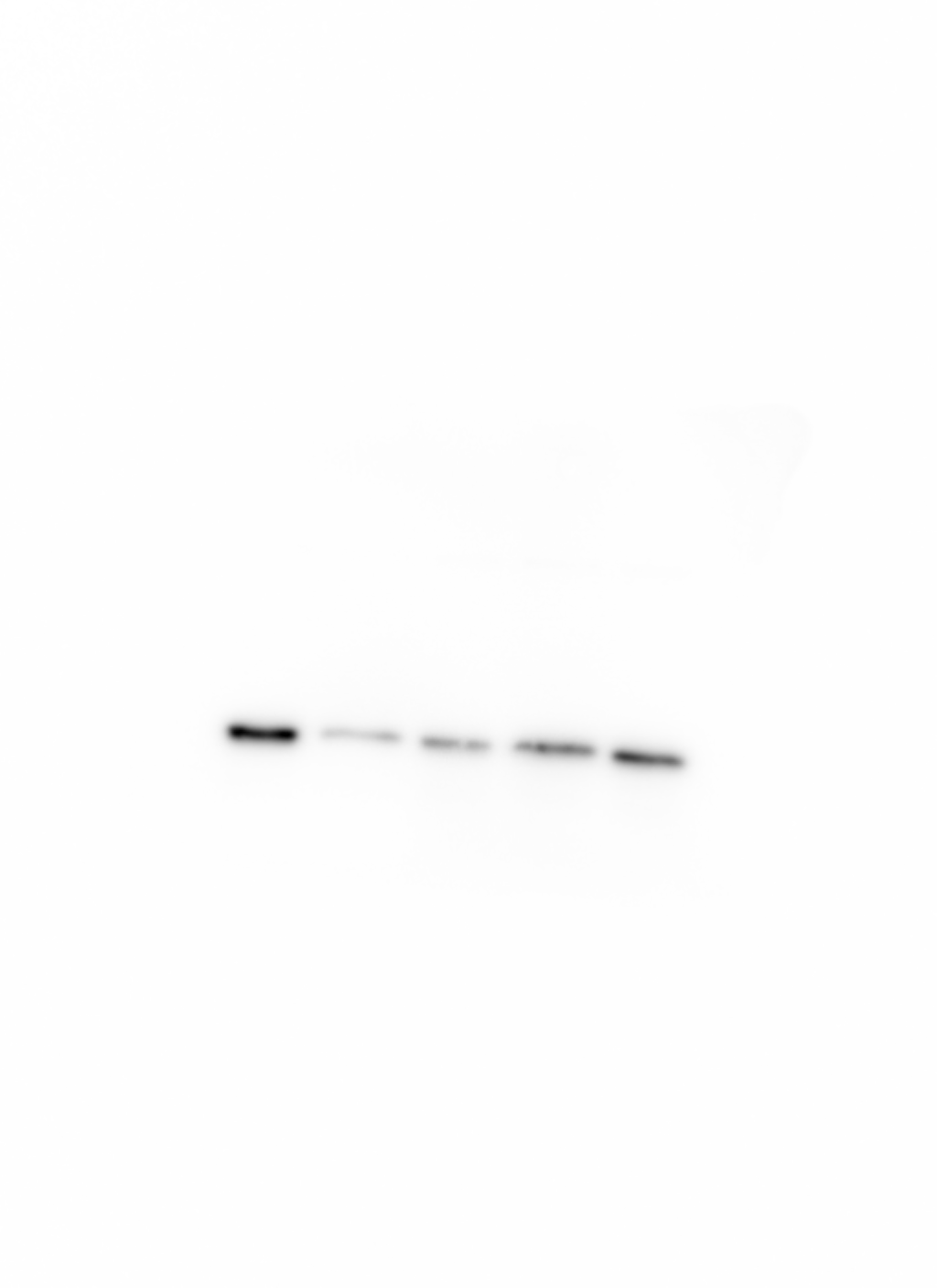

Supplement: Supplementary file 1 [file biomolecules-16-01059-s001.zip › File S1/Figure 6-8-11 Western blot original drawing/Figure 6 b SM22α/SM22α-2.tif]

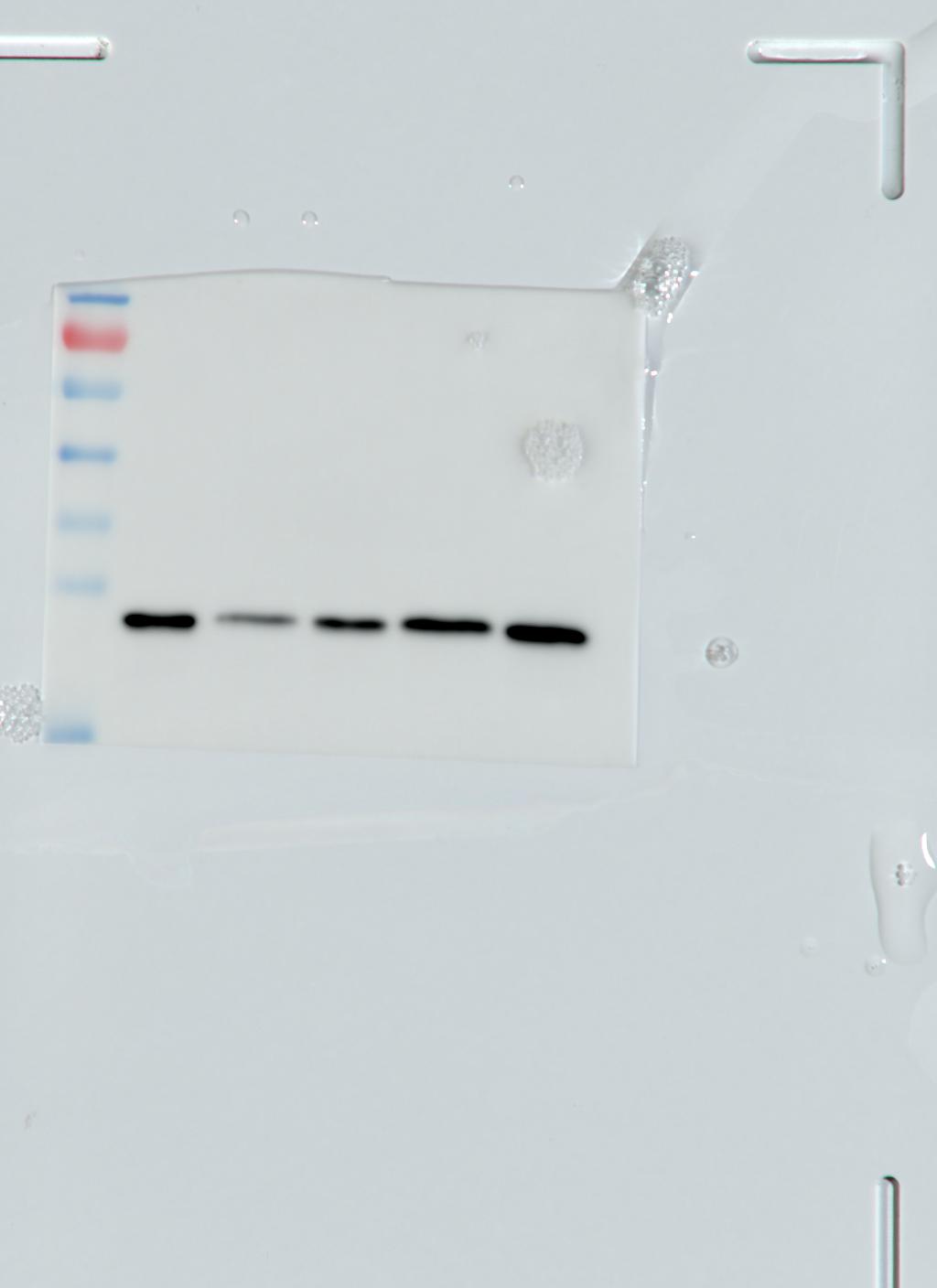

Supplement: Supplementary file 1 [file biomolecules-16-01059-s001.zip › File S1/Figure 6-8-11 Western blot original drawing/Figure 6 b SM22α/SM22α-3.jpg]

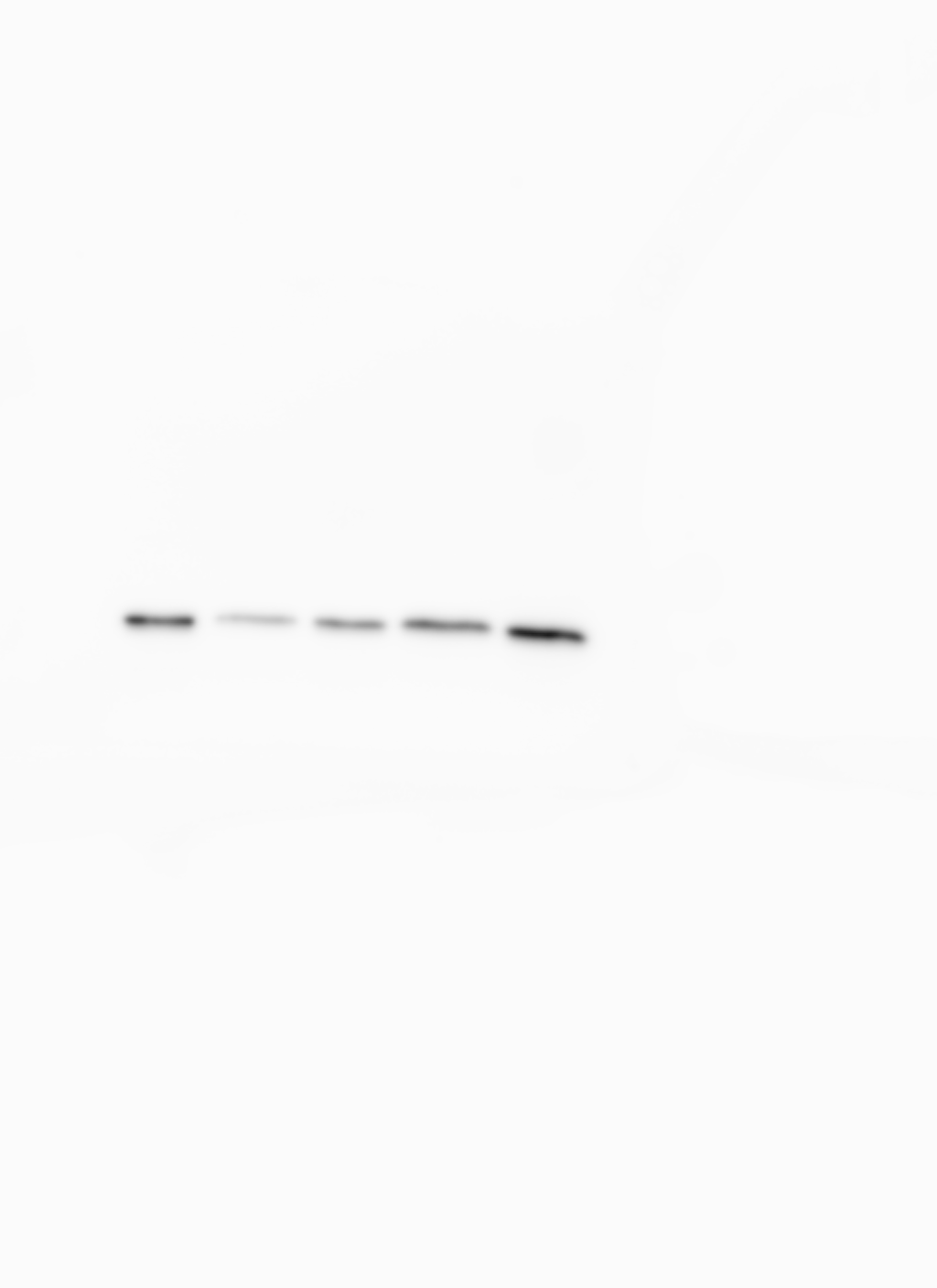

Supplement: Supplementary file 1 [file biomolecules-16-01059-s001.zip › File S1/Figure 6-8-11 Western blot original drawing/Figure 6 b SM22α/SM22α-3.tif]

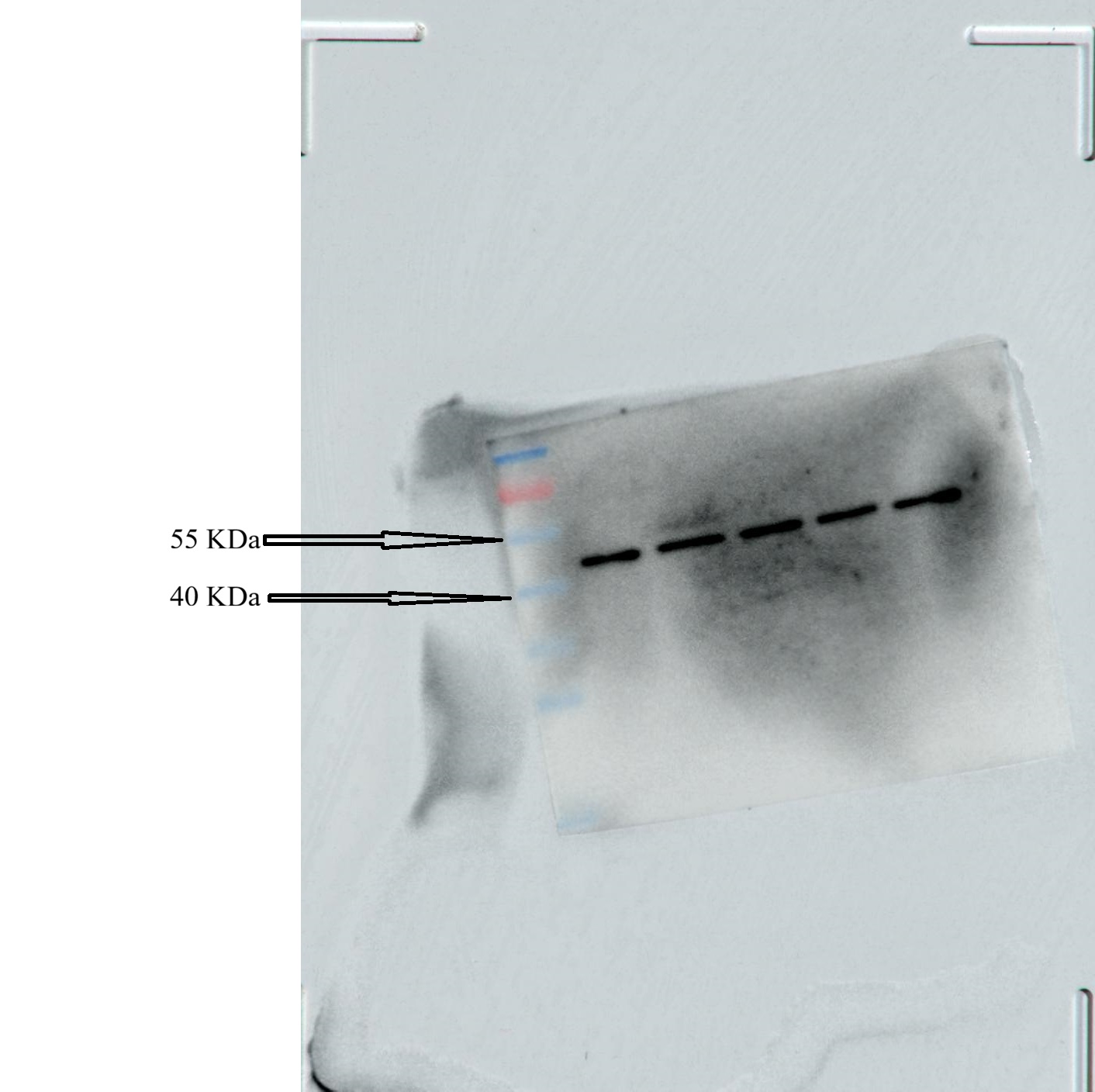

Supplement: Supplementary file 1 [file biomolecules-16-01059-s001.zip › File S1/Figure 6-8-11 Western blot original drawing/Figure 6 b SM22α/β-actin-1.jpg]

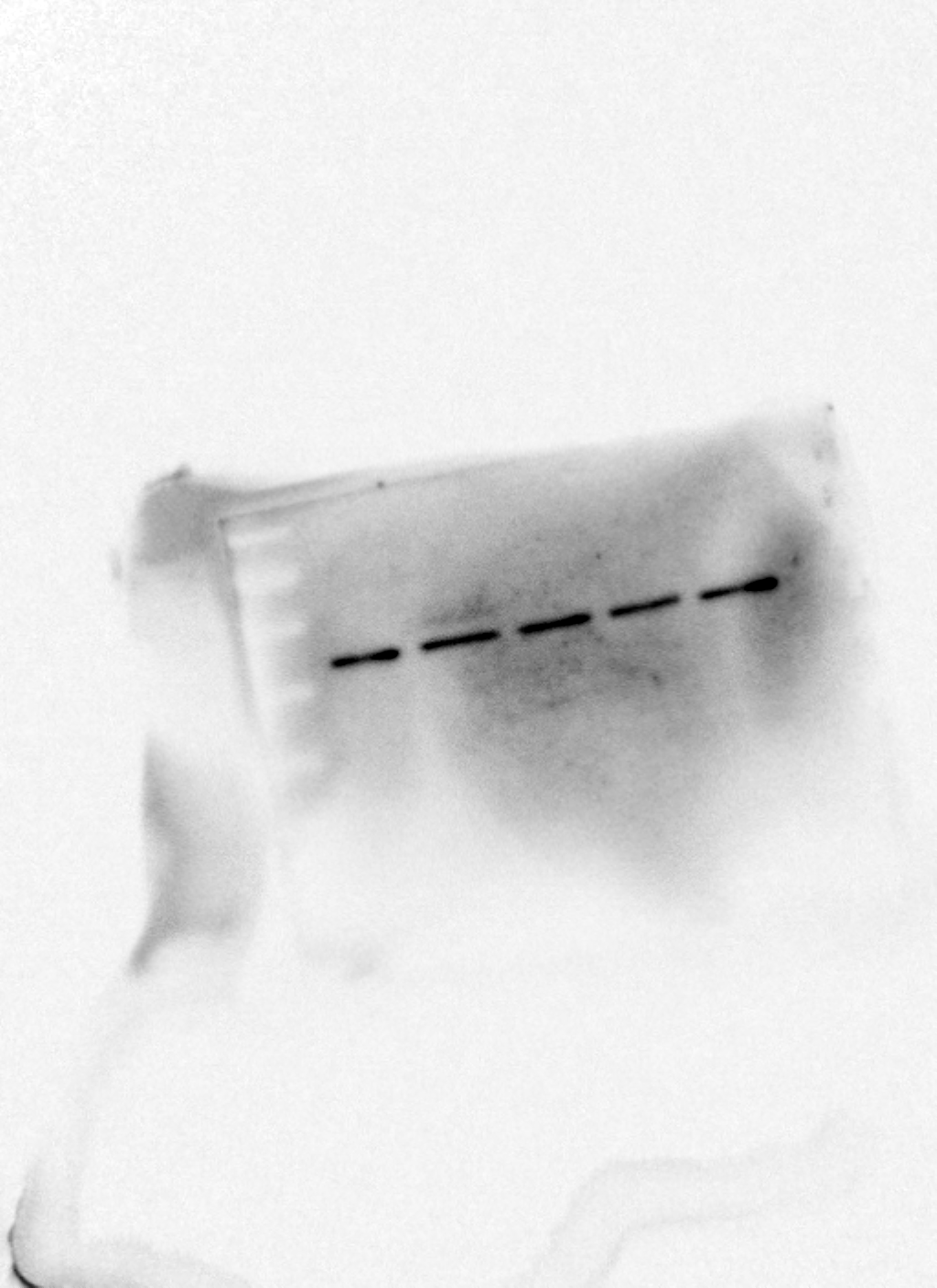

Supplement: Supplementary file 1 [file biomolecules-16-01059-s001.zip › File S1/Figure 6-8-11 Western blot original drawing/Figure 6 b SM22α/β-actin-1.tif]

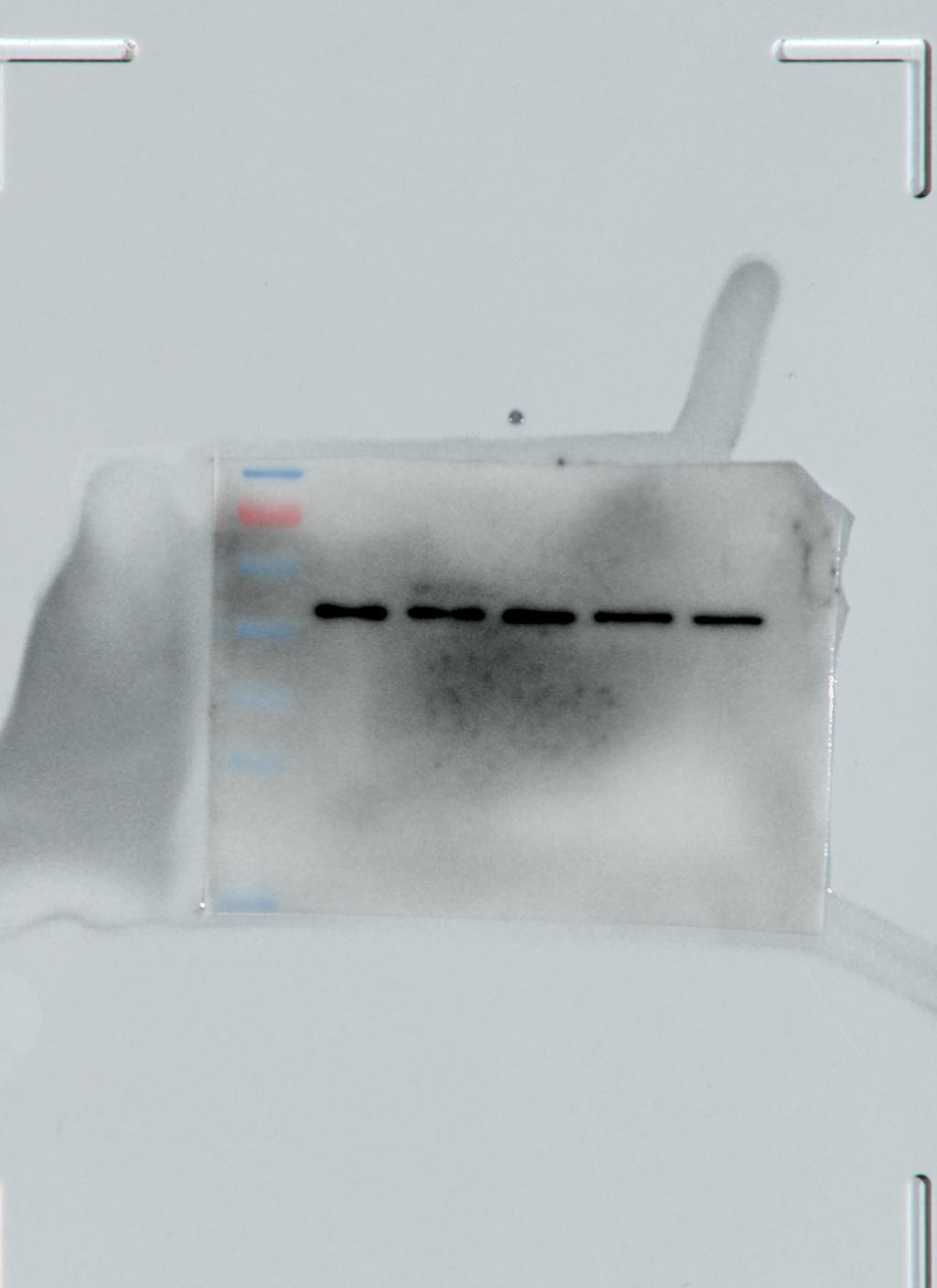

Supplement: Supplementary file 1 [file biomolecules-16-01059-s001.zip › File S1/Figure 6-8-11 Western blot original drawing/Figure 6 b SM22α/β-actin-2.jpg]

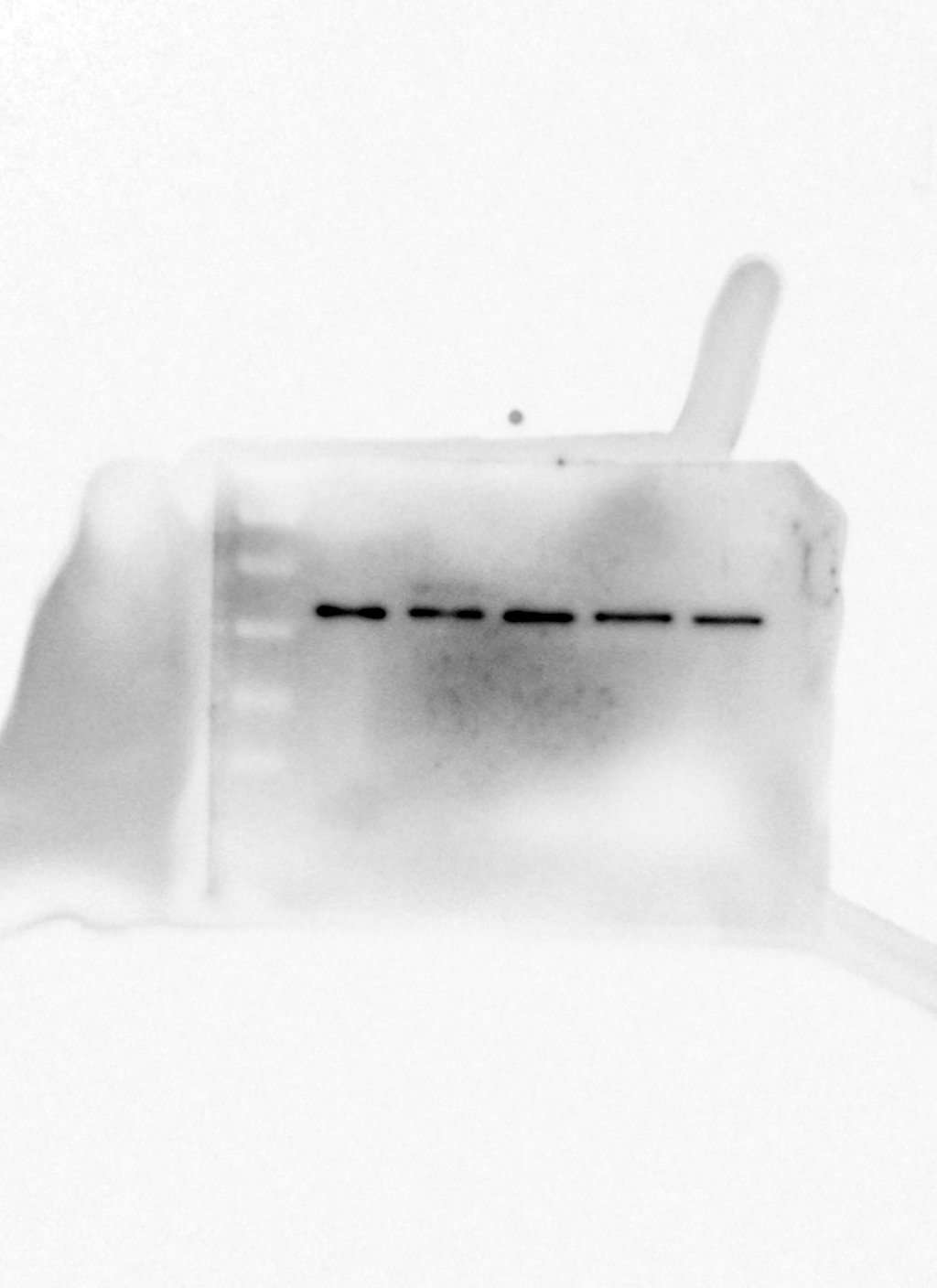

Supplement: Supplementary file 1 [file biomolecules-16-01059-s001.zip › File S1/Figure 6-8-11 Western blot original drawing/Figure 6 b SM22α/β-actin-2.tif]

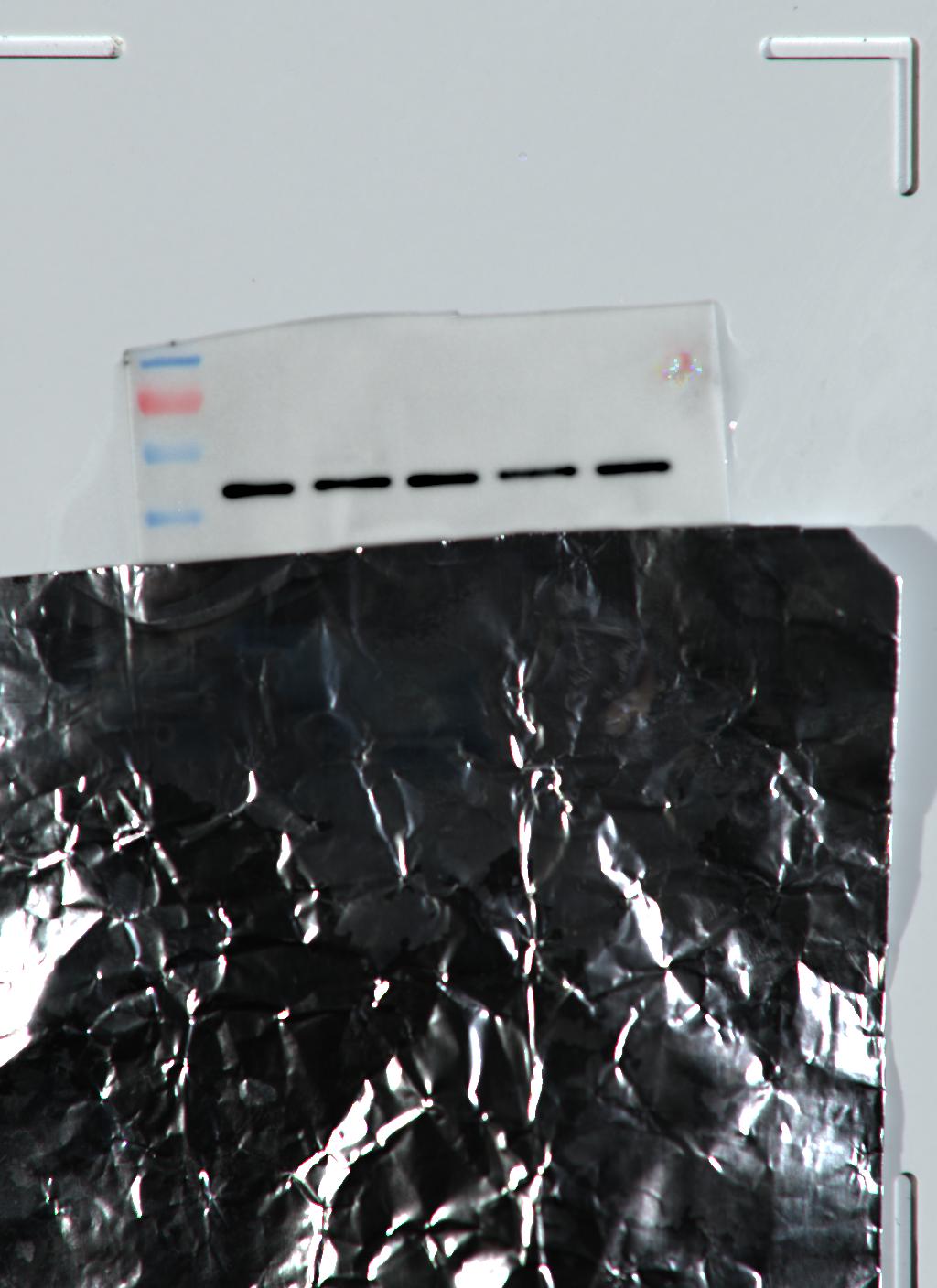

Supplement: Supplementary file 1 [file biomolecules-16-01059-s001.zip › File S1/Figure 6-8-11 Western blot original drawing/Figure 6 b SM22α/β-actin-3.jpg]

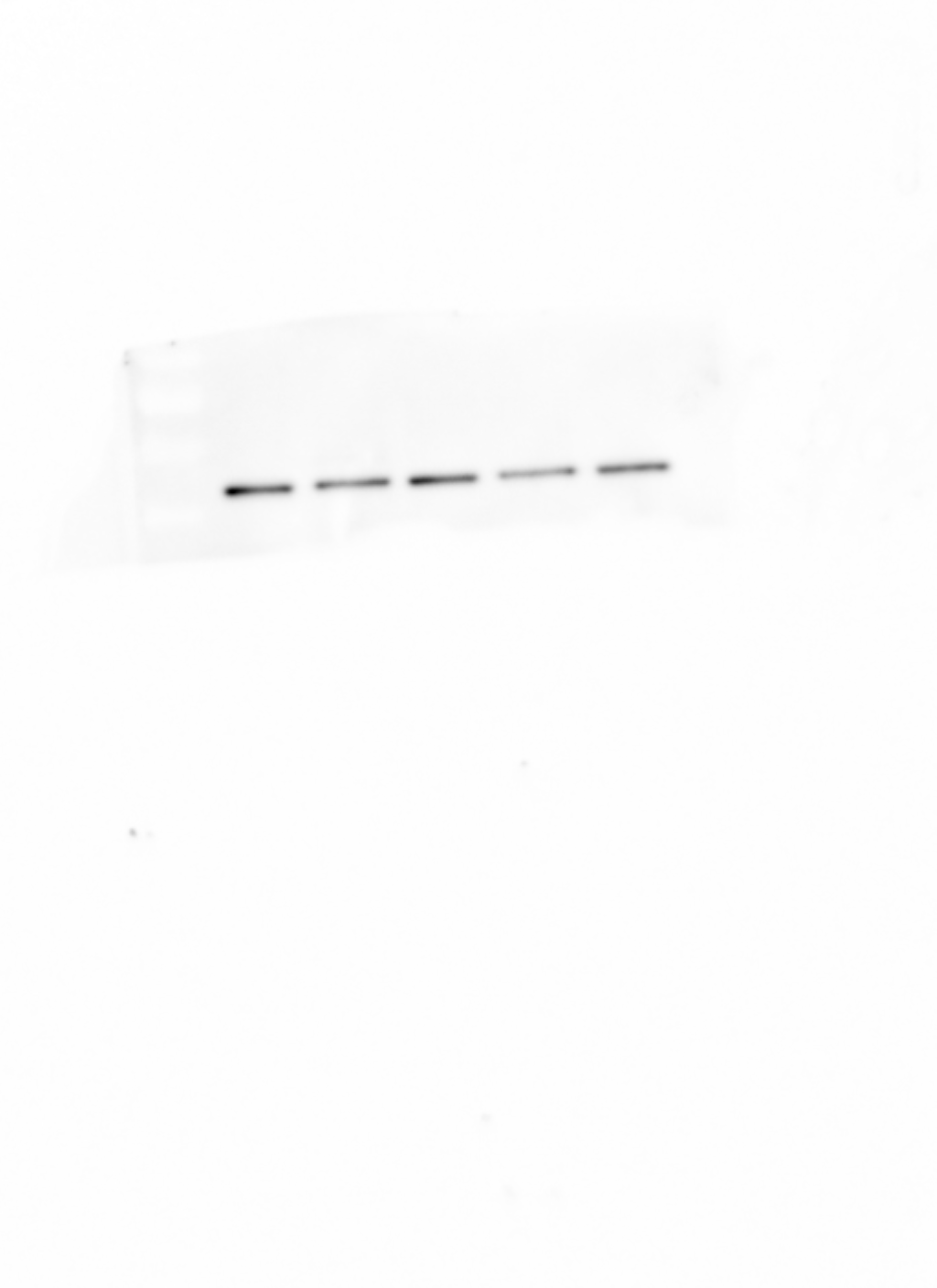

Supplement: Supplementary file 1 [file biomolecules-16-01059-s001.zip › File S1/Figure 6-8-11 Western blot original drawing/Figure 6 b SM22α/β-actin-3.tif]

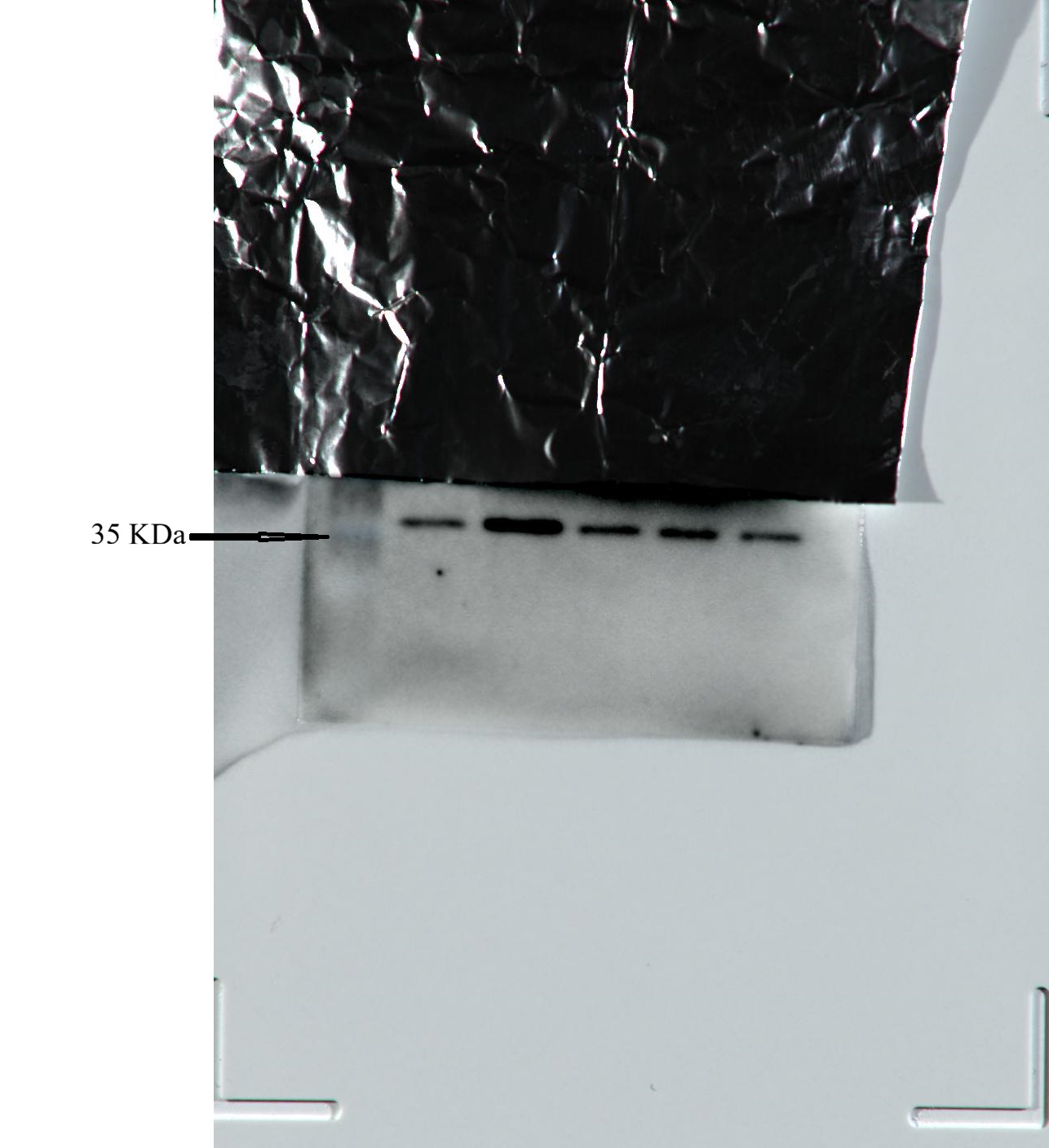

Supplement: Supplementary file 1 [file biomolecules-16-01059-s001.zip › File S1/Figure 6-8-11 Western blot original drawing/Figure 6 c PCNA/PCNA-1.jpg]

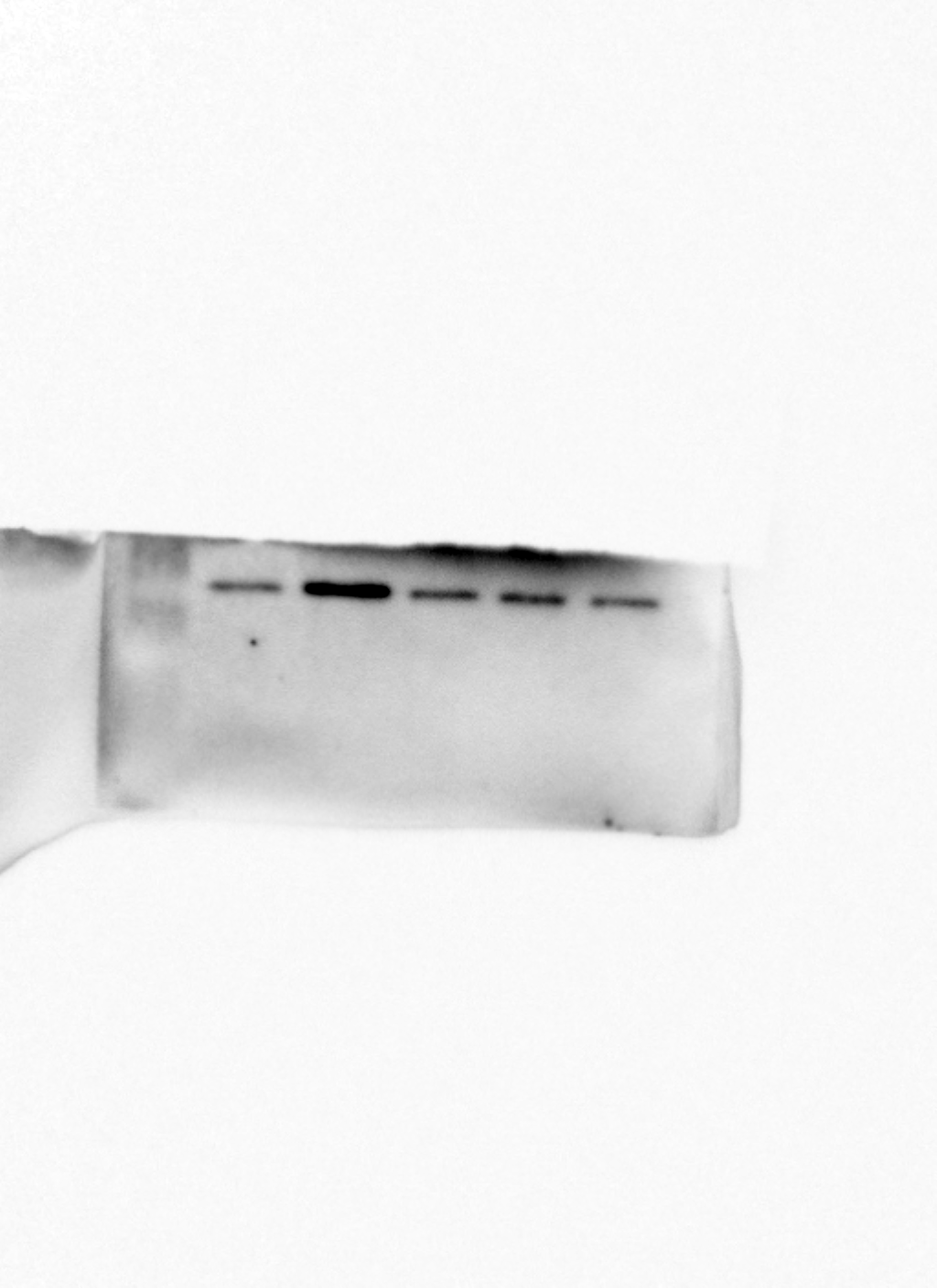

Supplement: Supplementary file 1 [file biomolecules-16-01059-s001.zip › File S1/Figure 6-8-11 Western blot original drawing/Figure 6 c PCNA/PCNA-1.tif]

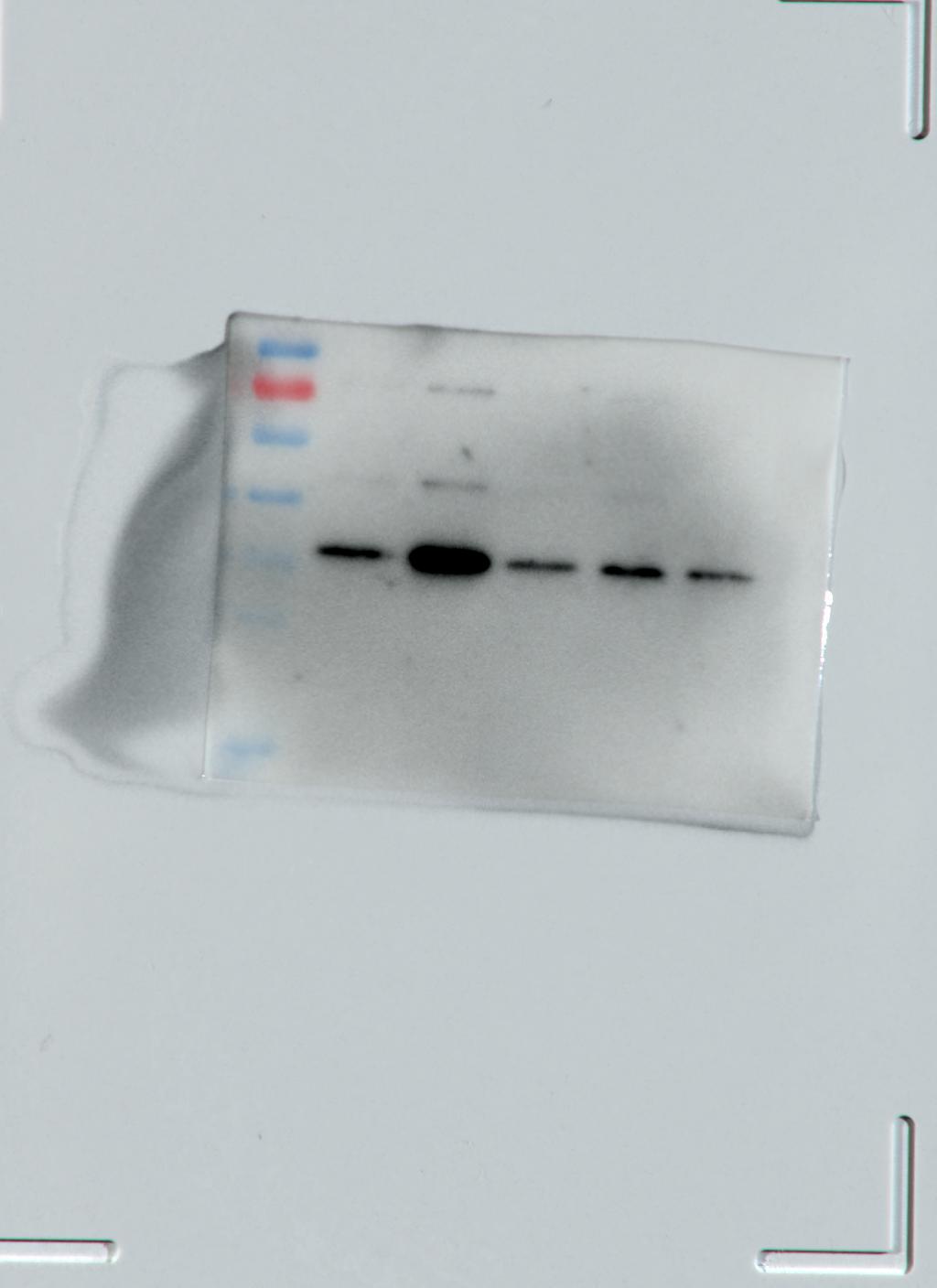

Supplement: Supplementary file 1 [file biomolecules-16-01059-s001.zip › File S1/Figure 6-8-11 Western blot original drawing/Figure 6 c PCNA/PCNA-2.jpg]

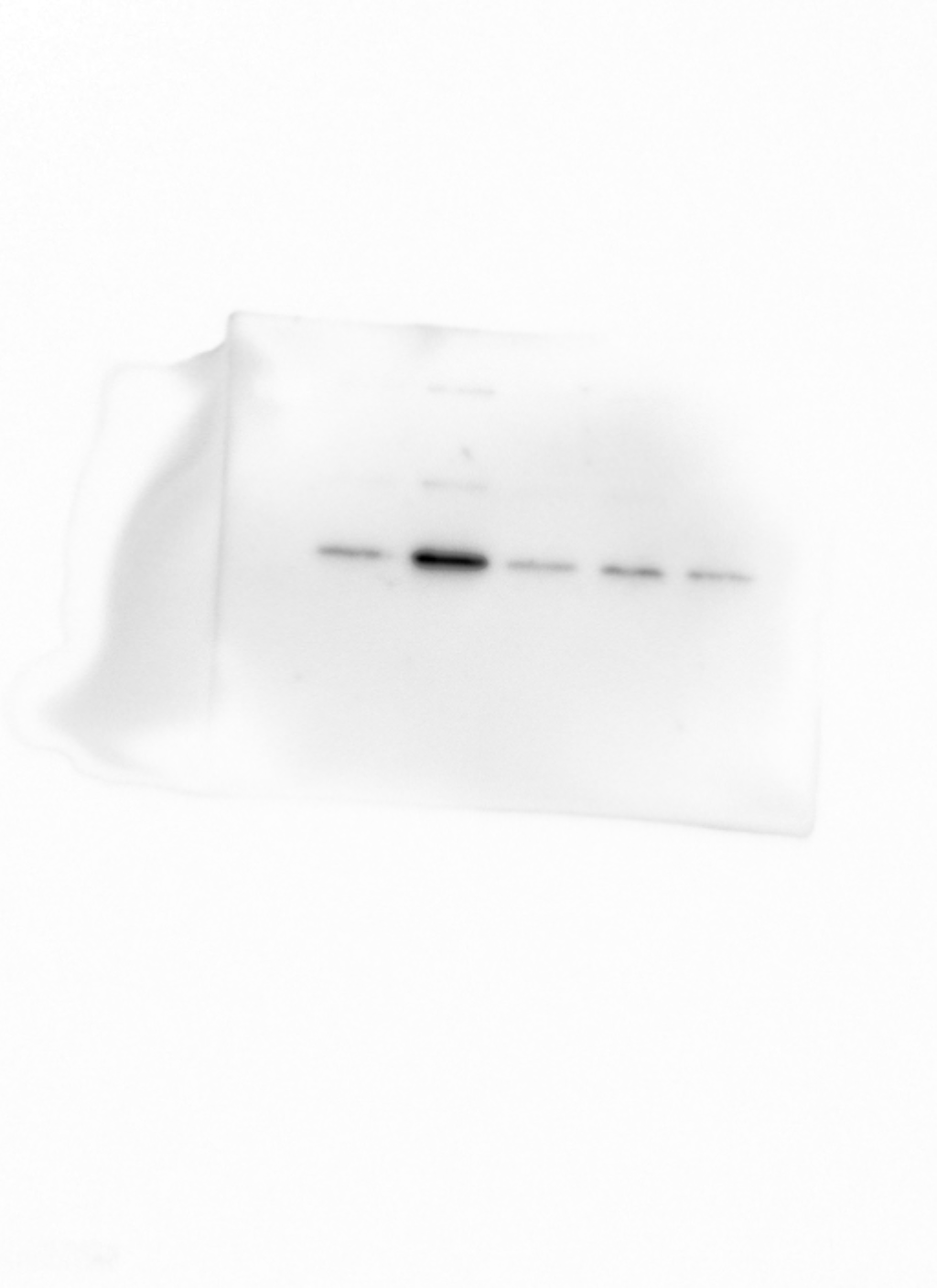

Supplement: Supplementary file 1 [file biomolecules-16-01059-s001.zip › File S1/Figure 6-8-11 Western blot original drawing/Figure 6 c PCNA/PCNA-2.tif]

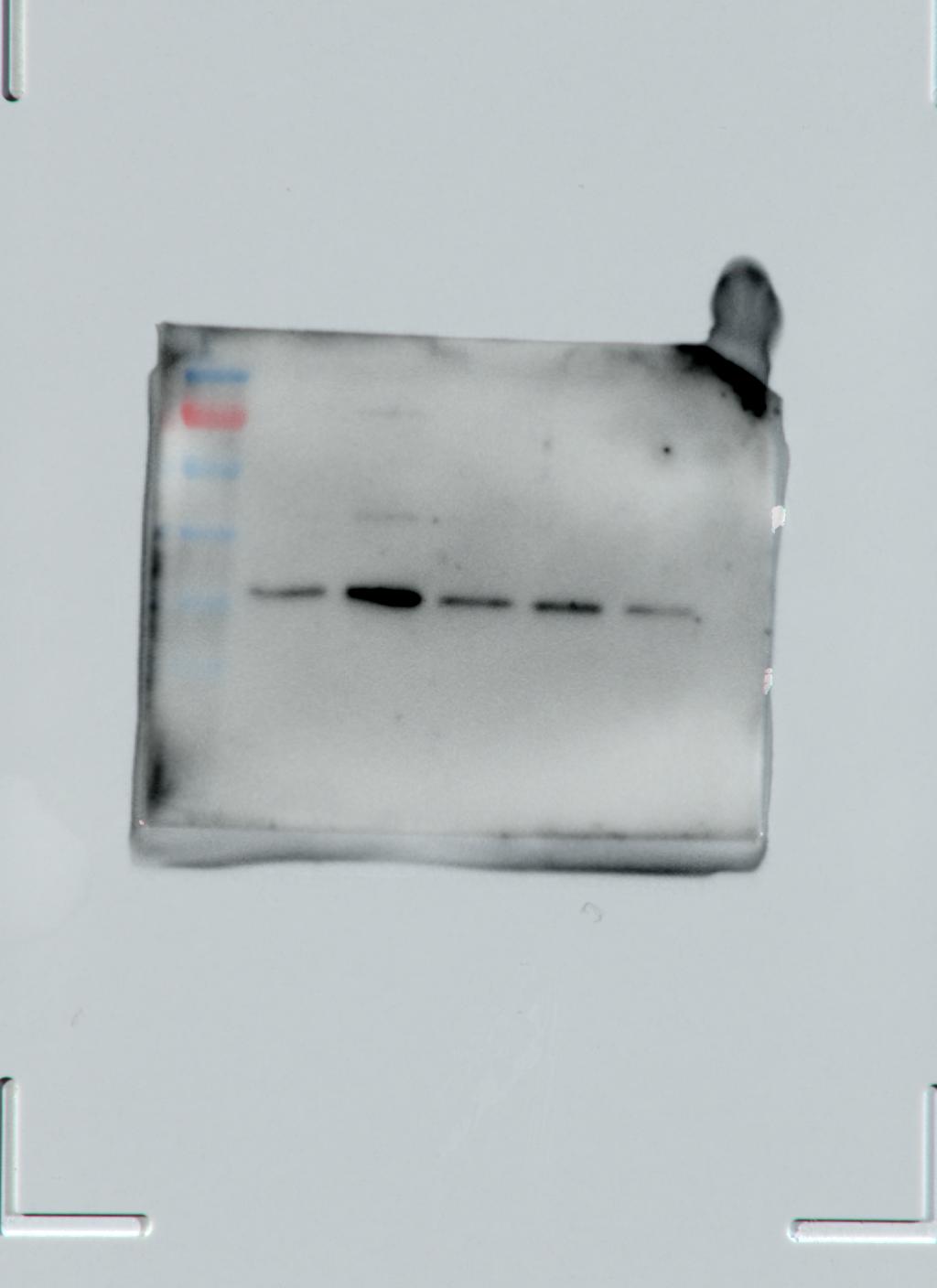

Supplement: Supplementary file 1 [file biomolecules-16-01059-s001.zip › File S1/Figure 6-8-11 Western blot original drawing/Figure 6 c PCNA/PCNA-3.jpg]

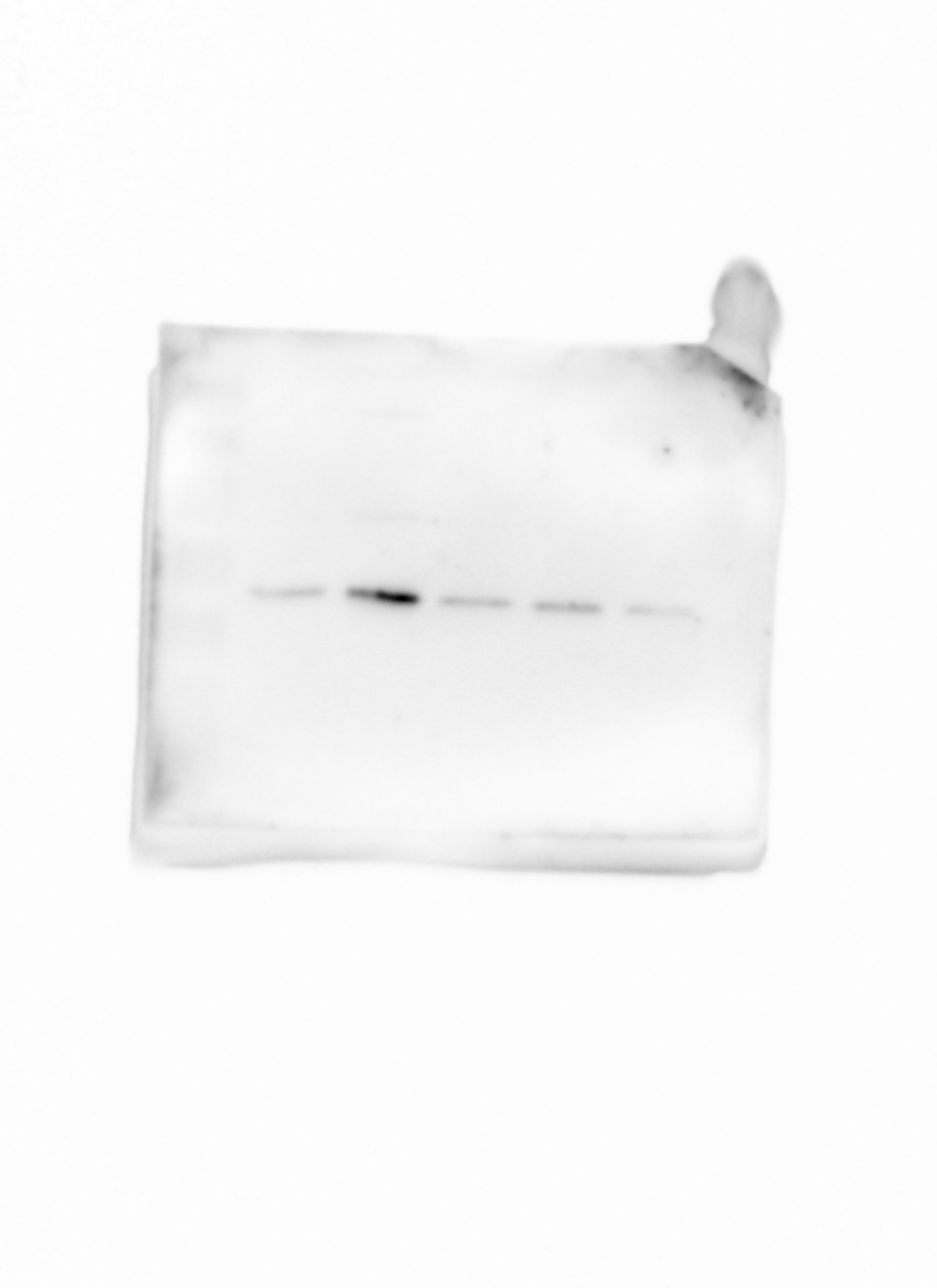

Supplement: Supplementary file 1 [file biomolecules-16-01059-s001.zip › File S1/Figure 6-8-11 Western blot original drawing/Figure 6 c PCNA/PCNA-3.tif]

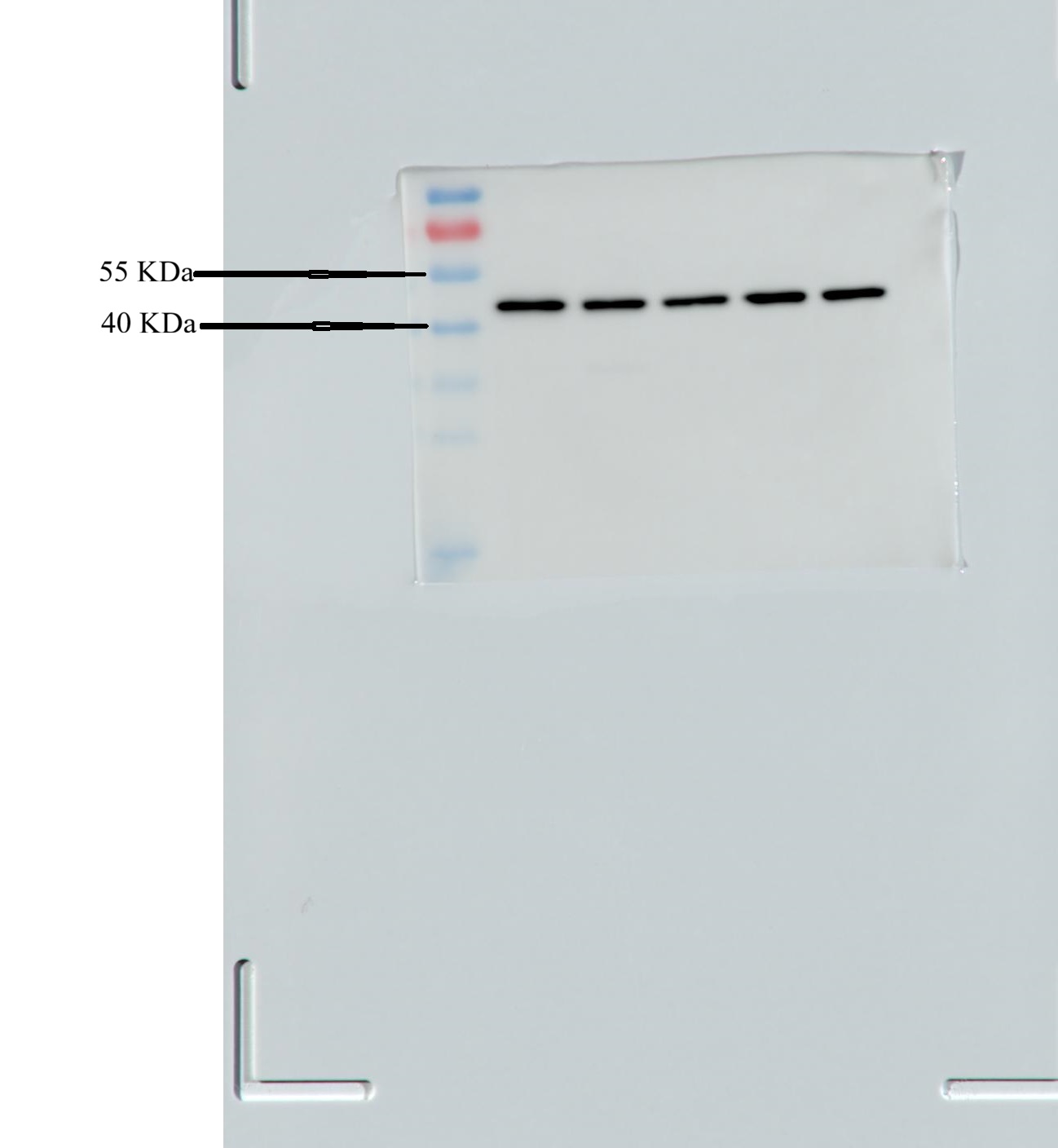

Supplement: Supplementary file 1 [file biomolecules-16-01059-s001.zip › File S1/Figure 6-8-11 Western blot original drawing/Figure 6 c PCNA/β-actin-1.jpg]

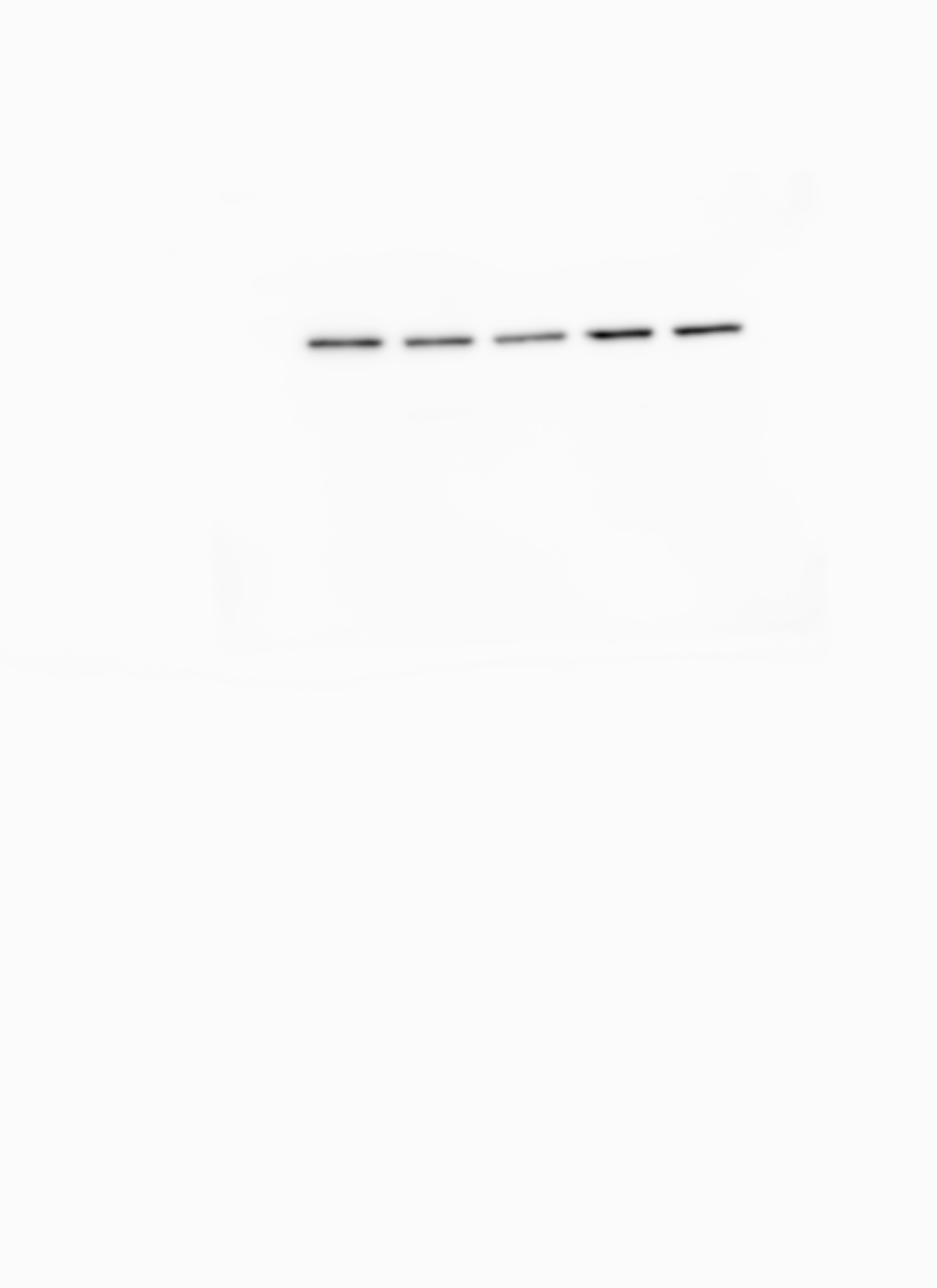

Supplement: Supplementary file 1 [file biomolecules-16-01059-s001.zip › File S1/Figure 6-8-11 Western blot original drawing/Figure 6 c PCNA/β-actin-1.tif]

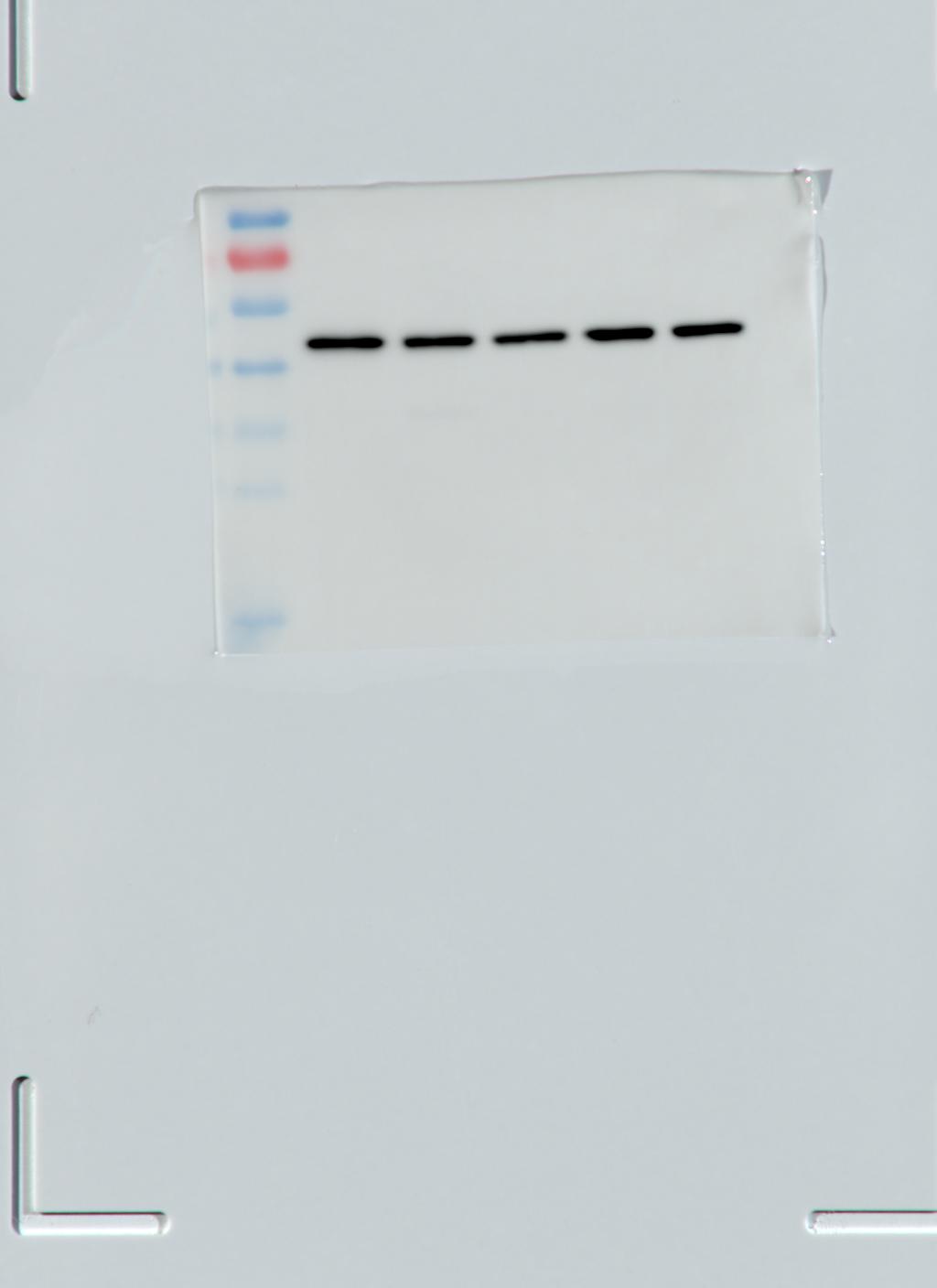

Supplement: Supplementary file 1 [file biomolecules-16-01059-s001.zip › File S1/Figure 6-8-11 Western blot original drawing/Figure 6 c PCNA/β-actin-2.jpg]

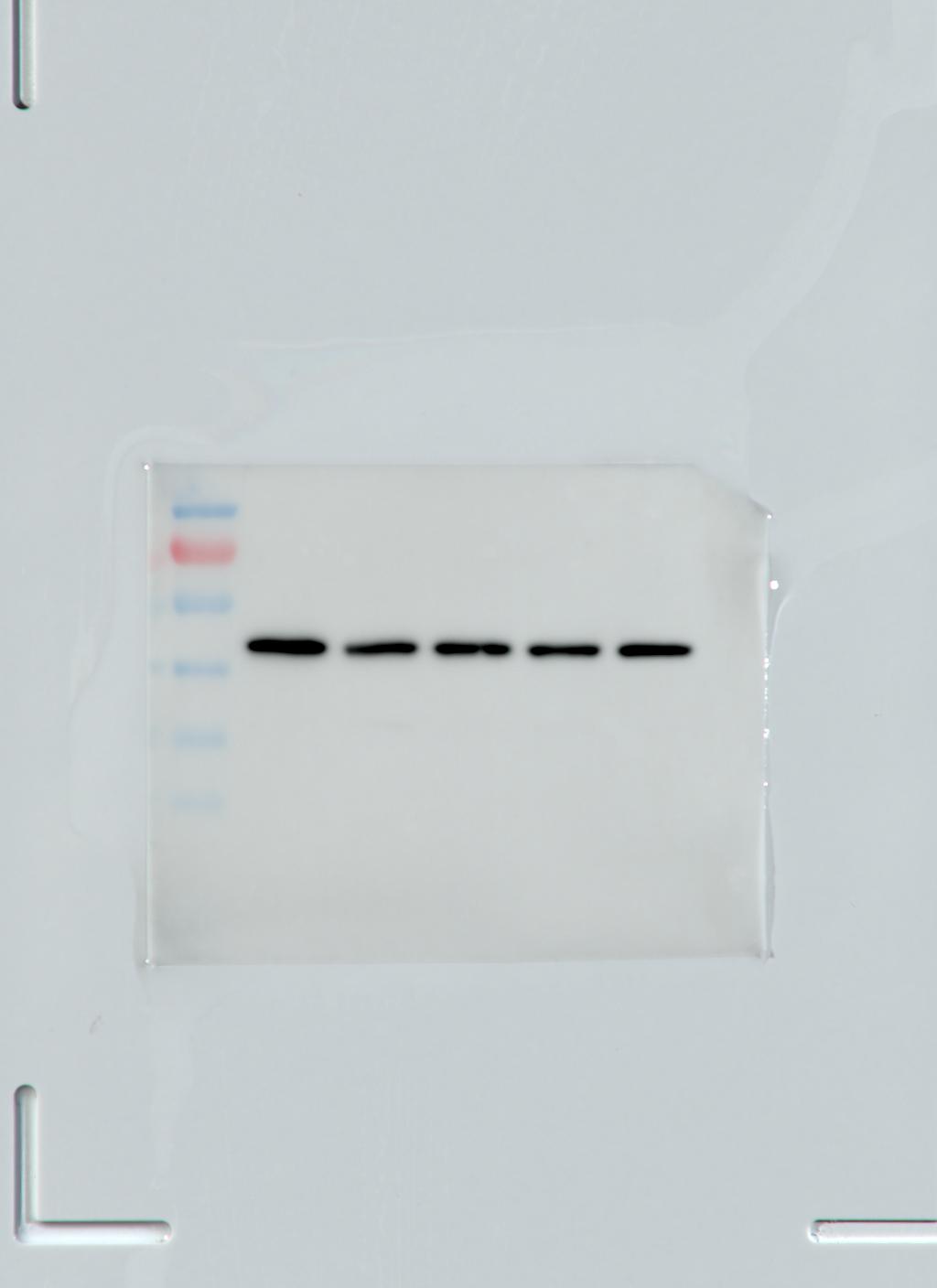

Supplement: Supplementary file 1 [file biomolecules-16-01059-s001.zip › File S1/Figure 6-8-11 Western blot original drawing/Figure 6 c PCNA/β-actin-3.jpg]

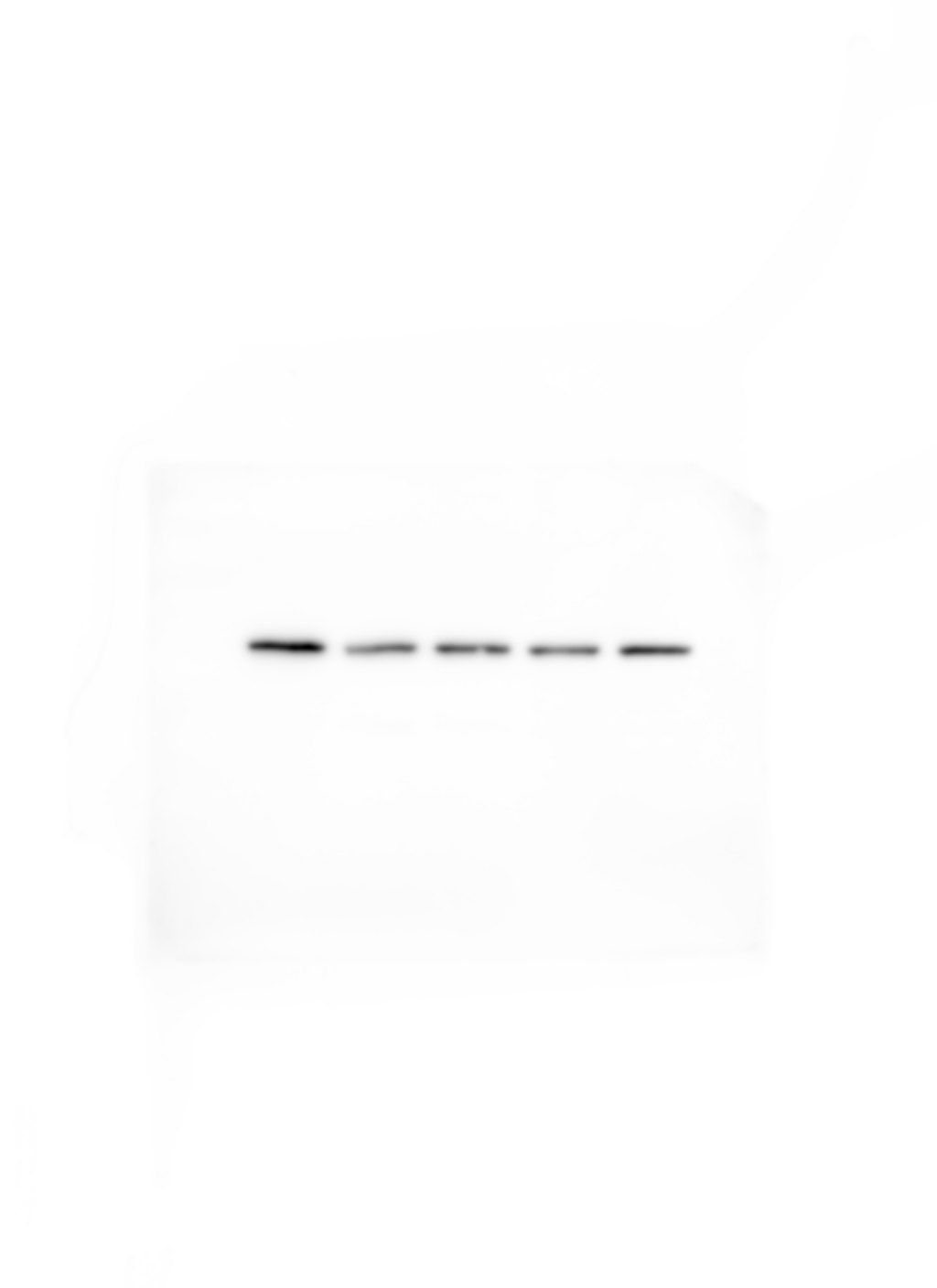

Supplement: Supplementary file 1 [file biomolecules-16-01059-s001.zip › File S1/Figure 6-8-11 Western blot original drawing/Figure 6 c PCNA/β-actin-3.tif]

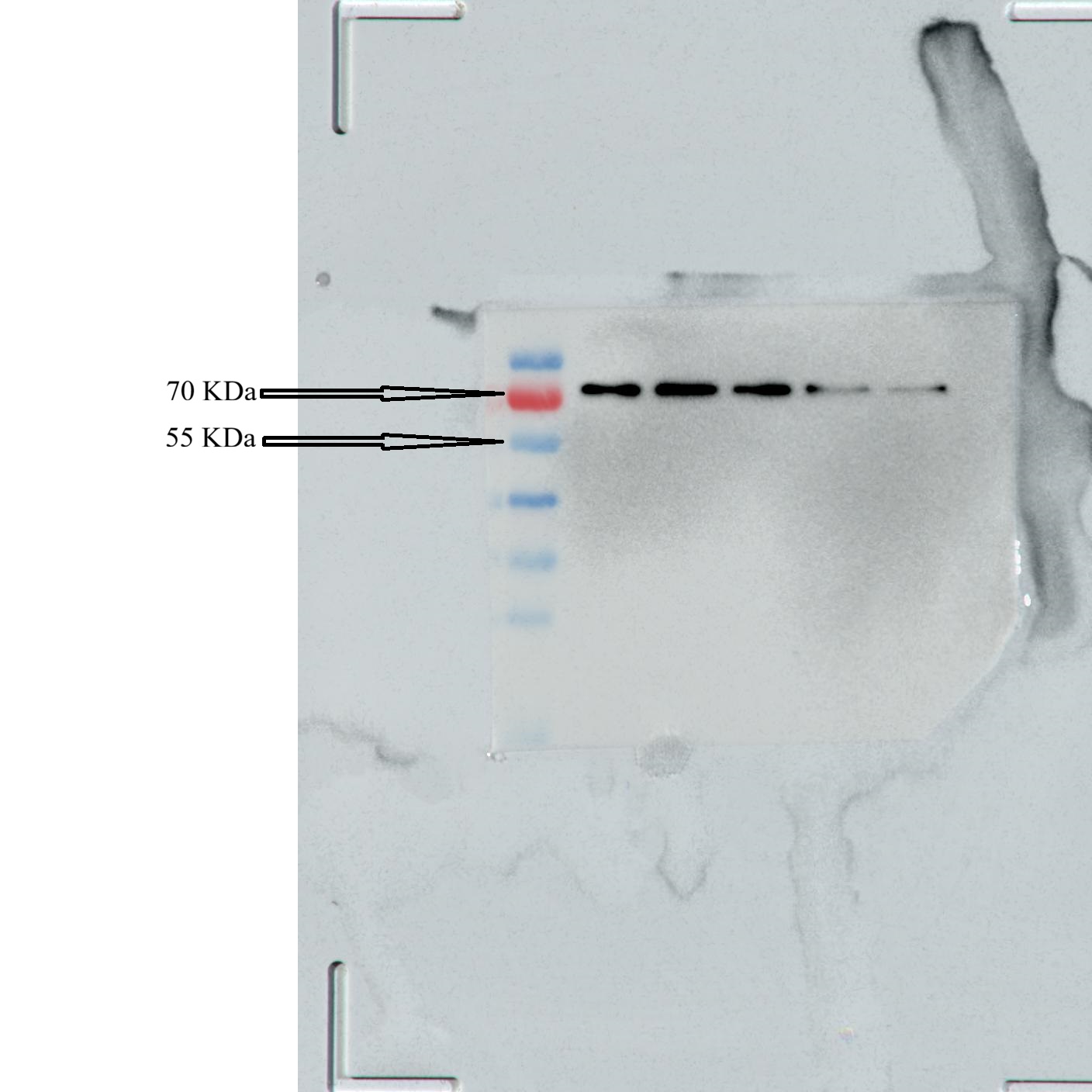

Supplement: Supplementary file 1 [file biomolecules-16-01059-s001.zip › File S1/Figure 6-8-11 Western blot original drawing/Figure 6 d OPN/OPN-1.jpg]

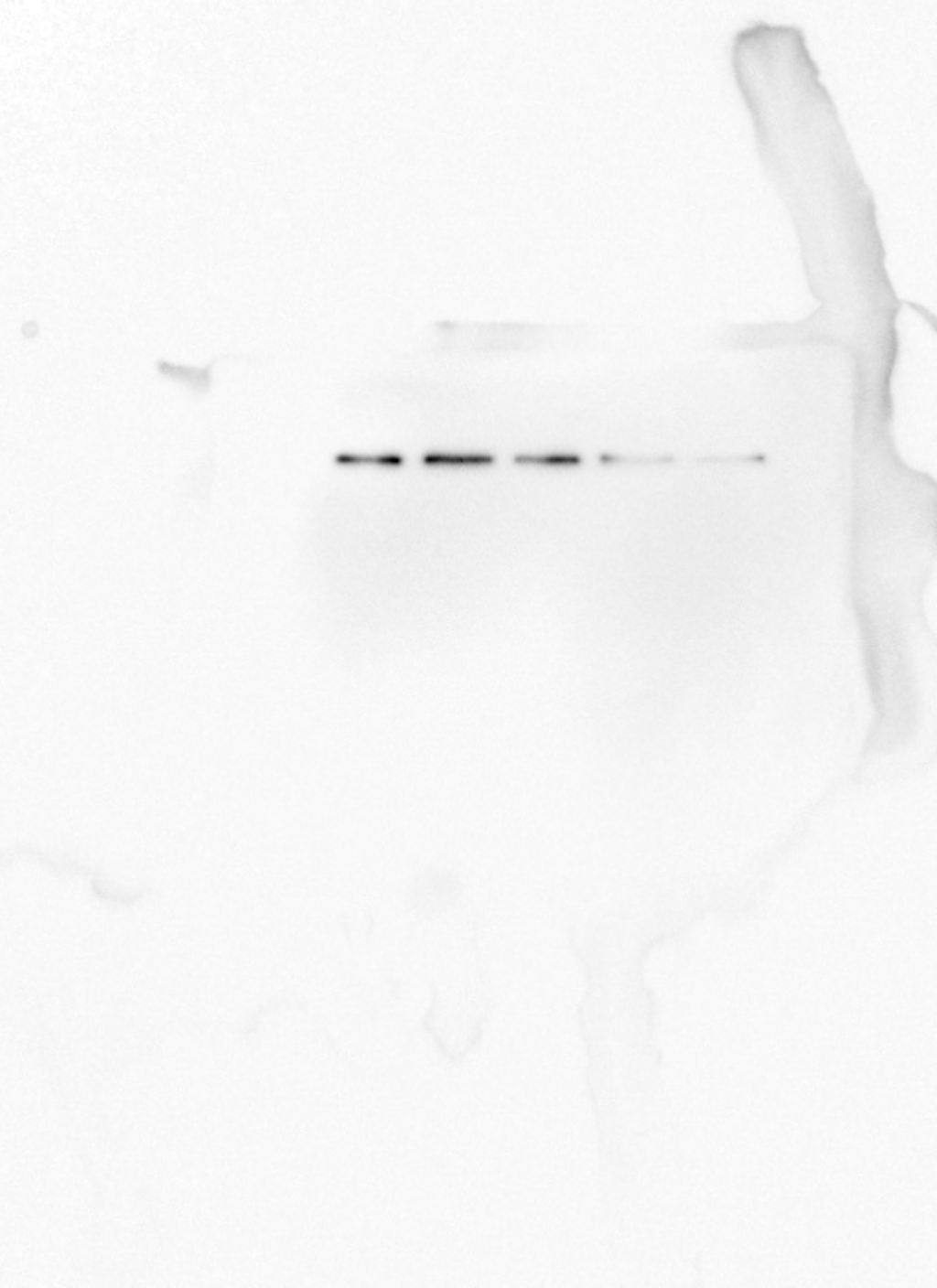

Supplement: Supplementary file 1 [file biomolecules-16-01059-s001.zip › File S1/Figure 6-8-11 Western blot original drawing/Figure 6 d OPN/OPN-1.tif]

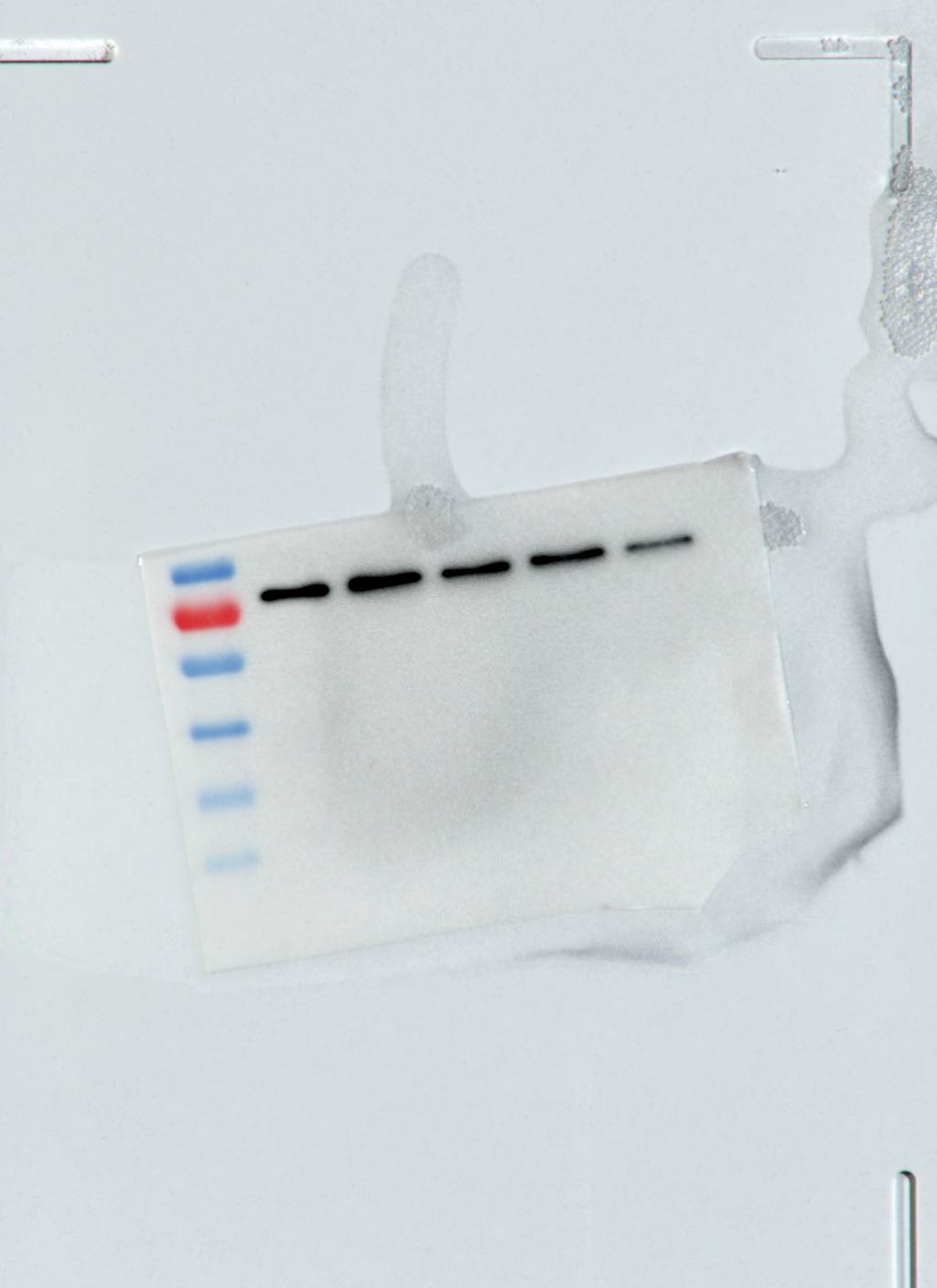

Supplement: Supplementary file 1 [file biomolecules-16-01059-s001.zip › File S1/Figure 6-8-11 Western blot original drawing/Figure 6 d OPN/OPN-2.jpg]

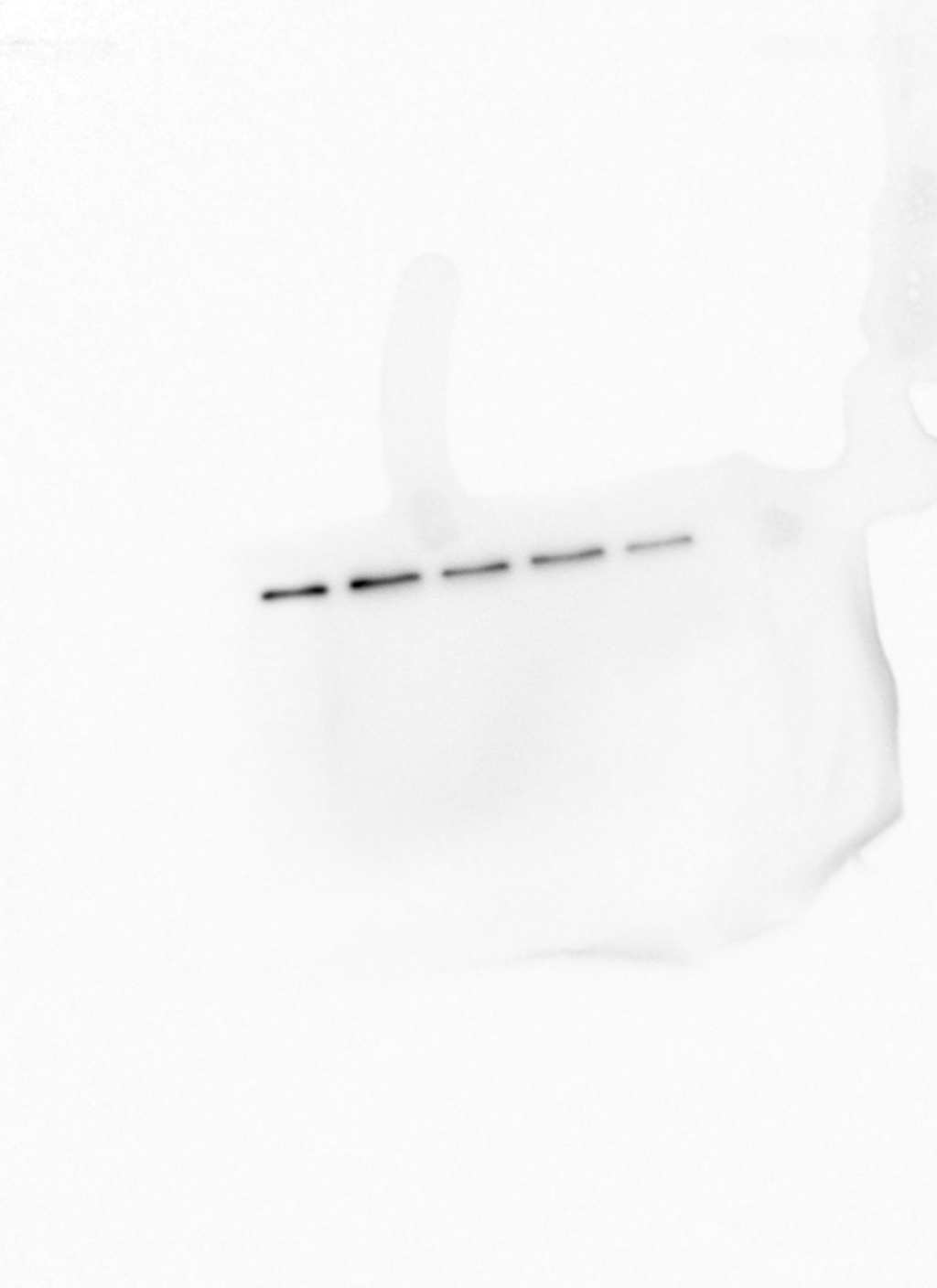

Supplement: Supplementary file 1 [file biomolecules-16-01059-s001.zip › File S1/Figure 6-8-11 Western blot original drawing/Figure 6 d OPN/OPN-2.tif]

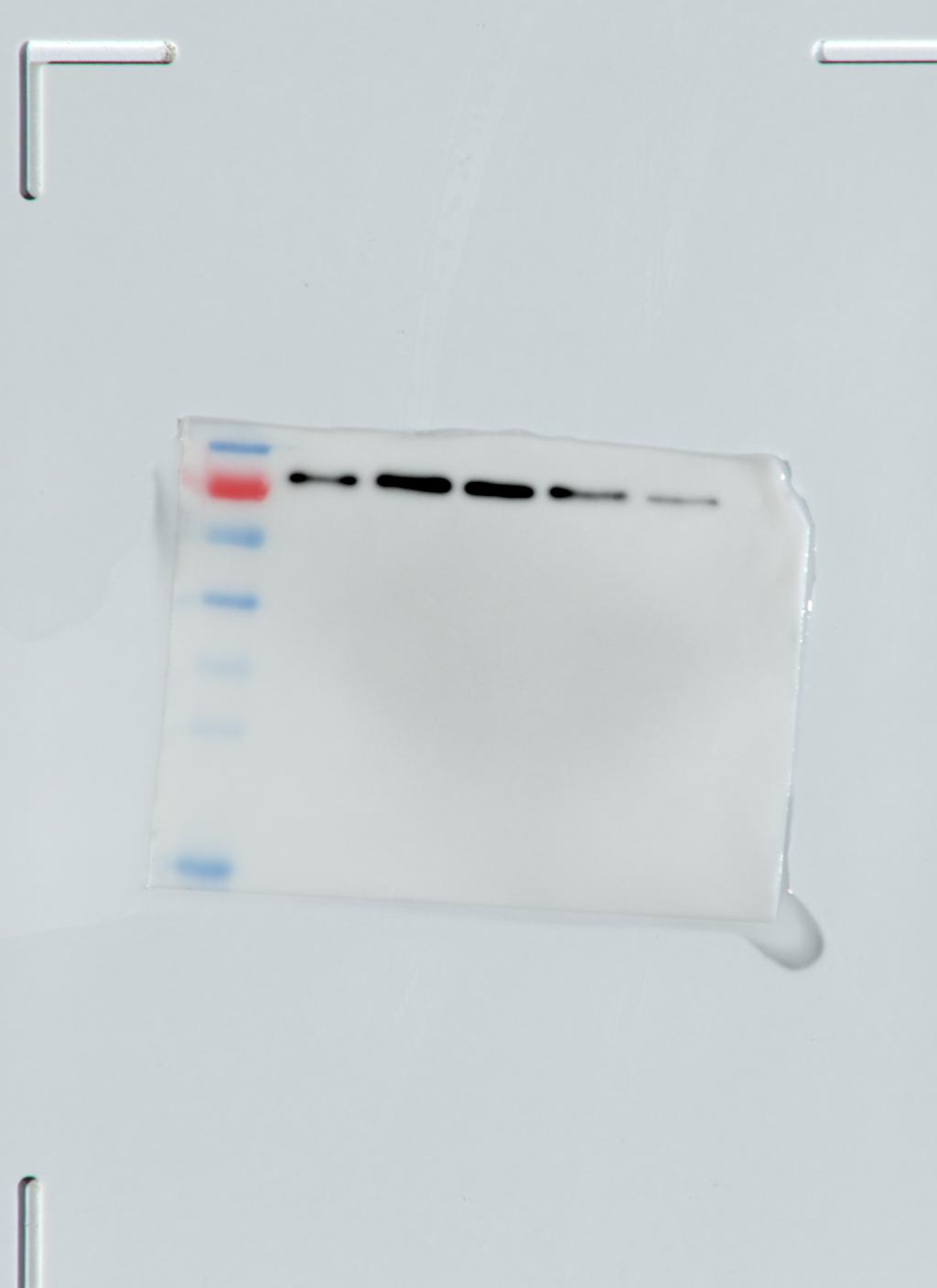

Supplement: Supplementary file 1 [file biomolecules-16-01059-s001.zip › File S1/Figure 6-8-11 Western blot original drawing/Figure 6 d OPN/OPN-3.jpg]

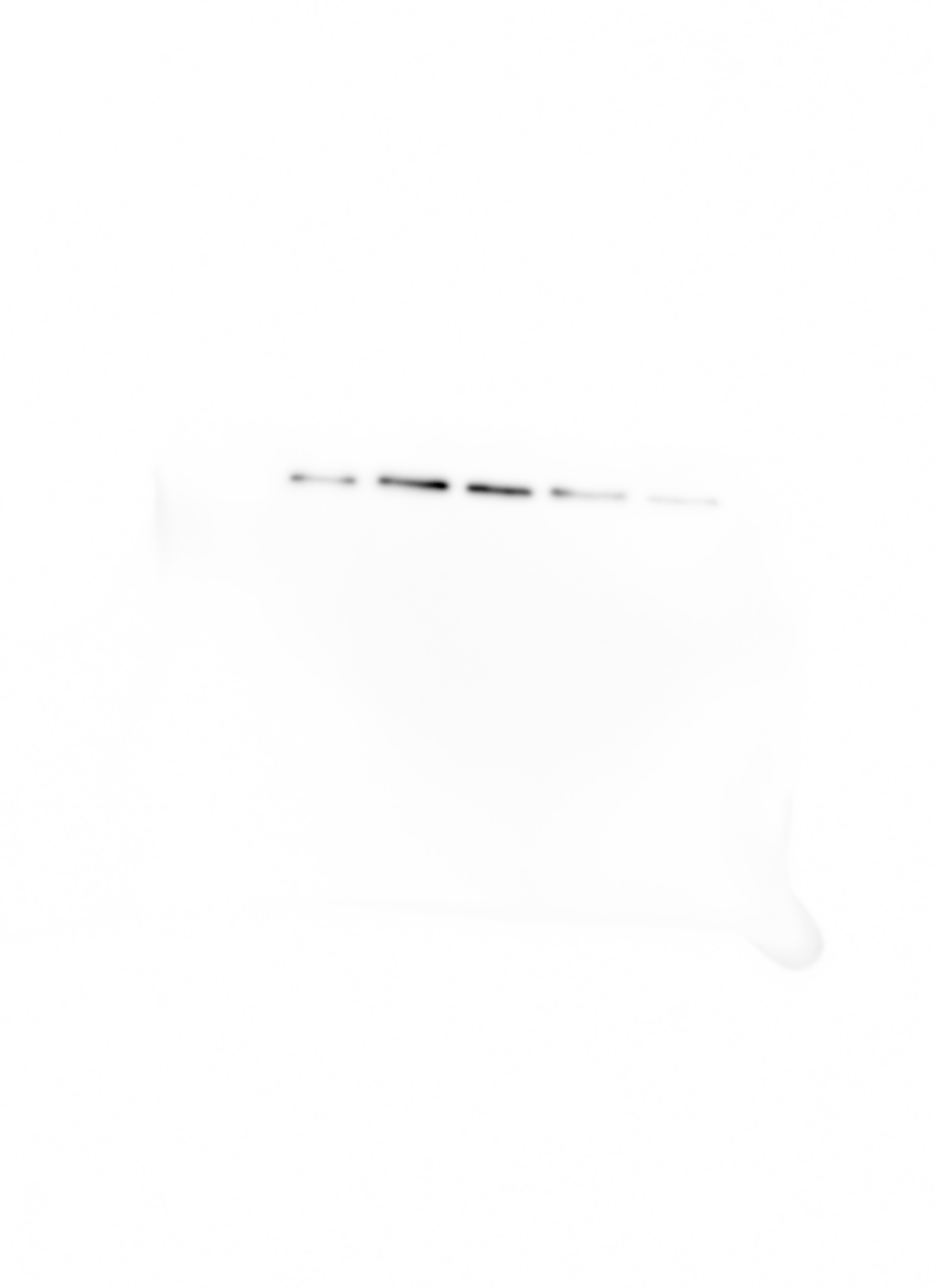

Supplement: Supplementary file 1 [file biomolecules-16-01059-s001.zip › File S1/Figure 6-8-11 Western blot original drawing/Figure 6 d OPN/OPN-3.tif]

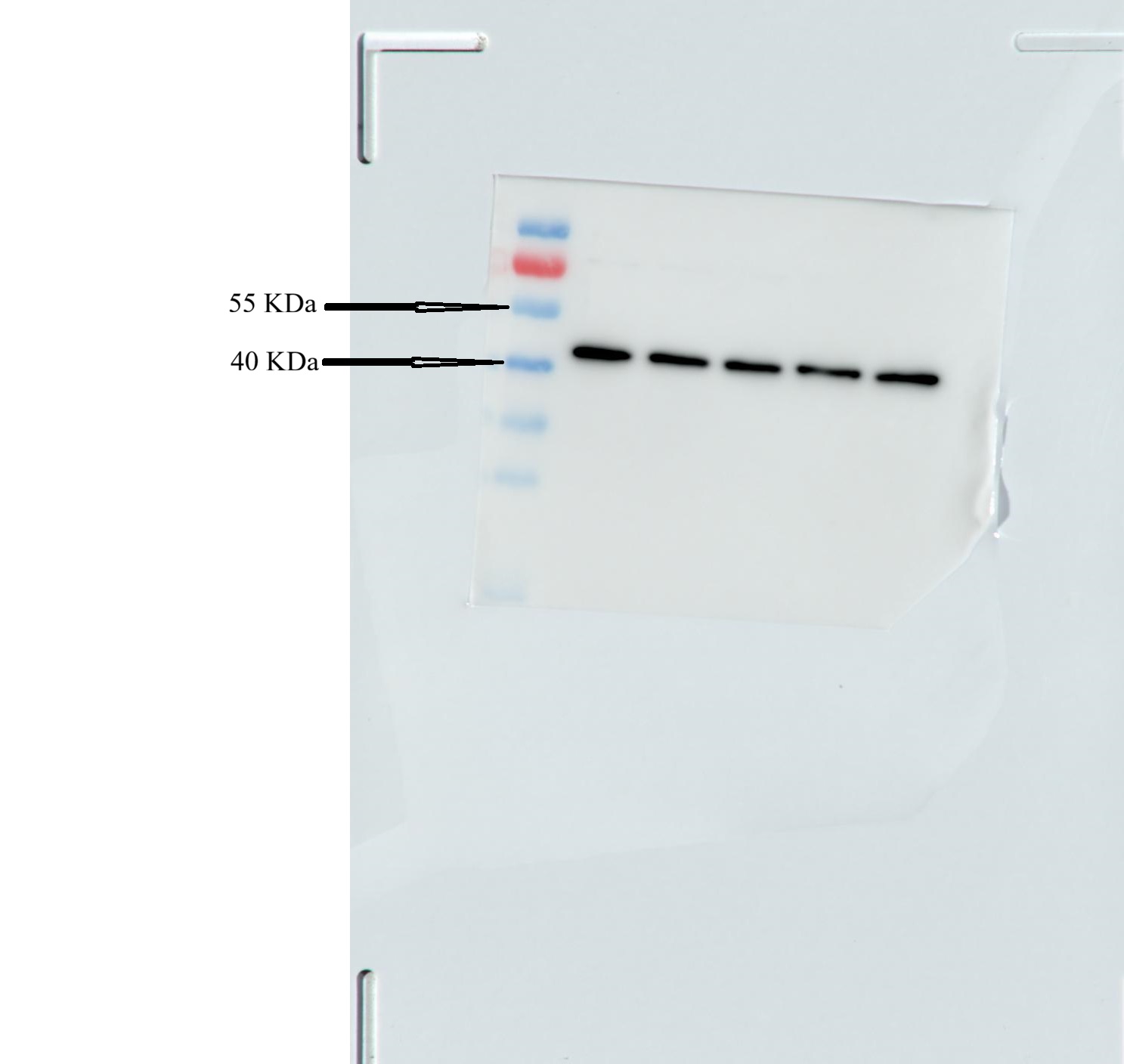

Supplement: Supplementary file 1 [file biomolecules-16-01059-s001.zip › File S1/Figure 6-8-11 Western blot original drawing/Figure 6 d OPN/β-actin-1.jpg]

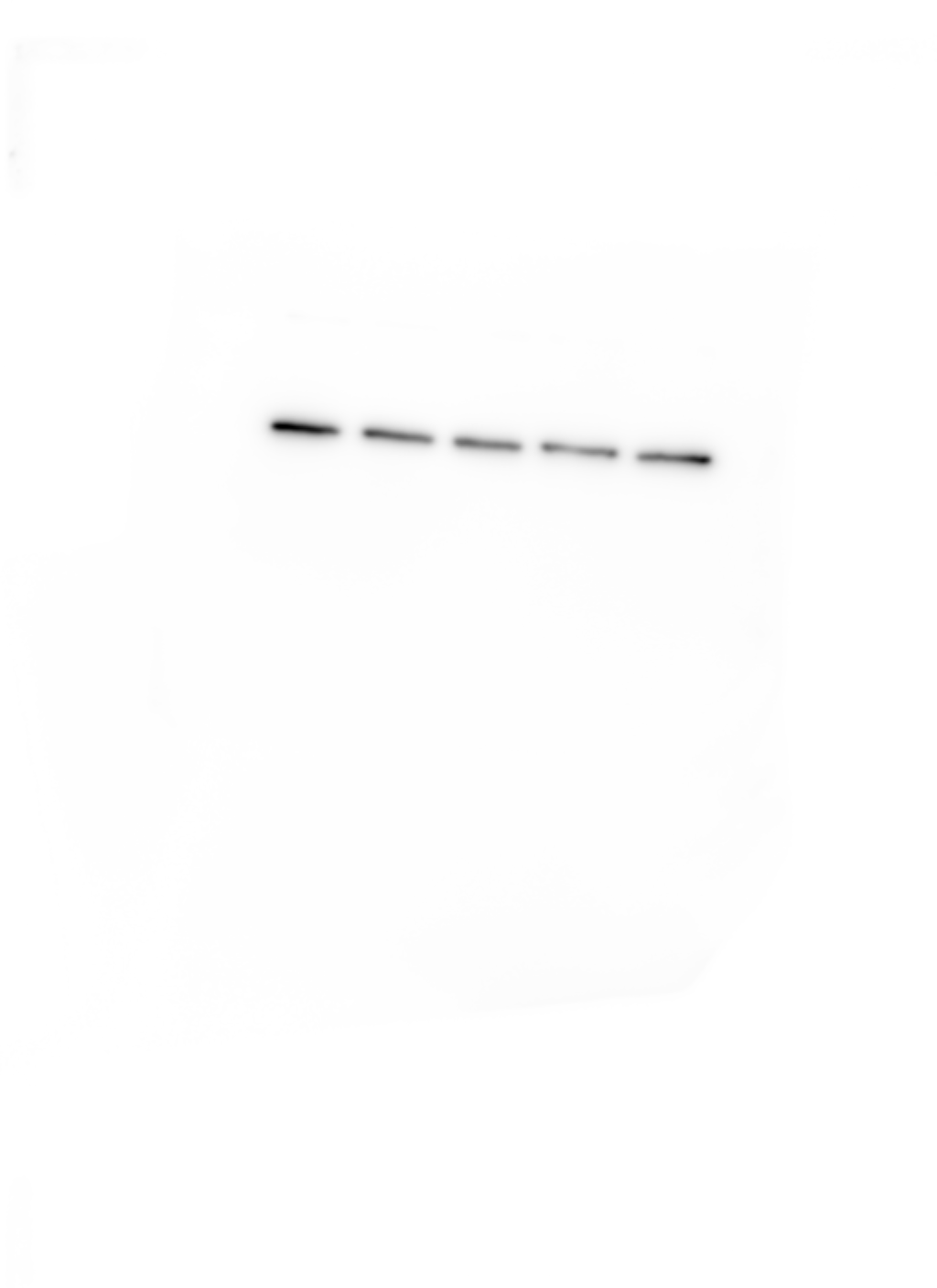

Supplement: Supplementary file 1 [file biomolecules-16-01059-s001.zip › File S1/Figure 6-8-11 Western blot original drawing/Figure 6 d OPN/β-actin-1.tif]

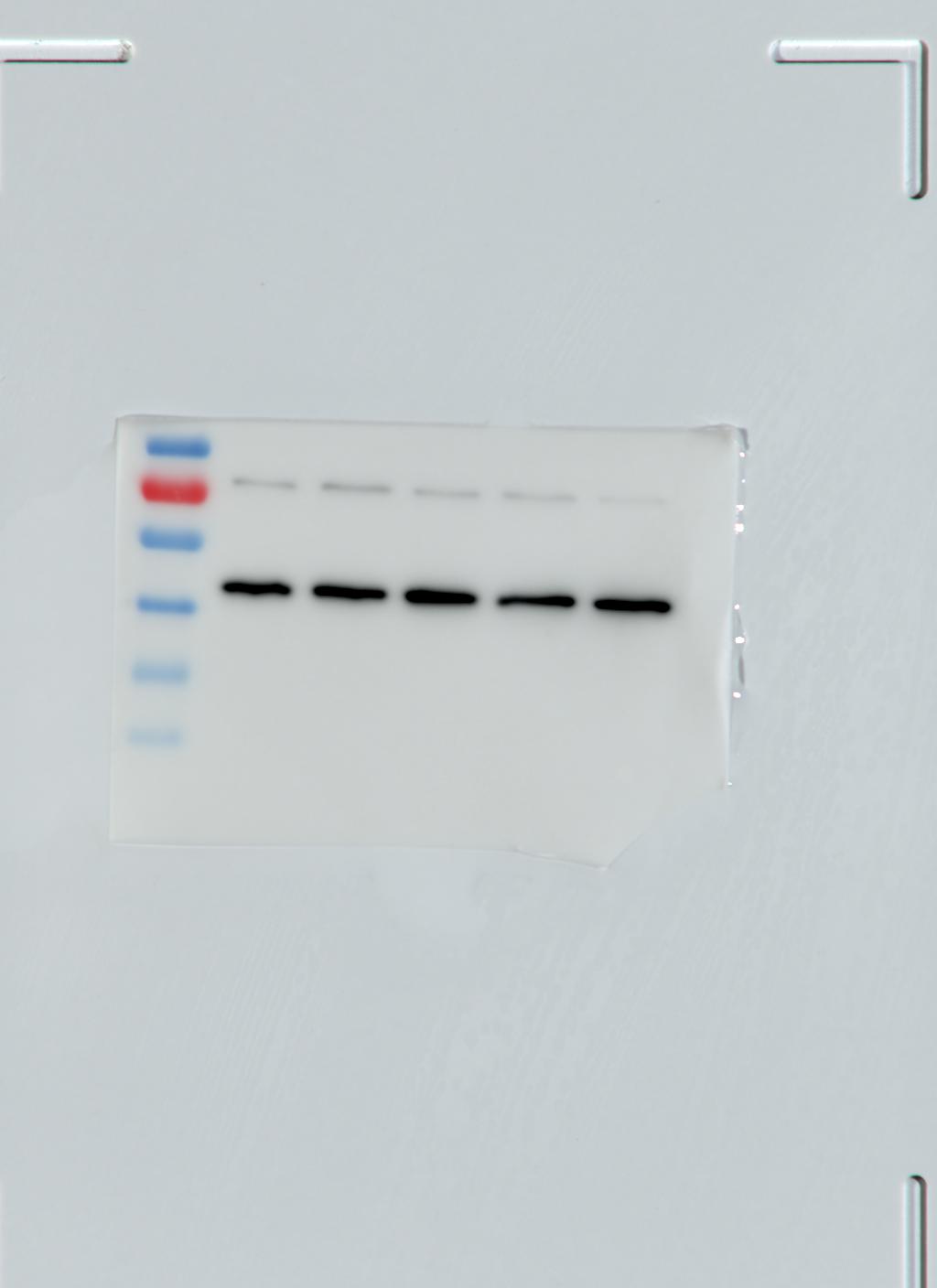

Supplement: Supplementary file 1 [file biomolecules-16-01059-s001.zip › File S1/Figure 6-8-11 Western blot original drawing/Figure 6 d OPN/β-actin-2.jpg]

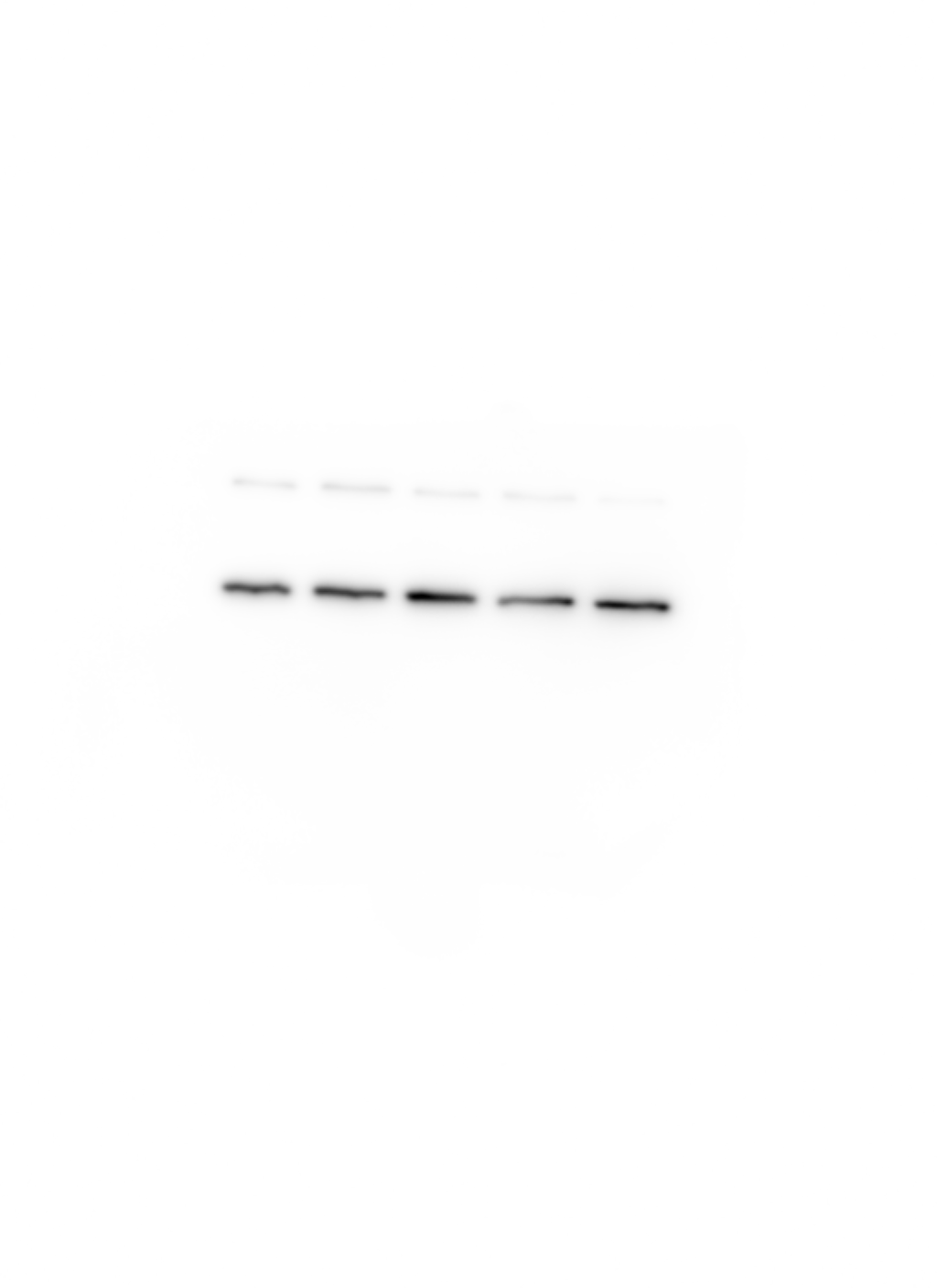

Supplement: Supplementary file 1 [file biomolecules-16-01059-s001.zip › File S1/Figure 6-8-11 Western blot original drawing/Figure 6 d OPN/β-actin-2.tif]

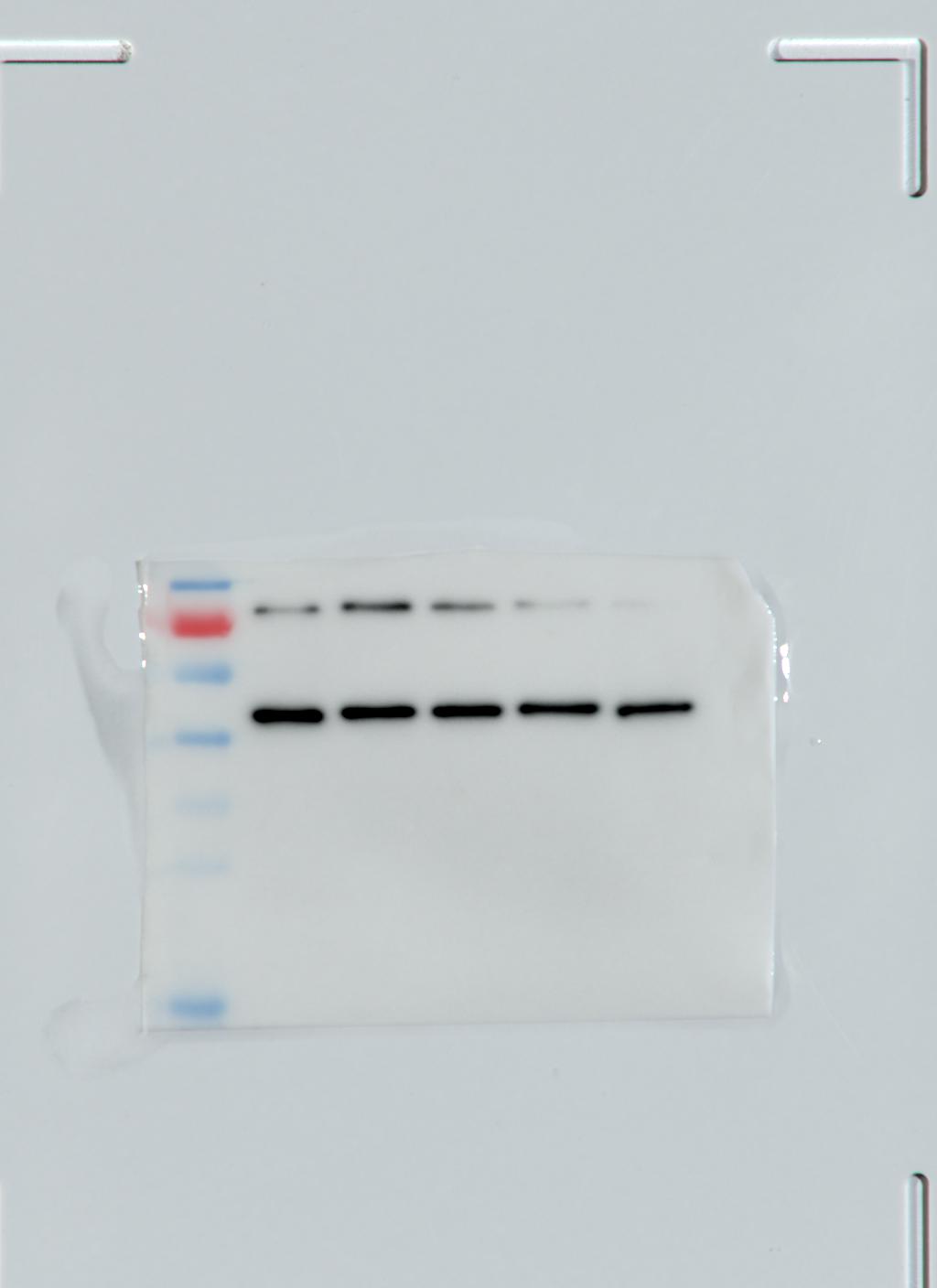

Supplement: Supplementary file 1 [file biomolecules-16-01059-s001.zip › File S1/Figure 6-8-11 Western blot original drawing/Figure 6 d OPN/β-actin-3.jpg]

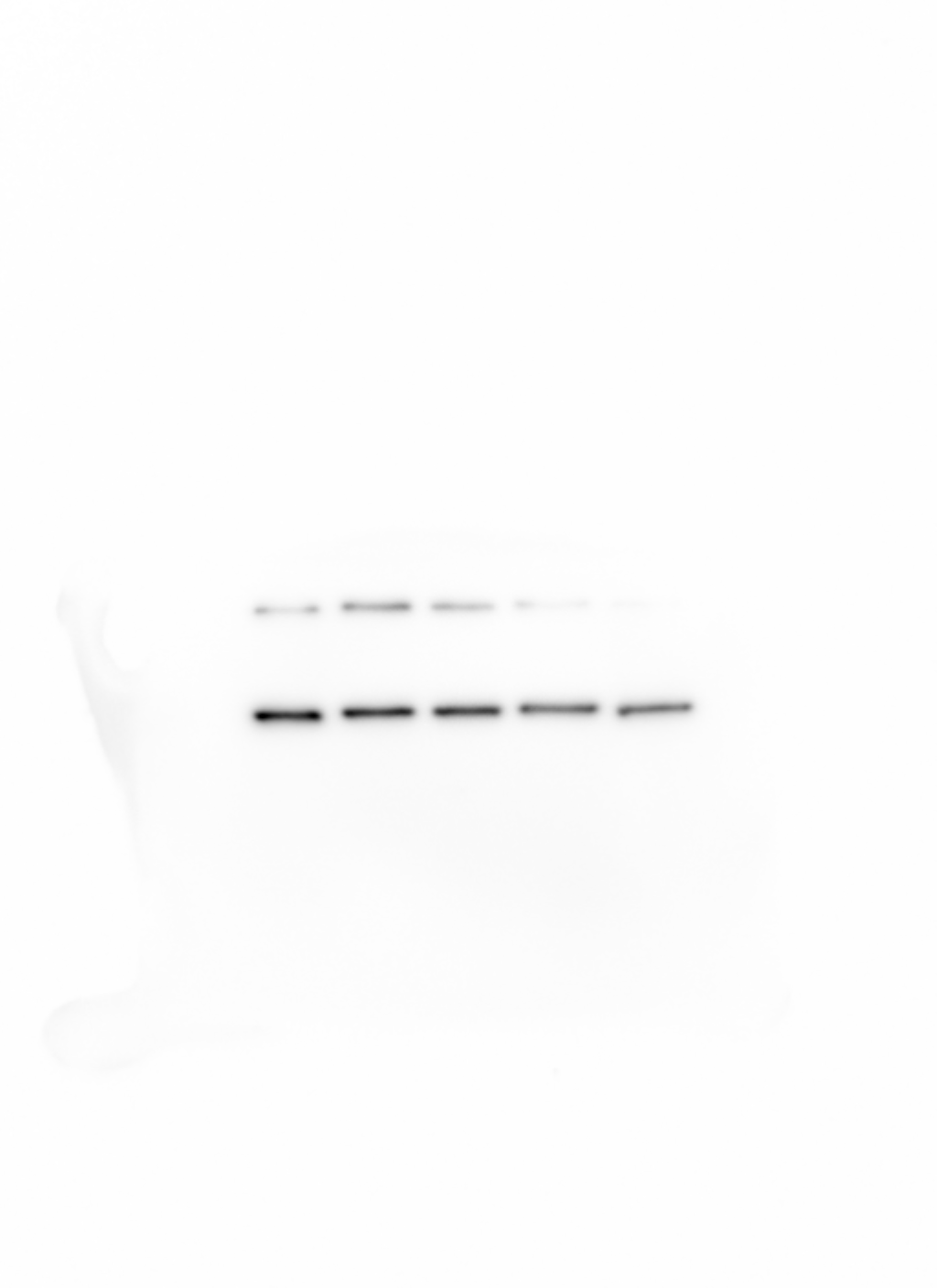

Supplement: Supplementary file 1 [file biomolecules-16-01059-s001.zip › File S1/Figure 6-8-11 Western blot original drawing/Figure 6 d OPN/β-actin-3.tif]

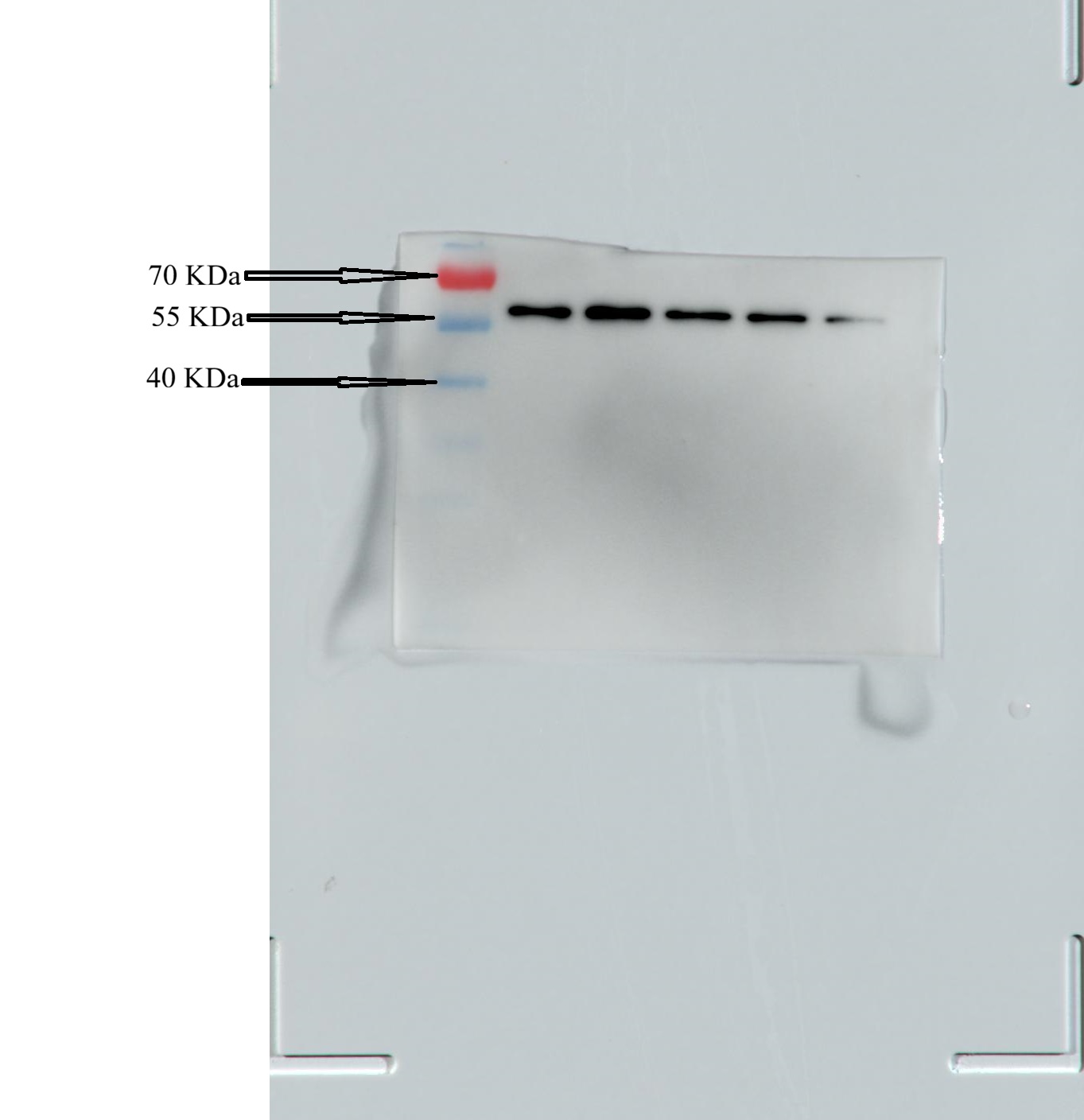

Supplement: Supplementary file 1 [file biomolecules-16-01059-s001.zip › File S1/Figure 6-8-11 Western blot original drawing/Figure 6 e vimentin/vimentin-1.jpg]

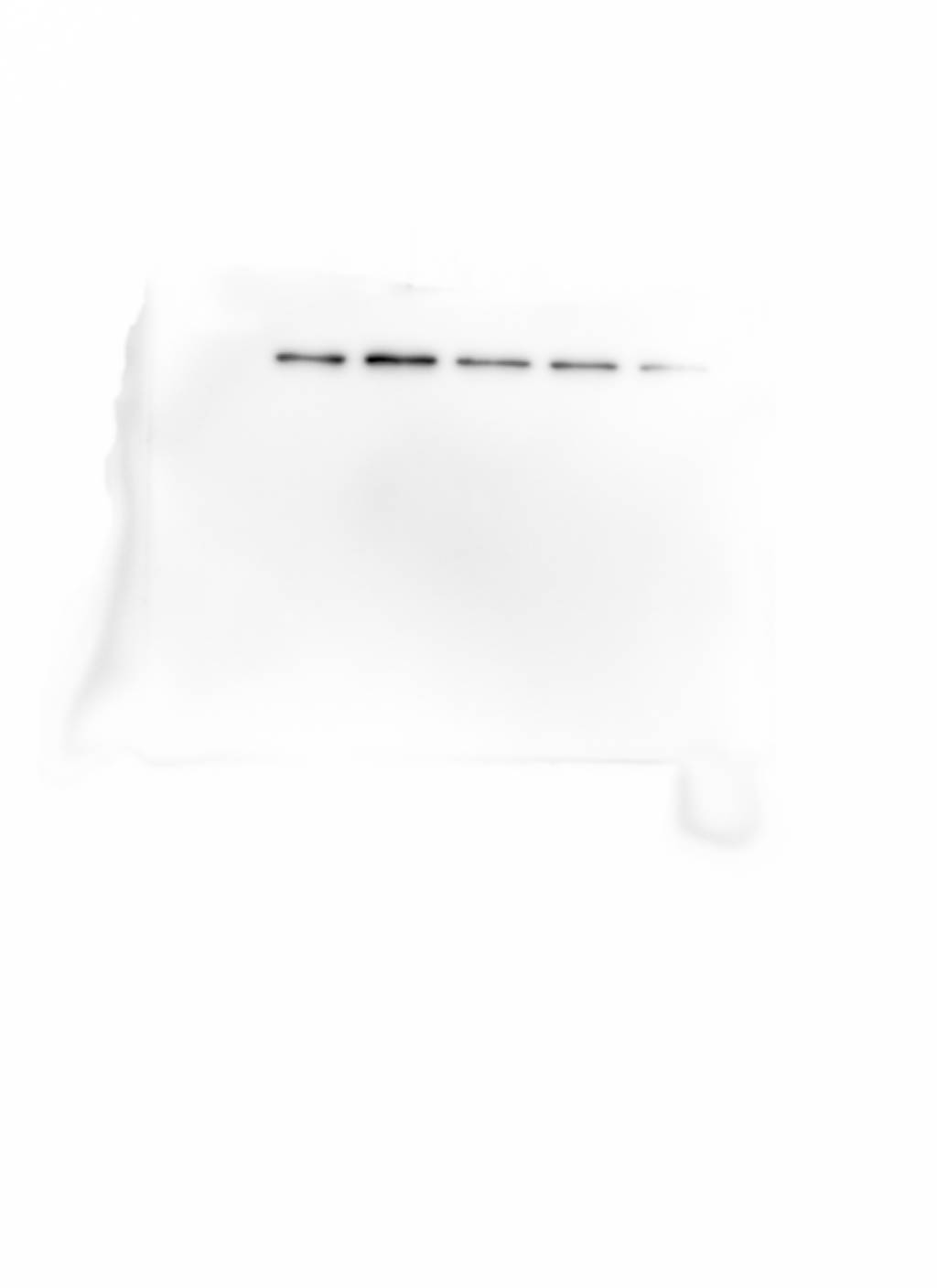

Supplement: Supplementary file 1 [file biomolecules-16-01059-s001.zip › File S1/Figure 6-8-11 Western blot original drawing/Figure 6 e vimentin/vimentin-1.tif]

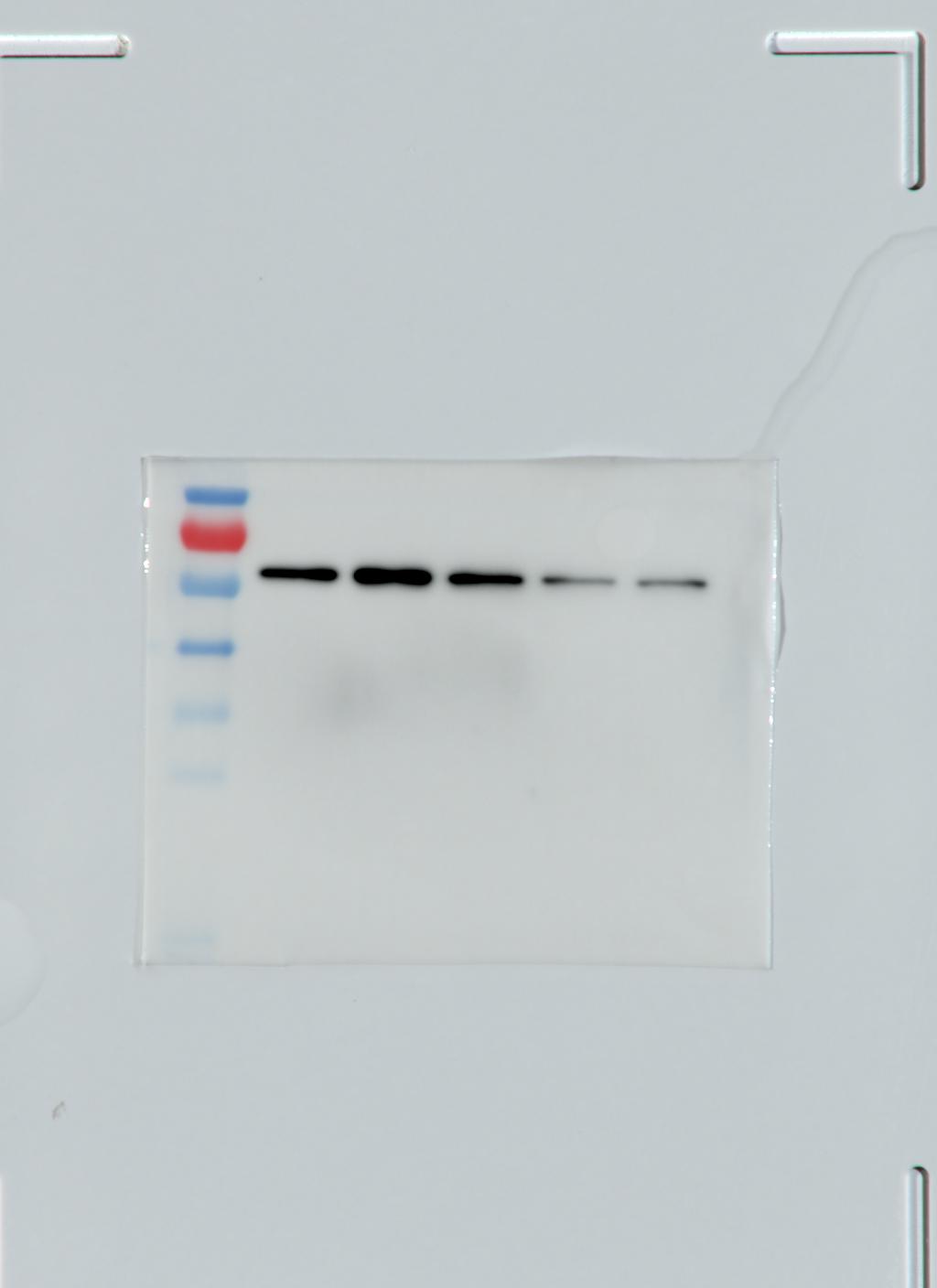

Supplement: Supplementary file 1 [file biomolecules-16-01059-s001.zip › File S1/Figure 6-8-11 Western blot original drawing/Figure 6 e vimentin/vimentin-2.jpg]

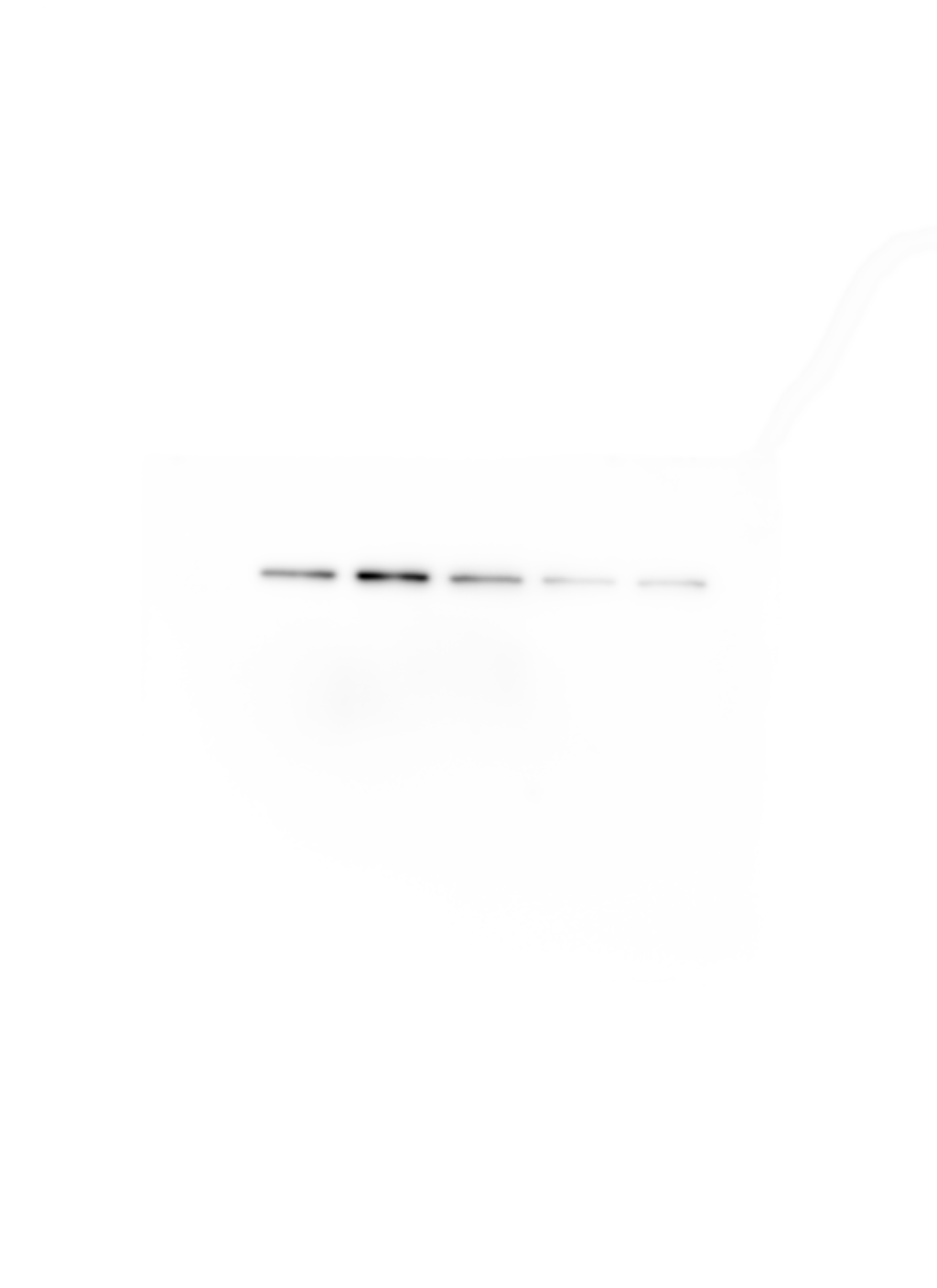

Supplement: Supplementary file 1 [file biomolecules-16-01059-s001.zip › File S1/Figure 6-8-11 Western blot original drawing/Figure 6 e vimentin/vimentin-2.tif]

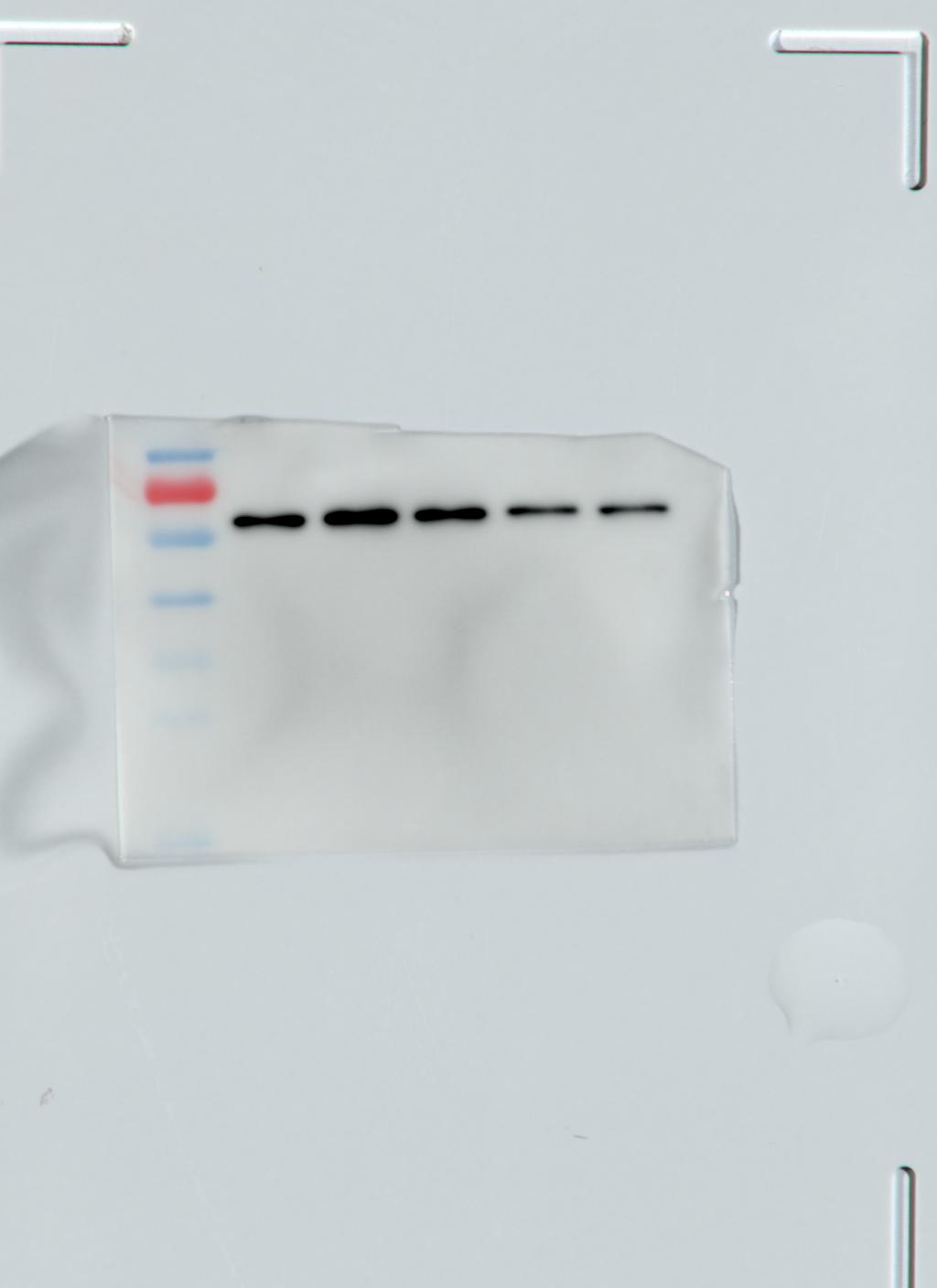

Supplement: Supplementary file 1 [file biomolecules-16-01059-s001.zip › File S1/Figure 6-8-11 Western blot original drawing/Figure 6 e vimentin/vimentin-3.jpg]

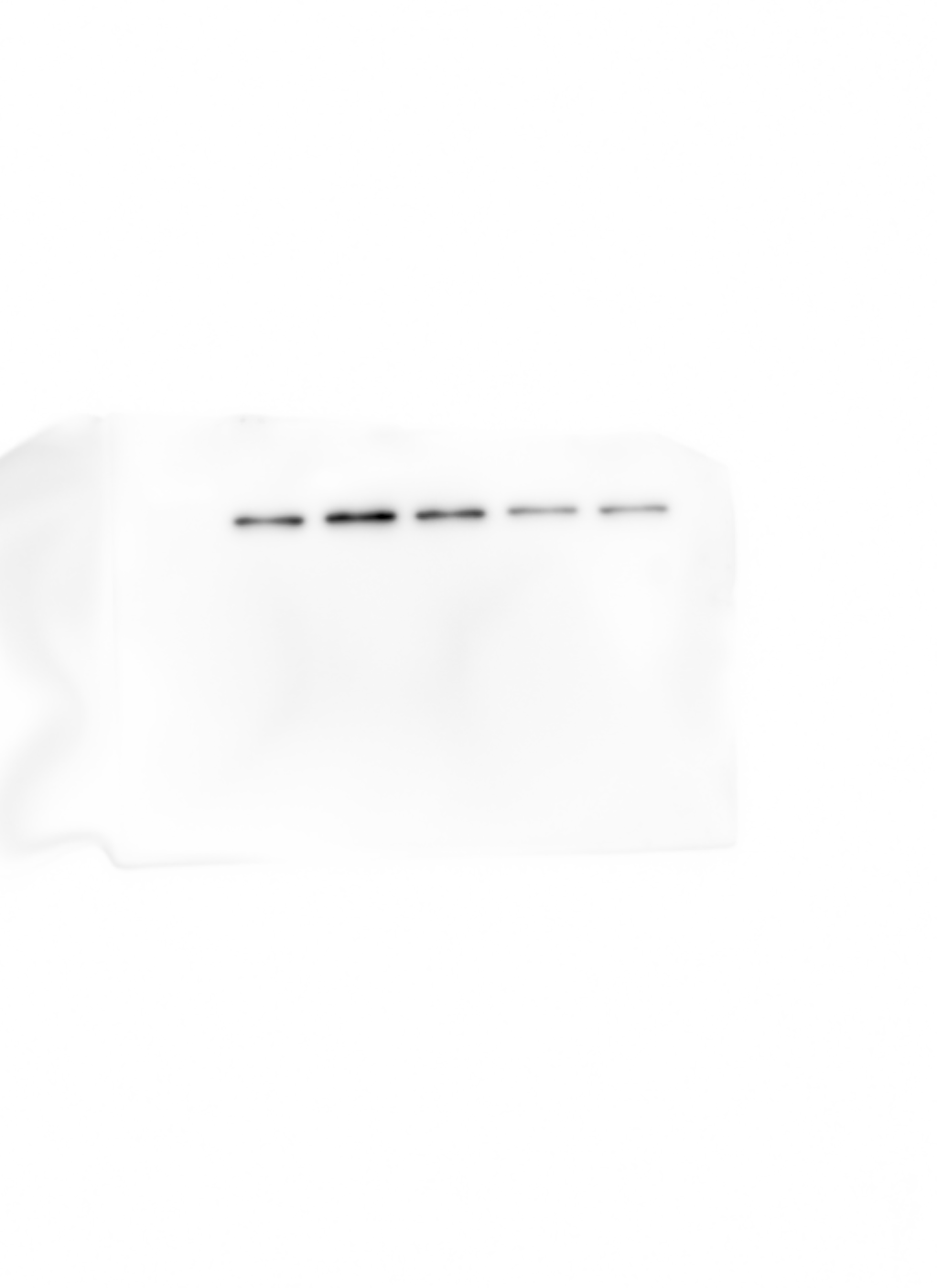

Supplement: Supplementary file 1 [file biomolecules-16-01059-s001.zip › File S1/Figure 6-8-11 Western blot original drawing/Figure 6 e vimentin/vimentin-3.tif]

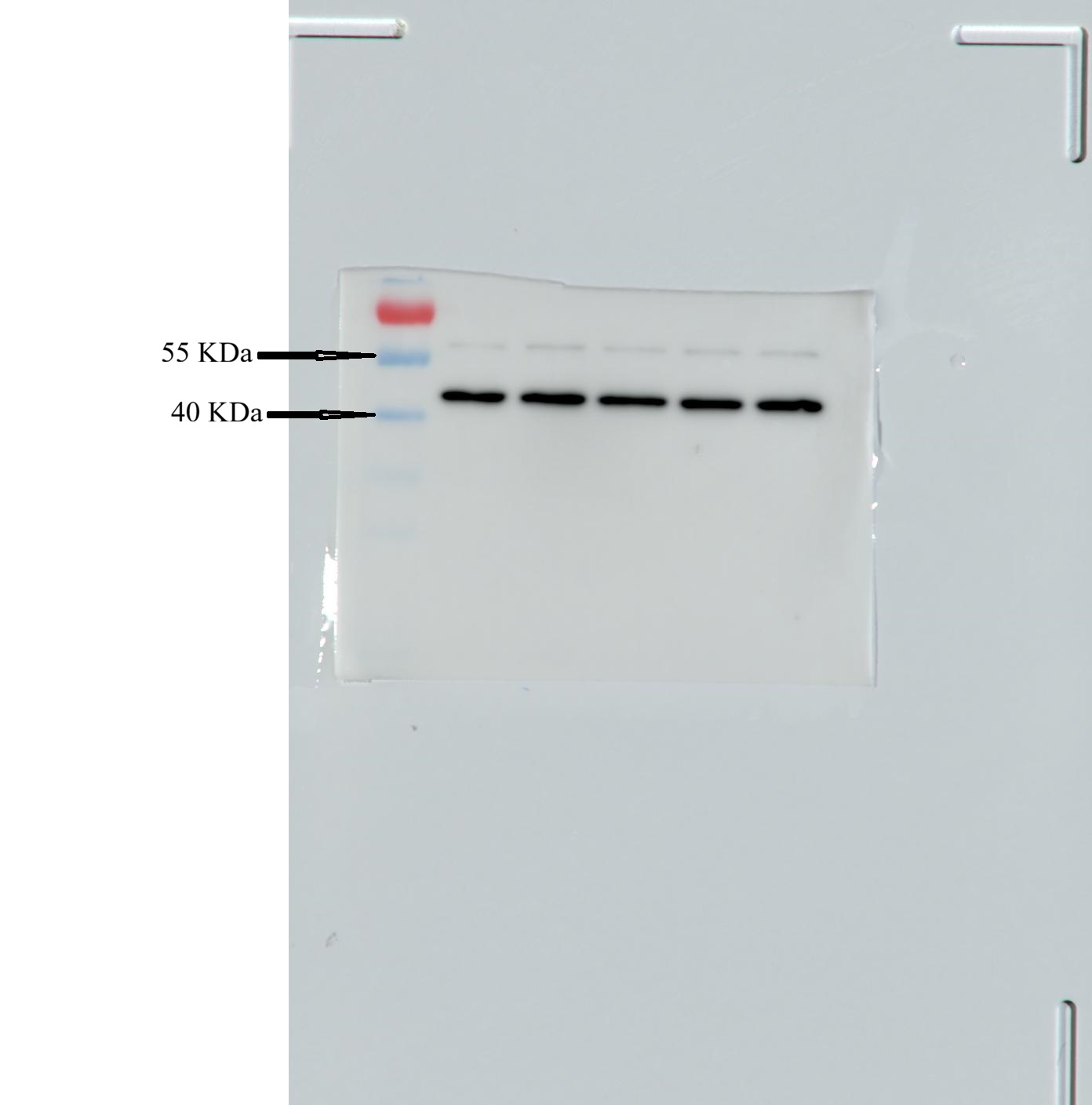

Supplement: Supplementary file 1 [file biomolecules-16-01059-s001.zip › File S1/Figure 6-8-11 Western blot original drawing/Figure 6 e vimentin/β-actin-1.jpg]

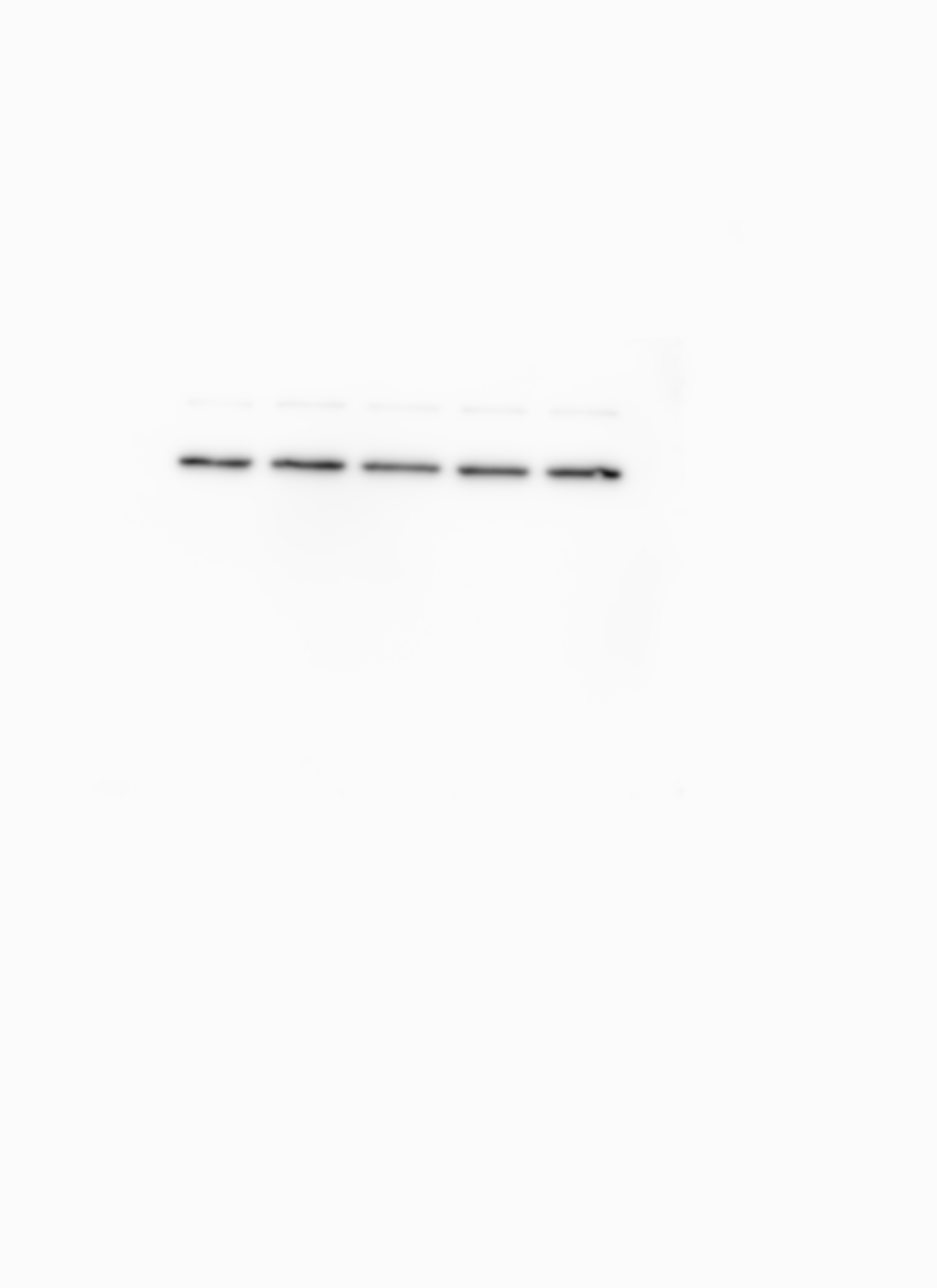

Supplement: Supplementary file 1 [file biomolecules-16-01059-s001.zip › File S1/Figure 6-8-11 Western blot original drawing/Figure 6 e vimentin/β-actin-1.tif]

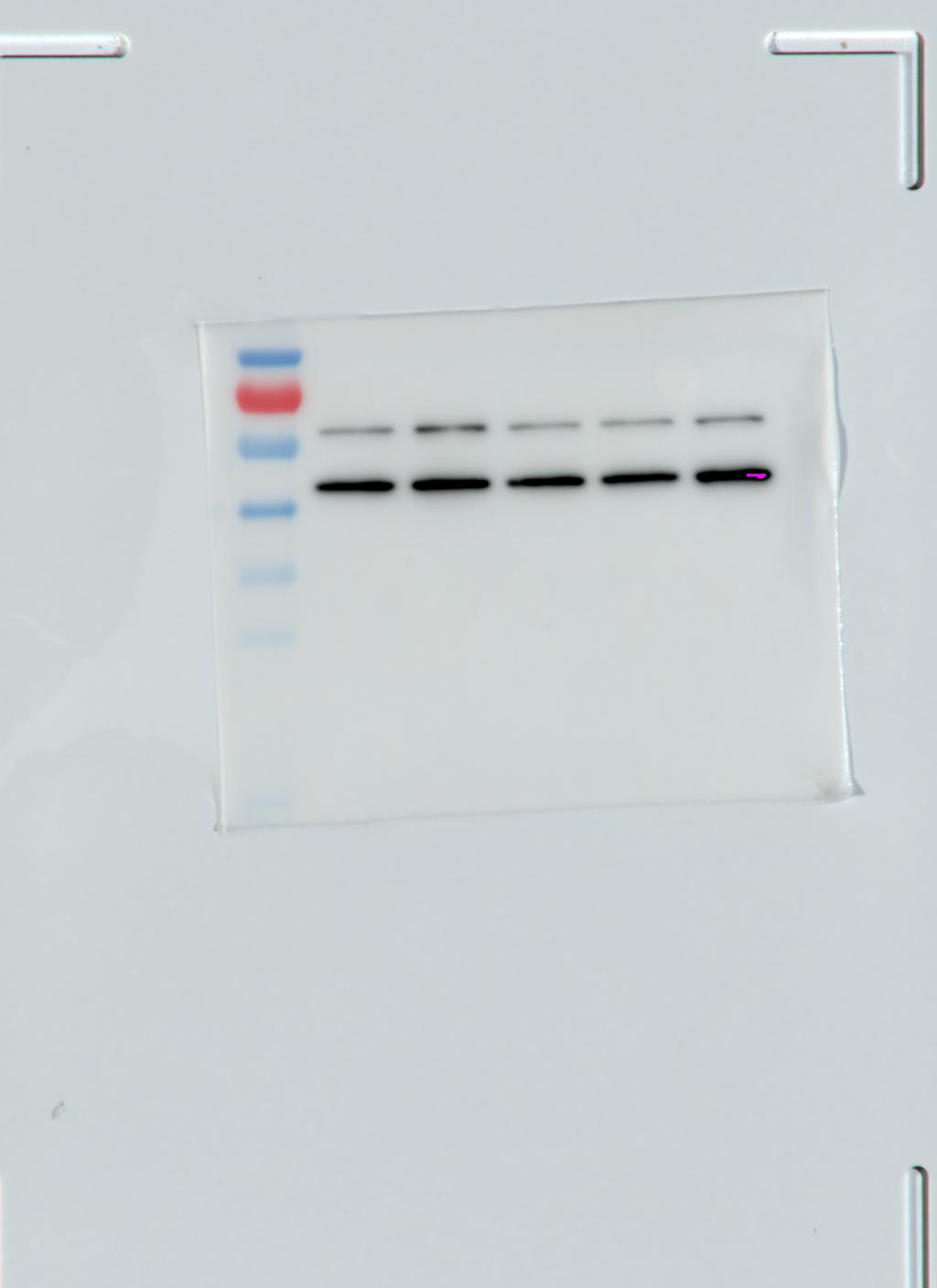

Supplement: Supplementary file 1 [file biomolecules-16-01059-s001.zip › File S1/Figure 6-8-11 Western blot original drawing/Figure 6 e vimentin/β-actin-2.jpg]

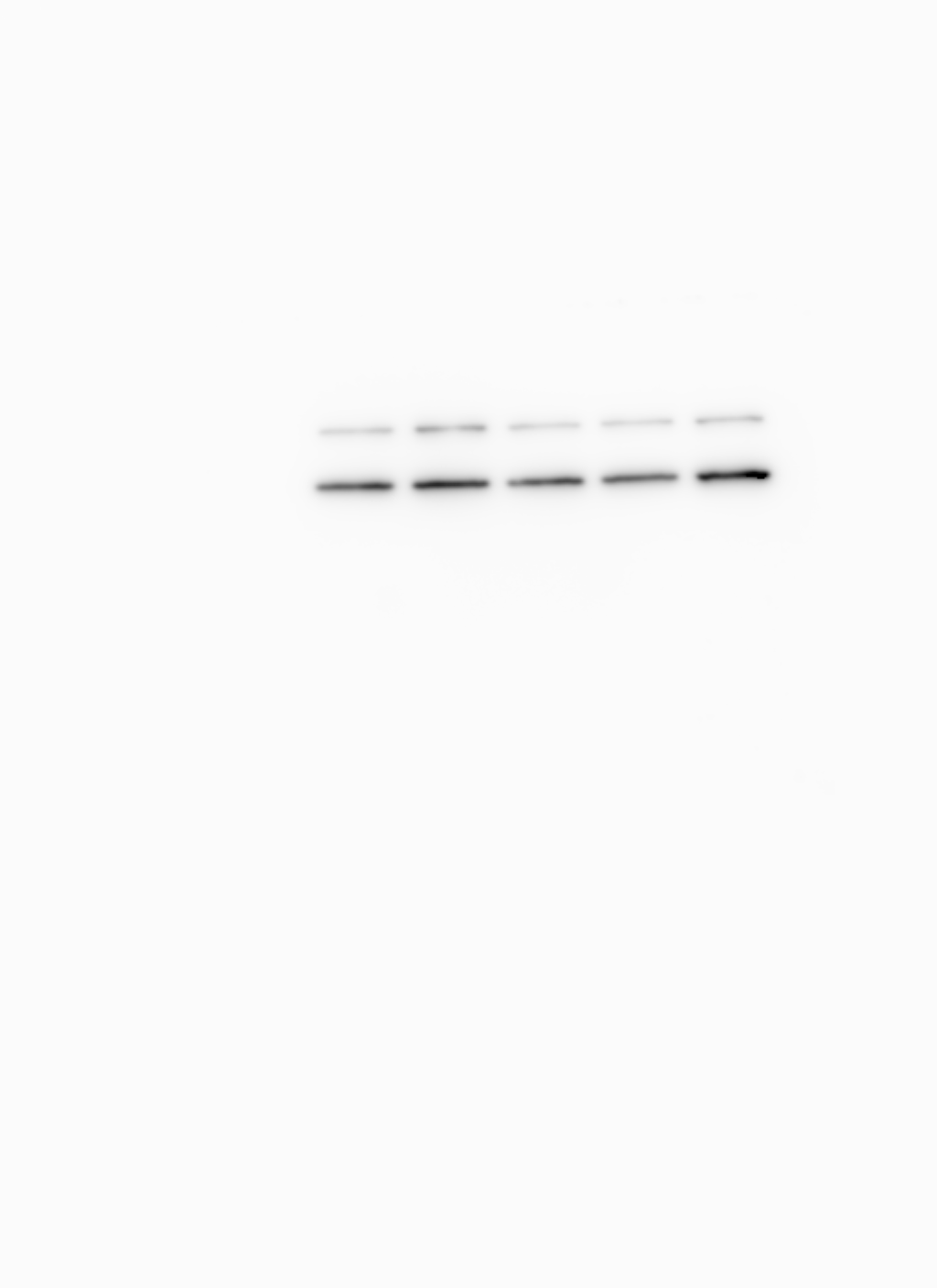

Supplement: Supplementary file 1 [file biomolecules-16-01059-s001.zip › File S1/Figure 6-8-11 Western blot original drawing/Figure 6 e vimentin/β-actin-2.tif]

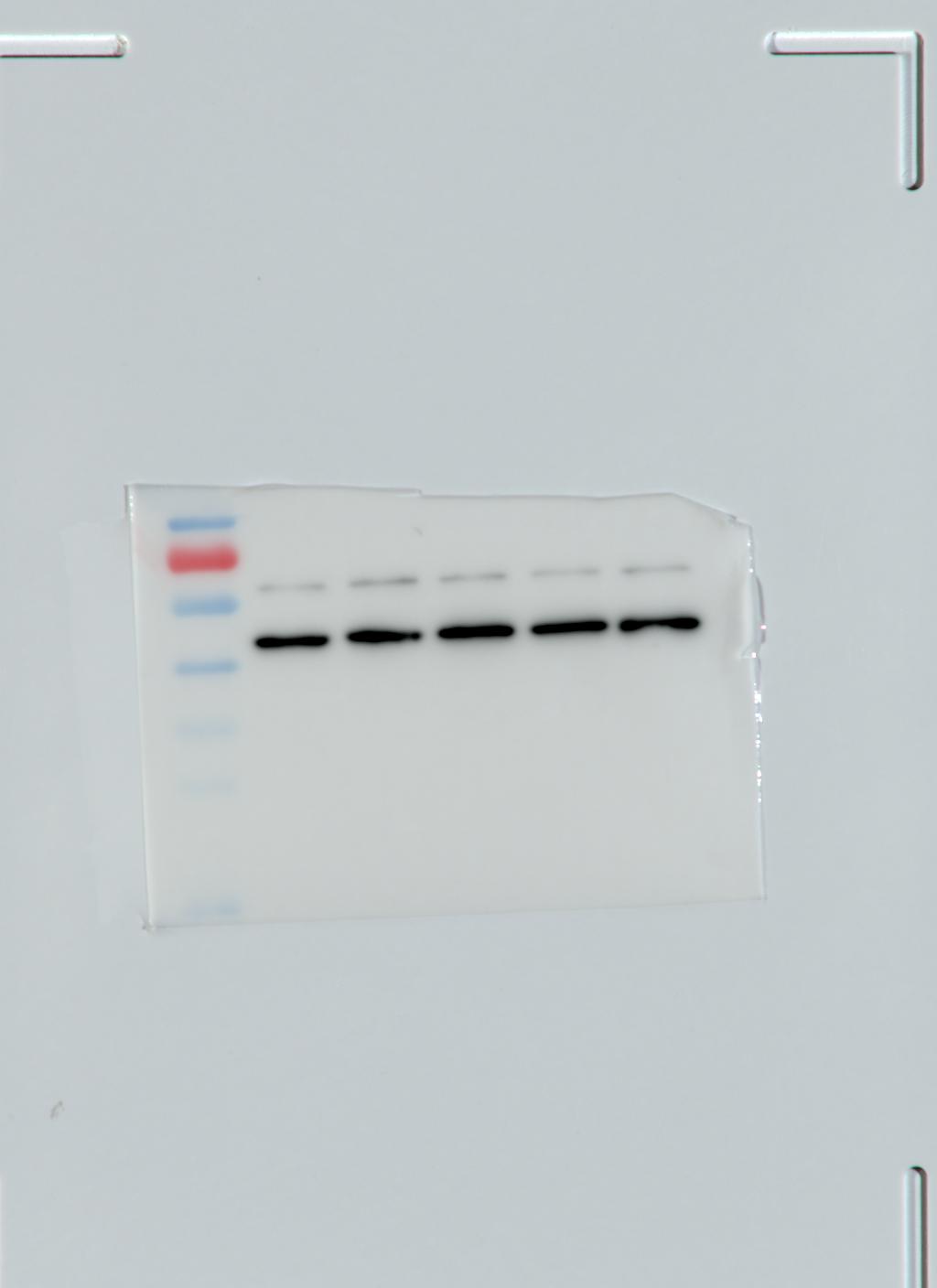

Supplement: Supplementary file 1 [file biomolecules-16-01059-s001.zip › File S1/Figure 6-8-11 Western blot original drawing/Figure 6 e vimentin/β-actin-3.jpg]

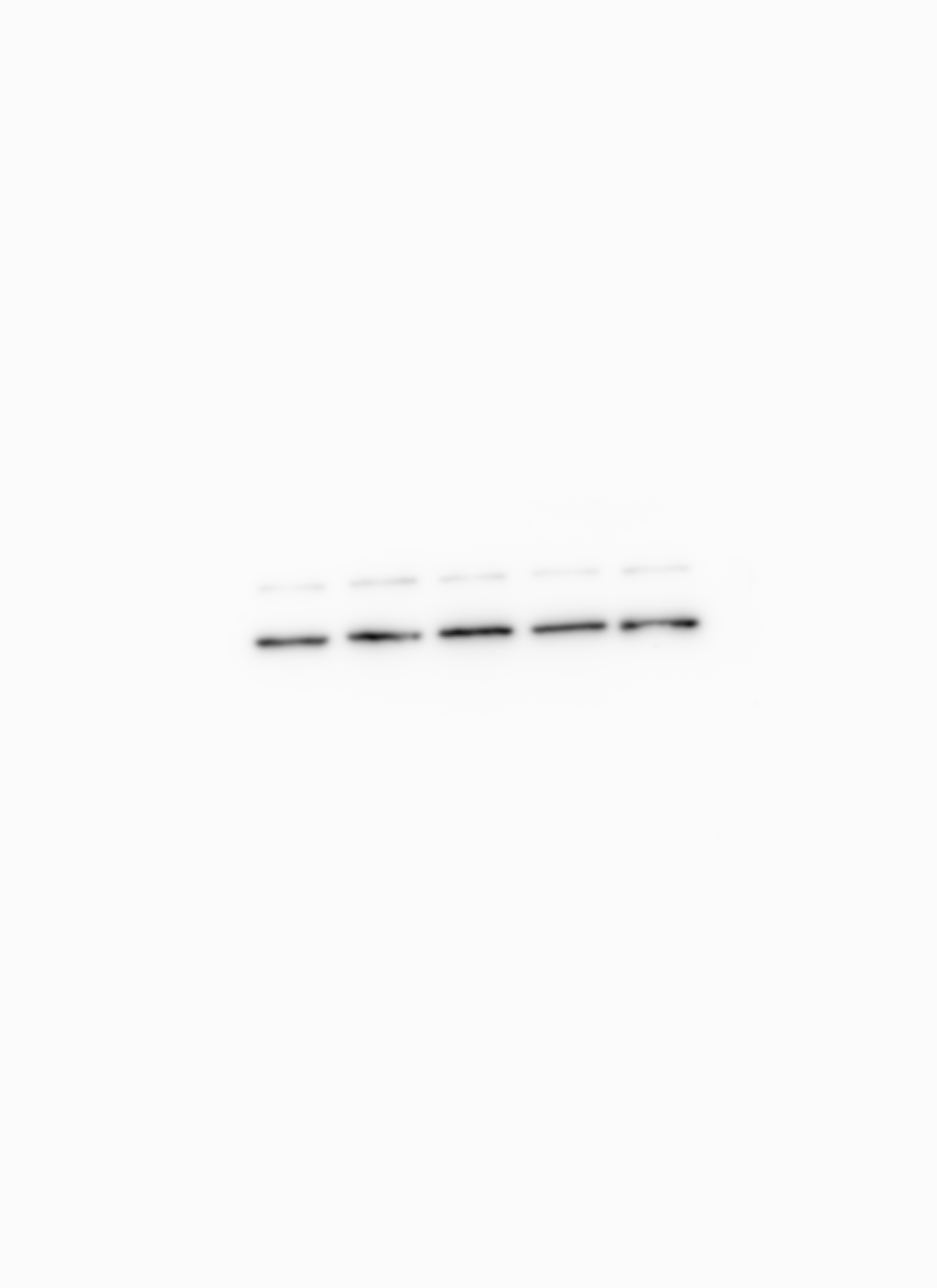

Supplement: Supplementary file 1 [file biomolecules-16-01059-s001.zip › File S1/Figure 6-8-11 Western blot original drawing/Figure 6 e vimentin/β-actin-3.tif]

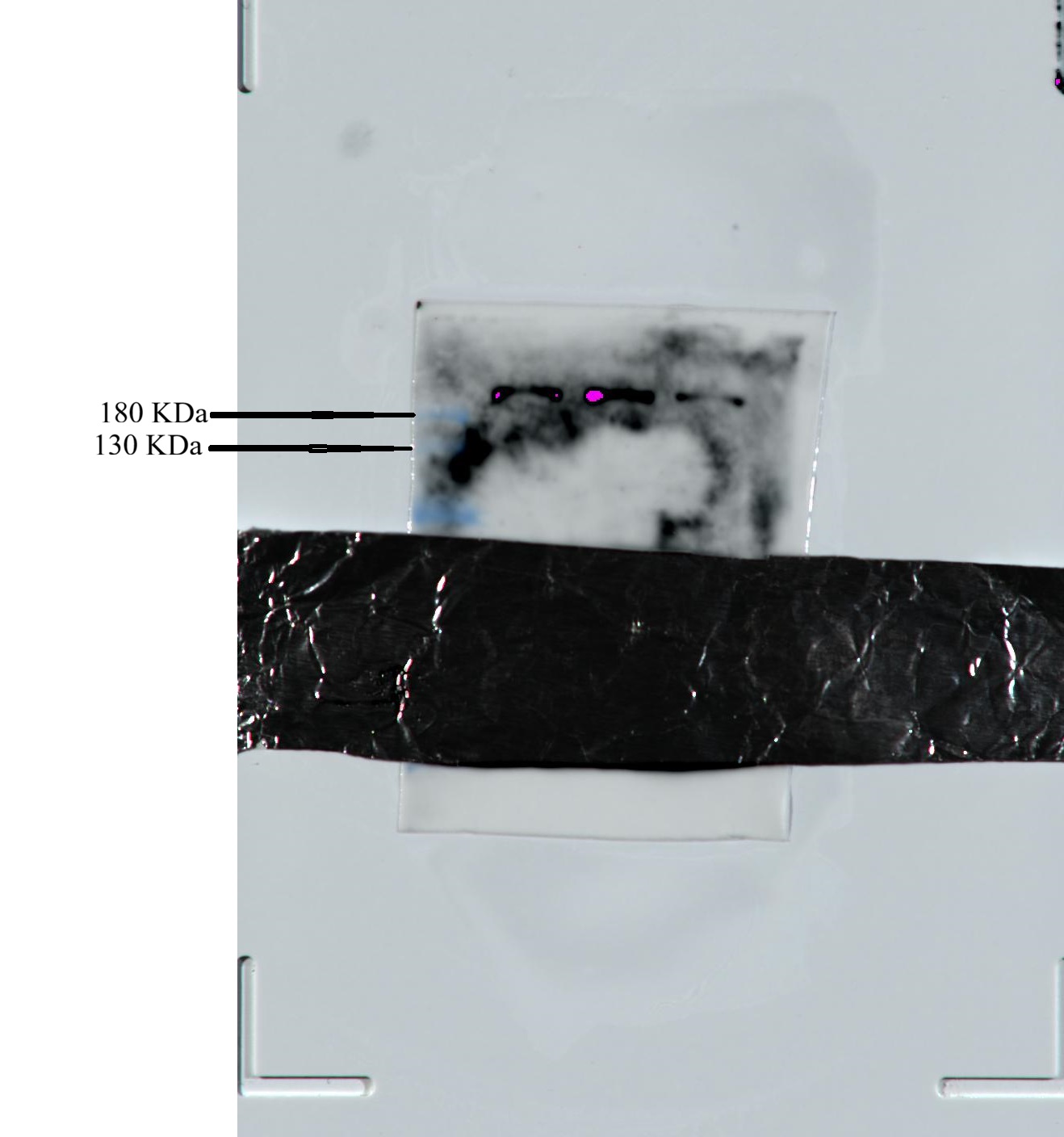

Supplement: Supplementary file 1 [file biomolecules-16-01059-s001.zip › File S1/Figure 6-8-11 Western blot original drawing/Figure 8 c Col4a6/COL4A6-1.jpg]

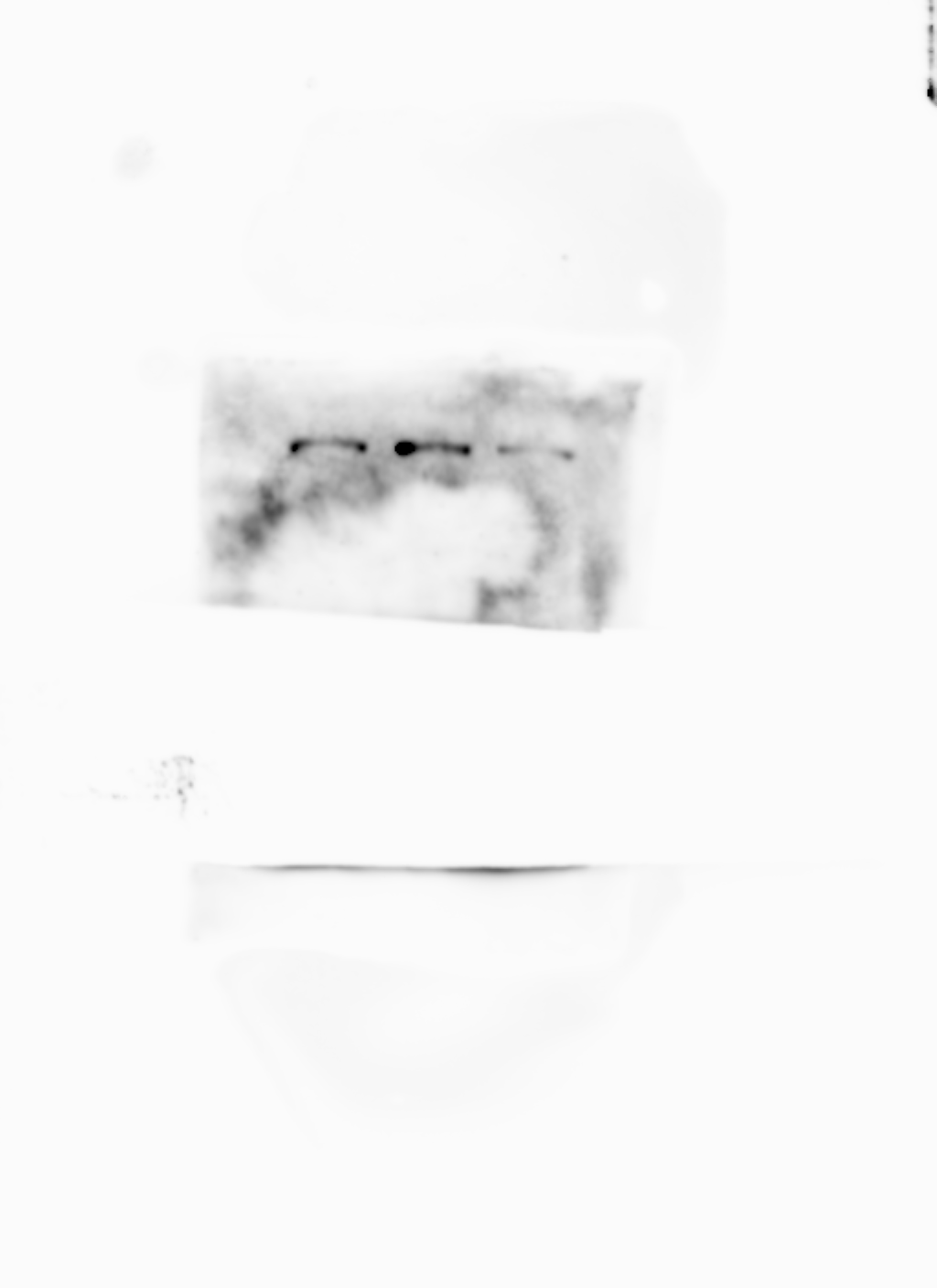

Supplement: Supplementary file 1 [file biomolecules-16-01059-s001.zip › File S1/Figure 6-8-11 Western blot original drawing/Figure 8 c Col4a6/COL4A6-1.tif]

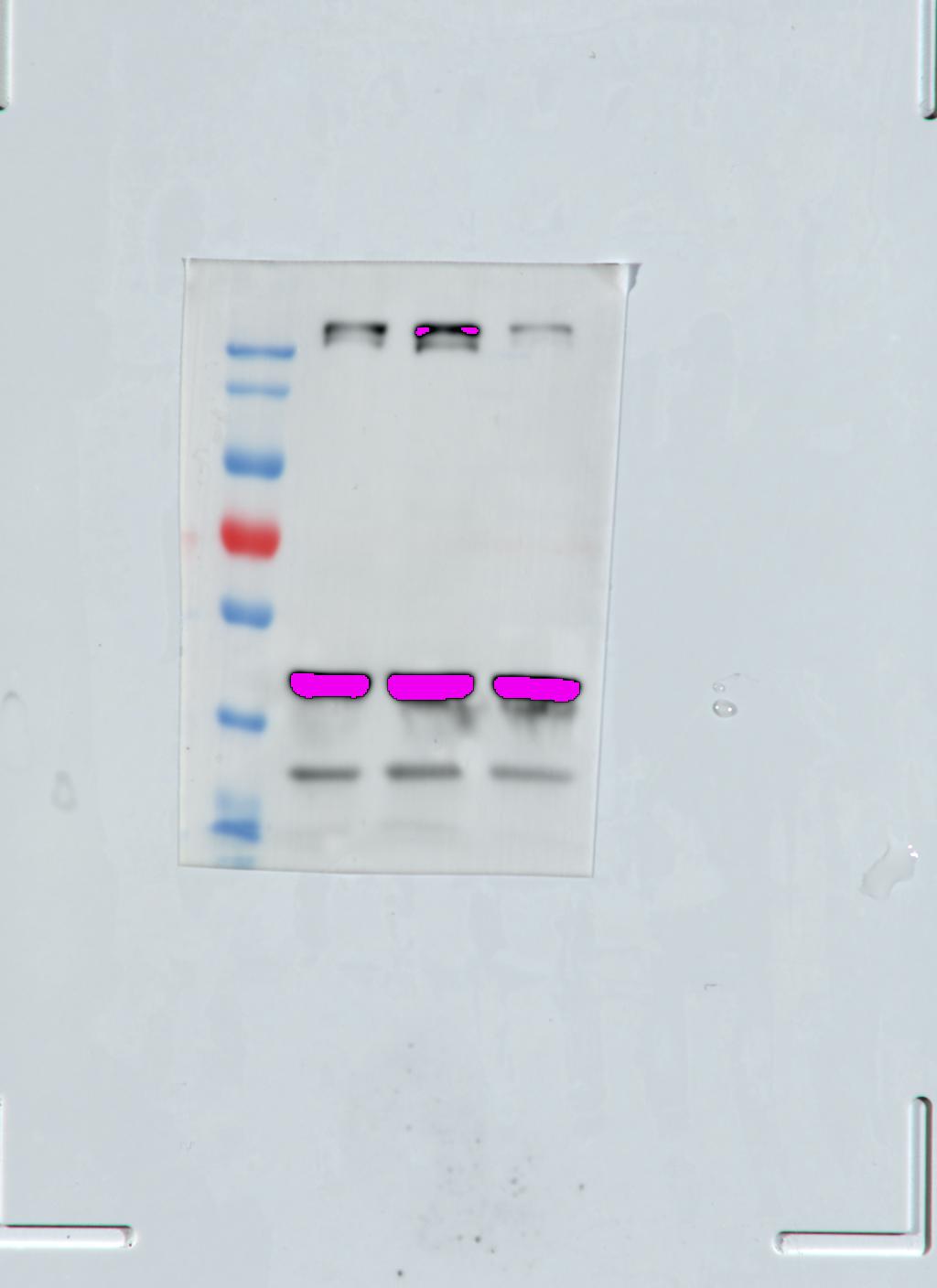

Supplement: Supplementary file 1 [file biomolecules-16-01059-s001.zip › File S1/Figure 6-8-11 Western blot original drawing/Figure 8 c Col4a6/COL4A6-β-actin-2.jpg]

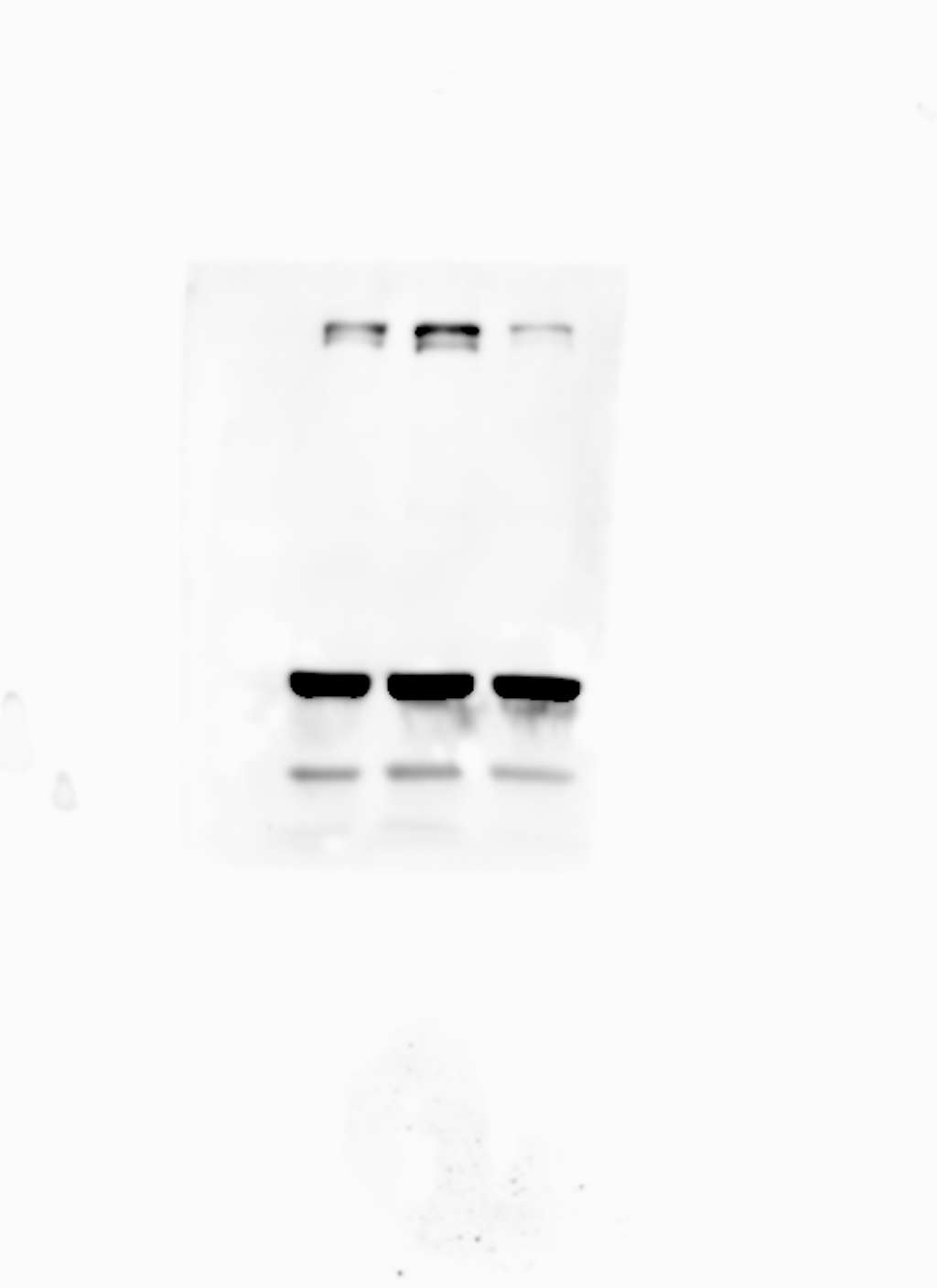

Supplement: Supplementary file 1 [file biomolecules-16-01059-s001.zip › File S1/Figure 6-8-11 Western blot original drawing/Figure 8 c Col4a6/COL4A6-β-actin-2.tif]

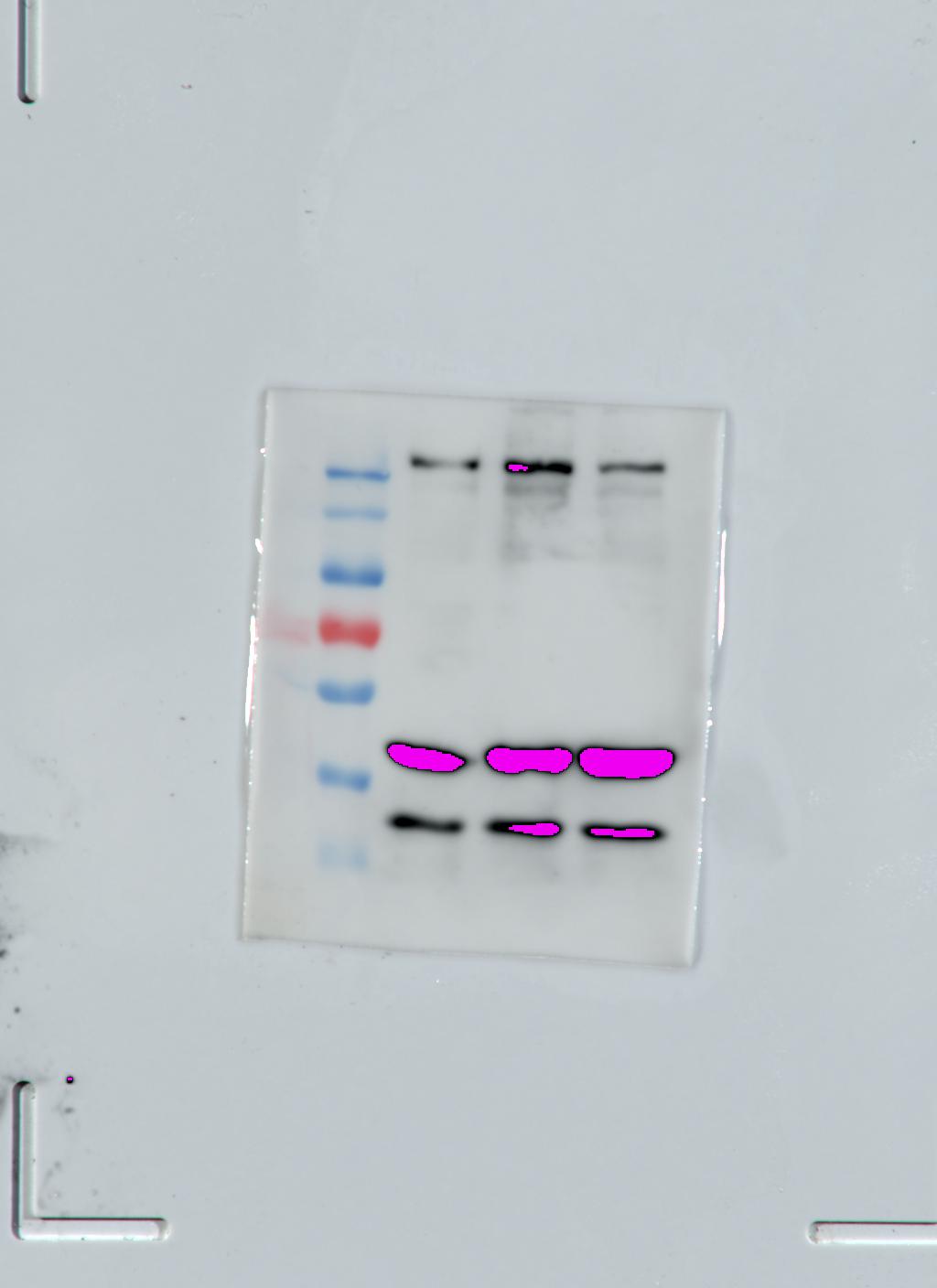

Supplement: Supplementary file 1 [file biomolecules-16-01059-s001.zip › File S1/Figure 6-8-11 Western blot original drawing/Figure 8 c Col4a6/COL4A6-β-actin-3.jpg]

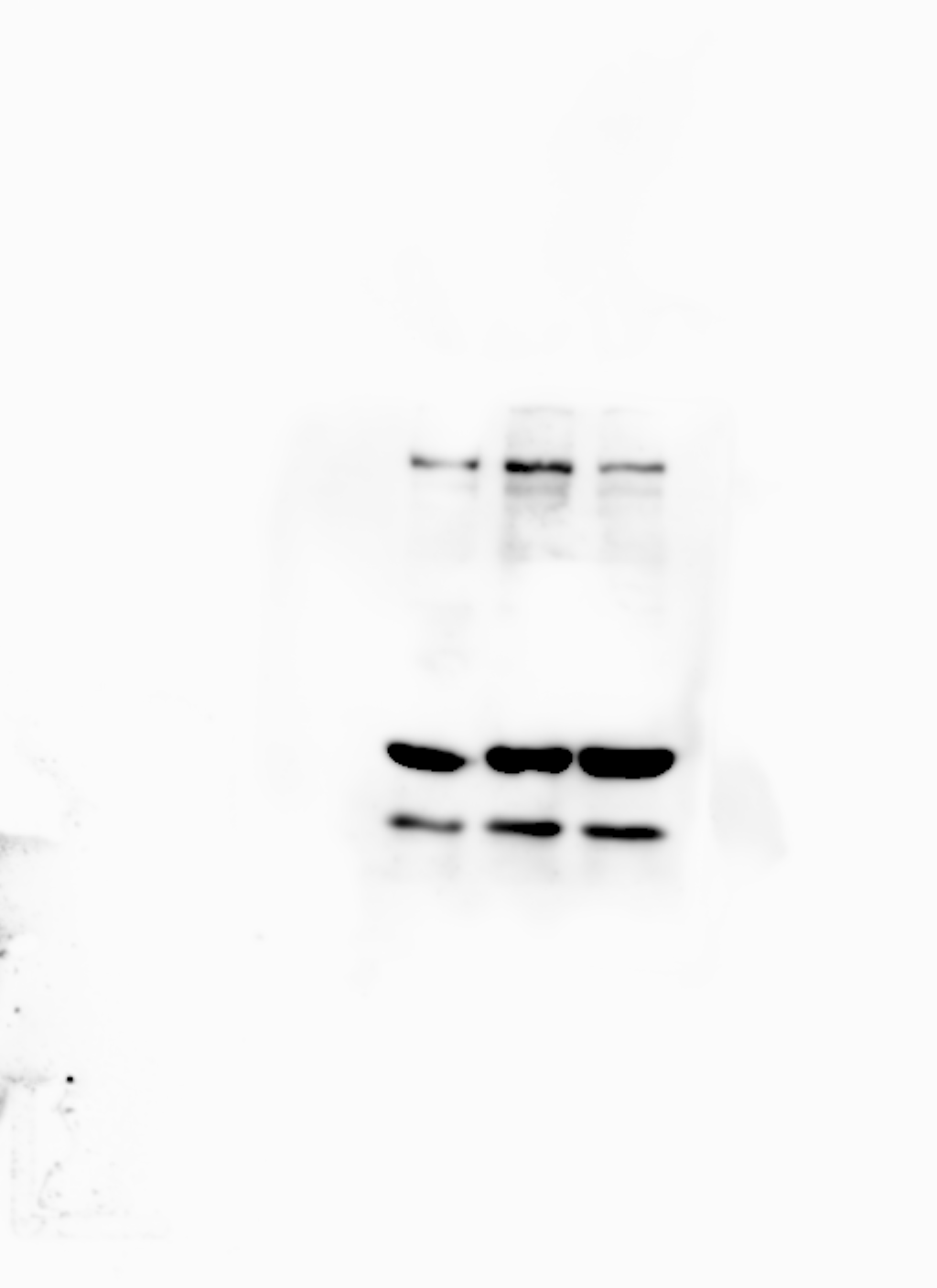

Supplement: Supplementary file 1 [file biomolecules-16-01059-s001.zip › File S1/Figure 6-8-11 Western blot original drawing/Figure 8 c Col4a6/COL4A6-β-actin-3.tif]

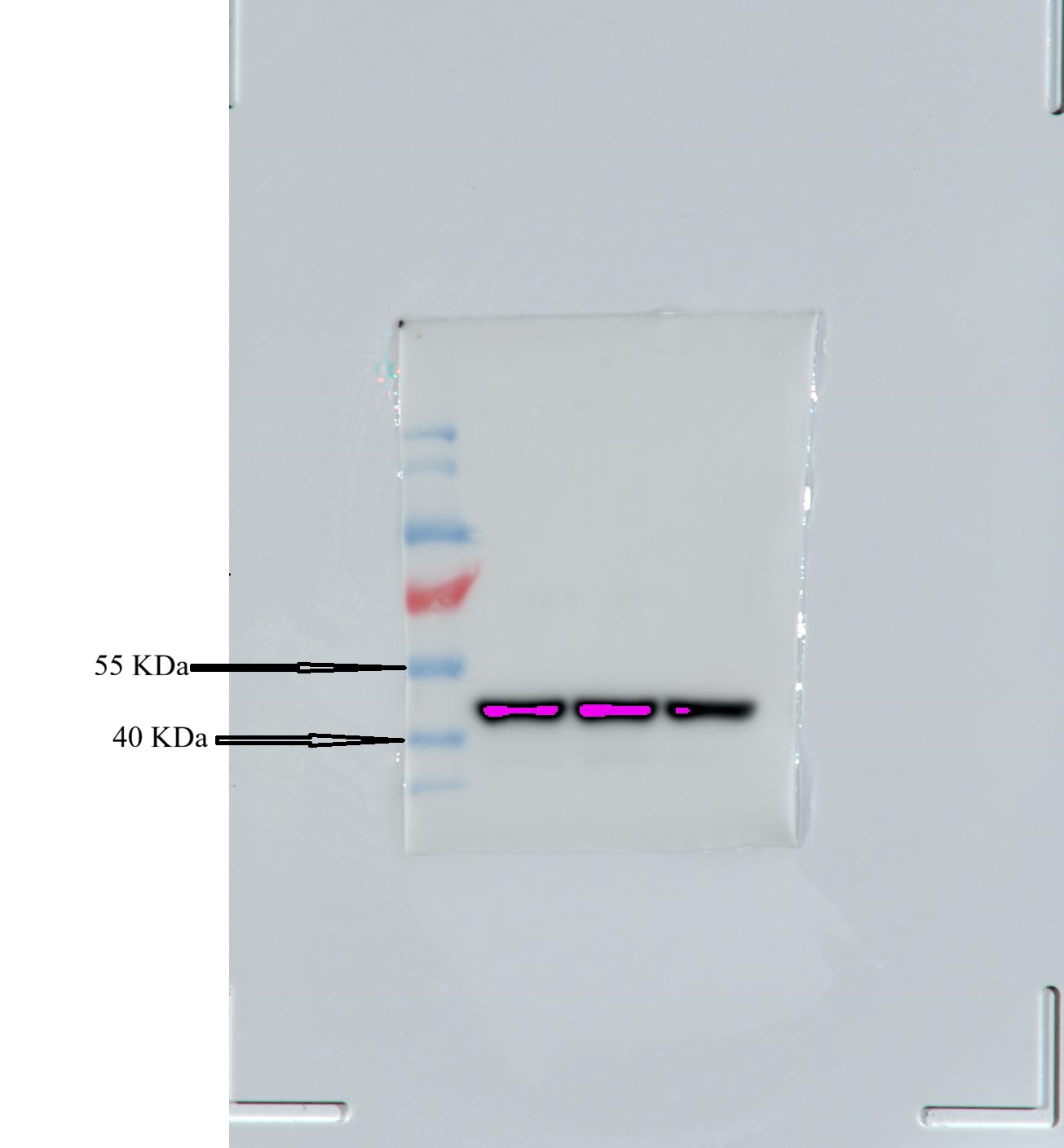

Supplement: Supplementary file 1 [file biomolecules-16-01059-s001.zip › File S1/Figure 6-8-11 Western blot original drawing/Figure 8 c Col4a6/β-actin-1.jpg]

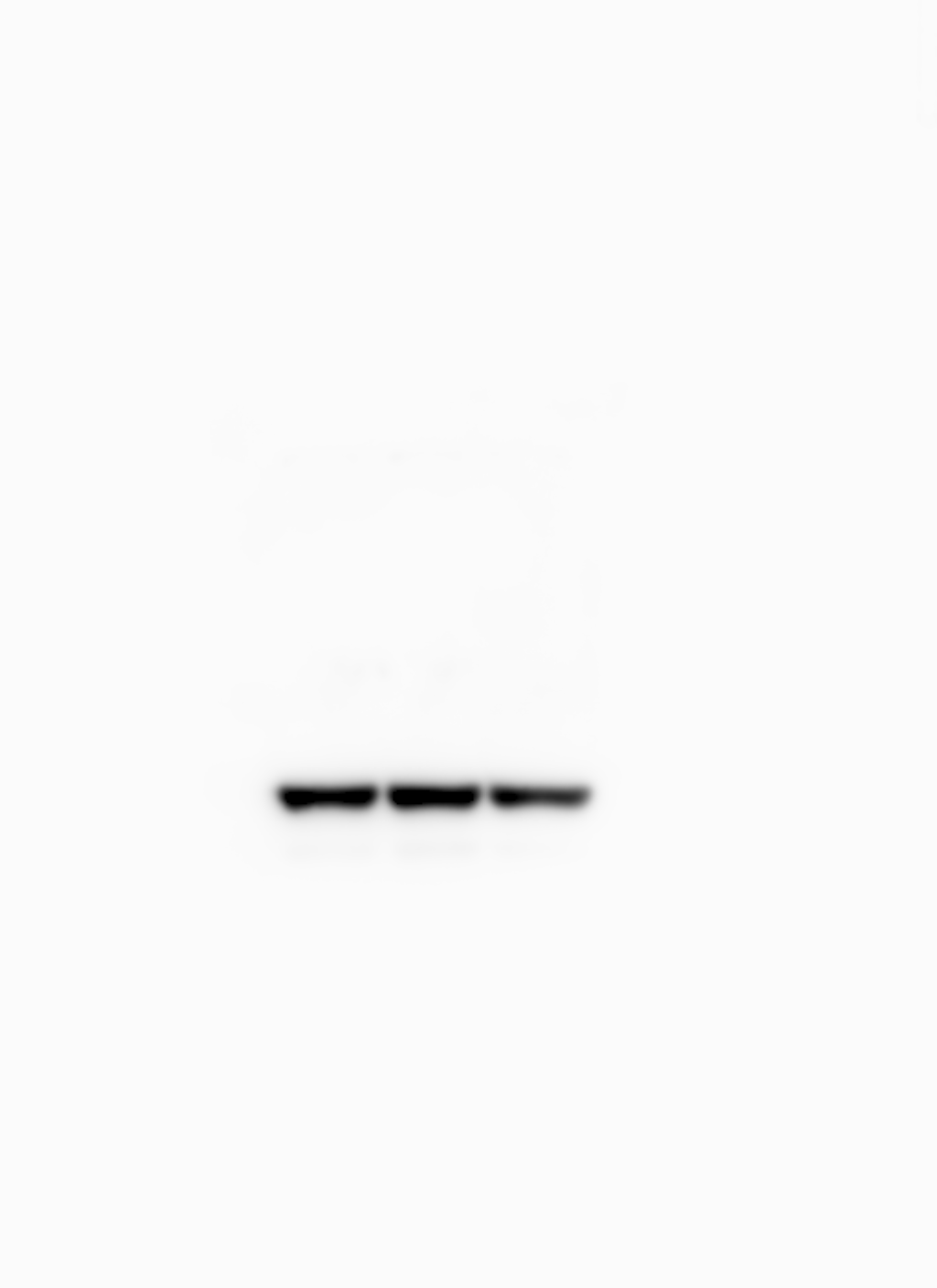

Supplement: Supplementary file 1 [file biomolecules-16-01059-s001.zip › File S1/Figure 6-8-11 Western blot original drawing/Figure 8 c Col4a6/β-actin-1.tif]

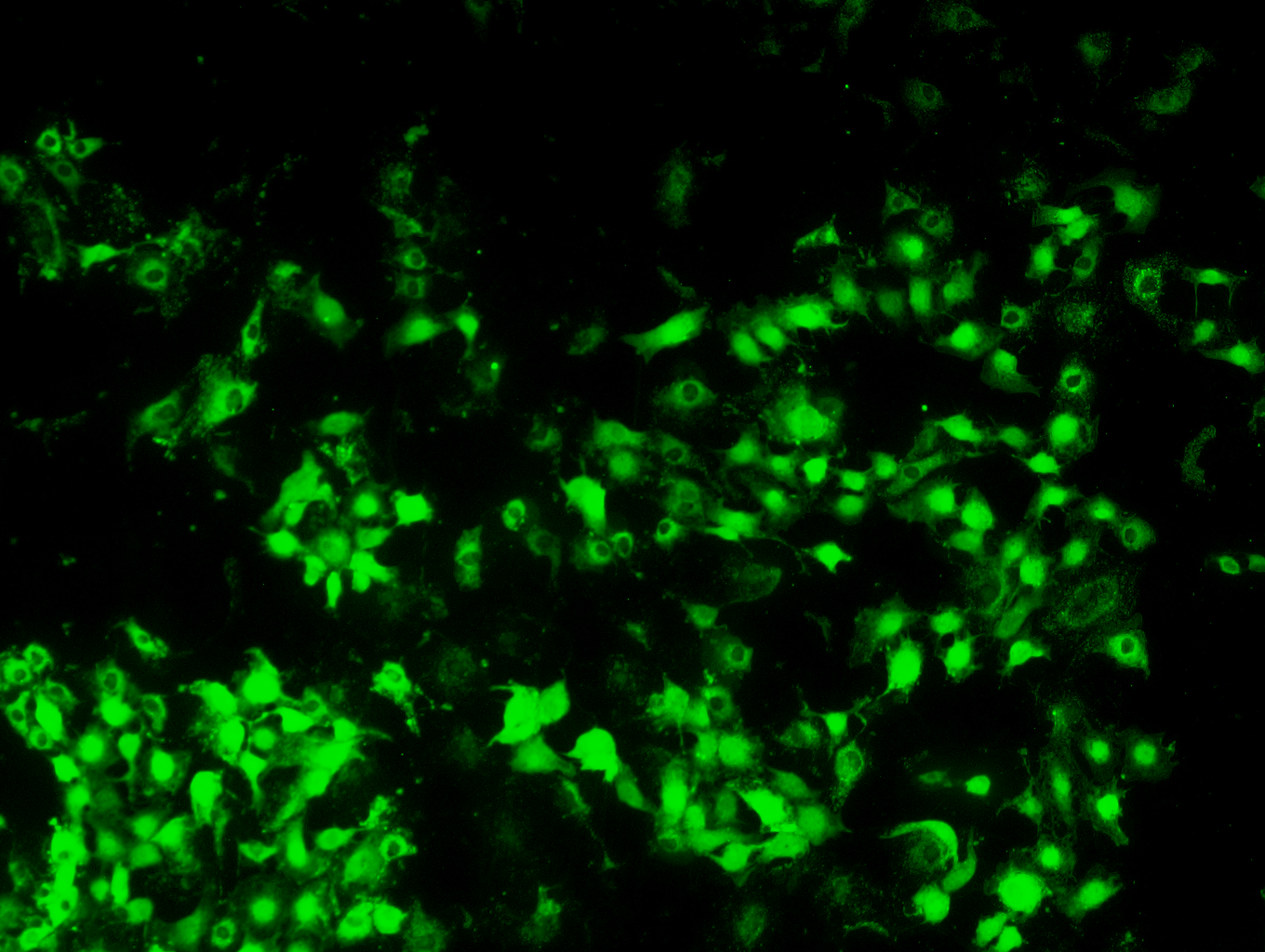

Supplement: Supplementary file 1 [file biomolecules-16-01059-s001.zip › File S1/Supplementary original experimental images-1/Figure 2c/CoCl2/1.tif]

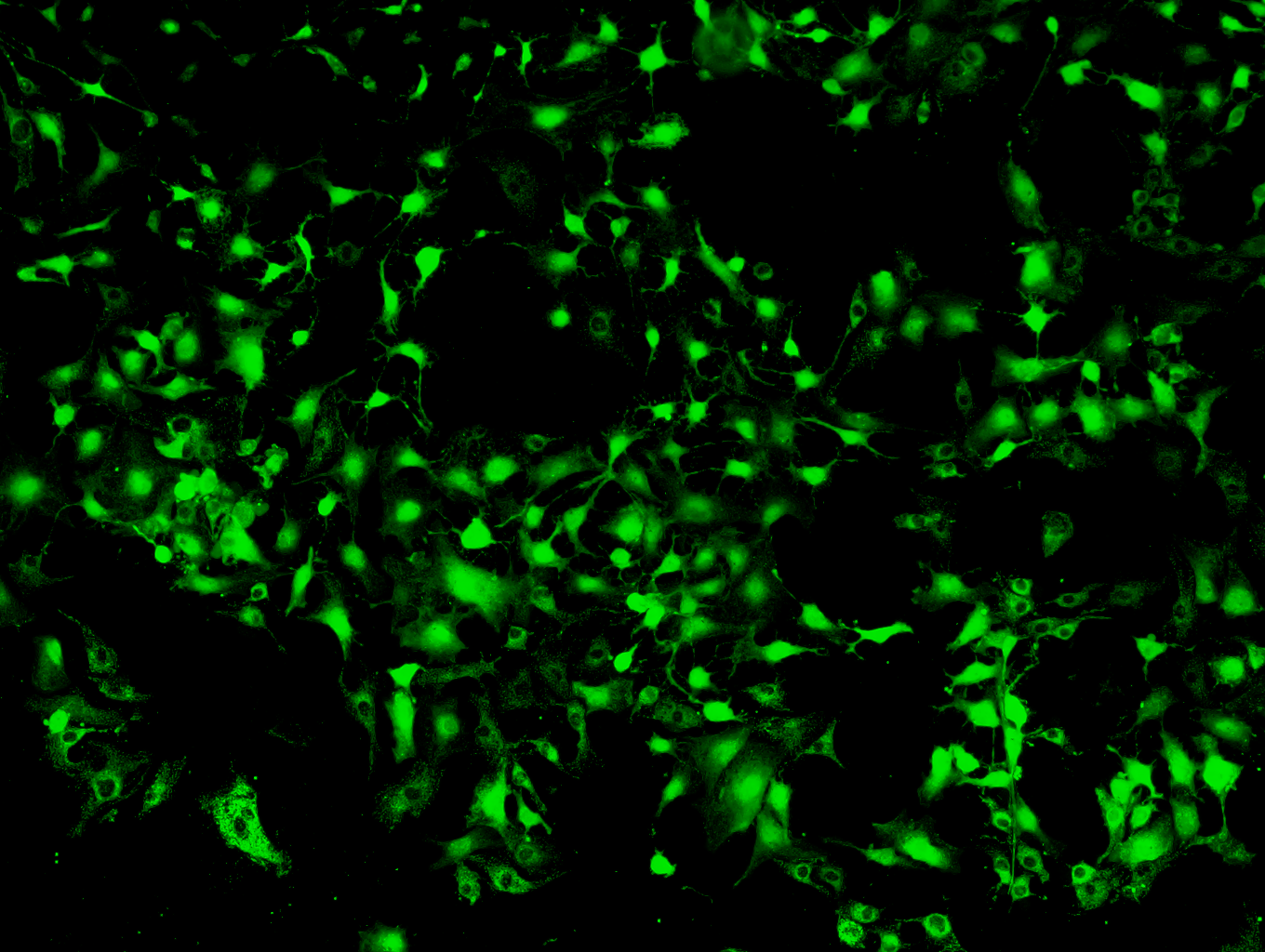

Supplement: Supplementary file 1 [file biomolecules-16-01059-s001.zip › File S1/Supplementary original experimental images-1/Figure 2c/CoCl2/2.tif]

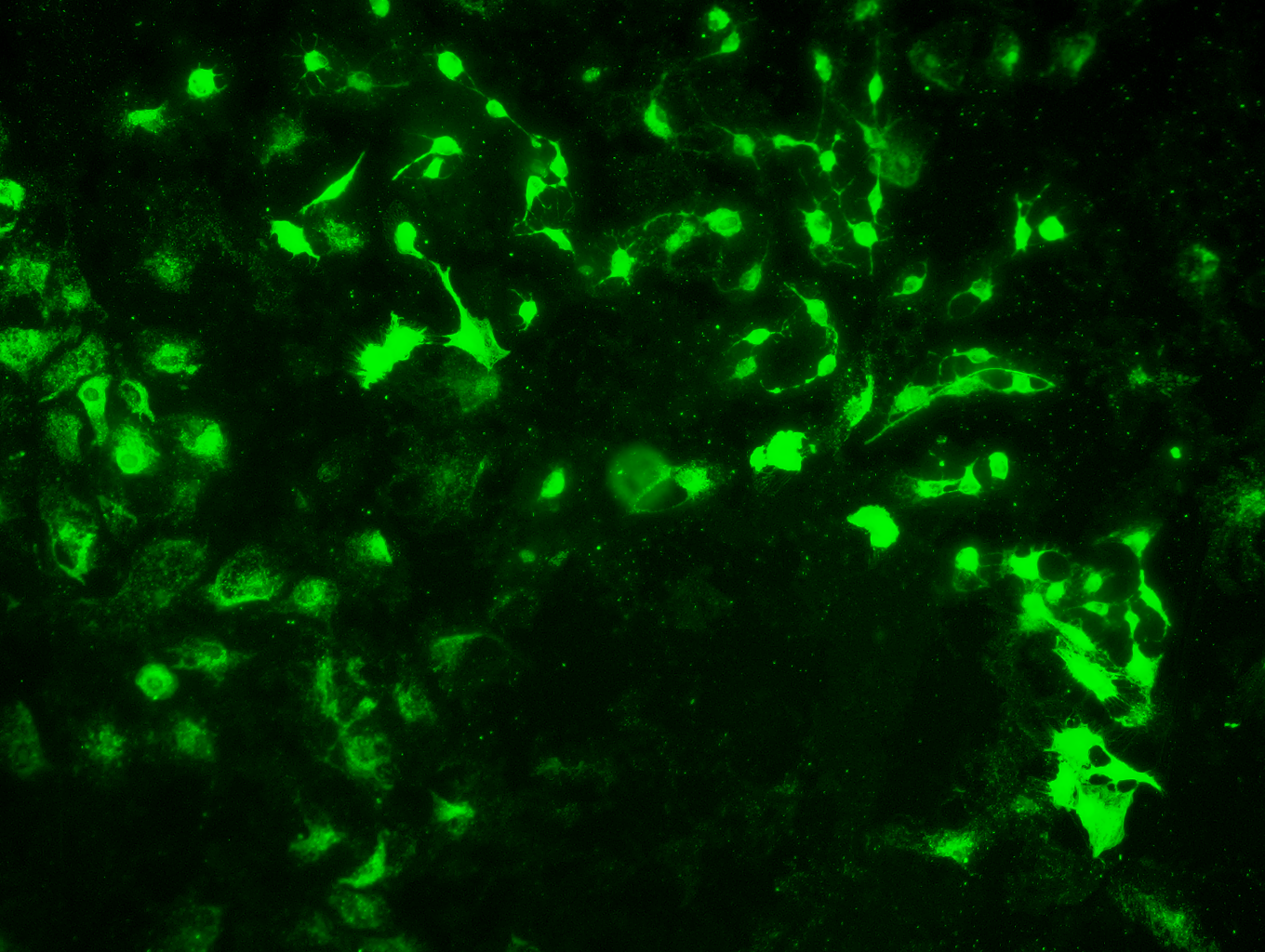

Supplement: Supplementary file 1 [file biomolecules-16-01059-s001.zip › File S1/Supplementary original experimental images-1/Figure 2c/CoCl2/3.tif]

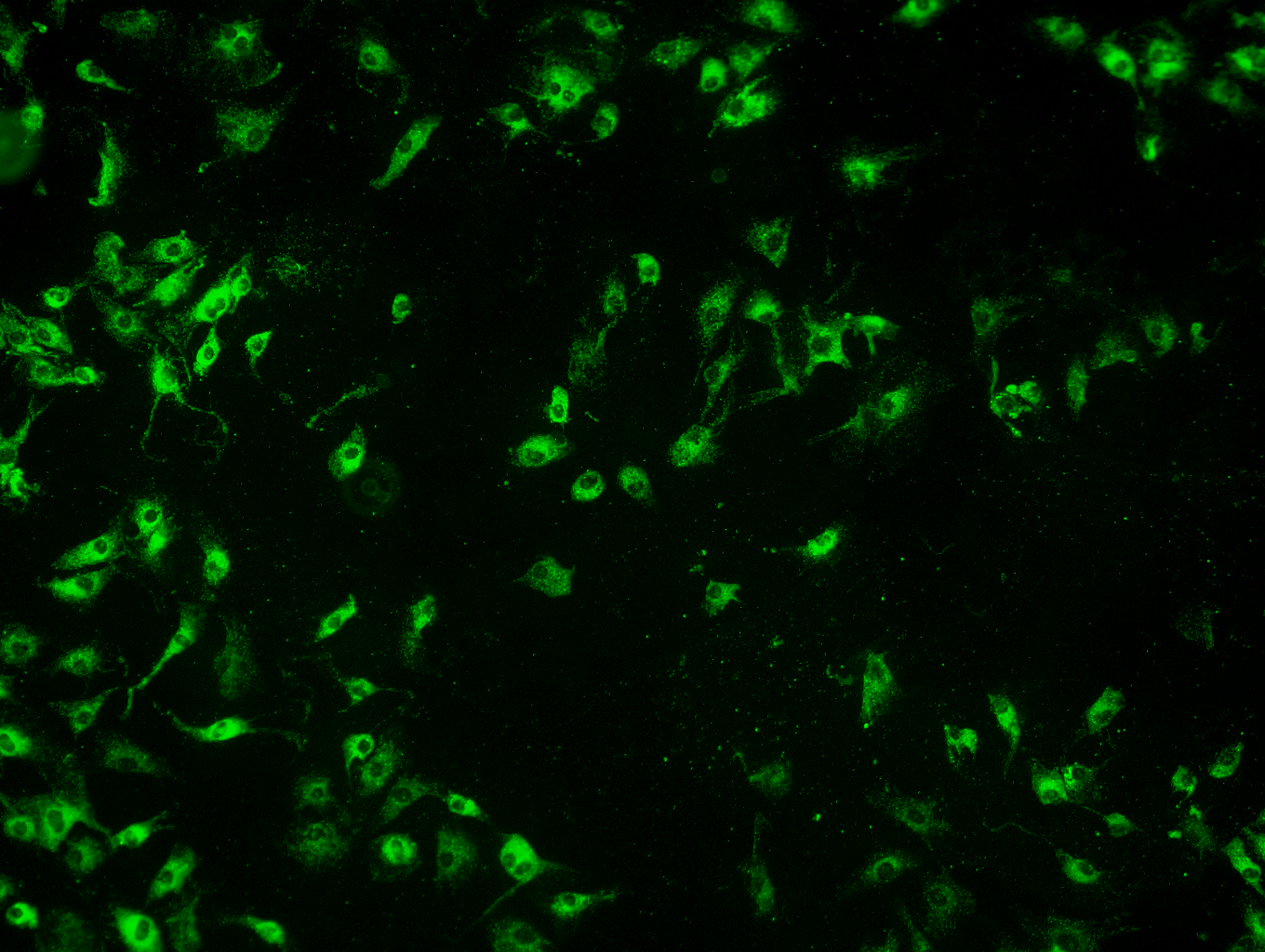

Supplement: Supplementary file 1 [file biomolecules-16-01059-s001.zip › File S1/Supplementary original experimental images-1/Figure 2c/CoCl2+BEL(20μmolL )/1.tif]

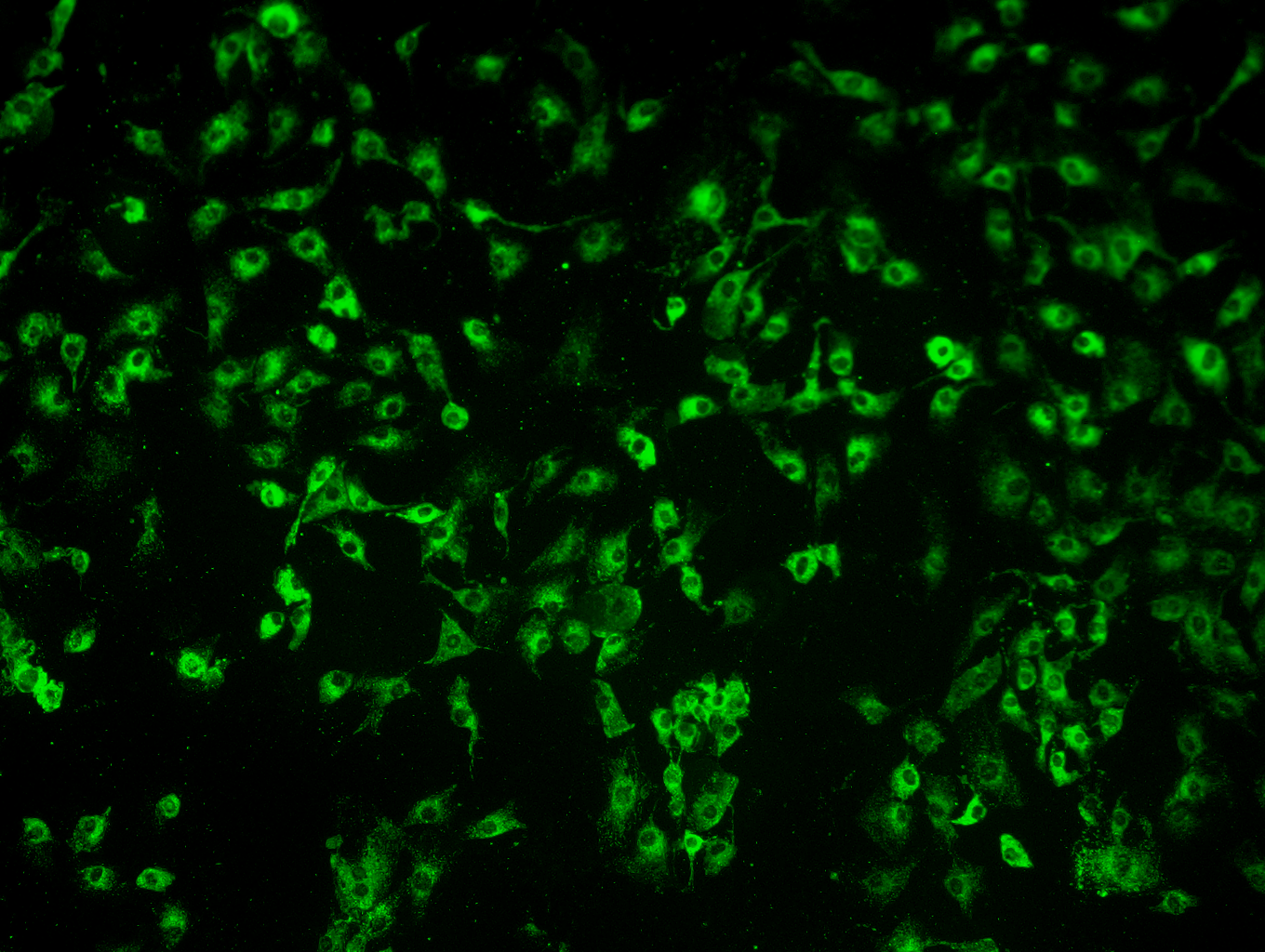

Supplement: Supplementary file 1 [file biomolecules-16-01059-s001.zip › File S1/Supplementary original experimental images-1/Figure 2c/CoCl2+BEL(20μmolL )/2.tif]

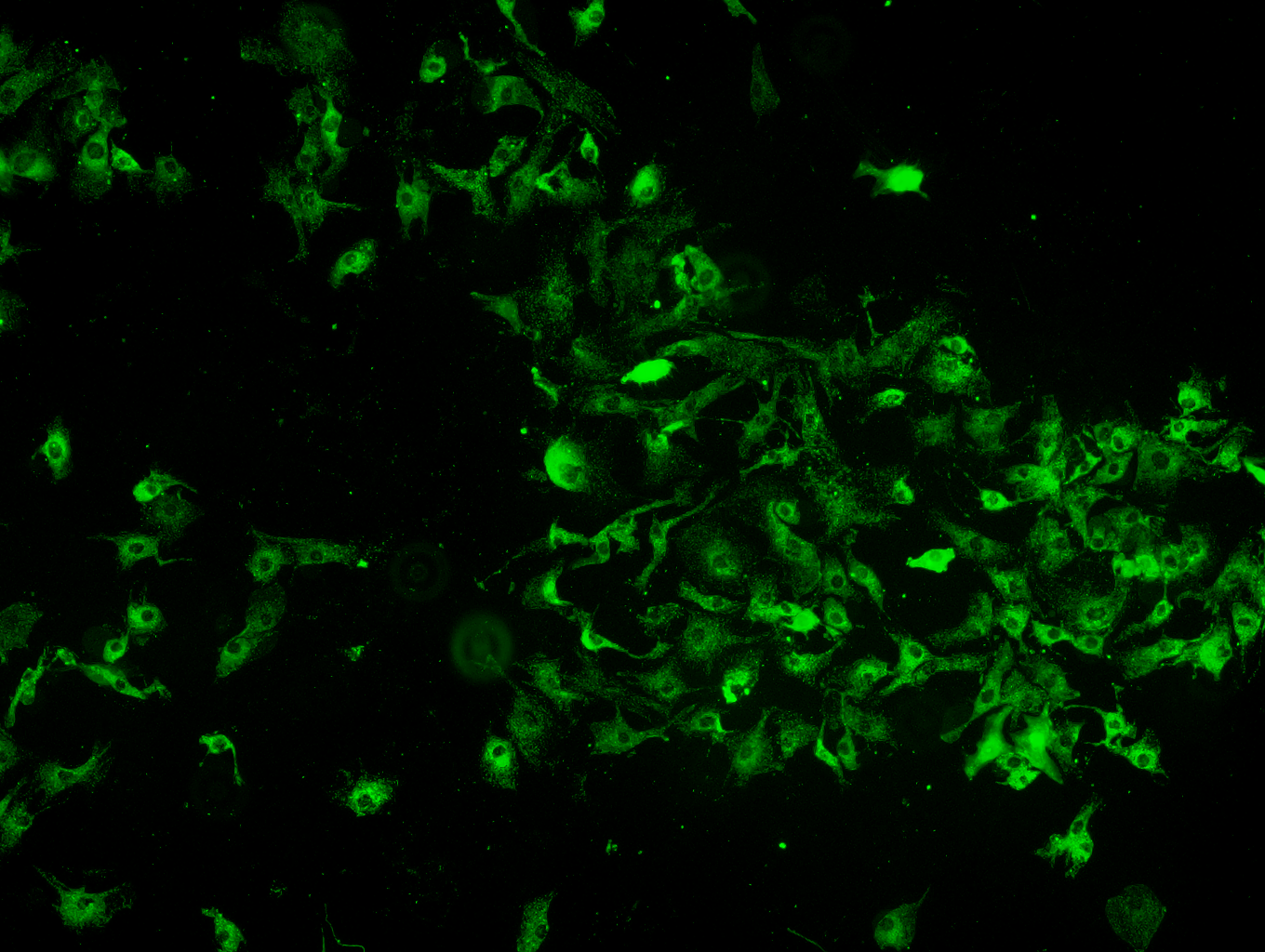

Supplement: Supplementary file 1 [file biomolecules-16-01059-s001.zip › File S1/Supplementary original experimental images-1/Figure 2c/CoCl2+BEL(20μmolL )/3.tif]

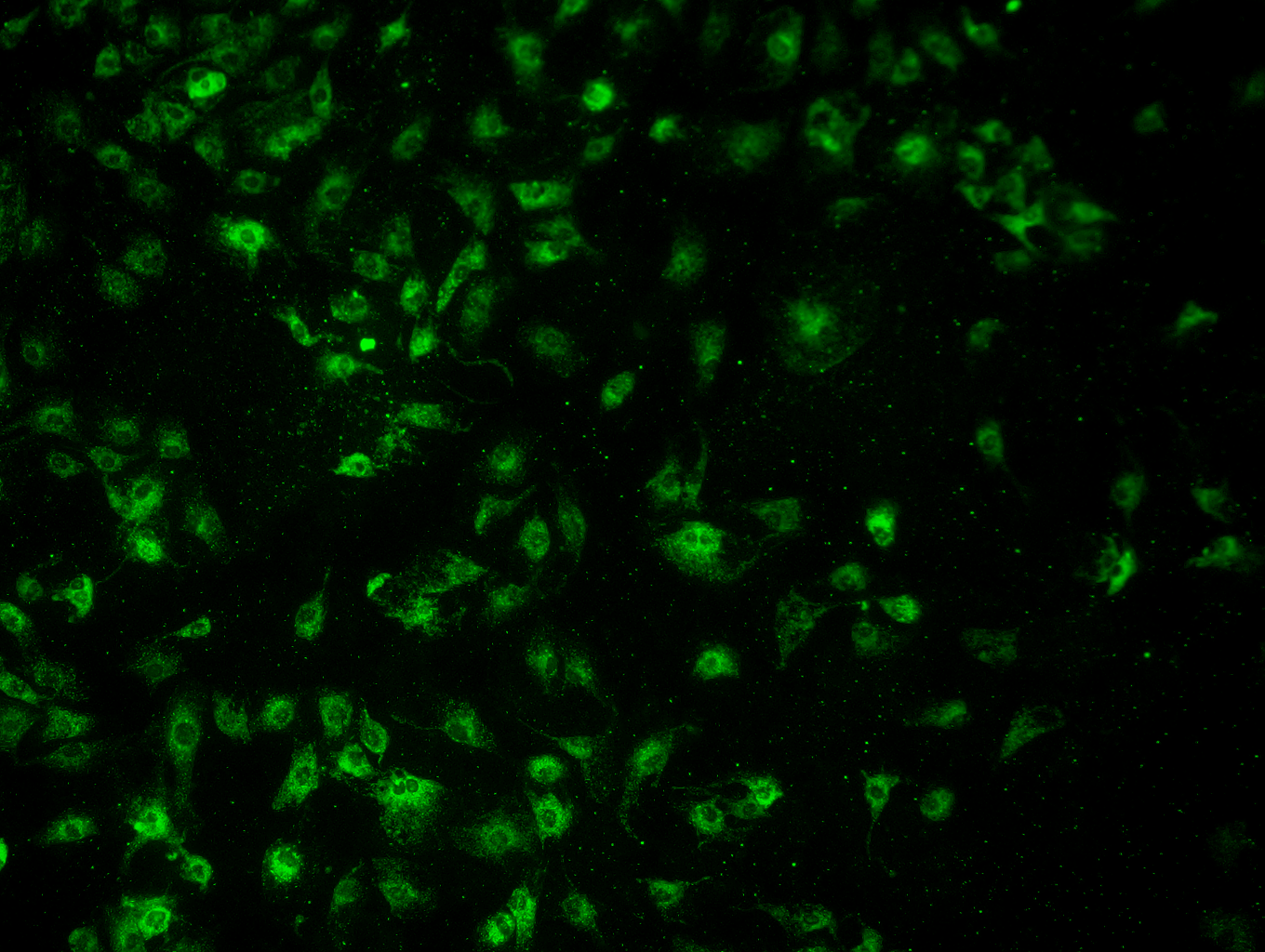

Supplement: Supplementary file 1 [file biomolecules-16-01059-s001.zip › File S1/Supplementary original experimental images-1/Figure 2c/CoCl2+BEL(50μmolL )/1.tif]

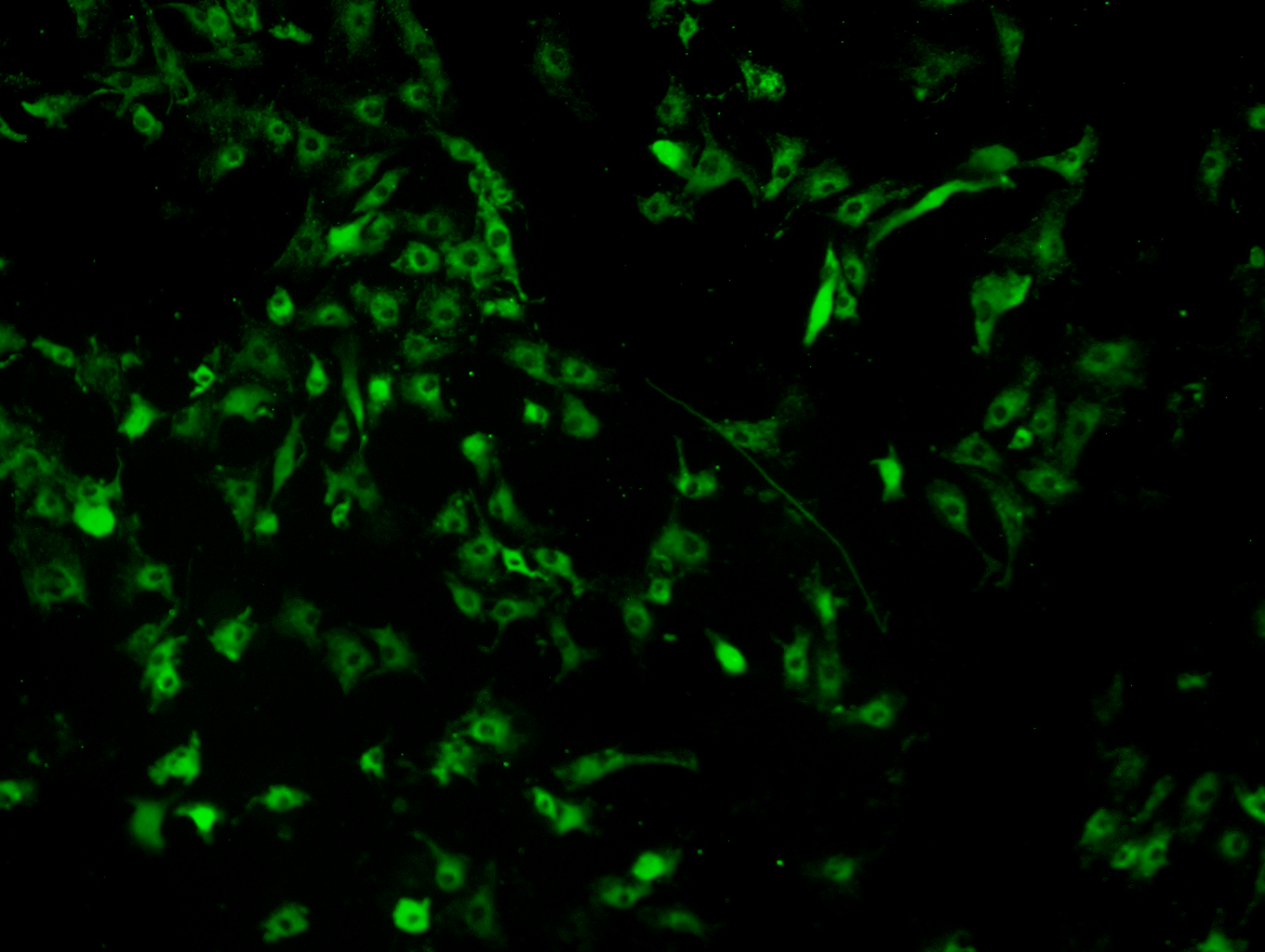

Supplement: Supplementary file 1 [file biomolecules-16-01059-s001.zip › File S1/Supplementary original experimental images-1/Figure 2c/CoCl2+BEL(50μmolL )/2.tif]

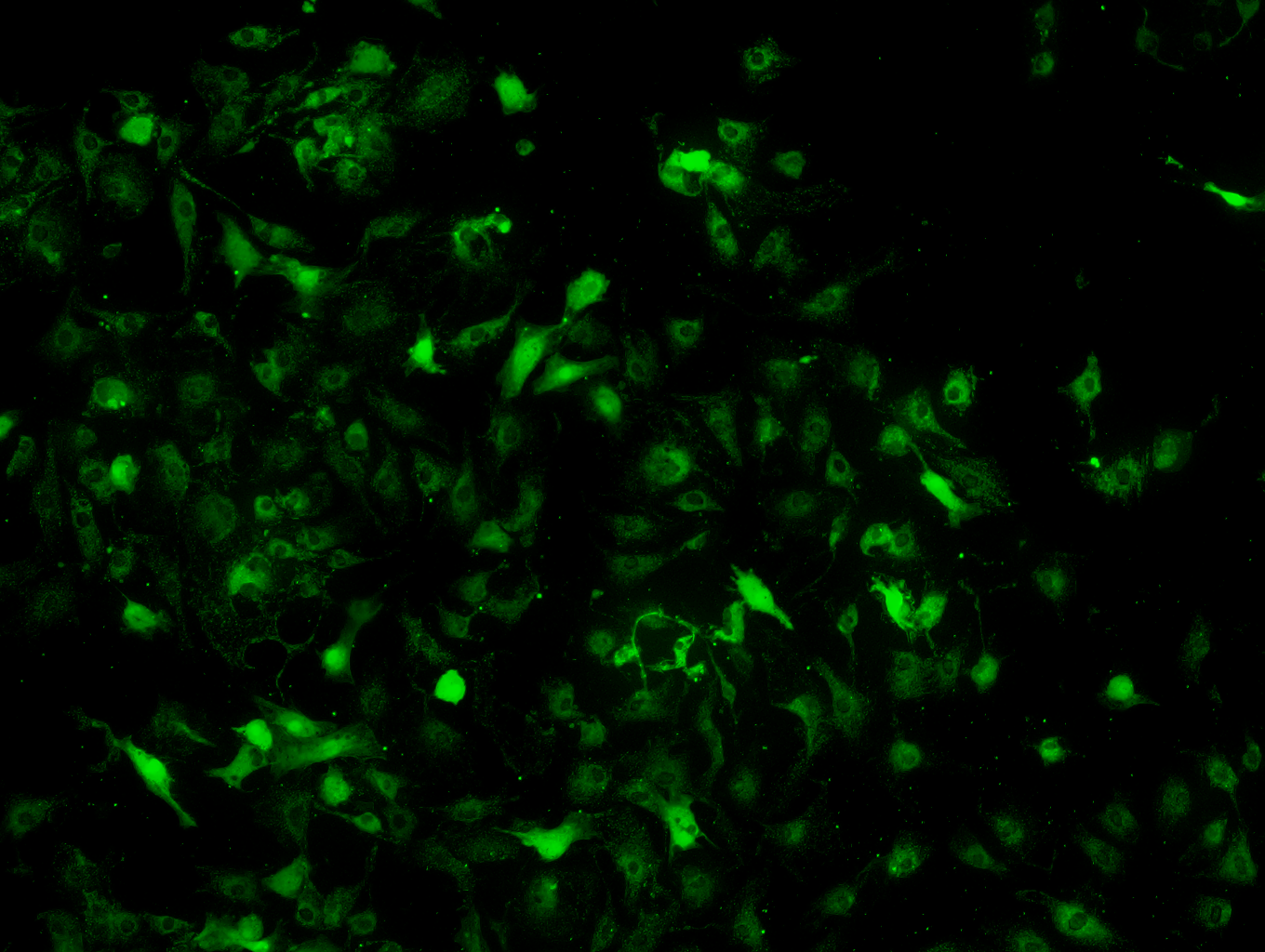

Supplement: Supplementary file 1 [file biomolecules-16-01059-s001.zip › File S1/Supplementary original experimental images-1/Figure 2c/CoCl2+BEL(50μmolL )/3.tif]

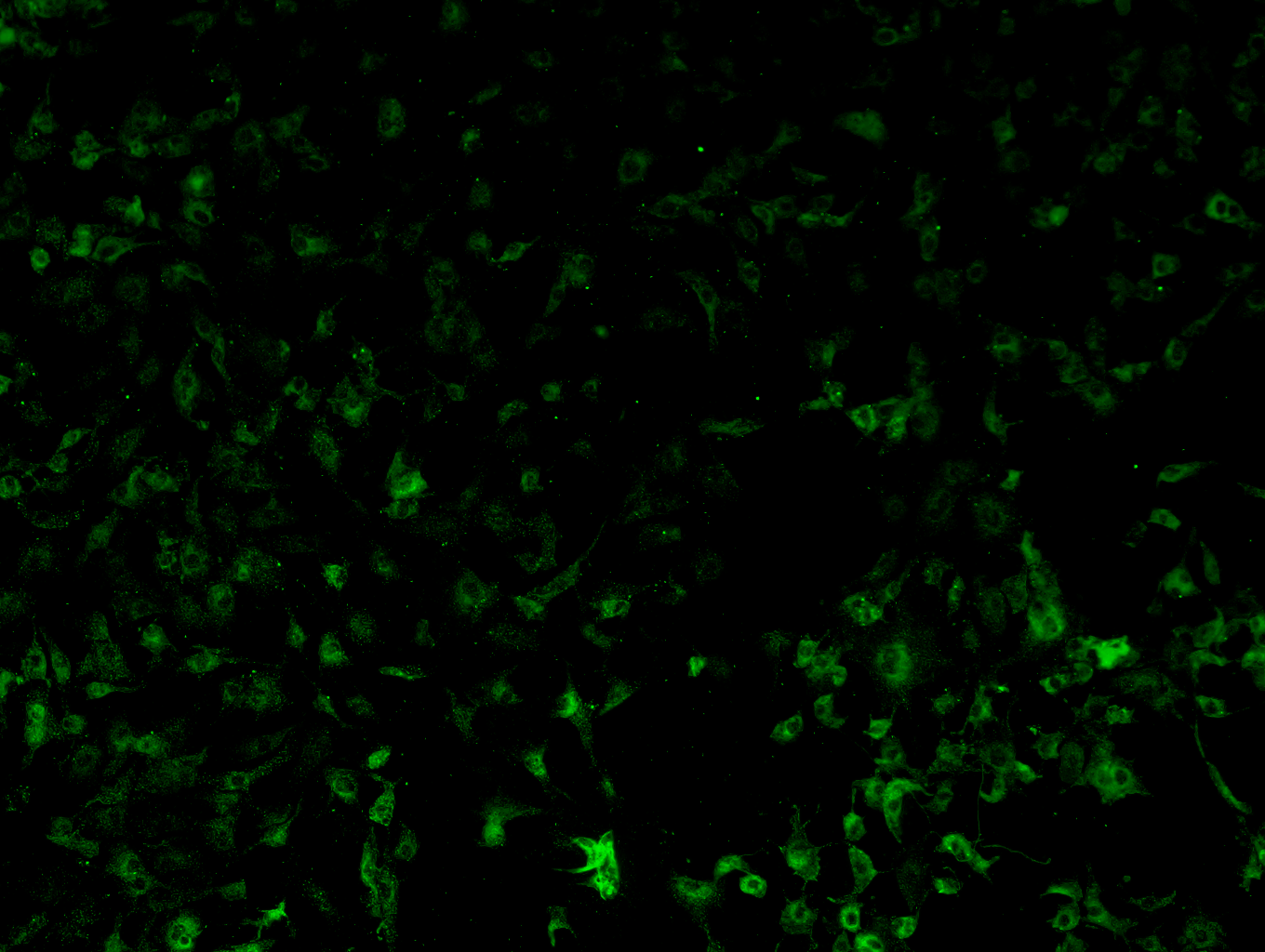

Supplement: Supplementary file 1 [file biomolecules-16-01059-s001.zip › File S1/Supplementary original experimental images-1/Figure 2c/CoCl2+BEL(80μmolL )/1.tif]

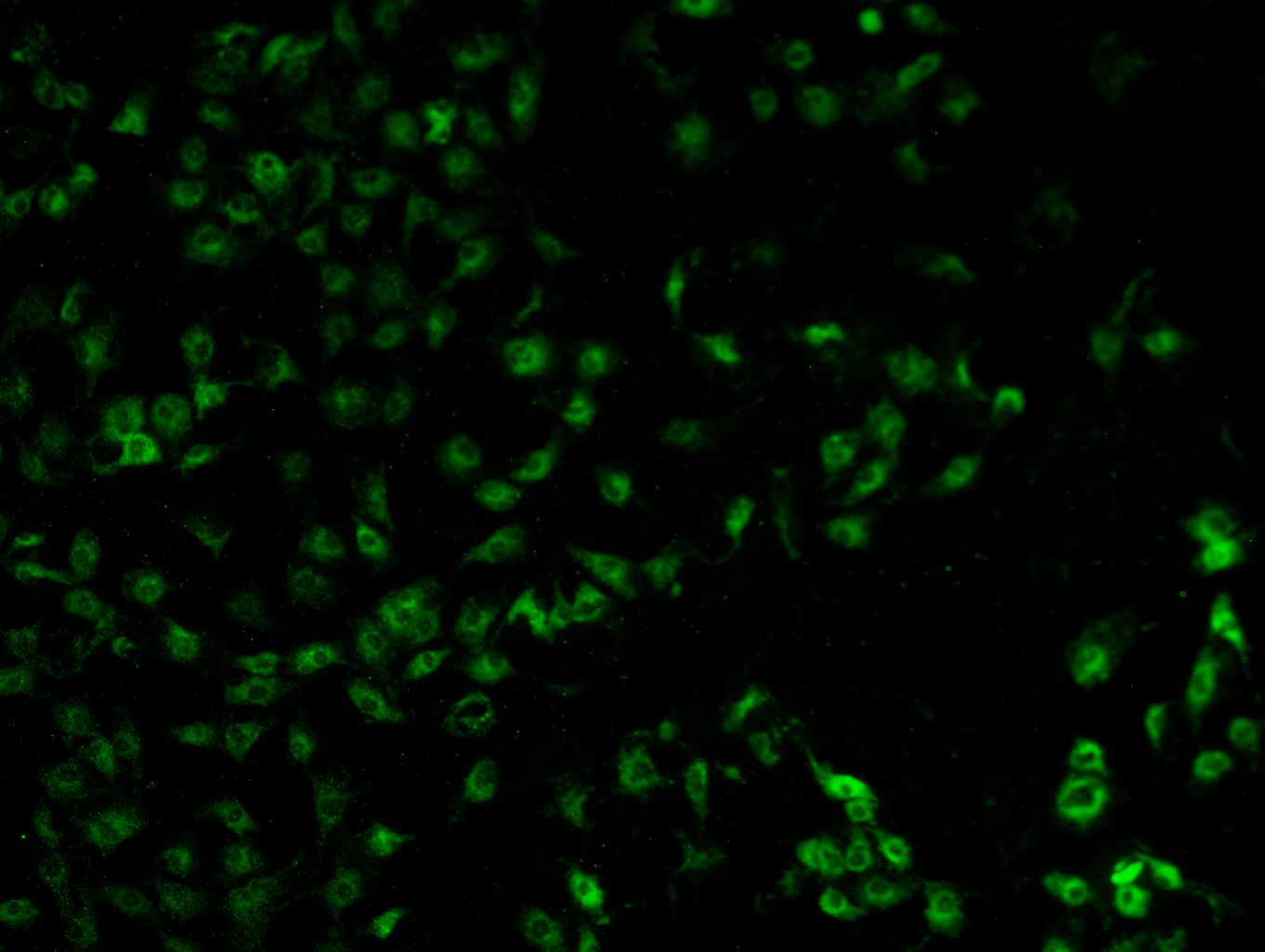

Supplement: Supplementary file 1 [file biomolecules-16-01059-s001.zip › File S1/Supplementary original experimental images-1/Figure 2c/CoCl2+BEL(80μmolL )/2.tif]

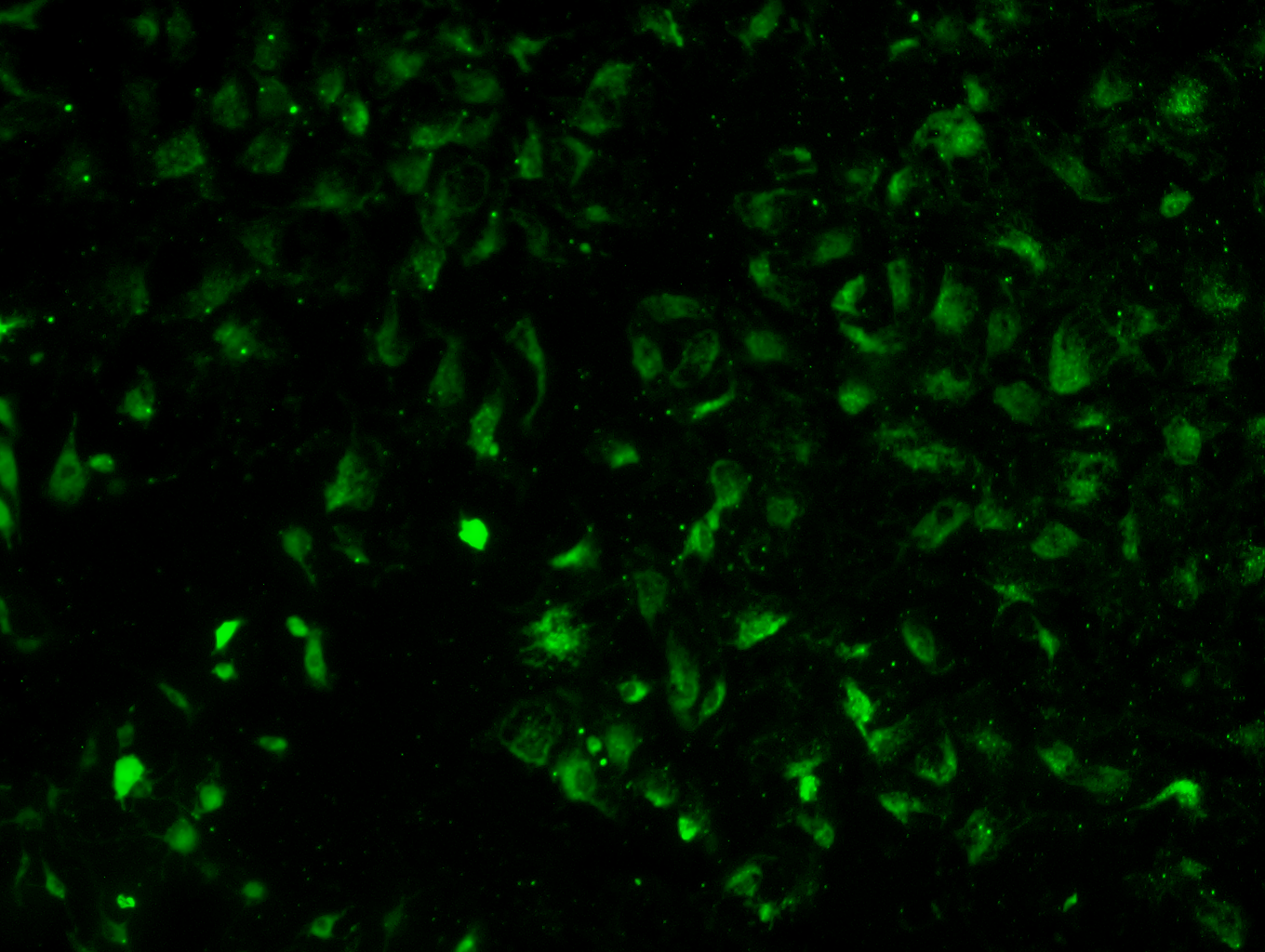

Supplement: Supplementary file 1 [file biomolecules-16-01059-s001.zip › File S1/Supplementary original experimental images-1/Figure 2c/CoCl2+BEL(80μmolL )/3.tif]

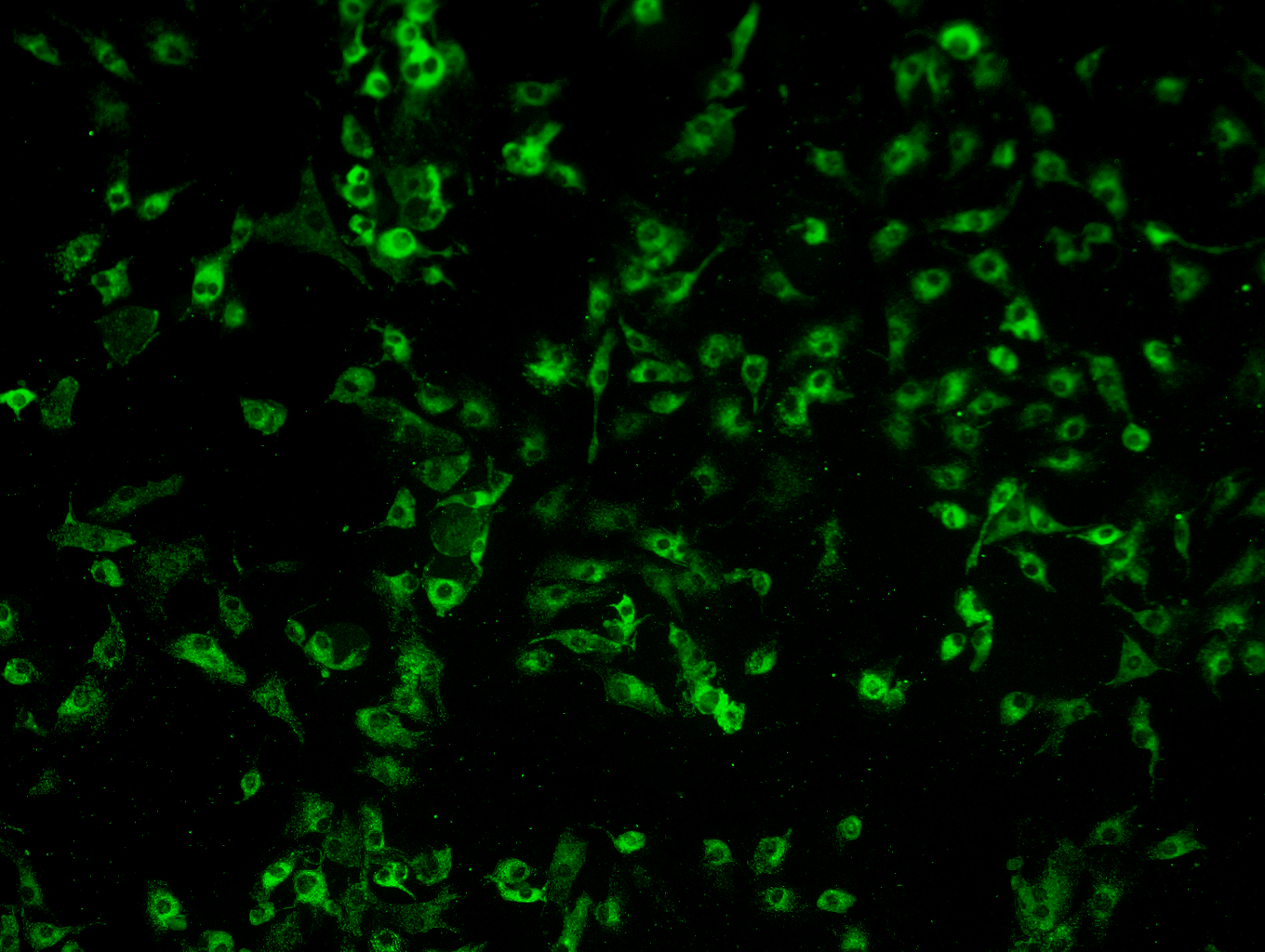

Supplement: Supplementary file 1 [file biomolecules-16-01059-s001.zip › File S1/Supplementary original experimental images-1/Figure 2c/control/1.tif]

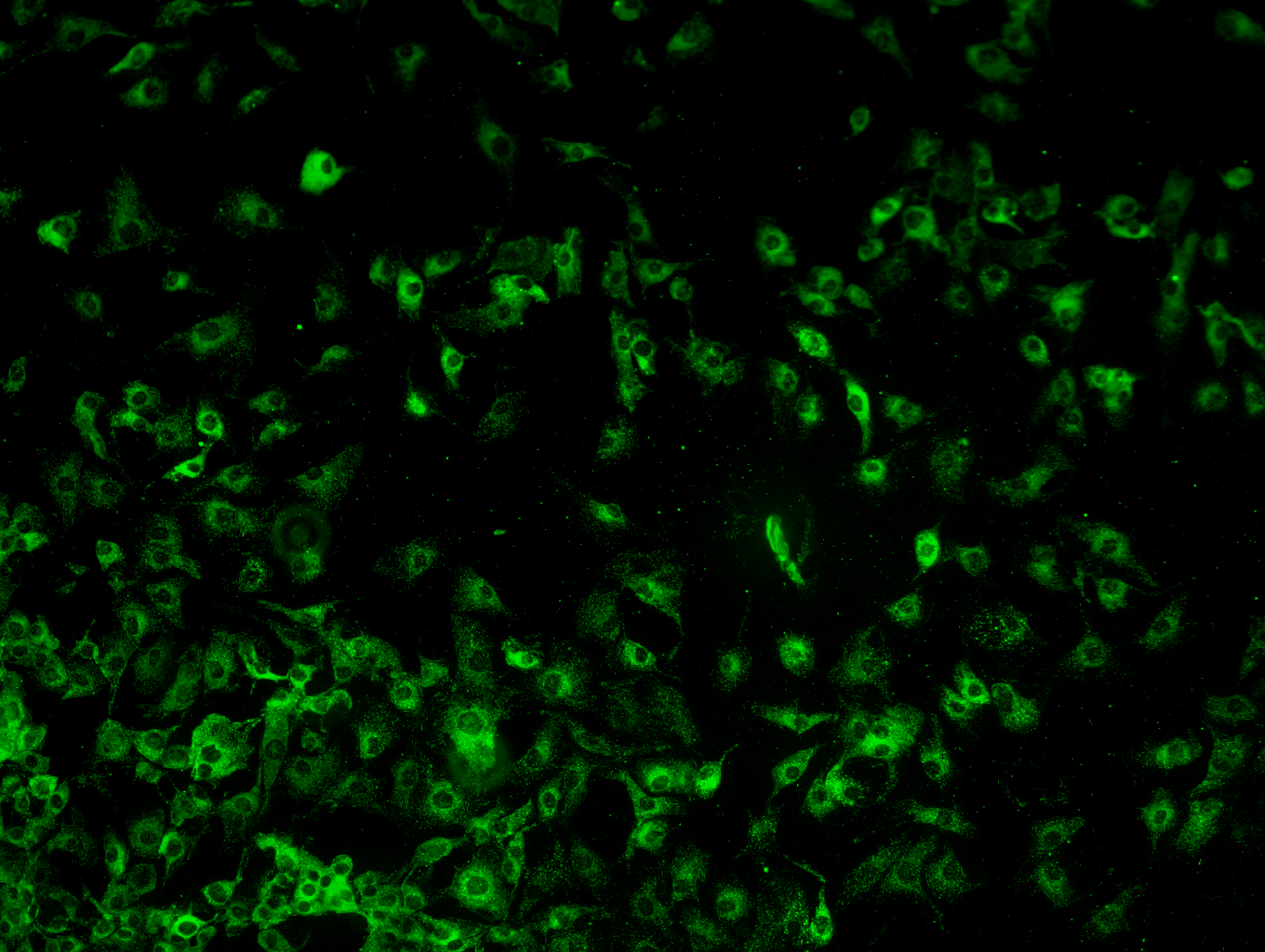

Supplement: Supplementary file 1 [file biomolecules-16-01059-s001.zip › File S1/Supplementary original experimental images-1/Figure 2c/control/2.tif]
