# Supplementary figures and images for: Bellidifolin Improves Pulmonary Artery Smooth Muscle Cells Proliferation by Targeting the IGFBP5-Mediated PI3K-AKT-mTOR Pathway and Dilates the Pulmonary Artery (part 1 of 2)
Source: Biomolecules. 2026 Jul 19;16(7):1059. doi: 10.3390/biom16071059 (PMC13406516; doi:10.3390/biom16071059)

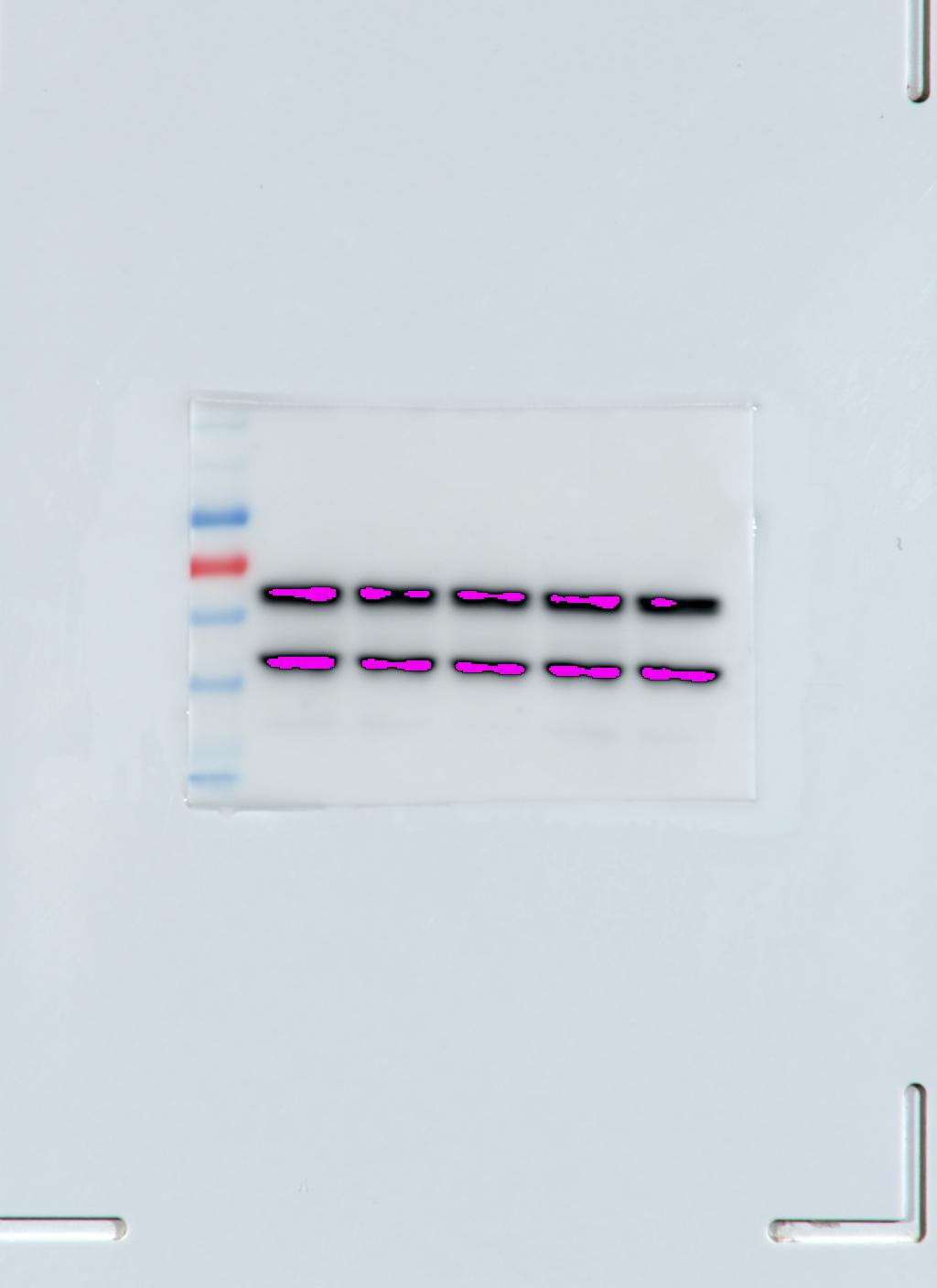

Supplement: Supplementary file 1 [file biomolecules-16-01059-s001.zip › File S1/Figure 6-8-11 Western blot original drawing/Figure 11a/AKt/1/Akt-1.jpg]

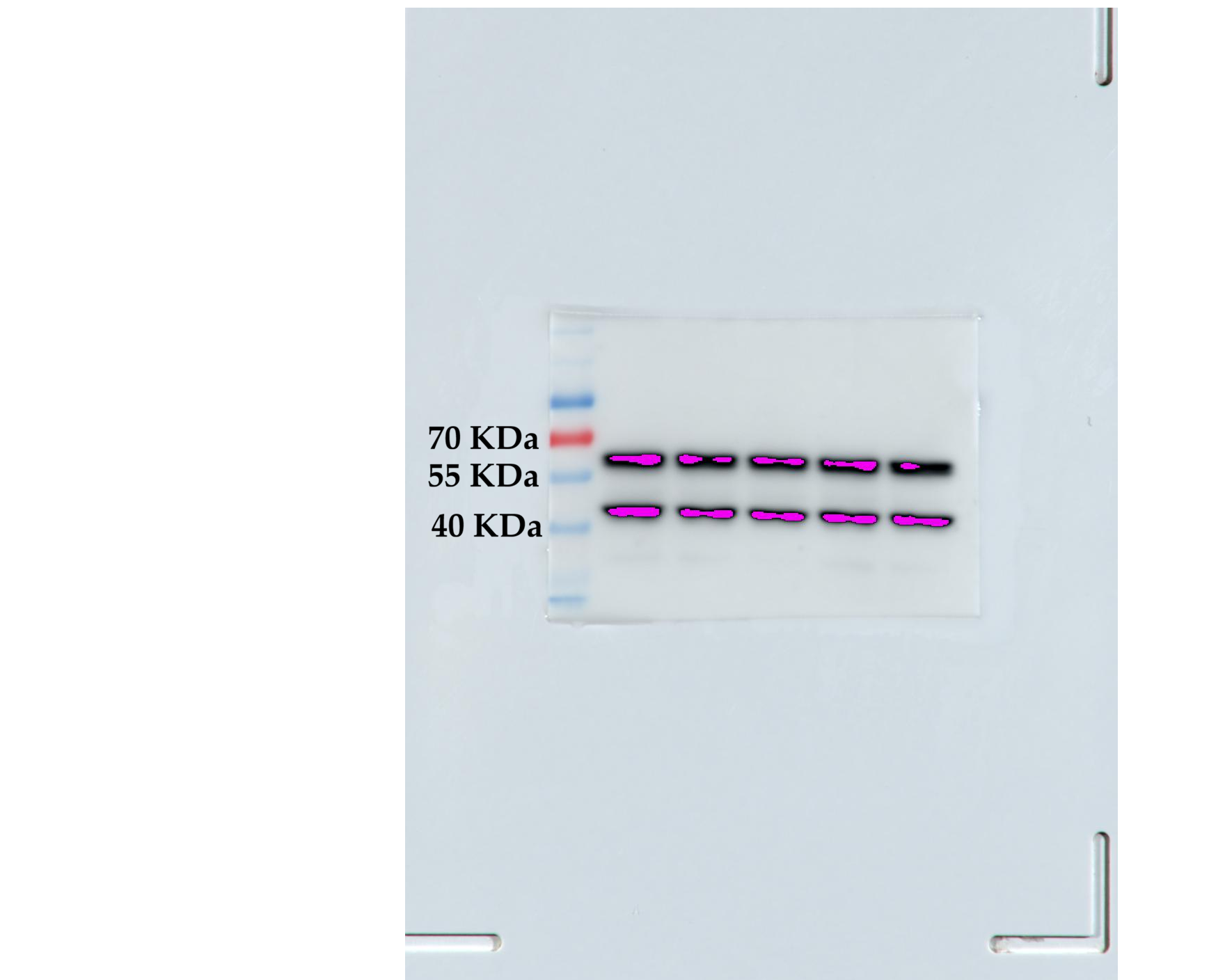

Supplement: Supplementary file 1 [file biomolecules-16-01059-s001.zip › File S1/Figure 6-8-11 Western blot original drawing/Figure 11a/AKt/1/Akt-1.png]

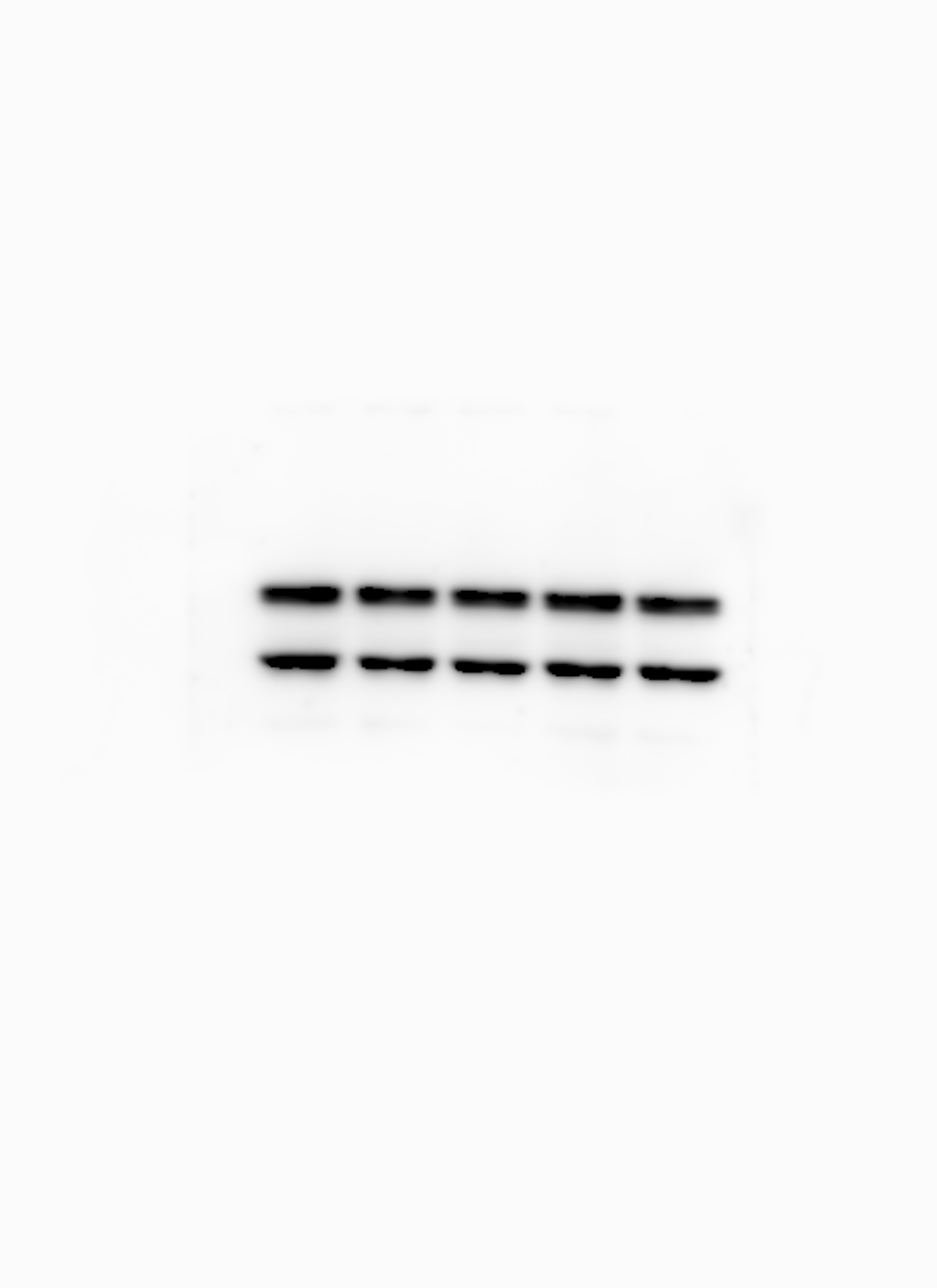

Supplement: Supplementary file 1 [file biomolecules-16-01059-s001.zip › File S1/Figure 6-8-11 Western blot original drawing/Figure 11a/AKt/1/Akt-1.tif]

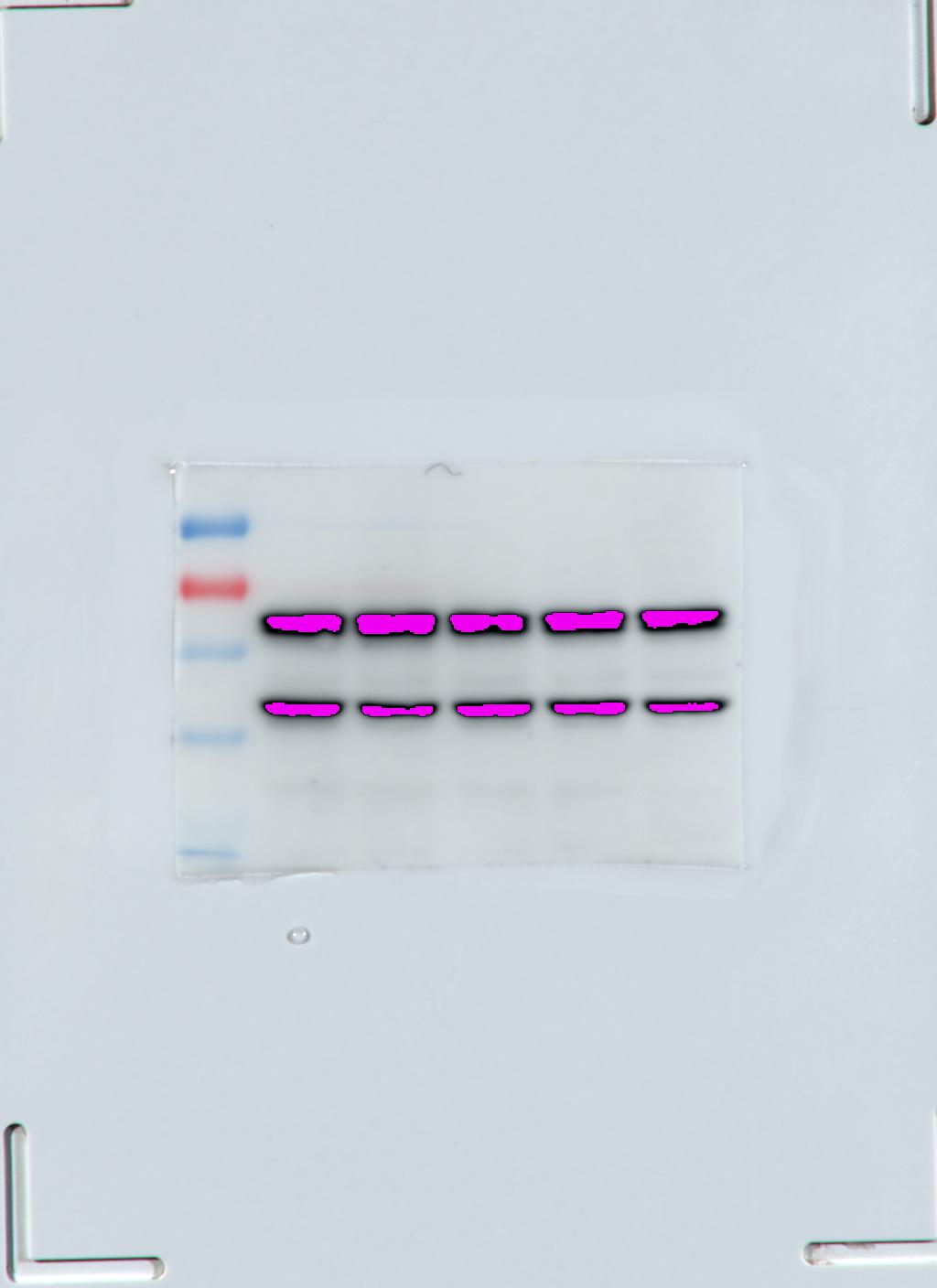

Supplement: Supplementary file 1 [file biomolecules-16-01059-s001.zip › File S1/Figure 6-8-11 Western blot original drawing/Figure 11a/AKt/2/Akt-2.jpg]

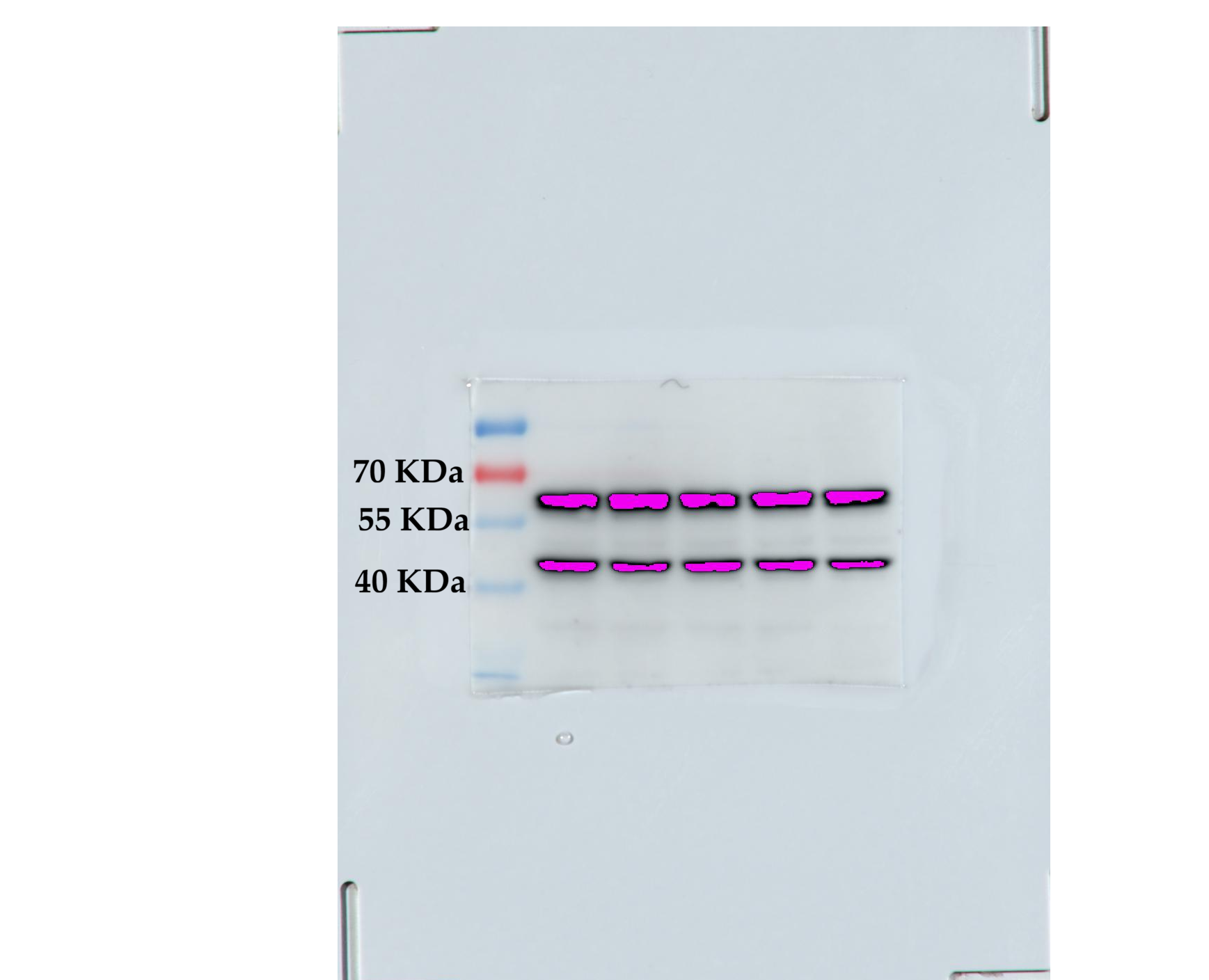

Supplement: Supplementary file 1 [file biomolecules-16-01059-s001.zip › File S1/Figure 6-8-11 Western blot original drawing/Figure 11a/AKt/2/Akt-2.png]

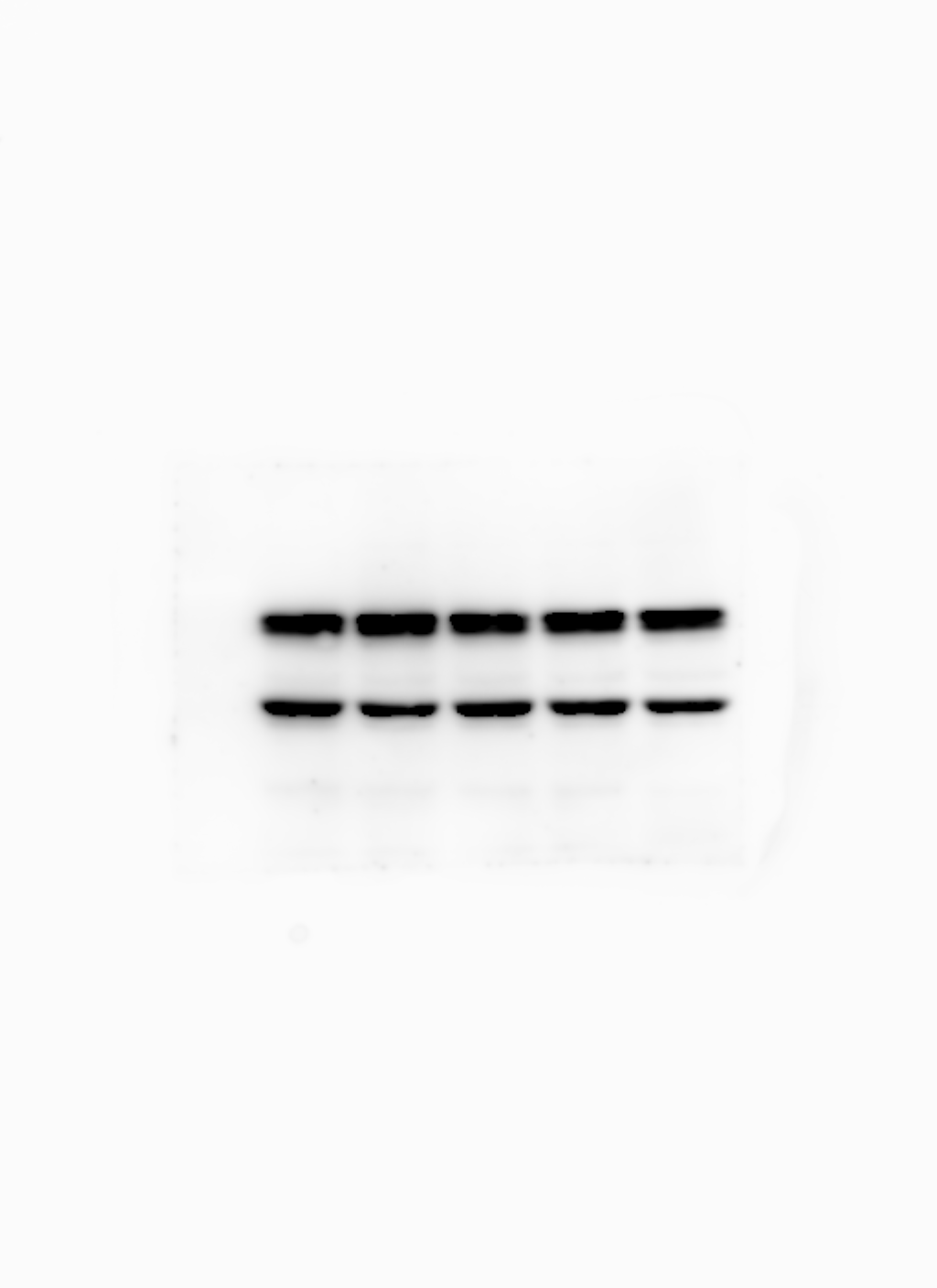

Supplement: Supplementary file 1 [file biomolecules-16-01059-s001.zip › File S1/Figure 6-8-11 Western blot original drawing/Figure 11a/AKt/2/Akt-2.tif]

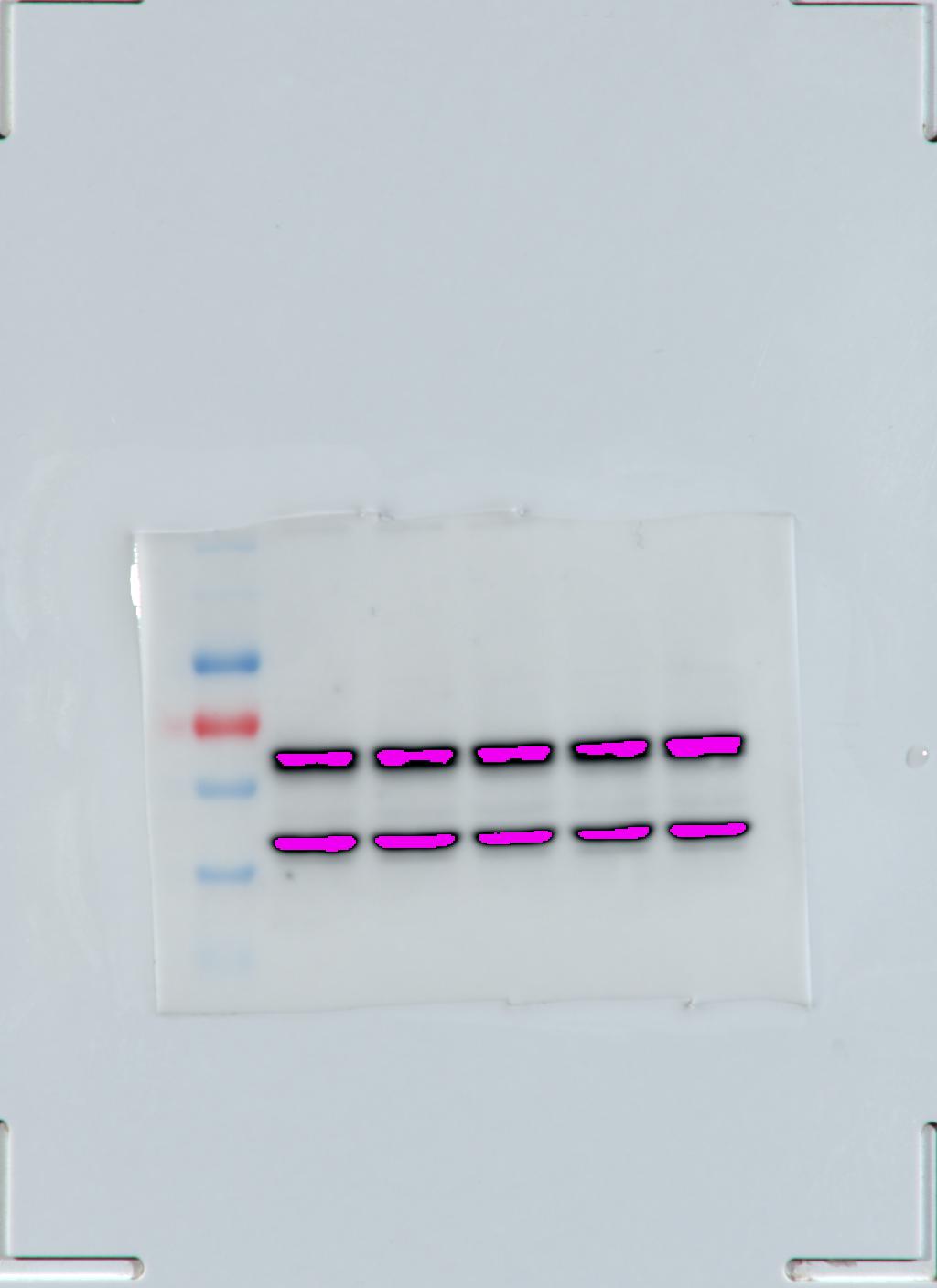

Supplement: Supplementary file 1 [file biomolecules-16-01059-s001.zip › File S1/Figure 6-8-11 Western blot original drawing/Figure 11a/AKt/3/Akt-3.jpg]

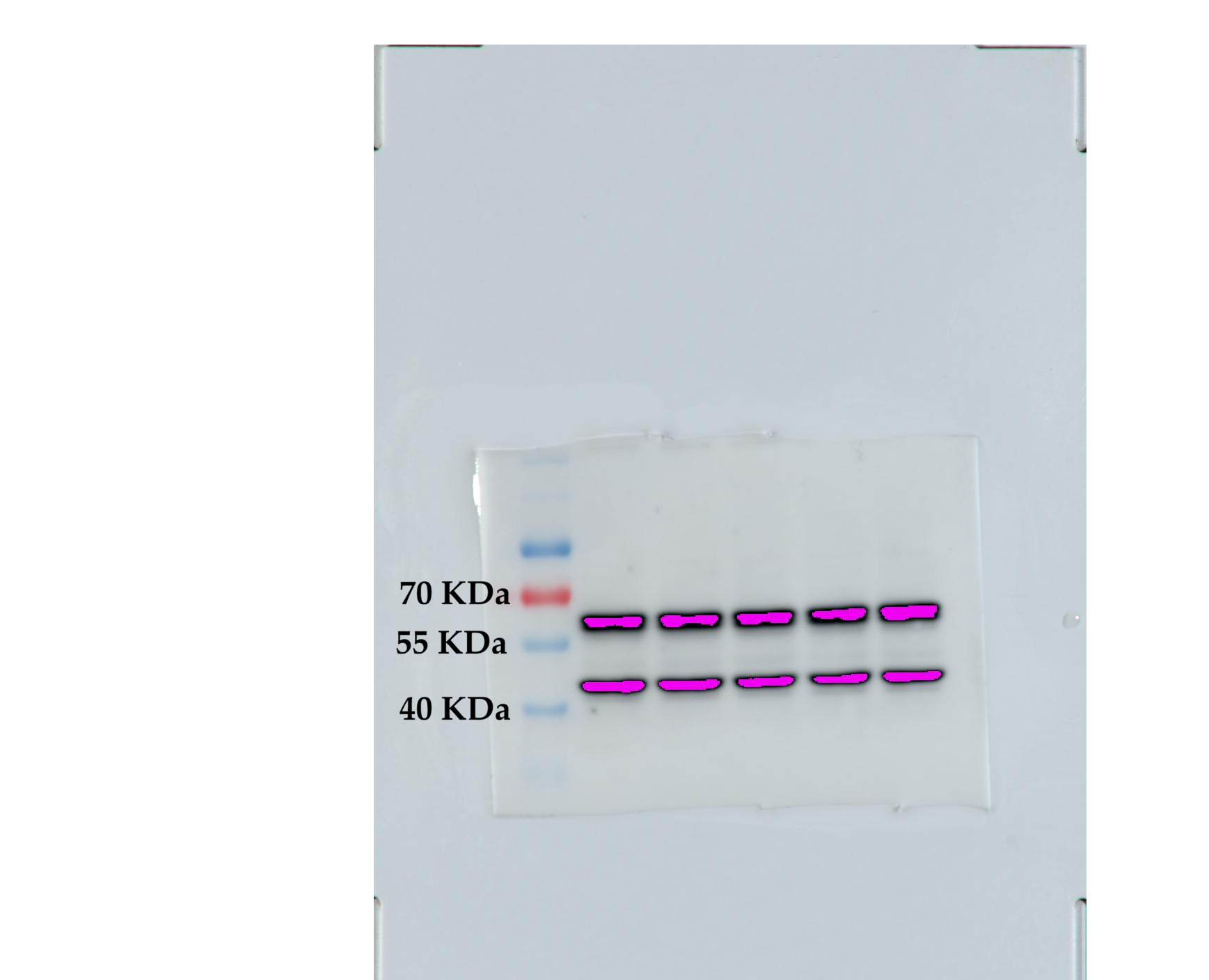

Supplement: Supplementary file 1 [file biomolecules-16-01059-s001.zip › File S1/Figure 6-8-11 Western blot original drawing/Figure 11a/AKt/3/Akt-3.png]

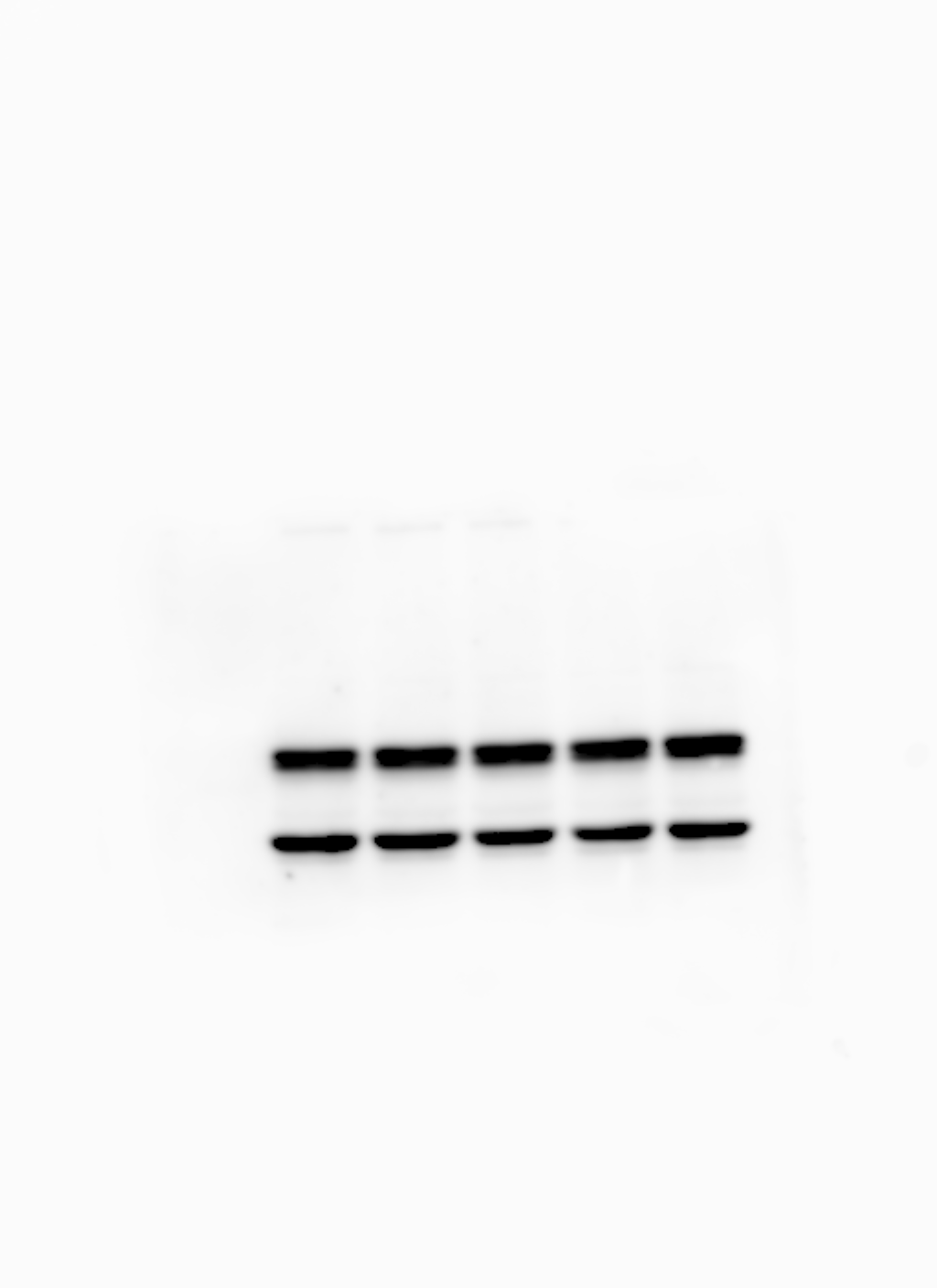

Supplement: Supplementary file 1 [file biomolecules-16-01059-s001.zip › File S1/Figure 6-8-11 Western blot original drawing/Figure 11a/AKt/3/Akt-3.tif]

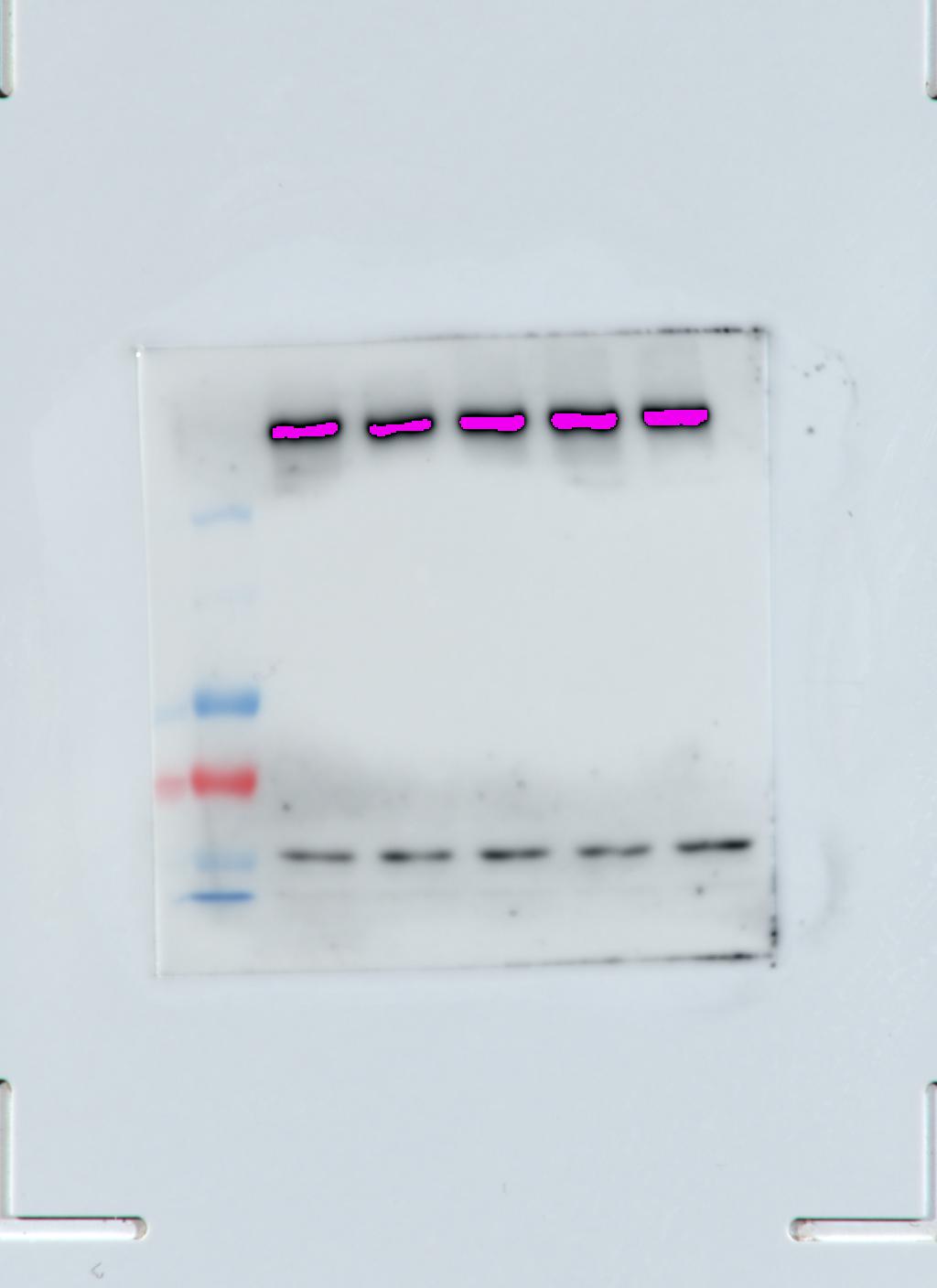

Supplement: Supplementary file 1 [file biomolecules-16-01059-s001.zip › File S1/Figure 6-8-11 Western blot original drawing/Figure 11a/mTOR/1/mTOR-1.jpg]

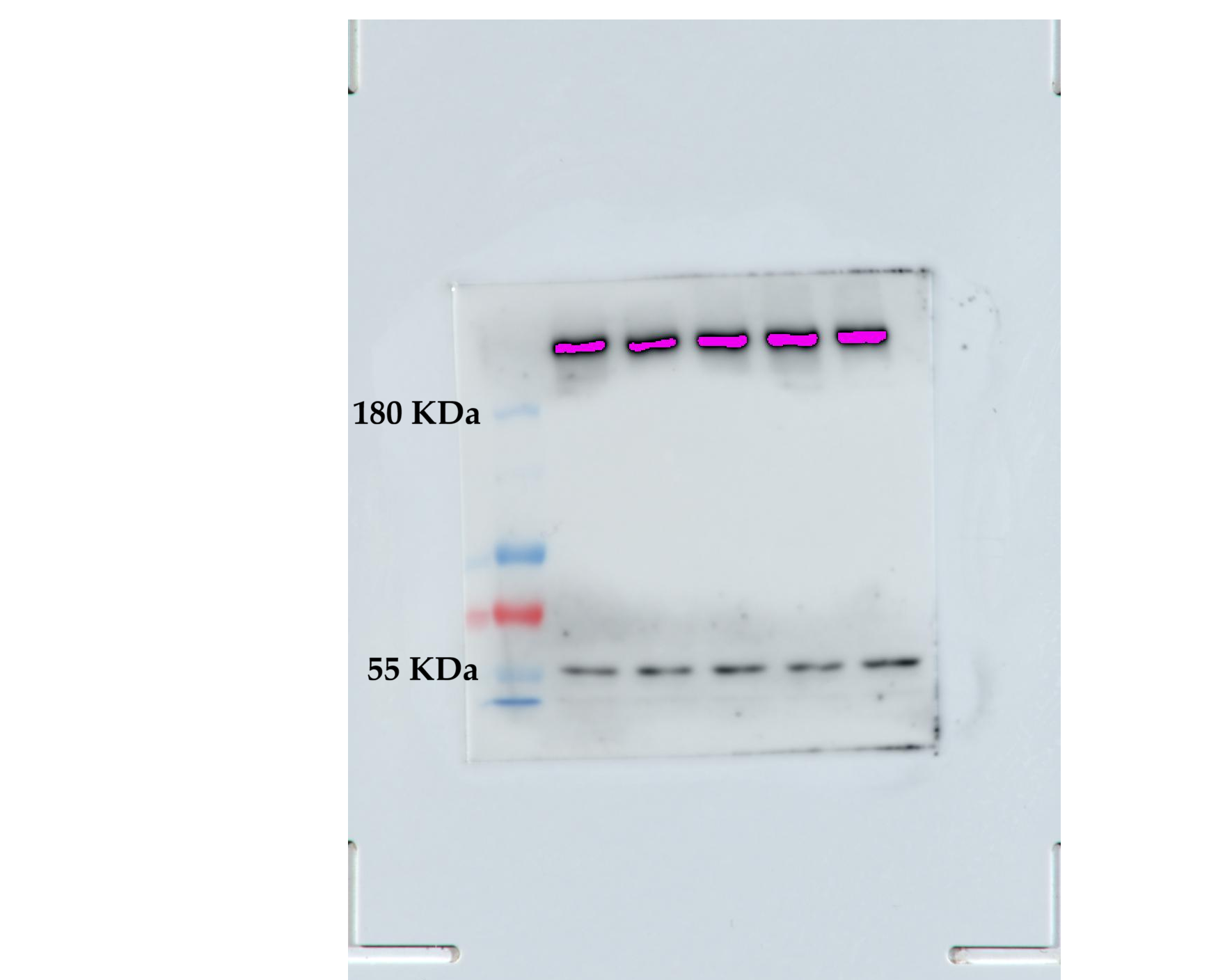

Supplement: Supplementary file 1 [file biomolecules-16-01059-s001.zip › File S1/Figure 6-8-11 Western blot original drawing/Figure 11a/mTOR/1/mTOR-1.png]

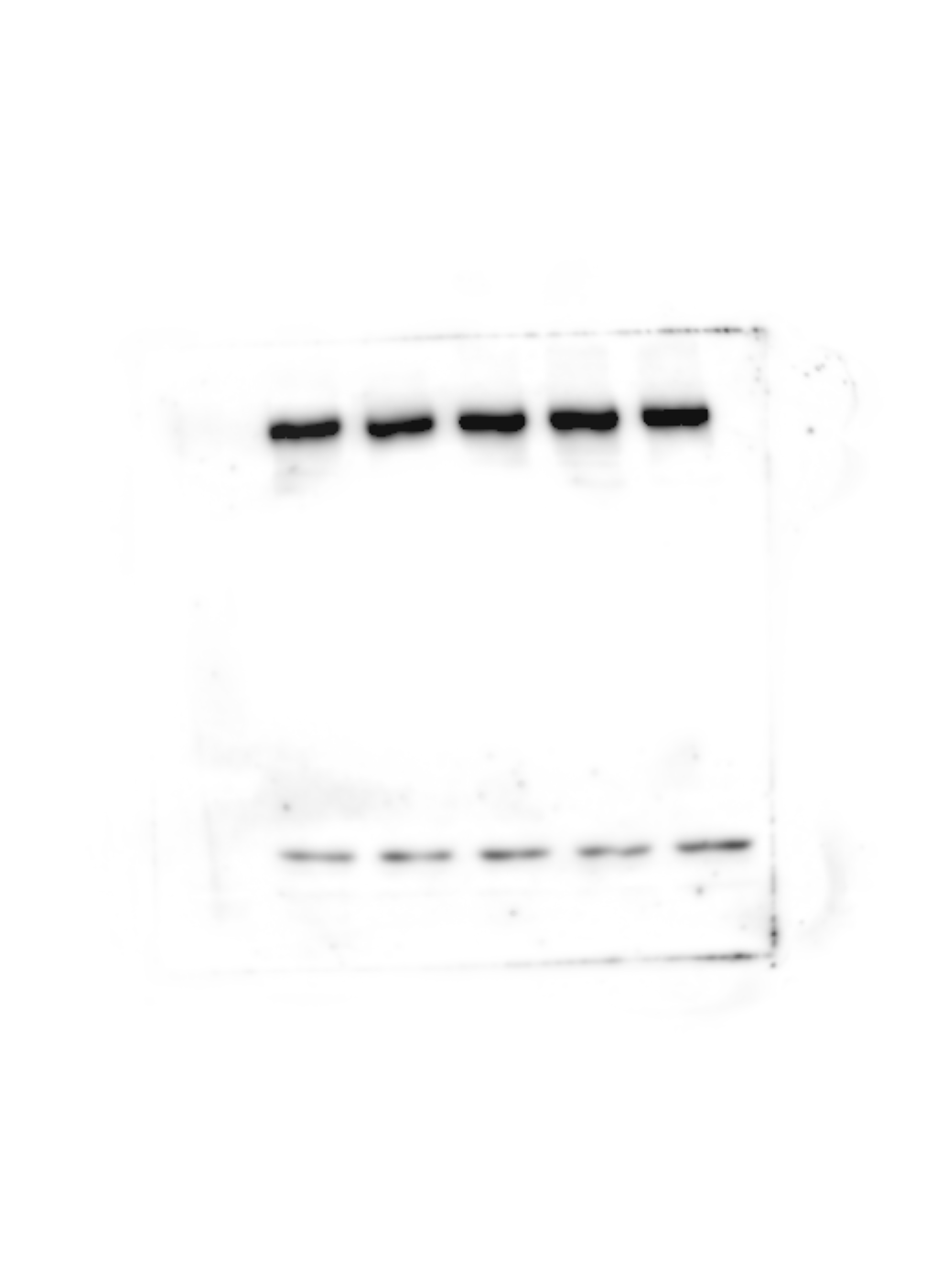

Supplement: Supplementary file 1 [file biomolecules-16-01059-s001.zip › File S1/Figure 6-8-11 Western blot original drawing/Figure 11a/mTOR/1/mTOR-1.tif]

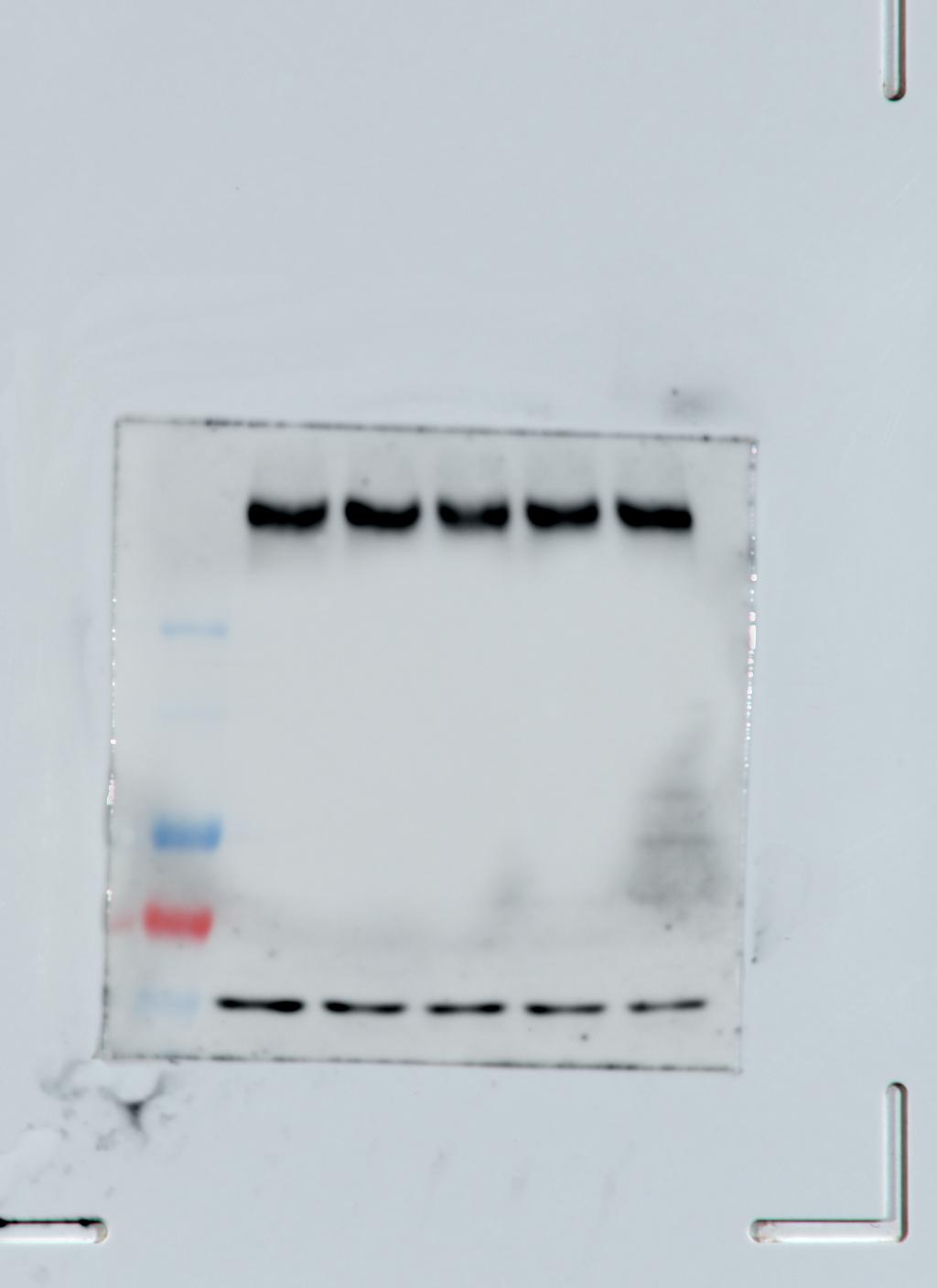

Supplement: Supplementary file 1 [file biomolecules-16-01059-s001.zip › File S1/Figure 6-8-11 Western blot original drawing/Figure 11a/mTOR/2/mTOR-2.jpg]

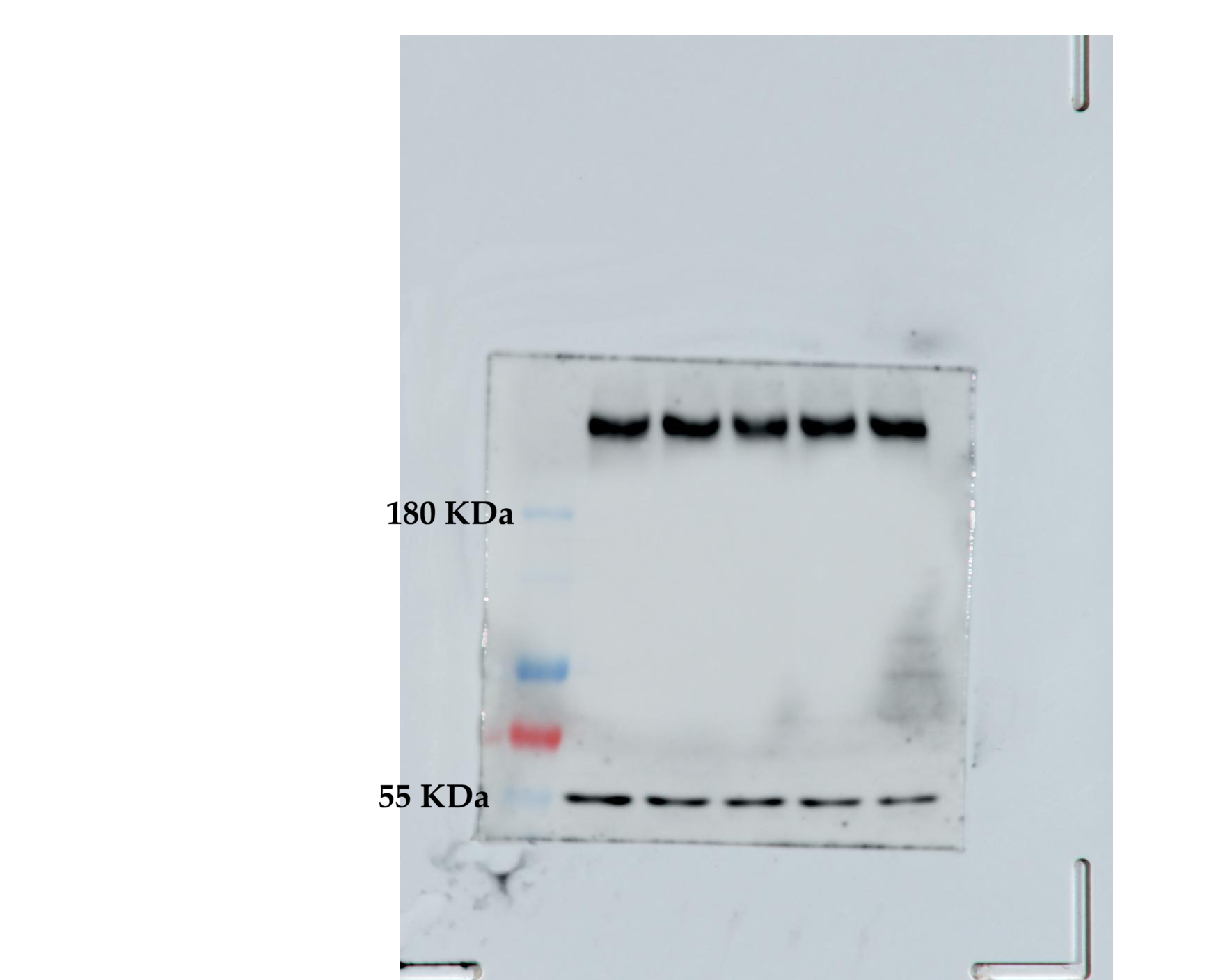

Supplement: Supplementary file 1 [file biomolecules-16-01059-s001.zip › File S1/Figure 6-8-11 Western blot original drawing/Figure 11a/mTOR/2/mTOR-2.png]

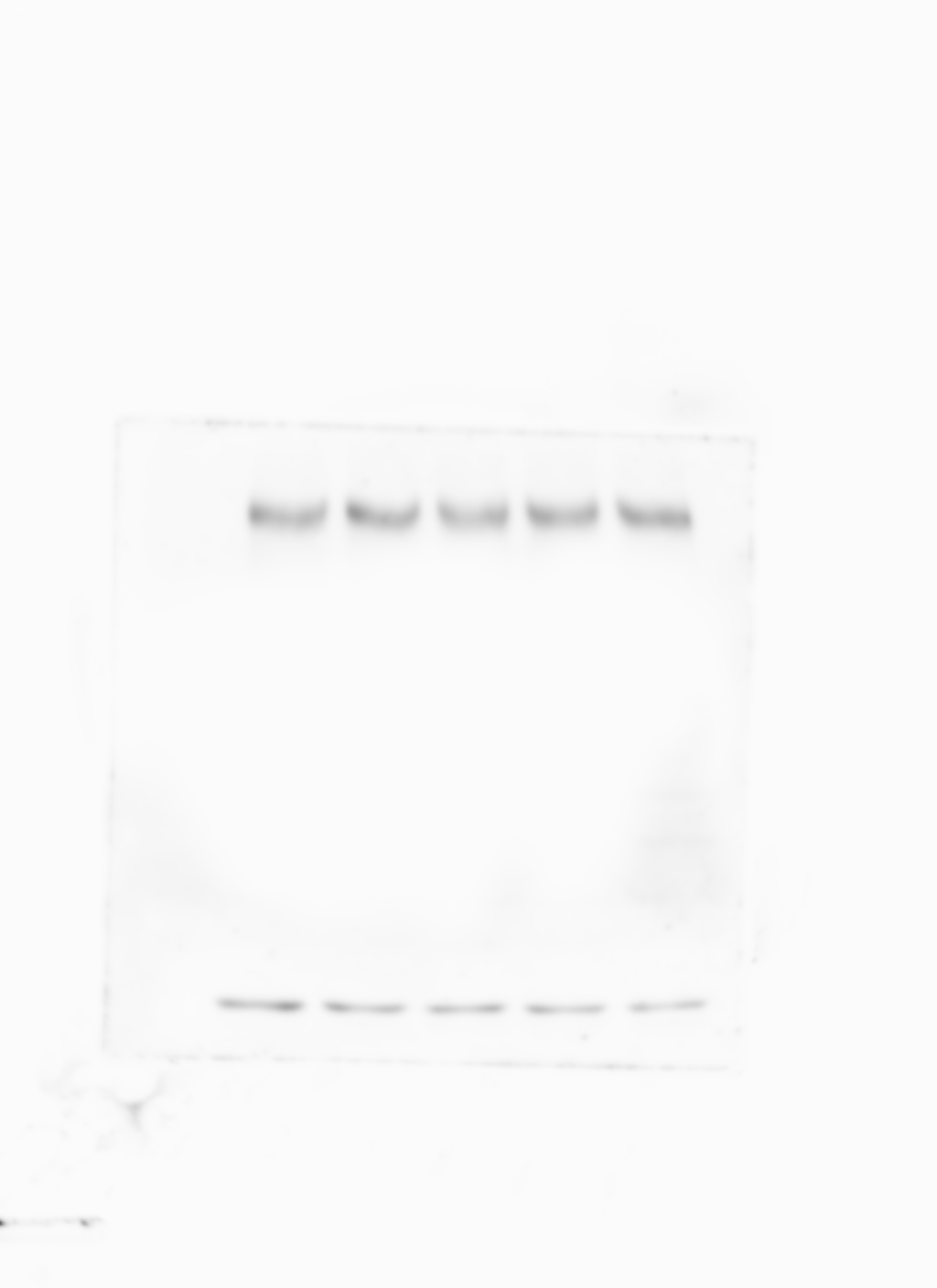

Supplement: Supplementary file 1 [file biomolecules-16-01059-s001.zip › File S1/Figure 6-8-11 Western blot original drawing/Figure 11a/mTOR/2/mTOR-2.tif]

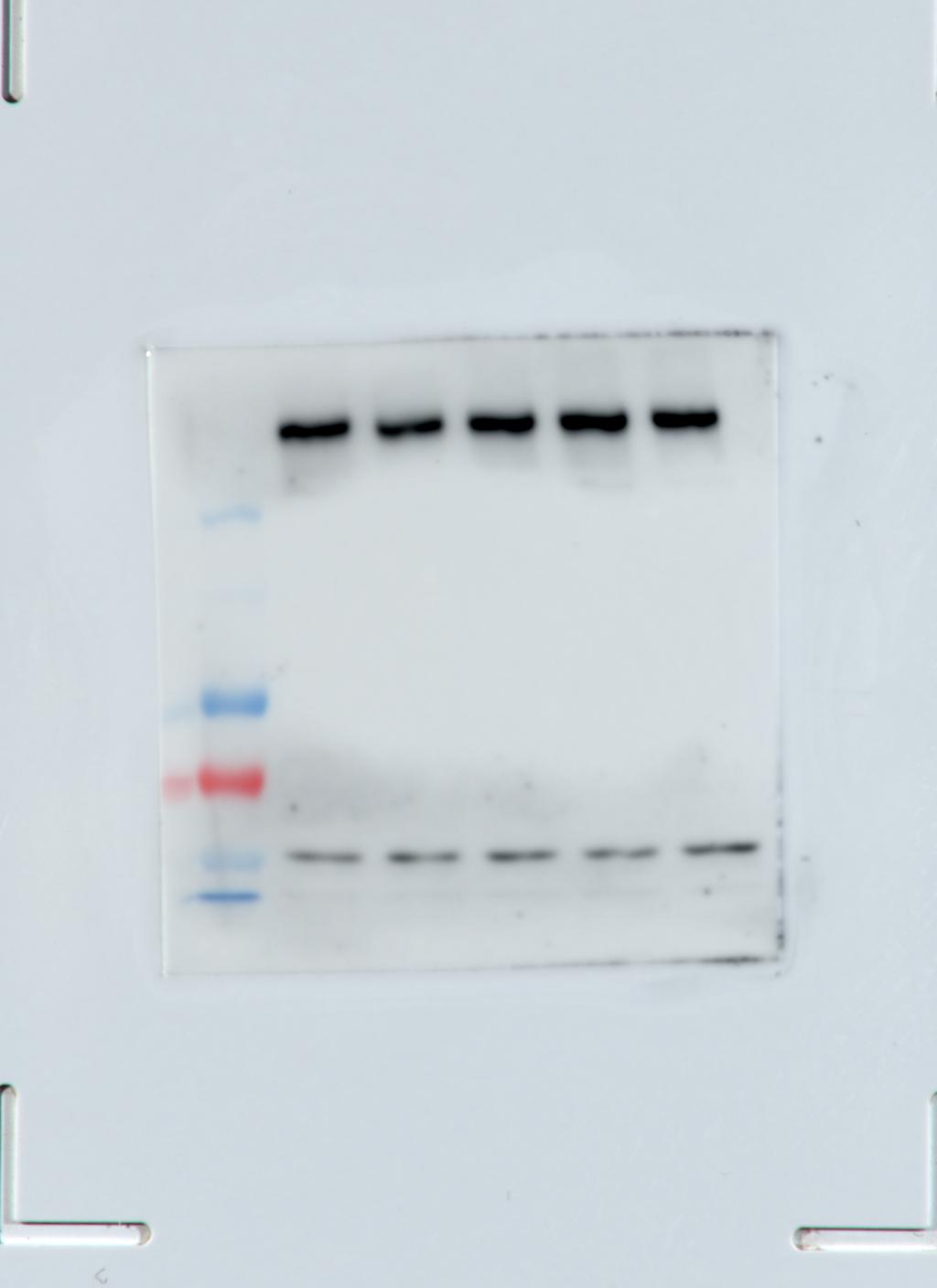

Supplement: Supplementary file 1 [file biomolecules-16-01059-s001.zip › File S1/Figure 6-8-11 Western blot original drawing/Figure 11a/mTOR/3/mTOR-3.jpg]

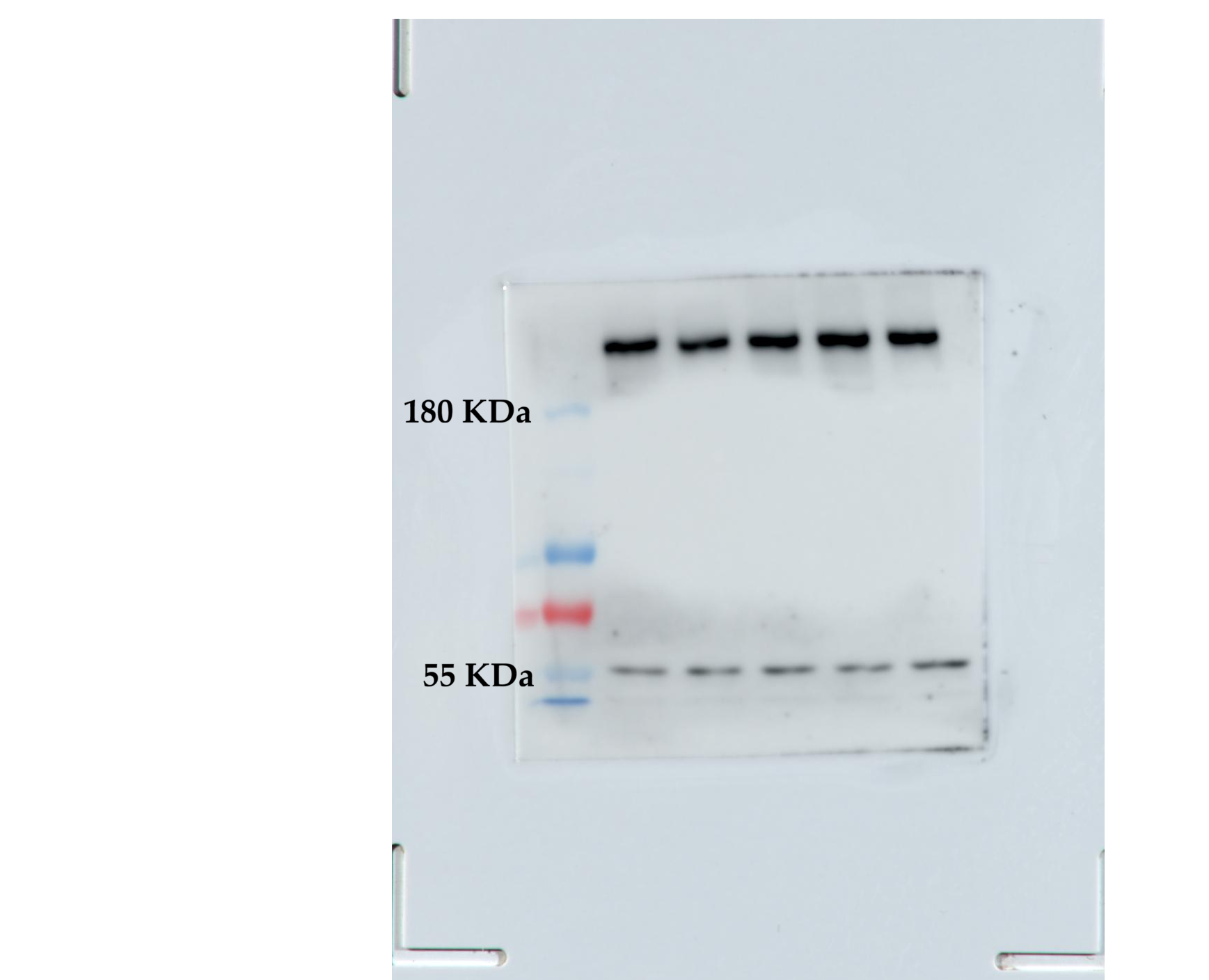

Supplement: Supplementary file 1 [file biomolecules-16-01059-s001.zip › File S1/Figure 6-8-11 Western blot original drawing/Figure 11a/mTOR/3/mTOR-3.png]

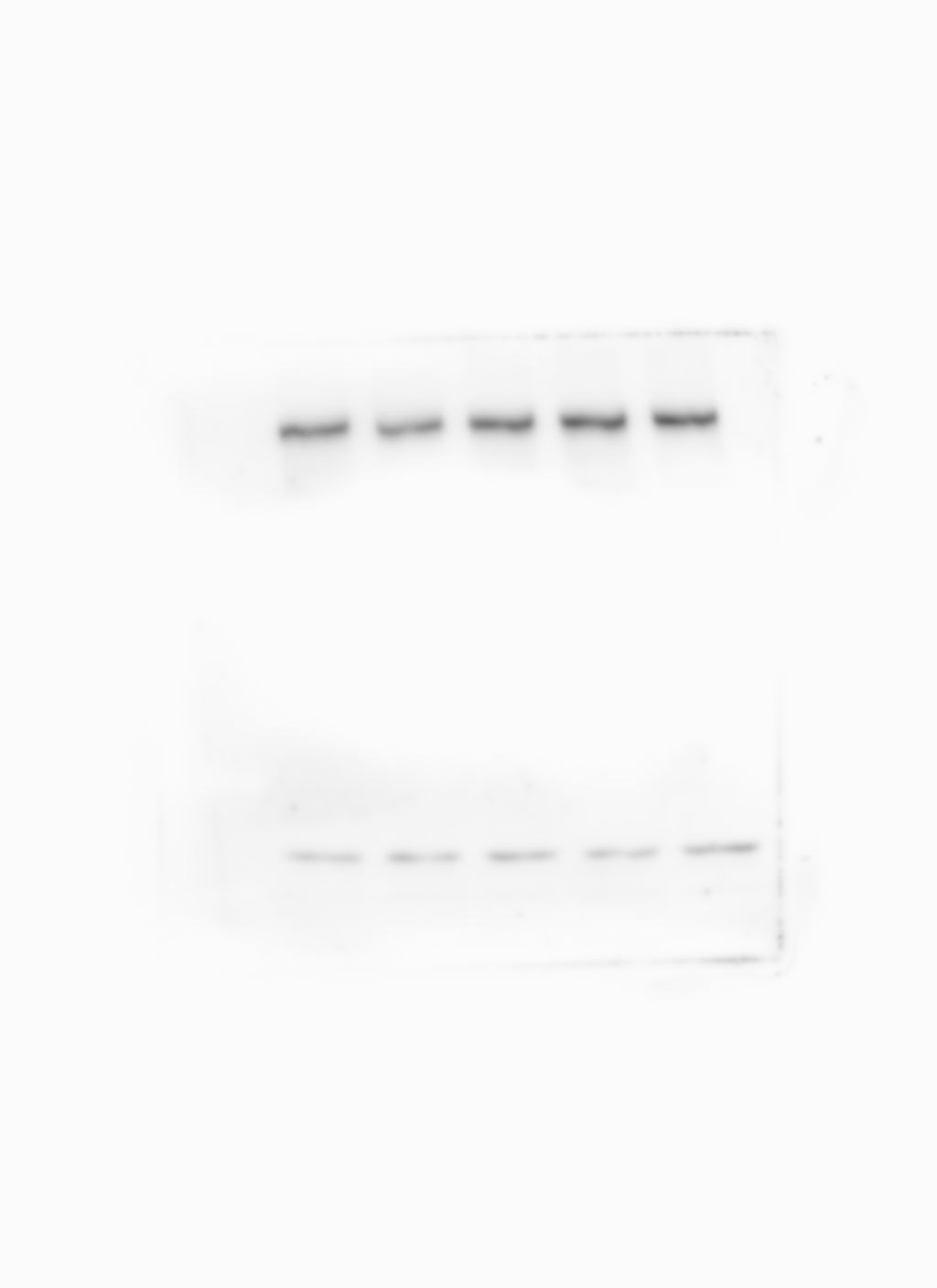

Supplement: Supplementary file 1 [file biomolecules-16-01059-s001.zip › File S1/Figure 6-8-11 Western blot original drawing/Figure 11a/mTOR/3/mTOR-3.tif]

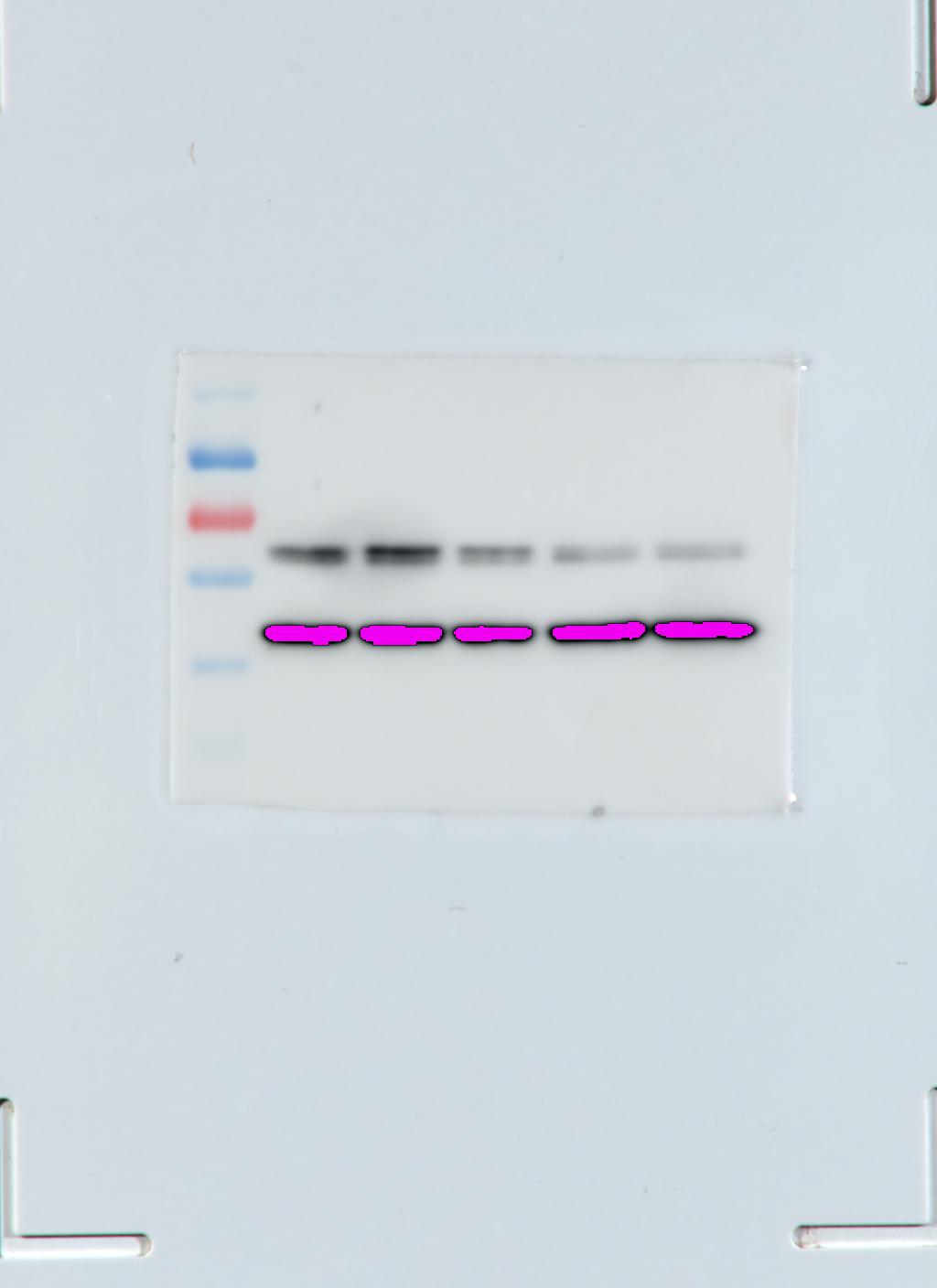

Supplement: Supplementary file 1 [file biomolecules-16-01059-s001.zip › File S1/Figure 6-8-11 Western blot original drawing/Figure 11a/P-Akt/1/p-Akt-1.jpg]

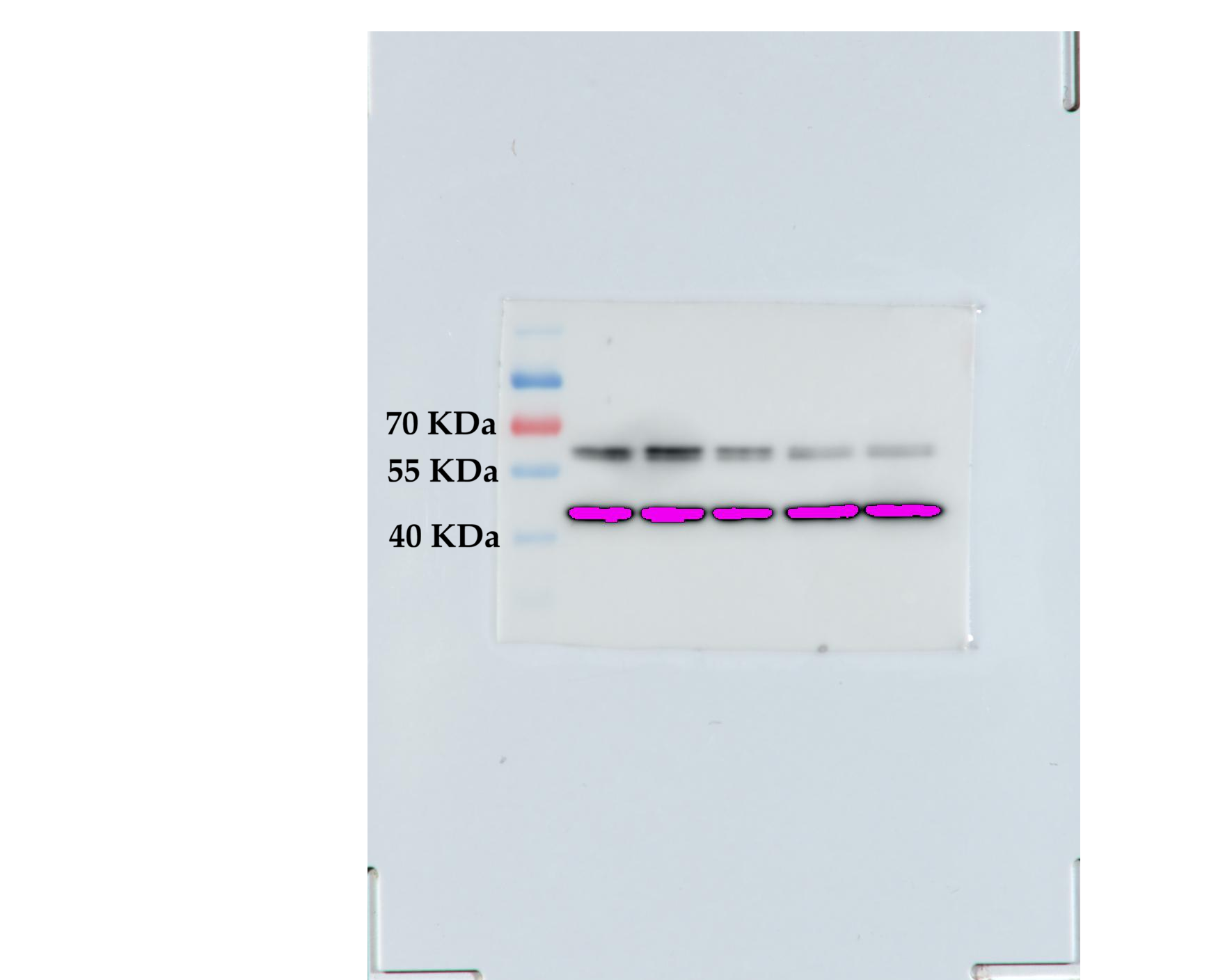

Supplement: Supplementary file 1 [file biomolecules-16-01059-s001.zip › File S1/Figure 6-8-11 Western blot original drawing/Figure 11a/P-Akt/1/p-Akt-1.png]

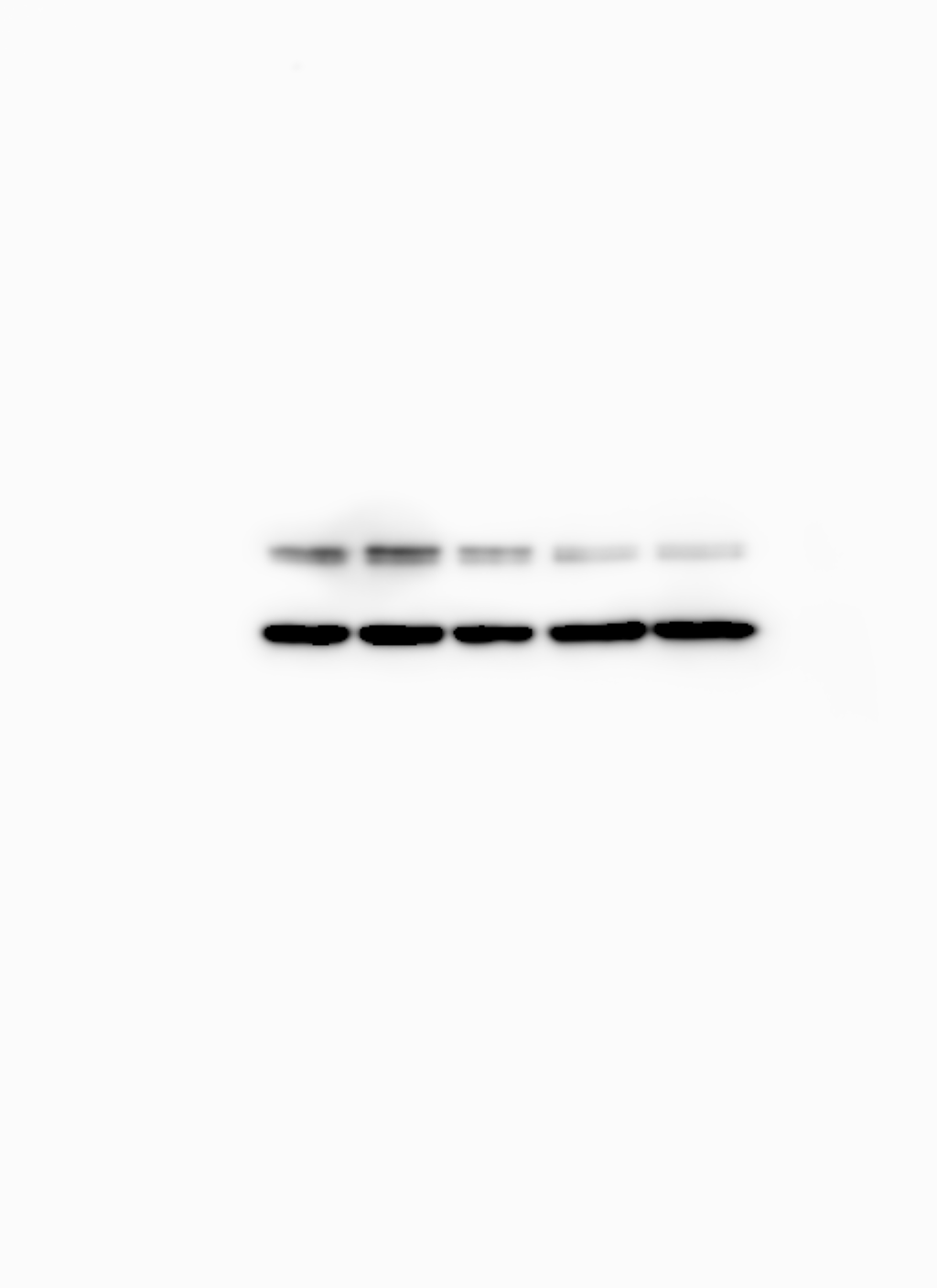

Supplement: Supplementary file 1 [file biomolecules-16-01059-s001.zip › File S1/Figure 6-8-11 Western blot original drawing/Figure 11a/P-Akt/1/p-Akt-1.tif]

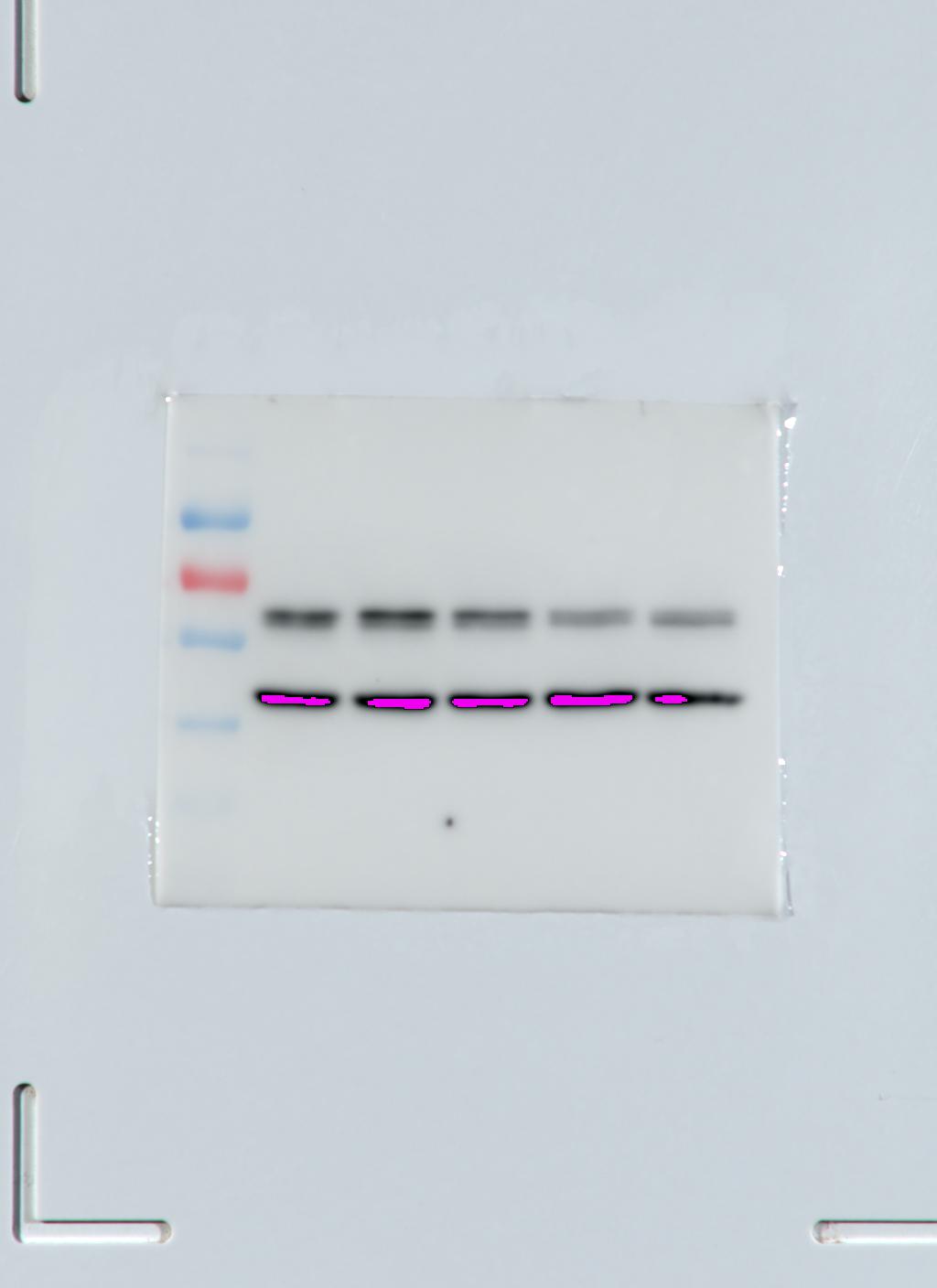

Supplement: Supplementary file 1 [file biomolecules-16-01059-s001.zip › File S1/Figure 6-8-11 Western blot original drawing/Figure 11a/P-Akt/2/p-Akt-2.jpg]

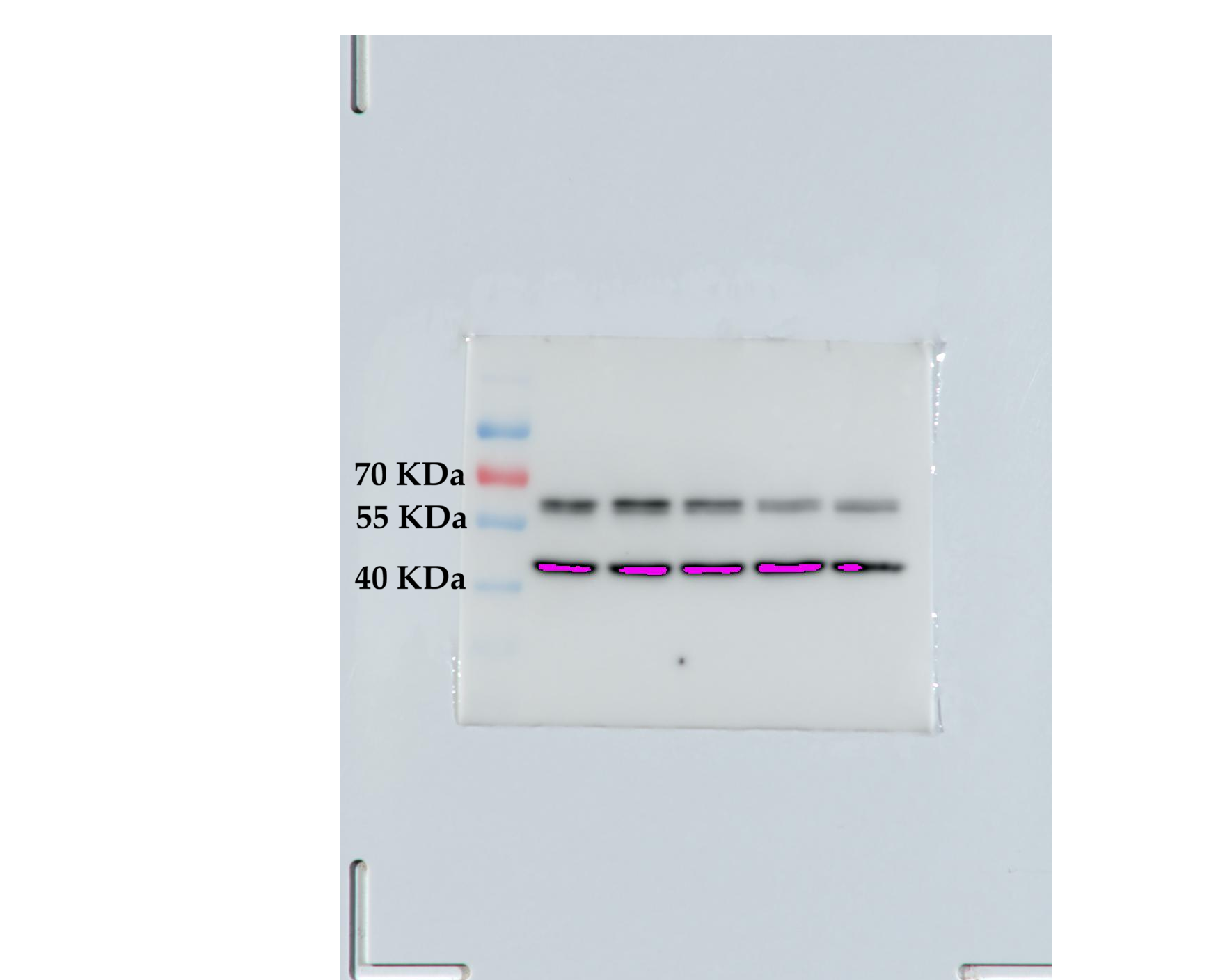

Supplement: Supplementary file 1 [file biomolecules-16-01059-s001.zip › File S1/Figure 6-8-11 Western blot original drawing/Figure 11a/P-Akt/2/p-Akt-2.png]

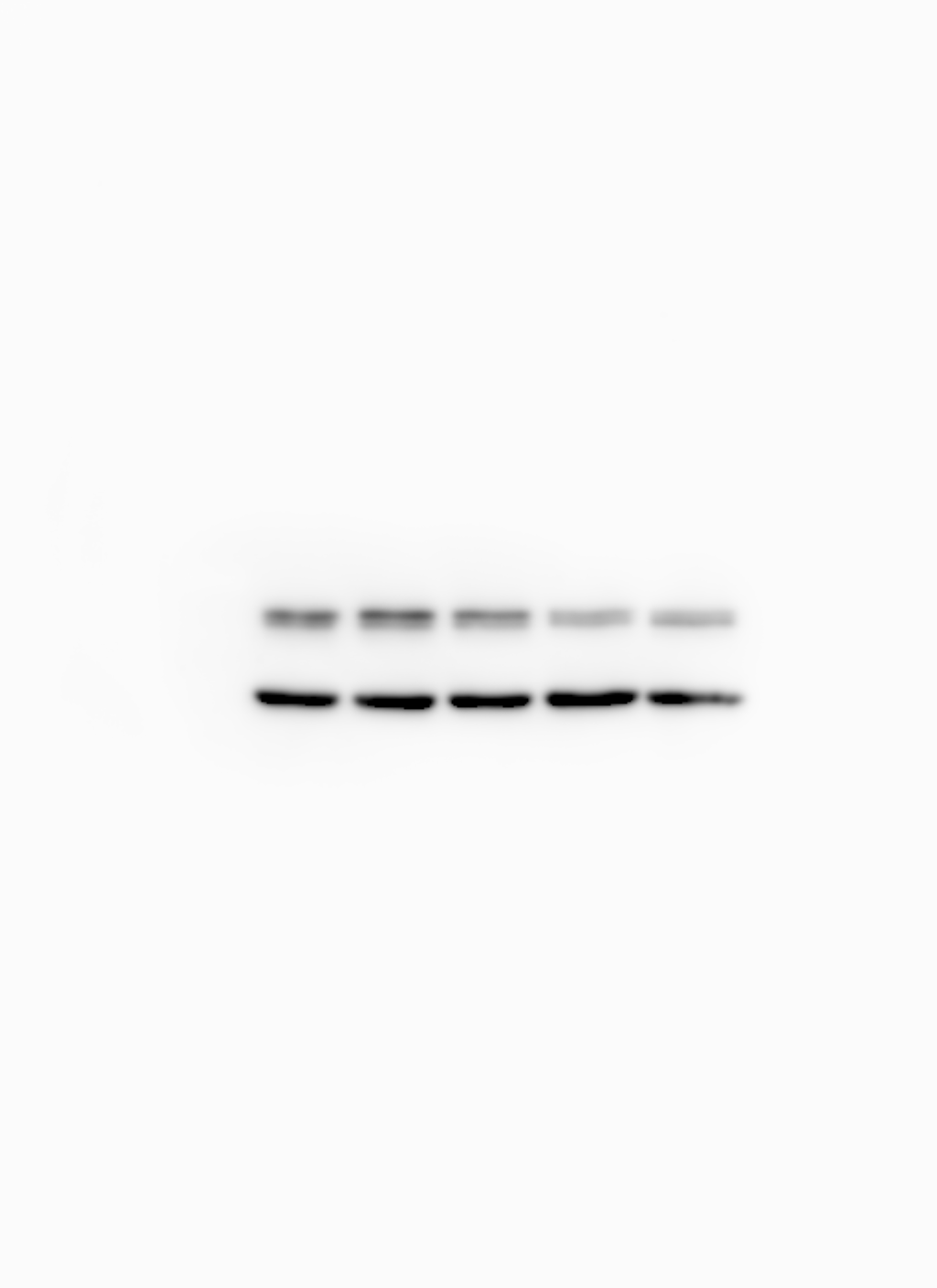

Supplement: Supplementary file 1 [file biomolecules-16-01059-s001.zip › File S1/Figure 6-8-11 Western blot original drawing/Figure 11a/P-Akt/2/p-Akt-2.tif]

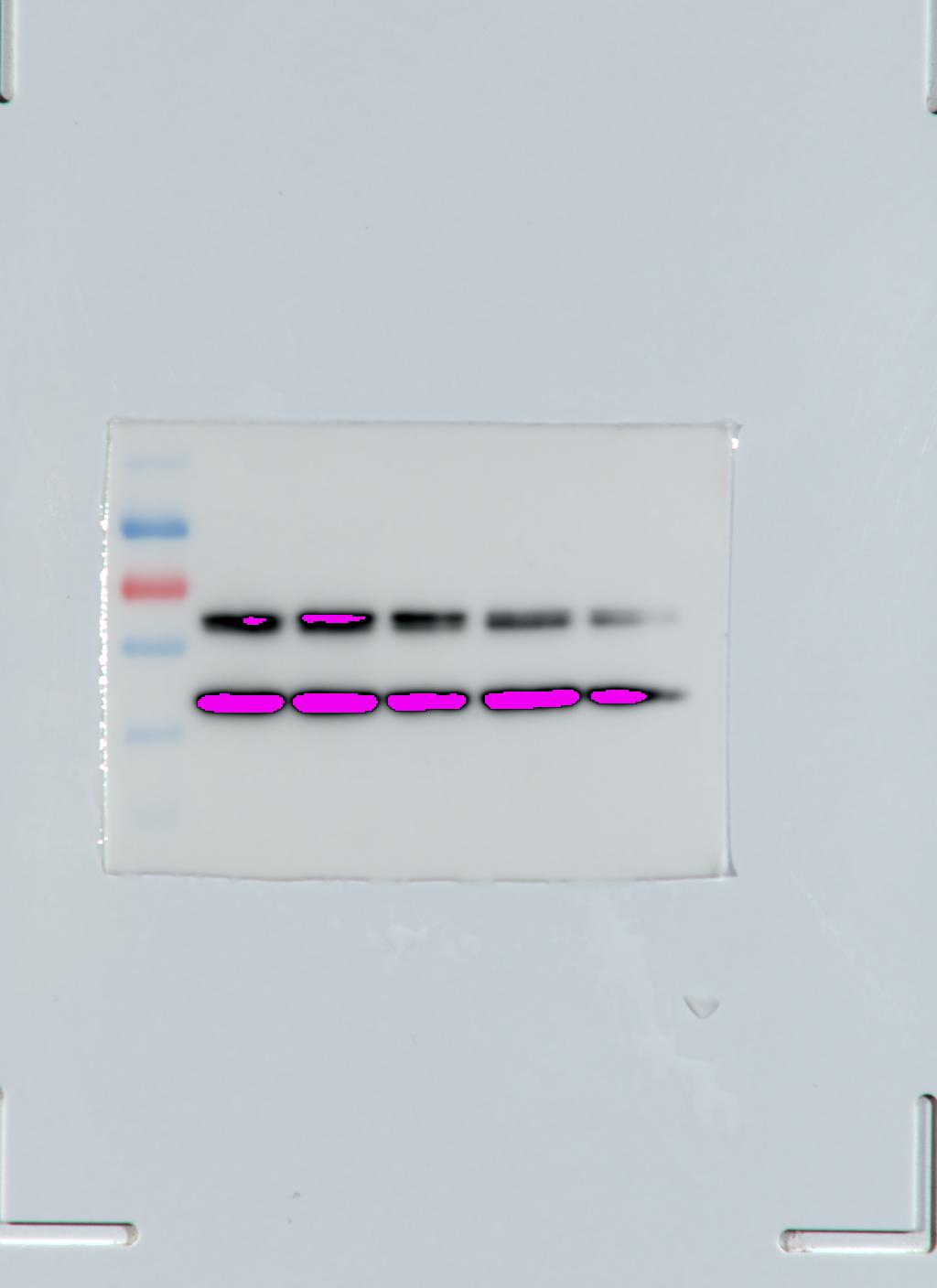

Supplement: Supplementary file 1 [file biomolecules-16-01059-s001.zip › File S1/Figure 6-8-11 Western blot original drawing/Figure 11a/P-Akt/3/p-Akt-3.jpg]

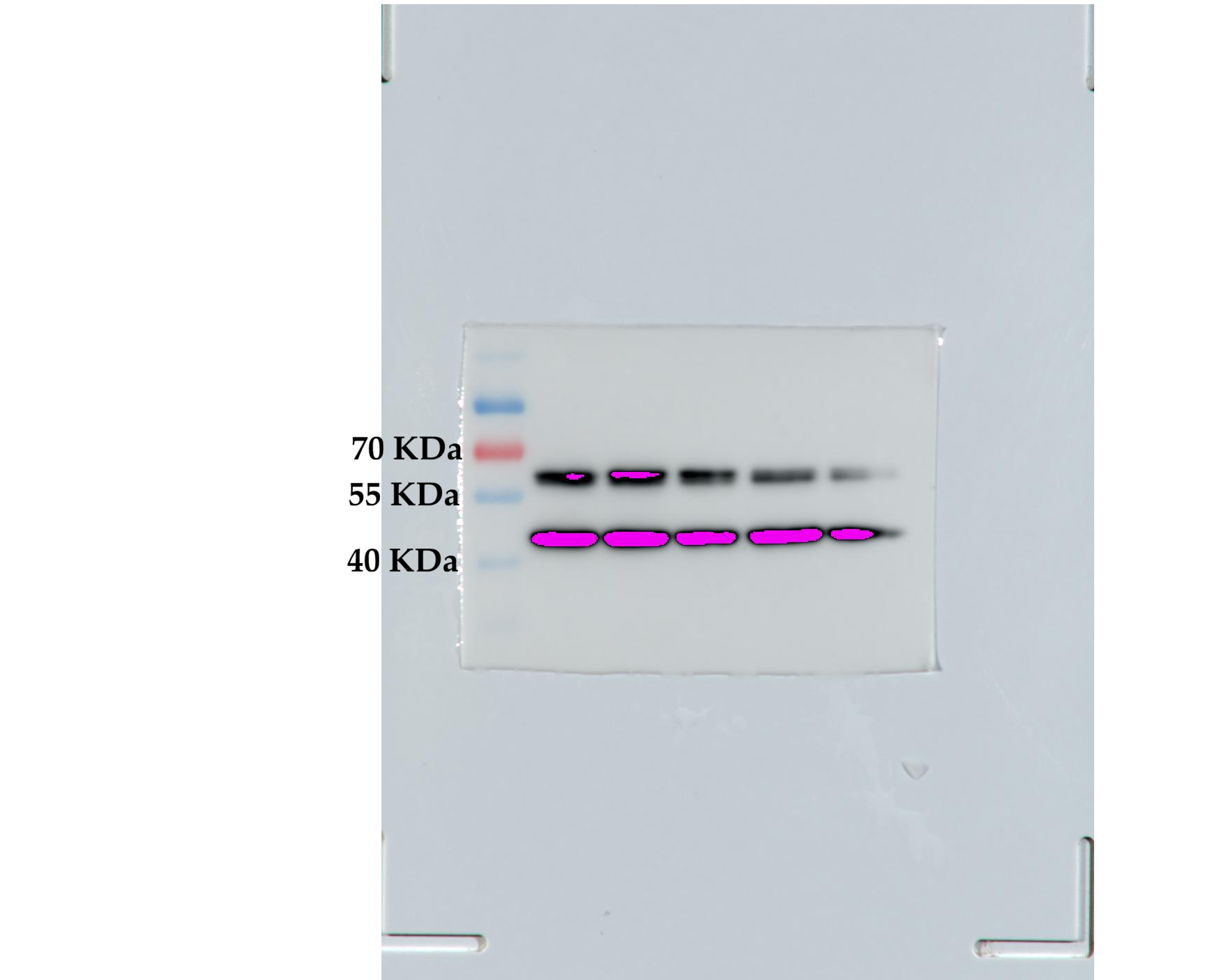

Supplement: Supplementary file 1 [file biomolecules-16-01059-s001.zip › File S1/Figure 6-8-11 Western blot original drawing/Figure 11a/P-Akt/3/p-Akt-3.png]

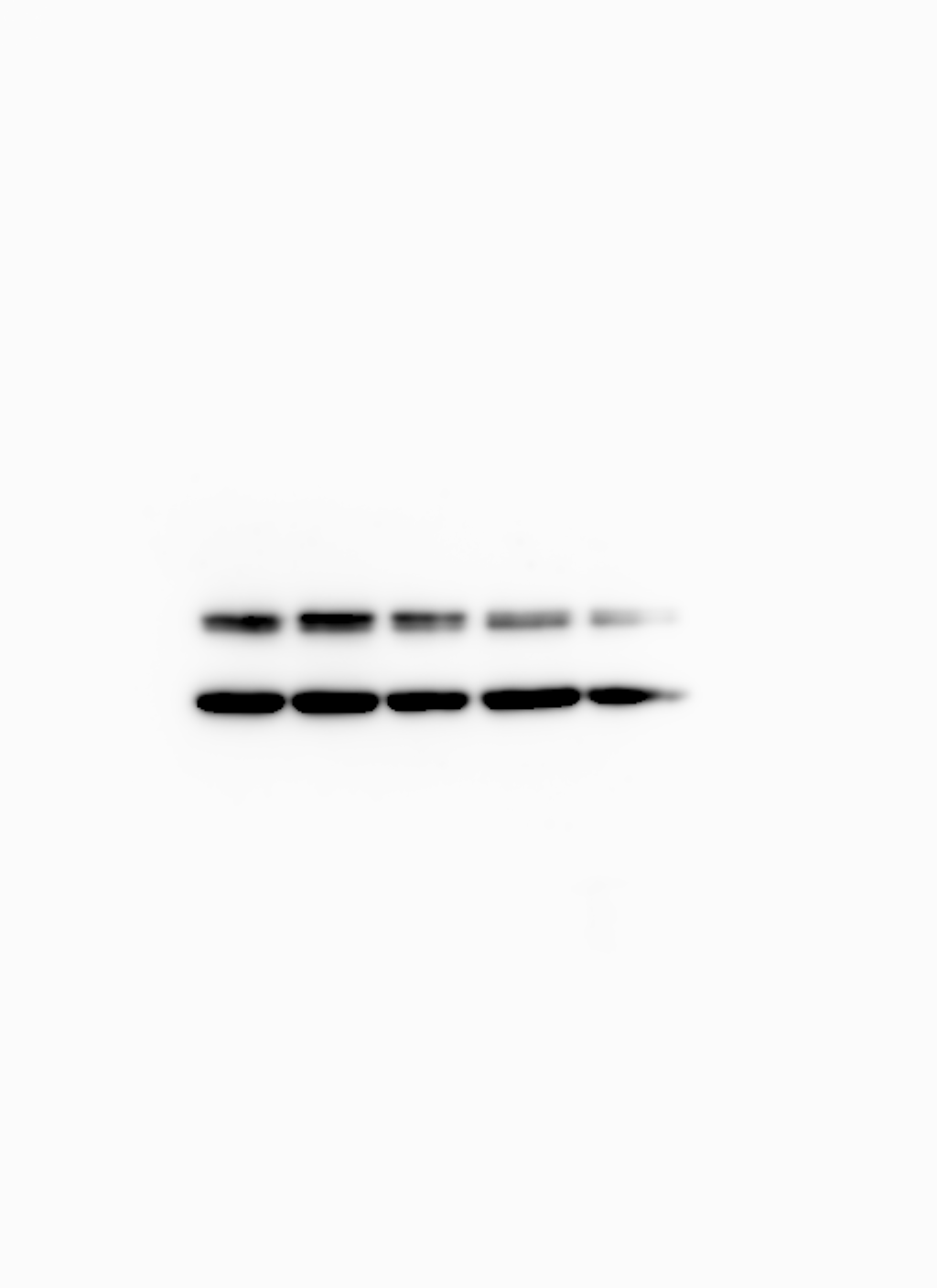

Supplement: Supplementary file 1 [file biomolecules-16-01059-s001.zip › File S1/Figure 6-8-11 Western blot original drawing/Figure 11a/P-Akt/3/p-Akt-3.tif]

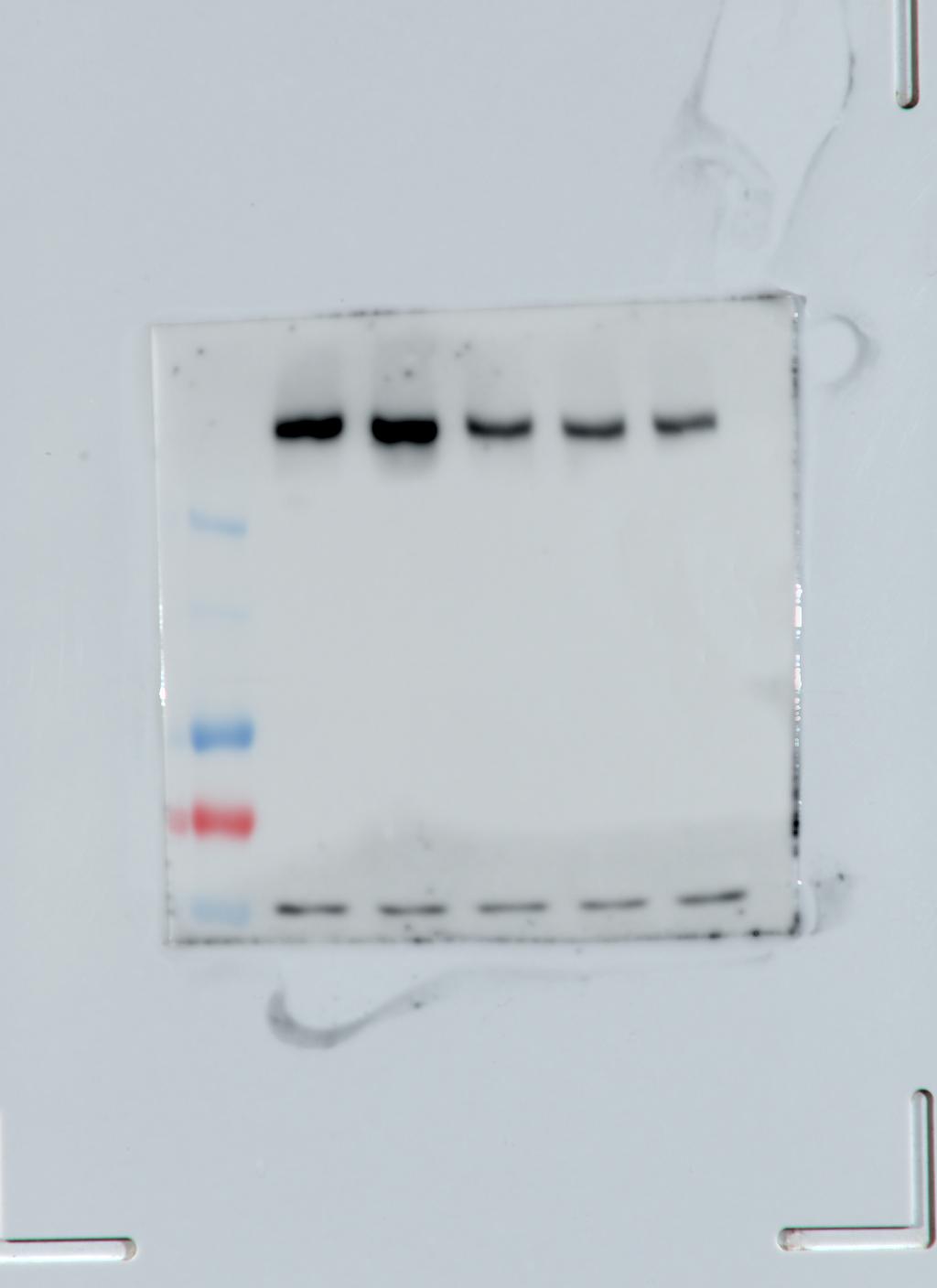

Supplement: Supplementary file 1 [file biomolecules-16-01059-s001.zip › File S1/Figure 6-8-11 Western blot original drawing/Figure 11a/P-mTOR/1/P-mTOR-1.jpg]

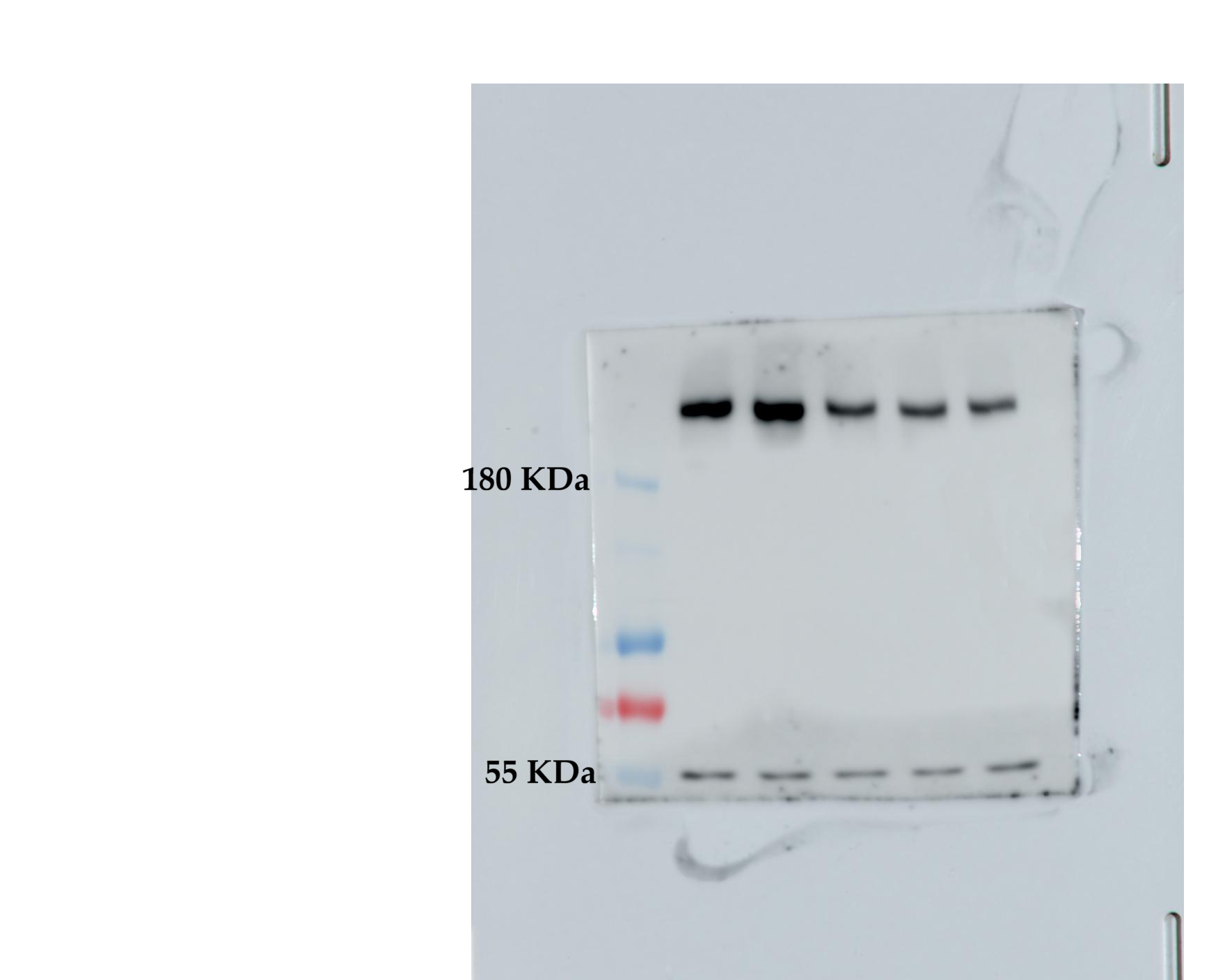

Supplement: Supplementary file 1 [file biomolecules-16-01059-s001.zip › File S1/Figure 6-8-11 Western blot original drawing/Figure 11a/P-mTOR/1/P-mTOR-1.png]

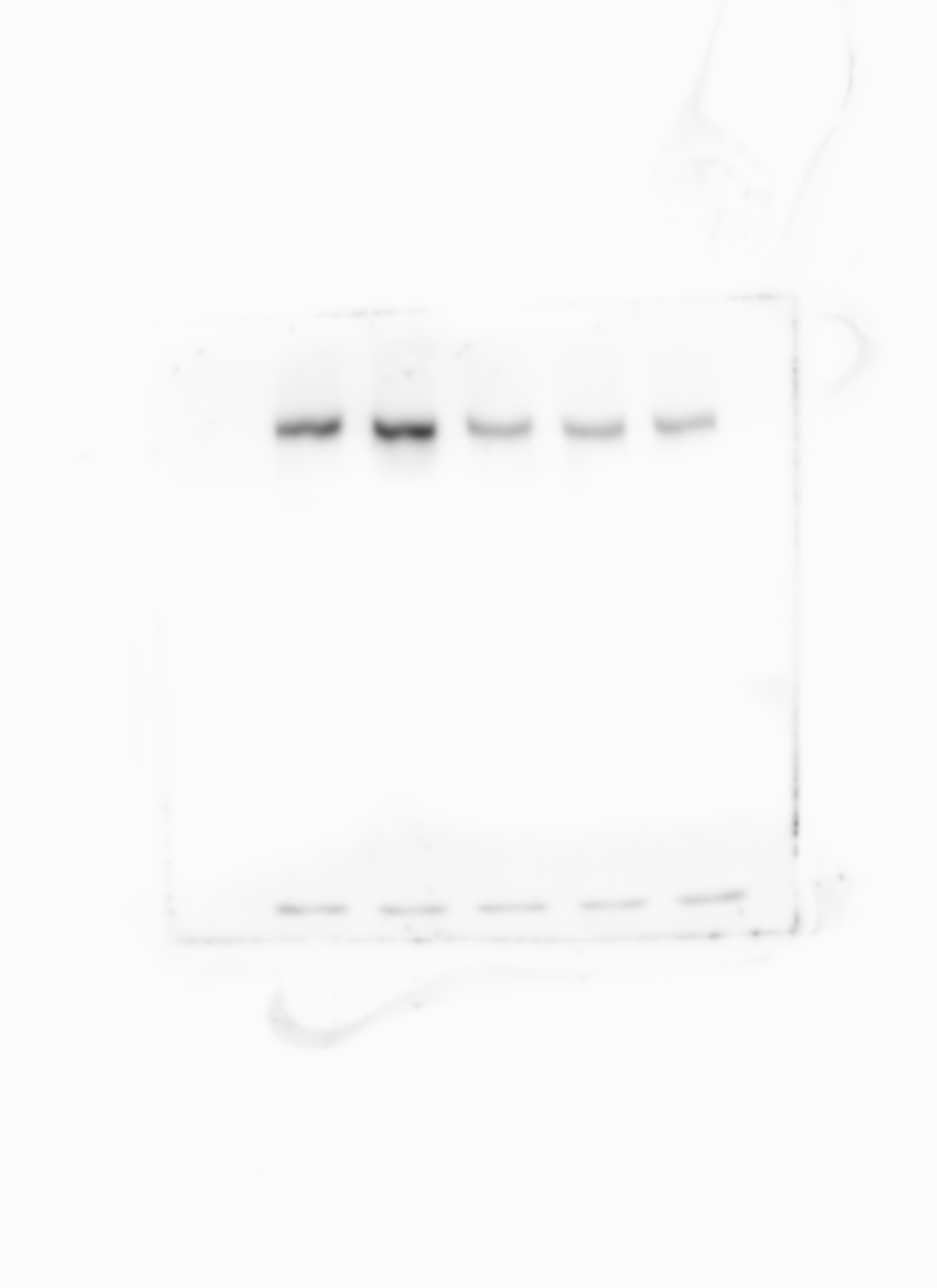

Supplement: Supplementary file 1 [file biomolecules-16-01059-s001.zip › File S1/Figure 6-8-11 Western blot original drawing/Figure 11a/P-mTOR/1/P-mTOR-1.tif]

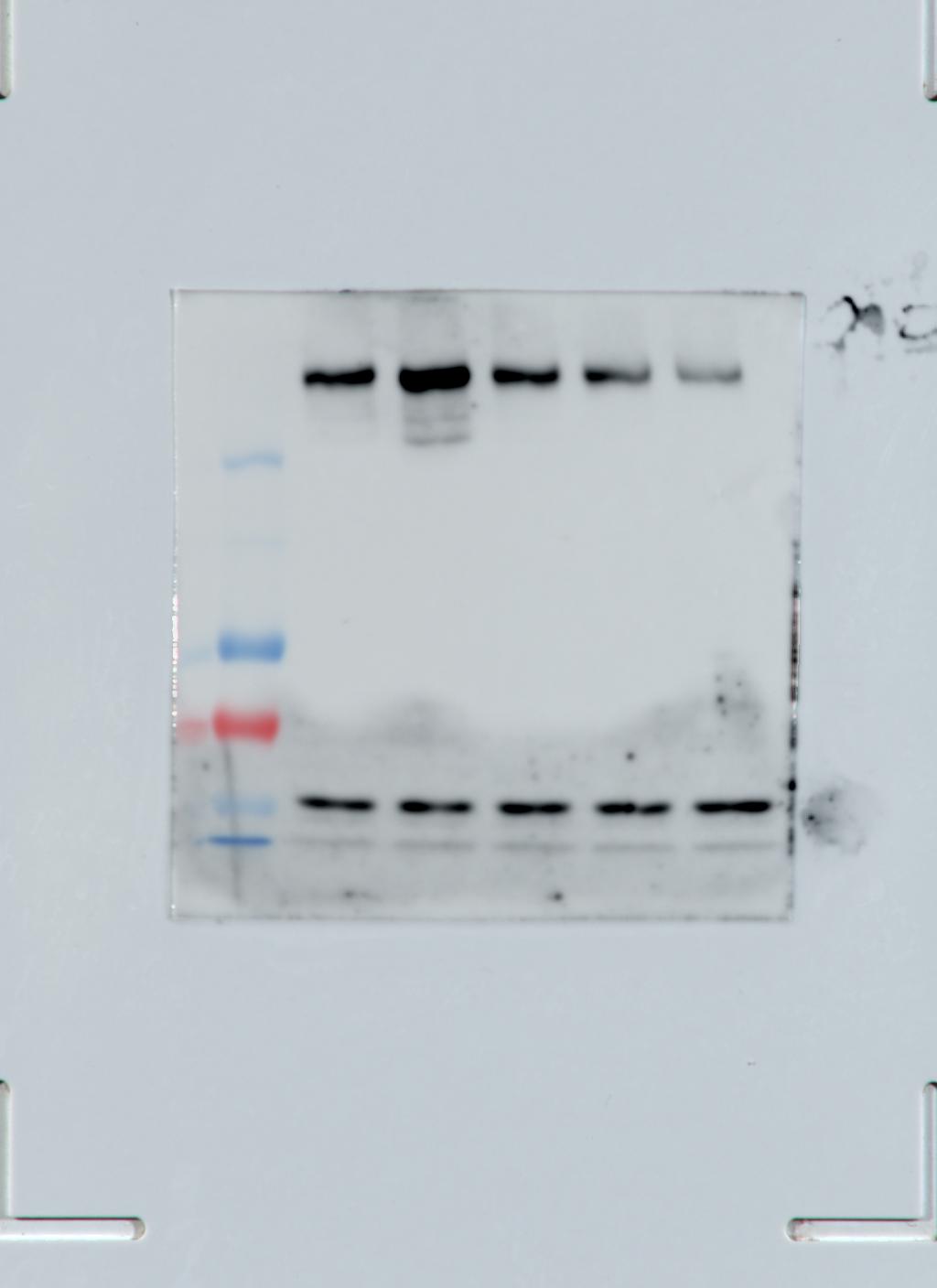

Supplement: Supplementary file 1 [file biomolecules-16-01059-s001.zip › File S1/Figure 6-8-11 Western blot original drawing/Figure 11a/P-mTOR/2/p-mTOR-2.jpg]

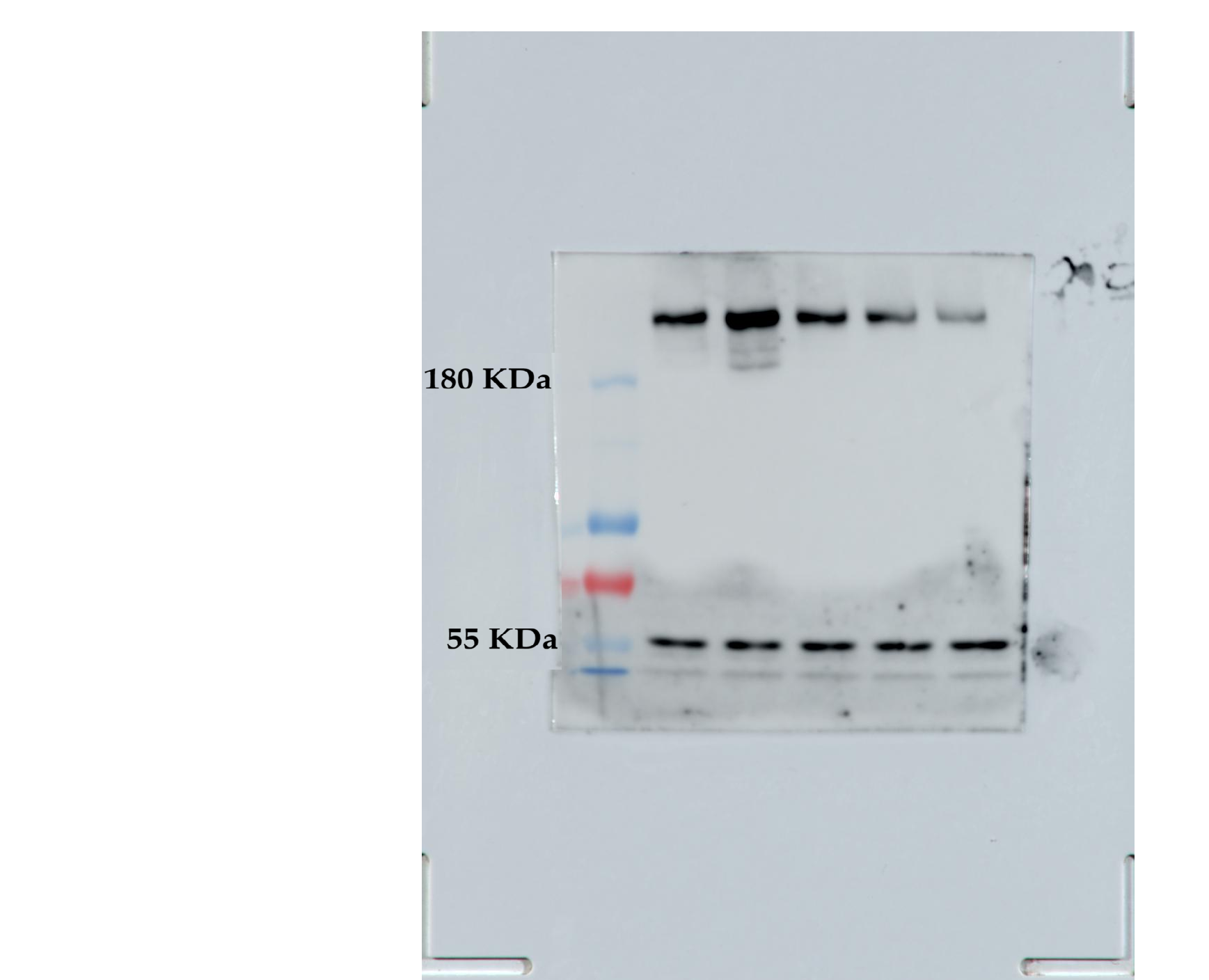

Supplement: Supplementary file 1 [file biomolecules-16-01059-s001.zip › File S1/Figure 6-8-11 Western blot original drawing/Figure 11a/P-mTOR/2/P-mTOR-2.png]

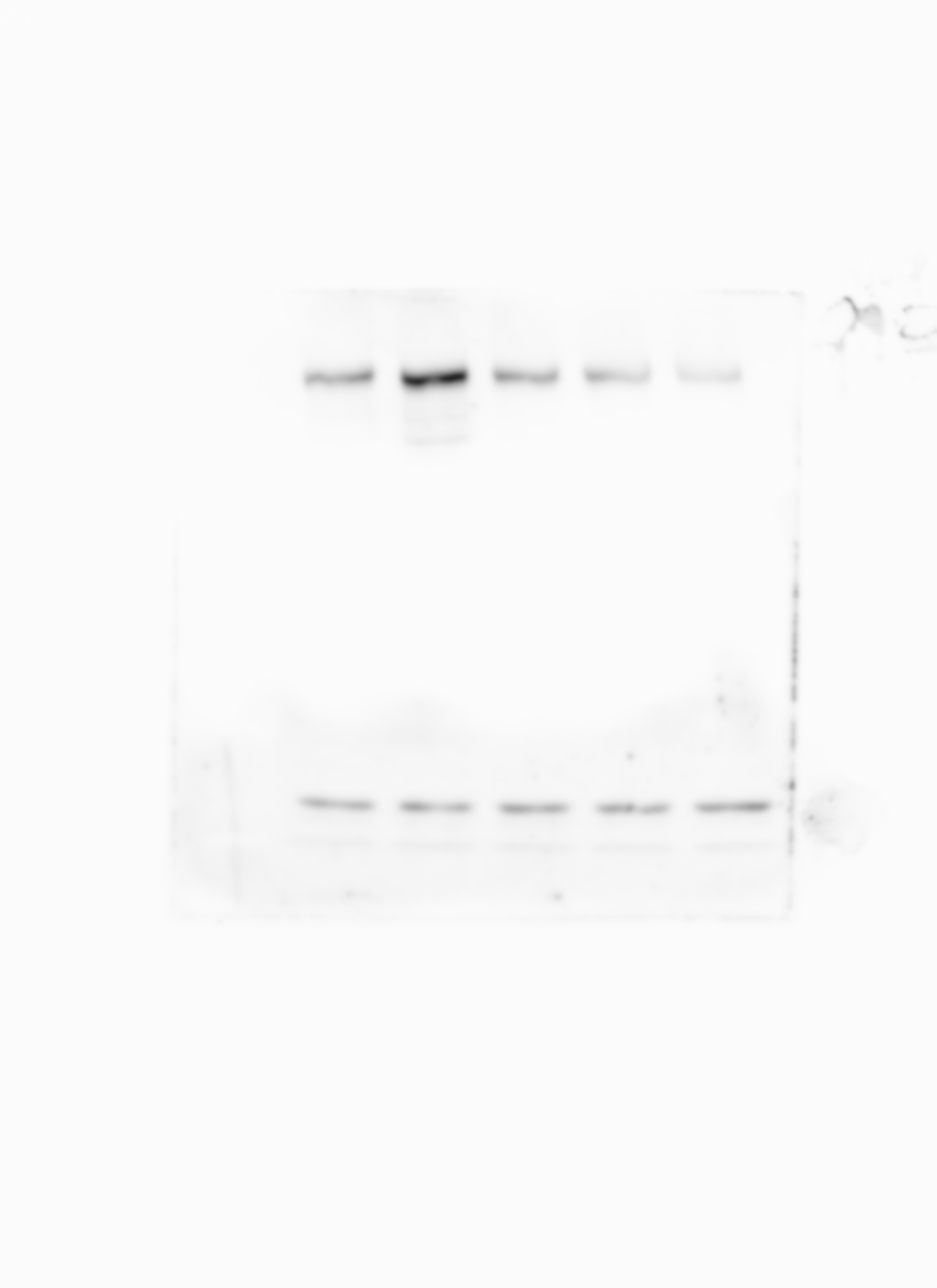

Supplement: Supplementary file 1 [file biomolecules-16-01059-s001.zip › File S1/Figure 6-8-11 Western blot original drawing/Figure 11a/P-mTOR/2/p-mTOR-2.tif]

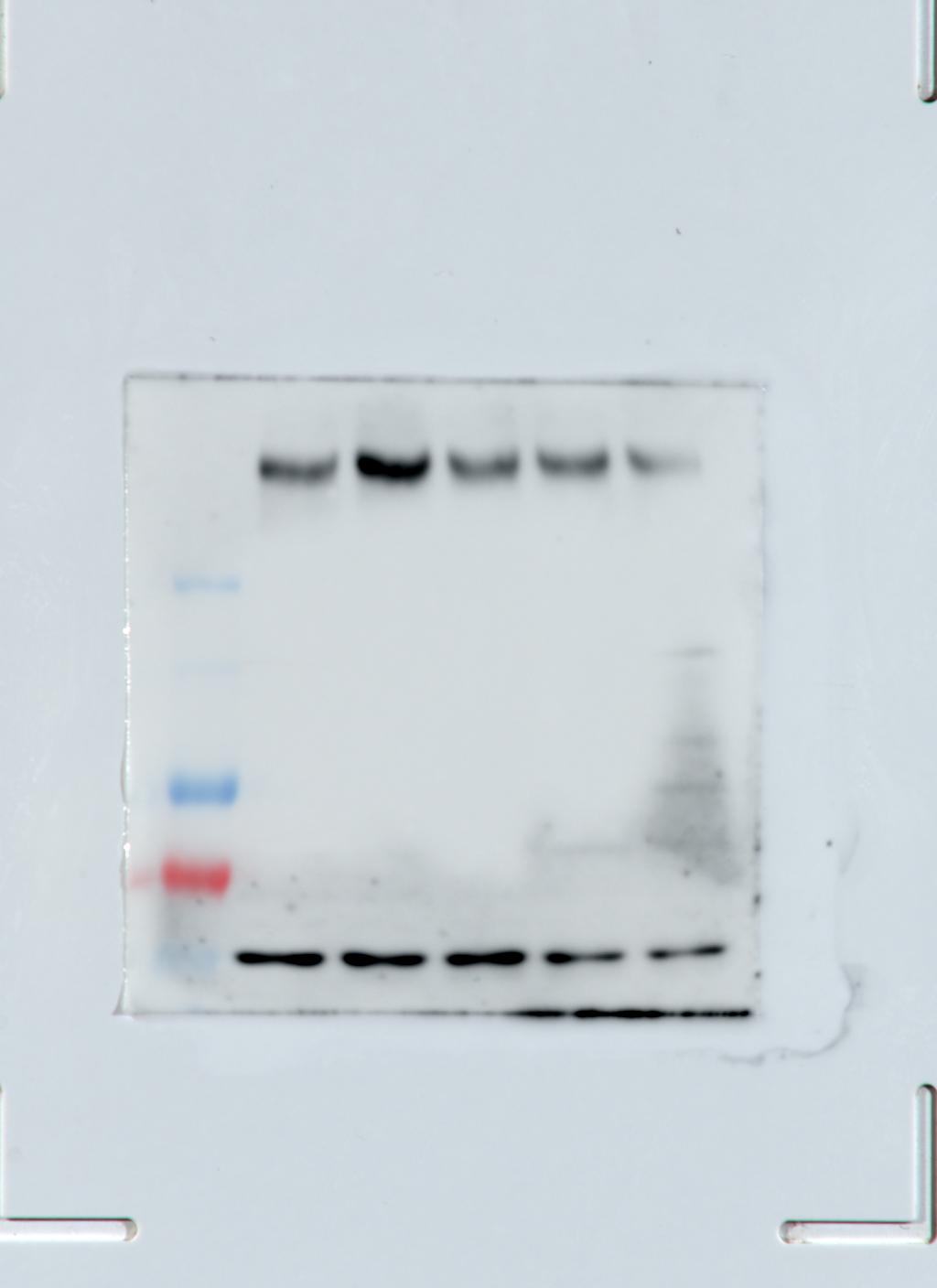

Supplement: Supplementary file 1 [file biomolecules-16-01059-s001.zip › File S1/Figure 6-8-11 Western blot original drawing/Figure 11a/P-mTOR/3/p-mTOR-3.jpg]

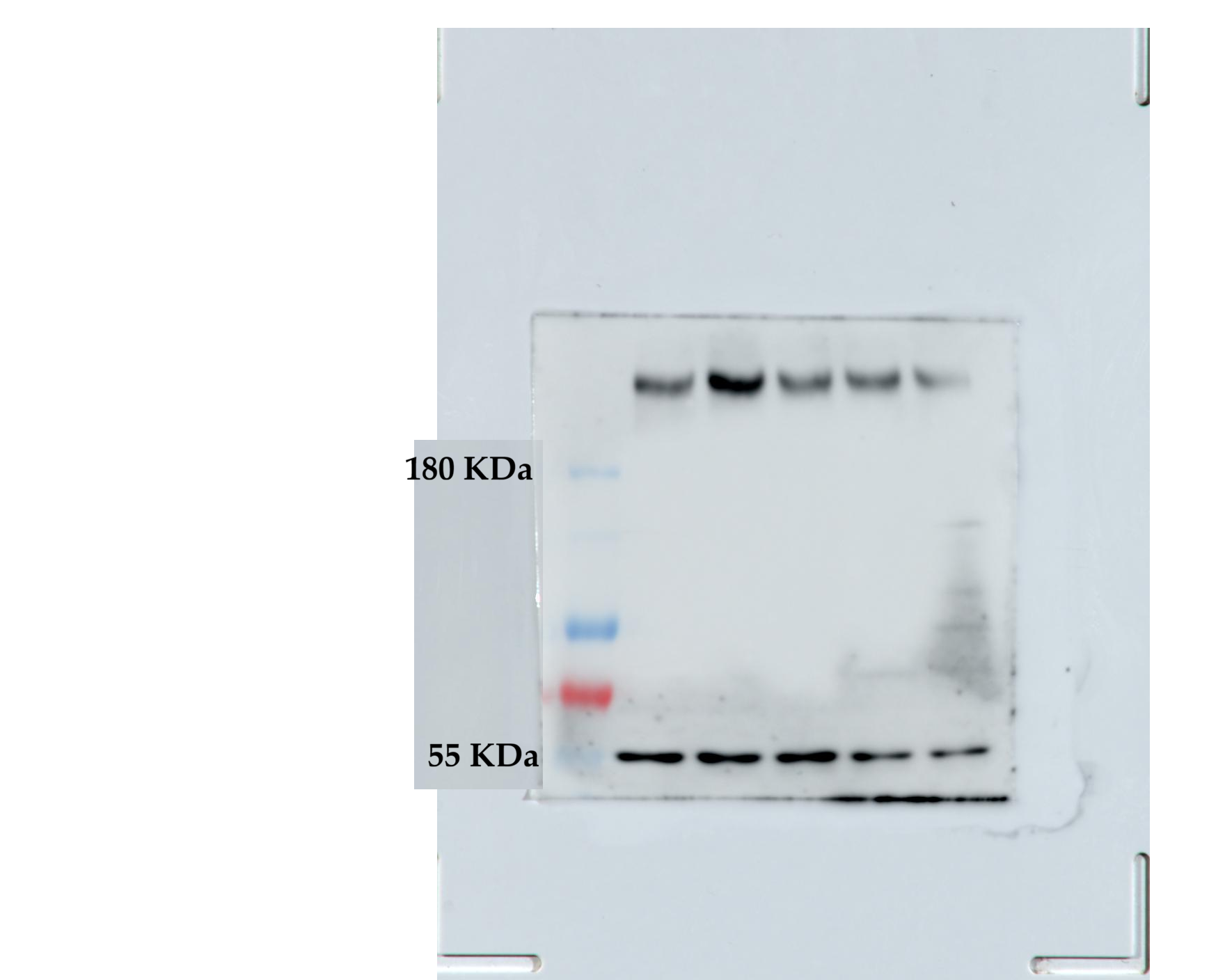

Supplement: Supplementary file 1 [file biomolecules-16-01059-s001.zip › File S1/Figure 6-8-11 Western blot original drawing/Figure 11a/P-mTOR/3/P-mTOR-3.png]

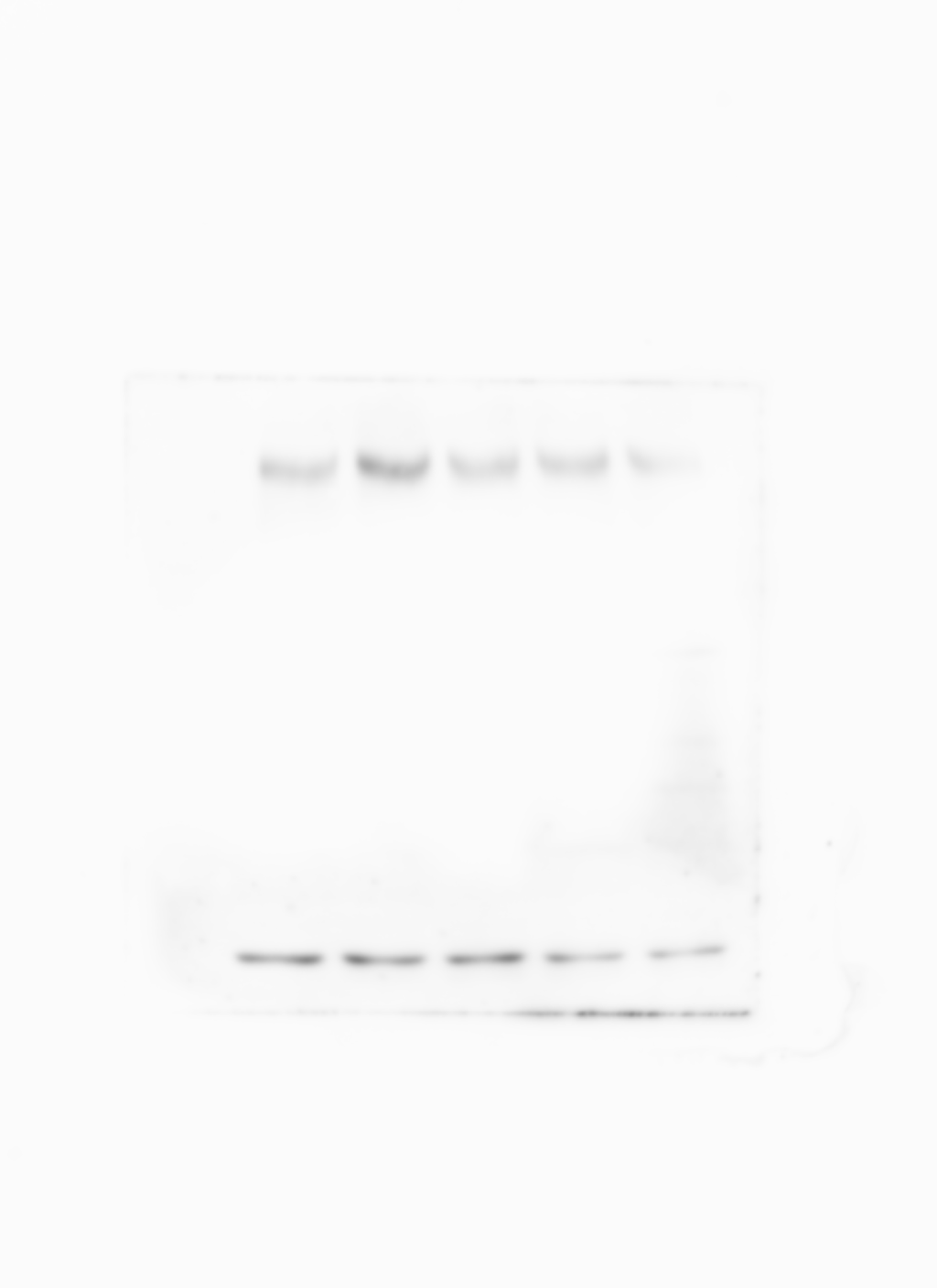

Supplement: Supplementary file 1 [file biomolecules-16-01059-s001.zip › File S1/Figure 6-8-11 Western blot original drawing/Figure 11a/P-mTOR/3/p-mTOR-3.tif]

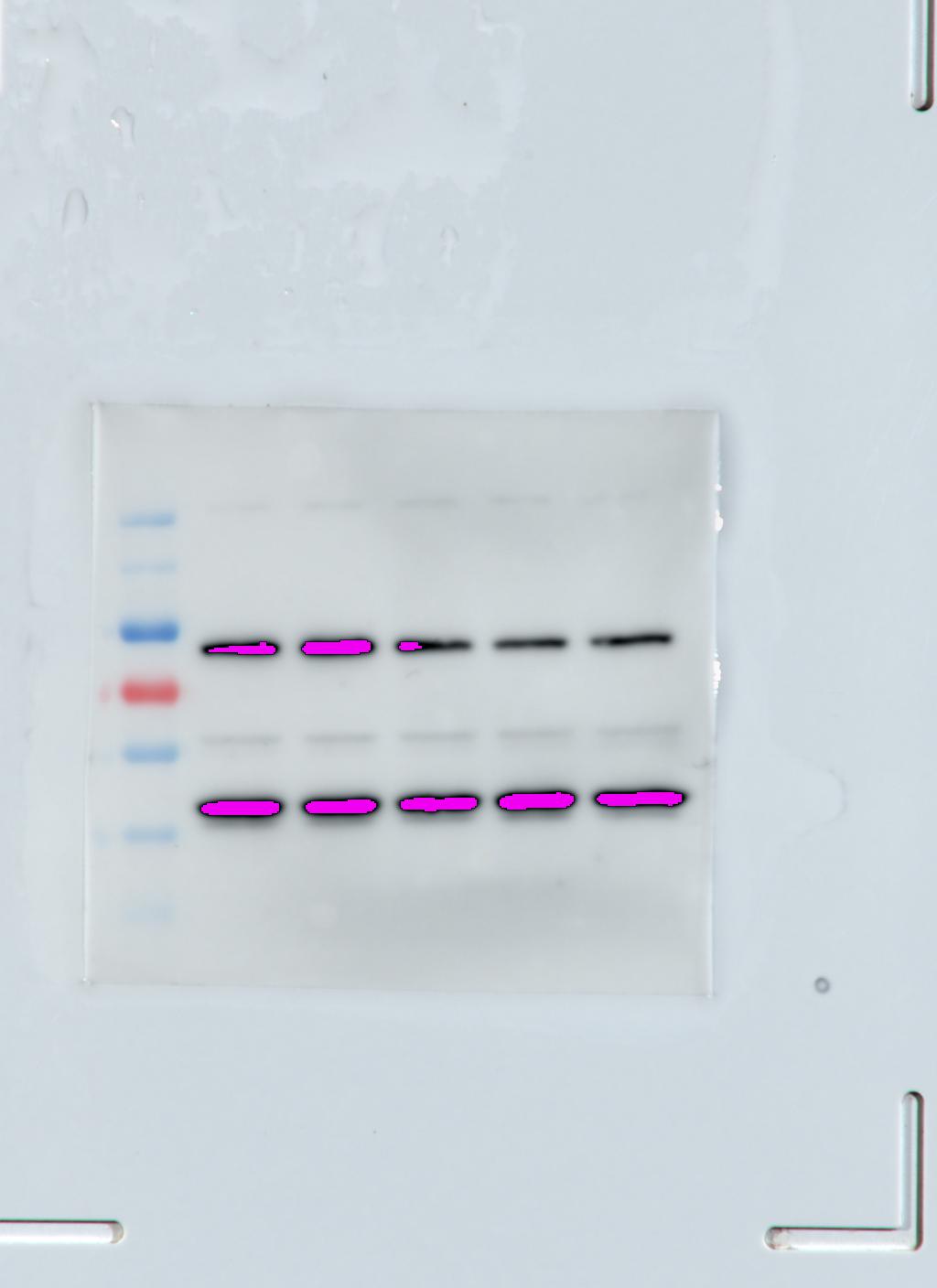

Supplement: Supplementary file 1 [file biomolecules-16-01059-s001.zip › File S1/Figure 6-8-11 Western blot original drawing/Figure 11a/P-PI3K/1/P-PI3K-1.jpg]

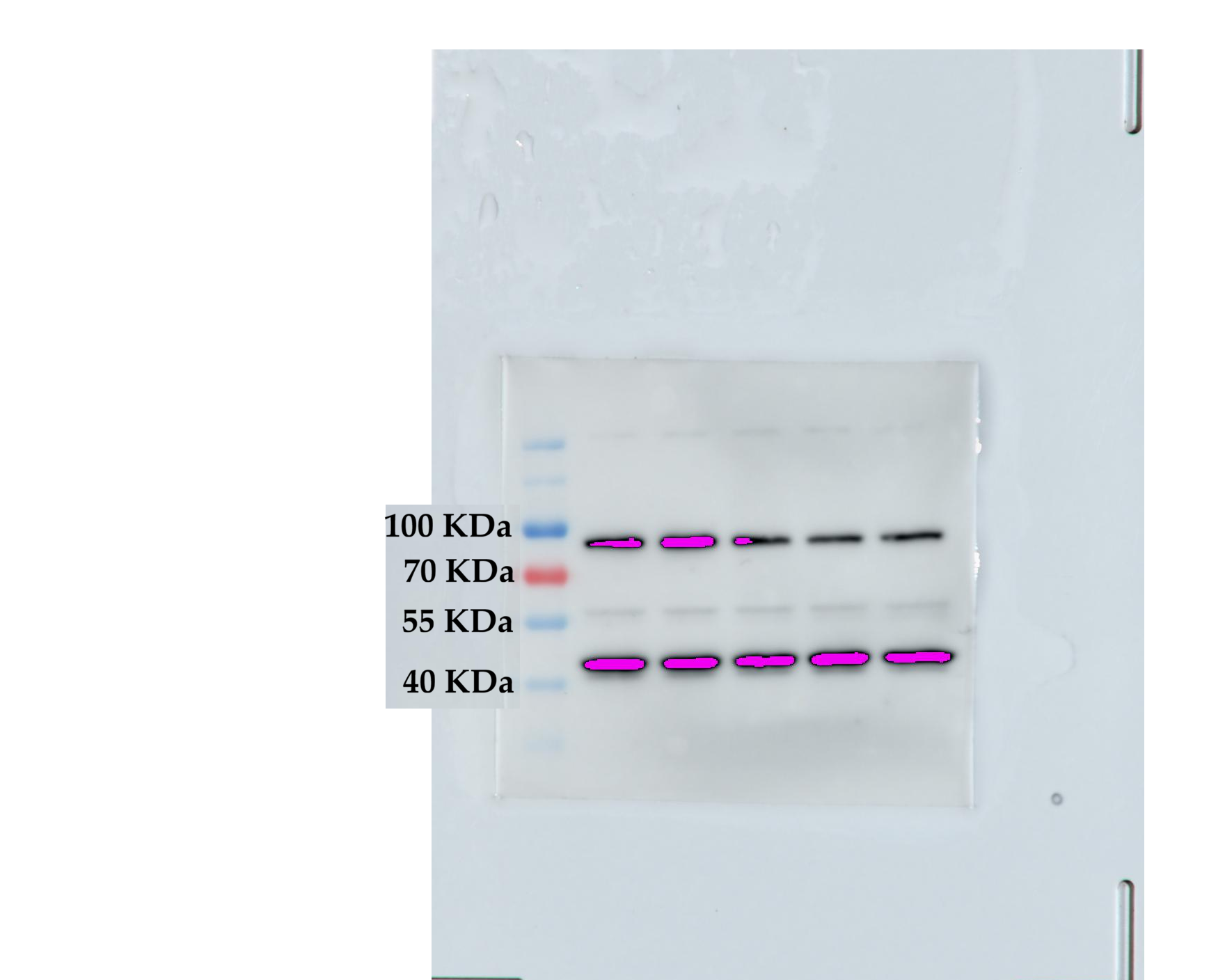

Supplement: Supplementary file 1 [file biomolecules-16-01059-s001.zip › File S1/Figure 6-8-11 Western blot original drawing/Figure 11a/P-PI3K/1/P-PI3K-1.png]

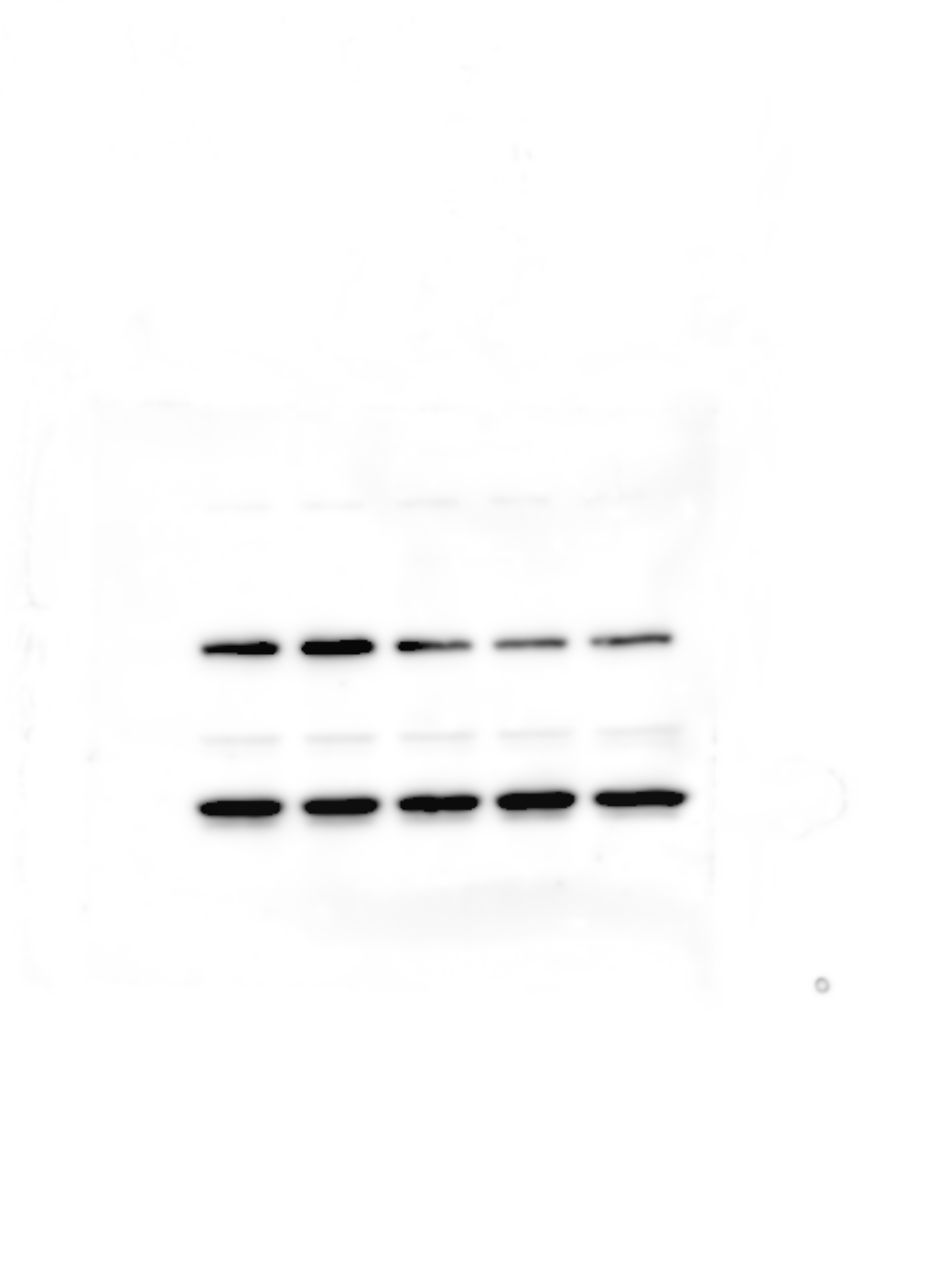

Supplement: Supplementary file 1 [file biomolecules-16-01059-s001.zip › File S1/Figure 6-8-11 Western blot original drawing/Figure 11a/P-PI3K/1/P-PI3K-1.tif]

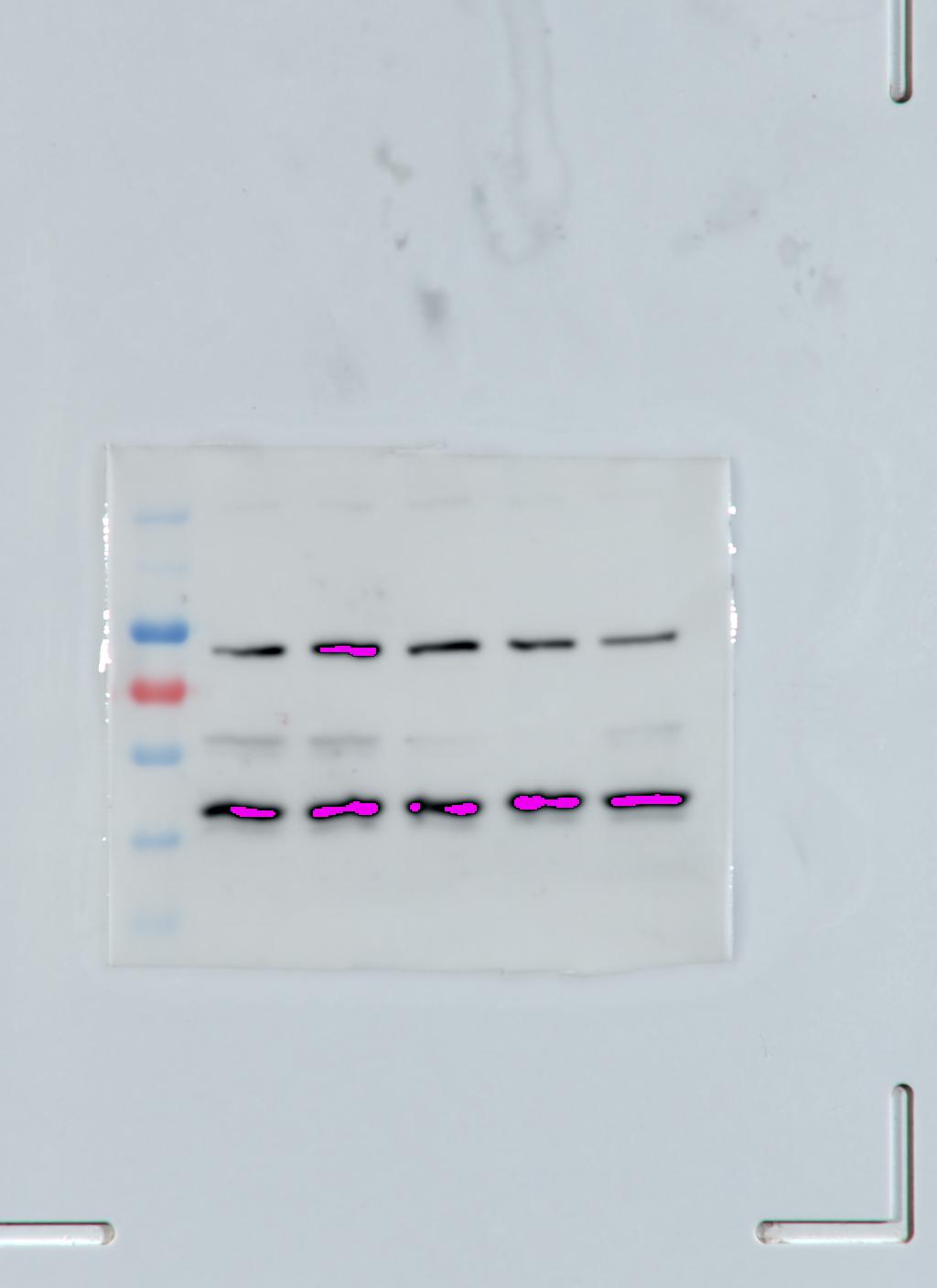

Supplement: Supplementary file 1 [file biomolecules-16-01059-s001.zip › File S1/Figure 6-8-11 Western blot original drawing/Figure 11a/P-PI3K/2/P-PI3K-2.jpg]

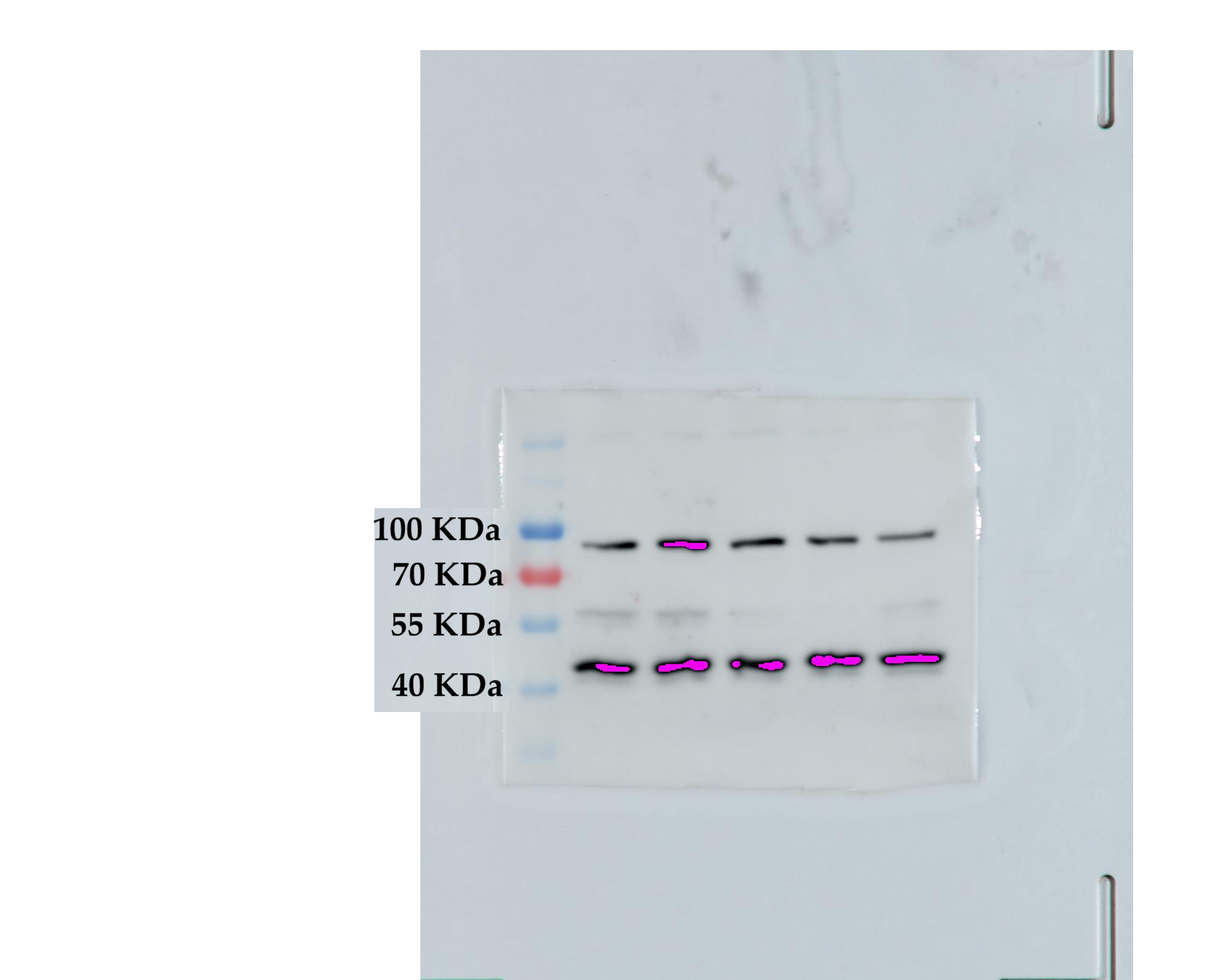

Supplement: Supplementary file 1 [file biomolecules-16-01059-s001.zip › File S1/Figure 6-8-11 Western blot original drawing/Figure 11a/P-PI3K/2/P-PI3K-2.png]

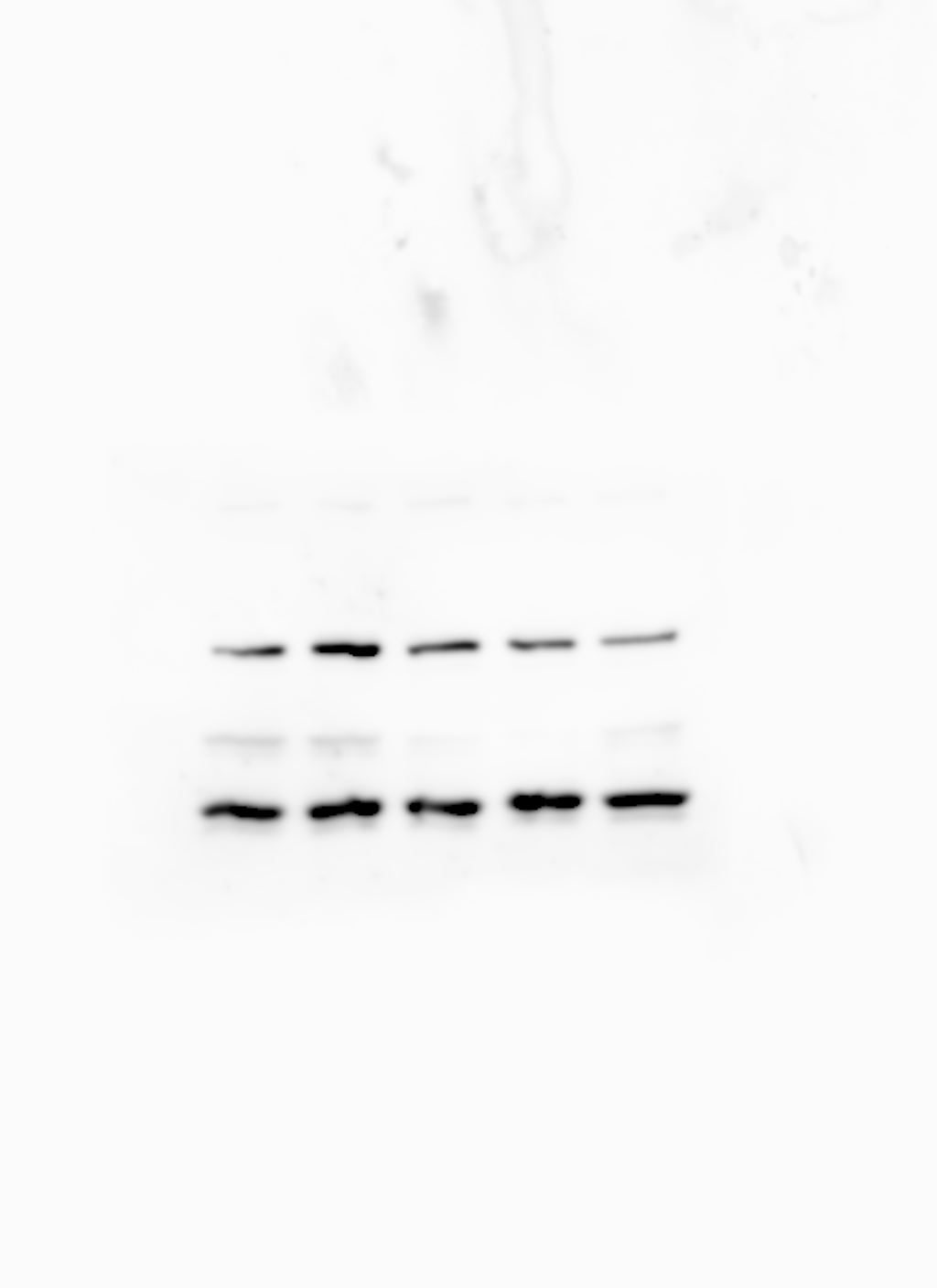

Supplement: Supplementary file 1 [file biomolecules-16-01059-s001.zip › File S1/Figure 6-8-11 Western blot original drawing/Figure 11a/P-PI3K/2/P-PI3K-2.tif]

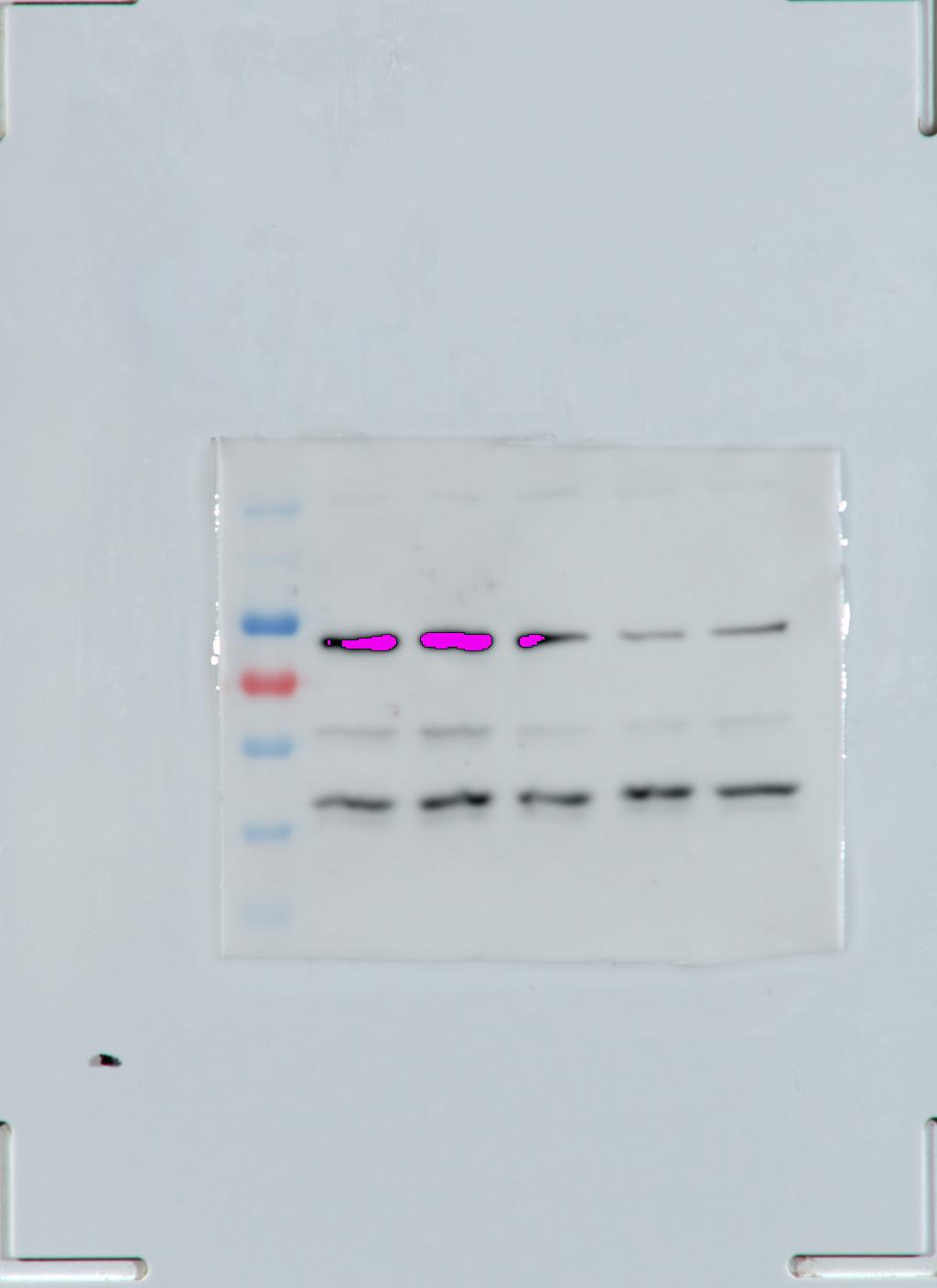

Supplement: Supplementary file 1 [file biomolecules-16-01059-s001.zip › File S1/Figure 6-8-11 Western blot original drawing/Figure 11a/P-PI3K/3/P-PI3K-3.jpg]

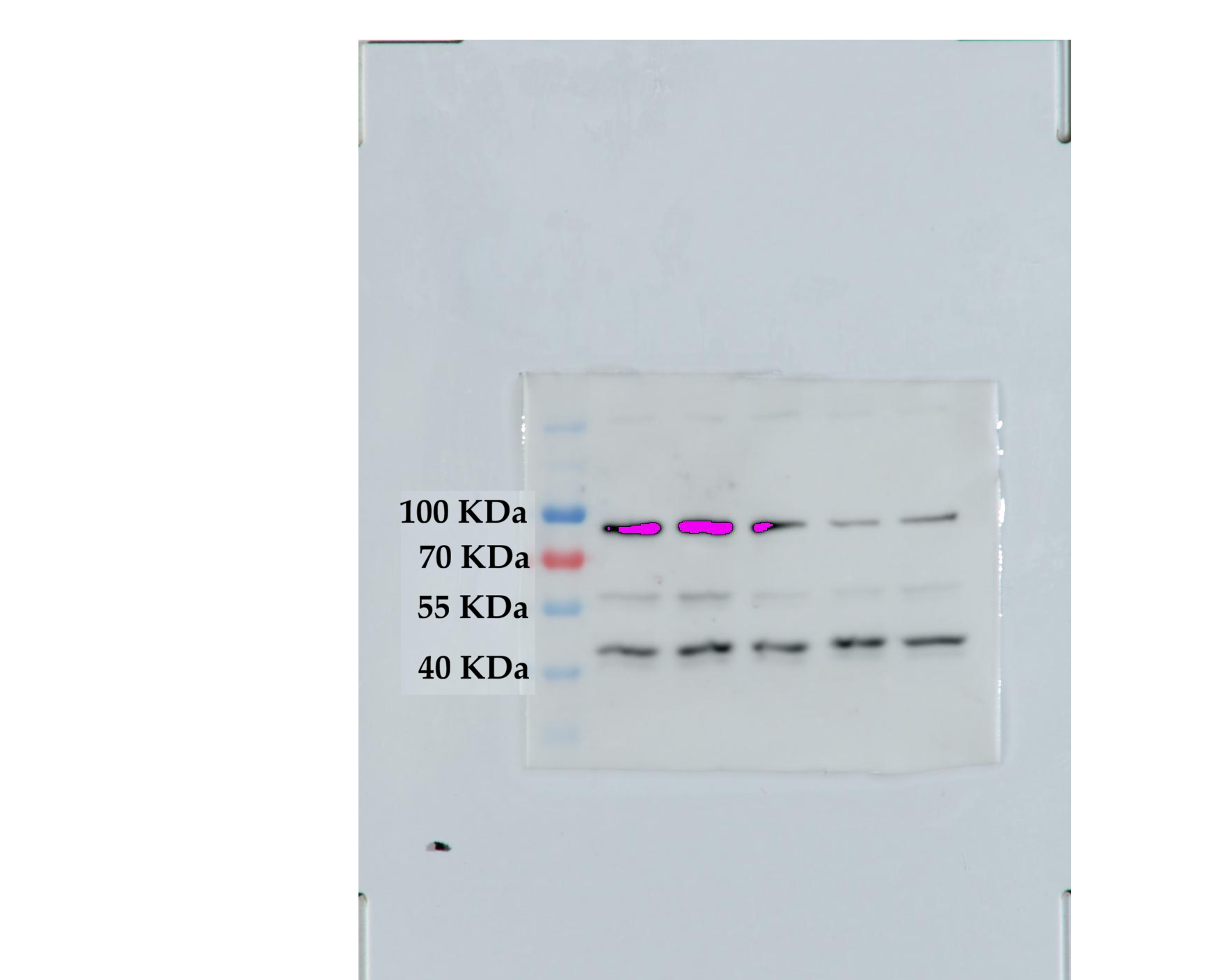

Supplement: Supplementary file 1 [file biomolecules-16-01059-s001.zip › File S1/Figure 6-8-11 Western blot original drawing/Figure 11a/P-PI3K/3/P-PI3K-3.png]

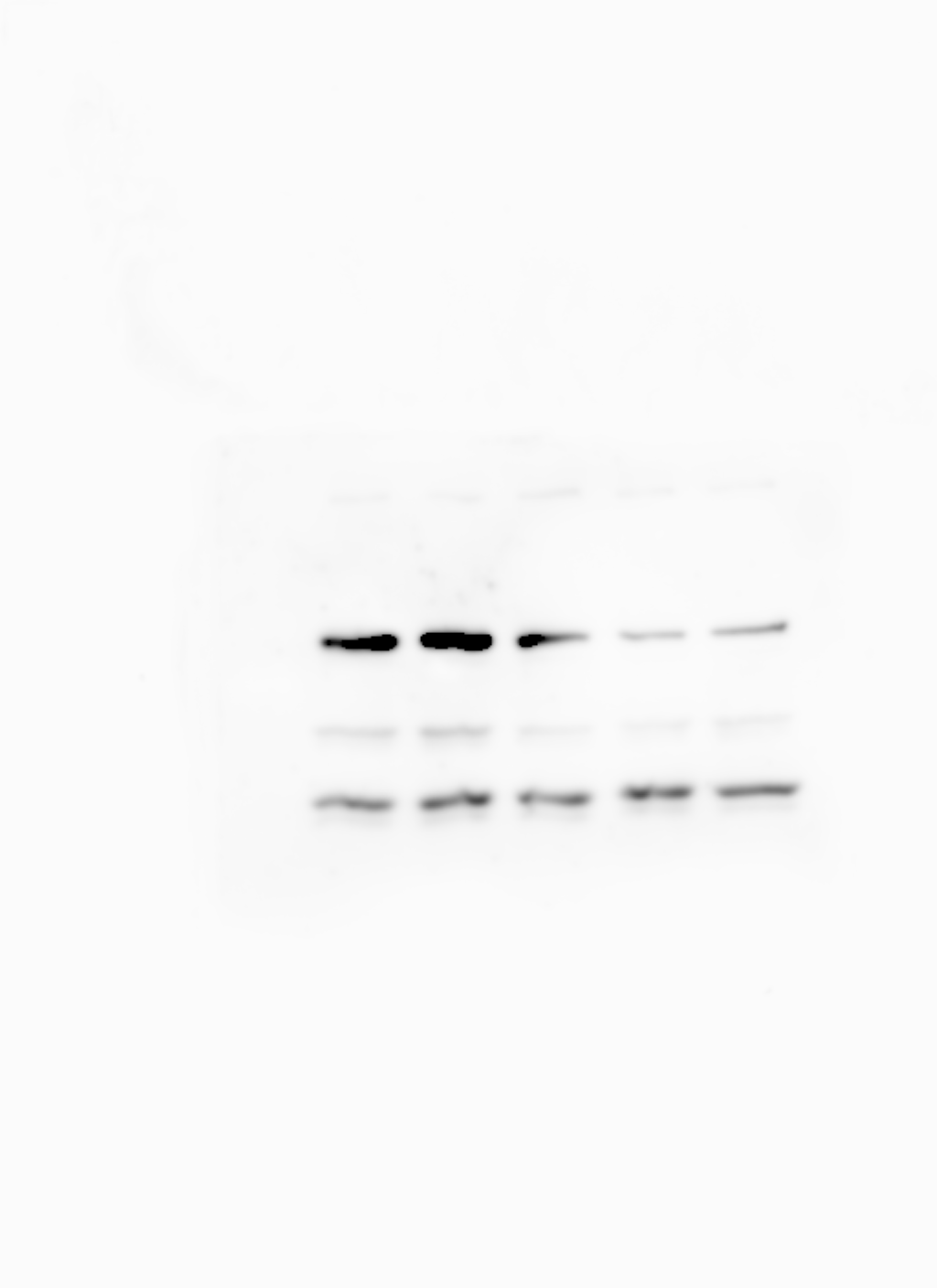

Supplement: Supplementary file 1 [file biomolecules-16-01059-s001.zip › File S1/Figure 6-8-11 Western blot original drawing/Figure 11a/P-PI3K/3/P-PI3K-3.tif]

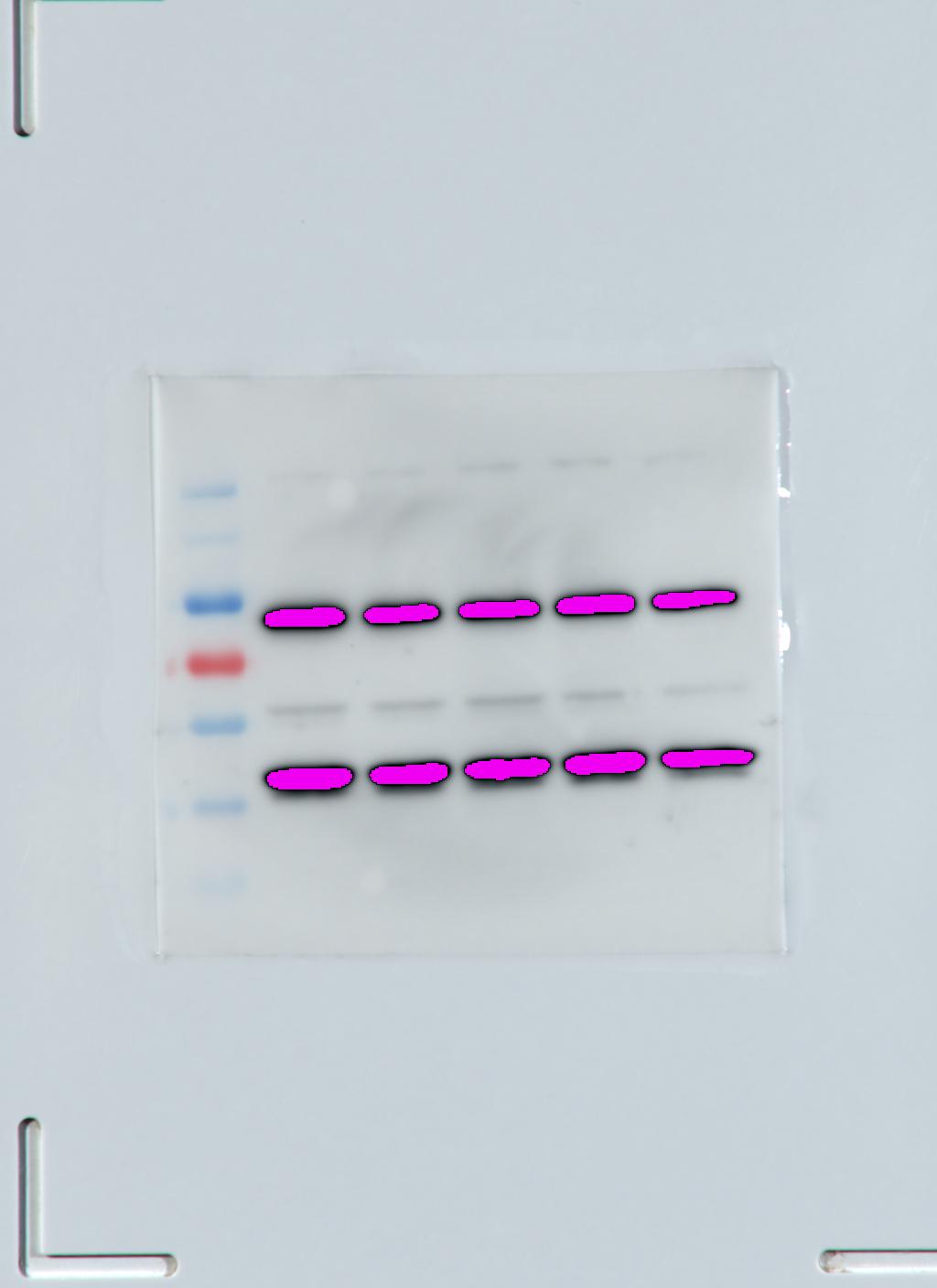

Supplement: Supplementary file 1 [file biomolecules-16-01059-s001.zip › File S1/Figure 6-8-11 Western blot original drawing/Figure 11a/PI3K/1/PI3K-1.jpg]

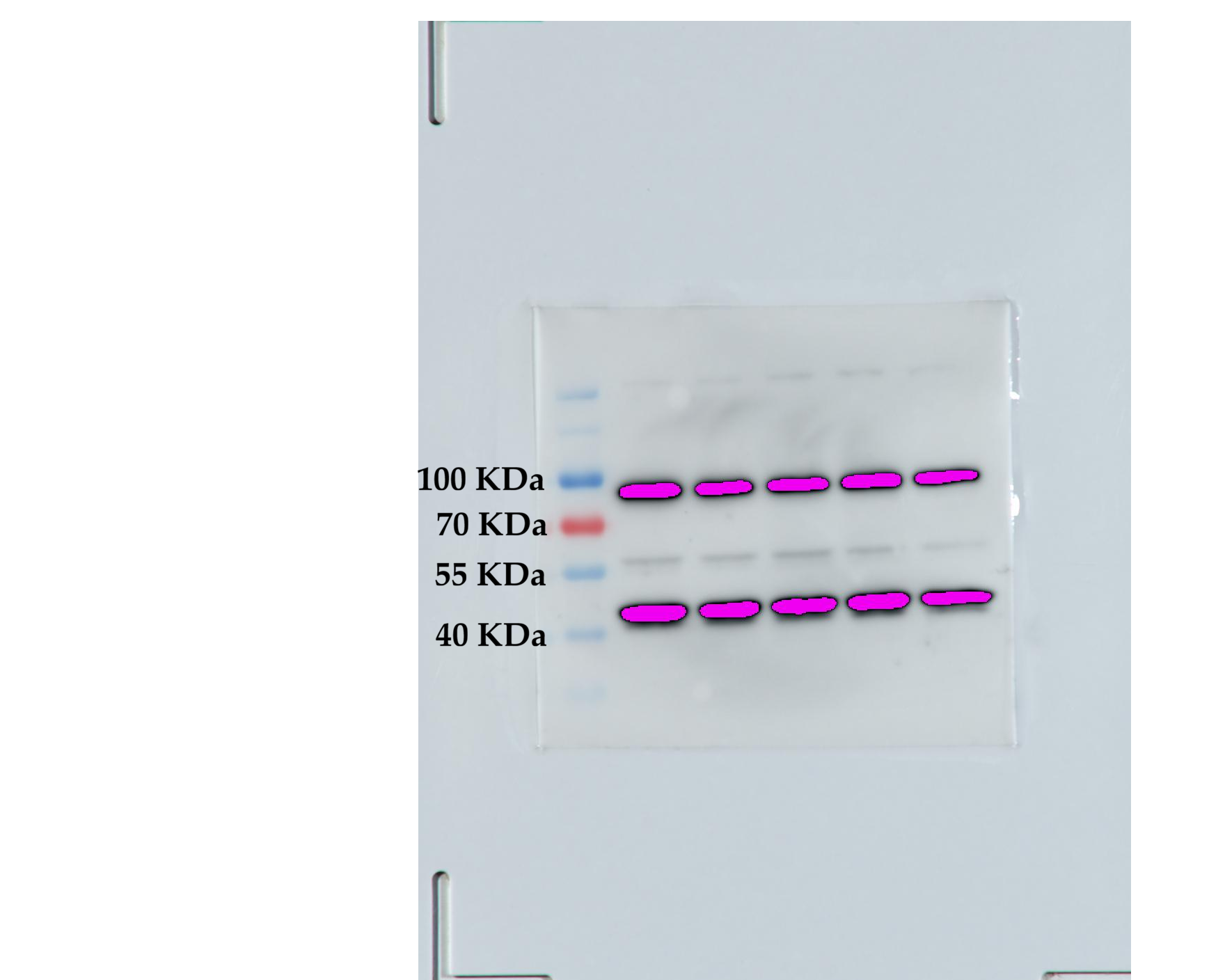

Supplement: Supplementary file 1 [file biomolecules-16-01059-s001.zip › File S1/Figure 6-8-11 Western blot original drawing/Figure 11a/PI3K/1/PI3K-1.png]

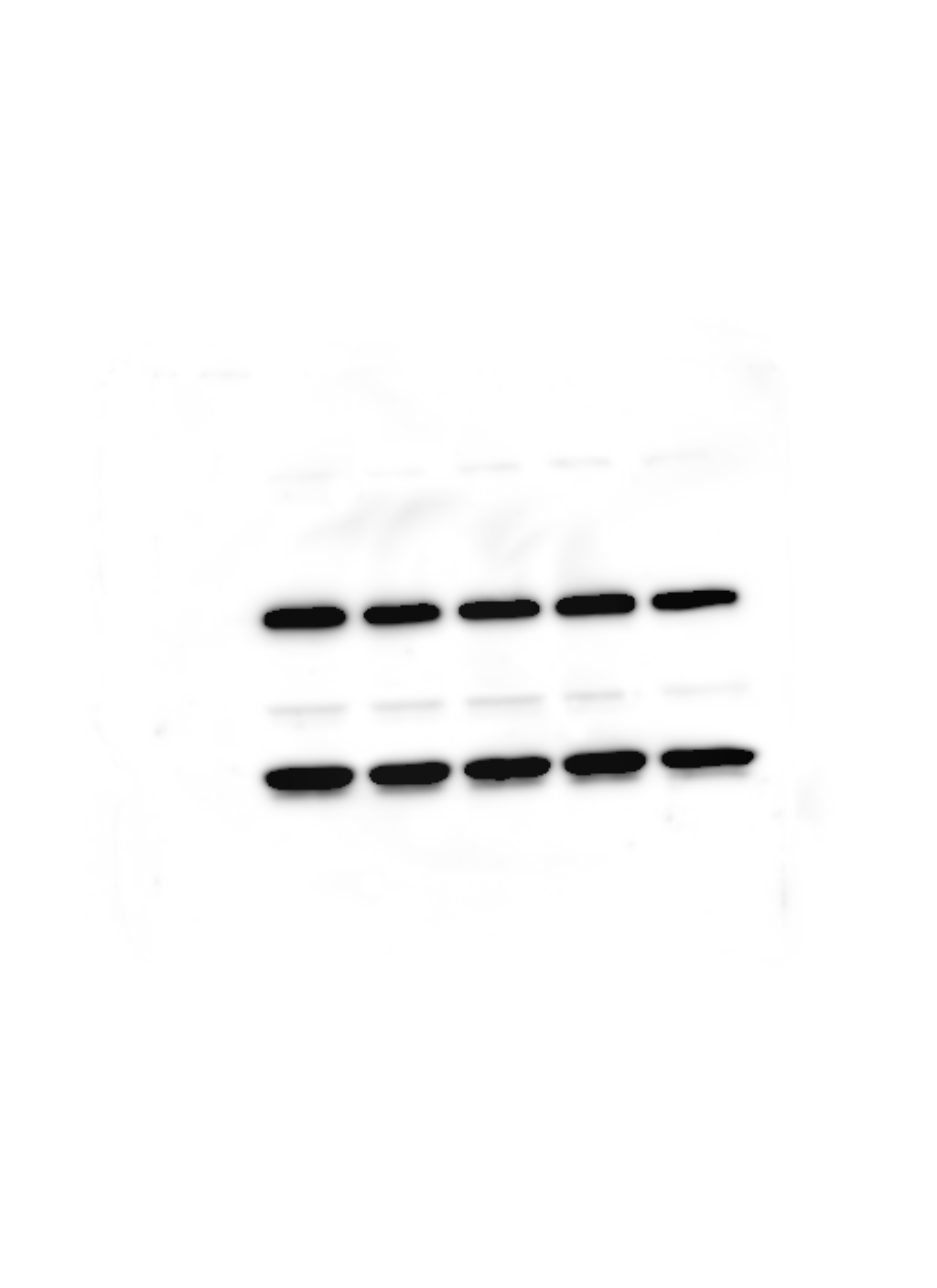

Supplement: Supplementary file 1 [file biomolecules-16-01059-s001.zip › File S1/Figure 6-8-11 Western blot original drawing/Figure 11a/PI3K/1/PI3K-1.tif]

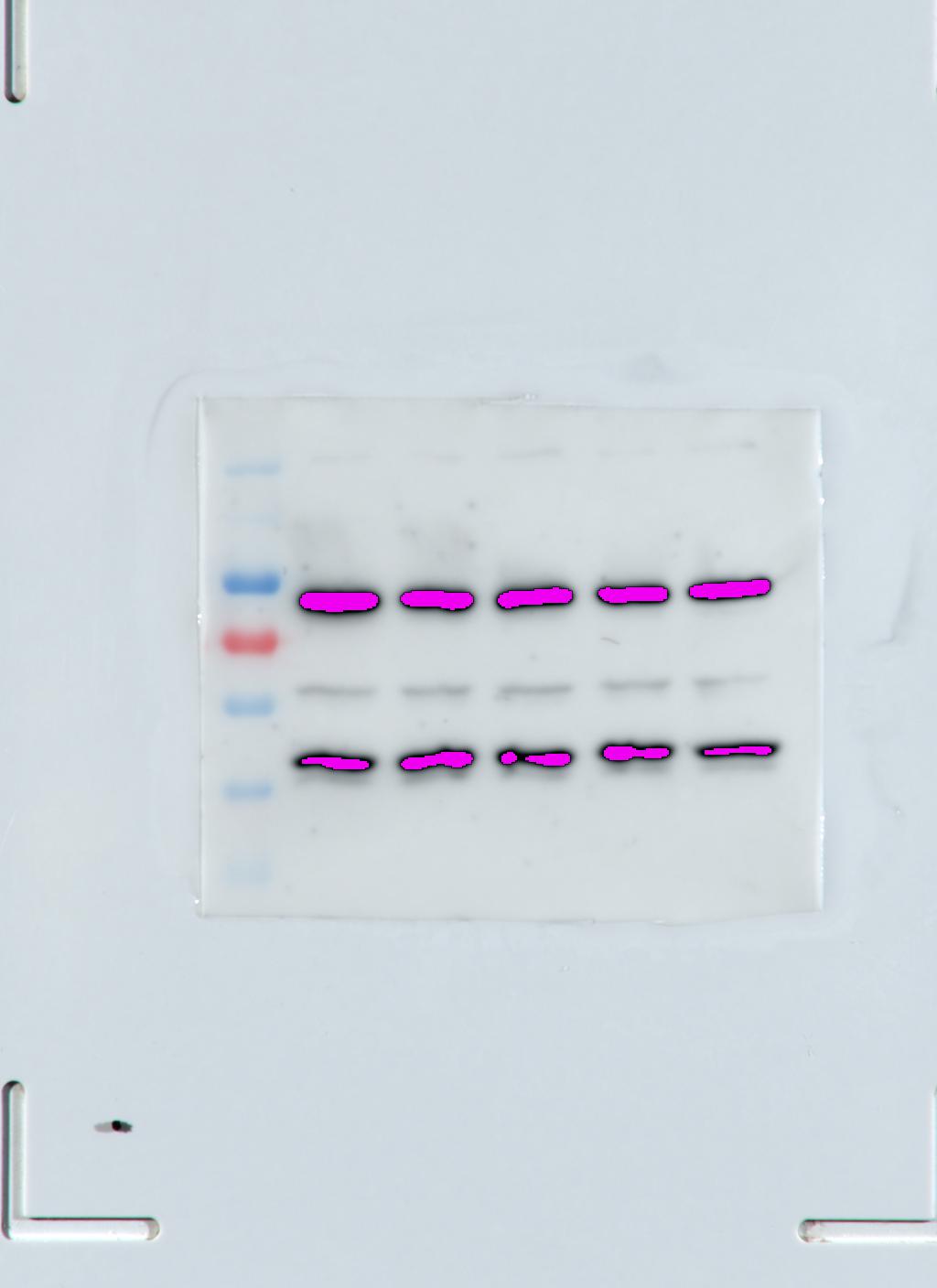

Supplement: Supplementary file 1 [file biomolecules-16-01059-s001.zip › File S1/Figure 6-8-11 Western blot original drawing/Figure 11a/PI3K/2/PI3K-2.jpg]

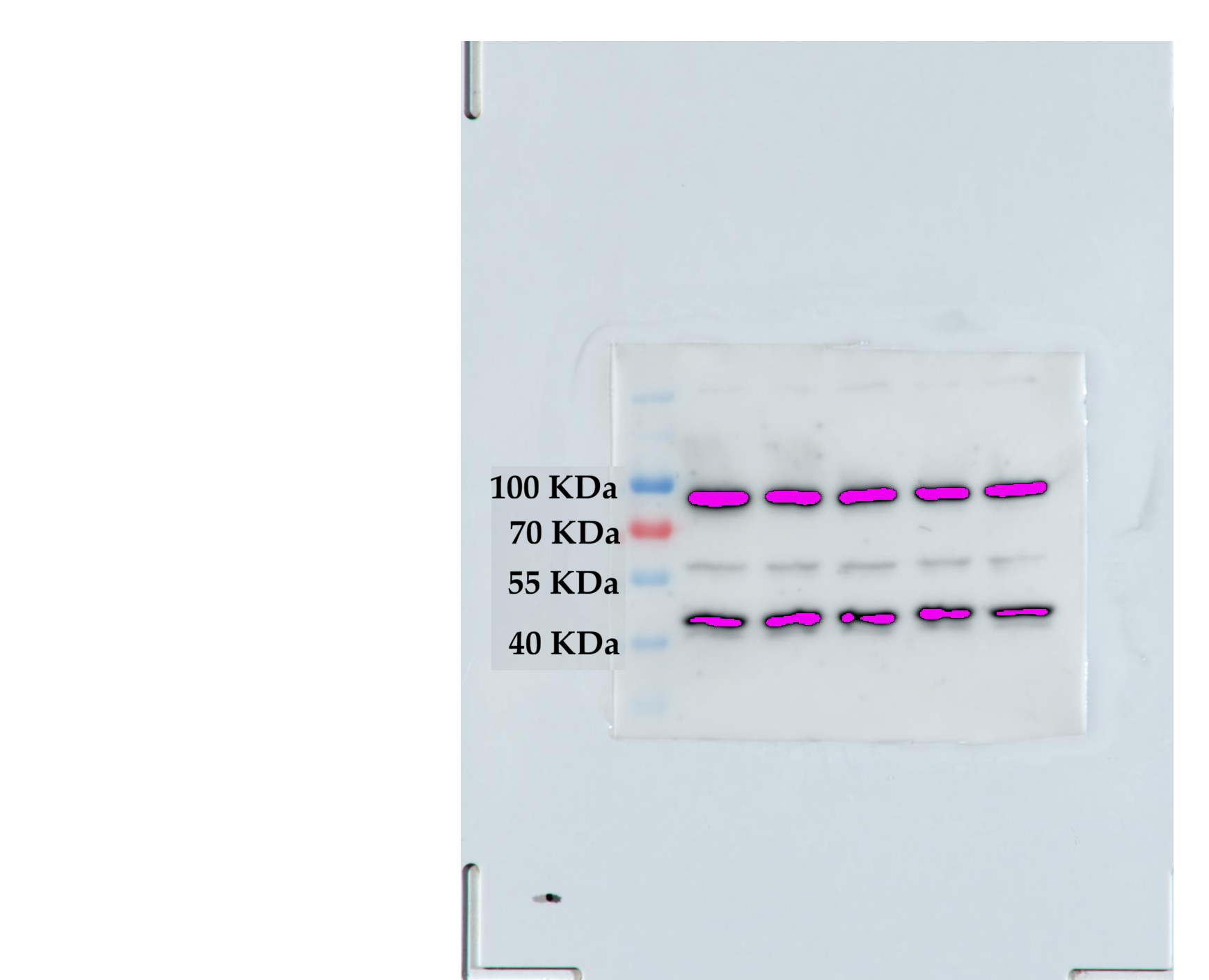

Supplement: Supplementary file 1 [file biomolecules-16-01059-s001.zip › File S1/Figure 6-8-11 Western blot original drawing/Figure 11a/PI3K/2/PI3K-2.png]

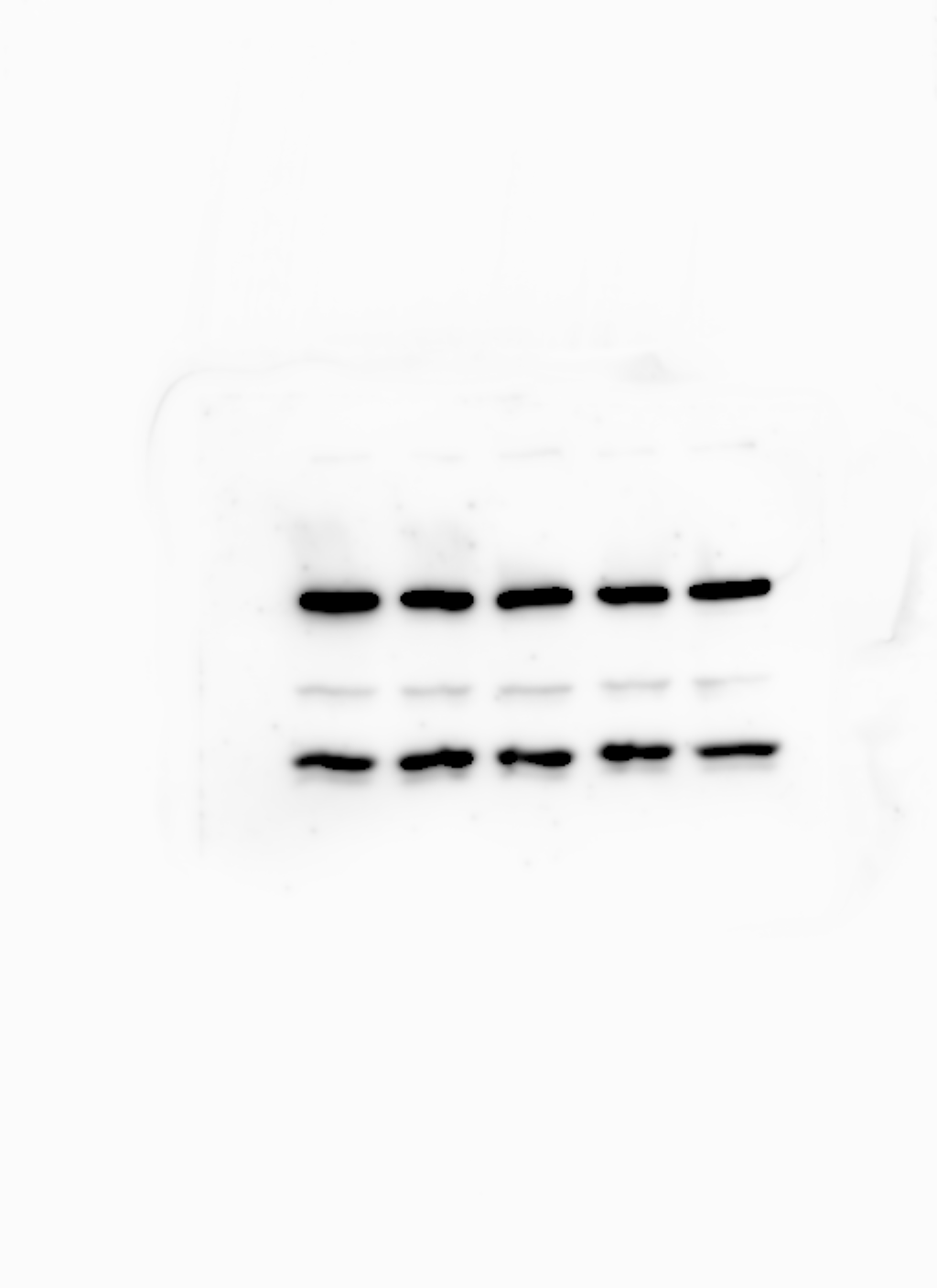

Supplement: Supplementary file 1 [file biomolecules-16-01059-s001.zip › File S1/Figure 6-8-11 Western blot original drawing/Figure 11a/PI3K/2/PI3K-2.tif]

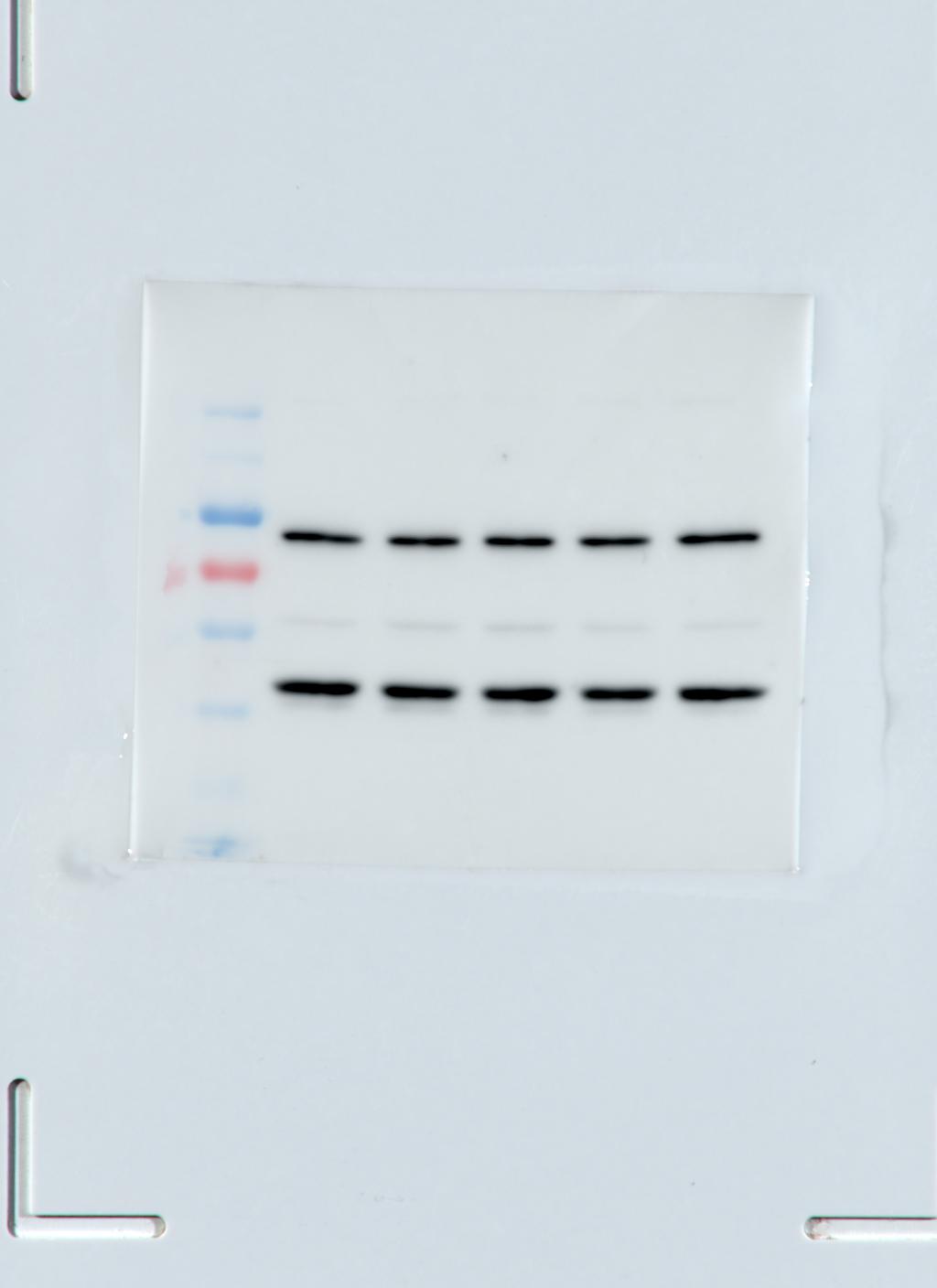

Supplement: Supplementary file 1 [file biomolecules-16-01059-s001.zip › File S1/Figure 6-8-11 Western blot original drawing/Figure 11a/PI3K/3/PI3K-3.jpg]

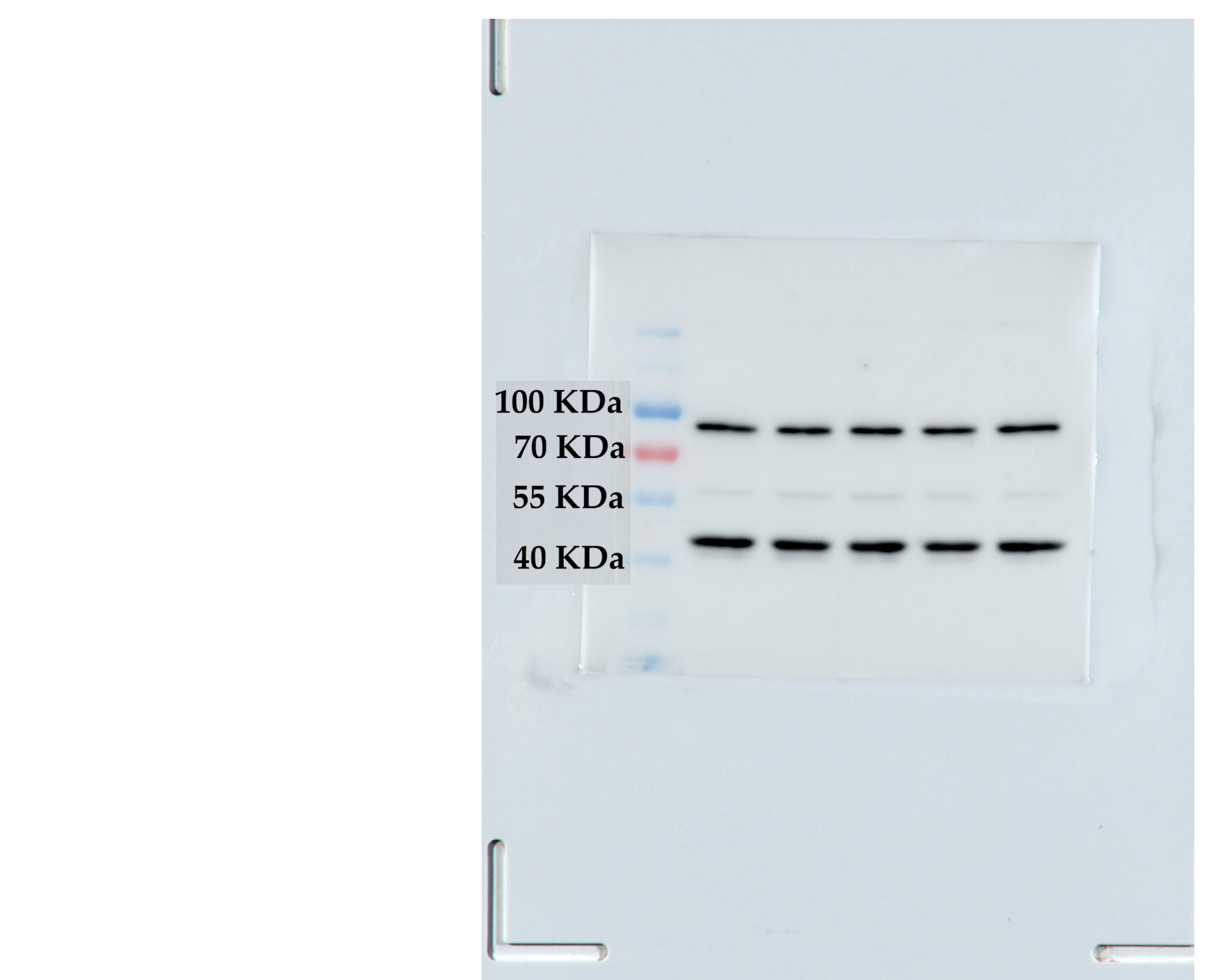

Supplement: Supplementary file 1 [file biomolecules-16-01059-s001.zip › File S1/Figure 6-8-11 Western blot original drawing/Figure 11a/PI3K/3/PI3K-3.png]

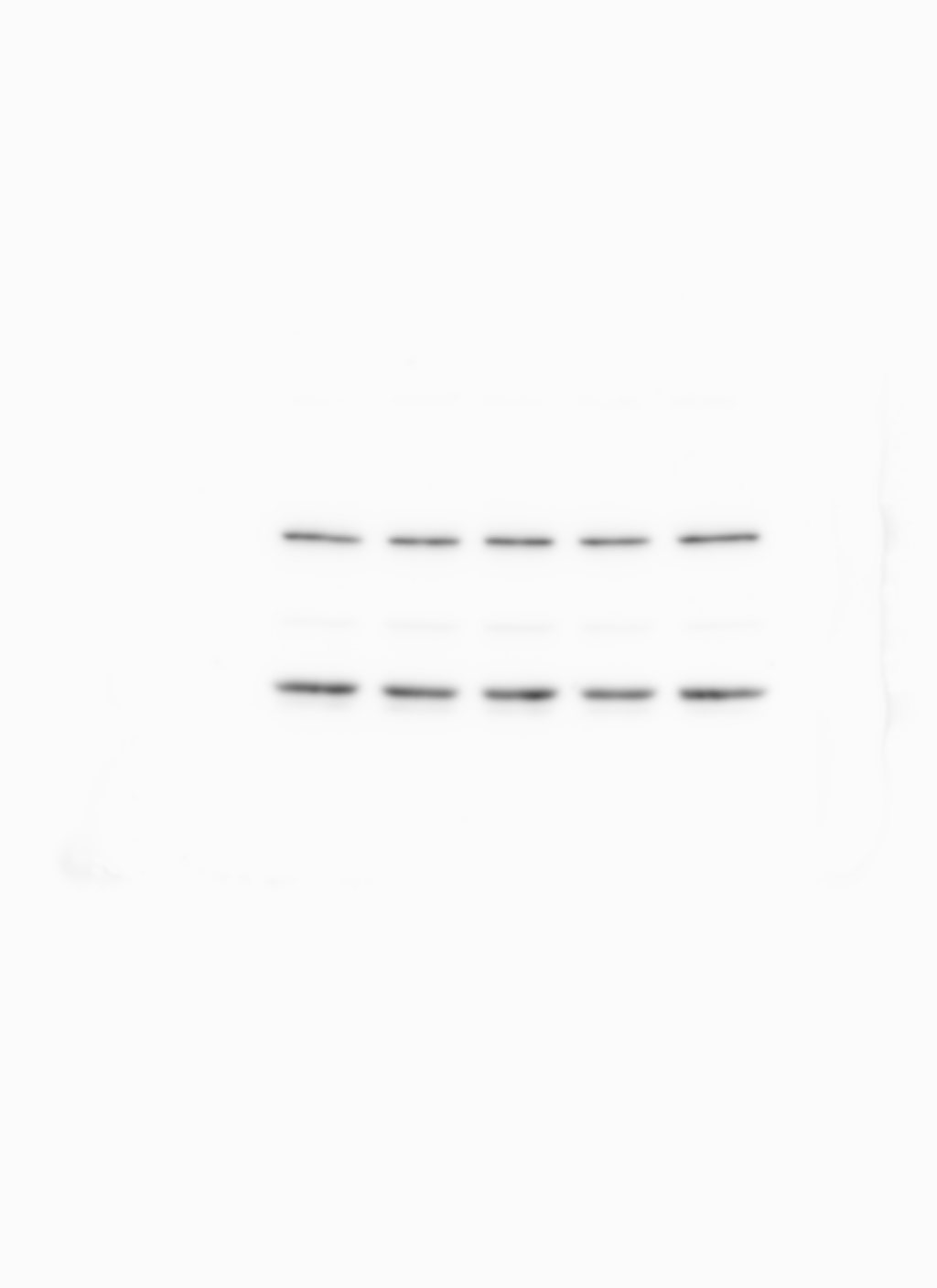

Supplement: Supplementary file 1 [file biomolecules-16-01059-s001.zip › File S1/Figure 6-8-11 Western blot original drawing/Figure 11a/PI3K/3/PI3K-3.tif]

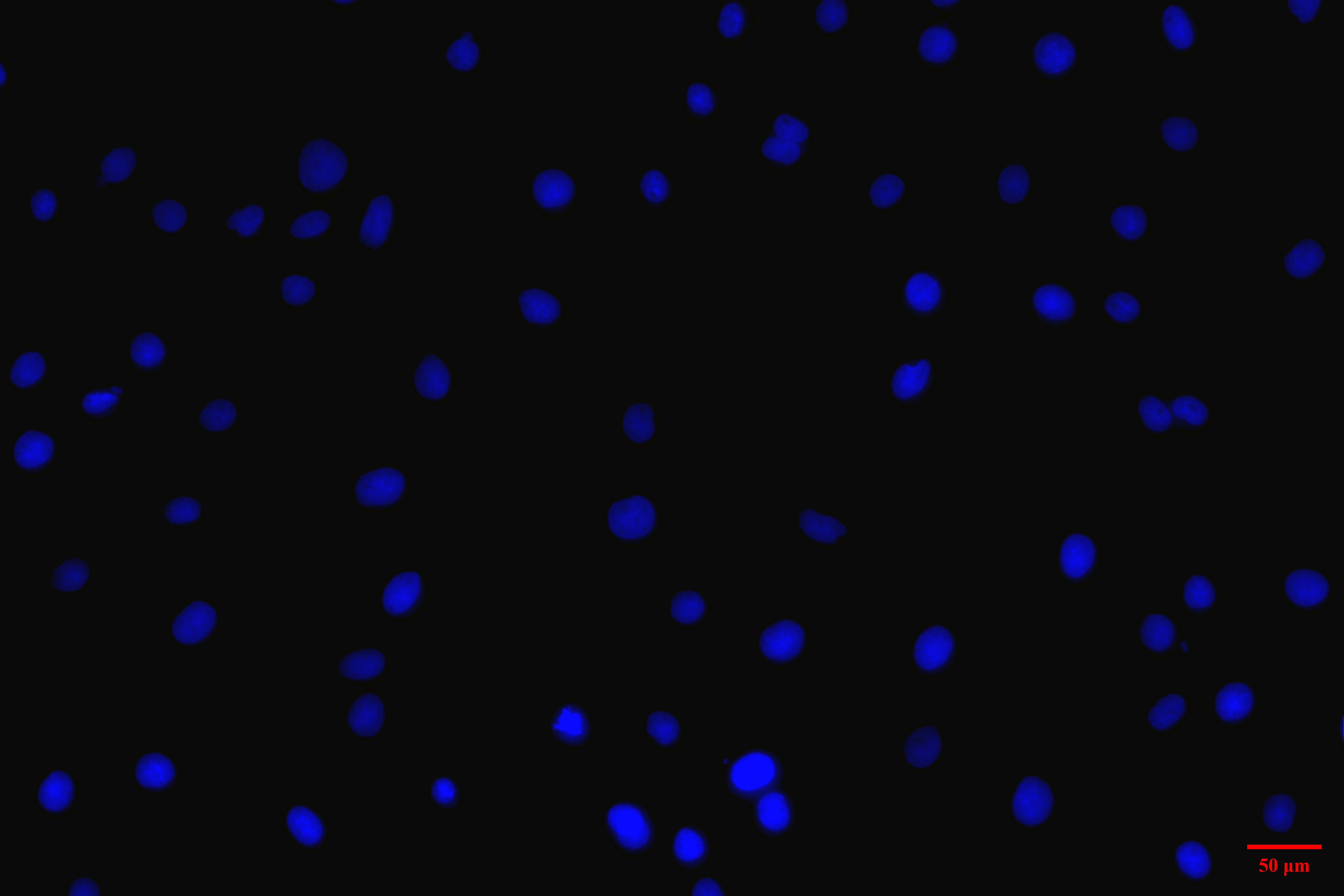

Supplement: Supplementary file 1 [file biomolecules-16-01059-s001.zip › File S1/Figure 6-8-11 Western blot original drawing/Figure 11e/CoCl2/DAPI-M-1.jpg]

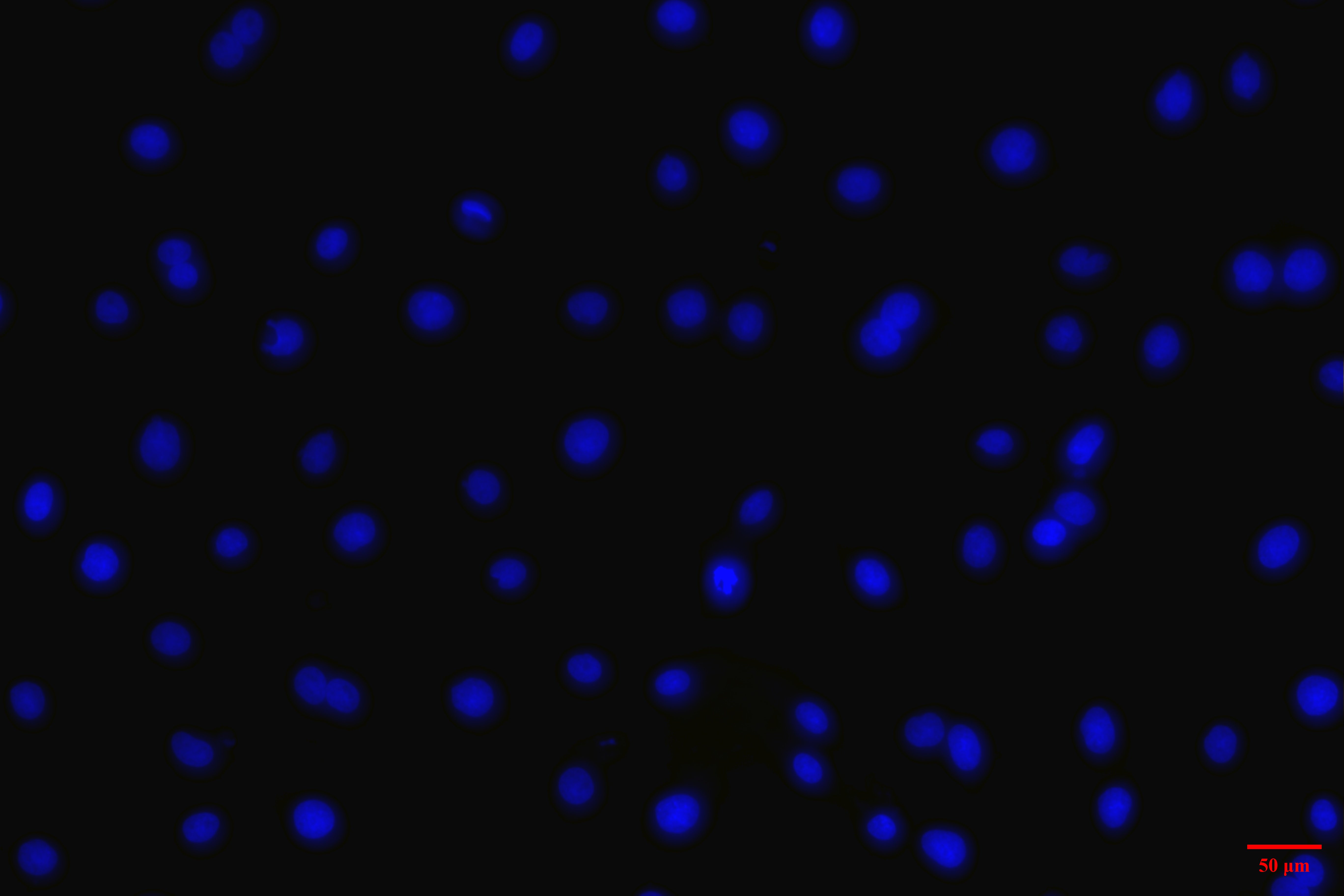

Supplement: Supplementary file 1 [file biomolecules-16-01059-s001.zip › File S1/Figure 6-8-11 Western blot original drawing/Figure 11e/CoCl2/DAPI-M-2.jpg]

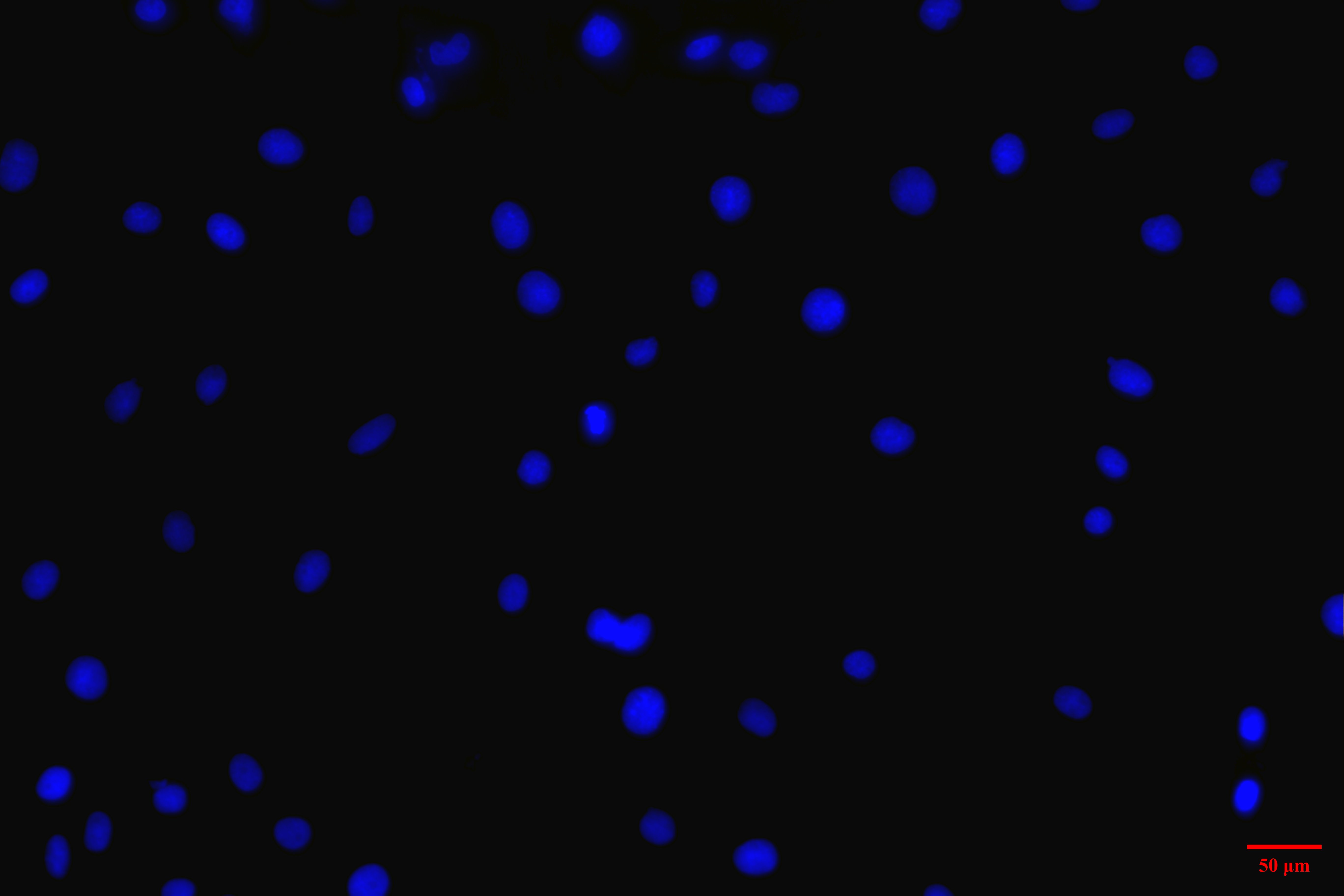

Supplement: Supplementary file 1 [file biomolecules-16-01059-s001.zip › File S1/Figure 6-8-11 Western blot original drawing/Figure 11e/CoCl2/DAPI-M-3.jpg]

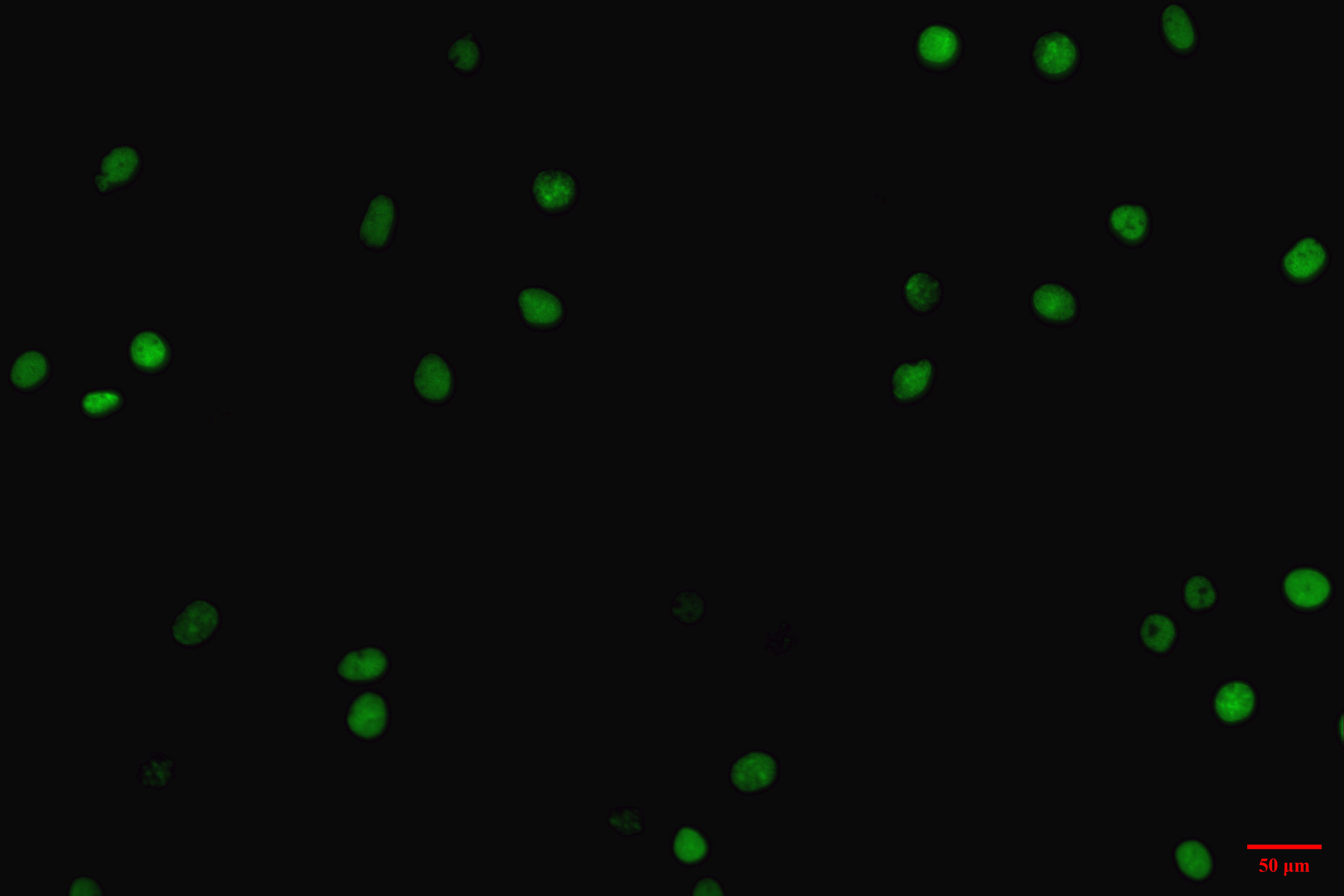

Supplement: Supplementary file 1 [file biomolecules-16-01059-s001.zip › File S1/Figure 6-8-11 Western blot original drawing/Figure 11e/CoCl2/EDU-M-1.jpg]

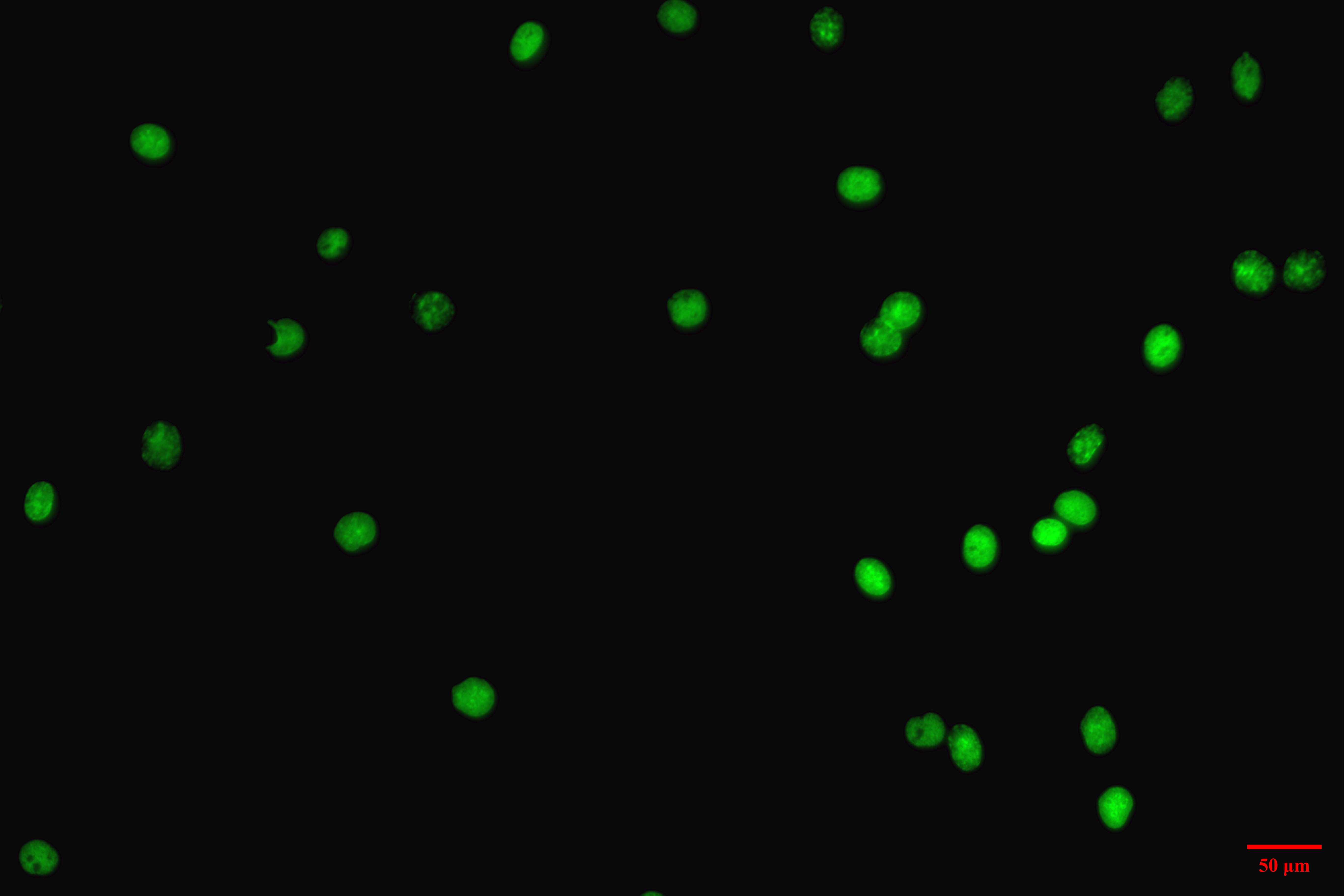

Supplement: Supplementary file 1 [file biomolecules-16-01059-s001.zip › File S1/Figure 6-8-11 Western blot original drawing/Figure 11e/CoCl2/EDU-M-2.jpg]

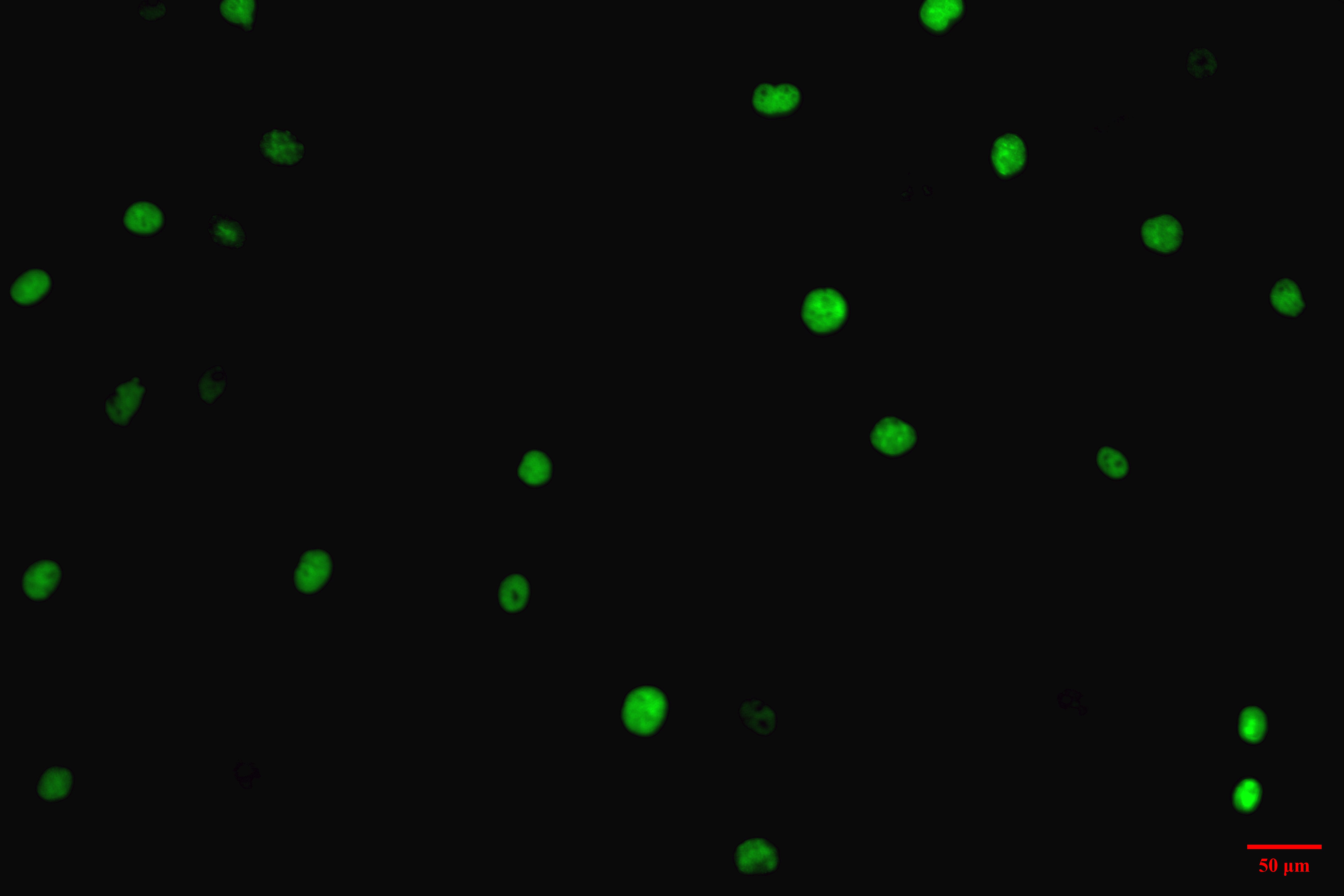

Supplement: Supplementary file 1 [file biomolecules-16-01059-s001.zip › File S1/Figure 6-8-11 Western blot original drawing/Figure 11e/CoCl2/EDU-M-3.jpg]

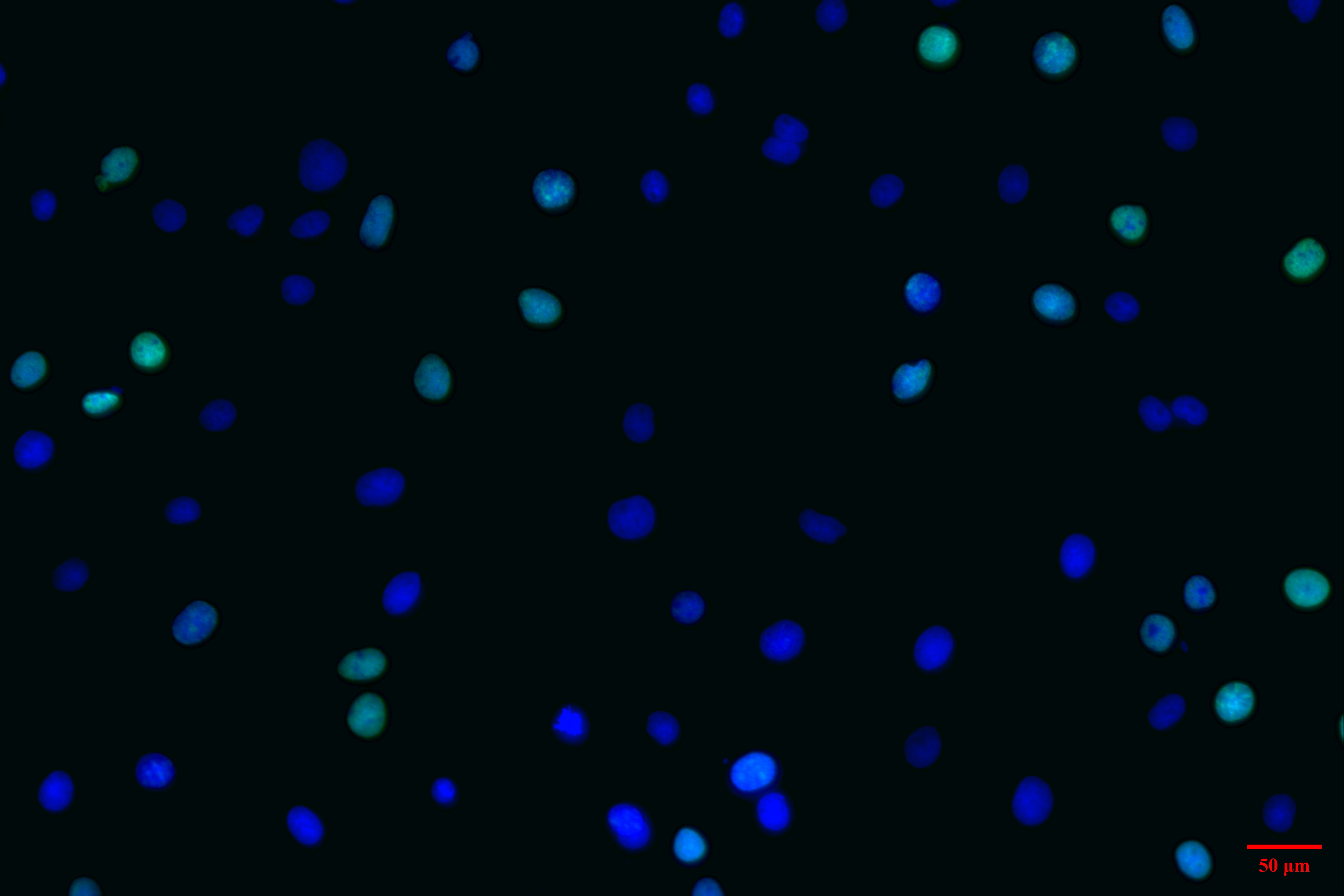

Supplement: Supplementary file 1 [file biomolecules-16-01059-s001.zip › File S1/Figure 6-8-11 Western blot original drawing/Figure 11e/CoCl2/Merge-M-1.jpg]

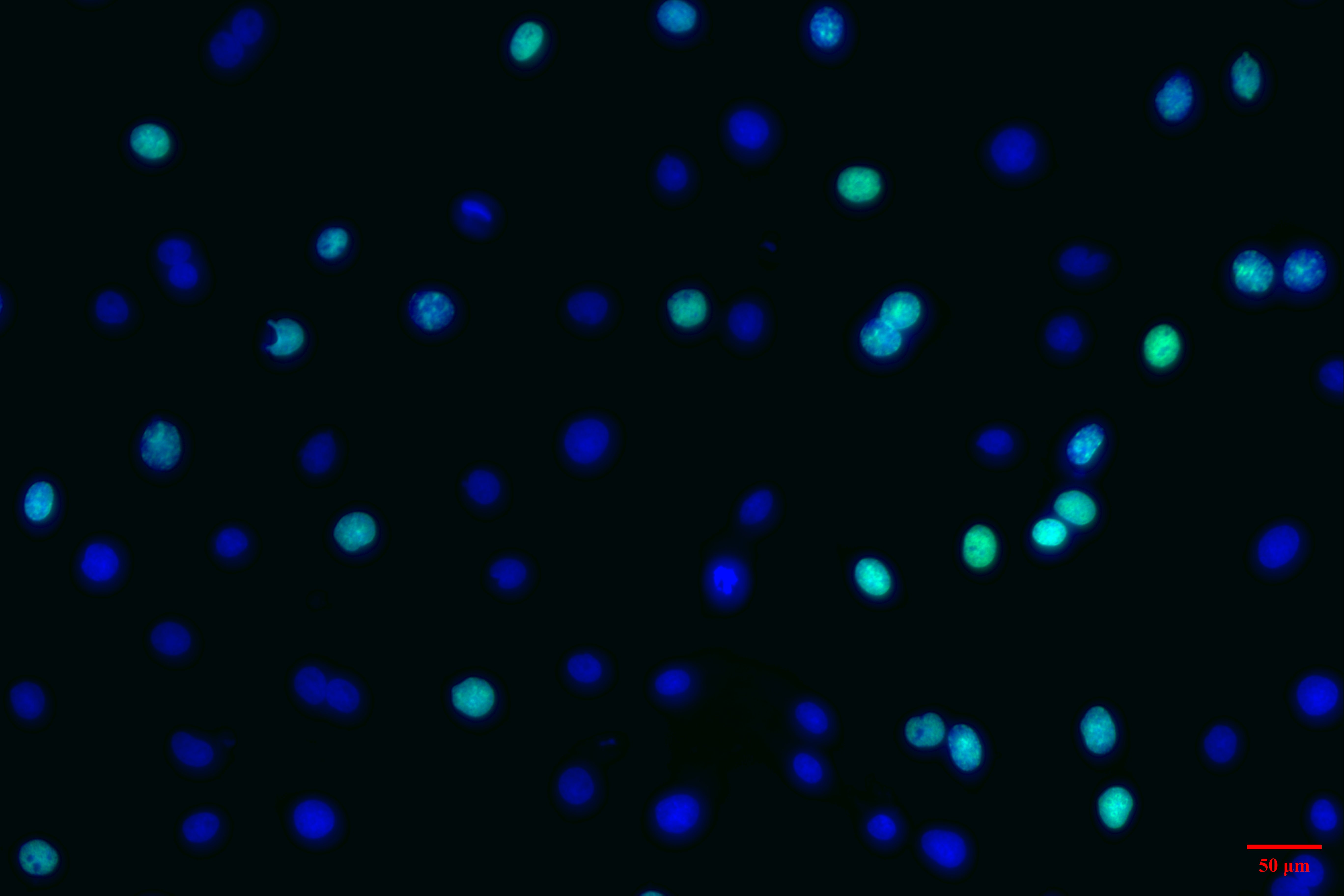

Supplement: Supplementary file 1 [file biomolecules-16-01059-s001.zip › File S1/Figure 6-8-11 Western blot original drawing/Figure 11e/CoCl2/Merge-M-2.jpg]

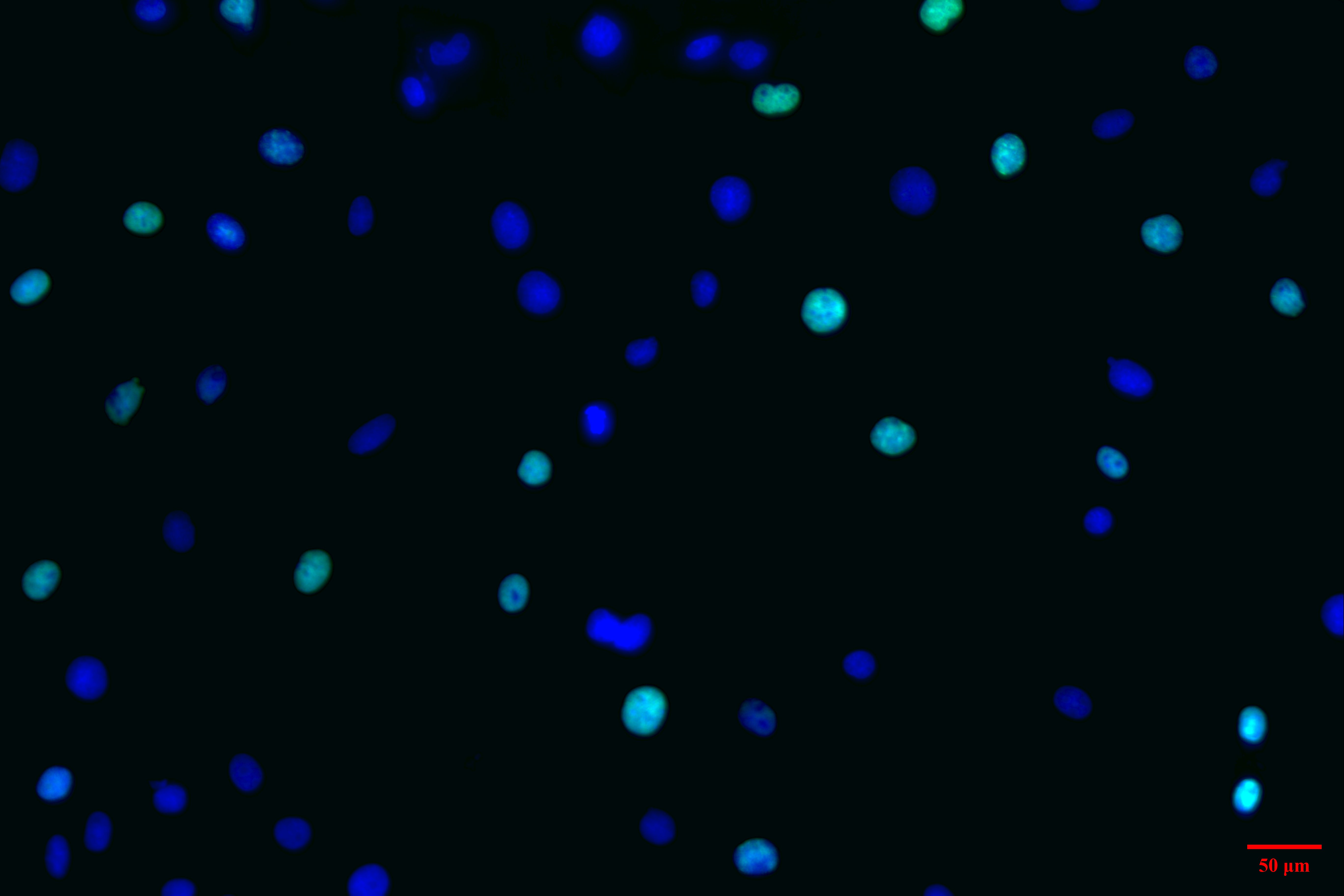

Supplement: Supplementary file 1 [file biomolecules-16-01059-s001.zip › File S1/Figure 6-8-11 Western blot original drawing/Figure 11e/CoCl2/Merge-M-3.jpg]

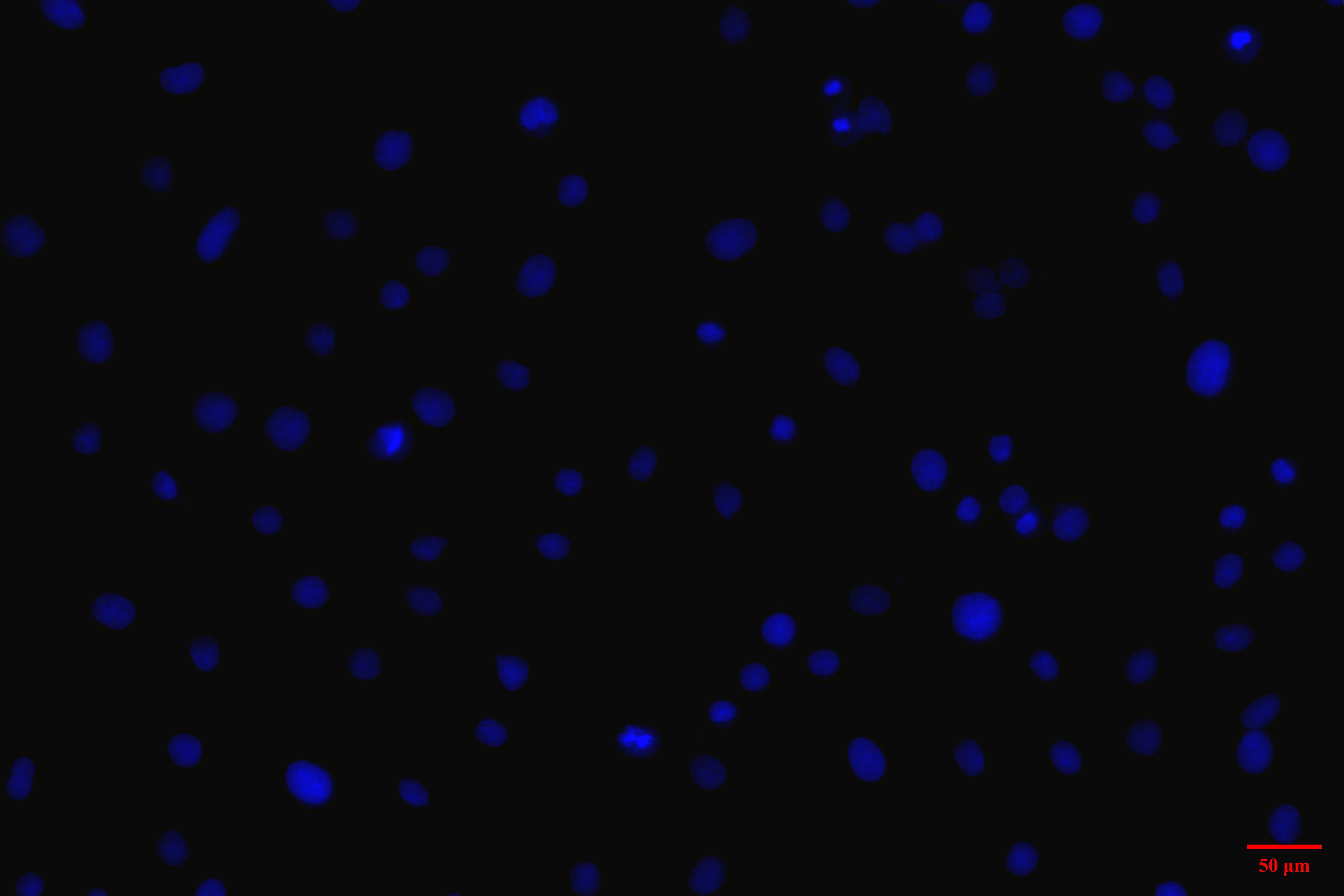

Supplement: Supplementary file 1 [file biomolecules-16-01059-s001.zip › File S1/Figure 6-8-11 Western blot original drawing/Figure 11e/CoCl2+BEL(20μmolL )/DAPI-20-1.jpg]

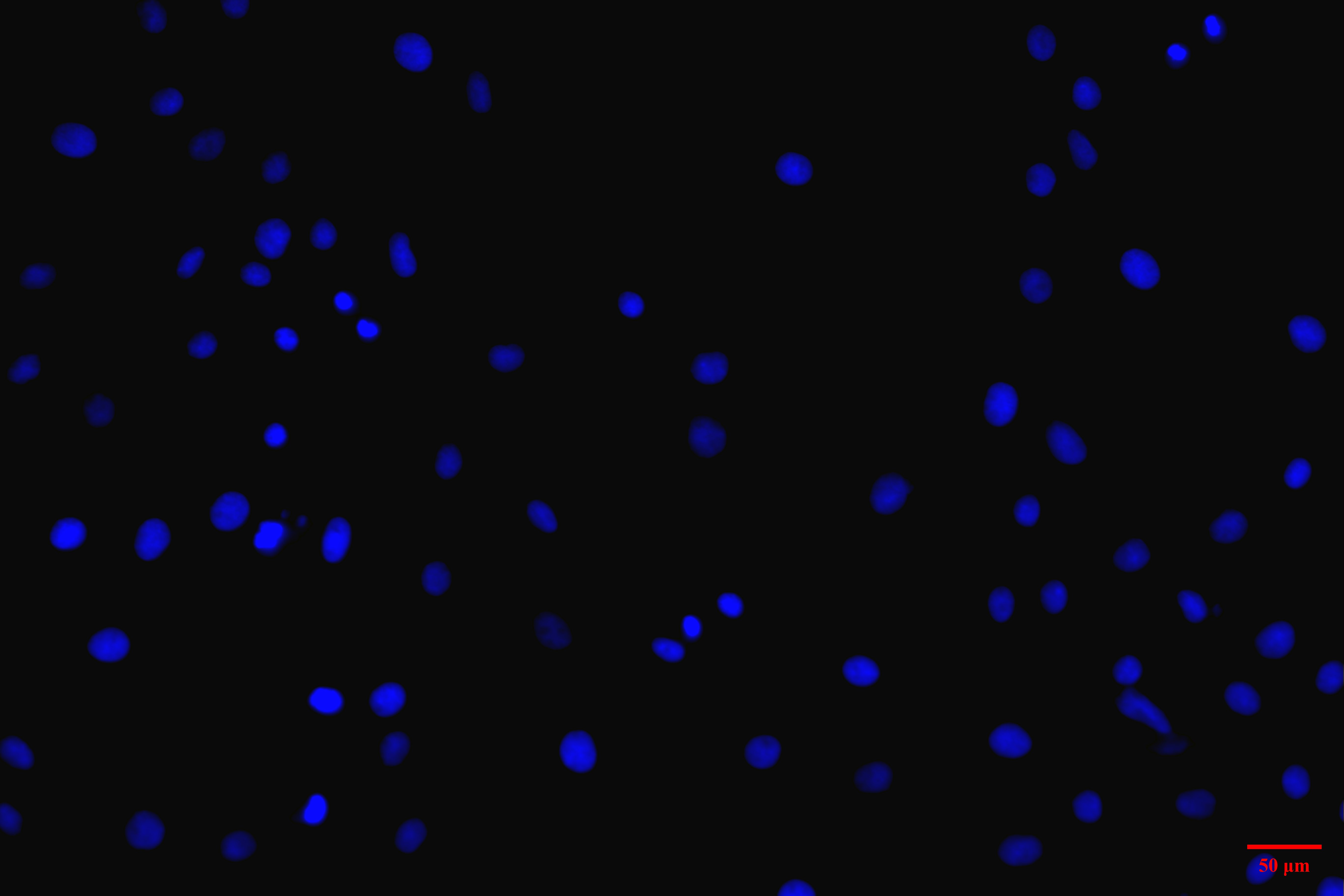

Supplement: Supplementary file 1 [file biomolecules-16-01059-s001.zip › File S1/Figure 6-8-11 Western blot original drawing/Figure 11e/CoCl2+BEL(20μmolL )/DAPI-20-2.jpg]

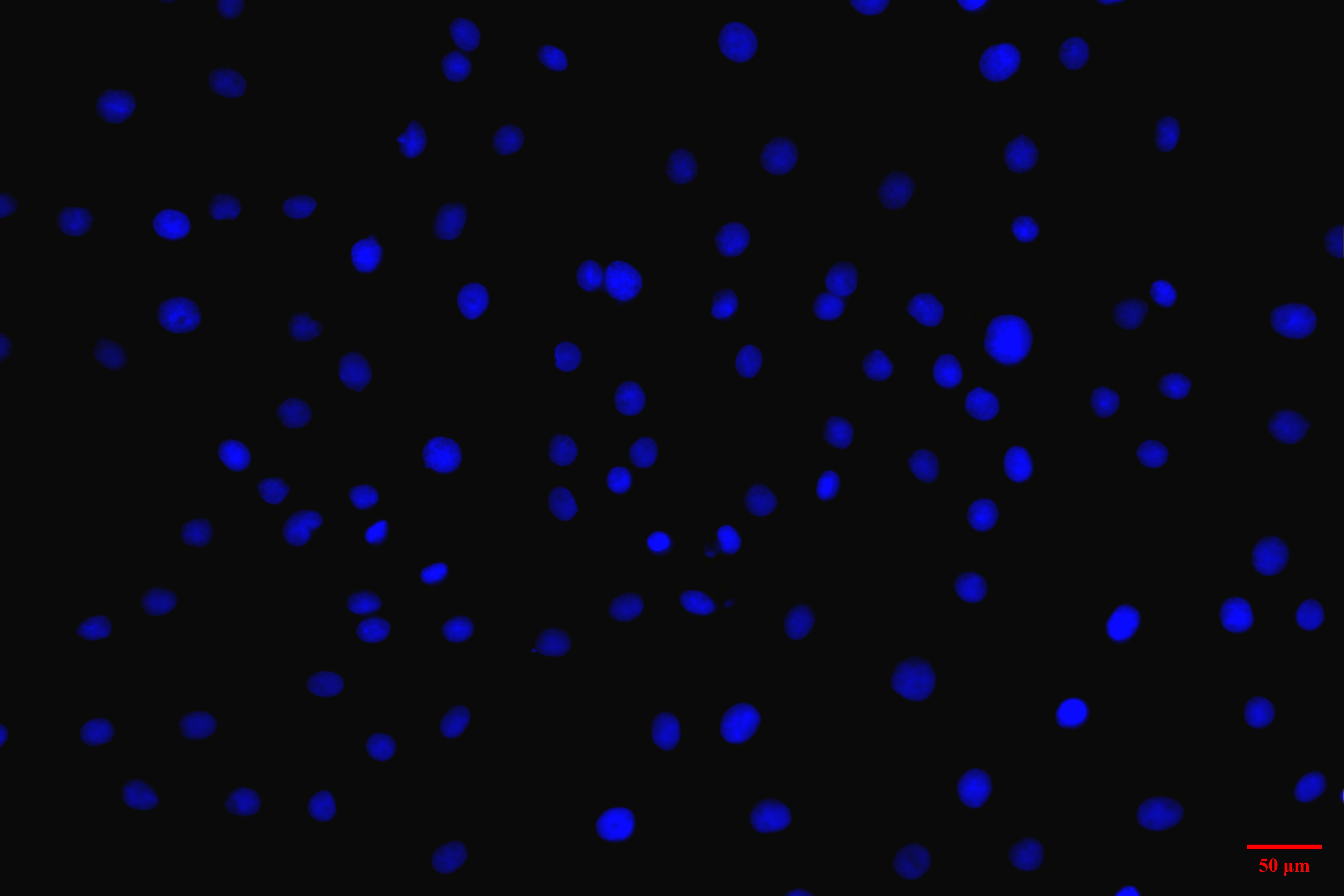

Supplement: Supplementary file 1 [file biomolecules-16-01059-s001.zip › File S1/Figure 6-8-11 Western blot original drawing/Figure 11e/CoCl2+BEL(20μmolL )/DAPI-20-3.jpg]

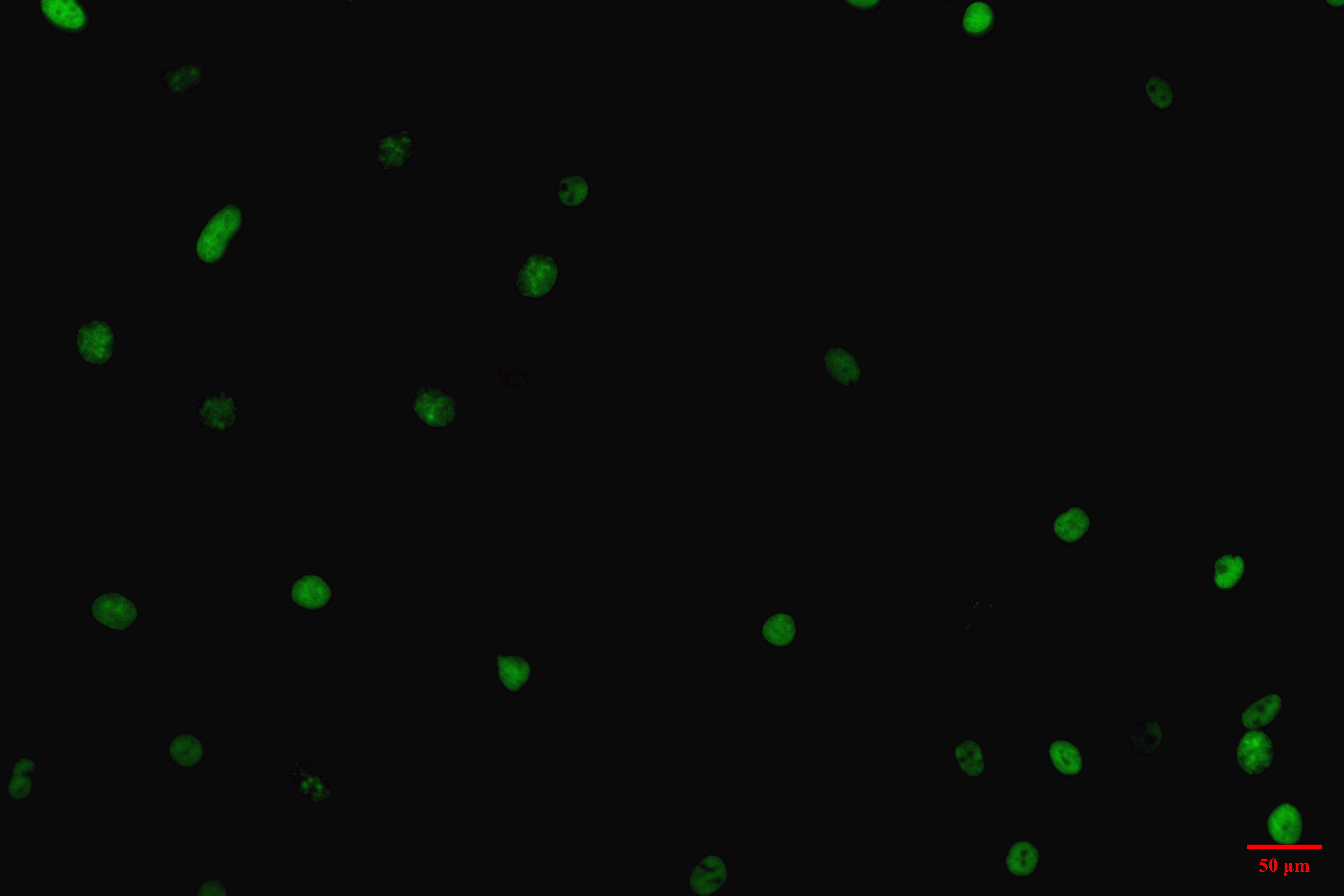

Supplement: Supplementary file 1 [file biomolecules-16-01059-s001.zip › File S1/Figure 6-8-11 Western blot original drawing/Figure 11e/CoCl2+BEL(20μmolL )/EDU-20-1.jpg]

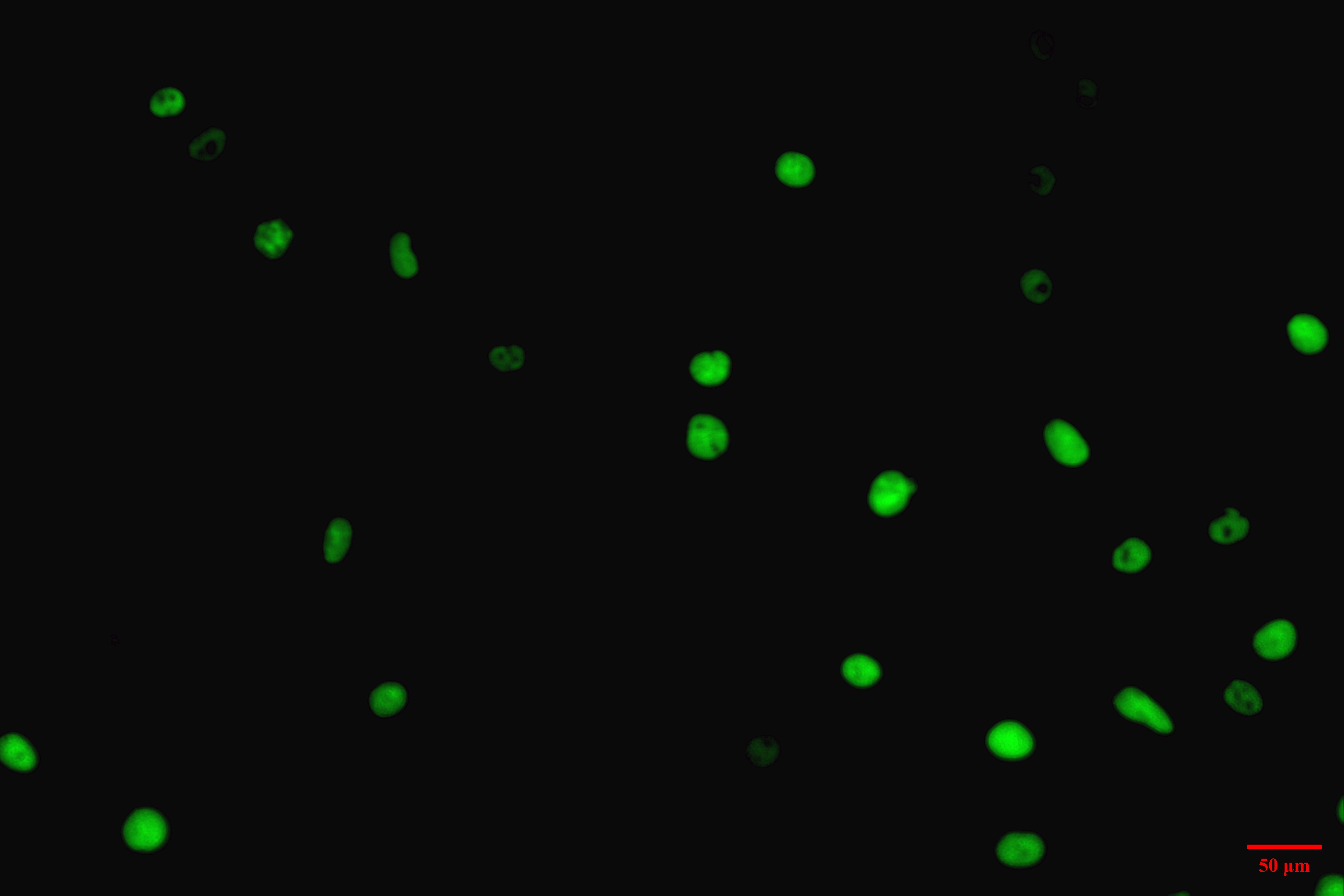

Supplement: Supplementary file 1 [file biomolecules-16-01059-s001.zip › File S1/Figure 6-8-11 Western blot original drawing/Figure 11e/CoCl2+BEL(20μmolL )/EDU-20-2.jpg]

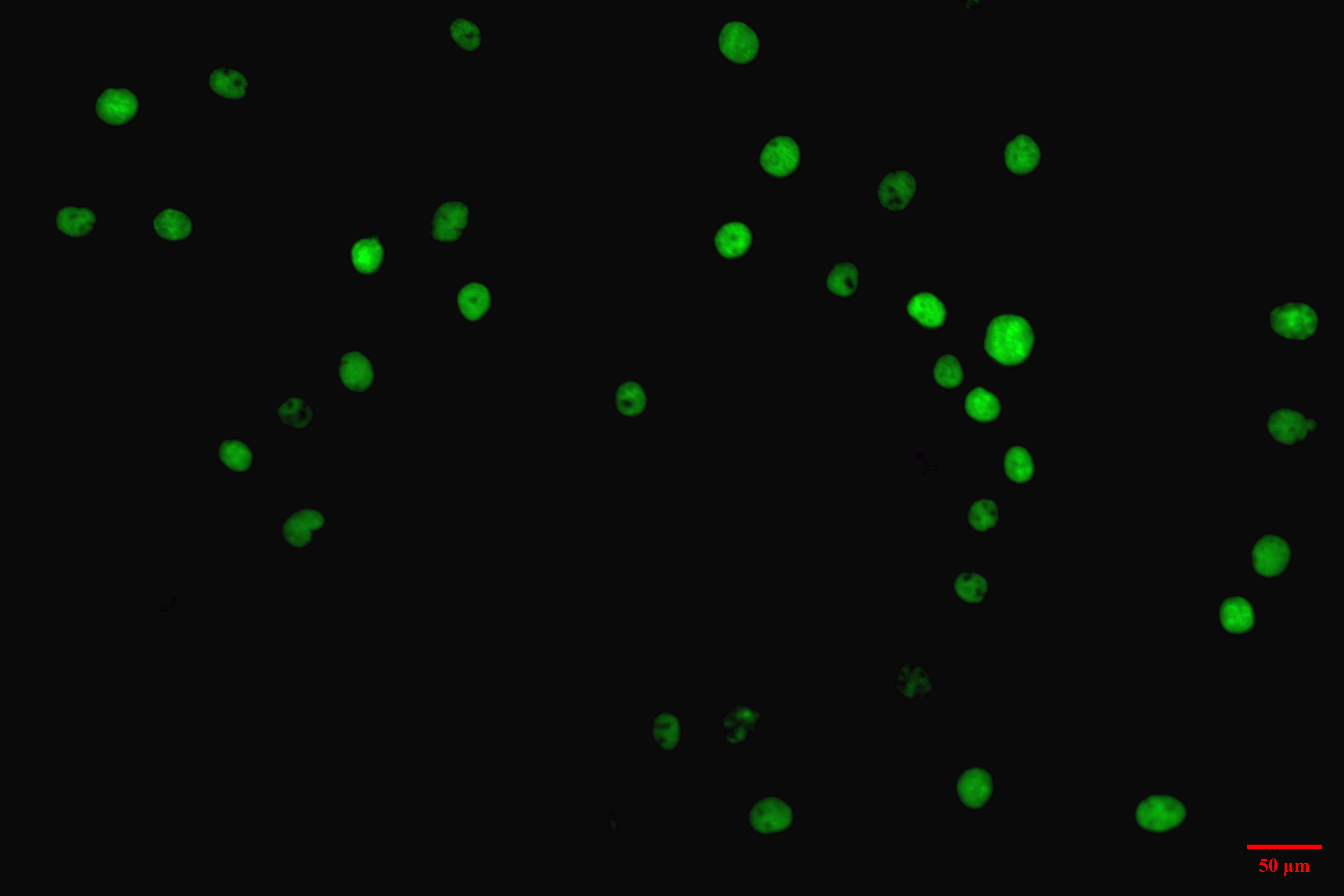

Supplement: Supplementary file 1 [file biomolecules-16-01059-s001.zip › File S1/Figure 6-8-11 Western blot original drawing/Figure 11e/CoCl2+BEL(20μmolL )/EDU-20-3.jpg]

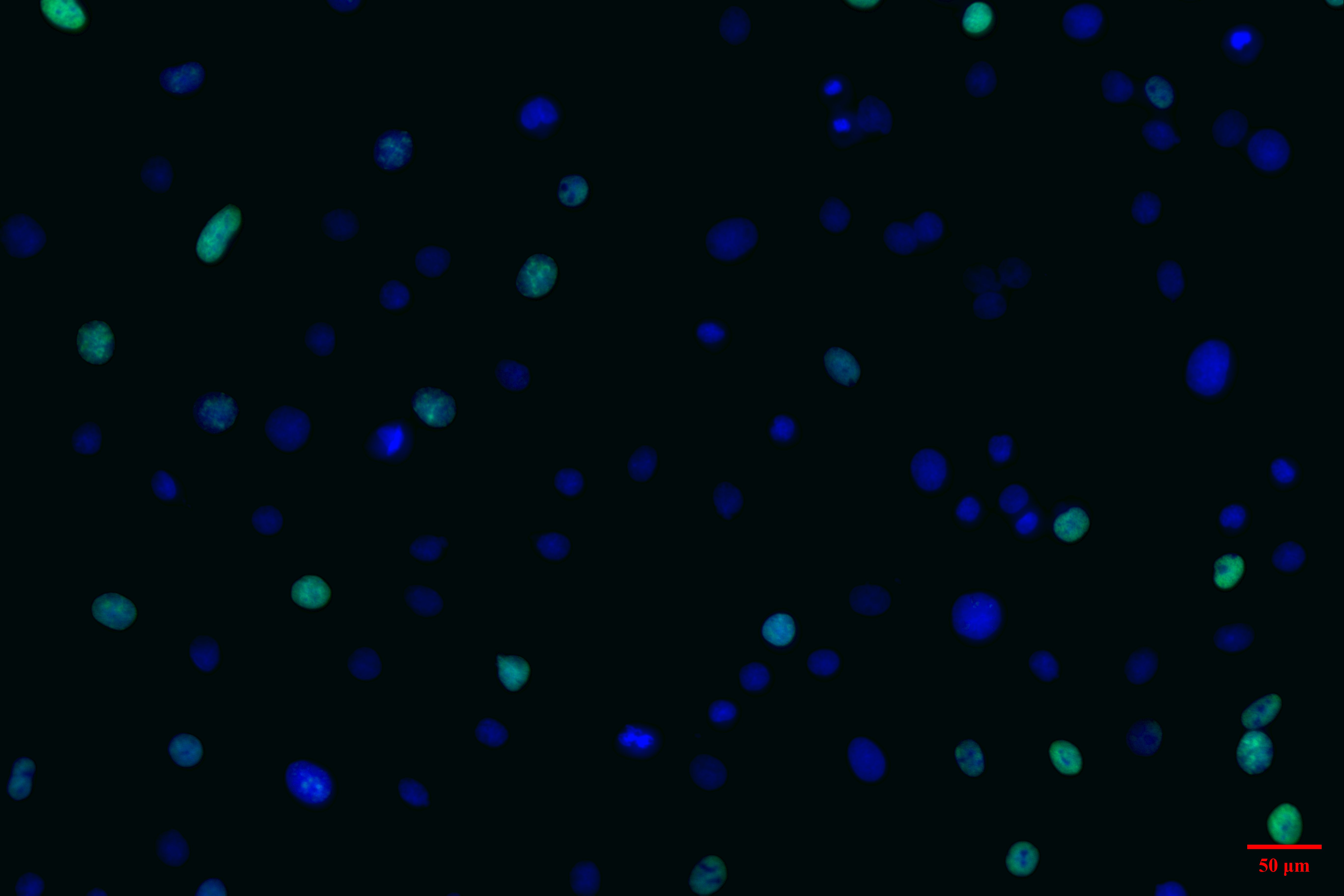

Supplement: Supplementary file 1 [file biomolecules-16-01059-s001.zip › File S1/Figure 6-8-11 Western blot original drawing/Figure 11e/CoCl2+BEL(20μmolL )/Merge-20-1.jpg]

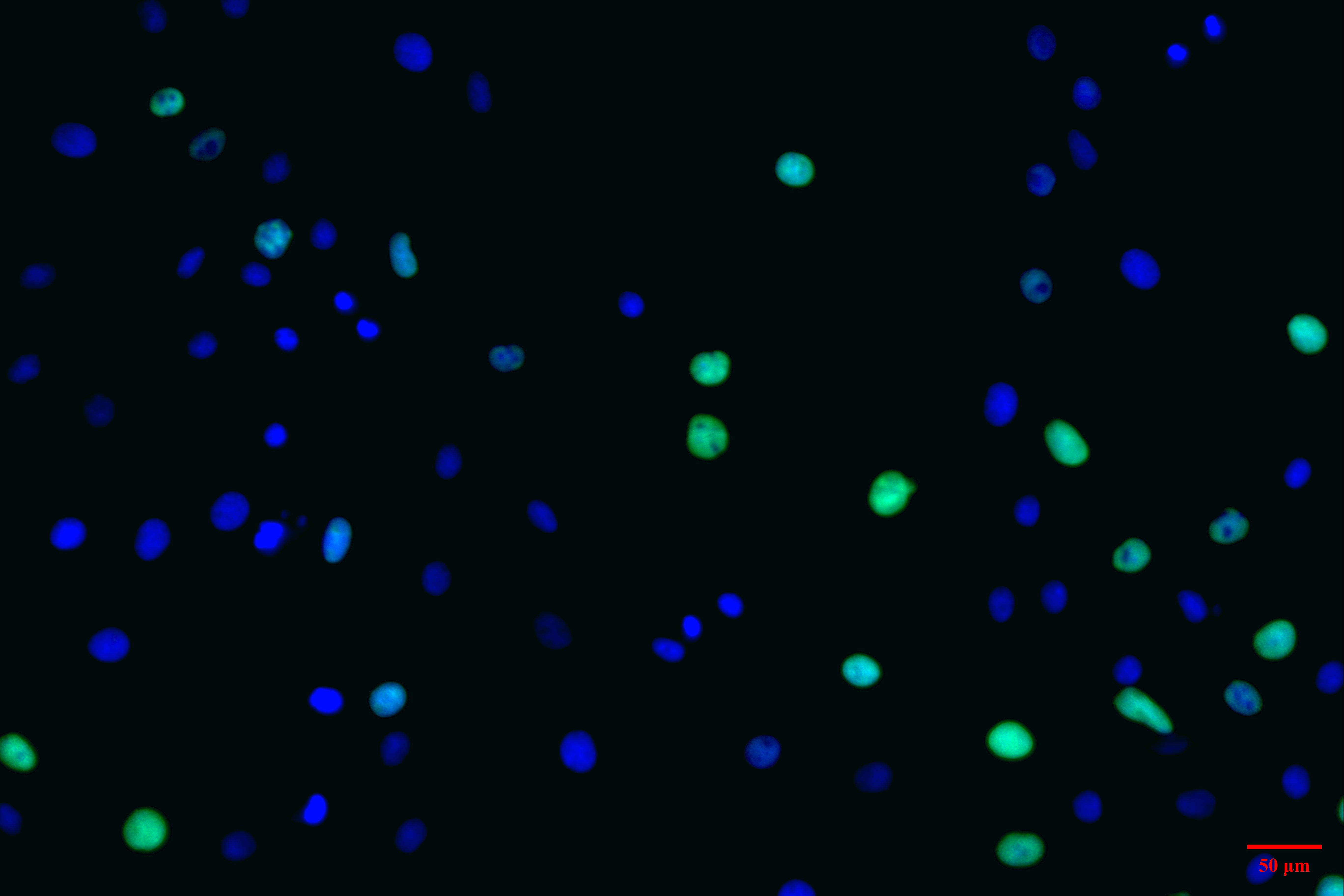

Supplement: Supplementary file 1 [file biomolecules-16-01059-s001.zip › File S1/Figure 6-8-11 Western blot original drawing/Figure 11e/CoCl2+BEL(20μmolL )/Merge-20-2.jpg]

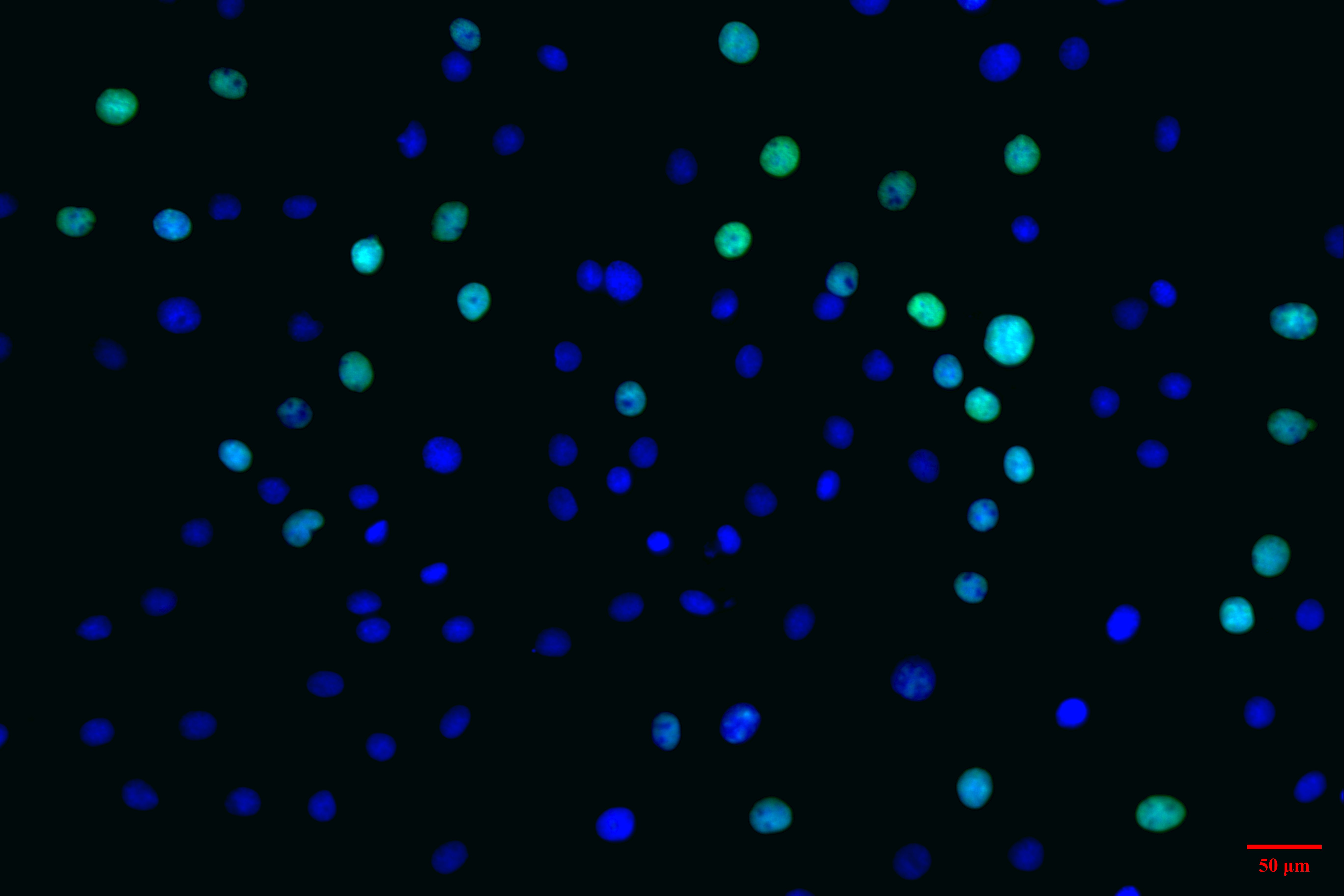

Supplement: Supplementary file 1 [file biomolecules-16-01059-s001.zip › File S1/Figure 6-8-11 Western blot original drawing/Figure 11e/CoCl2+BEL(20μmolL )/Merge-20-3.jpg]

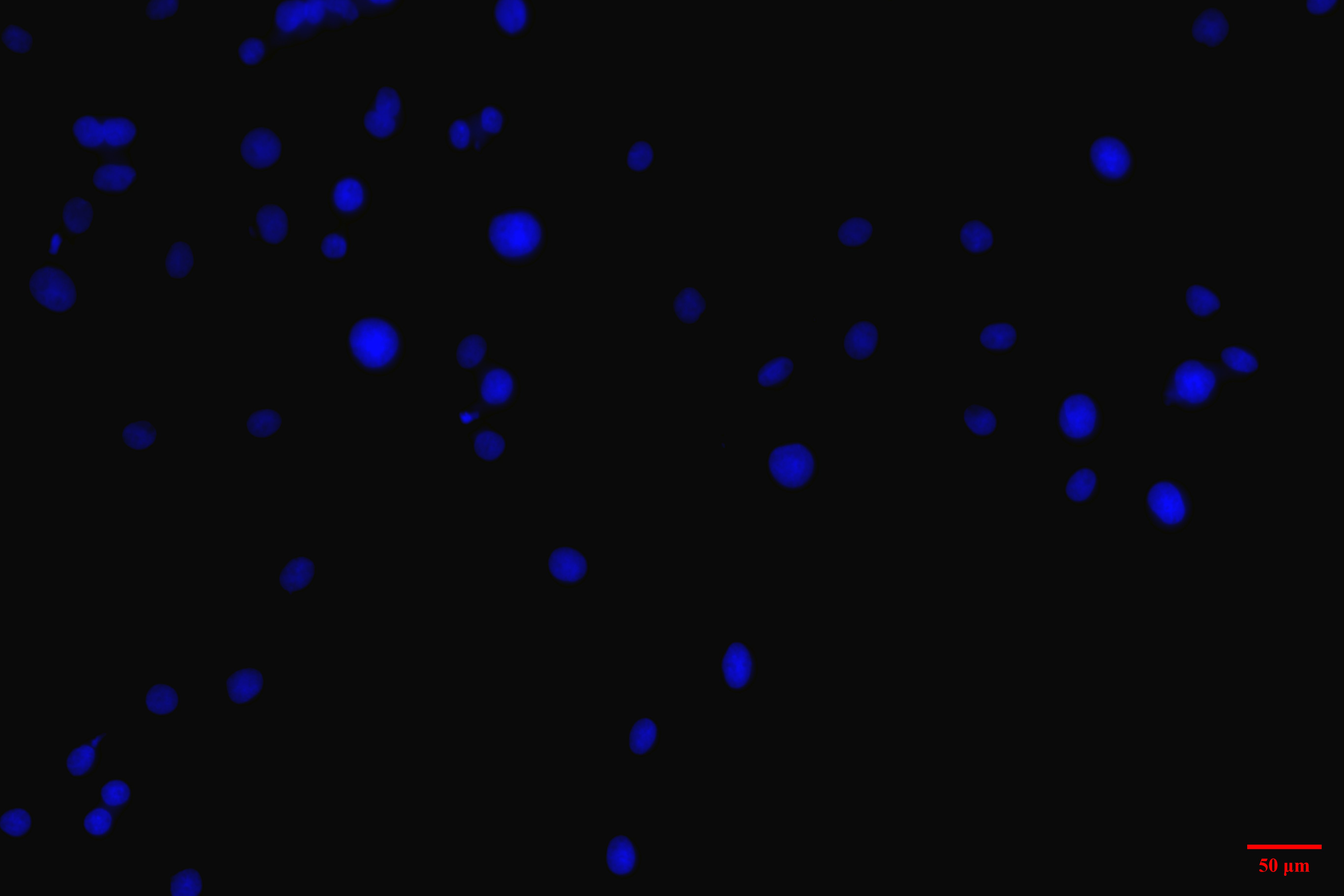

Supplement: Supplementary file 1 [file biomolecules-16-01059-s001.zip › File S1/Figure 6-8-11 Western blot original drawing/Figure 11e/CoCl2+BEL(50μmolL )/DAPI-50-1.jpg]

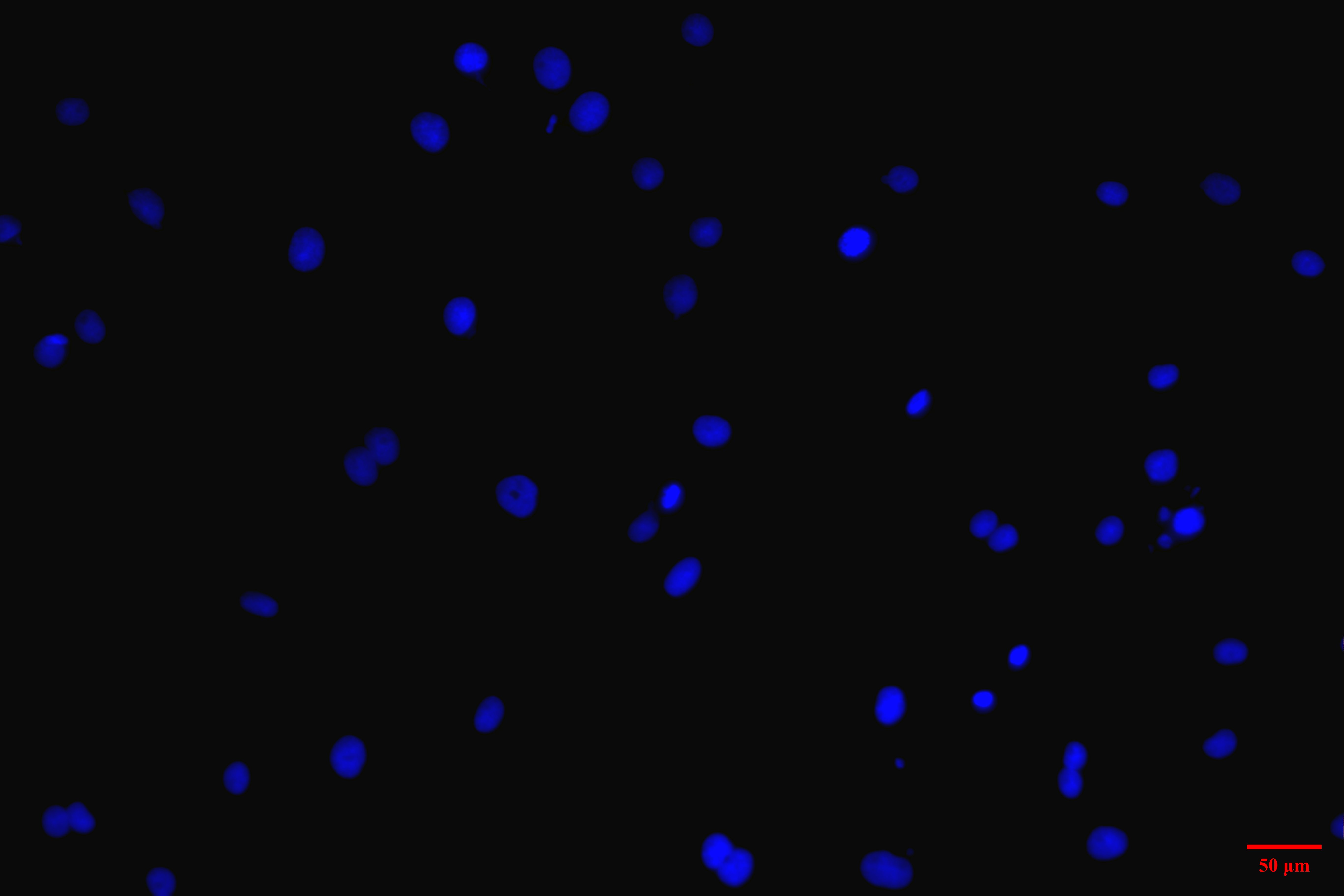

Supplement: Supplementary file 1 [file biomolecules-16-01059-s001.zip › File S1/Figure 6-8-11 Western blot original drawing/Figure 11e/CoCl2+BEL(50μmolL )/DAPI-50-2.jpg]

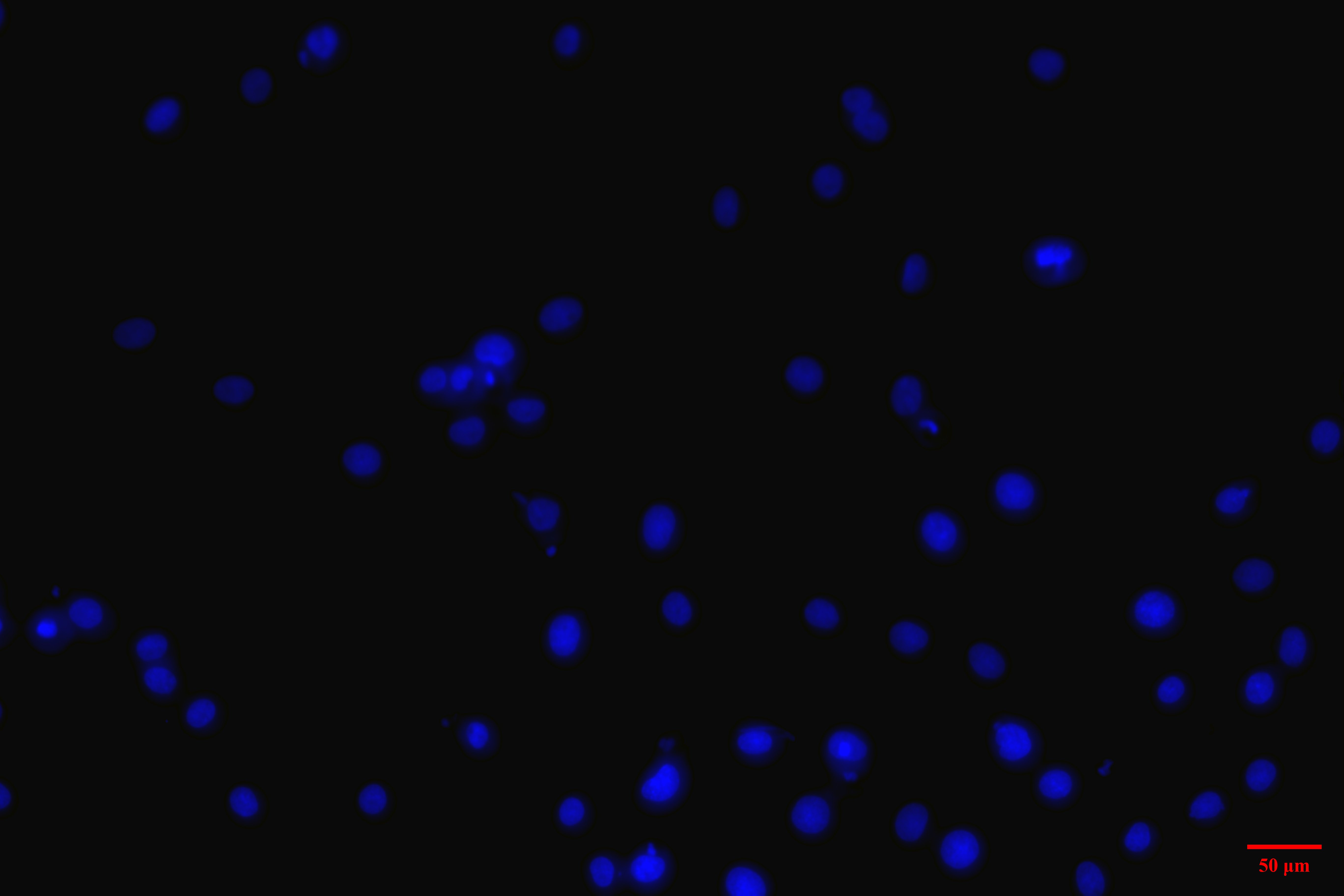

Supplement: Supplementary file 1 [file biomolecules-16-01059-s001.zip › File S1/Figure 6-8-11 Western blot original drawing/Figure 11e/CoCl2+BEL(50μmolL )/DAPI-50-3.jpg]

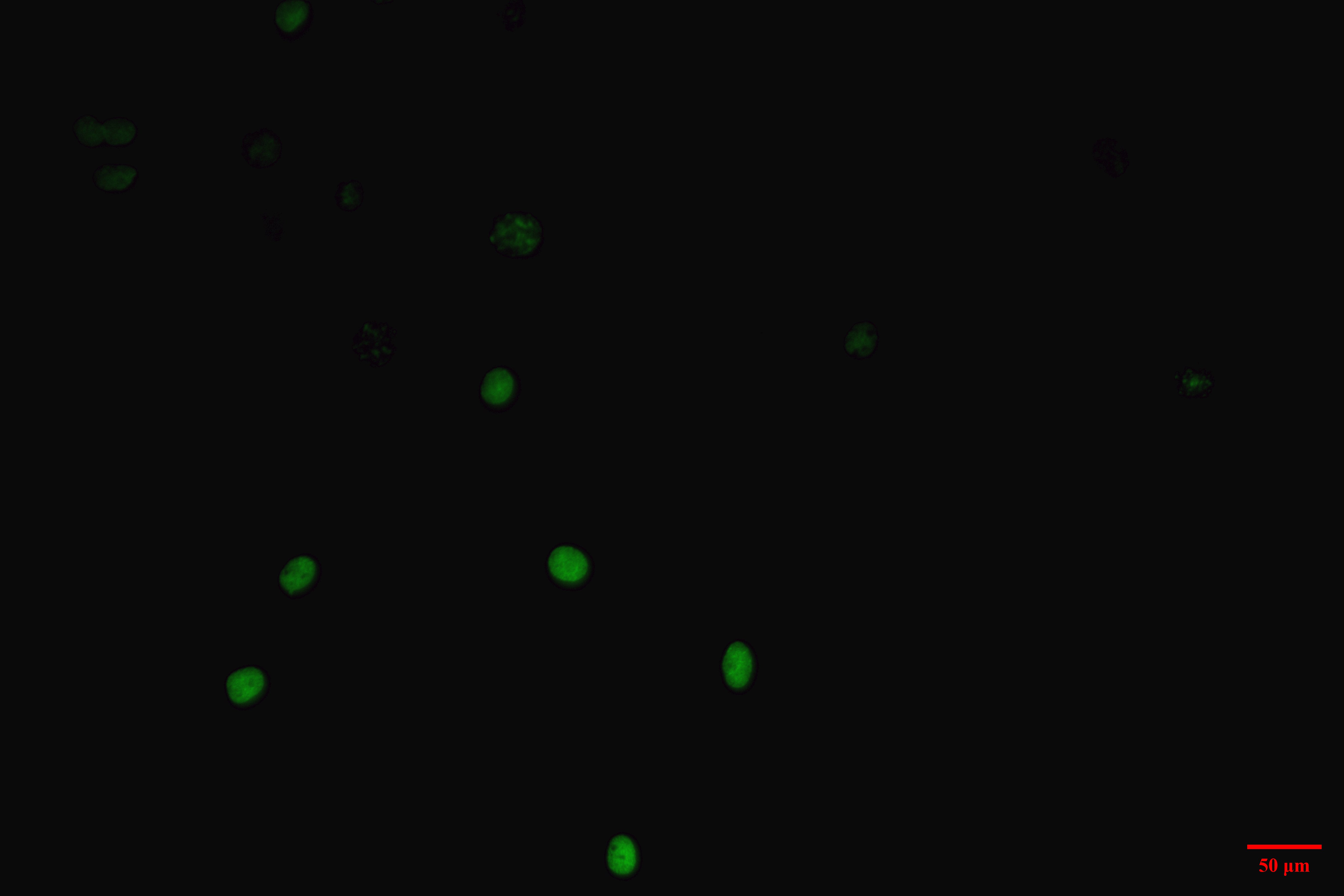

Supplement: Supplementary file 1 [file biomolecules-16-01059-s001.zip › File S1/Figure 6-8-11 Western blot original drawing/Figure 11e/CoCl2+BEL(50μmolL )/EDU-50-1.jpg]

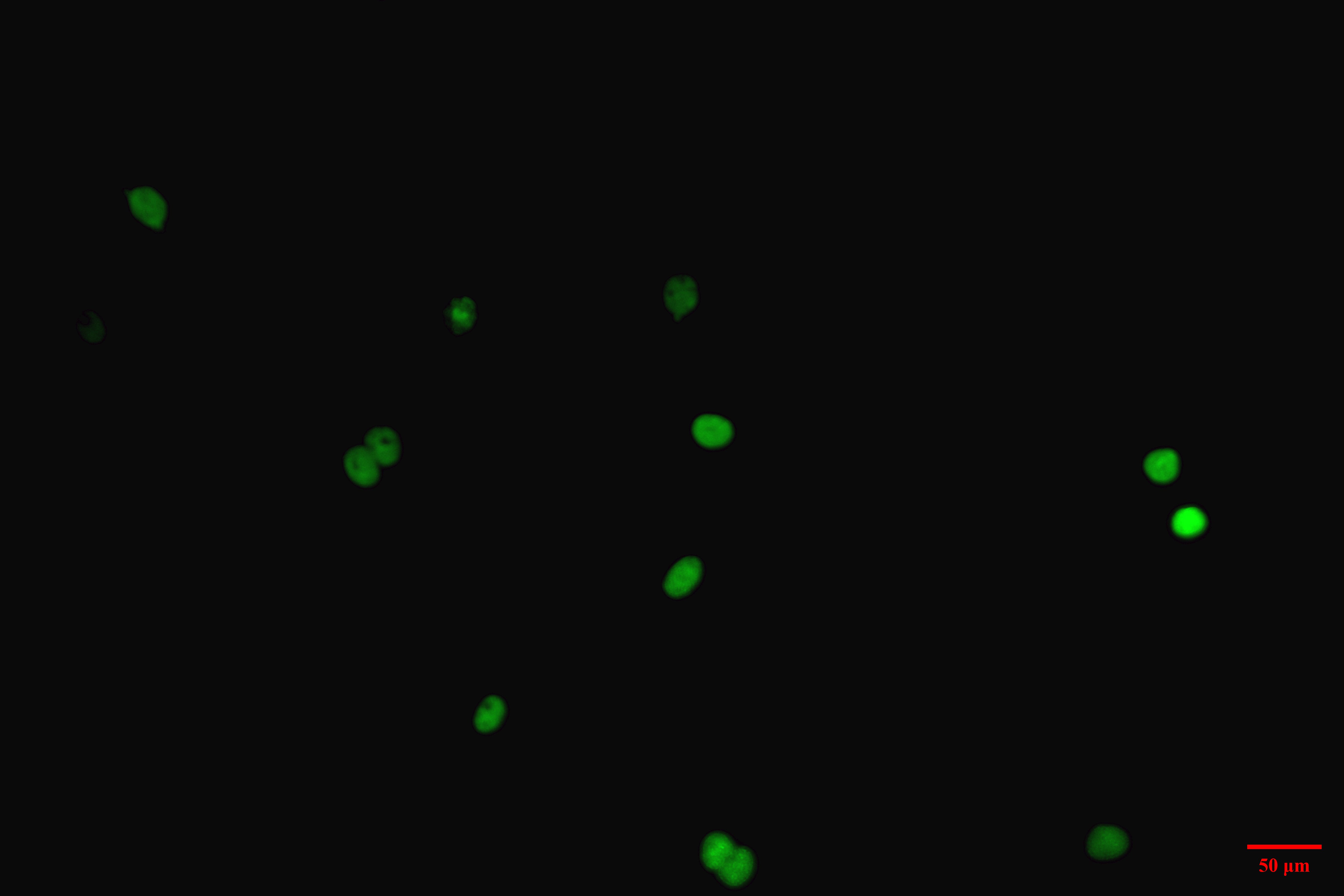

Supplement: Supplementary file 1 [file biomolecules-16-01059-s001.zip › File S1/Figure 6-8-11 Western blot original drawing/Figure 11e/CoCl2+BEL(50μmolL )/EDU-50-2.jpg]

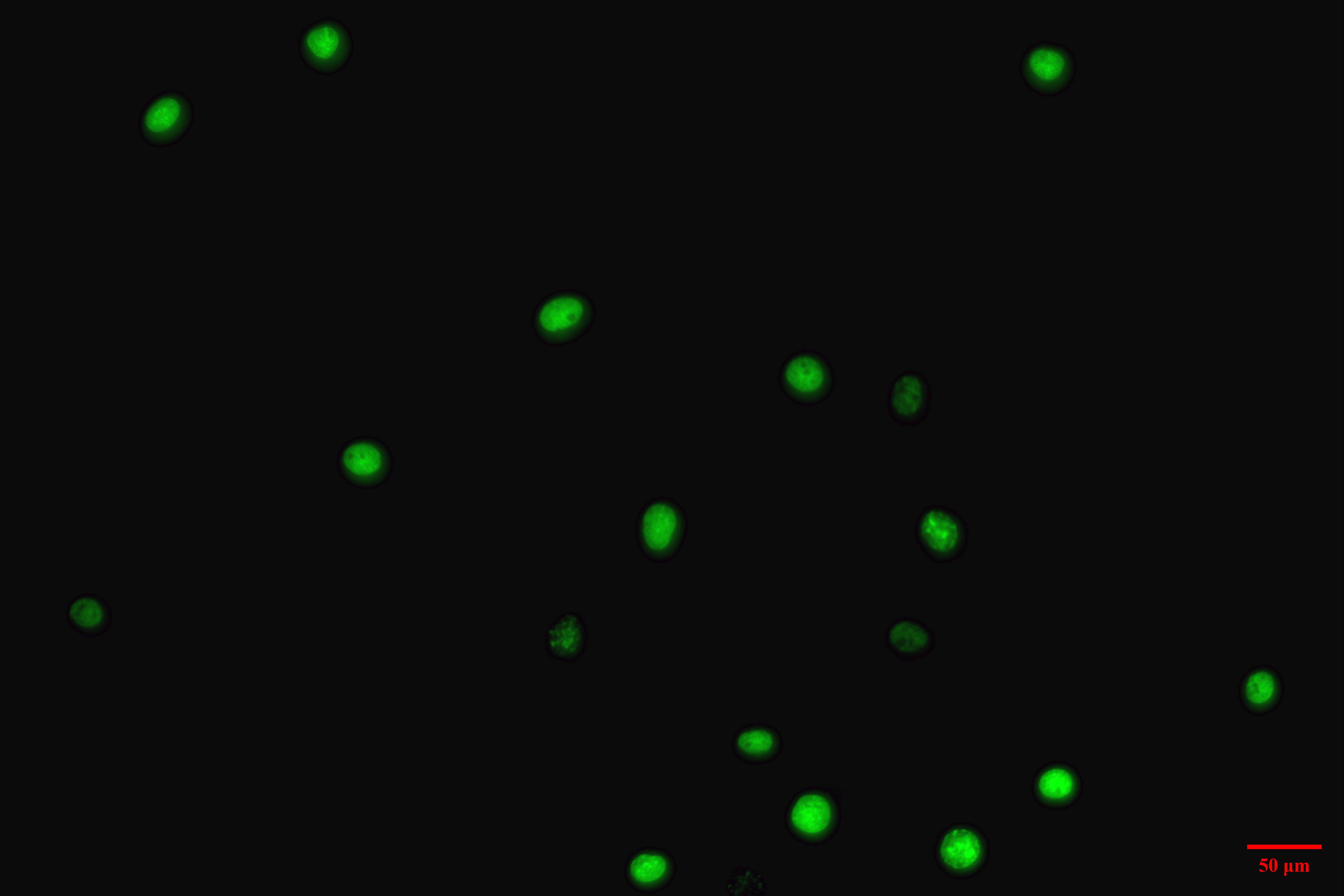

Supplement: Supplementary file 1 [file biomolecules-16-01059-s001.zip › File S1/Figure 6-8-11 Western blot original drawing/Figure 11e/CoCl2+BEL(50μmolL )/EDU-50-3.jpg]

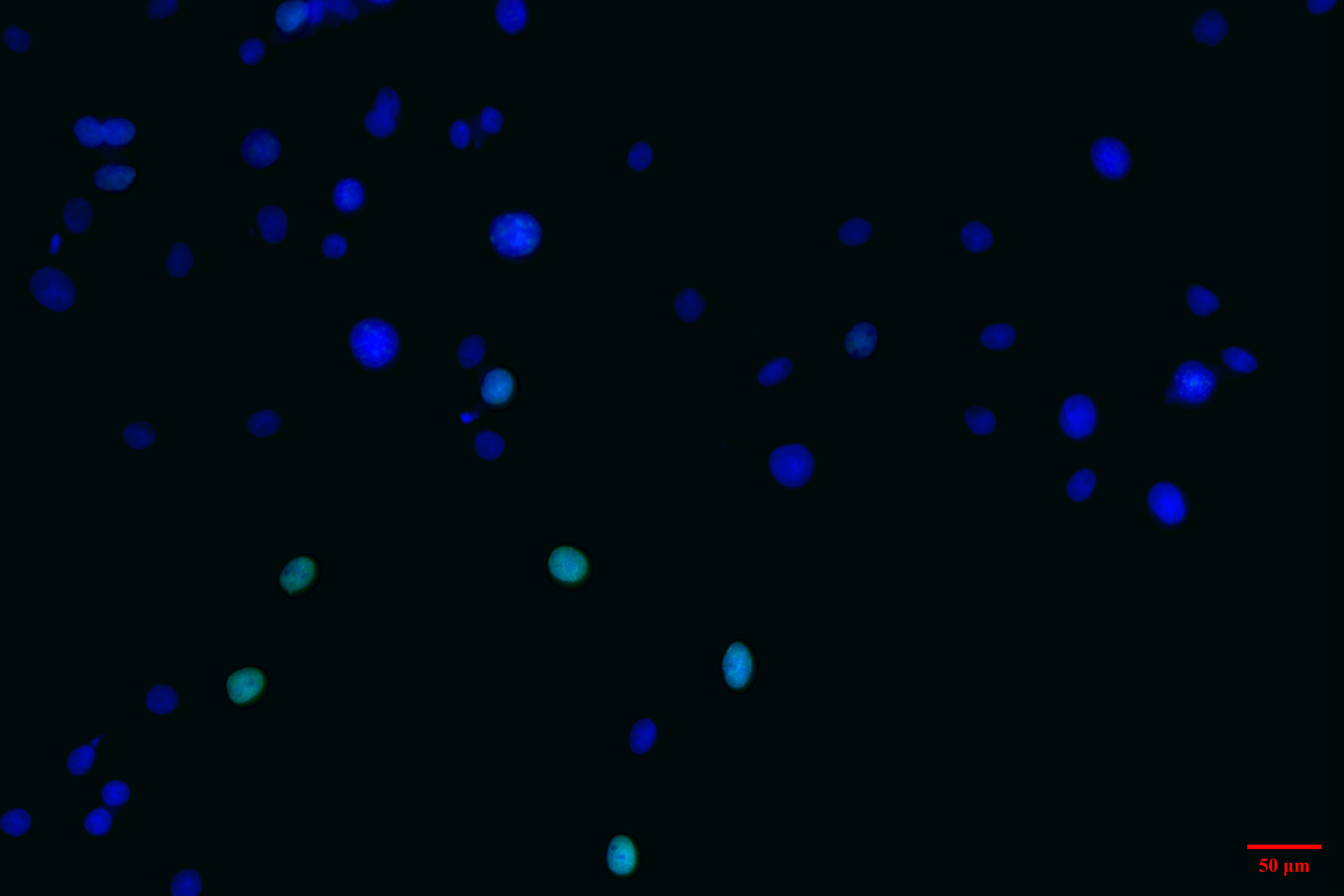

Supplement: Supplementary file 1 [file biomolecules-16-01059-s001.zip › File S1/Figure 6-8-11 Western blot original drawing/Figure 11e/CoCl2+BEL(50μmolL )/Merge-50-1.jpg]

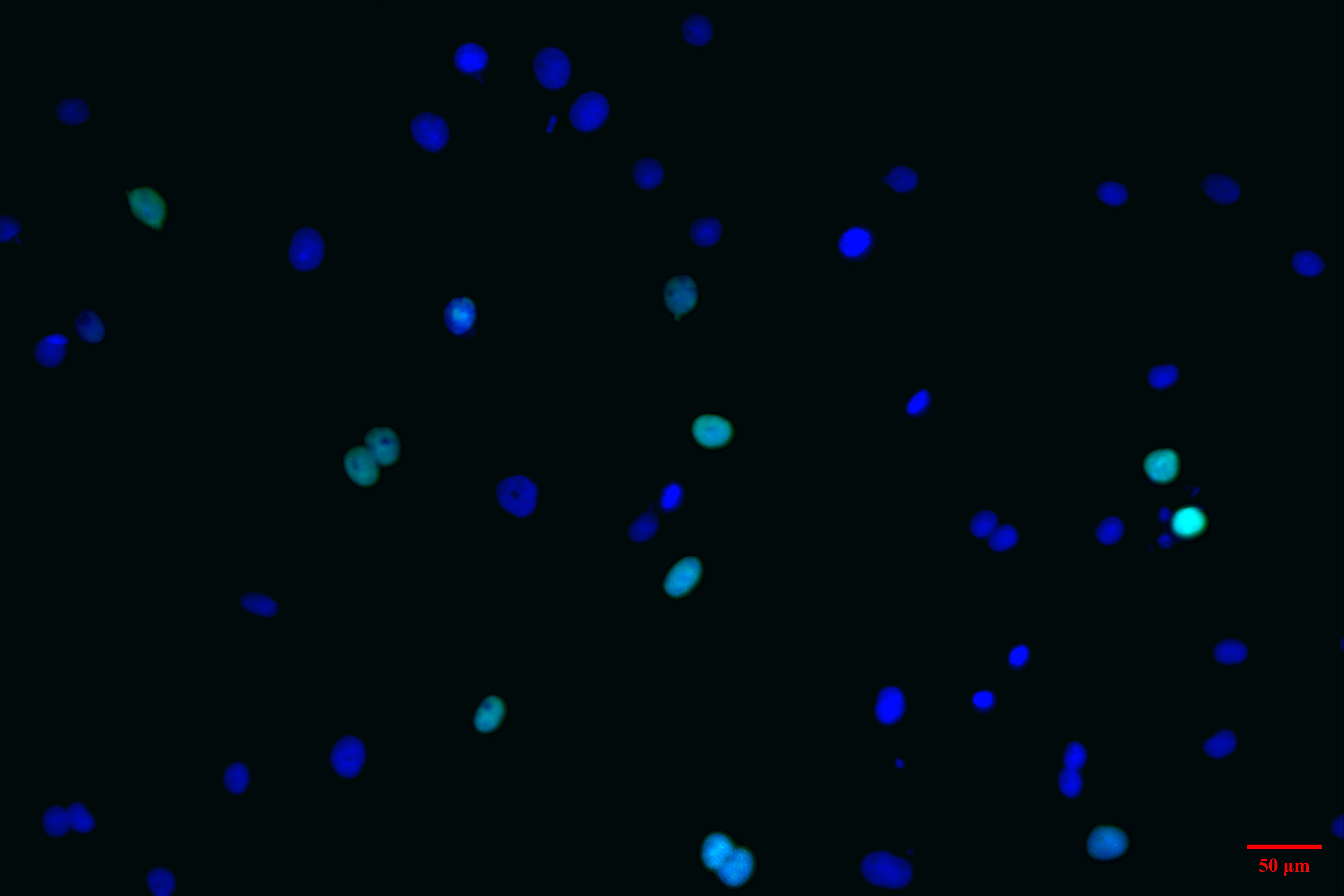

Supplement: Supplementary file 1 [file biomolecules-16-01059-s001.zip › File S1/Figure 6-8-11 Western blot original drawing/Figure 11e/CoCl2+BEL(50μmolL )/Merge-50-2.jpg]

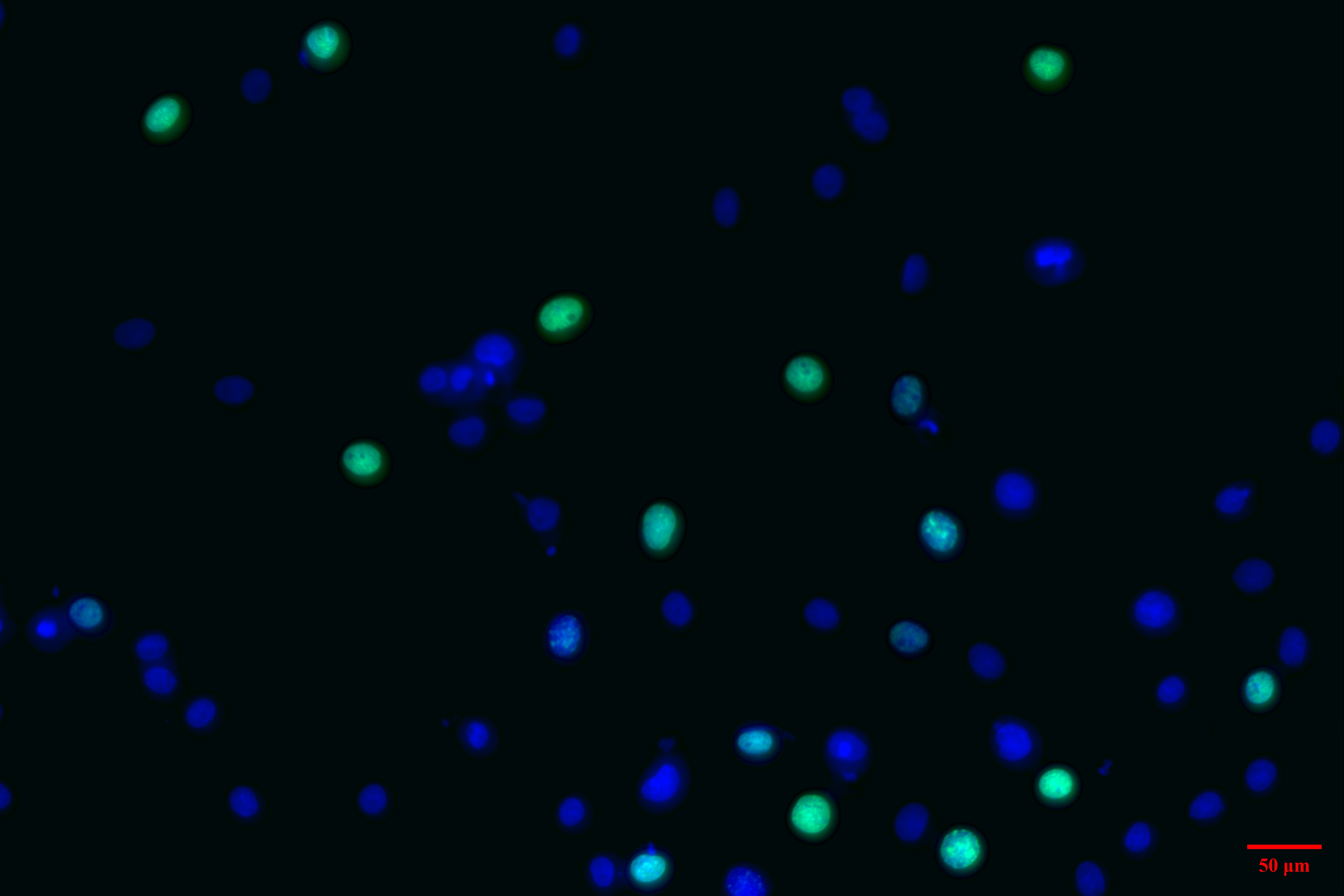

Supplement: Supplementary file 1 [file biomolecules-16-01059-s001.zip › File S1/Figure 6-8-11 Western blot original drawing/Figure 11e/CoCl2+BEL(50μmolL )/Merge-50-3.jpg]

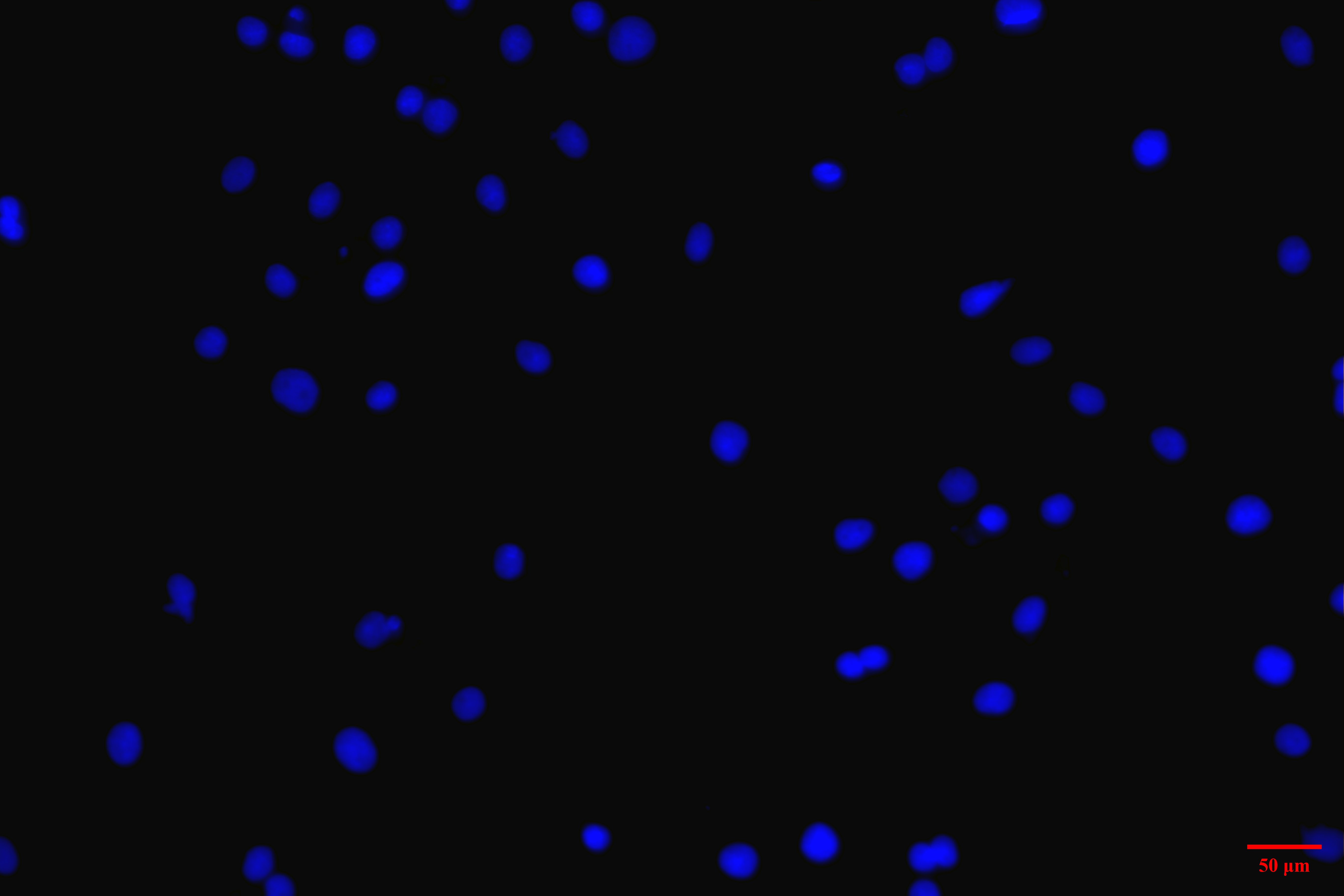

Supplement: Supplementary file 1 [file biomolecules-16-01059-s001.zip › File S1/Figure 6-8-11 Western blot original drawing/Figure 11e/CoCl2+BEL(80μmolL )/DAPI-80-1.jpg]

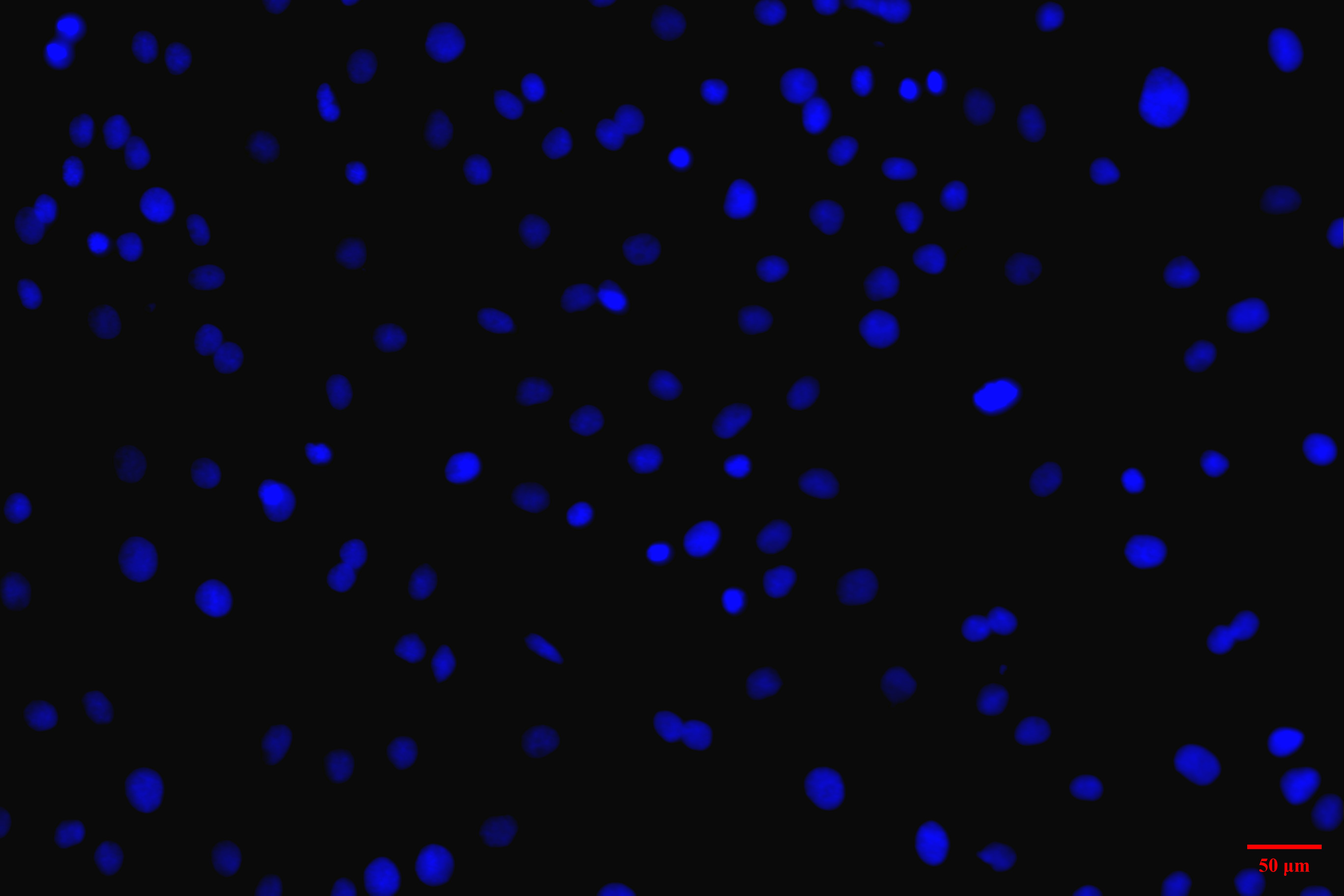

Supplement: Supplementary file 1 [file biomolecules-16-01059-s001.zip › File S1/Figure 6-8-11 Western blot original drawing/Figure 11e/CoCl2+BEL(80μmolL )/DAPI-80-2.jpg]

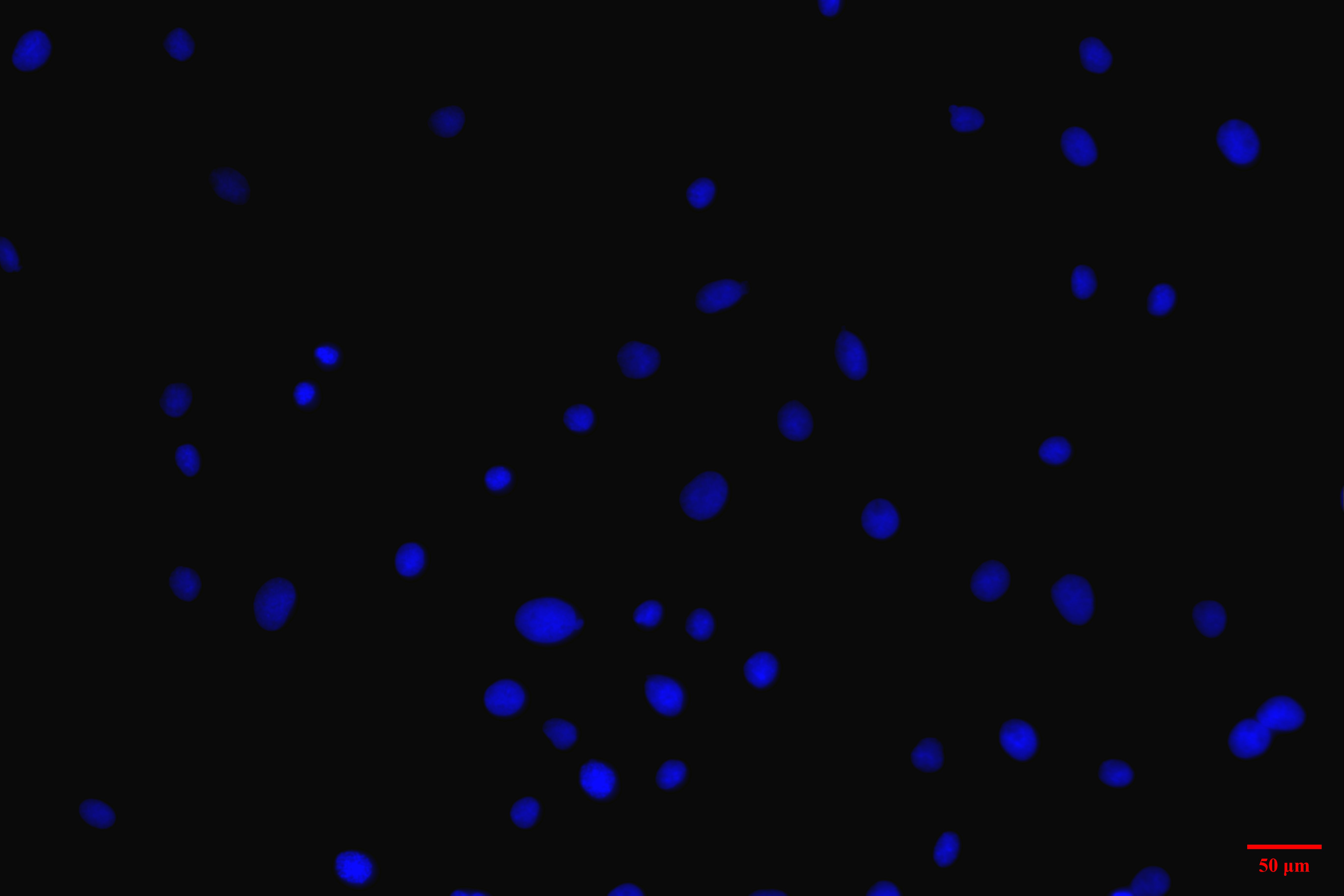

Supplement: Supplementary file 1 [file biomolecules-16-01059-s001.zip › File S1/Figure 6-8-11 Western blot original drawing/Figure 11e/CoCl2+BEL(80μmolL )/DAPI-80-3.jpg]

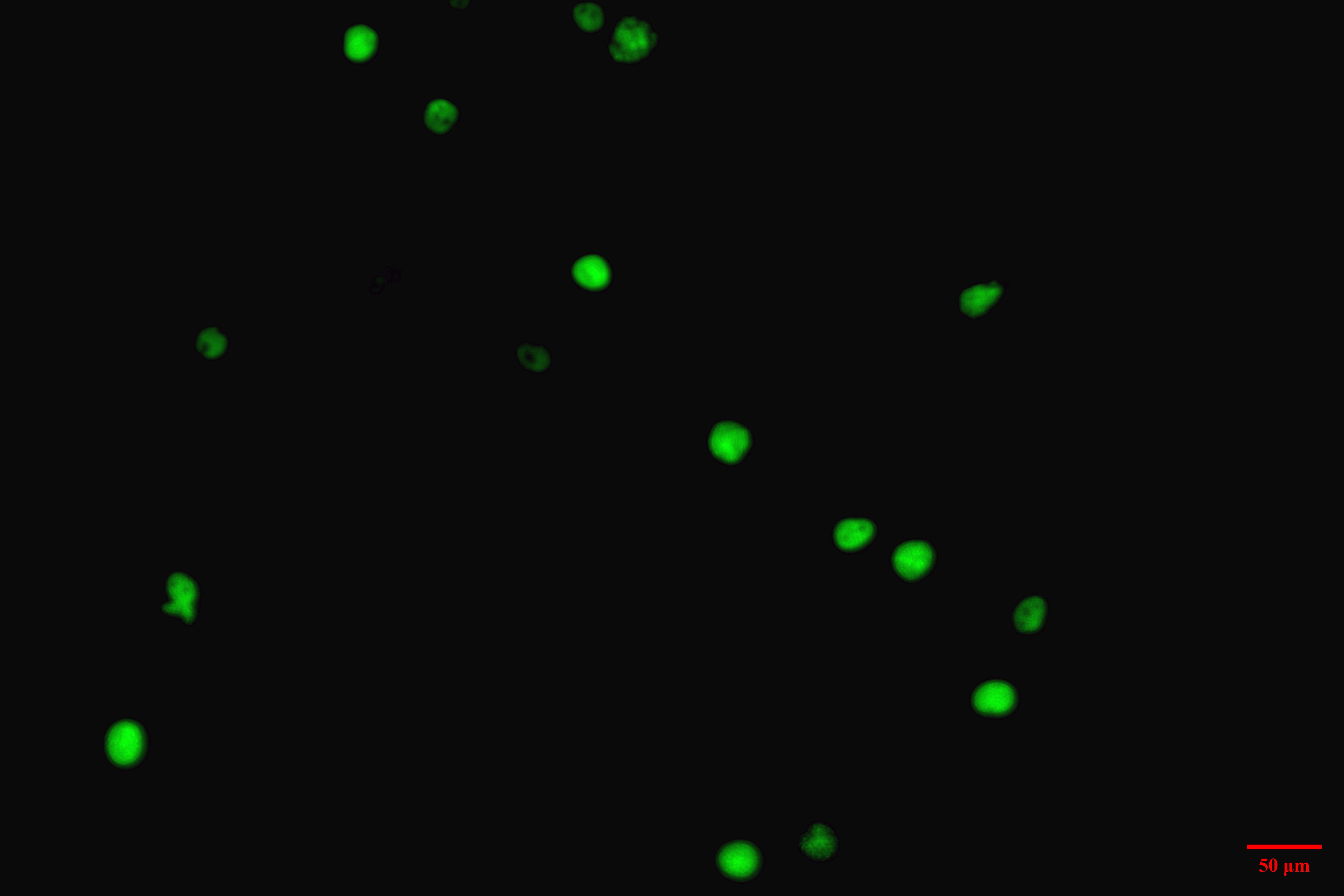

Supplement: Supplementary file 1 [file biomolecules-16-01059-s001.zip › File S1/Figure 6-8-11 Western blot original drawing/Figure 11e/CoCl2+BEL(80μmolL )/EDU-80-1.jpg]

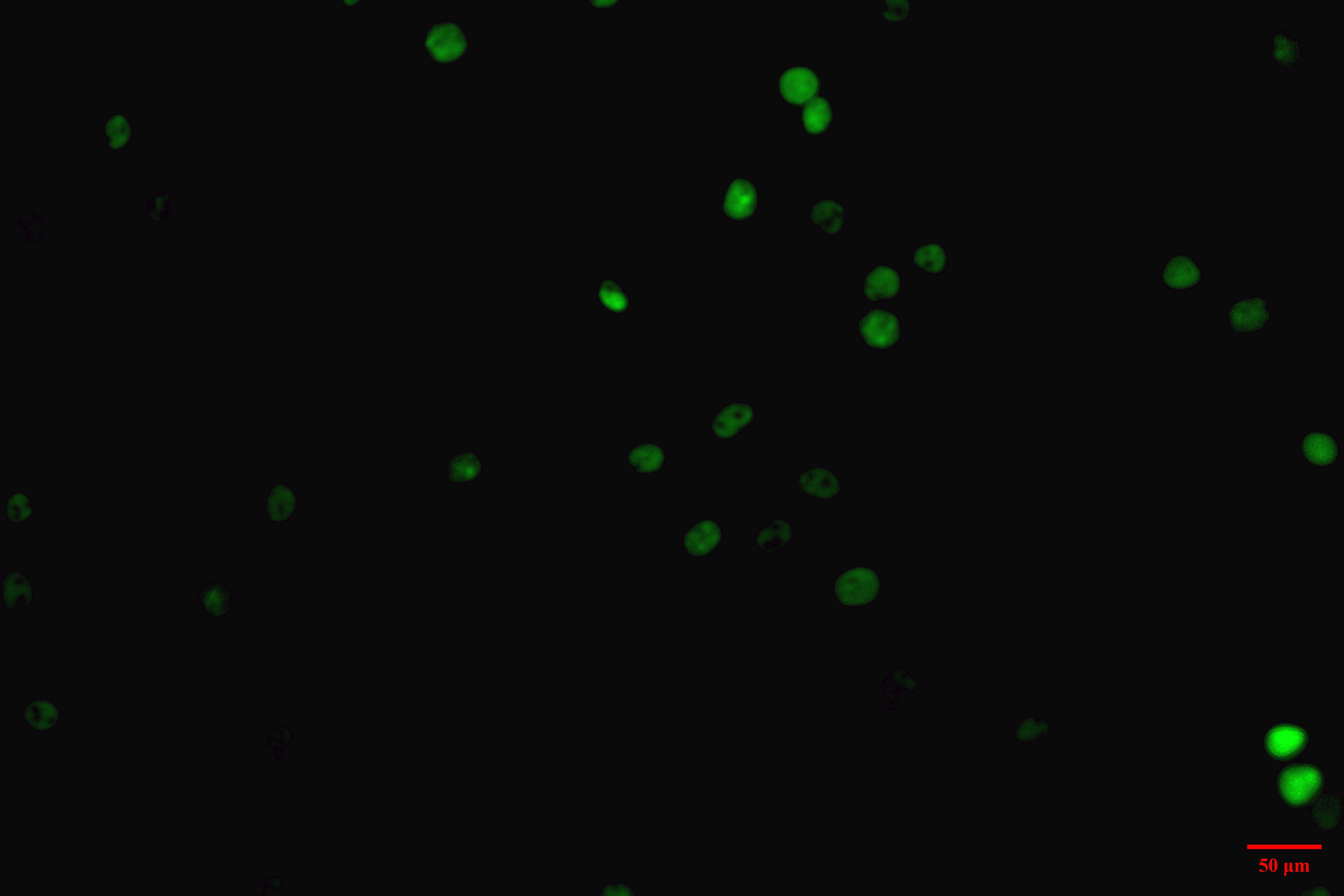

Supplement: Supplementary file 1 [file biomolecules-16-01059-s001.zip › File S1/Figure 6-8-11 Western blot original drawing/Figure 11e/CoCl2+BEL(80μmolL )/EDU-80-2.jpg]

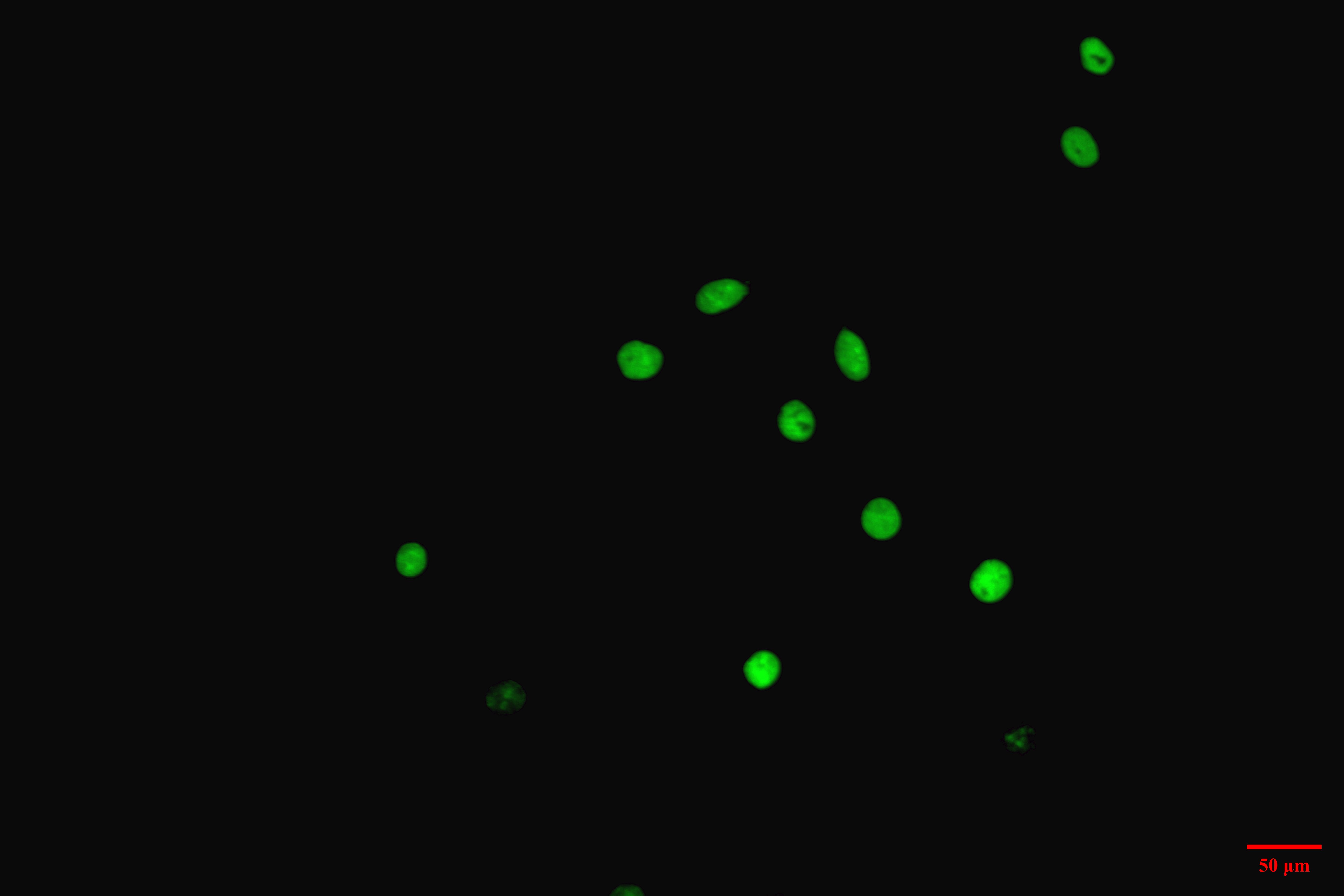

Supplement: Supplementary file 1 [file biomolecules-16-01059-s001.zip › File S1/Figure 6-8-11 Western blot original drawing/Figure 11e/CoCl2+BEL(80μmolL )/EDU-80-3.jpg]

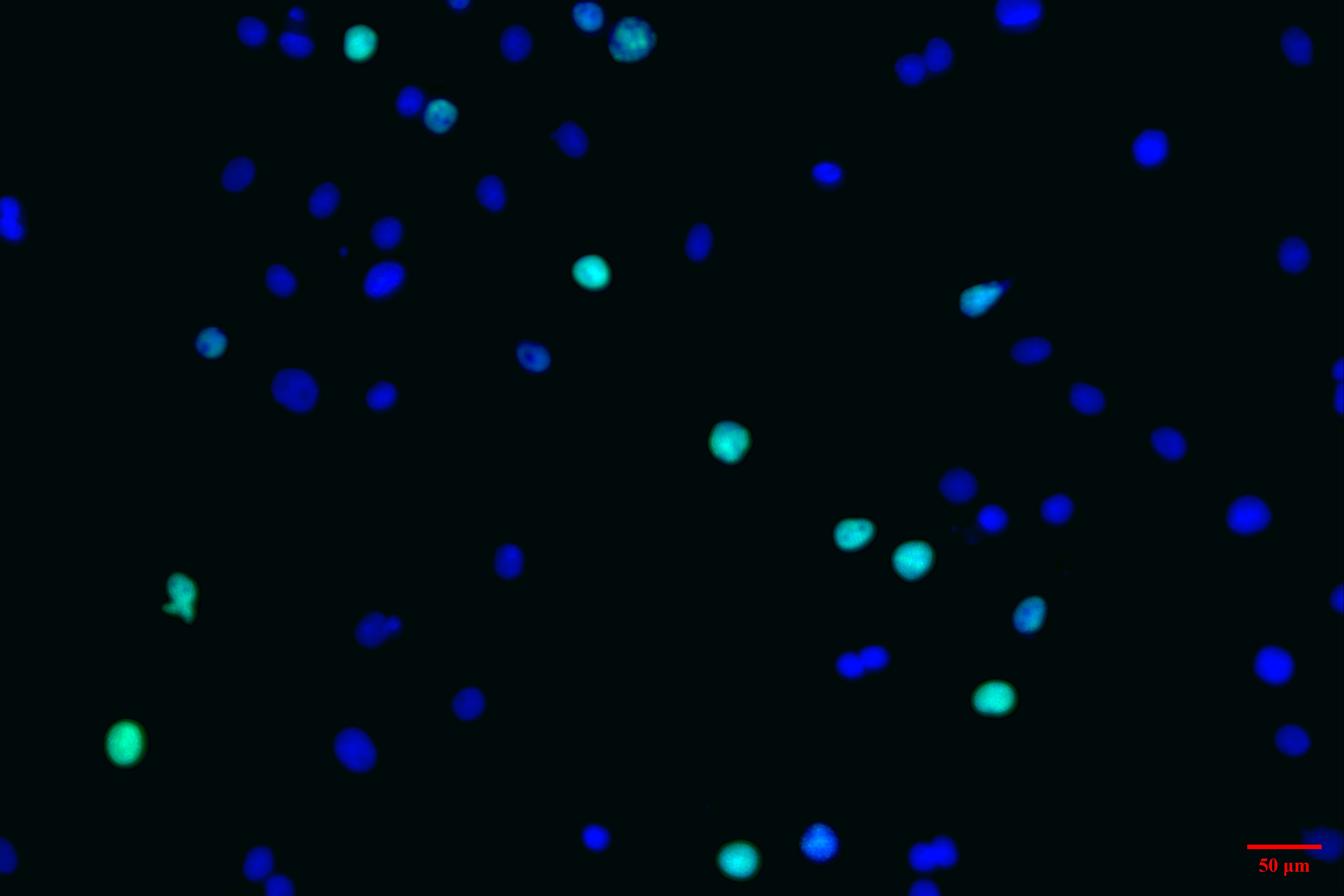

Supplement: Supplementary file 1 [file biomolecules-16-01059-s001.zip › File S1/Figure 6-8-11 Western blot original drawing/Figure 11e/CoCl2+BEL(80μmolL )/Merge-80-1.jpg]

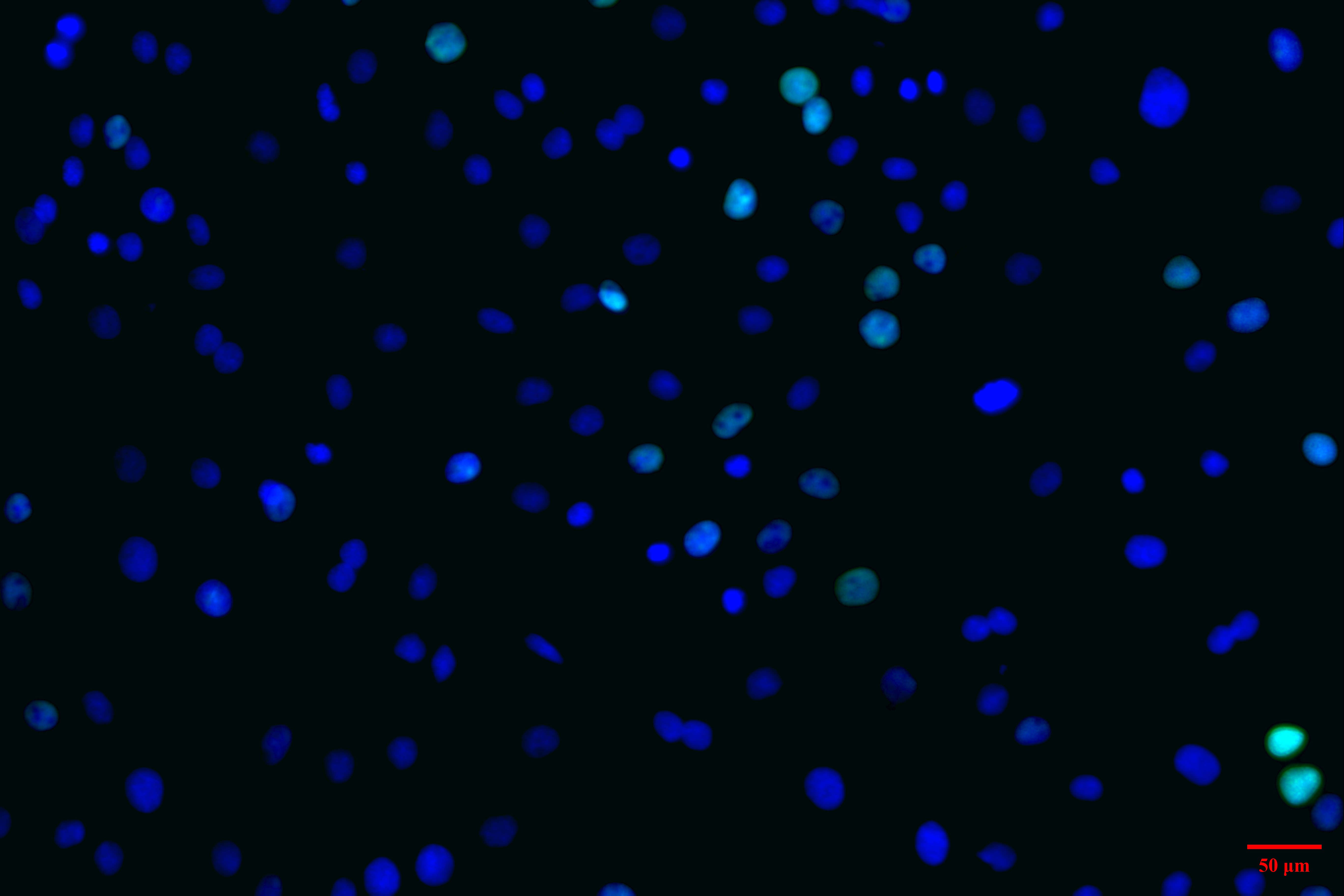

Supplement: Supplementary file 1 [file biomolecules-16-01059-s001.zip › File S1/Figure 6-8-11 Western blot original drawing/Figure 11e/CoCl2+BEL(80μmolL )/Merge-80-2.jpg]

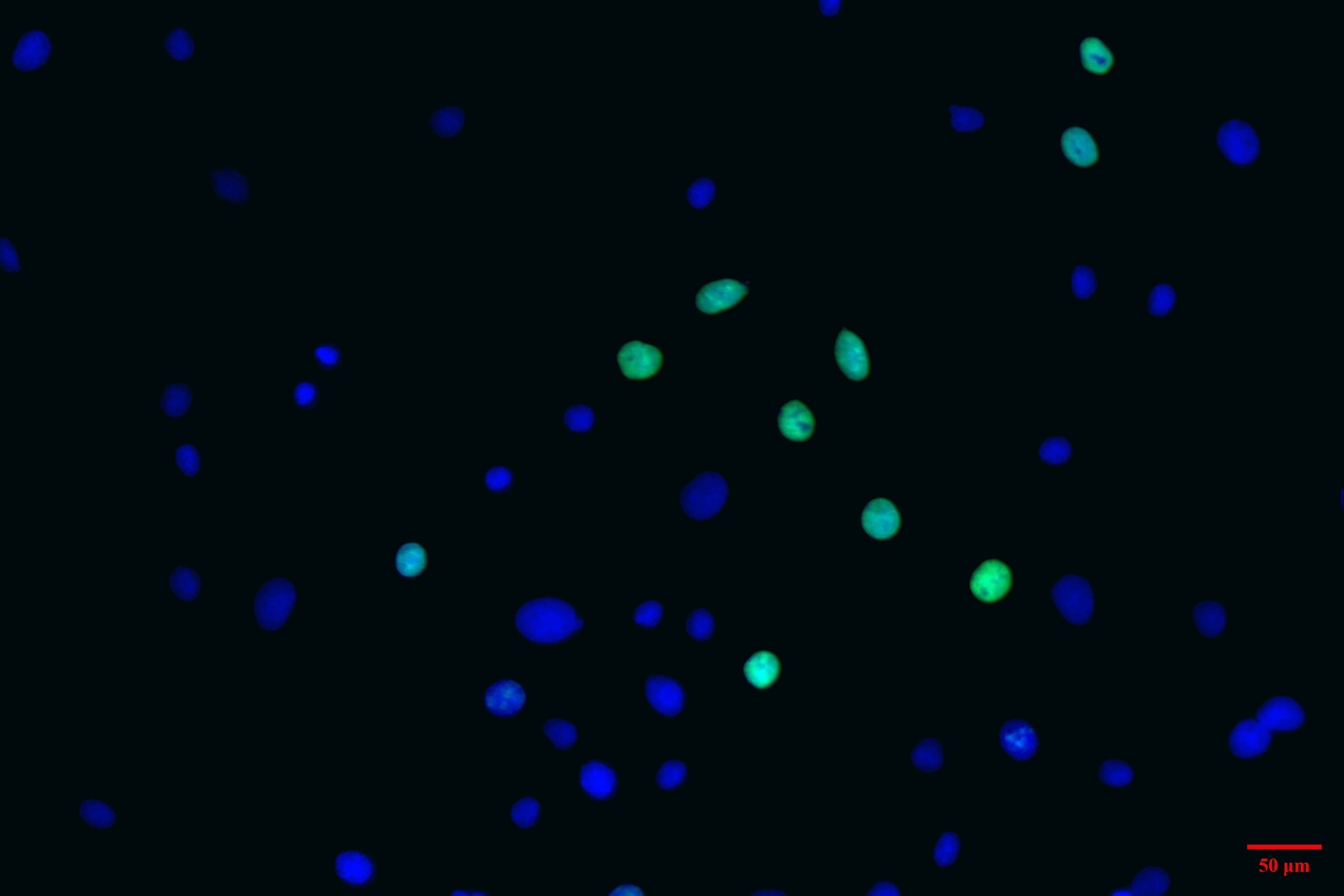

Supplement: Supplementary file 1 [file biomolecules-16-01059-s001.zip › File S1/Figure 6-8-11 Western blot original drawing/Figure 11e/CoCl2+BEL(80μmolL )/Merge-80-3.jpg]

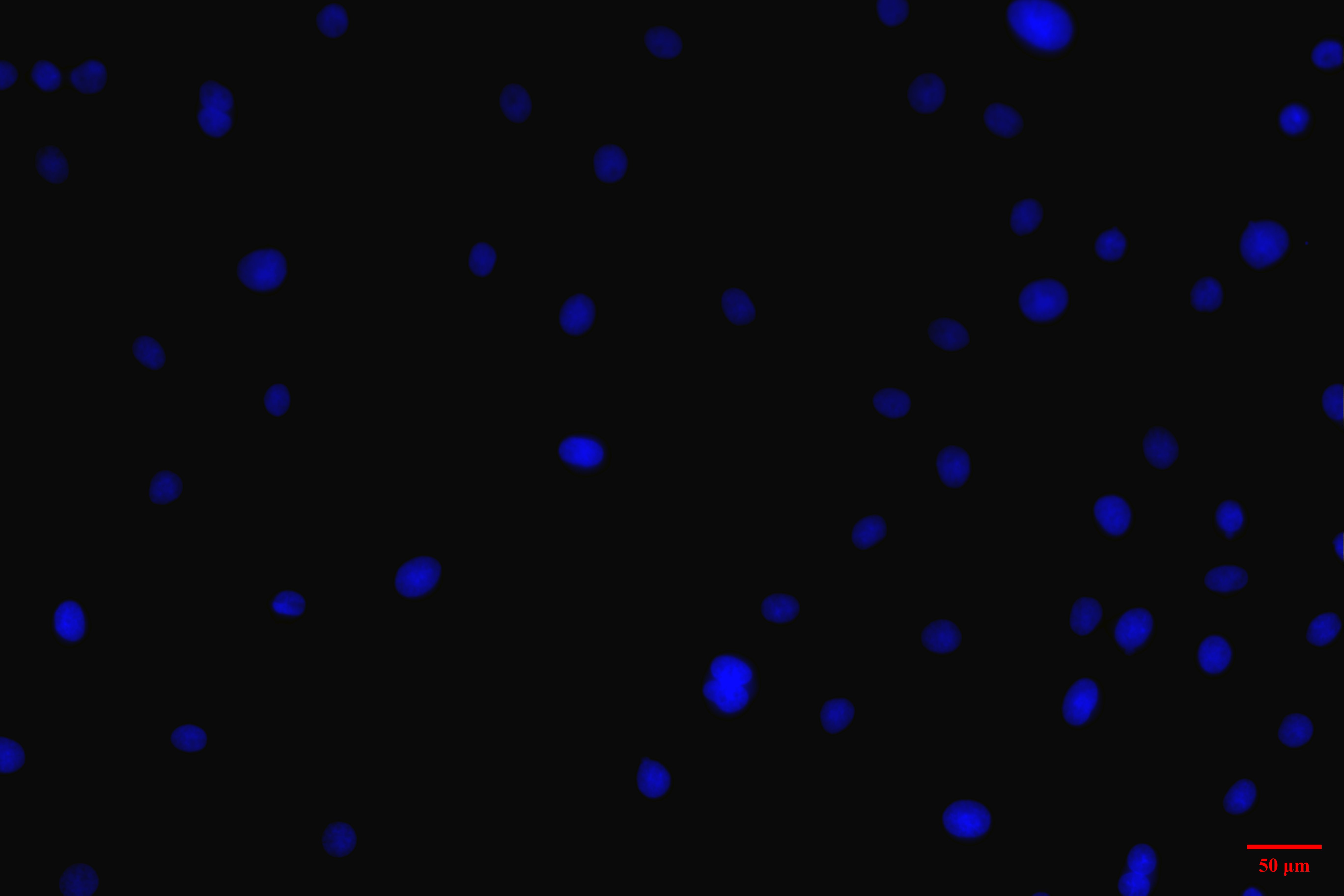

Supplement: Supplementary file 1 [file biomolecules-16-01059-s001.zip › File S1/Figure 6-8-11 Western blot original drawing/Figure 11e/CoCl2+KC7F2/DAPI-KC7F2-1.jpg]

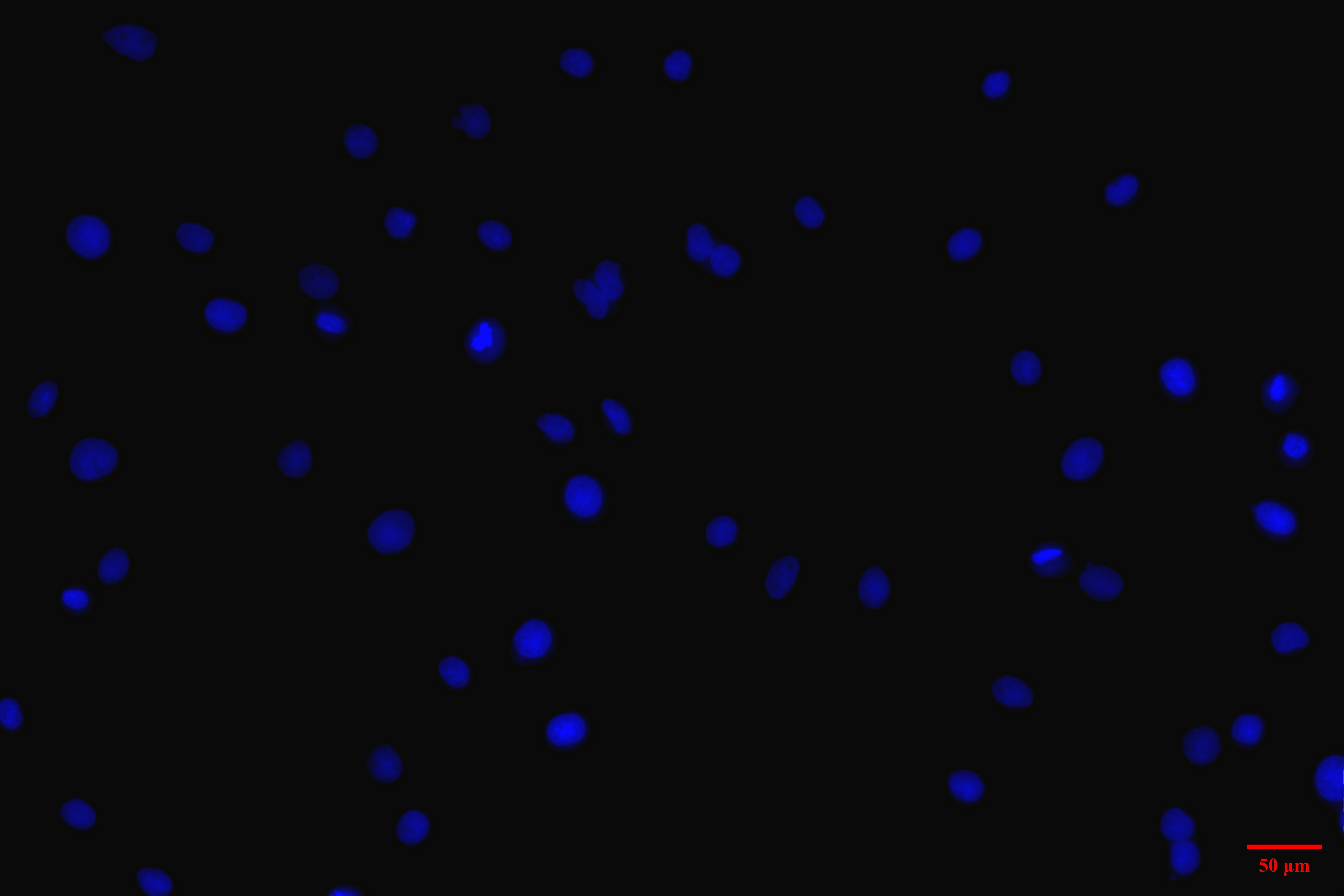

Supplement: Supplementary file 1 [file biomolecules-16-01059-s001.zip › File S1/Figure 6-8-11 Western blot original drawing/Figure 11e/CoCl2+KC7F2/DAPI-KC7F2-2.jpg]

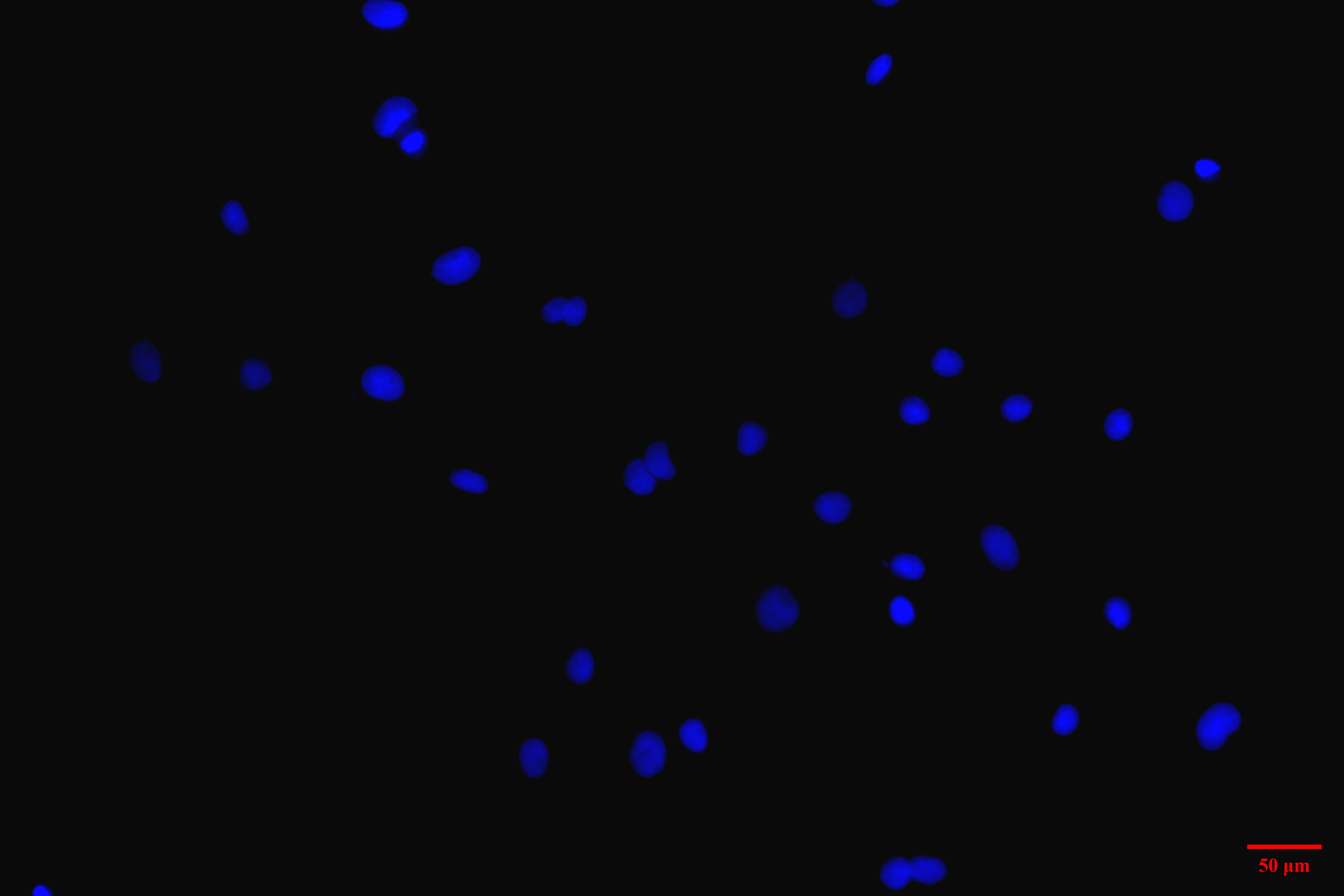

Supplement: Supplementary file 1 [file biomolecules-16-01059-s001.zip › File S1/Figure 6-8-11 Western blot original drawing/Figure 11e/CoCl2+KC7F2/DAPI-KC7F2-3.jpg]

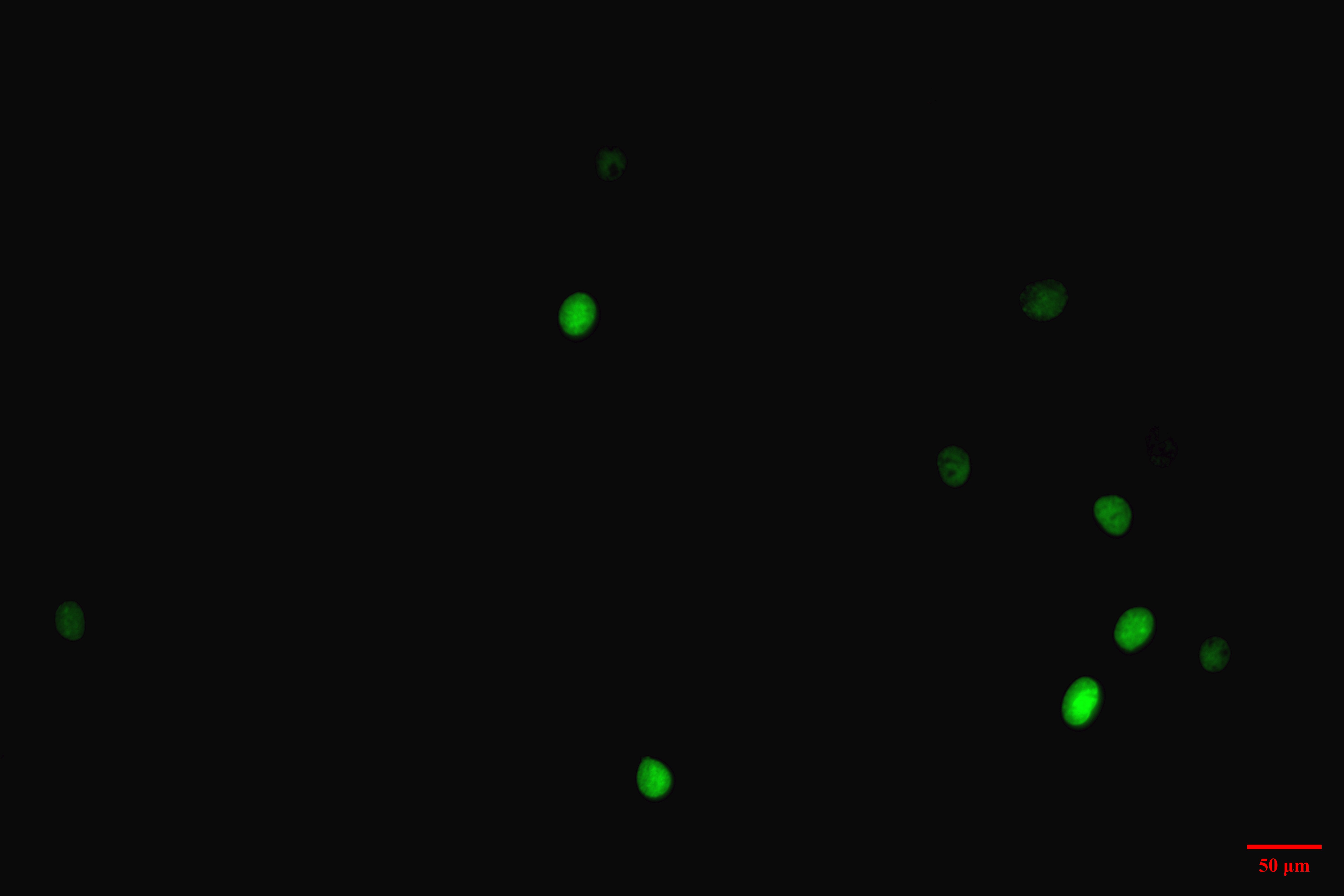

Supplement: Supplementary file 1 [file biomolecules-16-01059-s001.zip › File S1/Figure 6-8-11 Western blot original drawing/Figure 11e/CoCl2+KC7F2/EDU-KC7F2-1.jpg]

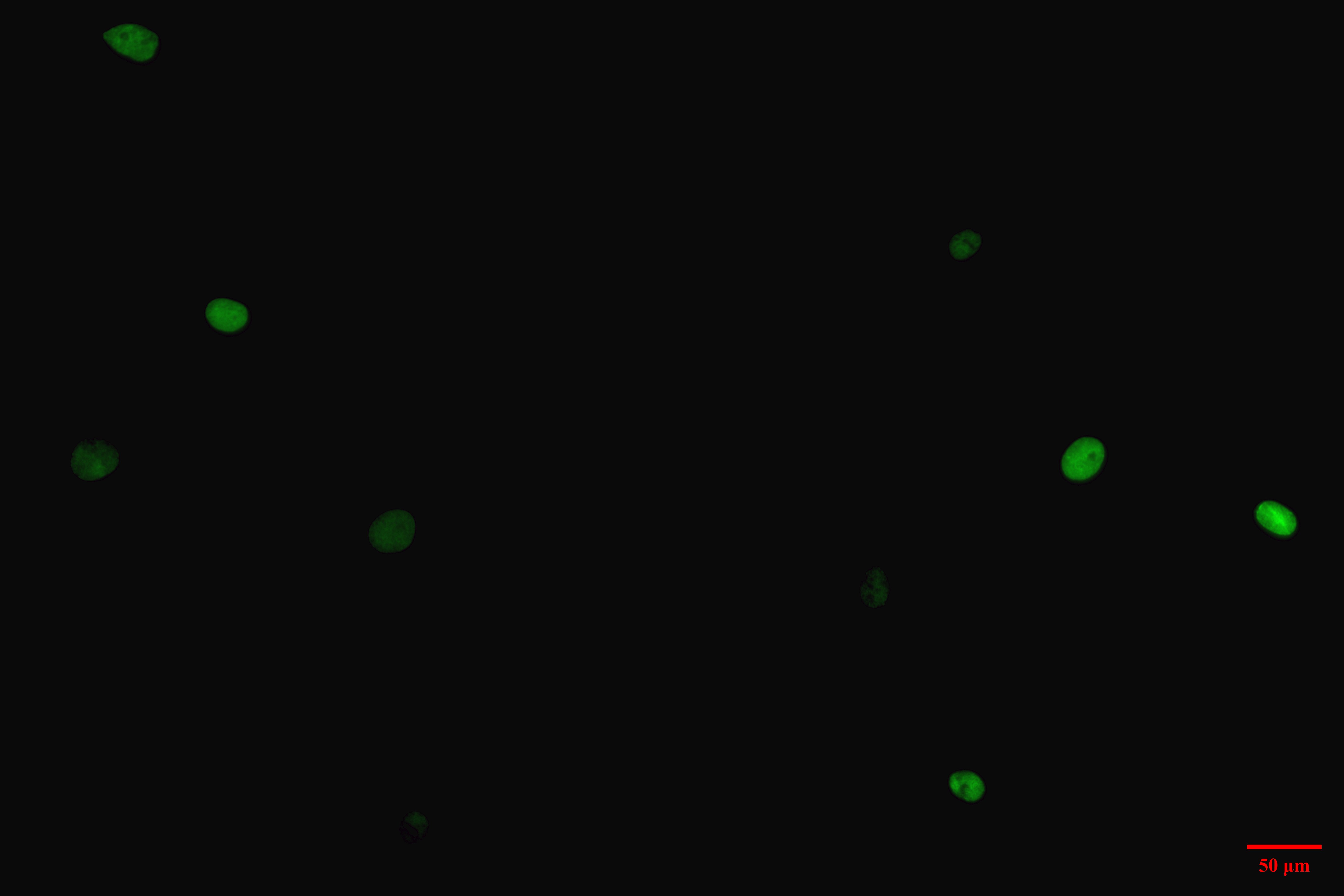

Supplement: Supplementary file 1 [file biomolecules-16-01059-s001.zip › File S1/Figure 6-8-11 Western blot original drawing/Figure 11e/CoCl2+KC7F2/EDU-KC7F2-2.jpg]

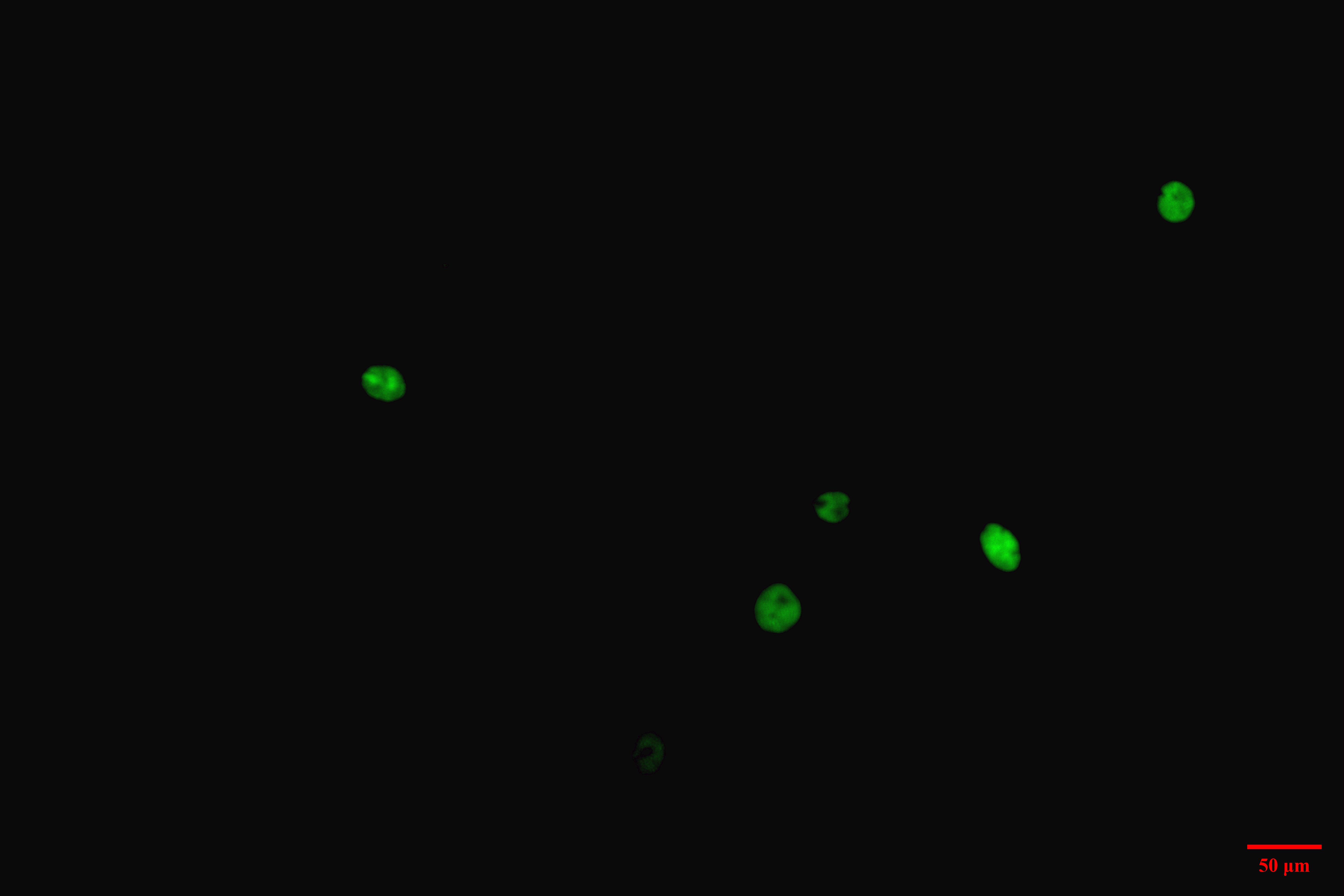

Supplement: Supplementary file 1 [file biomolecules-16-01059-s001.zip › File S1/Figure 6-8-11 Western blot original drawing/Figure 11e/CoCl2+KC7F2/EDU-KC7F2-3.jpg]

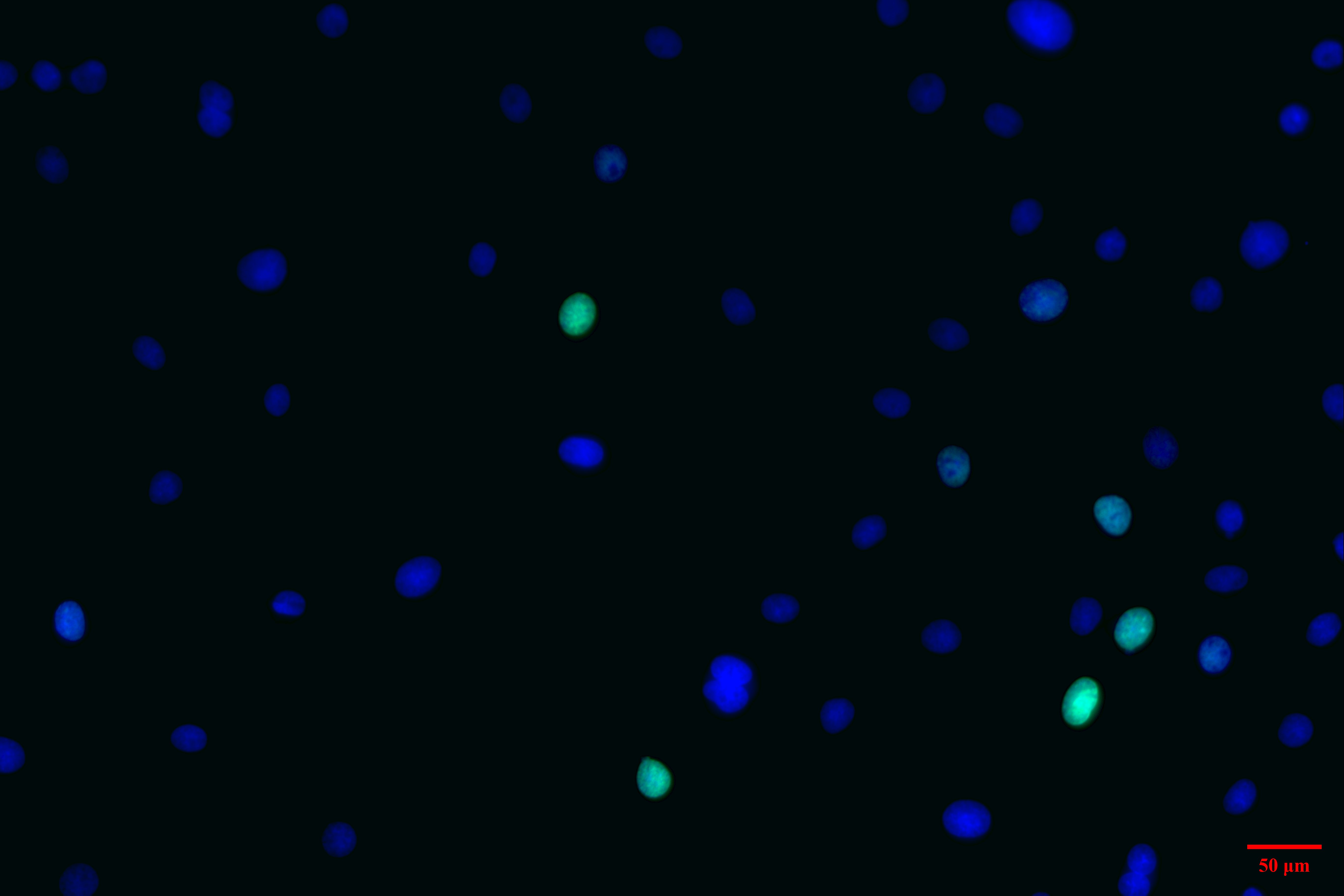

Supplement: Supplementary file 1 [file biomolecules-16-01059-s001.zip › File S1/Figure 6-8-11 Western blot original drawing/Figure 11e/CoCl2+KC7F2/Merge-KC7F2-1.jpg]

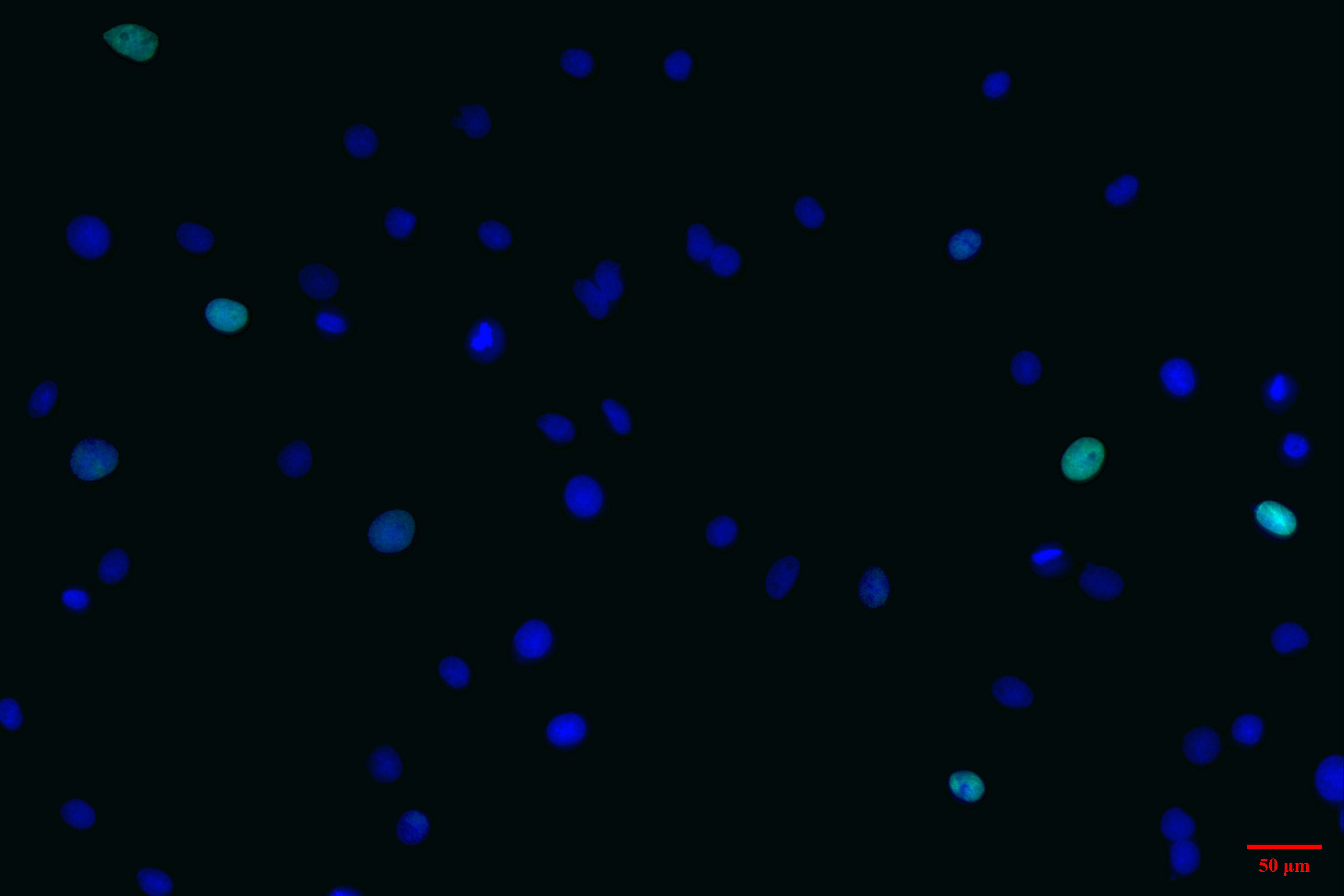

Supplement: Supplementary file 1 [file biomolecules-16-01059-s001.zip › File S1/Figure 6-8-11 Western blot original drawing/Figure 11e/CoCl2+KC7F2/Merge-KC7F2-2.jpg]

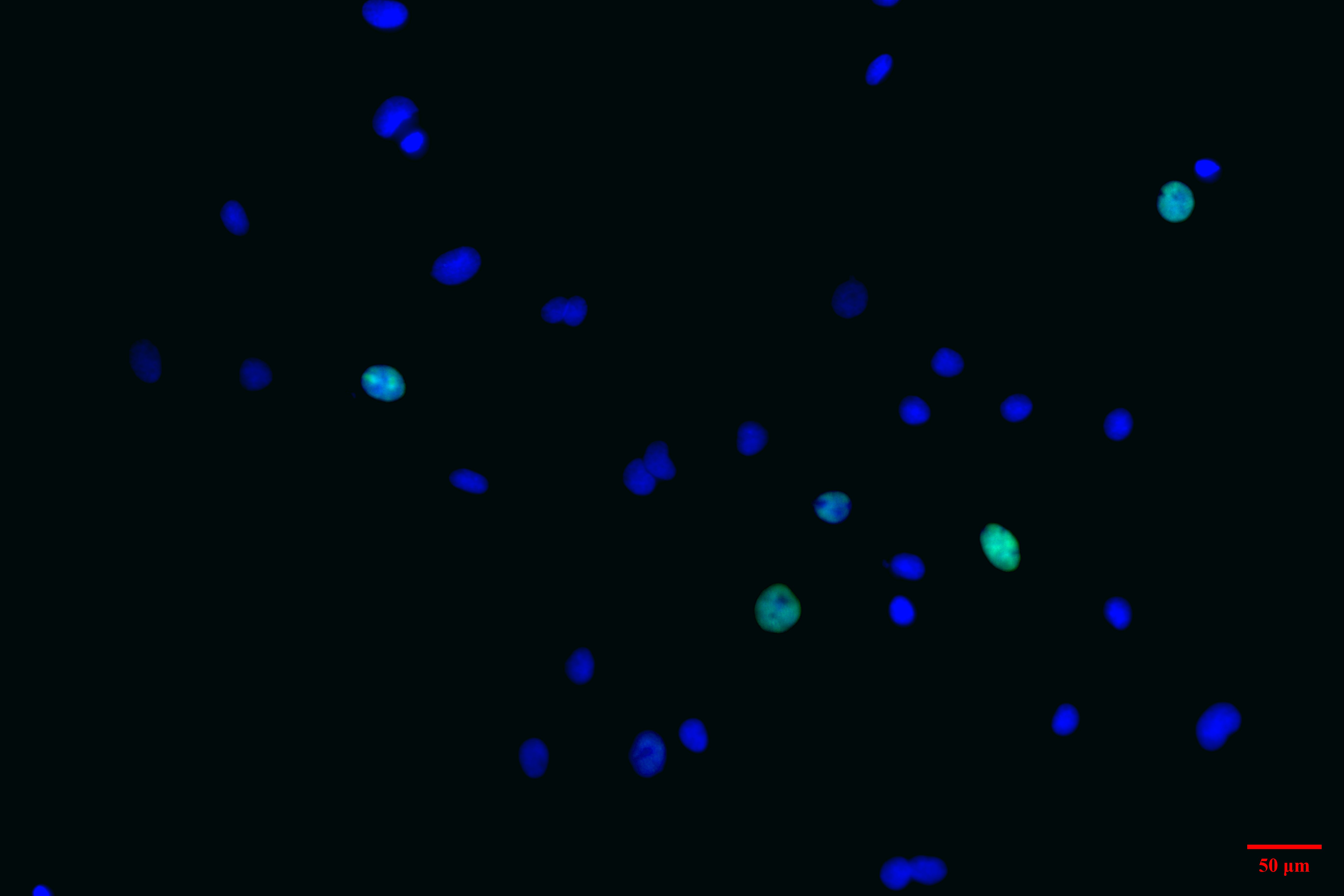

Supplement: Supplementary file 1 [file biomolecules-16-01059-s001.zip › File S1/Figure 6-8-11 Western blot original drawing/Figure 11e/CoCl2+KC7F2/Merge-KC7F2-3.jpg]

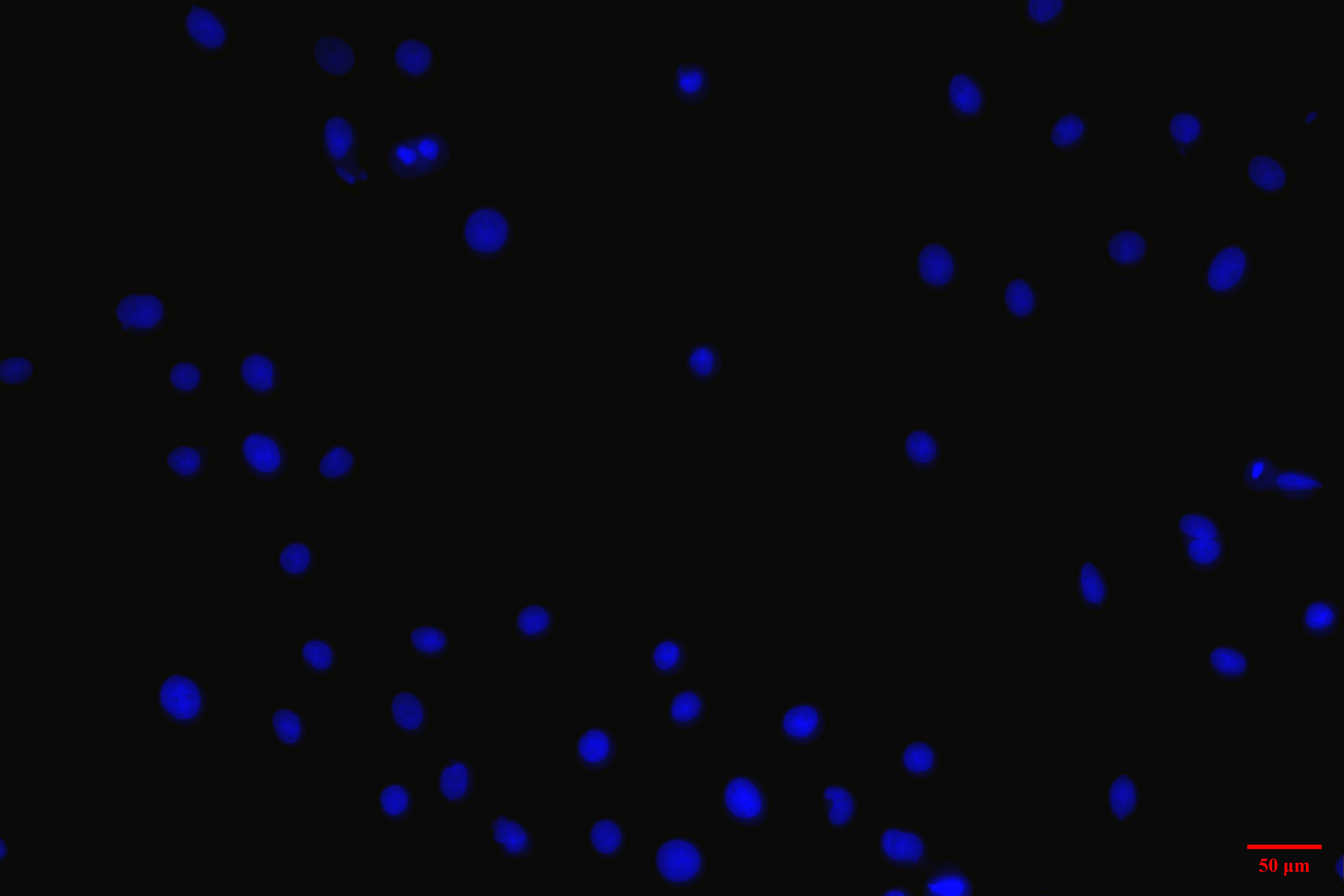

Supplement: Supplementary file 1 [file biomolecules-16-01059-s001.zip › File S1/Figure 6-8-11 Western blot original drawing/Figure 11e/CoCl2+NBI31772/DAPI-NBI-1.jpg]
